# Supplementary material for: Difluorocarbene-induced [1,2]- and [2,3]-Stevens rearrangement of tertiary amines
Source: Nat Commun. 2024 Jun 5;15:4794. doi: 10.1038/s41467-024-49054-x (PMC11153565; doi:10.1038/s41467-024-49054-x)
Supplement: Supplementary file 1 — Supplementary Information [file 41467_2024_49054_MOESM1_ESM.pdf]

Supplementary Information

for

Difluorocarbene-induced [1,2]- and [2,3]-Stevens rearrangement of tertiary amines

Jianke Su[1#], Yu Guo[1#], Chengbo Li[1] and Qiuling Song*[1,2,3,4]

[1] *Institute of Next Generation Matter Transformation, College of Material Sciences Engineering, Huaqiao University, Xiamen, Fujian, 361021, China*

[2]*Key Laboratory of Molecule Synthesis and Function Discovery, Fujian Province University, College of Chemistry at Fuzhou University, Fuzhou, Fujian, 350108, China*

[3]*State Key Laboratory of Organometallic Chemistry, Shanghai Institute of Organic Chemistry, Chinese Academy of Sciences, Shanghai 200032, China.*

[4]*School of Chemistry and Chemical Engineering, Henan Normal University, Xinxiang, Henan, 453007, China.*

**email:* qsong@fzu.edu.cn

These authors contributed equally： Jianke Su, Yu Guo

Table of Contents

1. Supplementary Methods 3

1.1 General information 3

1.2 General process 4

2. Supplementary Discussion 10

2.1 Optimization studies 10

2.2 Crystal data 14

2.3 Characterization data for products 15

2.4 NMR spectroscopic data 58

3. References 177

1. Supplementary Methods

1.1 General information

All chemicals were purchased from Leyan.com ($ClCF_2COONa$, $BrCF_2COONa$), Adamas Reagent (*N*-Allylmethylamine, K_3PO_4), Macklin Reagent (Rb_2CO_3), Energy chemical company ($BrCF_2COOEt$, $BrCF_2PO(OEt)_2$), Bide Pharmatech Ltd ($TMSCF_2Br$,), J&K SCIENTIFIC LTD (ICF_2COOEt)and Shang Fluoro Company ($ClCF_2H$). Unless otherwise stated, all experiments were conducted in a sealed tube under argon atmosphere. Reactions were monitored by TLC or GC-MS analysis. Flash column chromatography was performed over silica gel (200-300 mesh).

^{1}H-NMR and ^{13}C-NMR spectra were recorded in $CDCl_3$ and DMSO-d_6 on a Bruker Avance 500 spectrometer (500 MHz ^{1}H, 125 MHz ^{13}C (CPD), 470 MHz ^{19}F) at room temperature. Chemical shifts were reported in ppm on the scale relative to $CDCl_3$ (δ = 7.26 for ^{1}H-NMR, δ = 77.00 for ^{13}C-NMR) as an internal reference. Coupling constants (*J*) were reported in Hertz (Hz).

1.2 General process

General process 1：Preparation of tertiary amines 1 and 4

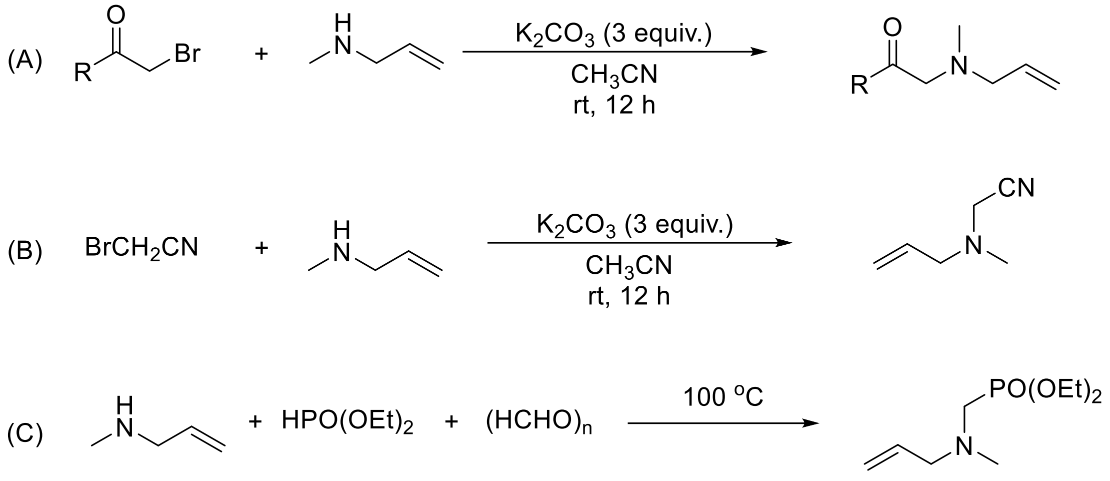

General process[1-2]: To a solution of the *N*-allyl methylamine (15 mmol, 3 equiv) and K_2CO_3 (15 mmol, 3 equiv) in CH_3CN (10 mL) was added halo alkane (5 mmol, 1 equiv) at 0 ºC. The reaction was stirred for 12h at room temperature. Then, water (20 mL) was added and the mixture was extracted with EtOAc (3 × 30 mL). Then, the organic layer was dried, concentrated, and purified by flash column chromatography (silica gel, petroleum ether: EtOAc =3:1, v/v) to give the desired products.

General process[3]: A mixture of *N*-allyl methylamine (10.0 mmol), diethyl phosphate (10.0 mmol), and paraformaldehyde (10.0 mmol) was stirred at 100 º C for 2 h. The mixture was cooled to room temperature and added diethyl ether (20 mL) and potassium carbonate (967 mg, 7.0 mmol). The mixture was stirred at room temperature for 2 h, and the organic layer was separated, dried over anhydrous sodium sulfate, and concentrated, and purified by flash column chromatography (silica gel, petroleum ether: EtOAc =1:1, v/v) to give the desired products.

General process 2：Preparation of allylamine 4 and benzylamine 5

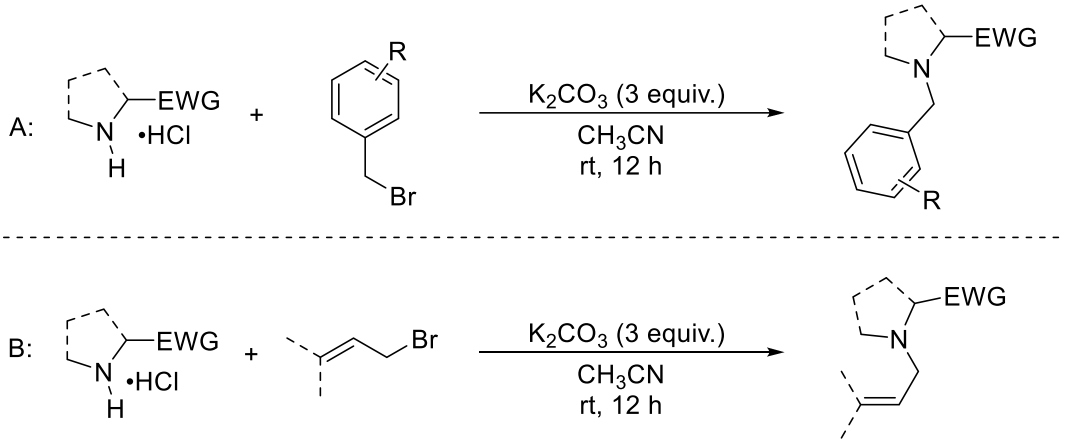

General process[4]: To a solution of the secondary amine (15 mmol, 3 equiv) and K_2CO_3 (15 mmol, 3 equiv) in CH_3CN (10 mL) was added benzyl bromide or allyl bromide (5 mmol, 1 equiv) at 0 °C. The reaction was stirred for 12h at room temperature. Then, water (20 mL) was added and the mixture was extracted with EtOAc (3 × 30 mL). Then, the organic layer was dried, concentrated, and purified by flash column chromatography (silica gel, petroleum ether: EtOAc =3:1, v/v) to give the desired products.

General process 3：Preparation of propargylamine 8

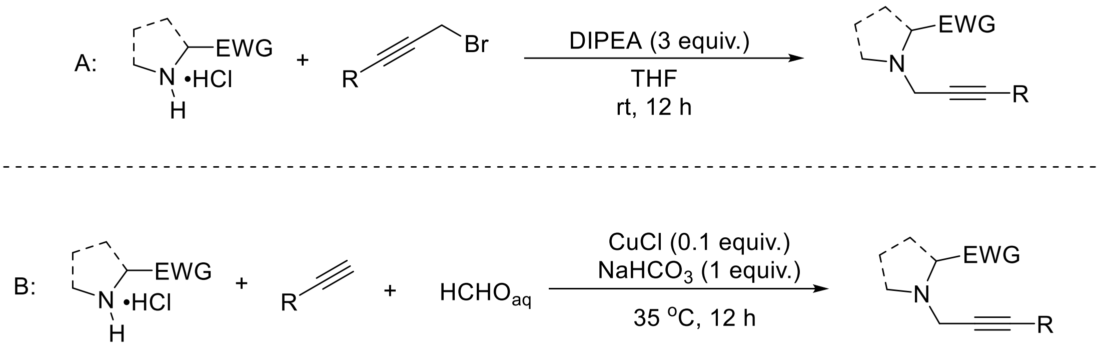

General process 3A[5]**:** To a solution of proline ester hydrochloride salt (5 mmol, 1.0 equiv) in THF (10 mL) was successively added DIPEA (15 mmol, 3.0 equiv) and propargyl bromide (5.5 mmol, 1.1 equiv) at 0 °C. The reaction mixture was stirred at room temperature for 12 h, then quenched with saturated NH_4Cl aqueous solution and the mixture was extracted with EtOAc (3 × 30 mL). Then, the organic layer was dried, concentrated, and purified by flash column chromatography (silica gel, petroleum ether: EtOAc =3:1, v/v) to give the desired products.

General process 3B[5]**:** A mixture of proline ester hydrochloride salt (5 mmol, 1.0 equiv), $NaHCO_3$ (5 mmol, 1.0 equiv), CuCl (0.5 mmol, 0.1 equiv) were added to a Schlenk tube equipped with a

magnetic stirring bar. The vessel was evacuated and filled with argon (three cycles). alkyne (6.5 mmol, 1.3 equiv), and CH_2O (6.5 mmol, 1.3 equiv, 37% in H_2O) was stirred at 35 °C for 12 h., then quenched with saturated NH_4Cl aqueous solution and the mixture was extracted with EtOAc (3 × 30 mL). Then, the organic layer was dried, concentrated, and purified by flash column chromatography (silica gel, petroleum ether: EtOAc =3:1, v/v) to give the desired products.

General process 4: The process of difluorocarbene-induced Stevens rearrangement of allylic tertiary amines

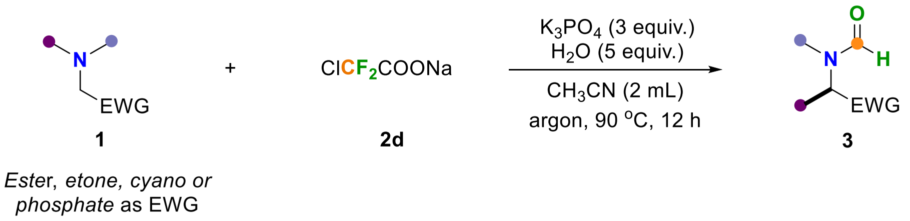

In air, tertiary amines **1** (1 eq, 0.2 mmol), $ClCF_2COONa$ (1.5 eq, 0.3 mmol) and K_3PO_4 (3 eq, 0.6 mmol) were added to a Schlenk tube equipped with a magnetic stirring bar. The vessel was evacuated and filled with argon (three cycles). CH_3CN (2 mL) and H_2O (5 eq, 1 mmol) was added by syringe under argon atmosphere. The resulting reaction mixture was stirred vigorously at 90 °C for 12 h. Upon completion of the reaction, the solvent was evaporated under reduced pressure and the residue was purified by flash column chromatography (silica gel, petroleum ether: EtOAc =2:1, v/v) to give the desired products.

General process 5: The process of difluorocarbene-induced allylic and benzyl Stevens rearrangement of tertiary amines

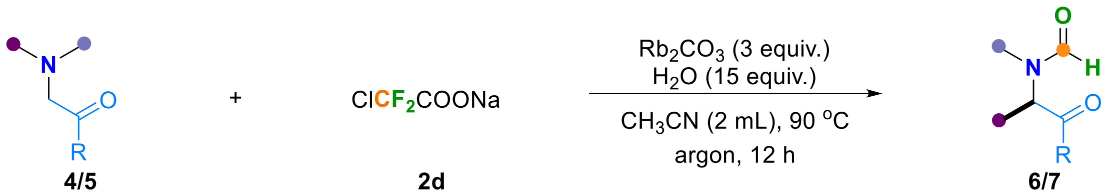

In air, allylic amines **4** or benzyl amines **5** (1 eq, 0.2 mmol), $ClCF_2COONa$ (1.5 eq, 0.3 mmol) and Rb_2CO_3 (3 eq, 0.6 mmol) were added to a Schlenk tube equipped with a magnetic stirring bar. The vessel was evacuated and filled with argon (three cycles). CH_3CN (2 mL) and H_2O (15 eq, 3 mmol) was added by syringe under argon atmosphere. The resulting reaction mixture was stirred

vigorously at 90 ºC for 12 h. Upon completion of the reaction, the solvent was evaporated under reduced pressure and the residue was purified by flash column chromatography (silica gel, petroleum ether: EtOAc =2:1, v/v) to give the desired products.

General process 6: The process of difluorocarbene-induced propargyl Stevens rearrangement of tertiary amines

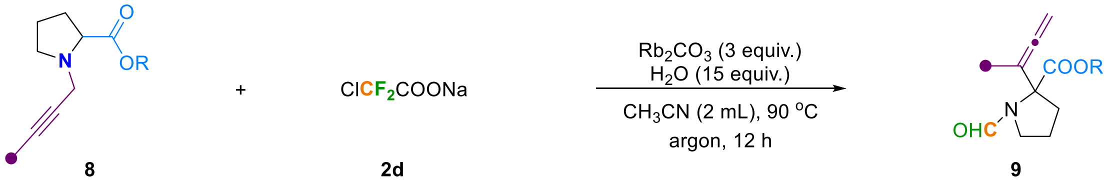

8 **2d** **9**

In air, propargyl tertiary amines **8** (1 eq, 0.2 mmol), $ClCF_2COONa$ (1.5 eq, 0.3 mmol) and Rb_2CO_3 (3 eq, 0.6 mmol) were added to a Schlenk tube equipped with a magnetic stirring bar. The vessel was evacuated and filled with argon (three cycles). CH_3CN (2 mL) and H_2O (15 eq, 3 mmol) was added by syringe under argon atmosphere. The resulting reaction mixture was stirred vigorously at 90 ºC for 12 h. Upon completion of the reaction, the solvent was evaporated under reduced pressure and the residue was purified by flash column chromatography (silica gel, petroleum ether: EtOAc =2:1, v/v) to give the desired products.

General process 7: The process of difluorocarbene-induced allylic Stevens rearrangement of the substrates derived from complex molecules

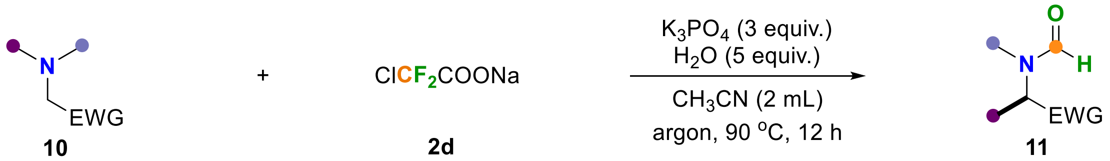

10 **2d** **11**

In air, tertiary amines **10** (1 eq, 0.2 mmol), $ClCF_2COONa$ (1.5 eq, 0.3 mmol) and K_3PO_4 (3 eq, 0.6 mmol) were added to a Schlenk tube equipped with a magnetic stirring bar. The vessel was evacuated and filled with argon (three cycles). CH_3CN (2 mL) and H_2O (5 eq, 1 mmol) was added by syringe under argon atmosphere. The resulting reaction mixture was stirred vigorously at 90 ºC for 12 h. Upon completion of the reaction, the solvent was evaporated under reduced pressure and the residue was purified by flash column chromatography (silica gel, petroleum ether: EtOAc =2:1, v/v) to give the desired products.

General process 8: Gram-scale synthesis of 3p

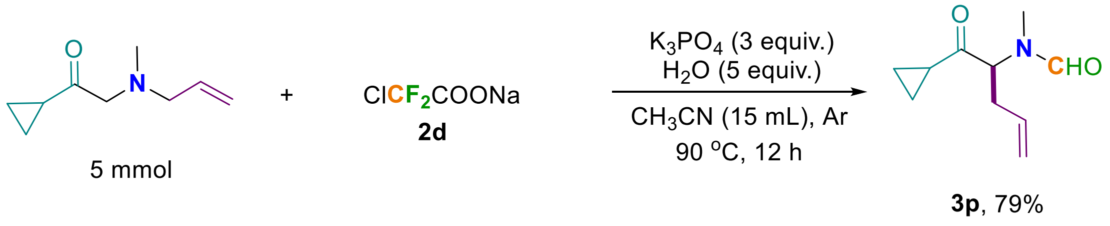

In air, tertiary amines **1** (1 eq, 5 mmol), $ClCF_2COONa$ (1.5 eq, 7.5 mmol) and K_3PO_4 (3 eq, 15 mmol) were added to a Schlenk tube equipped with a magnetic stirring bar. The vessel was evacuated and filled with argon (three cycles). CH_3CN (15 mL) and H_2O (5 eq, 25 mmol) was added by syringe under argon atmosphere. The resulting reaction mixture was stirred vigorously at 90 ºC for 24 h. Upon completion of the reaction, the solvent was evaporated under reduced pressure and the residue was purified by flash column chromatography (silica gel, petroleum ether: EtOAc =2:1, v/v) to give the desired products.

General process 9: Gram-scale synthesis of 7d and 9o

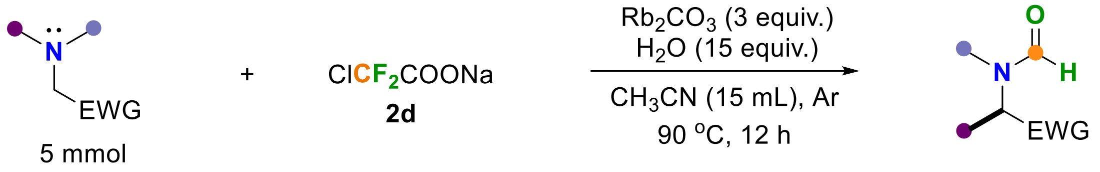

In air, tertiary amines (1 eq, 5 mmol), $ClCF_2COONa$ (1.5 eq, 7.5 mmol) and Rb_2CO_3 (3 eq, 15 mmol) were added to a Schlenk tube equipped with a magnetic stirring bar. The vessel was evacuated and filled with argon (three cycles). CH_3CN (15 mL) and H_2O (15 eq, 75 mmol) was added by syringe under argon atmosphere. The resulting reaction mixture was stirred vigorously at 90 ºC for 24 h. Upon completion of the reaction, the solvent was evaporated under reduced pressure and the residue was purified by flash column chromatography (silica gel, petroleum ether: EtOAc =2:1, v/v) to give the desired products.

General process 10: For synthesis of 12[6]

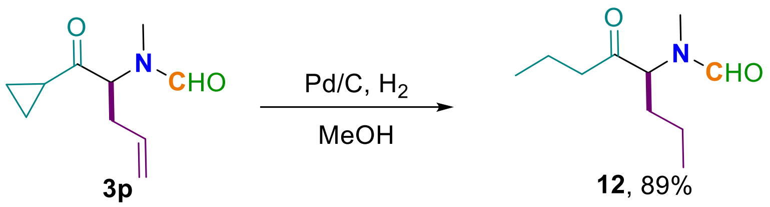

3p

12, 89%

To a Schlenk tube containing of the **3p** (0.2 mmol) and 2 mL of MeOH, Pd/C (10% w) was added. The tube was evacuated and refilled with H_2 with three times. MeOH (2 mL) was added by syringe under argon atmosphere. The reaction was refilled with hydrogen through a balloon. The resulting reaction mixture was stirred under the hydrogen atmosphere at room temperature for 12 h. After the reaction was completed, which was determined by TLC analysis. The resulting suspension was filtered through a plug of celite and the filter cake washed with DCM. The mixture was concentrated in vacuum and the residue was purified by flash column chromatography (silica gel, petroleum ether: EtOAc =2:1, v/v) to give the **12** as a yellow oil.

General process 11: For synthesis of 13[7]

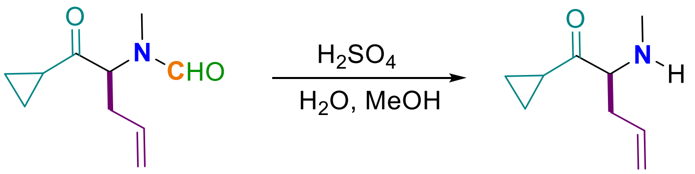

3p

13, 96%

A 50 mL round bottom flask was charged with **3p** (0.2 mmol) and methanol (1 mL), followed by slowly adding a mixture of water (0.2 mL) and concentrated sulfuric acid (98%, 0.2 mL) at room temperature. After stirring at 90 °C in an oil bath for 2 h, the reaction went to completion according to TLC analysis. The reaction mixture was firstly neutralized with aqueous NaOH (5 mol/L) and then extracted with ethyl acetate three times. The organic layers were combined and then concentrated under reduced pressure. And the residue was purified by flash column chromatography (silica gel, CH_2Cl_2: MeOH =30:1, v/v) to give the **13** as a yellow oil.

2. Supplementary Discussion

2.1 Optimization studies

2.1.1 The condition screening for the difluorocarbene-induced tertiary amine-involved Stevens rearrangement

Supplementary Table 1. The effects of halodifluorinated reagents

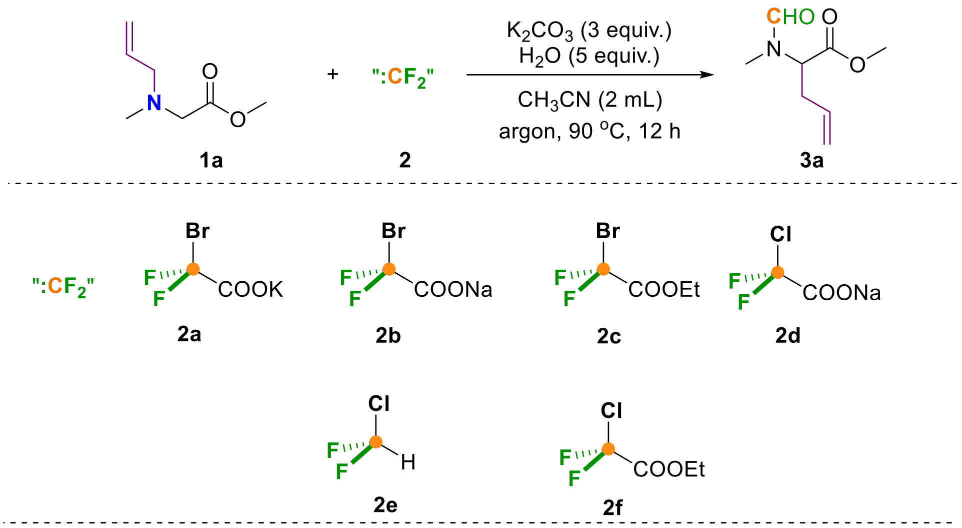

Entries	Base	Yield (%)[a]
1	**2a**	45[b]
2	**2b**	35
3	**2c**	25
4	**2d**	51
5	**2e**	trace
6	**2f**	trace

Reaction condition: [a] **1a** (1 equiv., 0.2 mmol), **2** (3 equiv., 0.6 mmol), K_2CO_3 (3 equiv.), H_2O (5 equiv., 1 mmol), CH_3CN (2 mL) at 90 °C for 12 h under argon; GC yields; [b] isolated yields; [c] ND = not detected.

Supplementary Table 2. The effects of base

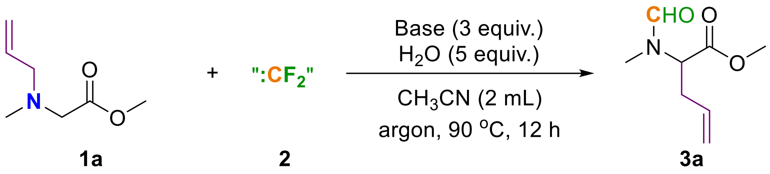

Entries	Base	Yield (%)[a]
1	HCOONa (3 equiv.)	trace
2	Rb_2CO_3 (3 equiv.)	64
3	LiOH (3 equiv.)	trace
4	Na_2CO_3 (3 equiv.)	31
5	Et_3N (3 equiv.)	ND
6	K_3PO_4 (3 equiv.)	85[b]
7	NaOH	Trace
8	KOH	Trace
9	CsF	Trace
10	Et_3N	ND
11	DBU	ND
12	KOMe	Trace
13	^{t}BuONa	ND

Reaction condition: [a] **1a** (1 equiv., 0.2 mmol), **2** (3 equiv., 0.6 mmol), Base (3 equiv.), H_2O (5 equiv., 1 mmol), CH_3CN (2 mL) at 90 °C for 12 h under argon; GC yields; [b] isolated yields; [c] ND = not detected.

Supplementary Table 3. The effects of H_2O

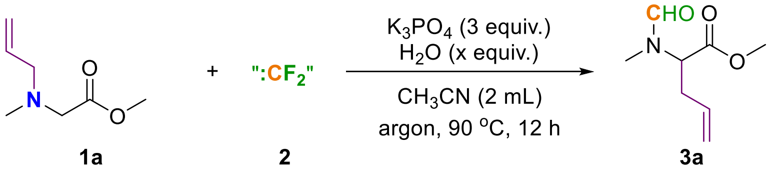

Entries	H_2O (x equiv.)	Yield (%)[a]
1	CH_3CN (dry)	trace
2	2	51
3	5	84
4	10	79

Reaction condition: [a] **1a** (1 equiv., 0.2 mmol), **2** (3 equiv., 0.6 mmol), K_3PO_4 (3 equiv.), H_2O (X equiv., X mmol), CH_3CN (2 mL) at 90 °C for 12 h under argon; GC yields; [b] isolated yields.

Supplementary Table 4. The effects of temperature, equivalent of halodifluorinated reagents and base

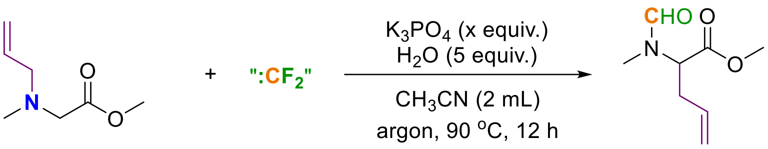

Entries	Base	T °C	[:CF_2] (equiv.)	Yield (%)[a]
1	K_3PO_4 (3eq)	80	$ClCF_2COONa$ (3)	67
2	K_3PO_4 (3eq)	90	$ClCF_2COONa$ (3)	85 [b]
3	K_3PO_4 (3eq)	100	$ClCF_2COONa$ (3)	79
4	K_3PO_4 (3eq)	90	$ClCF_2COONa$ (1)	71
5	K_3PO_4 (3eq)	90	$ClCF_2COONa$ (1.5)	86
6	K_3PO_4 (1.5 eq)	90	$ClCF_2COONa$ (1.5)	55
7	K_3PO_4 (2 eq)	90	$ClCF_2COONa$ (1.5)	71
8	K_3PO_4 (2.5 eq)	90	$ClCF_2COONa$ (1.5)	78

Reaction condition: [a] **1a** (1 equiv., 0.2 mmol), **2** (x equiv., x mmol), K_3PO_4 (x equiv.), H_2O (5 equiv., 1 mmol), CH_3CN (2 mL) at x °C for 12 h under argon; GC yields; [b] isolated yields.

2.1.2 The condition screening for the difluorocarbene-induced tertiary amine-involved Stevens rearrangement for polysubstituted amino esters

Supplementary Table 5. The effects of halodifluorinated reagents, base and H_2O

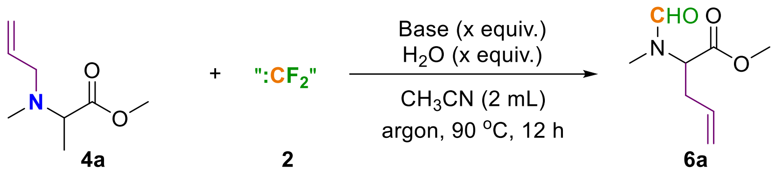

Entries	Base	[:CF2]	H_2O (x equiv.)	Yield (%)[a]
1	K_3PO_4	$BrCF_2COOK$ (**2a**)	5	7
2	K_3PO_4	$BrCF_2COONa$ (**2b**)	5	6
3	K_3PO_4	$BrCF_2COOEt$ (**2c**)	5	11
4	K_3PO_4	$ClCF_2COONa$ (**2d**)	5	15[b]
5	K_3PO_4	$ClCF_2H$ (**2e**)	5	trace
6	K_3PO_4	$ClCF_2COOEt$ (**2f**)	5	trace
7	Na_2CO_3	$ClCF_2COONa$ (**2d**)	5	11
8	K_2CO_3	$ClCF_2COONa$ (**2d**)	5	8
9	Na_3PO_4	$ClCF_2COONa$ (**2d**)	5	Trace
10	Cs_2CO_3	$ClCF_2COONa$ (**2d**)	5	Trace
11	EtOK	$ClCF_2COONa$ (**2d**)	5	ND
12	^{t}BuOK	$ClCF_2COONa$ (**2d**)	5	Trace
13	Rb_2CO_3	$ClCF_2COONa$ (**2d**)	5	22[b]
14	Rb_2CO_3	$ClCF_2COONa$ (**2d**)	15	68[b]
15	Rb_2CO_3	$ClCF_2COONa$ (**2d**)	30	66
16	Rb_2CO_3	$ClCF_2COONa$ (**2d**)	150	52

Reaction condition: [a] **4a** (1 equiv., 0.2 mmol), **2** (1.5 equiv., 0.3 mmol), Base (3 equiv.), H_2O (x equiv., x mmol), CH_3CN (2 mL) at 90 °C for 12 h under argon; GC yields; [b] isolated yields; [c] ND = not detected.

2.2 Crystal data

Crystallographic data for compound **9p** (CCDC-2305294) has been deposited with the Cambridge Crystallographic Data Centre, Copies of the data can be obtained, free of charge, on application to CCDC (Email: deposit@ccdc.cam.ac.uk).

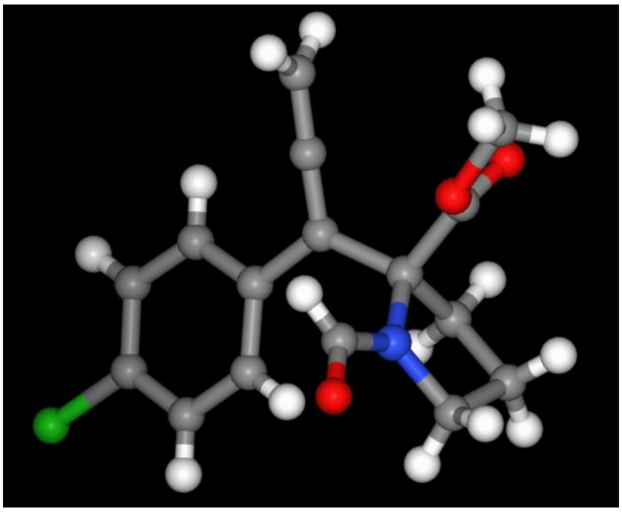

Bond precision: C-C = 0.0031 A Wavelength=0.71073

Cell:	a=7.7937(6)	b=9.7419(5)	c=11.2688(8)
	alpha=115.407(6)	beta=95.132(6)	gamma=92.018(5)
Temperature:	298 K		

	Calculated	Reported
Volume	767.16(10)	767.16(10)
Space group	P -1	P -1
Hall group	-P 1	-P 1
Moiety formula	C16 H16 Cl N O3	C16 H16 Cl N O3
Sum formula	C16 H16 Cl N O3	C16 H16 Cl N O3
Mr	305.75	305.76
Dx,g cm-3	1.324	1.324
Z	2	2
Mu (mm-1)	0.258	0.258
F000	320.0	320.5
F000'	320.44	
h,k,lmax	9,11,13	9,11,13
Nref	2717	2713
Tmin,Tmax		0.804,1.000
Tmin'		

Correction method= # Reported T Limits: Tmin=0.804 Tmax=1.000
AbsCorr = MULTI-SCAN

Data completeness= 0.999 Theta(max)= 25.000

R(reflections)= 0.0442(2070) wR2(reflections)= 0.1521(2713)

S = 0.998 Npar= 199

2.3 Characterization data for products

ethyl *N*-allyl-*N*-methylglycinate

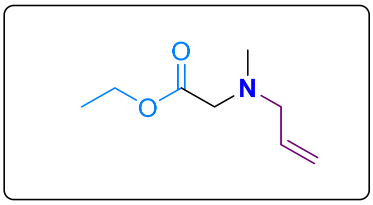

Following the **general procedure 1** on 3 mmol scale, yellow oil, yield: 61% (283 mg). R_f = 0.2 (silica gel, PE: EtOAc = 5:1, v/v), column chromatography (silica gel, PE: EtOAc: NEt_3 = 5: 1: 0.001, v/v/v).
^{1}H NMR (500 MHz, Chloroform-*d*) δ 5.8 (m, 1H), 5.2 – 5.0 (m, 2H), 4.1 (q, *J* = 7.1 Hz, 2H), 3.2 (s, 2H), 3.1 (m, 2H), 2.3 (s, 3H), 1.2 (t, *J* = 7.1 Hz, 3H).
^{13}C NMR (126 MHz, Chloroform-*d*) δ 170.9, 135.1, 118.2, 118.2, 60.4, 60.2, 57.6, 42.3, 14.2.
HRMS (ESI) m/z: $[M+H]^+$ Calcd. for $C_8H_{16}NO_2^+$ 158.1176; Found: 158.1179.

3-methylbut-3-en-1-yl *N*-allyl-*N*-methylglycinate

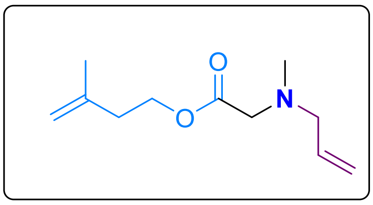

Following the **general procedure 1** on 3 mmol scale, yellow oil, yield: 63% (372 mg). R_f = 0.2 (silica gel, PE: EtOAc = 5:1, v/v), column chromatography (silica gel, PE: EtOAc: NEt_3 = 5: 1: 0.001, v/v/v).
^{1}H NMR (500 MHz, Chloroform-*d*) δ 5.9 – 5.7 (m, 1H), 5.2 – 5.0 (m, 2H), 4.8 – 4.6 (m, 2H), 4.2 (t, *J* = 6.9 Hz, 2H), 3.2 (s, 2H), 3.1 (d, *J* = 6.5 Hz, 2H), 2.3 (d, *J* = 2.2 Hz, 5H), 1.7 (s, 3H).
^{13}C NMR (126 MHz, Chloroform-*d*) δ 170.9, 141.4, 135.2, 118.1, 118.1, 112.3, 62.5, 60.2, 57.5, 42.3, 36.7, 22.4.
HRMS (ESI) m/z: $[M+H]^+$ Calcd. for $C_{11}H_{20}NO_2^+$ 198.1489; Found: 198.1489.

4-methylbenzyl *N*-allyl-*N*-methylglycinate

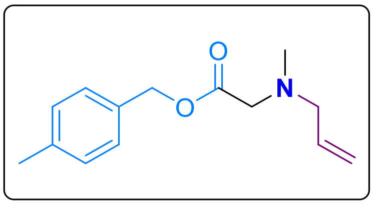

Following the **general procedure 1** on 3 mmol scale, yellow oil, yield: 59% (412 mg). R_f = 0.2 (silica gel, PE: EtOAc = 5:1, v/v), column chromatography (silica gel, PE: EtOAc: NEt_3 = 5: 1: 0.001, v/v/v).
^{1}H NMR (500 MHz, Chloroform-*d*) δ 7.2 (d, *J* = 7.8 Hz, 2H), 7.1 (d, *J* = 8.0 Hz, 2H), 5.8 (m, 1H), 5.2 – 5.1 (m, 2H), 5.1 (s, 2H), 3.2 (s, 2H), 3.1 (dt, *J* = 6.7, 1.4 Hz, 2H), 2.3 (s, 3H), 2.3 (s, 3H).
^{13}C NMR (126 MHz, Chloroform-*d*) δ 170.7, 138.0, 135.2, 132.8, 129.2, 128.5, 118.2, 66.1, 60.2, 57.5, 42.3, 21.2.
HRMS (ESI) m/z: $[M+H]^+$ Calcd. for $C_{14}H_{20}NO_2^+$ 234.1489; Found: 234.1488.

4-(*tert*-butyl)benzyl *N*-allyl-*N*-methylglycinate

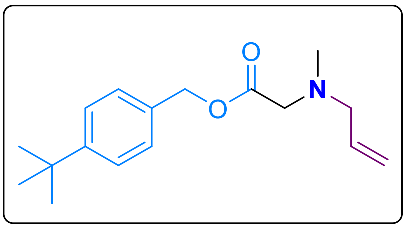

Following the **general procedure 1** on 3 mmol scale, yellow oil, yield: 64% (528 mg). R_f = 0.2 (silica gel, PE: EtOAc = 5:1, v/v), column chromatography (silica gel, PE: EtOAc: NEt_3 = 5: 1: 0.001, v/v/v).

^{1}H NMR (500 MHz, Chloroform-*d*) δ 7.4 (d, *J* = 8.3 Hz, 2H), 7.3 (d, *J* = 8.1 Hz, 2H), 5.8 (m, 1H), 5.2 – 5.1 (m, 2H), 5.1 (s, 2H), 3.3 (s, 2H), 3.1 (d, *J* = 6.6 Hz, 2H), 2.3 (s, 3H), 1.3 (s, 9H).

^{13}C NMR (126 MHz, Chloroform-*d*) δ 170.8, 151.3, 135.2, 132.8, 128.3, 125.5, 118.2, 66.1, 60.2, 57.6, 42.4, 34.6, 31.3.

HRMS (ESI) m/z: $[M+H]^+$ Calcd. for $C_{17}H_{26}NO_2^+$ 276.1958; Found: 176.1959.

[1,1'-biphenyl]-4-ylmethyl N-allyl-N-methylglycinate

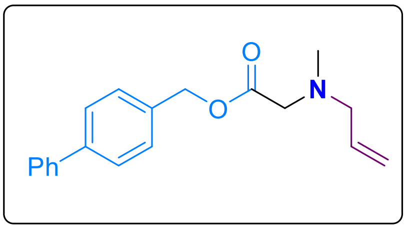

Following the **general procedure 1** on 3 mmol scale, yellow oil, yield: 70% (620 mg). R_f = 0.2 (silica gel, PE: EtOAc = 5:1, v/v), column chromatography (silica gel, PE: EtOAc: NEt_3 = 5: 1: 0.001, v/v/v).

^{1}H NMR (500 MHz, Chloroform-*d*) δ 7.6 (dd, *J* = 7.9, 3.0 Hz, 5H), 7.5 – 7.4 (m, 5H), 7.4 – 7.3 (m, 1H), 5.9 (m, 1H), 5.2 (s, 2H), 5.2 – 5.1 (m, 2H), 3.3 (s, 2H), 3.2 (d, *J* = 6.6 Hz, 2H), 2.4 (s, 3H).

^{13}C NMR (126 MHz, Chloroform-*d*) δ 170.9, 141.3, 140.6, 135.1, 134.8, 128.9, 128.8, 127.5, 127.3, 127.1, 118.3, 66.0, 60.3, 57.6, 42.5.

HRMS (ESI) m/z: $[M+H]^+$ Calcd. for $C_{19}H_{22}NO_2^+$ 296.1645; Found: 296.1463.

2-iodobenzyl *N*-allyl-*N*-methylglycinate

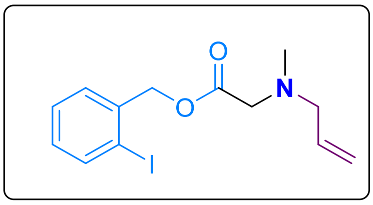

Following the **general procedure 1** on 3 mmol scale, yellow oil, yield: 68% (704 mg). R_f = 0.2 (silica gel, PE: EtOAc = 5:1, v/v), column chromatography (silica gel, PE: EtOAc: NEt_3 = 5: 1: 0.001, v/v/v).

^{1}H NMR (500 MHz, Chloroform-*d*) δ 7.8 (d, *J* = 7.9 Hz, 1H), 7.4 – 7.2 (m, 2H), 7.0 (td, *J* = 7.5, 2.0 Hz, 1H), 5.9 – 5.7 (m, 1H), 5.2 – 5.1 (m, 4H), 3.3 (s, 2H), 3.1 (d, *J* = 6.7 Hz, 2H), 2.4 (s, 3H).

^{13}C NMR (126 MHz, Chloroform-*d*) δ 170.4, 139.5, 138.2, 135.2, 129.9, 129.6, 128.3, 118.3, 118.3, 98.5, 69.9, 60.1, 57.3, 42.4.

HRMS (ESI) m/z: $[M+H]^+$ Calcd. for $C_{13}H_{17}INO_2^+$ 346.0298; Found: 34.0293.

3,5-dichlorobenzyl *N*-allyl-*N*-methylglycinate

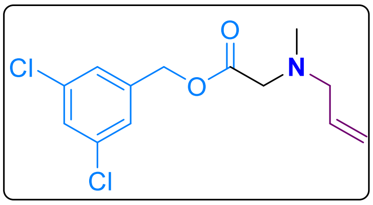

Following the **general procedure 1** on 3 mmol scale, yellow oil, yield: 71% (611 mg). R_f = 0.2 (silica gel, PE: EtOAc = 5:1, v/v), column chromatography (silica gel, PE: EtOAc: NEt_3 = 5: 1: 0.001, v/v/v).

^{1}H NMR (500 MHz, Chloroform-*d*) δ 7.2 (s, 1H), 7.2 (d, *J* = 2.0 Hz, 2H), 5.9 – 5.7 (m, 1H), 5.2 – 5.1 (m, 2H), 5.0 (s, 2H), 3.3 (s, 2H), 3.1 (d, *J* = 6.6 Hz, 2H), 2.3 (s, 3H).

^{13}C NMR (126 MHz, Chloroform-*d*) δ 170.5, 139.1, 135.1, 134.9, 128.3, 126.4, 118.4, 118.4, 64.5, 60.2, 57.3, 42.4.

HRMS (ESI) m/z: $[M+H]^+$ Calcd. for $C_{13}H_{16}ClNO_2^+$ 258.0553; Found: 258.0557.

cinnamyl *N*-allyl-*N*-methylglycinate

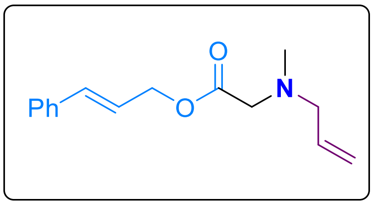

Following the **general procedure 1** on 3 mmol scale, yellow oil, yield: 65% (478 mg). R_f = 0.2 (silica gel, PE: EtOAc = 5:1, v/v), column chromatography (silica gel, PE: EtOAc: NEt_3 = 5: 1: 0.001, v/v/v).

^{1}H NMR (500 MHz, Chloroform-*d*) δ 7.3 (d, *J* = 7.4 Hz, 2H), 7.3 (dd, *J* = 8.3, 6.7 Hz, 2H), 7.2 – 7.2 (m, 1H), 6.6 (d, *J* = 15.9 Hz, 1H), 6.2 (dt, *J* = 15.9, 6.5 Hz, 1H), 5.9 – 5.8 (m, 1H), 5.2 – 5.1 (m, 2H), 4.7 (dd, *J* = 6.5, 1.4 Hz, 2H), 3.3 (s, 2H), 3.1 (dd, *J* = 6.7, 1.5 Hz, 2H), 2.3 (s, 3H).

^{13}C NMR (126 MHz, Chloroform-*d*) δ 170.7, 136.1, 135.1, 134.5, 128.6, 128.1, 126.6, 123.0, 118.3, 65.0, 60.3, 57.6, 42.4.

HRMS (ESI) m/z: $[M+H]^+$ Calcd. for $C_{15}H_{20}NO_2^+$ 246.1489; Found: 246.1494.

2-(benzyloxy)ethyl *N*-allyl-*N*-methylglycinate

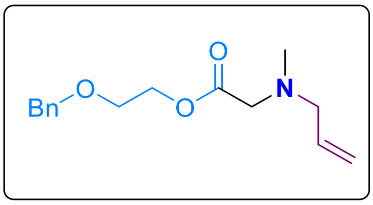

Following the **general procedure 1** on 3 mmol scale, yellow oil, yield: 69% (544 mg). R_f = 0.2 (silica gel, PE: EtOAc = 5:1, v/v), column chromatography (silica gel, PE: EtOAc: NEt_3 = 5: 1: 0.001, v/v/v).

^{1}H NMR (500 MHz, Chloroform-*d*) δ 7.3 (s, 3H), 7.2 – 7.2 (m, 2H), 5.9 – 5.7 (m, 1H), 5.2 – 5.0 (m, 2H), 4.5 (d, *J* = 1.5 Hz, 3H), 4.2 (dd, *J* = 5.8, 3.8 Hz, 2H), 3.7 (d, *J* = 4.7 Hz, 1H), 3.6 (dd, *J* = 5.5, 4.0 Hz, 2H), 3.5 (d, *J* = 4.8 Hz, 1H), 3.2 (s, 2H), 3.1 (d, *J* = 6.7 Hz, 2H), 2.3 (s, 3H).

^{13}C NMR (126 MHz, Chloroform-*d*) δ 170.8, 137.8, 135.1, 128.4, 128.4, 127.7, 127.7, 127.6, 118.3, 73.1, 73.0, 71.6, 67.8, 63.4, 61.6, 60.1, 57.3, 42.2.

HRMS (ESI) m/z: $[M+H]^+$ Calcd. for $C_{11}H_{20}NO_3^+$ 264.1594; Found: 264.1596.

2-(allyl(methyl)amino)-1-cyclopropylethan-1-one

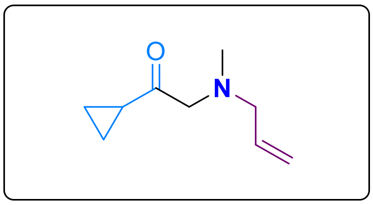

Following the **general procedure 1** on 3 mmol scale, yellow oil, yield: 73% (335 mg). R_f = 0.2 (silica gel, PE: EtOAc = 5:1, v/v), column chromatography (silica gel, PE: EtOAc: NEt_3 = 5: 1: 0.001, v/v/v).
^{1}H NMR (500 MHz, Chloroform-*d*) δ 5.9 (ddt, *J* = 16.8, 10.1, 6.5 Hz, 1H), 5.2 – 5.0 (m, 2H), 3.3 (s, 2H), 3.1 (dt, *J* = 6.6, 1.3 Hz, 2H), 2.3 (s, 3H), 2.1 (s, 1H), 1.1 – 0.9 (m, 2H), 0.9 (dd, *J* = 7.8, 3.6 Hz, 2H).
^{13}C NMR (126 MHz, Chloroform-*d*) δ 209.3, 135.1, 118.3, 118.2, 66.8, 60.9, 42.8, 18.2, 11.1, 11.1.
HRMS (ESI) m/z: $[M+H]^+$ Calcd. for $C_9H_{15}NO^+$ 154.1226; Found: 154.1224.

1-(allyl(methyl)amino)-3,3-dimethylbutan-2-one

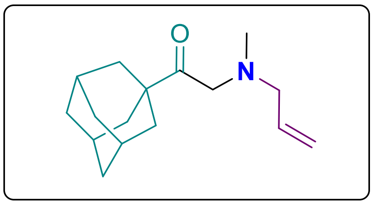

Following the **general procedure 1** on 3 mmol scale, yellow oil, yield: 73% (370 mg). R_f = 0.2 (silica gel, PE: EtOAc = 5:1, v/v), column chromatography (silica gel, PE: EtOAc: NEt_3 = 5: 1: 0.001, v/v/v).
^{1}H NMR (500 MHz, Chloroform-*d*) δ 5.9 – 5.7 (m, 1H), 5.1 – 5.0 (m, 2H), 3.3 (d, *J* = 2.2 Hz, 2H), 3.0 (d, *J* = 6.3 Hz, 2H), 2.2 (d, *J* = 3.6 Hz, 3H), 1.9 (d, *J* = 5.4 Hz, 3H), 1.7 (t, *J* = 4.7 Hz, 5H), 1.6 (d, *J* = 18.2 Hz, 7H).
^{13}C NMR (126 MHz, Chloroform-*d*) δ 212.1, 135.2, 118.1, 60.5, 59.9, 45.8, 42.4, 38.1, 36.5, 27.8.
HRMS (ESI) m/z: $[M+H]^+$ Calcd. for $C_{16}H_{26}NO^+$ 248.2009; Found: 248.2013

2-(allyl(methyl)amino)-1-(4-hydroxyphenyl)ethan-1-one

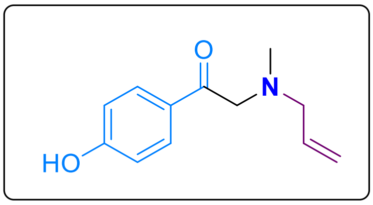

Following the **general procedure 1** on 3 mmol scale, yellow oil, yield: 52% (320 mg). R_f = 0.2 (silica gel, PE: EtOAc = 5:1, v/v), column chromatography (silica gel, PE: EtOAc: NEt_3 = 5: 1: 0.001, v/v/v).
^{1}H NMR (500 MHz, Chloroform-*d*) δ 7.8 (d, *J* = 8.7 Hz, 2H), 6.9 (d, *J* = 8.7 Hz, 2H), 6.0 – 5.9 (m, 1H), 5.8 (s, 1H), 5.3 – 5.1 (m, 2H), 3.8 (s, 2H), 3.2 (d, *J* = 6.8 Hz, 2H), 2.4 (s, 3H).
^{13}C NMR (126 MHz, Chloroform-*d*) δ 195.2, 162.2, 134.0, 130.6, 127.8, 119.5, 115.8, 61.6, 60.8, 42.6.
HRMS (ESI) m/z: $[M+H]^+$ Calcd. for $C_{12}H_{16}NO^+$ 206.1176; Found: 206.1179.

2-(allyl(methyl)amino)-1-(thiophen-2-yl)ethan-1-one

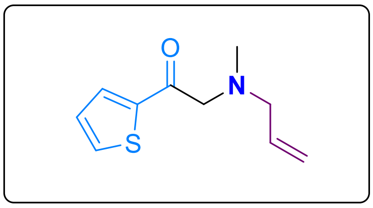

Following the **general procedure 1** on 3 mmol scale, yellow oil, yield: 72% (421 mg). R_f = 0.2 (silica gel, PE: EtOAc = 5:1, v/v), column chromatography (silica gel, PE: EtOAc: NEt_3 = 5: 1: 0.001, v/v/v).
^{1}H NMR (500 MHz, Chloroform-*d*) δ 7.9 (dt, *J* = 3.9, 1.0 Hz, 1H), 7.6 (dt, *J* = 5.0, 0.9 Hz, 1H), 7.1 (dd, *J* = 4.8, 3.9 Hz, 1H), 5.9 (ddt, *J* = 16.9, 10.1, 6.6 Hz, 1H), 5.3 – 5.0 (m, 2H), 3.6 (s, 2H), 3.1 (d, *J* = 6.5 Hz, 2H), 2.3 (s, 3H).
^{13}C NMR (126 MHz, Chloroform-*d*) δ 191.1, 141.9, 135.1, 133.8, 133.8, 132.6, 127.8, 118.5, 118.5, 63.9, 63.9, 61.0, 42.8.
HRMS (ESI) m/z: $[M+H]^+$ Calcd. for $C_{10}H_{14}NOS^+$ 196.0791; Found: 196.0786.

2-(allyl(methyl)amino)acetonitrile

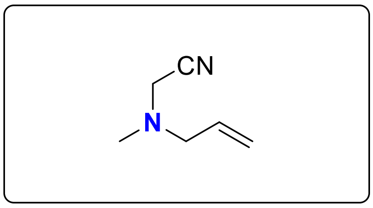

Following the **general procedure 1** on 3 mmol scale, yellow oil, yield: 62% (206 mg). R_f = 0.2 (silica gel, PE: EtOAc = 5:1, v/v), column chromatography (silica gel, PE: EtOAc: NEt_3 = 5: 1: 0.001, v/v/v).
^{1}H NMR (500 MHz, Chloroform-*d*) δ 5.8 (m, 1H), 5.3 (m, 1H), 5.2 (m, 1H), 3.5 (s, 2H), 3.1 (m, 2H), 2.4 (s, 3H).
^{13}C NMR (126 MHz, Chloroform-*d*) δ 134.0, 119.5, 114.5, 58.9, 44.2, 42.1.
HRMS (ESI) m/z: $[M+H]^+$ Calcd. for $C_6H_{11}N_2^+$ 111.0917; Found: 111.0919.

diethyl ((allyl(methyl)amino)methyl)phosphonate

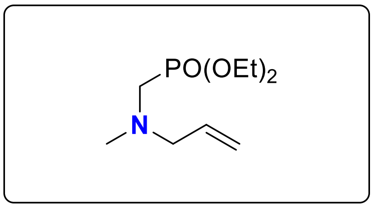

Following the **general procedure 1-2** on 3 mmol scale, yellow oil, yield: 32% (213 mg). R_f = 0.2 (silica gel, PE: EtOAc = 2:1, v/v), column chromatography (silica gel, PE: EtOAc: NEt_3 = 2: 1: 0.001, v/v/v).
^{1}H NMR (500 MHz, Chloroform-*d*) δ 5.8 (m, 1H), 5.2 – 5.1 (m, 2H), 4.1 (m, 4H), 3.1 (dm, 2H), 2.8 (d, *J* = 11.3 Hz, 2H), 2.4 (s, 3H), 1.3 (t, *J* = 7.1 Hz, 6H).
^{13}C NMR (126 MHz, Chloroform-*d*) δ 135.1, 118.3, 62.5, 62.4, 62.0, 61.9, 52.5, 51.2, 44.2, 44.2, 29.4, 16.5, 16.5.

^{31}**P NMR** (202 MHz, Chloroform-*d*) δ 25.5.
HRMS (ESI) m/z: $[M+H]^+$ Calcd. for $C_9H_{21}NO_3P^+$ 222.1254; Found: 222.1259.

methyl *N*-allyl-*N*-methylalaninate

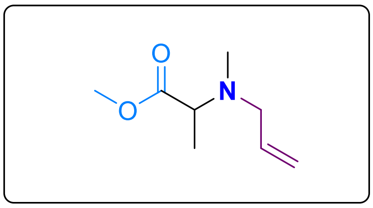

Following the **general procedure 1** on 3 mmol scale, yellow oil, yield: 74% (349 mg). R_f = 0.2 (silica gel, PE: EtOAc = 5:1, v/v), column chromatography (silica gel, PE: EtOAc: NEt_3 = 5: 1: 0.001, v/v/v).
^{1}**H NMR** (500 MHz, Chloroform-*d*) δ 5.8 (m, *J* = 15.6, 10.1, 7.1, 6.1 Hz, 1H), 5.3 – 5.0 (m, 2H), 3.7 (s, 3H), 3.4 (qd, *J* = 7.1, 1.0 Hz, 1H), 3.2 – 3.0 (m, 2H), 2.2 (d, *J* = 1.0 Hz, 3H), 1.2 (d, *J* = 7.1 Hz, 3H).
^{13}**C NMR** (126 MHz, Chloroform-*d*) δ 173.8, 135.8, 117.6, 60.5, 57.5, 51.3, 37.7, 14.3.
HRMS (ESI) m/z: $[M+H]^+$ Calcd. for $C_8H_{16}NO_2^+$ 158.1176; Found: 158.1175.

ethyl *N*-allyl-*N*-methylalaninate

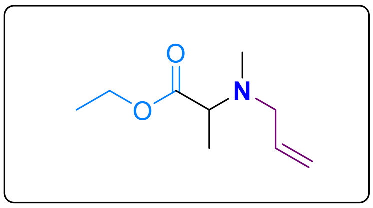

Following the **general procedure 1** on 3 mmol scale, yellow oil, yield: 72% (369 mg). R_f = 0.2 (silica gel, PE: EtOAc = 5:1, v/v), column chromatography (silica gel, PE: EtOAc: NEt_3 = 5: 1: 0.001, v/v/v).
^{1}**H NMR** (500 MHz, Chloroform-*d*) δ 5.8 (ddt, *J* = 16.8, 10.1, 6.5 Hz, 1H), 5.2 – 5.1 (m, 2H), 4.2 (qq, *J* = 7.2, 3.7 Hz, 2H), 3.4 (q, *J* = 7.1 Hz, 1H), 3.2 – 3.1 (m, 2H), 2.3 (s, 3H), 1.3 (dt, *J* = 7.2, 3.6 Hz, 6H).
^{13}**C NMR** (126 MHz, Chloroform-*d*) δ 173.2, 135.9, 117.4, 117.3, 60.5, 60.1, 57.5, 37.6, 14.6, 14.3.
HRMS (ESI) m/z: $[M+H]^+$ Calcd. for $C_9H_{18}NO_2^+$ 172.1332; Found: 172.1325.

4-iodobenzyl *N*-allyl-*N*-methylalaninate

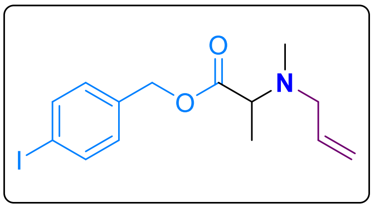

Following the **general procedure 1** on 3 mmol scale, yellow oil, yield: 68% (732 mg). R_f = 0.2 (silica gel, PE: EtOAc = 5:1, v/v), column chromatography (silica gel, PE: EtOAc: NEt_3 = 5: 1: 0.001, v/v/v).
^{1}**H NMR** (500 MHz, Chloroform-*d*) δ 7.7 (d, *J* = 8.3 Hz, 2H), 7.1 (d, *J* = 8.3 Hz, 2H), 5.8 (dd, *J* = 16.9, 10.3 Hz, 1H), 5.2 – 5.0 (m, 4H), 3.5 (q, *J* = 7.1 Hz, 1H), 3.2 – 3.0 (m, 2H), 2.3 (s, 3H), 1.3 (d, *J* = 7.1 Hz, 3H).

^{13}C NMR (126 MHz, Chloroform-*d*) δ 173.1, 137.7, 135.8, 135.7, 130.2, 117.7, 117.7, 94.0, 65.3, 60.5, 57.6, 37.7, 14.7.
HRMS (ESI) m/z: $[M+H]^+$ Calcd. for $C_{14}H_9INO_2^+$ 360.0455; Found: 360.0450.

ethyl 2-(allyl(methyl)amino)-2-phenylacetate

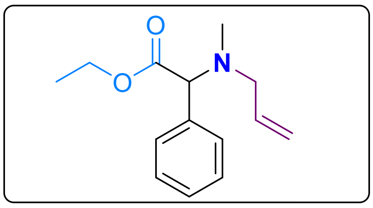

Following the **general procedure 1** on 3 mmol scale, yellow oil, yield: 62% (433 mg). R_f = 0.2 (silica gel, PE: EtOAc = 5:1, v/v), column chromatography (silica gel, PE: EtOAc: NEt_3 = 5: 1: 0.001, v/v/v).
^{1}H NMR (500 MHz, Chloroform-*d*) δ 7.4 – 7.4 (m, 2H), 7.4 – 7.3 (m, 3H), 6.0 – 5.8 (m, 1H), 5.2 – 5.1 (m, 2H), 4.2 (s, 1H), 3.7 (d, *J* = 0.7 Hz, 3H), 3.1 (ddt, *J* = 13.7, 6.6, 1.4 Hz, 1H), 3.0 – 2.9 (m, 1H), 2.2 (s, 3H).
^{13}C NMR (126 MHz, Chloroform-*d*) δ 172.4, 136.2, 135.0, 128.8, 128.5, 128.3, 118.0, 72.2, 57.8, 51.9, 51.9, 39.3.
HRMS (ESI) m/z: $[M+H]^+$ Calcd. for $C_{14}H_{20}NO_2^+$ 234.1489; Found: 234.1494.

3-(allyl(methyl)amino)dihydrofuran-2(3H)-one

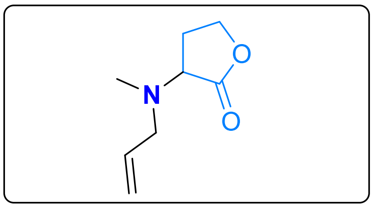

Following the **general procedure 1** on 3 mmol scale, yellow oil, yield: 66% (465 mg). R_f = 0.2 (silica gel, PE: EtOAc = 1:1, v/v), column chromatography (silica gel, PE: EtOAc: NEt_3 = 2: 1: 0.001, v/v/v).
^{1}H NMR (500 MHz, Chloroform-*d*) δ 5.8 (ddt, *J* = 16.8, 10.1, 6.5 Hz, 1H), 5.2 (dd, *J* = 17.1, 1.6 Hz, 1H), 5.1 (dd, *J* = 10.2, 1.6 Hz, 1H), 4.3 (ddd, *J* = 9.2, 6.7, 4.4 Hz, 1H), 4.2 – 4.1 (m, 1H), 3.7 (t, *J* = 9.7 Hz, 1H), 3.3 (ddt, *J* = 13.5, 6.4, 1.5 Hz, 1H), 3.2 (ddt, *J* = 13.5, 6.8, 1.3 Hz, 1H), 2.3 (s, 3H), 2.3 – 2.2 (m, 2H).
^{13}C NMR (126 MHz, Chloroform-*d*) δ 175.6, 135.4, 118.3, 118.3, 65.4, 60.9, 58.2, 37.5, 23.6.
HRMS (ESI) m/z: $[M+H]^+$ Calcd. for $C_8H_{14}NO_2^+$ 156.1019; Found: 156.1019.

methyl allylprolinate

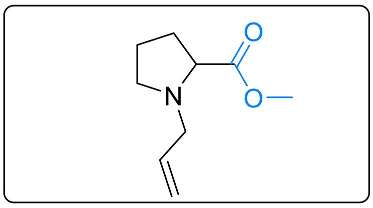

Following the **general procedure 2B** on 3 mmol scale, yellow oil, yield: 72% (365 mg). R_f = 0.2 (silica gel, PE: EtOAc = 3:1, v/v), column chromatography (silica gel, PE: EtOAc: NEt_3 = 4: 1: 0.001, v/v/v).
^{1}H NMR (500 MHz, Chloroform-*d*) δ 5.9 (ddt, *J* = 16.9, 10.1, 6.8 Hz, 1H), 5.3 – 5.0 (m, 2H), 3.7 (s, 3H), 3.3 – 3.2 (m, 1H), 3.2 – 3.0 (m, 3H), 2.3 (td, *J* = 8.9, 7.6 Hz, 1H), 2.1 – 2.0 (m, 1H), 1.9 – 1.8 (m, 2H), 1.8 – 1.7 (m, 1H).
^{13}C NMR (126 MHz, Chloroform-*d*) δ 174.7, 135.2, 117.5, 117.5, 65.3, 57.8, 53.5, 51.8, 29.5, 23.1.
HRMS (ESI) m/z: $[M+H]^+$ Calcd. for $C_9H_{16}NO_2^+$ 170.1176; Found: 170.1176.

tert-butyl allylprolinate

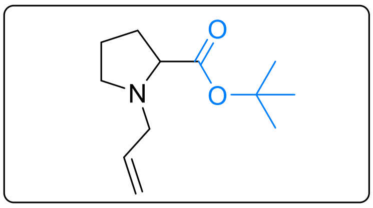

Following the **general procedure 2B** on 3 mmol scale, yellow oil, yield: 75% (475 mg). R_f = 0.2 (silica gel, PE: EtOAc = 3:1, v/v), column chromatography (silica gel, PE: EtOAc: NEt_3 = 4: 1: 0.001, v/v/v).
^{1}H NMR (500 MHz, Chloroform-*d*) δ 6.0 – 5.8 (m, 1H), 5.1 – 5.0 (m, 1H), 3.3 (ddt, *J* = 13.1, 6.3, 1.5 Hz, 1H), 3.1 – 3.0 (m, 3H), 2.4 (dt, *J* = 9.2, 8.0 Hz, 1H), 2.2 – 2.0 (m, 1H), 1.9 (ddt, *J* = 6.5, 4.4, 2.3 Hz, 2H), 1.8 – 1.7 (m, 1H), 1.4 (s, 10H).
^{13}C NMR (126 MHz, Chloroform-*d*) δ 173.3, 135.8, 117.1, 117.1, 80.5, 65.7, 57.4, 53.3, 29.3, 28.1, 22.9.
HRMS (ESI) m/z: $[M+H]^+$ Calcd. for $C_{12}H_{22}NO_2^+$ 21.1645; Found: 212.1642.

methyl (2-methylallyl)prolinate

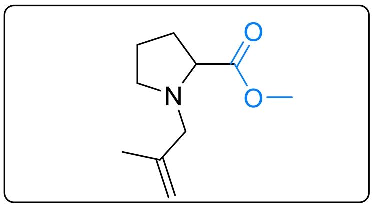

Following the **general procedure 2B** on 3 mmol scale, yellow oil, yield: 69% (379 mg). R_f = 0.2 (silica gel, PE: EtOAc = 3:1, v/v), column chromatography (silica gel, PE: EtOAc: NEt_3 = 4: 1: 0.001, v/v/v).
^{1}H NMR (500 MHz, Chloroform-*d*) δ 4.8 (dd, *J* = 2.3, 1.2 Hz, 1H), 4.8 (d, *J* = 1.8 Hz, 1H), 3.7 (s, 3H), 3.2 (d, *J* = 12.6 Hz, 1H), 3.1 (dd, *J* = 8.8, 5.7 Hz, 1H), 3.1 – 3.0 (m, 1H), 2.9 (dd, *J* = 12.6, 0.9 Hz, 1H), 2.3 (dt, *J* = 9.0, 7.7 Hz, 1H), 2.1 – 2.0 (m, 1H), 1.9 (ddd, *J* = 12.1, 5.8, 2.7 Hz, 2H), 1.7 (s, 3H).
^{13}C NMR (126 MHz, Chloroform-*d*) δ 174.8, 143.6, 112.7, 112.6, 65.6, 61.7, 53.5, 53.4, 51.6, 29.4, 23.1, 20.8.
HRMS (ESI) m/z: $[M+H]^+$ Calcd. for $C_{10}H_{18}NO_2^+$ 184.1332; Found: 184.1325.

methyl (3-methylbut-2-en-1-yl)prolinate

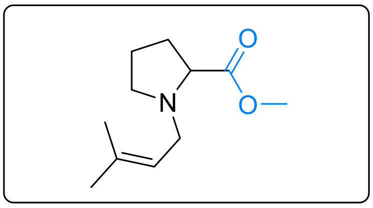

Following the **general procedure 2B** on 3 mmol scale, yellow oil, yield: 77% (455 mg). R_f = 0.2 (silica gel, PE: EtOAc = 3:1, v/v), column chromatography (silica gel, PE: EtOAc: NEt_3 = 4: 1: 0.001, v/v/v).
^{1}H NMR (500 MHz, Chloroform-*d*) δ 5.3 – 5.2 (m, 1H), 3.7 (s, 3H), 3.3 – 3.2 (m, 1H), 3.2 – 3.0 (m, 3H), 2.3 (td, *J* = 8.9, 7.7 Hz, 1H), 2.1 – 2.0 (m, 1H), 1.9 – 1.8 (m, 2H), 1.8 – 1.7 (m, 1H), 1.7 (q, *J* = 1.3 Hz, 3H), 1.6 (d, *J* = 1.4 Hz, 3H).
^{13}C NMR (126 MHz, Chloroform-*d*) δ 174.8, 135.1, 121.0, 65.4, 53.5, 51.8, 51.8, 29.5, 25.8, 23.1, 17.8.
HRMS (ESI) m/z: $[M+H]^+$ Calcd. for $C_{11}H_{20}NO_2^+$ 198.1489; Found: 198.1483.

methyl benzylprolinate

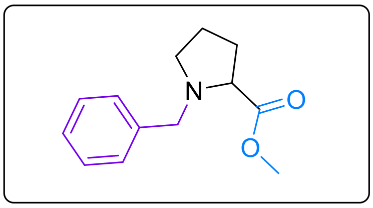

Following the **general procedure 2A** on 3 mmol scale, yellow oil, yield: 82% (539 mg). R_f = 0.2 (silica gel, PE: EtOAc = 3:1, v/v), column chromatography (silica gel, PE: EtOAc: NEt_3 = 4: 1: 0.001, v/v/v).
^{1}H NMR (500 MHz, Chloroform-*d*) δ 7.4 – 7.2 (m, 5H), 3.9 (d, *J* = 12.7 Hz, 1H), 3.6 (s, 3H), 3.6 (d, *J* = 12.8 Hz, 1H), 3.2 (dd, *J* = 9.0, 6.4 Hz, 1H), 3.0 (ddd, *J* = 8.9, 7.7, 3.0 Hz, 1H), 2.4 (td, *J* = 8.8, 7.7 Hz, 1H), 2.1 (ddd, *J* = 11.4, 6.2, 2.9 Hz, 1H), 2.0 – 1.8 (m, 2H), 1.8 – 1.7 (m, 1H).
^{13}C NMR (126 MHz, Chloroform-*d*) δ 174.6, 138.3, 129.3, 128.2, 127.1, 65.3, 58.8, 53.3, 51.7, 29.4, 23.0.
HRMS (ESI) m/z: $[M+H]^+$ Calcd. for $C_{13}H_{18}NO_2^+$ 220.1332; Found: 220.1338.

methyl ([1,1'-biphenyl]-4-ylmethyl)prolinate

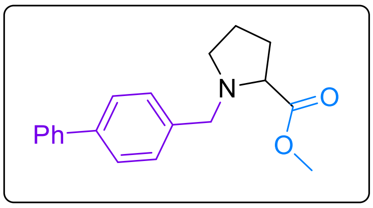

Following the **general procedure 2A** on 3 mmol scale, yellow oil, yield: 81% (717 mg). R_f = 0.2 (silica gel, PE: EtOAc = 3:1, v/v), column chromatography (silica gel, PE: EtOAc: NEt_3 = 4: 1: 0.001, v/v/v).
^{1}H NMR (500 MHz, Chloroform-*d*) δ 7.6 – 7.6 (m, 2H), 7.6 – 7.5 (m, 2H), 7.5 – 7.4 (m, 4H), 7.4 – 7.3 (m, 1H), 3.9 (d, *J* = 12.9 Hz, 1H), 3.7 (s, 3H), 3.6 (d, *J* = 12.9 Hz, 1H), 3.3 (dd, *J* = 8.9, 6.3 Hz, 1H), 3.1 (ddd, *J* = 9.0, 7.6, 3.0 Hz, 1H), 2.4 (td, *J* = 8.8, 7.7 Hz, 1H), 2.2 – 2.1 (m, 1H), 2.0 – 1.9 (m, 2H), 1.9 – 1.8 (m, 1H).

^{13}C NMR (126 MHz, Chloroform-*d*) δ 174.6, 141.0, 140.0, 137.5, 129.7, 129.7, 128.8, 127.2, 127.1, 126.9, 65.4, 58.4, 53.3, 51.7, 29.4, 23.1.
HRMS (ESI) m/z: [M+H]$^+$ Calcd. for $C_{19}H_{22}NO_2^+$ 296.1645; Found: 296.1640.

methyl (4-iodobenzyl)prolinate

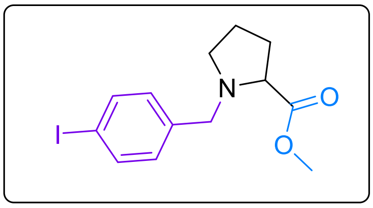

Following the **general procedure 2A** on 3 mmol scale, yellow oil, yield: 83% (859 mg). R_f = 0.2 (silica gel, PE: EtOAc = 3:1, v/v), column chromatography (silica gel, PE: EtOAc: NEt_3 = 4: 1: 0.001, v/v/v).
^{1}H NMR (500 MHz, Chloroform-*d*) δ 7.6 (d, *J* = 8.3 Hz, 2H), 7.1 (d, *J* = 8.3 Hz, 2H), 3.8 (d, *J* = 13.1 Hz, 1H), 3.6 (s, 3H), 3.5 (d, *J* = 13.0 Hz, 1H), 3.2 (dd, *J* = 8.9, 6.2 Hz, 1H), 3.0 – 2.9 (m, 1H), 2.4 – 2.3 (m, 1H), 2.2 – 2.1 (m, 1H), 2.0 – 1.8 (m, 2H), 1.8 (dt, *J* = 7.7, 4.6 Hz, 1H).
^{13}C NMR (126 MHz, Chloroform-*d*) δ 174.4, 138.3, 137.3, 131.1, 92.5, 65.2, 58.1, 58.1, 53.2, 51.7, 29.3, 23.0.
HRMS (ESI) m/z: [M+H]$^+$ Calcd. for $C_{13}H_{17}INO_2^+$ 346.0298; Found: 346.0297.

methyl (4-bromobenzyl)prolinate

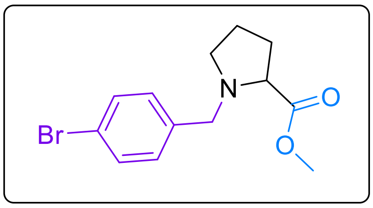

Following the **general procedure 2A** on 3 mmol scale, yellow oil, yield: 82% (731 mg). R_f = 0.2 (silica gel, PE: EtOAc = 3:1, v/v), column chromatography (silica gel, PE: EtOAc: NEt_3 = 4: 1: 0.001, v/v/v).
^{1}H NMR (500 MHz, Chloroform-*d*) δ 7.4 (d, *J* = 8.4 Hz, 2H), 7.2 (d, *J* = 8.3 Hz, 2H), 3.8 (d, *J* = 13.0 Hz, 1H), 3.5 (d, *J* = 13.0 Hz, 1H), 3.2 (dd, *J* = 8.9, 6.3 Hz, 1H), 3.0 (ddd, *J* = 8.9, 7.7, 3.1 Hz, 1H), 2.4 – 2.3 (m, 1H), 2.2 – 2.1 (m, 1H), 2.0 – 1.9 (m, 1H), 1.9 – 1.9 (m, 1H), 1.8 – 1.7 (m, 1H).
^{13}C NMR (126 MHz, Chloroform-*d*) δ 174.4, 137.6, 131.3, 130.8, 120.9, 65.3, 58.0, 53.2, 51.7, 29.3, 23.0.
HRMS (ESI) m/z: [M+H]$^+$ Calcd. for $C_{13}H_{17}BrNO_2^+$ 298.0437; Found: 298.0439.

methyl (4-bromo-3-fluorobenzyl)prolinate

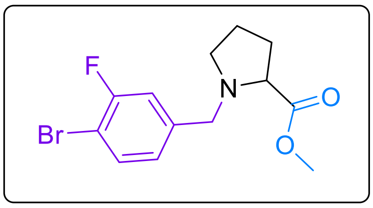

Following the **general procedure 2A** on 3 mmol scale, yellow oil, yield: 80% (756 mg). R_f = 0.2 (silica gel, PE: EtOAc = 3:1, v/v), column chromatography (silica gel, PE: EtOAc: NEt_3 = 4: 1: 0.001, v/v/v).
^{1}H NMR (500 MHz, Chloroform-*d*) δ 7.4 (dd, *J* = 8.1, 7.1 Hz, 1H), 7.1 (dd, *J* = 9.5, 2.0 Hz, 1H), 7.0 (dd, *J* = 8.2, 1.9 Hz, 1H), 3.9 (d, *J* = 13.3 Hz, 1H), 3.7 (s, 3H), 3.5 (d, *J* = 13.3 Hz, 1H), 3.3 (dd, *J* = 8.9, 6.1 Hz, 1H), 3.0 (ddd, *J* = 8.9, 7.7, 3.3 Hz, 1H), 2.4 (dt, *J* = 8.9, 7.8 Hz, 1H), 2.2 – 2.1 (m, 1H), 2.0 – 1.9 (m, 1H), 1.9 – 1.9 (m, 1H), 1.8 – 1.8 (m, 1H).
^{13}C NMR (126 MHz, Chloroform-*d*) δ 174.4, 158.0, 140.9, 140.8, 133.1, 125.6, 125.6, 117.0, 116.8, 107.3, 107.1, 65.2, 57.6, 53.2, 51.8, 29.3, 23.1.
^{19}F NMR (471 MHz, Chloroform-*d*) δ -108.0.
HRMS (ESI) m/z: $[M+H]^+$ Calcd. for $C_{13}H_{16}BrFNO_2^+$ 316.0343; Found: 316.0338.

methyl prop-2-yn-1-ylprolinate

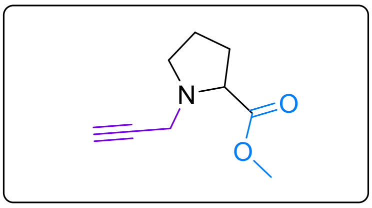

Following the **general procedure 3A** on 3 mmol scale, yellow oil, yield: 83% (416 mg). R_f = 0.2 (silica gel, PE: EtOAc = 3:1, v/v), column chromatography (silica gel, PE: EtOAc: NEt_3 = 4: 1: 0.001, v/v/v).
^{1}H NMR (500 MHz, Chloroform-*d*) δ 3.7 (s, 3H), 3.6 (dd, *J* = 3.4, 2.3 Hz, 2H), 3.4 (dd, *J* = 9.2, 6.7 Hz, 1H), 3.0 (ddd, *J* = 9.5, 7.5, 2.6 Hz, 1H), 2.7 (td, *J* = 9.1, 7.5 Hz, 1H), 2.2 (t, *J* = 2.4 Hz, 1H), 2.2 – 2.1 (m, 1H), 2.0 – 1.9 (m, 1H), 1.9 – 1.8 (m, 1H), 1.8 – 1.7 (m, 1H).
^{13}C NMR (126 MHz, Chloroform-*d*) δ 174.1, 73.2, 73.2, 62.5, 52.2, 52.0, 52.0, 41.2, 29.6, 23.3.
HRMS (ESI) m/z: $[M+H]^+$ Calcd. for $C_9H_{14}NO_2^+$ 168.1019; Found: 168.1021.

tert-butyl prop-2-yn-1-ylprolinate

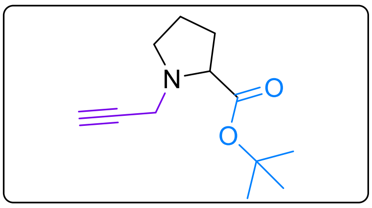

Following the **general procedure 3A** on 3 mmol scale, yellow oil, yield: 81% (508 mg). R_f = 0.2 (silica gel, PE: EtOAc = 3:1, v/v), column chromatography (silica gel, PE: EtOAc: NEt_3 = 4: 1: 0.001, v/v/v).
^{1}H NMR (500 MHz, Chloroform-*d*) δ 3.6 (dd, *J* = 8.1, 2.4 Hz, 2H), 3.3 – 3.2 (m, 1H), 3.0 (ddd, *J* = 9.9, 7.6, 2.8 Hz, 1H), 2.7 (td, *J* = 8.9, 7.6 Hz, 1H), 2.2 (t, *J* = 2.4 Hz, 1H), 2.1 – 2.0 (m, 1H), 2.0 – 1.8 (m, 2H), 1.7 (ddt, *J* = 12.1, 7.4, 2.8 Hz, 1H), 1.4 (s, 10H).
^{13}C NMR (126 MHz, Chloroform-*d*) δ 172.8, 80.7, 78.8, 72.9, 72.9, 63.0, 52.2, 41.1, 29.4, 28.1, 23.0.
HRMS (ESI) m/z: $[M+H]^+$ Calcd. for $C_{12}H_{20}NO_2^+$ 210.1489; Found: 210.1489.

methyl but-2-yn-1-ylprolinate

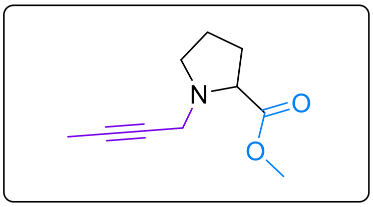

Following the **general procedure 3A** on 3 mmol scale, yellow oil, yield: 79% (429 mg). R_f = 0.2 (silica gel, PE: EtOAc = 3:1, v/v), column chromatography (silica gel, PE: EtOAc: NEt_3 = 4: 1: 0.001, v/v/v).
^{1}H NMR (500 MHz, Chloroform-*d*) δ 3.7 (s, 3H), 3.5 (d, *J* = 2.4 Hz, 2H), 3.4 (dd, *J* = 9.2, 6.7 Hz, 1H), 3.0 (ddd, *J* = 9.6, 7.5, 2.6 Hz, 1H), 2.6 (td, *J* = 9.0, 7.5 Hz, 1H), 2.2 – 2.1 (m, 1H), 2.0 – 1.8 (m, 3H), 1.8 (t, *J* = 2.4 Hz, 3H).
^{13}C NMR (126 MHz, Chloroform-*d*) δ 174.3, 80.8, 73.5, 62.8, 52.4, 51.9, 51.9, 41.7, 29.6, 23.2, 3.4.
HRMS (ESI) m/z: $[M+H]^+$ Calcd. for $C_{10}H_{16}NO_2^+$ 182.1176; Found: 182.1174.

methyl pent-2-yn-1-ylprolinate

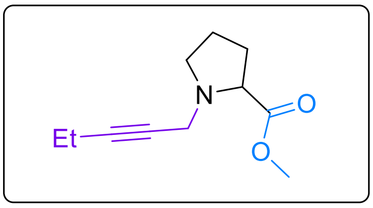

Following the **general procedure 3B** on 3 mmol scale, yellow oil, yield: 78% (456 mg). R_f = 0.2 (silica gel, PE: EtOAc = 3:1, v/v), column chromatography (silica gel, PE: EtOAc: NEt_3 = 4: 1: 0.001, v/v/v).
^{1}H NMR (500 MHz, Chloroform-*d*) δ 3.7 (s, 3H), 3.5 (d, *J* = 2.3 Hz, 2H), 3.4 (dd, *J* = 9.2, 6.7 Hz, 1H), 3.0 (ddd, *J* = 9.5, 7.4, 2.6 Hz, 1H), 2.6 (td, *J* = 9.1, 7.5 Hz, 1H), 2.1 (dddd, *J* = 9.8, 7.5, 4.9, 2.2 Hz, 2H), 2.1 – 2.1 (m, 1H), 2.0 – 1.8 (m, 2H), 1.8 (ddt, *J* = 9.3, 7.4, 3.0 Hz, 1H), 1.1 (t, *J* = 7.5 Hz, 3H).
^{13}C NMR (126 MHz, Chloroform-*d*) δ 174.3, 86.9, 73.6, 62.8, 52.3, 51.9, 51.9, 41.7, 29.7, 23.3, 14.1, 12.3.
HRMS (ESI) m/z: $[M+H]^+$ Calcd. for $C_{11}H_{18}NO_2^+$ 196.1332; Found: 196.1336.

methyl (3-cyclopropylprop-2-yn-1-yl)prolinate

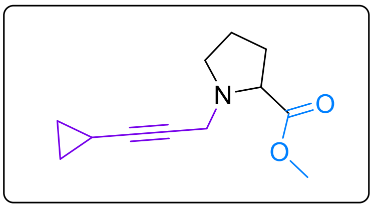

Following the **general procedure 3B** on 3 mmol scale, yellow oil, yield: 75% (466 mg). R_f = 0.2 (silica gel, PE: EtOAc = 3:1, v/v), column chromatography (silica gel, PE: EtOAc: NEt_3 = 4: 1: 0.001, v/v/v).
^{1}H NMR (500 MHz, Chloroform-*d*) δ 3.6 (s, 3H), 3.4 (d, *J* = 2.0 Hz, 2H), 3.3 (dd, *J* = 9.2, 6.7 Hz, 1H), 2.9 (ddd, *J* = 9.5, 7.4, 2.6 Hz, 1H), 2.5 (td, *J* = 9.1, 7.4 Hz, 1H), 2.1 – 2.0 (m, 1H), 1.9 – 1.7 (m, 2H), 1.7 – 1.6 (m, 1H), 1.2 – 1.1 (m, 1H), 0.7 – 0.6 (m, 2H), 0.5 (ddd, *J* = 7.0, 4.9, 3.8 Hz, 2H).
^{13}C NMR (126 MHz, Chloroform-*d*) δ 174.2, 88.6, 69.6, 62.7, 52.3, 51.8, 51.8, 41.7, 29.6, 23.2, 8.1.

HRMS (ESI) m/z: $[M+H]^+$ Calcd. for $C_{12}H_{18}NO_2^+$ 208.1332; Found: 208.1329.

methyl hept-2-yn-1-ylprolinate

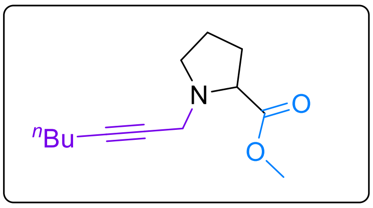

Following the **general procedure 3B** on 3 mmol scale, yellow oil, yield: 80% (535 mg). R_f = 0.2 (silica gel, PE: EtOAc = 3:1, v/v), column chromatography (silica gel, PE: EtOAc: NEt_3 = 4: 1: 0.001, v/v/v).
^{1}H NMR (500 MHz, Chloroform-*d*) δ 3.7 (s, 3H), 3.5 (t, *J* = 2.2 Hz, 2H), 3.4 (dd, *J* = 9.2, 6.7 Hz, 1H), 3.0 (ddd, *J* = 9.5, 7.4, 2.6 Hz, 1H), 2.7 (td, *J* = 9.1, 7.5 Hz, 1H), 2.2 (qt, *J* = 7.2, 2.8 Hz, 2H), 2.1 – 2.1 (m, 1H), 2.0 – 1.8 (m, 2H), 1.8 (dddd, *J* = 12.2, 7.5, 3.9, 1.7 Hz, 1H), 1.5 – 1.4 (m, 2H), 1.4 – 1.3 (m, 2H), 0.9 (t, *J* = 7.2 Hz, 3H).
^{13}C NMR (126 MHz, Chloroform-*d*) δ 174.3, 85.6, 74.1, 62.7, 52.3, 51.9, 41.7, 30.9, 29.7, 23.3, 21.9, 18.3, 13.6.
HRMS (ESI) m/z: $[M+H]^+$ Calcd. for $C_{13}H_{22}NO_2^+$ 224.1645; Found: 224.1650.

methyl (4,4-dimethylpent-2-yn-1-yl)prolinate

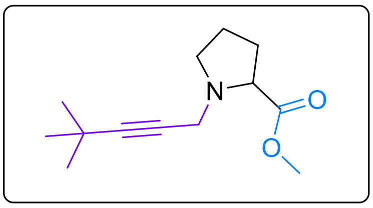

Following the **general procedure 3B** on 3 mmol scale, yellow oil, yield: 79% (529 mg). R_f = 0.2 (silica gel, PE: EtOAc = 3:1, v/v), column chromatography (silica gel, PE: EtOAc: NEt_3 = 4: 1: 0.001, v/v/v).
^{1}H NMR (500 MHz, Chloroform-*d*) δ 3.7 (s, 3H), 3.5 (d, *J* = 1.1 Hz, 2H), 3.4 (dd, *J* = 9.3, 6.5 Hz, 1H), 3.0 (ddd, *J* = 9.5, 7.3, 2.6 Hz, 1H), 2.6 (td, *J* = 9.0, 7.3 Hz, 1H), 2.2 – 2.0 (m, 1H), 2.0 – 1.9 (m, 1H), 1.9 – 1.8 (m, 1H), 1.8 – 1.7 (m, 1H), 1.2 (s, 9H).
^{13}C NMR (126 MHz, Chloroform-*d*) δ 174.4, 94.1, 72.6, 62.7, 52.3, 52.0, 52.0, 41.8, 31.2, 29.8, 27.4, 23.5.
HRMS (ESI) m/z: $[M+H]^+$ Calcd. for $C_{13}H_{22}NO_2^+$ 224.1645; Found: 224.1644.

methyl (4-((tetrahydro-2H-pyran-2-yl)oxy)but-2-yn-1-yl)prolinate

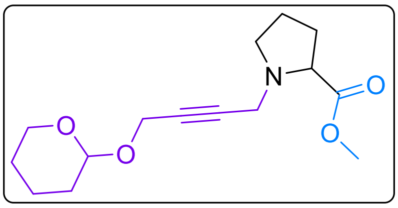

Following the **general procedure 3B** on 3 mmol scale, yellow oil, yield: 77% (649 mg). R_f = 0.2 (silica gel, PE: EtOAc = 3:1, v/v), column chromatography (silica gel, PE: EtOAc: NEt_3 = 4: 1: 0.001, v/v/v).

^{1}H NMR (500 MHz, Chloroform-*d*) δ 4.6 (dd, *J* = 4.2, 2.9 Hz, 1H), 3.8 (ddd, *J* = 11.2, 8.5, 3.1 Hz, 1H), 3.7 (dt, *J* = 9.6, 7.1 Hz, 1H), 3.7 (s, 3H), 3.5 – 3.4 (m, 4H), 3.4 (dd, *J* = 9.2, 6.7 Hz, 1H), 3.0 (ddd, *J* = 9.6, 7.5, 2.6 Hz, 1H), 2.6 (td, *J* = 9.1, 7.5 Hz, 1H), 2.5 – 2.4 (m, 2H), 2.1 – 2.0 (m, 1H), 2.0 – 1.9 (m, 1H), 1.9 – 1.8 (m, 1H), 1.8 – 1.7 (m, 2H), 1.7 – 1.6 (m, 1H), 1.6 – 1.4 (m, 4H).
^{13}C NMR (126 MHz, Chloroform-*d*) δ 174.2, 98.7, 82.3, 75.3, 65.9, 62.6, 62.1, 52.2, 51.9, 51.9, 41.6, 30.5, 29.6, 25.4, 23.3, 20.1, 19.4.
HRMS (ESI) m/z: $[M+H]^+$ Calcd. for $C_{15}H_{24}NO_2^+$ 282.1700; Found: 282.1695.

methyl (4-(2-hydroxypropoxy)but-2-yn-1-yl)prolinate

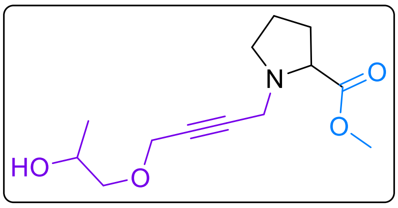

Following the **general procedure 3B** on 3 mmol scale, yellow oil, yield: 68% (520 mg). R_f = 0.2 (silica gel, PE: EtOAc = 3:1, v/v), column chromatography (silica gel, PE: EtOAc: NEt_3 = 4: 1: 0.001, v/v/v).
^{1}H NMR (500 MHz, Chloroform-*d*) δ 4.2 (d, *J* = 1.9 Hz, 2H), 3.9 (td, *J* = 3.3, 1.6 Hz, 1H), 3.7 (q, *J* = 1.4 Hz, 3H), 3.6 (d, *J* = 1.8 Hz, 2H), 3.5 (ddt, *J* = 10.6, 3.5, 1.4 Hz, 1H), 3.4 (ddd, *J* = 8.7, 6.4, 1.8 Hz, 1H), 3.3 (ddd, *J* = 9.4, 8.0, 1.5 Hz, 1H), 3.1 – 3.0 (m, 1H), 2.7 – 2.6 (m, 1H), 2.5 (s, 1H), 2.1 (dddd, *J* = 10.5, 7.1, 3.4, 1.6 Hz, 1H), 1.9 (ddt, *J* = 9.4, 7.9, 2.9 Hz, 1H), 1.9 (ddd, *J* = 11.5, 6.2, 2.4 Hz, 1H), 1.8 – 1.7 (m, 1H), 1.1 (dd, *J* = 6.4, 1.8 Hz, 3H).
^{13}C NMR (126 MHz, Chloroform-*d*) δ 174.1, 81.3, 80.9, 75.4, 66.3, 62.8, 58.7, 52.4, 52.0, 41.6, 29.6, 23.2, 18.7.
HRMS (ESI) m/z: $[M+H]^+$ Calcd. for $C_{13}H_{22}NO_4^+$ 256.1543; Found: 256.1546.

methyl (4-methylpent-4-en-2-yn-1-yl)prolinate

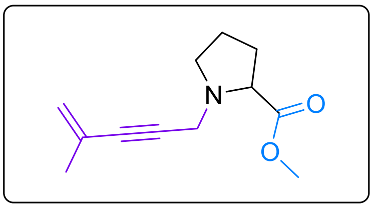

Following the **general procedure 3B** on 3 mmol scale, yellow oil, yield: 77% (478 mg). R_f = 0.2 (silica gel, PE: EtOAc = 3:1, v/v), column chromatography (silica gel, PE: EtOAc: NEt_3 = 4: 1: 0.001, v/v/v).
^{1}H NMR (500 MHz, Chloroform-*d*) δ 5.2 (dd, *J* = 2.1, 1.1 Hz, 1H), 5.1 (t, *J* = 1.8 Hz, 1H), 3.7 (s, 3H), 3.6 (s, 2H), 3.4 (dd, *J* = 9.2, 6.6 Hz, 1H), 3.0 (ddd, *J* = 9.4, 7.4, 2.6 Hz, 1H), 2.6 (td, *J* = 9.1, 7.5 Hz, 1H), 2.1 – 2.0 (m, 1H), 2.0 – 1.8 (m, 2H), 1.8 (t, *J* = 1.3 Hz, 3H), 1.8 – 1.7 (m, 1H).
^{13}C NMR (126 MHz, Chloroform-*d*) δ 174.2, 126.5, 121.6, 121.5, 86.6, 83.1, 62.8, 52.4, 51.9, 51.9, 42.0, 29.6, 23.6, 23.3.
HRMS (ESI) m/z: $[M+H]^+$ Calcd. for $C_{12}H_{18}NO_2^+$ 208.1332; Found: 208.1330.

methyl (3-(cyclohex-1-en-1-yl)prop-2-yn-1-yl)prolinate

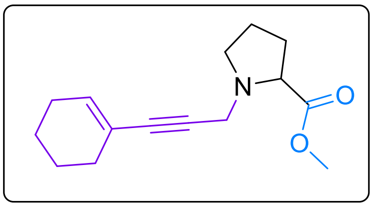

Following the **general procedure 3B** on 3 mmol scale, yellow oil, yield: 79% (585 mg). R_f = 0.2 (silica gel, PE: EtOAc = 3:1, v/v), column chromatography (silica gel, PE: EtOAc: NEt_3 = 4: 1: 0.001, v/v/v).
^{1}H NMR (500 MHz, Chloroform-*d*) δ 6.1 – 6.0 (m, 1H), 3.7 (s, 3H), 3.7 (d, *J* = 2.1 Hz, 2H), 3.4 (dd, *J* = 9.2, 6.6 Hz, 1H), 3.1 (ddd, *J* = 9.4, 7.3, 2.5 Hz, 1H), 2.7 (td, *J* = 9.0, 7.4 Hz, 1H), 2.2 – 2.1 (m, 1H), 2.1 – 2.0 (m, 4H), 2.0 – 1.9 (m, 1H), 1.9 – 1.9 (m, 1H), 1.9 – 1.7 (m, 1H), 1.6 (ddq, *J* = 6.3, 4.8, 2.3 Hz, 2H), 1.6 (dtd, *J* = 6.3, 4.8, 4.0, 2.0 Hz, 2H).
^{13}C NMR (126 MHz, Chloroform-*d*) δ 174.3, 134.5, 120.4, 87.2, 81.0, 62.7, 52.4, 52.0, 42.1, 29.7, 29.4, 25.5, 23.4, 22.3, 21.5.
HRMS (ESI) m/z: $[M+H]^+$ Calcd. for $C_{15}H_{22}NO_2^+$ 248.1645; Found: 248.1647.

methyl (3-phenylprop-2-yn-1-yl)prolinate

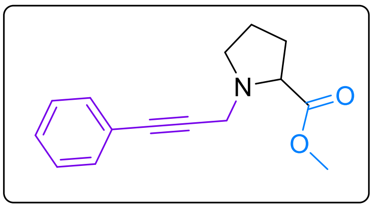

Following the **general procedure 3B** on 3 mmol scale, yellow oil, yield: 80% (583 mg). R_f = 0.2 (silica gel, PE: EtOAc = 3:1, v/v), column chromatography (silica gel, PE: EtOAc: NEt_3 = 4: 1: 0.001, v/v/v).
^{1}H NMR (500 MHz, Chloroform-*d*) δ 7.4 – 7.4 (m, 2H), 7.3 – 7.3 (m, 3H), 3.8 (d, *J* = 2.6 Hz, 2H), 3.7 (s, 3H), 3.5 (dd, *J* = 9.2, 6.7 Hz, 1H), 3.2 – 3.1 (m, 1H), 2.8 (td, *J* = 9.0, 7.4 Hz, 1H), 2.2 (dtd, *J* = 12.1, 9.0, 7.1 Hz, 1H), 2.0 – 1.9 (m, 2H), 1.8 (dddd, *J* = 9.2, 7.3, 4.5, 2.8 Hz, 1H).
^{13}C NMR (126 MHz, Chloroform-*d*) δ 174.3, 131.7, 128.3, 128.1, 123.0, 85.4, 84.1, 63.0, 52.6, 52.1, 42.3, 29.7, 23.4.
HRMS (ESI) m/z: $[M+H]^+$ Calcd. for $C_{15}H_{18}NO_2^+$ 244.1332; Found: 244.1329.

methyl (3-(4-ethylphenyl)prop-2-yn-1-yl)prolinate

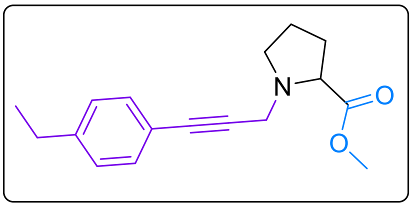

Following the **general procedure 3B** on 3 mmol scale, yellow oil, yield: 79% (642 mg). R_f = 0.2 (silica gel, PE: EtOAc = 3:1, v/v), column chromatography (silica gel, PE: EtOAc: NEt_3 = 4: 1: 0.001, v/v/v).
^{1}H NMR (500 MHz, Chloroform-*d*) δ 7.3 (d, *J* = 8.2 Hz, 2H), 7.1 (d, *J* = 7.9 Hz, 2H), 3.8 (d, *J* = 3.9 Hz, 2H), 3.7 (s, 3H), 3.5 (dd, *J* = 9.2, 6.6 Hz, 1H), 3.1 (ddd, *J* = 9.4, 7.4, 2.6 Hz, 1H), 2.7 (td, *J* = 9.0, 7.4 Hz,

1H), 2.6 (q, J = 7.6 Hz, 2H), 2.1 (ddd, J = 12.5, 6.1, 2.6 Hz, 1H), 2.1 – 1.9 (m, 2H), 1.8 – 1.7 (m, 1H), 1.2 (t, J = 7.6 Hz, 3H).

^{13}C NMR (126 MHz, Chloroform-*d*) δ 174.2, 144.5, 131.7, 127.8, 120.2, 85.5, 83.3, 62.9, 52.5, 52.0, 42.3, 29.7, 28.8, 23.4, 15.4, 15.4.

HRMS (ESI) m/z: $[M+H]^+$ Calcd. for $C_{17}H_{22}NO_2^+$ 272.1645; Found: 272.1645.

methyl (3-(4-methoxyphenyl)prop-2-yn-1-yl)prolinate

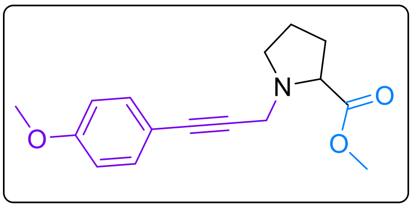

Following the **general procedure 3B** on 3 mmol scale, yellow oil, yield: 75% (614 mg). R_f = 0.2 (silica gel, PE: EtOAc = 3:1, v/v), column chromatography (silica gel, PE: EtOAc: NEt_3 = 4: 1: 0.001, v/v/v).

^{1}H NMR (500 MHz, Chloroform-*d*) δ 7.3 (d, J = 8.8 Hz, 2H), 6.8 (d, J = 8.8 Hz, 2H), 3.8 (s, 3H), 3.8 (d, J = 2.7 Hz, 2H), 3.7 (s, 3H), 3.5 (dd, J = 9.2, 6.7 Hz, 1H), 3.1 (dd, J = 5.5, 3.4 Hz, 1H), 2.8 – 2.7 (m, 1H), 2.2 – 2.1 (m, 1H), 2.0 – 1.9 (m, 2H), 1.8 (tdd, J = 9.0, 4.1, 2.4 Hz, 1H).

^{13}C NMR (126 MHz, Chloroform-*d*) δ 174.3, 159.5, 133.2, 115.1, 113.9, 85.2, 82.6, 63.0, 55.3, 52.6, 52.0, 42.3, 29.7, 23.4.

HRMS (ESI) m/z: $[M+H]^+$ Calcd. for $C_{16}H_{20}NO_2^+$ 274.1438; Found: 274.1436.

methyl (3-(4-chlorophenyl)prop-2-yn-1-yl)prolinate

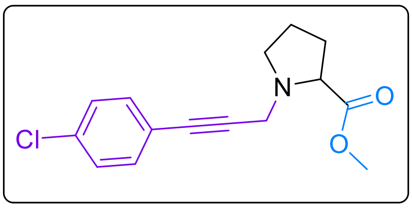

Following the **general procedure 3B** on 3 mmol scale, yellow oil, yield: 77% (640 mg). R_f = 0.2 (silica gel, PE: EtOAc = 3:1, v/v), column chromatography (silica gel, PE: EtOAc: NEt_3 = 4: 1: 0.001, v/v/v).

^{1}H NMR (500 MHz, Chloroform-*d*) δ 7.3 (d, J = 8.5 Hz, 2H), 7.2 (d, J = 8.5 Hz, 2H), 3.7 (d, J = 1.7 Hz, 2H), 3.7 (s, 3H), 3.4 (dd, J = 9.2, 6.7 Hz, 1H), 3.2 – 3.0 (m, 1H), 2.7 (td, J = 9.1, 7.6 Hz, 1H), 2.2 – 2.1 (m, 1H), 2.0 – 1.8 (m, 3H).

^{13}C NMR (126 MHz, Chloroform-*d*) δ 174.1, 134.1, 132.9, 128.6, 121.5, 85.3, 84.2, 63.0, 52.6, 52.0, 52.0, 42.2, 29.7, 23.3.

HRMS (ESI) m/z: $[M+H]^+$ Calcd. for $C_{15}H_{17}ClNO_2^+$ 278.0942; Found: 278.0939.

methyl (3-(4-bromophenyl)prop-2-yn-1-yl)prolinate

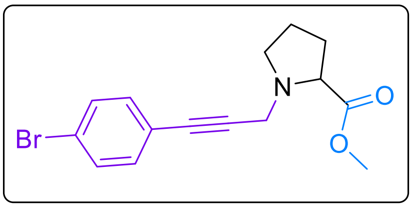

Following the **general procedure 3B** on 3 mmol scale, yellow oil, yield: 80% (770 mg). R_f = 0.2 (silica gel, PE: EtOAc = 3:1, v/v), column chromatography (silica gel, PE: EtOAc: NEt_3 = 4: 1: 0.001, v/v/v).

^{1}H NMR (500 MHz, Chloroform-*d*) δ 7.3 (d, *J* = 8.5 Hz, 2H), 7.2 (d, *J* = 8.5 Hz, 2H), 3.8 (d, *J* = 1.6 Hz, 2H), 3.7 (s, 3H), 3.4 (dd, *J* = 9.2, 6.6 Hz, 1H), 3.1 (ddd, *J* = 9.5, 7.4, 2.6 Hz, 1H), 2.7 (td, *J* = 9.0, 7.5 Hz, 1H), 2.2 – 2.1 (m, 1H), 2.0 – 1.9 (m, 2H), 1.8 (dtd, *J* = 9.1, 4.7, 2.0 Hz, 1H).

^{13}C NMR (126 MHz, Chloroform-*d*) δ 174.1, 134.1, 132.9, 128.6, 121.5, 85.3, 84.2, 63.0, 52.6, 52.0, 52.0, 42.3, 29.7, 23.3.

HRMS (ESI) m/z: $[M+H]^+$ Calcd. for $C_{15}H_{17}BrNO_2^+$ 322.0437; Found: 322.0441.

methyl (3-(4-formylphenyl)prop-2-yn-1-yl)prolinate

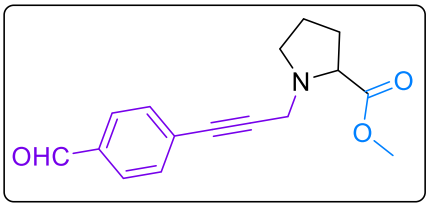

Following the **general procedure 3B** on 3 mmol scale, yellow oil, yield: 69% (561 mg). R_f = 0.2 (silica gel, PE: EtOAc = 3:1, v/v), column chromatography (silica gel, PE: EtOAc: NEt_3 = 4: 1: 0.001, v/v/v).

^{1}H NMR (500 MHz, Chloroform-*d*) δ 9.9 (s, 1H), 7.8 (d, *J* = 8.0 Hz, 2H), 7.5 (d, *J* = 8.0 Hz, 2H), 3.8 (s, 2H), 3.7 (s, 3H), 3.4 (dd, *J* = 9.2, 6.6 Hz, 1H), 3.1 (ddd, *J* = 9.5, 7.4, 2.6 Hz, 1H), 2.7 (td, *J* = 8.9, 7.5 Hz, 1H), 2.2 – 2.1 (m, 1H), 2.0 – 1.9 (m, 1H), 1.9 – 1.9 (m, 1H), 1.8 – 1.8 (m, 1H).

^{13}C NMR (126 MHz, Chloroform-*d*) δ 191.4, 174.0, 135.4, 132.2, 129.5, 129.3, 88.8, 84.6, 63.0, 52.7, 52.0, 52.0, 42.3, 29.6, 23.3.

HRMS (ESI) m/z: $[M+H]^+$ Calcd. for $C_{16}H_{18}NO_3^+$ 272.1281; Found: 272.1277.

methyl -2-(*N*-methylformamido)pent-4-enoate（3a)

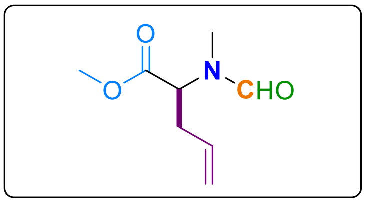

Following the **general procedure 4** on 0.2 mmol scale, yellow oil, yield: 85% (29 mg). R_f = 0.2 (silica gel, PE: EtOAc = 5:1, v/v), column chromatography (silica gel, PE: EtOAc= 5: 1, v/v).

^{1}H NMR (500 MHz, Chloroform-*d*) δ 8.1 (d, *J* = 16.4 Hz, 1H), 5.8 – 5.6 (m, 1H), 5.2 – 5.1 (m, 2H), 5.1 – 5.1 (m, 1H), 3.8 (d, *J* = 13.1 Hz, 3H), 2.9 (d, *J* = 49.0 Hz, 3H), 2.8 – 2.5 (m, 2H).

^{13}C NMR (126 MHz, Chloroform-*d*) δ 170.6, 170.4, 163.4, 163.3, 133.1, 132.2, 119.4, 118.2, 60.5, 53.4, 52.6, 52.4, 33.0, 32.3, 31.3, 27.0.

HRMS (ESI) m/z: $[M+H]^+$ Calcd. for $C_8H_{14}NO_3^+$ 172.0968; Found: 172.0970.

ethyl 2-(*N*-methylformamido)pent-4-enoate (3b)

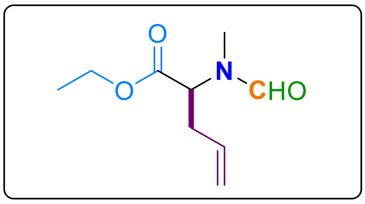

Following the **general procedure 4** on 0.2 mmol scale, yellow oil, yield: 79% (29 mg). R_f = 0.2 (silica gel, PE: EtOAc = 5:1, v/v), column chromatography (silica gel, PE: EtOAc= 5: 1, v/v).

^{1}H NMR (500 MHz, Chloroform-*d*) δ 8.0 (d, *J* = 15.6 Hz, 1H), 5.7 – 5.6 (m, 1H), 5.2 – 5.1 (m, 1H), 5.1 – 5.0 (m, 1H), 4.2 – 4.1 (m, 2H), 4.1 (m, *J* = 10.3, 5.2 Hz, 1H), 2.8 (dd, *J* = 49.9, 1.3 Hz, 3H), 2.7 (dddd, *J* = 14.8, 6.5, 4.5, 2.9 Hz, 1H), 2.5 – 2.4 (m, 1H), 1.3 – 1.2 (m, 3H).

^{13}C NMR (126 MHz, Chloroform-*d*) δ 170.1, 169.9, 163.4, 163.3, 133.2, 132.3, 119.2, 118.0, 61.7, 61.4, 60.6, 53.5, 33.0, 32.4, 31.3, 27.0, 14.1, 14.1.

HRMS (ESI) m/z: $[M+H]^+$ Calcd. for $C_9H_{16}NO_3^+$ 186.1125; Found: 186.1126.

tert-butyl 2-(*N*-methylformamido)pent-4-enoate (3c)

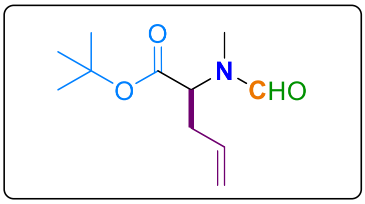

Following the **general procedure 4** on 0.2 mmol scale, yellow oil, yield: 60% (26 mg). R_f = 0.2 (silica gel, PE: EtOAc = 5:1, v/v), column chromatography (silica gel, PE: EtOAc= 5: 1, v/v).

^{1}H NMR (500 MHz, Chloroform-*d*) δ 8.1 (d, *J* = 17.1 Hz, 1H), 5.7 – 5.5 (m, 1H), 5.2 – 5.0 (m, 2H), 4.0 (dd, *J* = 10.1, 5.4 Hz, 1H), 2.8 (d, *J* = 48.4 Hz, 3H), 2.7 – 2.6 (m, 1H), 2.5 – 2.4 (m, 1H), 1.4 (d, *J* = 3.4 Hz, 9H).

^{13}C NMR (126 MHz, Chloroform-*d*) δ 169.2, 169.0, 163.4, 163.4, 133.5, 132.6, 119.0, 117.8, 82.6, 82.0, 61.3, 54.1, 33.0, 32.5, 31.2, 27.9, 27.9, 26.9.

HRMS (ESI) m/z: $[M+H]^+$ Calcd. for $C_{11}H_{20}NO_3^+$ 214.1438; Found: 214.1436.

cyclopropylmethyl 2-(*N*-methylformamido)pent-4-enoate (3d)

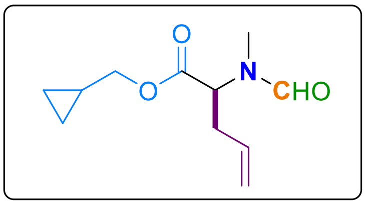

Following the **general procedure 4** on 0.2 mmol scale, yellow oil, yield: 76% (32 mg). R_f = 0.2 (silica gel, PE: EtOAc = 5:1, v/v), column chromatography (silica gel, PE: EtOAc= 5: 1, v/v).

^{1}H NMR (500 MHz, Chloroform-*d*) δ 8.1 (d, *J* = 13.3 Hz, 1H), 5.7 – 5.6 (m, 1H), 5.2 – 5.1 (m, 2H), 4.1 (dd, *J* = 10.4, 5.3 Hz, 1H), 3.9 (dt, *J* = 10.0, 7.6 Hz, 2H), 2.9 (d, *J* = 47.8 Hz, 3H), 2.8 – 2.7 (m, 1H), 2.6 – 2.4 (m, 1H), 1.1 (ddt, *J* = 7.6, 4.9, 2.4 Hz, 1H), 0.6 – 0.5 (m, 2H), 0.3 (td, *J* = 4.7, 2.5 Hz, 2H).

^{13}C NMR (126 MHz, Chloroform-*d*) δ 170.2, 170.0, 163.5, 163.4, 133.3, 132.3, 119.3, 118.1, 70.5, 70.2, 60.7, 53.5, 33.1, 32.5, 31.3, 27.0, 9.7, 3.4, 3.3, 3.3.

HRMS (ESI) m/z: $[M+H]^+$ Calcd. for $C_{11}H_{20}NO_3^+$ 212.1281; Found: 212.1278.

isopentyl 2-(*N*-methylformamido)pent-4-enoate (3e)

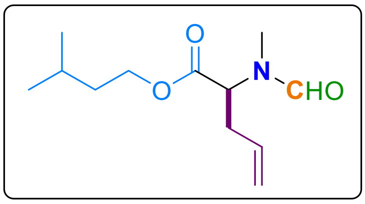

Following the **general procedure 4** on 0.2 mmol scale, yellow oil, yield: 68% (30 mg). R_f = 0.2 (silica gel, PE: EtOAc = 5:1, v/v), column chromatography (silica gel, PE: EtOAc= 5: 1, v/v).

^{1}H NMR (500 MHz, Chloroform-*d*) δ 8.1 (d, *J* = 16.3 Hz, 1H), 5.7 – 5.6 (m, 1H), 5.2 – 5.1 (m, 2H), 4.2 – 4.1 (m, 2H), 4.0 – 3.8 (m, 0H), 2.9 (d, *J* = 48.2 Hz, 3H), 2.8 – 2.7 (m, 1H), 2.6 – 2.4 (m, 1H), 1.6 (dtp, *J* = 13.2, 6.5, 3.0 Hz, 1H), 1.5 (qd, *J* = 6.9, 2.7 Hz, 2H), 0.9 (dd, *J* = 6.7, 1.9 Hz, 6H).

^{13}C NMR (126 MHz, Chloroform-*d*) δ 170.2, 170.0, 163.5, 163.3, 133.3, 132.3, 119.3, 118.1, 64.5, 64.1, 60.7, 53.5, 37.2, 37.1, 33.0, 32.4, 31.3, 27.1, 25.0, 25.0, 22.4, 22.4.

HRMS (ESI) m/z: $[M+H]^+$ Calcd. for $C_{12}H_{22}NO_3^+$ 228.1594; Found: 228.1592.

3-methylbut-3-en-1-yl -2-(*N*-methylformamido)pent-4-enoate (3f)

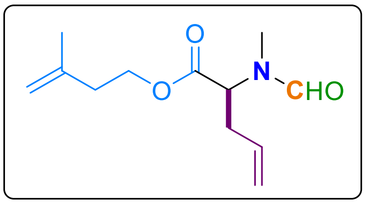

Following the **general procedure 4** on 0.2 mmol scale, yellow oil, yield: 84% (38 mg). R_f = 0.2 (silica gel, PE: EtOAc = 5:1, v/v), column chromatography (silica gel, PE: EtOAc= 5: 1, v/v).

^{1}H NMR (500 MHz, Chloroform-*d*) δ 8.1 (d, *J* = 16.9 Hz, 1H), 5.7 – 5.5 (m, 1H), 5.2 – 5.1 (m, 2H), 4.8 – 4.7 (m, 2H), 4.3 – 4.2 (m, 2H), 4.1 (dd, *J* = 10.4, 5.3 Hz, 1H), 2.8 (d, *J* = 46.5 Hz, 3H), 2.8 – 2.7 (m, 1H), 2.5 (dddd, *J* = 18.8, 15.1, 10.6, 7.8 Hz, 1H), 2.3 (dt, *J* = 7.1, 3.5 Hz, 2H), 1.7 (s, 3H).

^{13}C NMR (126 MHz, Chloroform-*d*) δ 170.1, 169.9, 163.4, 163.3, 141.3, 141.0, 133.2, 132.3, 119.3, 118.1, 112.8, 112.6, 63.6, 63.4, 60.6, 53.5, 36.6, 33.0, 32.4, 31.3, 27.1, 22.3, 22.2.

HRMS (ESI) m/z: $[M+H]^+$ Calcd. for $C_{12}H_{20}NO_3^+$ 226.1438; Found: 226.1436.

benzyl 2-(*N*-methylformamido)pent-4-enoate (3g)

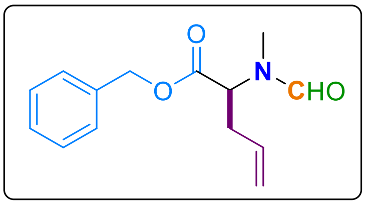

Following the **general procedure 4** on 0.2 mmol scale, yellow oil, yield: 91% (45 mg). R_f = 0.2 (silica gel, PE: EtOAc = 5:1, v/v), column chromatography (silica gel, PE: EtOAc= 5: 1, v/v).

^{1}H NMR (500 MHz, Chloroform-*d*) δ 8.1 (d, *J* = 13.3 Hz, 1H), 7.4 – 7.3 (m, 5H), 5.7 – 5.6 (m, 1H), 5.2 – 5.1 (m, 4H), 4.2 (dd, *J* = 10.4, 5.2 Hz, 1H), 2.8 (d, *J* = 45.9 Hz, 3H), 2.8 – 2.7 (m, 1H), 2.6 – 2.5 (m, 1H).

^{13}C NMR (126 MHz, Chloroform-*d*) δ 170.0, 169.8, 163.5, 163.3, 135.3, 133.1, 132.2, 128.7, 128.7, 128.6, 128.4, 128.4, 128.2, 119.4, 118.2, 67.4, 67.1, 60.6, 53.6, 33.0, 32.4, 31.3, 27.1.

HRMS (ESI) m/z: $[M+H]^+$ Calcd. for $C_{14}H_{18}NO_3^+$ 248.1281; Found: 248.1279.

4-methylbenzyl 2-(*N*-methylformamido)pent-4-enoate (3h)

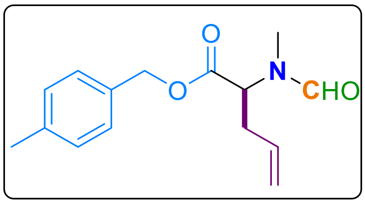

Following the **general procedure 4** on 0.2 mmol scale, yellow oil, yield: 92% (48 mg). R_f = 0.2 (silica gel, PE: EtOAc = 5:1, v/v), column chromatography (silica gel, PE: EtOAc= 5: 1, v/v).

^{1}H NMR (500 MHz, Chloroform-*d*) δ 8.1 (d, *J* = 16.8 Hz, 1H), 7.3 – 7.0 (m, 4H), 5.7 – 5.6 (m, 1H), 5.2 – 5.1 (m, 4H), 4.2 (dd, *J* = 10.4, 5.2 Hz, 1H), 2.8 (d, *J* = 45.2 Hz, 3H), 2.8 – 2.7 (m, 1H), 2.5 (tdd, *J* = 15.1, 10.6, 7.8 Hz, 1H), 2.3 (s, 3H).

^{13}C NMR (126 MHz, Chloroform-*d*) δ 170.0, 169.8, 163.5, 163.3, 138.6, 138.3, 133.2, 132.3, 132.2, 132.0, 129.4, 129.3, 128.6, 128.4, 119.4, 118.1, 67.4, 67.1, 60.6, 53.6, 33.0, 32.4, 31.3, 27.1, 21.2.

HRMS (ESI) m/z: $[M+H]^+$ Calcd. for $C_{15}H_{20}NO_3^+$ 262.1438; Found: 262.1439.

4-(*tert*-butyl)benzyl 2-(*N*-methylformamido)pent-4-enoate (3i)

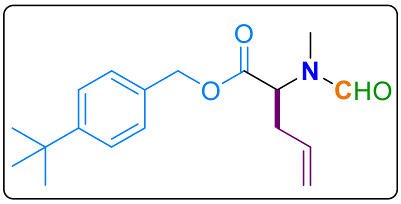

Following the **general procedure 4** on 0.2 mmol scale, yellow oil, yield: 63% (38 mg). R_f = 0.2 (silica gel, PE: EtOAc = 5:1, v/v), column chromatography (silica gel, PE: EtOAc= 5: 1, v/v).

^{1}H NMR (500 MHz, Chloroform-*d*) δ 8.1 (d, *J* = 17.9 Hz, 1H), 7.5 – 7.2 (m, 4H), 5.8 – 5.6 (m, 1H), 5.2 – 5.1 (m, 4H), 4.2 (dd, *J* = 10.4, 5.2 Hz, 1H), 2.9 (d, *J* = 45.6 Hz, 3H), 2.8 – 2.7 (m, 1H), 2.6 – 2.5 (m, 1H), 1.3 (s, 9H).

^{13}C NMR (126 MHz, Chloroform-*d*) δ 170.1, 169.8, 163.5, 163.3, 133.2, 132.3, 132.2, 132.0, 128.3, 128.1, 125.7, 125.6, 119.4, 118.1, 67.3, 67.0, 60.7, 53.6, 34.7, 34.6, 33.0, 32.4, 31.3, 31.3, 31.3, 27.1.

HRMS (ESI) m/z: $[M+H]^+$ Calcd. for $C_{18}H_{26}NO_3^+$ 304.1907; Found: 304.1903.

4-(benzyloxy)benzyl 2-(*N*-methylformamido)pent-4-enoate (3j)

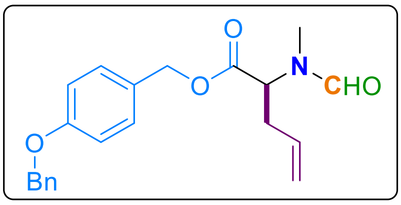

Following the **general procedure 4** on 0.2 mmol scale, yellow oil, yield: 49% (35 mg). R_f = 0.2 (silica gel, PE: EtOAc = 5:1, v/v), column chromatography (silica gel, PE: EtOAc= 5: 1, v/v).

^{1}H NMR (500 MHz, Chloroform-*d*) δ 8.1 (d, *J* = 18.2 Hz, 1H), 7.5 – 7.4 (m, 4H), 7.3 (d, *J* = 7.2 Hz, 1H), 7.3 – 7.2 (m, 2H), 7.0 (dd, *J* = 8.7, 2.9 Hz, 2H), 5.6 (qdd, *J* = 17.1, 9.0, 6.2 Hz, 1H), 5.1 – 5.1 (m, 4H), 5.1 (s, 2H), 4.1 (dd, *J* = 10.4, 5.2 Hz, 1H), 2.8 (d, *J* = 44.6 Hz, 3H), 2.8 – 2.7 (m, 1H), 2.5 (ddd, *J* = 15.1, 7.4, 3.1 Hz, 1H).

^{13}C NMR (126 MHz, Chloroform-*d*) δ 170.1, 163.5, 163.3, 159.1, 133.2, 132.2, 130.3, 130.2, 128.7, 128.1, 128.1, 127.8, 127.5, 119.4, 118.2, 115.0, 114.9, 70.0, 67.3, 67.0, 60.6, 53.5, 33.0, 32.4, 31.3, 27.1.

HRMS (ESI) m/z: $[M+H]^+$ Calcd. for $C_{21}H_{24}NO_3^+$ 354.1700; Found: 354.1698.

[1,1'-biphenyl]-4-ylmethyl 2-(*N*-methylformamido)pent-4-enoate (3k)

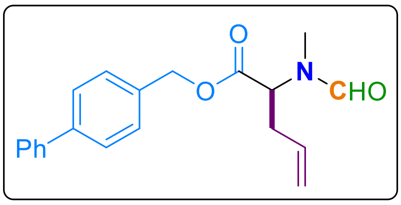

Following the **general procedure 4** on 0.2 mmol scale, yellow oil, yield: 76% (49 mg). R_f = 0.2 (silica gel, PE: EtOAc = 5:1, v/v), column chromatography (silica gel, PE: EtOAc= 5: 1, v/v).

^{1}H NMR (500 MHz, Chloroform-*d*) δ 8.1 (d, *J* = 12.7 Hz, 1H), 7.6 – 7.5 (m, 4H), 7.5 – 7.3 (m, 5H), 5.8 – 5.6 (m, 1H), 5.3 – 5.2 (m, 3H), 5.2 – 5.1 (m, 3H), 4.2 (dd, *J* = 10.4, 5.2 Hz, 1H), 2.9 (d, *J* = 42.5 Hz, 3H), 2.8 – 2.7 (m, 1H), 2.5 (dtd, *J* = 15.1, 11.0, 7.9 Hz, 1H).

^{13}C NMR (126 MHz, Chloroform-*d*) δ 170.1, 169.8, 163.5, 163.3, 140.6, 134.3, 134.0, 133.1, 132.2, 128.9, 128.9, 128.9, 128.7, 127.6, 127.5, 127.5, 127.4, 127.1, 119.5, 118.2, 67.2, 66.9, 60.7, 53.6, 33.0, 32.4, 31.4, 27.1.

HRMS (ESI) m/z: $[M+H]^+$ Calcd. for $C_{20}H_{22}NO_3^+$ 324.1594; Found: 324.1593.

2-iodobenzyl 2-(*N*-methylformamido)pent-4-enoate (3l)

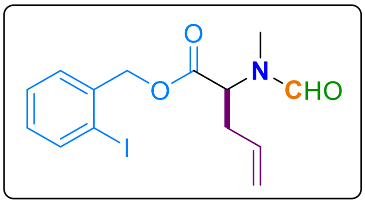

Following the **general procedure 4** on 0.2 mmol scale, yellow oil, yield: 65% (48 mg). R_f = 0.2 (silica gel, PE: EtOAc = 5:1, v/v), column chromatography (silica gel, PE: EtOAc= 5: 1, v/v).

^{1}H NMR (500 MHz, Chloroform-*d*) δ 8.1 (d, *J* = 11.6 Hz, 1H), 7.9 (t, *J* = 7.8 Hz, 1H), 7.4 – 7.3 (m, 2H), 7.0 (td, *J* = 8.6, 8.1, 3.6 Hz, 1H), 5.8 – 5.6 (m, 1H), 5.2 – 5.1 (m, 4H), 4.2 (dd, *J* = 10.4, 5.2 Hz, 1H), 2.9 (d, *J* = 45.4 Hz, 3H), 2.8 (d, *J* = 14.4 Hz, 1H), 2.6 – 2.5 (m, 1H).

^{13}C NMR (126 MHz, Chloroform-*d*) δ 169.8, 169.6, 163.5, 163.3, 139.8, 139.6, 137.7, 137.4, 133.1, 132.1, 130.4, 130.2, 130.1, 129.9, 128.5, 128.5, 119.5, 118.3, 98.5, 71.2, 70.9, 60.6, 53.5, 33.1, 32.4, 31.5, 27.2.

HRMS (ESI) m/z: $[M+H]^+$ Calcd. for $C_{14}H_{17}INO_3^+$ 374.0248; Found: 374.0247.

3,5-dichlorobenzyl 2-(*N*-methylformamido)pent-4-enoate (3m)

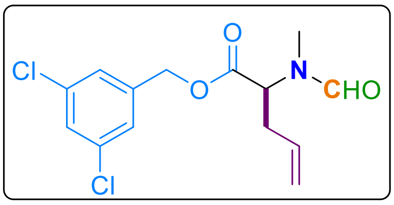

Following the **general procedure 4** on 0.2 mmol scale, yellow oil, yield: 82% (51 mg). R_f = 0.2 (silica gel, PE: EtOAc = 5:1, v/v), column chromatography (silica gel, PE: EtOAc= 5: 1, v/v).

^{1}H NMR (500 MHz, Chloroform-*d*) δ 8.1 (d, *J* = 11.2 Hz, 1H), 7.3 (dt, *J* = 9.5, 2.0 Hz, 1H), 7.2 (d, *J* = 1.9 Hz, 2H), 5.7 – 5.6 (m, 1H), 5.1 – 5.1 (m, 4H), 4.2 (dd, *J* = 10.3, 5.4 Hz, 1H), 2.9 (d, *J* = 51.4 Hz, 3H), 2.8 – 2.7 (m, 1H), 2.6 – 2.5 (m, 1H).
^{13}C NMR (126 MHz, Chloroform-*d*) δ 169.8, 163.4, 163.2, 138.6, 135.3, 135.2, 132.9, 131.9, 128.8, 128.6, 126.6, 126.4, 119.7, 118.4, 65.7, 65.4, 60.5, 53.6, 33.0, 32.4, 31.5, 27.1.
HRMS (ESI) m/z: $[M+H]^+$ Calcd. for $C_{14}H_{16}Cl_2NO_3^+$ 316.0502; Found: 316.0503.

cinnamyl 2-(*N*-methylformamido)pent-4-enoate (3n)

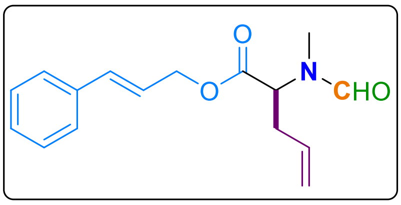

Following the **general procedure 4** on 0.2 mmol scale, yellow oil, yield: 88% (48 mg). R_f = 0.2 (silica gel, PE: EtOAc = 5:1, v/v), column chromatography (silica gel, PE: EtOAc= 5: 1, v/v).
^{1}H NMR (500 MHz, Chloroform-*d*) δ 8.1 (d, *J* = 11.5 Hz, 1H), 7.4 – 7.2 (m, 5H), 6.7 (dd, *J* = 15.9, 6.2 Hz, 1H), 6.3 (dtd, *J* = 15.9, 6.6, 1.2 Hz, 1H), 5.8 – 5.6 (m, 1H), 5.2 – 5.1 (m, 2H), 4.8 – 4.7 (m, 2H), 4.2 (dd, *J* = 10.4, 5.2 Hz, 1H), 2.9 (d, *J* = 43.6 Hz, 3H), 2.8 – 2.7 (m, 1H), 2.6 – 2.4 (m, 1H).
^{13}C NMR (126 MHz, Chloroform-*d*) δ 163.5, 163.3, 135.5, 134.9, 133.2, 132.2, 128.7, 128.7, 128.4, 128.3, 126.7, 126.7, 122.4, 122.0, 119.4, 118.2, 66.3, 66.0, 60.7, 53.6, 33.0, 32.4, 31.4, 27.1.
HRMS (ESI) m/z: $[M+H]^+$ Calcd. for $C_{16}H_{20}NO_3^+$ 274.1438; Found: 274.1434.

2-(benzyloxy)ethyl 2-(*N*-methylformamido)pent-4-enoate (3o)

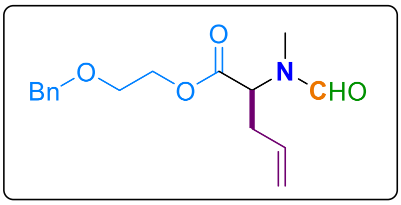

Following the **general procedure 4** on 0.2 mmol scale, yellow oil, yield: 61% (35 mg). R_f = 0.2 (silica gel, PE: EtOAc = 5:1, v/v), column chromatography (silica gel, PE: EtOAc= 5: 1, v/v).
^{1}H NMR (500 MHz, Chloroform-*d*) δ 8.1 (d, *J* = 11.4 Hz, 1H), 7.3 (ddt, *J* = 11.1, 8.5, 5.6 Hz, 5H), 5.8 – 5.6 (m, 1H), 5.2 – 5.1 (m, 2H), 4.5 (s, 2H), 4.3 (qq, *J* = 11.9, 7.2, 6.0 Hz, 2H), 4.1 (dd, *J* = 10.5, 5.1 Hz, 1H), 3.7 (t, *J* = 4.9 Hz, 2H), 2.9 (d, *J* = 38.5 Hz, 3H), 2.8 – 2.7 (m, 1H), 2.6 – 2.4 (m, 1H).
^{13}C NMR (126 MHz, Chloroform-*d*) δ 170.1, 169.9, 163.5, 163.3, 137.7 (d, *J* = 20.7 Hz), 133.2, 132.3, 128.5, 128.5, 127.9, 127.8, 127.8, 127.7, 119.4, 118.2, 73.2, 73.1, 67.7, 67.5, 64.6, 64.4, 60.5, 53.5, 33.0, 32.4, 31.3, 27.1.
HRMS (ESI) m/z: $[M+H]^+$ Calcd. for $C_{16}H_{22}NO_4^+$ 292.1543; Found: 292.1540.

N-(1-cyclopropyl-1-oxopent-4-en-2-yl)-*N*-methylformamide (3p)

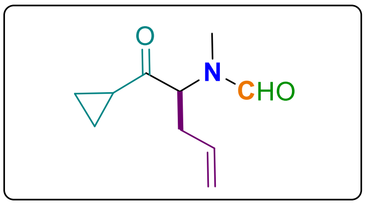

Following the **general procedure 4** on 0.2 mmol scale, yellow oil, yield: 86% (31 mg). R_f = 0.2 (silica gel, PE: EtOAc = 5:1, v/v), column chromatography (silica gel, PE: EtOAc= 5: 1, v/v).

^{1}H NMR (500 MHz, Chloroform-*d*) δ 8.1 (s, 1H), 5.7 – 5.5 (m, 1H), 5.1 – 5.0 (m, 2H), 4.2 (dd, *J* = 10.2, 4.7 Hz, 0H), 2.7 (d, *J* = 45.2 Hz, 3H), 2.6 – 2.6 (m, 1H), 2.4 – 2.3 (m, 1H), 1.9 (dddd, *J* = 9.2, 7.8, 4.6, 3.2 Hz, 1H), 1.0 – 0.8 (m, 4H).

^{13}C NMR (126 MHz, Chloroform-*d*) δ 207.0, 206.8, 163.7, 163.4, 133.4, 132.8, 119.1, 119.0, 117.8, 66.6, 59.4, 31.4, 31.1, 30.5, 27.3, 18.4, 18.2, 12.1, 12.1, 11.9, 11.9, 11.5, 11.5.

HRMS (ESI) m/z: $[M+H]^+$ Calcd. for $C_{10}H_{16}NO_2^+$ 182.1176; Found: 182.1177.

N-(6,6-dimethyl-5-oxohept-1-en-4-yl)-*N*-methylformamide (3q)

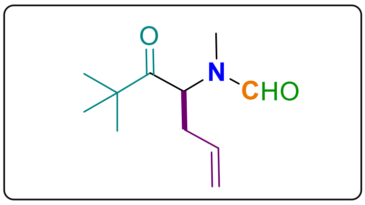

Following the **general procedure 4** on 0.2 mmol scale, yellow oil, yield: 77% (30 mg). R_f = 0.2 (silica gel, PE: EtOAc = 5:1, v/v), column chromatography (silica gel, PE: EtOAc= 5: 1, v/v).

^{1}H NMR (500 MHz, Chloroform-*d*) δ 8.1 (d, *J* = 72.1 Hz, 1H), 5.7 – 5.5 (m, 2H), 5.2 – 5.0 (m, 1H), 4.5 (d, *J* = 3.0 Hz, 1H), 2.8 (d, *J* = 37.0 Hz, 3H), 2.5 – 2.3 (m, 2H), 1.1 (dd, *J* = 8.1, 1.3 Hz, 9H).

^{1}H NMR (500 MHz, Chloroform-*d*) δ 8.1 (d, *J* = 72.1 Hz, 1H), 5.7 – 5.5 (m, 2H), 5.2 – 5.0 (m, 2H), 4.5 (d, *J* = 3.0 Hz, 0H), 2.8 (d, *J* = 37.0 Hz, 3H), 2.5 – 2.3 (m, 2H), 1.1 (dd, *J* = 8.1, 1.3 Hz, 9H).

HRMS (ESI) m/z: $[M+H]^+$ Calcd. for $C_{11}H_{20}NO_2^+$ 198.1489; Found: 198.1488.

N-(adamantan-1-yl)-1-oxopent-4-en-2-yl)-*N*-methylformamide (3r)

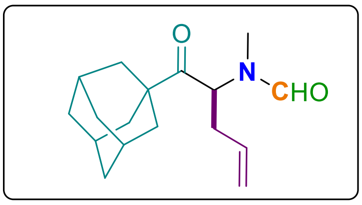

Following the **general procedure 4** on 0.2 mmol scale, yellow oil, yield: 83% (23 mg). R_f = 0.2 (silica gel, PE: EtOAc = 5:1, v/v), column chromatography (silica gel, PE: EtOAc= 5: 1, v/v).

^{1}H NMR (500 MHz, Chloroform-*d*) δ 8.0 (d, *J* = 83.4 Hz, 1H), 5.6 – 5.5 (m, 1H), 5.1 – 5.0 (m, 2H), 4.5 (dd, *J* = 8.9, 6.2 Hz, 1H), 2.7 (d, *J* = 43.5 Hz, 3H), 2.5 – 2.2 (m, 2H), 2.0 (dd, *J* = 8.7, 5.6 Hz, 3H), 1.8 – 1.6 (m, 12H).

^{13}C NMR (126 MHz, Chloroform-*d*) δ 211.1, 209.5, 163.4, 162.6, 133.1, 132.4, 119.4, 118.4, 58.0, 50.9, 46.7, 37.9, 37.3, 36.3, 36.2, 33.2, 32.3, 30.8, 27.6, 27.6, 26.9.

HRMS (ESI) m/z: $[M+H]^+$ Calcd. for $C_{17}H_{26}NO_2^+$ 276.1958; Found: 276.1960.

N-methyl-*N*-(1-oxo-1-phenylpent-4-en-2-yl)formamide (3s)

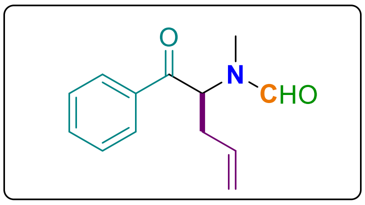

Following the **general procedure 4** on 0.2 mmol scale, yellow oil, yield: 79% (34 mg). R_f = 0.2 (silica gel, PE: EtOAc = 5:1, v/v), column chromatography (silica gel, PE: EtOAc= 5: 1, v/v).

^{1}H NMR (500 MHz, Chloroform-*d*) δ 8.2 (d, *J* = 115.8 Hz, 1H), 8.0 – 7.8 (m, 2H), 7.6 – 7.5 (m, 1H), 7.5 – 7.4 (m, 2H), 6.0 (dd, *J* = 9.5, 5.8 Hz, 1H), 5.7 (dddd, *J* = 17.2, 10.2, 8.2, 5.5 Hz, 1H), 5.2 – 5.1 (m, 2H), 2.7 (d, *J* = 11.7 Hz, 3H), 2.7 (dtt, *J* = 14.5, 5.6, 1.5 Hz, 1H), 2.6 – 2.5 (m, 1H).

^{13}C NMR (126 MHz, Chloroform-*d*) δ 197.2, 196.0, 163.5, 162.8, 134.9, 133.9, 133.3, 129.1, 128.8, 128.6, 128.3, 119.6, 118.3, 61.2, 54.0, 32.6, 31.4, 30.6, 27.2.

HRMS (ESI) m/z: $[M+H]^+$ Calcd. for $C_{13}H_{16}NO_2^+$ 218.1176; Found: 218.1171.

N-(1-([1,1'-biphenyl]-4-yl)-1-oxopent-4-en-2-yl)-*N*-methylformamide (3t)

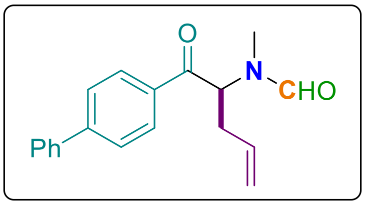

Following the **general procedure 4** on 0.2 mmol scale, yellow oil, yield: 72% (42 mg). R_f = 0.2 (silica gel, PE: EtOAc = 5:1, v/v), column chromatography (silica gel, PE: EtOAc= 5: 1, v/v).

^{1}H NMR (500 MHz, Chloroform-*d*) δ 8.1 – 8.1 (m, 2H), 8.1 (s, 1H), 7.7 – 7.7 (m, 2H), 7.7 – 7.6 (m, 2H), 7.5 (td, *J* = 6.8, 1.8 Hz, 2H), 7.4 (d, *J* = 7.3 Hz, 1H), 6.0 (dd, *J* = 9.5, 5.8 Hz, 1H), 5.8 – 5.7 (m, 1H), 5.3 – 5.2 (m, 1H), 5.1 (dq, *J* = 10.2, 1.3 Hz, 1H), 2.8 (d, *J* = 7.1 Hz, 3H), 2.7 (dtt, *J* = 14.8, 5.7, 1.6 Hz, 1H), 2.7 – 2.5 (m, 1H).

^{13}C NMR (126 MHz, Chloroform-*d*) δ 196.6, 195.5, 162.8, 146.5, 139.7, 133.5, 133.4, 129.3, 129.1, 129.0, 129.0, 128.6, 128.4, 127.7, 127.5, 127.3, 127.3, 118.4, 61.3, 54.0, 32.7, 31.5, 30.7.

HRMS (ESI) m/z: $[M+H]^+$ Calcd. for $C_{19}H_{20}NO_2^+$ 294.1489; Found: 294.1488.

N-(1-(4-methoxyphenyl)-1-oxopent-4-en-2-yl)-*N*-methylformamide (3u)

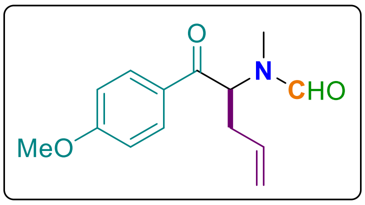

Following the **general procedure 4** on 0.2 mmol scale, yellow oil, yield: 81% (40 mg). R_f = 0.2 (silica gel, PE: EtOAc = 5:1, v/v), column chromatography (silica gel, PE: EtOAc= 5: 1, v/v).

^{1}H NMR (500 MHz, Chloroform-*d*) δ 8.0 (s, 1H), 8.0 – 8.0 (m, 2H), 6.9 (t, *J* = 8.4 Hz, 2H), 5.9 (dd, *J* = 9.4, 5.9 Hz, 1H), 5.7 (dddd, *J* = 17.3, 10.2, 8.2, 5.5 Hz, 1H), 5.2 – 5.1 (m, 1H), 5.0 (dt, *J* = 10.2, 1.4 Hz, 1H), 3.8 (d, *J* = 6.7 Hz, 3H), 2.7 (d, *J* = 7.6 Hz, 3H), 2.6 (ddt, *J* = 13.1, 5.8, 2.8 Hz, 1H), 2.6 – 2.5 (m, 1H).

^{13}C NMR (126 MHz, Chloroform-*d*) δ 195.2, 194.2, 164.1, 163.4, 162.7, 133.5, 132.8, 131.1, 130.7, 127.8, 127.8, 119.4, 118.2, 114.2, 114.0, 60.8, 55.5, 53.5, 32.8, 31.4, 30.5, 27.2.

HRMS (ESI) m/z: $[M+H]^+$ Calcd. for $C_{14}H_{18}NO_3^+$ 248.1281; Found: 248.1278.

N-(1-(4-bromophenyl)-1-oxopent-4-en-2-yl)-*N*-methylformamide (3v)

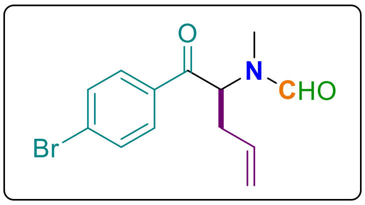

Following the **general procedure 4** on 0.2 mmol scale, yellow oil, yield: 73% (43 mg). R_f = 0.2 (silica gel, PE: EtOAc = 5:1, v/v), column chromatography (silica gel, PE: EtOAc= 5: 1, v/v).

^{1}H NMR (500 MHz, Chloroform-*d*) δ 8.1 (d, *J* = 111.6 Hz, 1H), 7.9 – 7.7 (m, 2H), 7.6 – 7.5 (m, 2H), 5.9 (dd, *J* = 9.5, 5.8 Hz, 1H), 5.8 – 5.6 (m, 1H), 5.2 – 5.1 (m, 2H), 2.7 (d, *J* = 13.2 Hz, 3H), 2.7 – 2.5 (m, 2H).

^{13}C NMR (126 MHz, Chloroform-*d*) δ 196.2, 195.1, 162.8, 133.6, 133.1, 132.4, 132.2, 130.1, 129.8, 129.2, 118.5, 61.3, 53.9, 32.6, 31.3, 30.6.

HRMS (ESI) m/z: $[M+H]^+$ Calcd. for $C_{13}H_{15}BrNO_2^+$ 296.0281; Found: 296.0280.

N-(1-(4-hydroxyphenyl)-1-oxopent-4-en-2-yl)-*N*-methylformamide (3w)

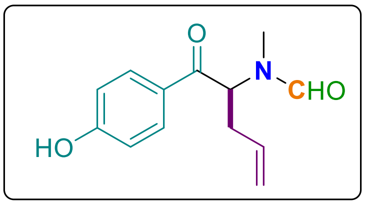

Following the **general procedure 4** on 0.2 mmol scale, yellow oil, yield: 49% (23 mg). R_f = 0.2 (silica gel, PE: EtOAc = 5:1, v/v), column chromatography (silica gel, PE: EtOAc= 5: 1, v/v).

^{1}H NMR (500 MHz, Chloroform-*d*) δ 9.7 (s, 1H), 8.1 (s, 1H), 8.0 (d, *J* = 8.8 Hz, 2H), 6.9 (d, *J* = 8.8 Hz, 2H), 6.0 (dd, *J* = 9.1, 6.2 Hz, 1H), 5.7 (dddd, *J* = 17.2, 10.2, 8.1, 5.8 Hz, 1H), 5.2 (dd, *J* = 17.1, 1.5 Hz, 1H), 5.1 (dd, *J* = 10.4, 1.4 Hz, 1H), 2.8 (s, 3H), 2.7 – 2.6 (m, 2H).

^{13}C NMR (126 MHz, Chloroform-*d*) δ 194.1, 163.6, 163.1, 133.0, 131.4, 131.0, 126.7, 118.6, 115.8, 53.4, 31.3, 31.0.

HRMS (ESI) m/z: $[M+H]^+$ Calcd. for $C_{13}H_{16}NO_3^+$ 234.1125; Found: 234.1123.

N-(1-(3-methoxyphenyl)-1-oxopent-4-en-2-yl)-*N*-methylformamide (3x)

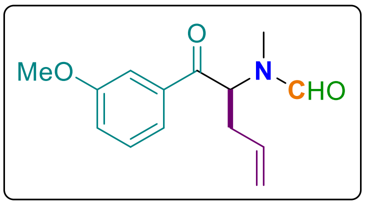

Following the **general procedure 4** on 0.2 mmol scale, yellow oil, yield: 76% (37 mg). R_f = 0.2 (silica gel, PE: EtOAc = 5:1, v/v), column chromatography (silica gel, PE: EtOAc= 5: 1, v/v).

^{1}H NMR (500 MHz, Chloroform-*d*) δ 8.2 (d, *J* = 106.4 Hz, 1H), 7.7 – 7.3 (m, 3H), 7.1 (dd, *J* = 8.2, 2.6 Hz, 1H), 6.0 (dd, *J* = 9.6, 5.8 Hz, 1H), 5.8 – 5.7 (m, 1H), 5.2 – 5.1 (m, 2H), 3.8 (d, *J* = 1.4 Hz, 3H), 2.8 – 2.7 (m, 3H), 2.7 – 2.6 (m, 1H), 2.6 (dt, *J* = 15.1, 8.8 Hz, 1H).

^{13}C NMR (126 MHz, Chloroform-*d*) δ 196.9, 195.9, 163.4, 162.8, 159.9, 136.1, 133.3, 132.6, 129.9, 121.2, 120.9, 120.6, 120.2, 119.6, 118.4, 112.9, 112.3, 61.3, 55.5, 54.1, 32.7, 31.4, 30.6, 27.2.

HRMS (ESI) m/z: $[M+H]^+$ Calcd. for $C_{14}H_{18}NO_3^+$ 248.1281; Found: 248.1280.

N-methyl-*N*-(1-oxo-1-(thiophen-2-yl)pent-4-en-2-yl)formamide (3y)

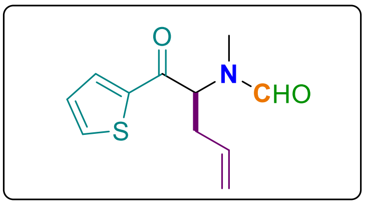

Following the **general procedure 4** on 0.2 mmol scale, yellow oil, yield: 86% (38 mg). R_f = 0.2 (silica gel, PE: EtOAc = 5:1, v/v), column chromatography (silica gel, PE: EtOAc= 5: 1, v/v).

^{1}H NMR (500 MHz, Chloroform-*d*) δ 8.2 (d, *J* = 107.7 Hz, 1H), 8.0 (dd, *J* = 3.8, 1.1 Hz, 1H), 7.8 – 7.6 (m, 1H), 7.1 (dd, *J* = 4.9, 3.8 Hz, 1H), 5.9 (dd, *J* = 9.3, 6.2 Hz, 1H), 5.7 (dddd, *J* = 17.1, 10.3, 8.1, 5.8 Hz, 1H), 5.3 – 5.2 (m, 1H), 5.1 (dq, *J* = 10.2, 1.3 Hz, 1H), 2.8 (d, *J* = 20.7 Hz, 3H), 2.8 – 2.5 (m, 2H).

^{13}C NMR (126 MHz, Chloroform-*d*) δ 189.1, 163.3, 162.8, 142.3, 135.2, 135.1, 133.8, 133.0, 132.9, 132.4, 128.6, 119.7, 118.5, 62.7, 54.4, 32.5, 31.2, 30.6, 27.2.

HRMS (ESI) m/z: $[M+H]^+$ Calcd. for $C_{11}H_{14}NO_2S^+$ 224.0740; Found: 224.0743.

N-methyl-*N*-(1-(naphthalen-2-yl)-1-oxopent-4-en-2-yl)formamide (3z)

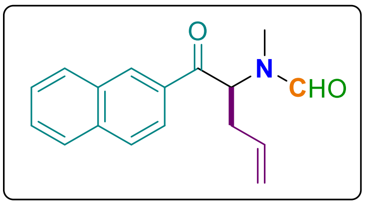

Following the **general procedure 4** on 0.2 mmol scale, yellow oil, yield: 69% (37 mg). R_f = 0.2 (silica gel, PE: EtOAc = 5:1, v/v), column chromatography (silica gel, PE: EtOAc= 5: 1, v/v).

^{1}H NMR (500 MHz, Chloroform-*d*) δ 8.6 (d, *J* = 1.8 Hz, 1H), 8.1 (s, 1H), 8.0 – 8.0 (m, 2H), 8.0 – 7.8 (m, 3H), 7.6 – 7.5 (m, 2H), 6.2 (dd, *J* = 9.5, 5.8 Hz, 1H), 5.8 (m, 1H), 5.3 – 5.1 (m, 1H), 5.1 (dd, *J* = 10.3, 1.6 Hz, 1H), 2.8 (s, 3H), 2.8 – 2.7 (m, 1H), 2.7 – 2.6 (m, 1H).

^{13}C NMR (126 MHz, Chloroform-*d*) δ 197.1, 196.0, 163.5, 162.8, 135.9, 133.4, 132.7, 132.5, 132.1, 130.8, 130.1, 130.0, 129.7, 129.1, 128.9, 128.7, 127.9, 127.7, 126.9, 123.9, 123.7, 118.4, 61.3, 54.0, 32.8, 31.5, 30.7, 27.3.

HRMS (ESI) m/z: $[M+H]^+$ Calcd. for $C_{17}H_{18}NO_2^+$ 268.1332; Found: 268.1334.

N-(1-cyanobut-3-en-1-yl)-N-methylformamide (3za)

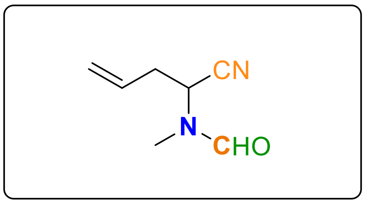

Following the **general procedure 4** on 0.2 mmol scale, yellow oil, yield: 69% (37 mg). R_f = 0.2 (silica gel, PE: EtOAc = 5:1, v/v), column chromatography (silica gel, PE: EtOAc= 5: 1, v/v).

^{1}H NMR (500 MHz, Chloroform-*d*) δ 8.1 (d, *J* = 16.8 Hz, 1H), 5.7 (m, 1H), 5.5 (t, *J* = 7.9 Hz, 1H), 5.3 – 5.2 (m, 2H), 3.0 (d, *J* = 42.9 Hz, 3H), 2.7 – 2.6 (m, 1H), 2.6 – 2.5 (m, 1H).

^{13}C NMR (126 MHz, Chloroform-*d*) δ 162.2, 161.7, 130.3, 129.6, 121.8, 120.7, 116.4, 116.2, 49.9, 42.6, 36.0, 34.9, 31.0, 26.9.

HRMS (ESI) m/z: $[M+H]^+$ Calcd. for $C_{11}H_{11}N_2O^+$ 139.0866; Found: 139.0863.

diethyl (1-(N-methylformamido)but-3-en-1-yl)phosphonate (3zb)

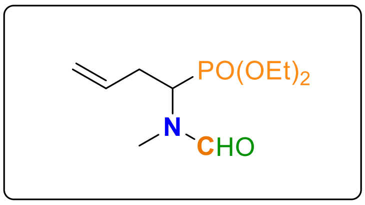

Following the **general procedure 4** on 0.2 mmol scale, yellow oil, yield: 31% (37 mg). R_f = 0.2 (silica gel, PE: EtOAc = 5:1, v/v), column chromatography (silica gel, PE: EtOAc= 1: 1, v/v).

^{1}H NMR (500 MHz, Chloroform-*d*) δ 8.0 (d, *J* = 35.8 Hz, 1H), 5.6 (m, 1H), 5.2 – 5.1 (m, 2H), 4.9 (m, 1H), 4.2 – 4.1 (m, 4H), 3.7 (m, 1H), 2.9 (d, *J* = 32.4 Hz, 3H), 2.7 – 2.5 (m, 2H), 1.3 (m, 6H).

^{13}C NMR (126 MHz, Chloroform-*d*) δ 163.6, 163.5, 163.4, 163.3, 133.2, 133.1, 132.3, 119.4, 118.2, 63.1, 62.6, 62.5, 47.6, 46.4, 31.3, 30.6, 30.5, 29.9, 27.6, 16.6, 16.5, 16.4,

^{31}P NMR (202 MHz, Chloroform-*d*) δ 23.0, 22.1.

HRMS (ESI) m/z: $[M+H]^+$ Calcd. for $C_{10}H_{21}NO_4P^+$ 250.1203; Found: 250.1204.

methyl 2-methyl-2-(*N*-methylformamido)pent-4-enoate (6a)

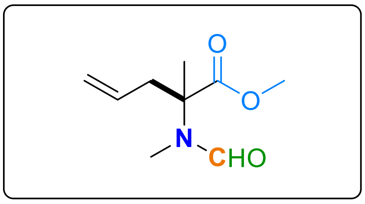

Following the **general procedure 5** on 0.2 mmol scale, yellow oil, yield: 68% (25 mg). R_f = 0.2 (silica gel, PE: EtOAc = 5:1, v/v), column chromatography (silica gel, PE: EtOAc= 5: 1, v/v).

^{1}H NMR (500 MHz, Chloroform-*d*) δ 8.1 (d, *J* = 135.8 Hz, 1H), 5.8 – 5.6 (m, 1H), 5.2 – 5.1 (m, 2H), 3.7 (d, *J* = 25.1 Hz, 3H), 3.0 – 2.8(d, 3H), 2.9 (ddt, *J* = 13.8, 7.3, 1.2 Hz, 1H), 2.6 – 2.5 (m, 1H), 1.5 (d, *J* = 73.3 Hz, 3H).

^{13}C NMR (126 MHz, Chloroform-*d*) δ 173.5, 173.2, 162.8, 162.0, 132.2, 130.7, 120.8, 119.7, 64.5, 61.7, 52.9, 52.3, 40.2, 39.6, 32.4, 28.4, 22.0, 20.5.

HRMS (ESI) m/z: $[M+H]^+$ Calcd. for $C_9H_{16}NO_3^+$ 186.1125; Found: 186.1126.

ethyl 2-methyl-2-(*N*-methylformamido)pent-4-enoate (6b)

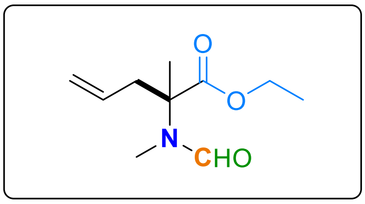

Following the **general procedure 5** on 0.2 mmol scale, yellow oil, yield: 66% (26 mg). R_f = 0.2 (silica gel, PE: EtOAc = 5:1, v/v), column chromatography (silica gel, PE: EtOAc= 5: 1, v/v).

^{1}H NMR (500 MHz, Chloroform-*d*) δ 8.1 (d, *J* = 134.8 Hz, 1H), 5.8 – 5.5 (m, 1H), 5.2 – 5.1 (m, 2H), 4.3 – 4.1 (m, 2H), 3.0 (d, 3H), 2.8 – 2.7 (m, 1H), 2.6 – 2.5 (m, 1H), 1.5 (d, *J* = 71.5 Hz, 3H), 1.2 (dt, *J* = 14.2, 7.1 Hz, 3H).

^{13}C NMR (126 MHz, Chloroform-*d*) δ 173.0, 172.6, 162.8, 162.0, 132.3, 130.8, 120.7, 119.6, 64.5, 61.9, 61.2, 40.1, 39.5, 32.4, 28.4, 21.9, 20.5, 14.1.

HRMS (ESI) m/z: $[M+H]^+$ Calcd. for $C_{10}H_{18}NO_3^+$ 200.1281; Found: 200.1282.

4-iodobenzyl 2-methyl-2-(*N*-methylformamido)pent-4-enoate (6c)

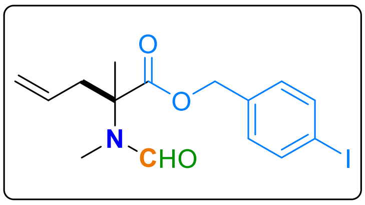

Following the **general procedure 5** on 0.2 mmol scale, yellow oil, yield: 68% (52 mg). R_f = 0.2 (silica gel, PE: EtOAc = 5:1, v/v), column chromatography (silica gel, PE: EtOAc= 5: 1, v/v).

^{1}H NMR (500 MHz, Chloroform-*d*) δ 8.1 (d, *J* = 131.4 Hz, 1H), 7.7 – 7.6 (m, 2H), 7.2 – 7.0 (m, 2H), 5.7 (ddt, *J* = 16.6, 10.6, 7.4 Hz, 1H), 5.2 – 5.0 (m, 4H), 3.0 (d, 3H), 2.9 (dd, *J* = 13.8, 7.3 Hz, 1H), 2.6 – 2.5 (m, 1H), 1.5 (d, *J* = 67.7 Hz, 3H).

^{13}C NMR (126 MHz, Chloroform-*d*) δ 172.8, 172.4, 162.8, 162.0, 137.9, 137.6, 135.6, 132.0, 130.5, 130.2, 130.2, 120.9, 119.9, 94.5, 93.9, 66.9, 66.3, 64.5, 61.7, 40.1, 39.6, 32.4, 28.5, 21.9, 20.5.

HRMS (ESI) m/z: $[M+H]^+$ Calcd. for $C_{15}H_{19}INO_3^+$ 388.0404; Found: 388.0403.

ethyl 2-(*N*-methylformamido)-2-phenylpent-4-enoate (6d)

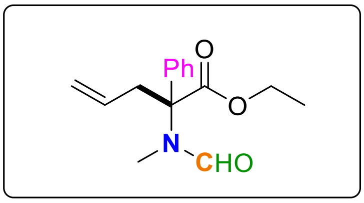

Following the **general procedure 5** on 0.2 mmol scale, yellow oil, yield: 54% (28 mg). R_f = 0.2 (silica gel, PE: EtOAc = 5:1, v/v), column chromatography (silica gel, PE: EtOAc= 5: 1, v/v).

^{1}H NMR (500 MHz, Chloroform-*d*) δ 8.2 (d, *J* = 31.0 Hz, 1H), 7.4 – 7.3 (m, 5H), 5.7 (ddt, *J* = 17.2, 10.4, 7.1 Hz, 1H), 5.2 – 5.1 (m, 2H), 3.7 (d, *J* = 38.9 Hz, 3H), 3.2 (ddt, *J* = 14.6, 6.9, 1.5 Hz, 1H), 3.0 (dd, *J* = 14.5, 7.3 Hz, 1H), 2.7 (d, *J* = 14.3 Hz, 3H).

^{13}C NMR (126 MHz, Chloroform-*d*) δ 171.1, 164.8, 163.9, 137.3, 133.5, 131.6, 128.8, 128.6, 128.5, 128.4, 128.4, 127.5, 120.8, 119.2, 71.3, 53.1, 52.6, 39.9, 37.7, 29.8.

HRMS (ESI) m/z: $[M+H]^+$ Calcd. for $C_{14}H_{18}NO_3^+$ 248.1281; Found: 248.1285.

N-(3-allyl-2-oxotetrahydrofuran-3-yl)-*N*-methylformamide (6e)

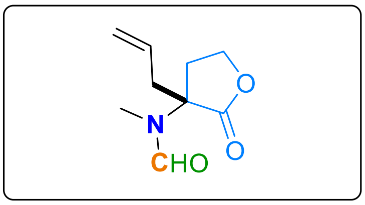

Following the **general procedure 5** on 0.2 mmol scale, yellow oil, yield: 74% (27 mg). R_f = 0.2 (silica gel, PE: EtOAc = 1:2, v/v), column chromatography (silica gel, PE: EtOAc= 1: 2, v/v).

^{1}H NMR (500 MHz, Chloroform-*d*) δ 8.2 (d, *J* = 179.7 Hz, 1H), 5.7 (dddd, *J* = 18.9, 15.1, 9.2, 7.6 Hz, 1H), 5.4 – 5.2 (m, 2H), 4.4 (ddd, *J* = 9.7, 8.8, 4.9 Hz, 0.7H), 4.3 (ddd, *J* = 7.2, 6.3, 4.5 Hz, 0.7H), 4.2 (td, *J* = 8.9, 6.7 Hz, 0.7H), 3.0 (d, *J* = 55.0 Hz, 3H), 2.8 – 2.7 (m, 1H), 2.6 – 2.5 (m, 1H), 2.5 (tdd, *J* = 13.6, 10.2, 7.0 Hz, 1H), 2.4 (ddd, *J* = 13.4, 9.0, 5.0 Hz, 1H).

^{13}C NMR (126 MHz, Chloroform-*d*) δ 175.5, 173.9, 163.4, 161.8, 130.1, 129.8, 121.8, 121.7, 121.7, 65.5, 65.0, 63.7, 61.8, 39.3, 38.9, 32.5, 32.4, 32.0, 27.8.

HRMS (ESI) m/z: $[M+H]^+$ Calcd. for $C_9H_{14}NO_3^+$ 184.0968; Found: 184.0967.

methyl 2-allyl-1-formylpyrrolidine-2-carboxylate (6f)

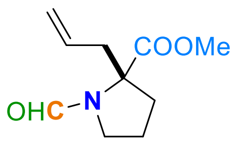

Following the **general procedure 5** on 0.2 mmol scale, yellow oil, yield: 63% (25 mg). R_f = 0.2 (silica gel, PE: EtOAc = 5:1, v/v), column chromatography (silica gel, PE: EtOAc= 5: 1, v/v).

^{1}H NMR (500 MHz, Chloroform-*d*) δ 8.3 – 8.1 (m, 1H), 5.7 – 5.5 (m, 1H), 5.2 – 5.0 (m, 2H), 3.7 (d, *J* = 17.9 Hz, 3H), 3.6 (td, *J* = 8.0, 4.1 Hz, 1H), 3.4 – 3.4 (m, 1H), 2.7 (ddt, *J* = 14.1, 7.9, 1.0 Hz, 1H), 2.7 – 2.6 (m, 1H), 2.3 (ddd, *J* = 12.9, 7.0, 4.5 Hz, 1H), 2.0 (tdd, *J* = 13.0, 8.4, 6.0 Hz, 1H), 1.9 – 1.7 (m, 2H).

^{13}C NMR (126 MHz, Chloroform-*d*) δ 173.5, 173.4, 161.8, 160.3, 132.7, 131.0, 120.8, 120.8, 119.6, 68.1, 66.6, 53.0, 52.5, 48.2, 45.5, 41.9, 37.3, 35.9, 35.5, 23.3, 21.7.

HRMS (ESI) m/z: $[M+H]^+$ Calcd. for $C_{10}H_{16}NO_3^+$ 198.1125; Found: 198.1126.

tert-butyl 2-allyl-1-formylpyrrolidine-2-carboxylate (6g)

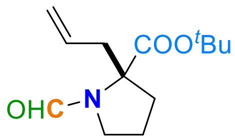

Following the **general procedure 5** on 0.2 mmol scale, yellow oil, yield: 81% (38 mg). R_f = 0.2 (silica gel, PE: EtOAc = 5:1, v/v), column chromatography (silica gel, PE: EtOAc= 5: 1, v/v).

^{1}H NMR (500 MHz, Chloroform-*d*) δ 8.3 – 8.2 (m, 1H), 5.7 (dddd, *J* = 16.9, 10.4, 7.9, 6.6 Hz, 1H), 5.2 – 5.0 (m, 2H), 3.6 (ddd, *J* = 12.2, 8.1, 4.4 Hz, 1H), 3.5 – 3.3 (m, 1H), 2.7 – 2.5 (m, 2H), 2.3 (ddd, *J* = 12.9, 7.0, 4.5 Hz, 1H), 2.0 (s, 1H), 1.9 (ddd, *J* = 12.8, 9.6, 7.0 Hz, 1H), 1.9 – 1.7 (m, 2H), 1.4 (d, *J* = 5.5 Hz, 9H).

^{13}C NMR (126 MHz, Chloroform-*d*) δ 172.0, 171.8, 161.9, 160.2, 133.2, 131.3, 120.4, 119.2, 82.6, 68.5, 48.2, 45.5, 42.0, 37.3, 35.9, 35.6, 27.8, 27.8, 23.4, 21.7.

HRMS (ESI) m/z: $[M+H]^+$ Calcd. for $C_{13}H_{21}NO_3^+$ 239.1521; Found: 239.1521.

methyl 1-formyl-2-(2-methylallyl)pyrrolidine-2-carboxylate (6h)

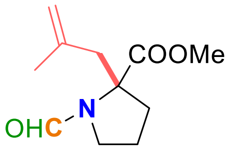

Following the **general procedure 5** on 0.2 mmol scale, yellow oil, yield: 49% (21 mg). R_f = 0.2 (silica gel, PE: EtOAc = 5:1, v/v), column chromatography (silica gel, PE: EtOAc= 5: 1, v/v).

^{1}H NMR (500 MHz, Chloroform-*d*) δ 8.7 – 8.5 (m, 1H), 5.3 – 5.2 (m, 1H), 5.2 – 5.1 (m, 1H), 4.1 (d, *J* = 20.8 Hz, 3H), 4.0 (ddd, *J* = 12.4, 8.0, 4.8 Hz, 1H), 3.8 (dtd, *J* = 11.7, 7.6, 1.1 Hz, 1H), 3.1 – 3.1 (m,

1H), 3.0 – 2.9 (m, 1H), 2.7 (ddd, J = 12.5, 6.9, 5.1 Hz, 1H), 2.4 (ddd, J = 13.0, 9.0, 7.0 Hz, 1H), 2.3 – 2.2 (m, 1H), 2.2 – 2.1 (m, 1H), 2.1 (dt, J = 23.9, 1.1 Hz, 3H).
^{13}C NMR (126 MHz, Chloroform-*d*) δ 173.8, 173.4, 162.2, 160.9, 141.2, 139.5, 117.4, 116.8, 68.1, 66.8, 52.9, 52.5, 48.1, 45.7, 45.3, 39.5, 36.4, 35.2, 24.0, 23.5, 23.2, 21.7.
HRMS (ESI) m/z: $[M+H]^+$ Calcd. for $C_{11}H_{17}NO_3^+$ 211.1208; Found: 211.1204.

methyl 1-formyl-2-(2-methylbut-3-en-2-yl)pyrrolidine-2-carboxylate (6i)

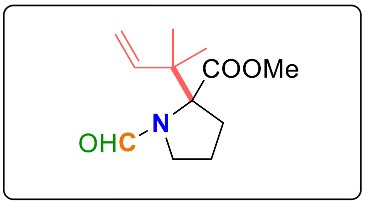

Following the **general procedure 5** on 0.2 mmol scale, yellow oil, yield: 36% (16 mg). R_f = 0.2 (silica gel, PE: EtOAc = 5:1, v/v), column chromatography (silica gel, PE: EtOAc= 5: 1, v/v).
^{1}H NMR (500 MHz, Chloroform-*d*) δ 8.5 (s, 1H), 5.8 (dd, J = 17.4, 10.9 Hz, 1H), 5.2 – 5.0 (m, 2H), 4.0 (ddd, J = 11.6, 8.3, 1.4 Hz, 1H), 3.8 (s, 3H), 3.1 (tdd, J = 11.3, 6.5, 1.2 Hz, 1H), 2.5 (ddt, J = 13.2, 6.7, 1.6 Hz, 1H), 2.1 (td, J = 12.9, 6.8 Hz, 1H), 1.9 – 1.8 (m, 1H), 1.6 – 1.5 (m, 1H), 1.2 (s, 3H), 1.1 (s, 3H).
^{13}C NMR (126 MHz, Chloroform-*d*) δ 173.0, 163.7, 143.4, 114.4, 76.8, 52.7, 46.3, 43.6, 35.1, 25.0, 22.3, 21.9.
HRMS (ESI) m/z: $[M+H]^+$ Calcd. for $C_{12}H_{20}NO_3^+$ 226.1438; Found: 226.1439.

methyl 2-benzyl-1-formylpyrrolidine-2-carboxylate (7a)

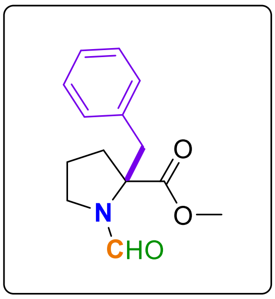

Following the **general procedure 5** on 0.2 mmol scale, yellow oil, yield: 56% (28 mg). R_f = 0.2 (silica gel, PE: EtOAc = 5:1, v/v), column chromatography (silica gel, PE: EtOAc= 5: 1, v/v).
^{1}H NMR (500 MHz, Chloroform-*d*) δ 8.2 (d, J = 98.6 Hz, 1H), 7.3 – 7.2 (m, 3H), 7.2 – 7.1 (m, 1H), 7.1 (dd, J = 7.9, 1.7 Hz, 1H), 3.8 (d, J = 14.0 Hz, 1H), 3.8 (d, J = 4.8 Hz, 3H), 3.6 – 3.5 (m, 1H), 3.4 – 3.3 (m, 1H), 3.3 (d, J = 13.7 Hz, 1H), 3.2 (d, J = 13.7 Hz, 1H), 2.3 (dt, J = 13.4, 6.9 Hz, 1H), 2.1 – 2.0 (m, 1H), 1.8 – 1.7 (m, 1H), 1.6 (ddd, J = 13.1, 7.5, 6.1 Hz, 1H).
^{13}C NMR (126 MHz, Chloroform-*d*) δ 173.7, 173.4, 162.3, 160.9, 136.4, 134.9, 130.9, 130.3, 128.7, 128.2, 127.5, 126.8, 69.3, 67.4, 53.0, 52.7, 48.1, 45.6, 43.7, 37.4, 36.5, 35.1, 23.0, 21.6.
HRMS (ESI) m/z: $[M+H]^+$ Calcd. for $C_{14}H_{18}NO_3^+$ 248.1281; Found: 248.1279.

methyl 2-([1,1'-biphenyl]-4-ylmethyl)-1-formylpyrrolidine-2-carboxylate (7b)

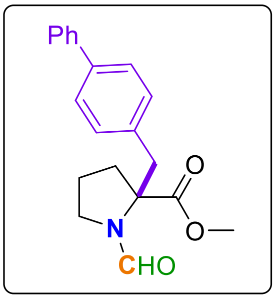

Following the **general procedure 5** on 0.2 mmol scale, yellow oil, yield: 53% (34 mg). R_f = 0.2 (silica gel, PE: EtOAc = 5:1, v/v), column chromatography (silica gel, PE: EtOAc= 5: 1, v/v).

^{1}H NMR (500 MHz, Chloroform-*d*) δ 8.3 (d, *J* = 72.2 Hz, 1H), 7.6 (ddd, *J* = 10.8, 7.4, 1.4 Hz, 2H), 7.5 (dd, *J* = 8.0, 5.1 Hz, 2H), 7.4 (t, *J* = 7.6 Hz, 2H), 7.4 – 7.3 (m, 1H), 7.3 – 7.2 (m, 1H), 7.2 – 7.2 (m, 1H), 3.9 (d, *J* = 13.9 Hz, 1H), 3.8 (d, *J* = 6.5 Hz, 3H), 3.7 – 3.5 (m, 1H), 3.4 (dt, *J* = 11.9, 7.1 Hz, 1H), 3.3 (d, *J* = 13.8 Hz, 1H), 2.3 (dt, *J* = 13.3, 6.7 Hz, 1H), 2.2 – 2.1 (m, 1H), 1.8 – 1.7 (m, 1H), 1.3 – 1.2 (m, 1H).

^{13}C NMR (126 MHz, Chloroform-*d*) δ 173.7, 173.4, 162.3, 160.9, 140.4, 140.3, 133.9, 131.3, 130.8, 128.8, 128.8, 127.4, 127.3, 127.3, 127.0, 126.9, 126.9, 69.3, 67.5, 53.0, 52.7, 48.1, 45.6, 43.3, 37.1, 36.5, 35.2, 23.0, 21.7.

HRMS (ESI) m/z: $[M+H]^+$ Calcd. for $C_{20}H_{22}NO_3^+$ 324.1596; Found: 324.1594.

methyl 1-formyl-2-(4-iodobenzyl)pyrrolidine-2-carboxylate (7c)

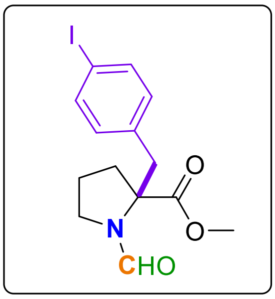

Following the **general procedure 5** on 0.2 mmol scale, yellow oil, yield: 51% (38 mg). R_f = 0.2 (silica gel, PE: EtOAc = 5:1, v/v), column chromatography (silica gel, PE: EtOAc= 5: 1, v/v).

^{1}H NMR (500 MHz, Chloroform-*d*) δ 8.4 – 8.1 (m, 1H), 7.6 (dd, *J* = 10.5, 8.2 Hz, 2H), 6.9 (dd, *J* = 23.4, 8.3 Hz, 2H), 3.8 (d, *J* = 5.0 Hz, 3H), 3.6 (ddd, *J* = 9.9, 7.1, 4.7 Hz, 1H), 3.4 (d, *J* = 11.8 Hz, 1H), 3.3 – 3.1 (m, 1H), 3.0 (d, *J* = 13.9 Hz, 1H), 2.1 – 2.0 (m, 1H), 1.8 – 1.7 (m, 1H), 1.6 – 1.6 (m, 1H), 1.3 – 1.2 (m, 1H).

^{13}C NMR (126 MHz, Chloroform-*d*) δ 173.4, 173.2, 162.1, 161.0, 137.8, 137.3, 136.0, 134.5, 132.9, 132.3, 93.2, 92.4, 69.0, 67.3, 53.1, 52.8, 48.1, 45.6, 43.1, 37.0, 36.5, 35.1, 23.0, 21.7.

HRMS (ESI) m/z: $[M+H]^+$ Calcd. for $C_{14}H_{17}INO_3^+$ 374.0248; Found: 374.0249.

methyl 2-(4-bromobenzyl)-1-formylpyrrolidine-2-carboxylate (7d)

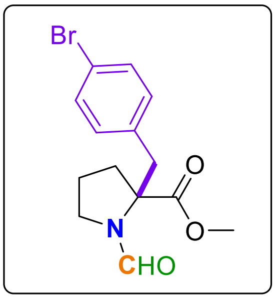

Following the **general procedure 5** on 0.2 mmol scale, yellow oil, yield: 49% (32 mg). R_f = 0.2 (silica gel, PE: EtOAc = 5:1, v/v), column chromatography (silica gel, PE: EtOAc= 5: 1, v/v).

^{1}H NMR (500 MHz, Chloroform-*d*) δ 8.2 (d, *J* = 96.8 Hz, 1H), 7.5 – 7.4 (m, 2H), 7.0 (dd, *J* = 22.4, 8.4 Hz, 2H), 3.8 (d, *J* = 5.3 Hz, 3H), 3.6 – 3.5 (m, 1H), 3.4 (d, *J* = 11.8 Hz, 1H), 3.3 – 3.1 (m, 1H), 3.0 (d, *J* = 13.9 Hz, 1H), 2.1 – 2.0 (m, 1H), 1.8 (dq, *J* = 12.8, 7.5 Hz, 1H), 1.6 (ddd, *J* = 13.1, 7.4, 6.0 Hz, 1H), 1.3 – 1.2 (m, 1H).

^{13}C NMR (126 MHz, Chloroform-*d*) δ 173.4, 173.2, 162.1, 161.0, 135.4, 133.9, 132.6, 132.0, 131.8, 131.4, 69.0, 67.2, 53.1, 52.8, 48.1, 45.6, 43.0, 36.9, 36.5, 35.1, 23.0, 21.7.

HRMS (ESI) m/z: $[M+H]^+$ Calcd. for $C_{14}H_{17}BrNO_3^+$ 326.0386; Found: 326.0384.

methyl 2-(4-bromo-3-fluorobenzyl)-1-formylpyrrolidine-2-carboxylate (7e)

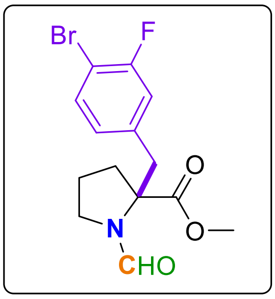

Following the **general procedure 5** on 0.2 mmol scale, yellow oil, yield: 56% (38 mg). R_f = 0.2 (silica gel, PE: EtOAc = 5:1, v/v), column chromatography (silica gel, PE: EtOAc= 5: 1, v/v).

^{1}H NMR (500 MHz, Chloroform-*d*) δ 8.2 (d, *J* = 106.2 Hz, 1H), 7.5 (dt, *J* = 11.9, 7.8 Hz, 1H), 6.9 (ddd, *J* = 14.3, 9.4, 2.0 Hz, 1H), 6.8 (ddd, *J* = 27.6, 8.2, 2.0 Hz, 1H), 3.8 (s, 1H), 3.8 (d, *J* = 8.6 Hz, 3H), 3.6 (ddd, *J* = 10.0, 7.3, 4.9 Hz, 1H), 3.1 (d, *J* = 14.0 Hz, 1H), 3.0 – 2.9 (m, 1H), 2.1 – 2.0 (m, 1H), 1.8 (dq, *J* = 12.8, 7.6 Hz, 1H), 1.7 (dq, *J* = 13.2, 6.9, 6.5 Hz, 1H), 1.3 (dd, *J* = 4.9, 1.9 Hz, 1H).

^{13}C NMR (126 MHz, Chloroform-*d*) δ 173.2, 172.9, 161.9, 161.1, 133.7, 133.1, 127.8, 127.8, 127.3, 118.9, 118.7, 118.5, 68.9, 67.2, 53.2, 52.8, 48.1, 45.6, 42.9, 37.0, 36.6, 35.1, 23.0, 21.7.

^{19}F NMR (471 MHz, Chloroform-*d*) δ -106.4, -107.6.

HRMS (ESI) m/z: $[M+H]^+$ Calcd. for $C_{14}H_{16}BrFNO_3^+$ 344.0292; Found: 344.0290.

methyl 1-formyl-2-(propa-1,2-dien-1-yl)pyrrolidine-2-carboxylate (9a)

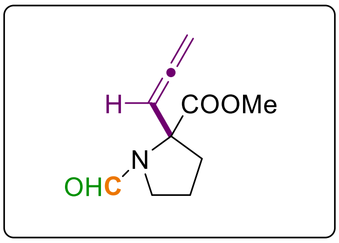

Following the **general procedure 6** on 0.2 mmol scale, yellow oil, yield: 82% (32 mg). R_f = 0.2 (silica gel, PE: EtOAc = 5:1, v/v), column chromatography (silica gel, PE: EtOAc= 5: 1, v/v).

^{1}H NMR (500 MHz, Chloroform-*d*) δ 8.3 – 8.1 (m, 1H), 5.5 (td, *J* = 6.7, 1.8 Hz, 1H), 5.0 – 4.9 (m, 2H), 3.7 (s, 3H), 3.7 – 3.5 (m, 2H), 2.5 (dtd, *J* = 12.9, 6.5, 2.0 Hz, 1H), 2.1 (ddddd, *J* = 14.5, 12.7, 7.9, 6.6, 2.0 Hz, 1H), 2.0 – 1.8 (m, 2H).

^{13}C NMR (126 MHz, Chloroform-*d*) δ 207.3, 206.8, 172.3, 171.9, 162.6, 159.9, 93.8, 91.3, 80.2, 79.1, 67.7, 65.8, 53.1, 52.9, 47.2, 44.9, 38.5, 37.0, 23.4, 21.7.

HRMS (ESI) m/z: $[M+H]^+$ Calcd. for $C_{10}H_{14}NO_3^+$ 196.0968; Found: 196.0966.

tert-butyl 1-formyl-2-(propa-1,2-dien-1-yl)pyrrolidine-2-carboxylate (9b)

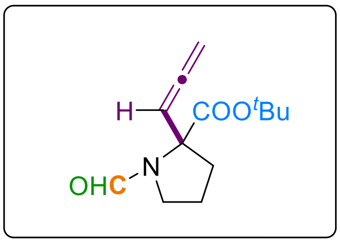

Following the **general procedure 6** on 0.2 mmol scale, yellow oil, yield: 88% (42 mg). R_f = 0.2 (silica gel, PE: EtOAc = 5:1, v/v), column chromatography (silica gel, PE: EtOAc= 5: 1, v/v).

^{1}H NMR (500 MHz, Chloroform-*d*) δ 8.3 – 8.1 (m, 1H), 5.5 (t, *J* = 6.6 Hz, 1H), 5.0 – 4.8 (m, 2H), 3.6 – 3.5 (m, 2H), 2.4 (dt, *J* = 12.8, 6.5 Hz, 1H), 2.1 (s, 1H), 1.9 – 1.8 (m, 1H), 1.8 – 1.7 (m, 1H), 1.4 (s, 10H).

^{13}C NMR (126 MHz, Chloroform-*d*) δ 207.4, 170.3, 162.7, 159.7, 94.1, 91.7, 83.0, 80.0, 67.8, 47.3, 44.9, 38.6, 37.2, 27.9, 27.8, 23.5, 21.7.

HRMS (ESI) m/z: $[M+H]^+$ Calcd. for $C_{13}H_{19}NO_3^+$ 237.1365; Found: 237.1369.

methyl 2-(buta-2,3-dien-2-yl)-1-formylpyrrolidine-2-carboxylate (9c)

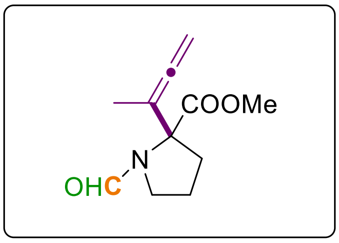

Following the **general procedure 6** on 0.2 mmol scale, yellow oil, yield: 84% (35 mg). R_f = 0.2 (silica gel, PE: EtOAc = 5:1, v/v), column chromatography (silica gel, PE: EtOAc= 5: 1, v/v).

^{1}H NMR (500 MHz, Chloroform-*d*) δ 8.3 (s, 1H), 4.8 (t, *J* = 3.0 Hz, 2H), 3.7 (s, 3H), 3.6 (td, *J* = 8.0, 3.8 Hz, 1H), 3.5 – 3.3 (m, 1H), 2.6 (ddd, *J* = 12.9, 6.7, 4.4 Hz, 1H), 2.1 – 2.1 (m, 1H), 1.9 – 1.8 (m, 1H), 1.8 – 1.7 (m, 1H), 1.7 (t, *J* = 3.1 Hz, 3H).

^{13}C NMR (126 MHz, Chloroform-*d*) δ 206.7, 171.8, 163.0, 98.9, 78.1, 71.7, 52.9, 44.8, 34.9, 22.0, 14.2.

HRMS (ESI) m/z: $[M+H]^+$ Calcd. for $C_{11}H_{16}NO_3^+$ 210.1125; Found: 210.1124.

methyl 1-formyl-2-(penta-1,2-dien-3-yl)pyrrolidine-2-carboxylate (9d)

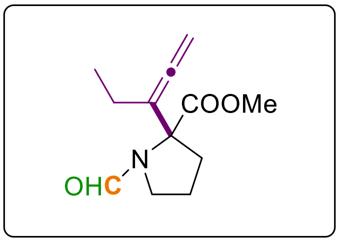

Following the **general procedure 6** on 0.2 mmol scale, yellow oil, yield: 86% (38 mg). R_f = 0.2 (silica gel, PE: EtOAc = 5:1, v/v), column chromatography (silica gel, PE: EtOAc= 5: 1, v/v).
^{1}H NMR (500 MHz, Chloroform-*d*) δ 8.3 (s, 1H), 4.9 (td, *J* = 3.9, 1.4 Hz, 2H), 3.7 (s, 3H), 3.6 (ddd, *J* = 11.8, 8.2, 3.9 Hz, 1H), 3.4 (dddd, *J* = 11.8, 8.6, 7.3, 1.2 Hz, 1H), 2.6 (ddd, *J* = 12.9, 6.6, 4.2 Hz, 1H), 2.1 (ddd, *J* = 12.9, 10.0, 6.9 Hz, 1H), 2.0 – 1.8 (m, 3H), 1.8 – 1.6 (m, 1H), 1.0 (t, *J* = 7.3 Hz, 3H).
^{13}C NMR (126 MHz, Chloroform-*d*) δ 206.1, 171.9, 163.0, 106.2, 80.7, 72.0, 52.9, 44.8, 35.1, 22.0, 19.7, 12.2.
HRMS (ESI) m/z: $[M+H]^+$ Calcd. for $C_{12}H_{18}NO_3^+$ 224.1281; Found: 224.1282.

methyl 2-(1-cyclopropylpropa-1,2-dien-1-yl)-1-formylpyrrolidine-2-carboxylate (9e)

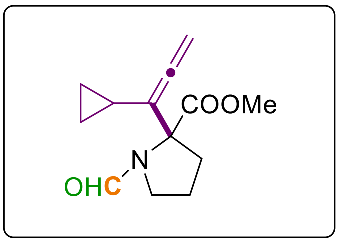

Following the **general procedure 6** on 0.2 mmol scale, yellow oil, yield: 82% (39 mg). R_f = 0.2 (silica gel, PE: EtOAc = 5:1, v/v), column chromatography (silica gel, PE: EtOAc= 5: 1, v/v).
^{1}H NMR (500 MHz, Chloroform-*d*) δ 8.3 (s, 1H), 4.9 (d, *J* = 2.2 Hz, 2H), 3.7 (s, 3H), 3.6 (ddd, *J* = 12.2, 8.2, 4.4 Hz, 1H), 3.5 – 3.4 (m, 1H), 2.7 – 2.5 (m, 1H), 2.4 – 2.3 (m, 1H), 1.9 – 1.8 (m, 1H), 1.8 – 1.7 (m, 1H), 1.0 (ddd, *J* = 8.0, 5.4, 2.7 Hz, 1H), 0.8 – 0.7 (m, 2H), 0.4 (dddd, *J* = 17.3, 9.4, 4.8, 2.3 Hz, 2H).
^{13}C NMR (126 MHz, Chloroform-*d*) δ 204.4, 171.9, 163.2, 108.2, 81.1, 71.9, 52.9, 44.9, 35.6, 22.0, 8.4, 8.0, 6.8.
HRMS (ESI) m/z: $[M+H]^+$ Calcd. for $C_{13}H_{18}NO_3^+$ 236.1281; Found: 236.1283.

methyl 1-formyl-2-(hepta-1,2-dien-3-yl)pyrrolidine-2-carboxylate (9f)

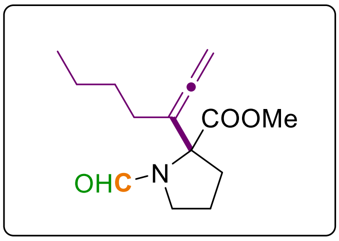

Following the **general procedure 6** on 0.2 mmol scale, yellow oil, yield: 82% (41 mg). R_f = 0.2 (silica gel, PE: EtOAc = 5:1, v/v), column chromatography (silica gel, PE: EtOAc= 5: 1, v/v).

^{1}H NMR (500 MHz, Chloroform-*d*) δ 8.3 (s, 1H), 5.0 – 4.8 (m, 2H), 3.7 (s, 3H), 3.6 (ddd, *J* = 12.0, 8.2, 3.9 Hz, 1H), 3.5 – 3.4 (m, 1H), 2.6 (ddd, *J* = 12.8, 6.6, 4.1 Hz, 1H), 2.2 – 2.1 (m, 1H), 1.9 (dddd, *J* = 11.0, 9.5, 4.7, 3.1 Hz, 2H), 1.8 – 1.7 (m, 1H), 1.7 – 1.6 (m, 1H), 1.4 – 1.3 (m, 2H), 1.3 – 1.2 (m, 2H), 0.8 (t, *J* = 7.2 Hz, 3H).
^{13}C NMR (126 MHz, Chloroform-*d*) δ 206.1, 171.9, 163.0, 104.5, 80.3, 72.0, 52.9, 52.9, 44.8, 35.0, 35.0, 29.8, 26.2, 22.3, 22.0, 13.9.
HRMS (ESI) m/z: $[M+H]^+$ Calcd. for $C_{14}H_{22}NO_3^+$ 252.1594; Found: 252.1597.

methyl 2-(4,4-dimethylpenta-1,2-dien-3-yl)-1-formylpyrrolidine-2-carboxylate (9g)

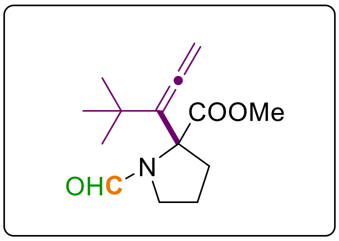

Following the **general procedure 6** on 0.2 mmol scale, yellow oil, yield: 82% (41 mg). R_f = 0.2 (silica gel, PE: EtOAc = 5:1, v/v), column chromatography (silica gel, PE: EtOAc= 5: 1, v/v).
^{1}H NMR (500 MHz, Chloroform-*d*) δ 8.4 (s, 1H), 5.0 – 4.8 (m, 2H), 3.7 (s, 3H), 3.6 (ddd, *J* = 11.8, 8.3, 3.6 Hz, 1H), 3.3 (dddd, *J* = 11.8, 8.7, 7.2, 1.3 Hz, 1H), 2.9 – 2.8 (m, 1H), 2.3 (ddd, *J* = 12.7, 10.2, 6.8 Hz, 1H), 1.9 (ddt, *J* = 13.8, 7.1, 3.4 Hz, 1H), 1.8 (ddt, *J* = 10.7, 6.4, 2.2 Hz, 1H), 1.1 (s, 9H).
^{13}C NMR (126 MHz, Chloroform-*d*) δ 207.5, 172.1, 163.4, 114.1, 80.2, 71.3, 52.9, 44.4, 36.6, 33.3, 31.2, 22.2.
HRMS (ESI) m/z: $[M+H]^+$ Calcd. for $C_{14}H_{22}NO_3^+$ 252.1594 Found: 252.1593.

methyl 1-formyl-2-(1-((tetrahydro-2H-pyran-2-yl)oxy)buta-2,3-dien-2-yl)pyrrolidine-2-carboxylate (9h)

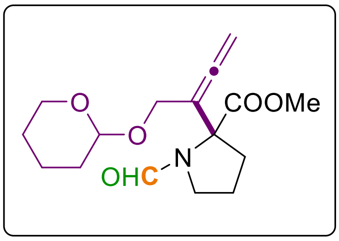

Following the **general procedure 6** on 0.2 mmol scale, yellow oil, yield: 77% (47 mg). R_f = 0.2 (silica gel, PE: EtOAc = 5:1, v/v), column chromatography (silica gel, PE: EtOAc= 5: 1, v/v).
^{1}H NMR (500 MHz, Chloroform-*d*) δ 8.3 (d, *J* = 3.8 Hz, 1H), 4.9 – 4.9 (m, 2H), 4.5 (d, *J* = 3.5 Hz, 1H), 3.8 – 3.7 (m, 2H), 3.7 (s, 3H), 3.6 (s, 1H), 3.5 – 3.4 (m, 3H), 2.6 – 2.5 (m, 1H), 2.3 – 2.2 (m, 1H), 2.1 (ddd, *J* = 13.0, 6.5, 3.7 Hz, 2H), 1.9 (dd, *J* = 8.0, 4.5 Hz, 1H), 1.7 (ddd, *J* = 10.0, 6.2, 3.1 Hz, 2H), 1.7 – 1.6 (m, 1H), 1.5 – 1.5 (m, 2H).
^{13}C NMR (126 MHz, Chloroform-*d*) δ 205.9, 171.7, 162.9, 101.4, 99.0, 98.9, 80.7, 80.6, 71.9, 71.9, 65.6, 65.6, 62.2, 62.2, 52.9, 44.8, 35.0, 35.0, 30.6, 30.5, 27.1, 27.1, 25.4, 21.9, 19.4.
HRMS (ESI) m/z: $[M+H]^+$ Calcd. for $C_{16}H_{24}NO_5^+$ 310.1649; Found: 310.1650.

tert-butyl 4-(1-(1-formyl-2-(methoxycarbonyl)pyrrolidin-2-yl)propa-1,2-dien-1-yl)piperidine-1-carboxylate (9i)

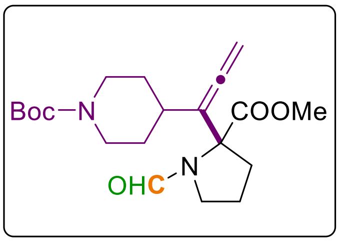

Following the **general procedure 6** on 0.2 mmol scale, yellow oil, yield: 62% (47 mg). R_f = 0.2 (silica gel, PE: EtOAc = 5:1, v/v), column chromatography (silica gel, PE: EtOAc= 5: 1, v/v).

^{1}H NMR (500 MHz, Chloroform-*d*) δ 8.2 (s, 1H), 4.9 (d, *J* = 2.2 Hz, 2H), 4.1 – 3.9 (m, 2H), 3.7 (s, 3H), 3.6 (ddd, *J* = 12.0, 8.3, 3.9 Hz, 1H), 3.4 – 3.3 (m, 1H), 2.7 – 2.5 (m, 3H), 2.0 (ddd, *J* = 12.8, 10.0, 6.8 Hz, 1H), 1.9 (ddt, *J* = 14.1, 7.2, 3.3 Hz, 1H), 1.7 – 1.7 (m, 2H), 1.6 (d, *J* = 13.3 Hz, 1H), 1.4 (s, 9H), 1.3 – 1.2 (m, 2H).

^{13}C NMR (126 MHz, Chloroform-*d*) δ 206.5, 171.7, 162.8, 154.6, 81.6, 79.5, 52.9, 44.8, 35.0, 33.3, 32.8, 28.4, 21.8.

HRMS (ESI) m/z: $[M+H]^+$ Calcd. for $C_{20}H_{30}N_2O_5^+$ 378.2155; Found: 378.2157.

methyl 1-formyl-2-(1-(2-hydroxypropoxy)buta-2,3-dien-2-yl)pyrrolidine-2-carboxylate (9j)

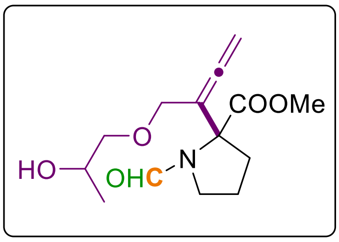

Following the **general procedure 6** on 0.2 mmol scale, yellow oil, yield: 62% (35 mg). R_f = 0.2 (silica gel, PE: EtOAc = 5:1, v/v), column chromatography (silica gel, PE: EtOAc= 5: 1, v/v).

^{1}H NMR (500 MHz, Chloroform-*d*) δ 8.3 (s, 1H), 5.0 (d, *J* = 2.0 Hz, 2H), 4.0 (dd, *J* = 5.4, 3.3 Hz, 2H), 3.9 – 3.8 (m, 1H), 3.7 (d, *J* = 1.2 Hz, 3H), 3.6 (dd, *J* = 7.1, 4.0 Hz, 1H), 3.5 – 3.4 (m, 1H), 3.3 (ddd, *J* = 10.1, 7.2, 3.1 Hz, 1H), 3.2 (ddd, *J* = 9.3, 7.8, 5.8 Hz, 1H), 2.7 (dd, *J* = 8.8, 3.4 Hz, 1H), 2.6 – 2.5 (m, 1H), 2.3 – 2.2 (m, 1H), 1.9 (dt, *J* = 12.8, 6.4 Hz, 1H), 1.7 (dt, *J* = 12.9, 7.4 Hz, 1H), 1.1 (dd, *J* = 6.4, 2.8 Hz, 3H).

^{13}C NMR (126 MHz, Chloroform-*d*) δ 207.6, 207.5, 171.9, 171.8, 163.6, 163.5, 101.9, 101.8, 79.7, 79.6, 76.2, 76.1, 69.6, 69.2, 69.1, 66.3, 66.3, 53.1, 53.1, 45.0, 36.4, 36.3, 21.9, 21.9, 18.8, 18.7.

HRMS (ESI) m/z: $[M+H]^+$ Calcd. for $C_{13}H_{20}NO_5^+$ 270.1336; Found: 270.1338.

methyl 1-formyl-2-(4-methylpenta-1,2,4-trien-3-yl)pyrrolidine-2-carboxylate (9k)

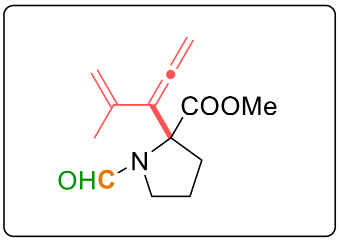

Following the **general procedure 6** on 0.2 mmol scale, yellow oil, yield: 83% (39 mg). R_f = 0.2 (silica gel, PE: EtOAc = 5:1, v/v), column chromatography (silica gel, PE: EtOAc= 5: 1, v/v).

^{1}H NMR (500 MHz, Chloroform-*d*) δ 8.4 (s, 1H), 5.2 – 5.1 (m, 2H), 5.0 (q, *J* = 1.5 Hz, 1H), 4.7 (s, 1H), 3.7 (s, 3H), 3.7 – 3.5 (m, 1H), 3.5 (dtd, *J* = 11.7, 7.5, 1.2 Hz, 1H), 2.8 (dt, *J* = 12.7, 6.2 Hz, 1H), 2.3 (dt, *J* = 13.0, 7.8 Hz, 1H), 1.9 – 1.9 (m, 2H), 1.9 (d, *J* = 1.2 Hz, 3H).

^{13}C NMR (126 MHz, Chloroform-*d*) δ 209.0, 171.6, 162.9, 136.2, 113.6, 109.8, 81.5, 70.0, 53.1, 44.8, 36.1, 23.6, 22.1.

HRMS (ESI) m/z: $[M+H]^+$ Calcd. for $C_{13}H_{18}NO_3^+$ 236.1281; Found: 236.1284.

methyl 2-(1-(cyclohex-1-en-1-yl)propa-1,2-dien-1-yl)-1-formylpyrrolidine-2-carboxylate (9l)

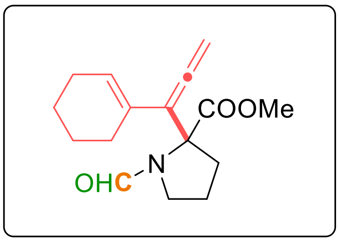

Following the **general procedure 6** on 0.2 mmol scale, yellow oil, yield: 85% (47 mg). R_f = 0.2 (silica gel, PE: EtOAc = 5:1, v/v), column chromatography (silica gel, PE: EtOAc= 5: 1, v/v).

^{1}H NMR (500 MHz, Chloroform-*d*) δ 8.3 (s, 1H), 5.5 (t, *J* = 3.9 Hz, 1H), 5.1 – 5.0 (m, 2H), 3.8 – 3.7 (m, 1H), 3.7 (d, *J* = 0.7 Hz, 3H), 3.5 (dd, *J* = 7.9, 4.7 Hz, 1H), 3.5 – 3.4 (m, 1H), 2.8 – 2.7 (m, 1H), 2.3 (ddd, *J* = 13.0, 9.0, 7.0 Hz, 1H), 1.9 – 1.8 (m, 3H), 1.6 (dd, *J* = 5.8, 2.2 Hz, 2H), 1.5 (dd, *J* = 8.7, 3.8 Hz, 2H).

^{13}C NMR (126 MHz, Chloroform-*d*) δ 208.0, 171.9, 163.1, 129.4, 126.0, 109.7, 81.0, 70.4, 53.0, 44.7, 36.2, 28.5, 25.9, 22.8, 22.0, 21.8.

HRMS (ESI) m/z: $[M+H]^+$ Calcd. for $C_{16}H_{21}NO_3^+$ 275.1521; Found: 275.1518.

methyl 1-formyl-2-(1-phenylpropa-1,2-dien-1-yl)pyrrolidine-2-carboxylate (9m)

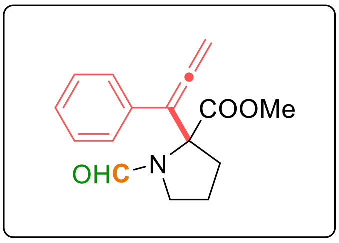

Following the **general procedure 6** on 0.2 mmol scale, yellow oil, yield: 83% (45 mg). R_f = 0.2 (silica gel, PE: EtOAc = 5:1, v/v), column chromatography (silica gel, PE: EtOAc= 5: 1, v/v).

^{1}H NMR (500 MHz, Chloroform-*d*) δ 8.4 (d, *J* = 1.3 Hz, 1H), 7.3 (dd, *J* = 7.1, 1.3 Hz, 2H), 7.3 (d, *J* = 7.2 Hz, 1H), 7.2 – 7.2 (m, 2H), 5.2 – 5.1 (m, 2H), 3.7 (s, 3H), 3.7 – 3.6 (m, 1H), 3.5 – 3.4 (m, 1H), 2.7 (ddd, *J* = 13.0, 6.8, 5.1 Hz, 1H), 2.2 (ddd, *J* = 13.0, 9.2, 7.0 Hz, 1H), 1.9 – 1.8 (m, 2H).

^{13}C NMR (126 MHz, Chloroform-*d*) δ 208.4, 171.8, 163.1, 133.1, 128.7, 128.0, 127.9, 107.0, 71.3, 53.2, 53.1, 44.9, 35.9, 22.0.

HRMS (ESI) m/z: $[M+H]^+$ Calcd. for $C_{16}H_{18}NO_3^+$ 272.1281; Found: 272.1280.

methyl 2-(1-(4-ethylphenyl)propa-1,2-dien-1-yl)-1-formylpyrrolidine-2-carboxylate (9n)

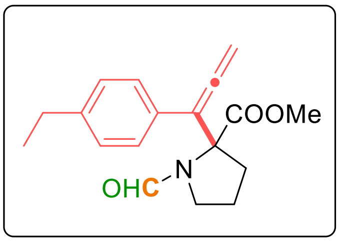

Following the **general procedure 6** on 0.2 mmol scale, yellow oil, yield: 82% (49 mg). R_f = 0.2 (silica gel, PE: EtOAc = 5:1, v/v), column chromatography (silica gel, PE: EtOAc= 5: 1, v/v).

^{1}H NMR (500 MHz, Chloroform-*d*) δ 8.4 (d, *J* = 1.3 Hz, 1H), 7.1 (d, *J* = 1.1 Hz, 4H), 5.2 – 5.0 (m, 2H), 3.7 (s, 3H), 3.7 – 3.6 (m, 1H), 3.5 – 3.5 (m, 1H), 2.7 (ddd, *J* = 13.0, 6.7, 5.0 Hz, 1H), 2.6 (q, *J* = 7.6 Hz, 2H), 2.2 (ddd, *J* = 13.0, 9.3, 7.0 Hz, 1H), 1.9 – 1.7 (m, 2H), 1.2 (t, *J* = 7.6 Hz, 3H).

^{13}C NMR (126 MHz, Chloroform-*d*) δ 208.4, 171.9, 163.1, 144.1, 130.2, 128.2, 127.9, 106.8, 79.9, 71.4, 53.1, 44.9, 35.8, 28.4, 22.0, 15.4.

HRMS (ESI) m/z: $[M+H]^+$ Calcd. for $C_{18}H_{22}NO_3^+$ 300.1594; Found: 300.1596.

methyl 1-formyl-2-(1-(4-methoxyphenyl)propa-1,2-dien-1-yl)pyrrolidine-2-carboxylate (9o)

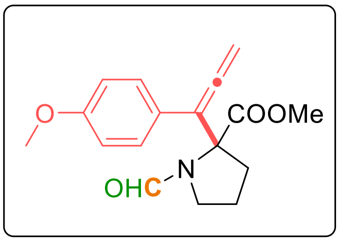

Following the **general procedure 6** on 0.2 mmol scale, yellow oil, yield: 81% (49 mg). R_f = 0.2 (silica gel, PE: EtOAc = 5:1, v/v), column chromatography (silica gel, PE: EtOAc= 5: 1, v/v).

^{1}H NMR (500 MHz, Chloroform-*d*) δ 8.4 (s, 1H), 7.2 – 7.1 (m, 2H), 6.9 – 6.8 (m, 2H), 5.2 – 5.1 (m, 2H), 3.8 (s, 3H), 3.7 (s, 3H), 3.6 (ddd, *J* = 12.5, 8.0, 4.7 Hz, 1H), 3.5 – 3.4 (m, 1H), 2.7 (ddd, *J* = 12.2, 6.8, 5.0 Hz, 1H), 2.2 (ddd, *J* = 12.8, 9.2, 7.0 Hz, 1H), 2.0 – 1.7 (m, 3H).

^{13}C NMR (126 MHz, Chloroform-*d*) δ 208.3, 171.9, 163.1, 159.2, 129.2, 125.1, 114.2, 106.4, 79.8, 71.5, 55.3, 53.1, 44.9, 35.8, 22.0.

HRMS (ESI) m/z: $[M+H]^+$ Calcd. for $C_{17}H_{19}NO_4^+$ 301.1314; Found: 301.1316.

methyl 2-(1-(4-chlorophenyl)propa-1,2-dien-1-yl)-1-formylpyrrolidine-2-carboxylate (9p)

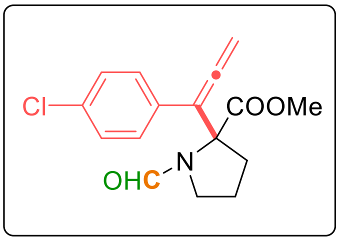

Following the **general procedure 6** on 0.2 mmol scale, white solid, m.p. 161-163 °C, yield: 83% (51 mg). R_f = 0.2 (silica gel, PE: EtOAc = 5:1, v/v), column chromatography (silica gel, PE: EtOAc= 5: 1, v/v).

^{1}H NMR (500 MHz, Chloroform-*d*) δ 8.4 (s, 1H), 7.3 – 7.2 (m, 2H), 7.1 (d, *J* = 8.6 Hz, 2H), 5.2 – 5.1 (m, 2H), 3.7 (s, 3H), 3.6 (ddd, *J* = 12.5, 8.1, 4.9 Hz, 1H), 3.5 (dtd, *J* = 11.8, 7.7, 1.1 Hz, 1H), 2.7 – 2.7 (m, 1H), 2.2 (ddd, *J* = 13.1, 9.1, 7.0 Hz, 1H), 1.9 – 1.7 (m, 2H).

^{13}C NMR (126 MHz, Chloroform-*d*) δ 208.4, 171.6, 162.9, 133.8, 131.7, 129.2, 128.9, 106.3, 80.6, 71.1, 53.2, 53.2, 44.9, 35.9, 21.9.

HRMS (ESI) m/z: $[M+H]^+$ Calcd. for $C_{16}H_{17}ClNO_3^+$ 306.0891; Found: 306.0888.

methyl 2-(1-(4-bromophenyl)propa-1,2-dien-1-yl)-1-formylpyrrolidine-2-carboxylate (9q)

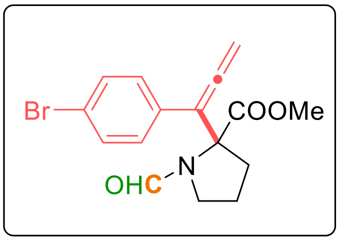

Following the **general procedure 6** on 0.2 mmol scale, white solid, m.p. 159-161 °C, yield: 79% (55 mg). R_f = 0.2 (silica gel, PE: EtOAc = 5:1, v/v), column chromatography (silica gel, PE: EtOAc= 5: 1, v/v).

^{1}H NMR (500 MHz, Chloroform-*d*) δ 8.4 (s, 1H), 7.3 – 7.2 (m, 3H), 7.1 (d, *J* = 8.5 Hz, 2H), 5.2 – 5.1 (m, 2H), 3.7 (s, 3H), 3.7 – 3.6 (m, 1H), 3.5 – 3.4 (m, 1H), 2.7 (ddd, *J* = 12.4, 6.7, 5.1 Hz, 1H), 2.2 (ddd, *J* = 13.0, 9.1, 7.0 Hz, 1H), 1.9 – 1.7 (m, 2H).

^{13}C NMR (126 MHz, Chloroform-*d*) δ 208.4, 171.6, 162.9, 133.8, 131.7, 129.2, 128.9, 106.3, 80.6, 71.1, 53.2, 44.9, 35.9, 22.0.

HRMS (ESI) m/z: $[M+H]^+$ Calcd. for $C_{16}H_{17}BrNO_3^+$ 350.0386; Found: 350.0389.

methyl 1-formyl-2-(1-(4-formylphenyl)propa-1,2-dien-1-yl)pyrrolidine-2-carboxylate (9r)

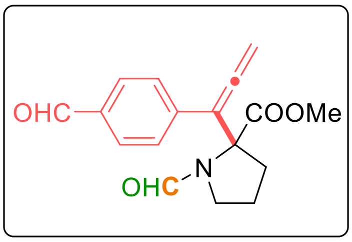

Following the **general procedure 6** on 0.2 mmol scale, yellow oil, yield: 73% (44 mg). R_f = 0.2 (silica gel, PE: EtOAc = 5:1, v/v), column chromatography (silica gel, PE: EtOAc= 5: 1, v/v).

^{1}H NMR (500 MHz, Chloroform-*d*) δ 10.0 (s, 1H), 8.4 (s, 1H), 7.8 (d, *J* = 8.4 Hz, 2H), 7.4 (d, *J* = 8.3 Hz, 2H), 5.4 – 5.2 (m, 2H), 3.7 (s, 3H), 3.7 – 3.6 (m, 1H), 3.6 – 3.5 (m, 1H), 2.8 (ddd, *J* = 12.6, 6.6, 5.4 Hz, 1H), 2.2 (ddd, *J* = 13.0, 8.8, 7.1 Hz, 1H), 1.9 – 1.8 (m, 2H).

^{13}C NMR (126 MHz, Chloroform-*d*) δ 209.1, 191.5, 171.4, 162.7, 139.6, 135.5, 130.0, 128.2, 107.2, 81.5, 70.7, 53.3, 45.0, 36.2, 22.0.

HRMS (ESI) m/z: $[M+H]^+$ Calcd. for $C_{17}H_{18}NO_4^+$ 300.1230; Found: 300.1235.

(Z)-hex-3-en-1-yl 2-(*N*-methylformamido)pent-4-enoate (11a)

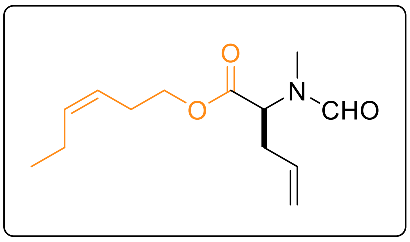

Following the **general procedure 7** on 0.2 mmol scale, yellow oil, yield: 52% (25 mg). $R_f = 0.2$ (silica gel, PE: EtOAc = 5:1, v/v), column chromatography (silica gel, PE: EtOAc= 5: 1, v/v).

^{1}H NMR (500 MHz, Chloroform-*d*) δ 8.1 (d, *J* = 18.9 Hz, 1H), 5.7 – 5.6 (m, 1H), 5.6 – 5.4 (m, 1H), 5.3 – 5.2 (m, 1H), 5.2 – 5.0 (m, 2H), 4.1 (dt, *J* = 14.4, 7.2 Hz, 3H), 2.9 (d, *J* = 46.4 Hz, 3H), 2.8 – 2.7 (m, 1H), 2.5 (ddd, *J* = 15.6, 9.0, 6.4 Hz, 1H), 2.4 (dd, *J* = 7.3, 3.8 Hz, 2H), 2.0 (t, *J* = 7.5 Hz, 2H), 1.0 (t, *J* = 7.5 Hz, 3H).

^{13}C NMR (126 MHz, Chloroform-*d*) δ 170.2, 170.0, 163.5, 163.3, 135.0, 134.8, 133.2, 132.3, 123.3, 123.1, 119.4, 118.1, 65.2, 64.9, 60.6, 53.5, 33.0, 32.4, 31.3, 27.1, 26.6, 26.6, 20.6, 14.2.

HRMS (ESI) m/z: $[M+H]^+$ Calcd. for $C_{13}H_{22}NO_3^+$ 240.1594; Found: 240.1592.

(S)-3,7-dimethyloct-6-en-1-yl 2-(*N*-methylformamido)pent-4-enoate (11b)

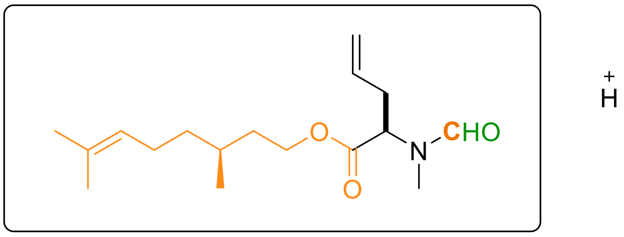

Following the **general procedure 7** on 0.2 mmol scale, yellow oil, yield: 65% (38 mg). $R_f = 0.2$ (silica gel, PE: EtOAc = 5:1, v/v), column chromatography (silica gel, PE: EtOAc= 5: 1, v/v).

^{1}H NMR (500 MHz, Chloroform-*d*) δ 8.1 (d, *J* = 16.4 Hz, 1H), 5.7 – 5.6 (m, 1H), 5.2 – 5.0 (m, 3H), 4.2 – 4.1 (m, 3H), 2.9 (d, *J* = 47.3 Hz, 3H), 2.8 – 2.7 (m, 1H), 2.6 – 2.4 (m, 1H), 1.9 (tp, *J* = 14.7, 7.5 Hz, 2H), 1.7 – 1.6 (m, 4H), 1.6 (d, *J* = 1.3 Hz, 3H), 1.6 – 1.4 (m, 2H), 1.4 – 1.2 (m, 2H), 1.2 (dddd, *J* = 13.0, 8.1, 5.6, 2.2 Hz, 1H), 0.9 (dd, *J* = 6.5, 2.0 Hz, 3H).

^{13}C NMR (126 MHz, Chloroform-*d*) δ 170.2, 170.0, 163.4, 163.3, 133.2, 132.3, 131.5, 131.4, 124.4, 124.3, 119.3, 118.1, 64.3, 64.0, 60.6, 53.5, 36.9, 36.9, 35.3, 35.3, 33.0, 32.4, 31.3, 29.4, 27.0, 25.7, 25.3, 25.3, 19.3, 17.7.

HRMS (ESI) m/z: $[M+H]^+$ Calcd. for $C_{17}H_{30}NO_3^+$ 296.2220; Found: 296.2217.

(E)-3,7-dimethylocta-2,6-dien-1-yl 2-(*N*-methylformamido)pent-4-enoate (11c)

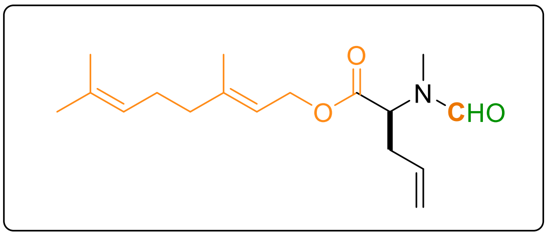

Following the **general procedure 7** on 0.2 mmol scale, yellow oil, yield: 46% (25 mg). $R_f = 0.2$ (silica gel, PE: EtOAc = 5:1, v/v), column chromatography (silica gel, PE: EtOAc= 5: 1, v/v).

^{1}H NMR (500 MHz, Chloroform-*d*) δ 8.1 (d, *J* = 17.8 Hz, 1H), 5.7 – 5.6 (m, 1H), 5.3 (t, *J* = 7.3 Hz, 1H), 5.2 – 5.0 (m, 3H), 4.7 – 4.6 (m, 2H), 4.1 (dd, *J* = 10.4, 5.2 Hz, 1H), 2.8 (d, *J* = 47.3 Hz, 3H), 2.8 – 2.7 (m, 1H), 2.6 – 2.4 (m, 1H), 2.1 (s, 2H), 2.0 (t, *J* = 4.0 Hz, 2H), 1.7 – 1.6 (m, 6H), 1.6 (s, 3H).
^{13}C NMR (126 MHz, Chloroform-*d*) δ 170.1, 169.9, 163.5, 163.3, 143.6, 143.0, 133.3, 132.3, 132.0, 131.9, 123.6, 123.5, 119.3, 118.1, 117.8, 117.4, 62.5, 62.3, 60.7, 53.4, 39.5, 33.1, 32.4, 31.2, 27.0, 26.2, 26.2, 25.7, 17.7, 16.5, 16.5.
HRMS (ESI) m/z: [M+H]$^{+}$ Calcd. for $C_{17}H_{27}NO_3^{+}$ 293.1991; Found: 293.1988.

1,3,3-trimethylbicyclo[2.2.1]heptan-2-yl 2-(*N*-methylformamido)pent-4-enoate (11d)

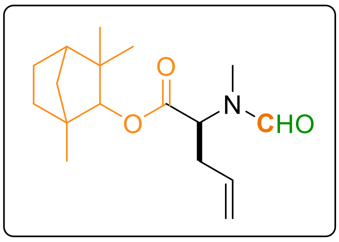

Following the **general procedure 7** on 0.2 mmol scale, yellow oil, yield: 67% (39 mg). R_f = 0.2 (silica gel, PE: EtOAc = 5:1, v/v), column chromatography (silica gel, PE: EtOAc= 5: 1, v/v).
^{1}H NMR (500 MHz, Chloroform-*d*) δ 8.1 – 8.1 (m, 1H), 5.7 – 5.6 (m, 1H), 5.2 – 5.1 (m, 2H), 4.4 – 4.3 (m, 1H), 4.2 – 4.1 (m, 1H), 2.9 (dd, *J* = 43.4, 15.5 Hz, 3H), 2.8 – 2.7 (m, 1H), 2.5 (dddd, *J* = 18.6, 15.0, 10.8, 7.7 Hz, 1H), 1.7 – 1.7 (m, 1H), 1.6 (qt, *J* = 10.7, 8.6, 4.1 Hz, 2H), 1.6 – 1.5 (m, 1H), 1.4 (ddt, *J* = 13.9, 7.0, 3.7 Hz, 1H), 1.2 (s, 1H), 1.1 (t, *J* = 4.0 Hz, 4H), 1.0 (dd, *J* = 3.0, 1.4 Hz, 3H), 0.7 (dd, *J* = 9.7, 4.1 Hz, 3H).
^{13}C NMR (126 MHz, Chloroform-*d*) δ 170.6, 170.3, 163.4, 163.4, 163.3, 163.3, 133.3, 133.3, 132.4, 132.3, 119.3, 118.0, 118.0, 88.1, 88.0, 87.7, 87.6, 60.8, 60.7, 53.5, 53.4, 48.4, 48.3, 48.3, 48.2, 48.2, 41.3, 41.2, 41.2, 39.5, 33.1, 32.9, 32.6, 32.3, 31.2, 29.6, 29.6, 27.3, 27.2, 26.6, 26.6, 26.6, 25.7, 25.7, 20.4, 20.3, 19.4, 19.4.
HRMS (ESI) m/z: [M+H]$^{+}$ Calcd. for $C_{17}H_{28}NO_3^{+}$ 294.2064; Found: 294.2061.

(1S,4S)-1,7,7-trimethylbicyclo[2.2.1]heptan-2-yl 2-(*N*-methylformamido)pent-4-enoate (11e)

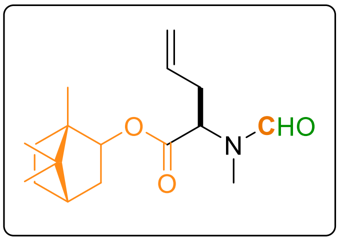

Following the **general procedure 7** on 0.2 mmol scale, yellow oil, yield: 56% (32 mg). R_f = 0.2 (silica gel, PE: EtOAc = 5:1, v/v), column chromatography (silica gel, PE: EtOAc= 5: 1, v/v).
^{1}H NMR (500 MHz, Chloroform-*d*) δ 8.1 – 8.1 (m, 1H), 5.7 (dddd, *J* = 24.2, 17.2, 10.3, 4.8 Hz, 1H), 5.2 – 5.1 (m, 2H), 4.9 – 4.8 (m, 1H), 4.1 (dd, *J* = 10.3, 5.3 Hz, 1H), 2.9 (dd, *J* = 48.6, 2.6 Hz, 3H), 2.8 – 2.7 (m, 1H), 2.5 (dq, *J* = 15.5, 7.5, 6.3 Hz, 1H), 2.4 (qd, *J* = 9.1, 8.6, 4.5 Hz, 1H), 1.8 – 1.8 (m, 1H), 1.8 – 1.6 (m, 2H), 1.3 – 1.2 (m, 1H), 1.2 – 1.1 (m, 1H), 1.0 – 0.9 (m, 1H), 0.9 (d, *J* = 3.3 Hz, 3H), 0.9 – 0.8 (m, 6H).

^{13}C NMR (126 MHz, Chloroform-*d*) δ 170.4, 170.2, 163.4, 163.4, 163.4, 133.3, 132.4, 119.3, 118.1, 81.8, 81.4, 81.3, 60.8, 60.8, 53.7, 48.9, 48.8, 47.9, 47.9, 47.8, 44.8, 44.8, 36.8, 36.6, 33.1, 33.0, 32.6, 32.5, 31.4, 28.0, 28.0, 28.0, 27.2, 27.1, 27.1, 19.7, 18.8, 18.8, 13.5.
HRMS (ESI) m/z: $[M+H]^+$ Calcd. for $C_{17}H_{28}NO_3^+$ 294.2064; Found: 294.2065.

(1R,2S,5R)-2-isopropyl-5-methylcyclohexyl 2-(*N*-methylformamido)pent-4-enoate (11f)

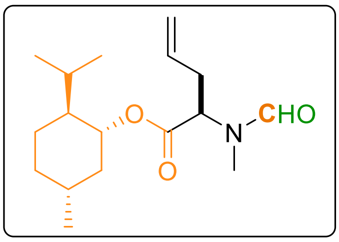

Following the **general procedure 7** on 0.2 mmol scale, yellow oil, yield: 71% (42 mg). R_f = 0.2 (silica gel, PE: EtOAc = 5:1, v/v), column chromatography (silica gel, PE: EtOAc= 5: 1, v/v).
^{1}H NMR (500 MHz, Chloroform-*d*) δ 8.1 – 8.0 (m, 1H), 5.7 (dddd, *J* = 28.1, 17.2, 9.0, 6.1 Hz, 1H), 5.2 – 5.0 (m, 2H), 4.7 (ddd, *J* = 10.7, 6.8, 4.2 Hz, 1H), 4.1 (dd, *J* = 10.3, 5.4 Hz, 1H), 2.8 (dd, *J* = 48.6, 3.2 Hz, 3H), 2.7 (qd, *J* = 14.0, 8.2 Hz, 1H), 2.6 – 2.4 (m, 1H), 2.0 – 1.9 (m, 1H), 1.8 – 1.7 (m, 1H), 1.7 – 1.6 (m, 2H), 1.5 (dq, *J* = 6.2, 2.9 Hz, 1H), 1.4 – 1.3 (m, 1H), 1.0 (ddd, *J* = 26.9, 12.6, 3.5 Hz, 2H), 0.9 (td, *J* = 7.3, 6.7, 3.3 Hz, 7H), 0.8 – 0.7 (m, 3H).
^{13}C NMR (126 MHz, Chloroform-*d*) δ 169.8, 169.7, 169.5, 169.4, 163.4, 163.4, 133.3, 132.4, 132.3, 119.3, 119.3, 118.0, 76.1, 76.0, 75.6, 75.6, 60.8, 60.8, 53.6, 53.4, 46.8, 46.8, 46.8, 40.7, 40.7, 40.7, 40.6, 34.1, 34.0, 33.0, 33.0, 32.5, 32.4, 31.4, 31.2, 31.2, 27.1, 27.0, 26.4, 26.1, 26.1, 23.3, 23.2, 23.1, 22.0, 21.9, 20.8, 20.7, 20.7, 16.2, 16.1, 16.1, 16.0.
HRMS (ESI) m/z: $[M+H]^+$ Calcd. for $C_{17}H_{30}NO_3^+$ 296.2220; Found: 296.2217.

(3aR,5R,6S,6aR)-5-(2,2-dimethyl-1,3-dioxolan-4-yl)-2,2-dimethyltetrahydrofuro[2,3-d][1,3]dioxol-6-yl -2-(*N*-methylformamido)pent-4-enoate (11g)

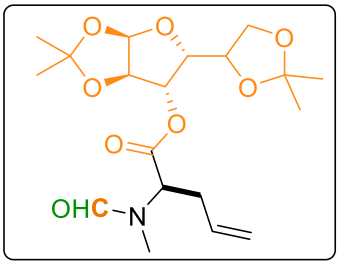

Following the **general procedure 7** on 0.2 mmol scale, yellow oil, yield: 56% (45 mg). R_f = 0.2 (silica gel, PE: EtOAc = 5:1, v/v), column chromatography (silica gel, PE: EtOAc= 5: 1, v/v).
^{1}H NMR (500 MHz, Chloroform-*d*) δ 8.1 (t, *J* = 4.8 Hz, 1H), 5.8 (dd, *J* = 3.8, 1.5 Hz, 1H), 5.8 – 5.6 (m, 1H), 5.3 (ddd, *J* = 22.5, 11.2, 3.0 Hz, 1H), 5.2 – 5.1 (m, 2H), 4.5 – 4.4 (m, 1H), 4.2 – 4.0 (m, 4H), 4.0 (ddt, *J* = 6.9, 5.2, 3.5 Hz, 1H), 3.0 – 2.8 (m, 3H), 2.8 – 2.7 (m, 1H), 2.6 – 2.4 (m, 1H), 1.5 (d, *J* = 3.4 Hz, 3H), 1.4 – 1.4 (m, 3H), 1.3 (q, *J* = 4.0, 3.4 Hz, 6H).
^{13}C NMR (126 MHz, Chloroform-*d*) δ 169.0, 168.9, 163.4, 163.1, 132.9, 132.8, 131.9, 119.7, 118.5, 118.4, 112.5, 112.4, 112.4, 109.4, 109.4, 105.2, 105.1, 105.1, 83.3, 83.2, 83.1, 80.0, 79.9, 79.9, 72.4,

72.4, 72.3, 72.2, 67.7, 67.7, 67.6, 60.5, 54.1, 53.4, 32.7, 32.4, 32.1, 31.9, 31.2, 26.9, 26.9, 26.8, 26.7, 26.7, 26.7, 26.2, 26.2, 25.2, 25.2, 25.1.
HRMS (ESI) m/z: $[M+H]^+$ Calcd. for $C_{19}H_{30}NO_8^+$ 400.1966; Found: 400.1968.

N-methyl-*N*-(5-oxooctan-4-yl)formamide (13)

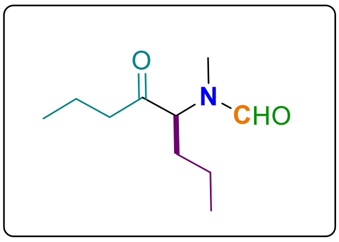

Following the **general procedure 12** on 0.2 mmol scale, yellow oil, yield: 89% (33 mg). R_f = 0.2 (silica gel, PE: EtOAc = 5:1, v/v), column chromatography (silica gel, PE: EtOAc= 5: 1, v/v).
^{1}H NMR (500 MHz, Chloroform-*d*) δ 8.1 (d, *J* = 15.7 Hz, 1H), 5.0 (dd, *J* = 10.2, 5.2 Hz, 1H), 2.8 (d, *J* = 50.6 Hz, 3H), 2.4 – 2.4 (m, 2H), 1.8 – 1.8 (m, 1H), 1.6 (dtd, *J* = 14.7, 7.0, 1.5 Hz, 2H), 1.4 – 1.1 (m, 3H), 1.0 – 0.9 (m, 6H).
^{13}C NMR (126 MHz, Chloroform-*d*) δ 207.9, 207.5, 163.4, 163.4, 65.8, 59.0, 42.2, 41.7, 31.0, 28.8, 28.1, 27.1, 19.2, 19.0, 16.9, 16.9, 13.7, 13.6, 13.5.
HRMS (ESI) m/z: $[M+H]^+$ Calcd. for $C_{10}H_{19}NO_2^+$ 185.1416; Found: 185.1412.

1-cyclopropyl-2-(methylamino)pent-4-en-1-one (14)

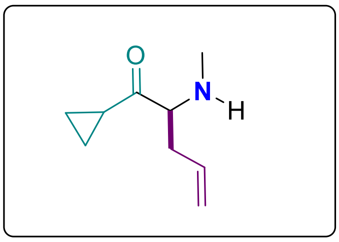

Following the **general procedure 13** on 0.2 mmol scale, yellow oil, yield: 89% (29 mg). R_f = 0.2 (silica gel, PE: EtOAc = 5:1, v/v), column chromatography (silica gel, PE: EtOAc= 5: 1, v/v).
^{1}H NMR (500 MHz, Chloroform-*d*) δ 5.8 – 5.6 (m, 1H), 5.2 – 4.9 (m, 2H), 3.4 (dd, *J* = 7.0, 5.4 Hz, 1H), 2.5 – 2.4 (m, 1H), 2.4 – 2.4 (m, 1H), 2.3 (s, 3H), 2.1 – 2.1 (m, 1H), 1.1 – 1.0 (m, 2H), 1.0 – 0.9 (m, 2H).
^{13}C NMR (126 MHz, Chloroform-*d*) δ 212.7, 133.7, 118.1, 69.5, 36.8, 34.8, 18.4, 11.2, 11.1.
HRMS (ESI) m/z: $[M+H]^+$ Calcd. for $C_9H_{15}NO^+$ 153.1154; Found: 153.1157.

2.4 NMR spectroscopic data

ethyl *N*-allyl-*N*-methylglycinate

^{1}H NMR (500 MHz, Chloroform-*d*)

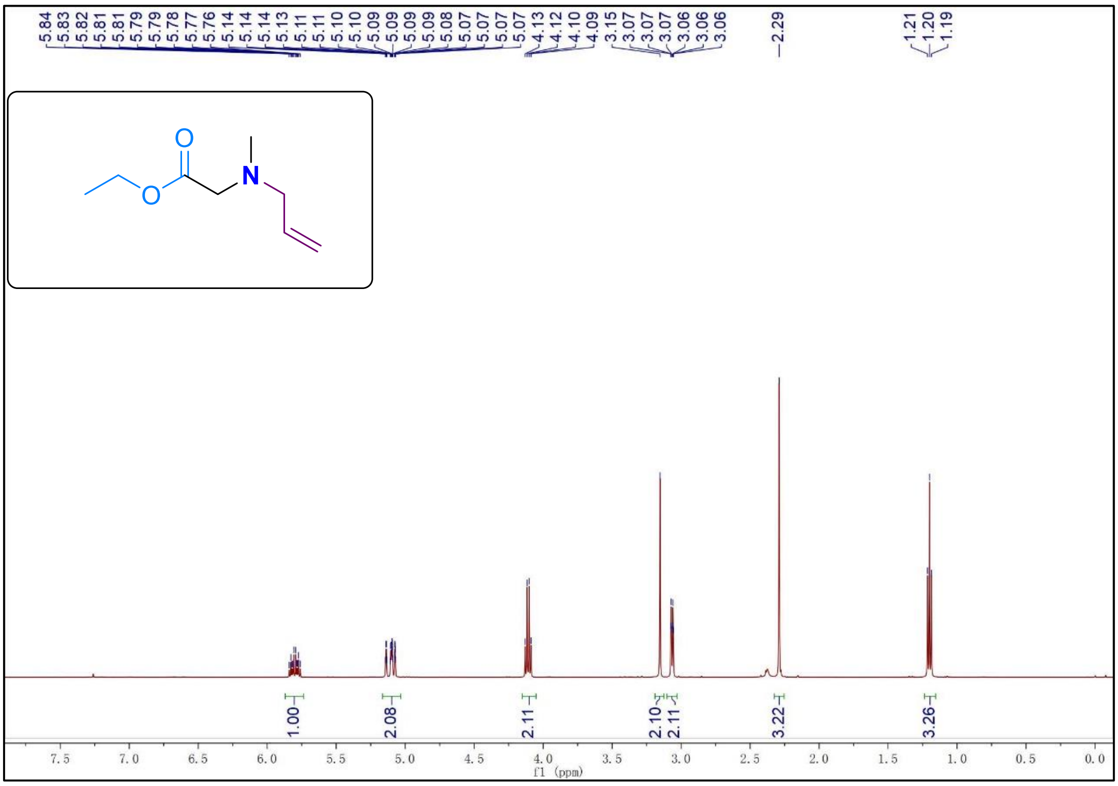

^{13}C NMR (126 MHz, Chloroform-*d*)

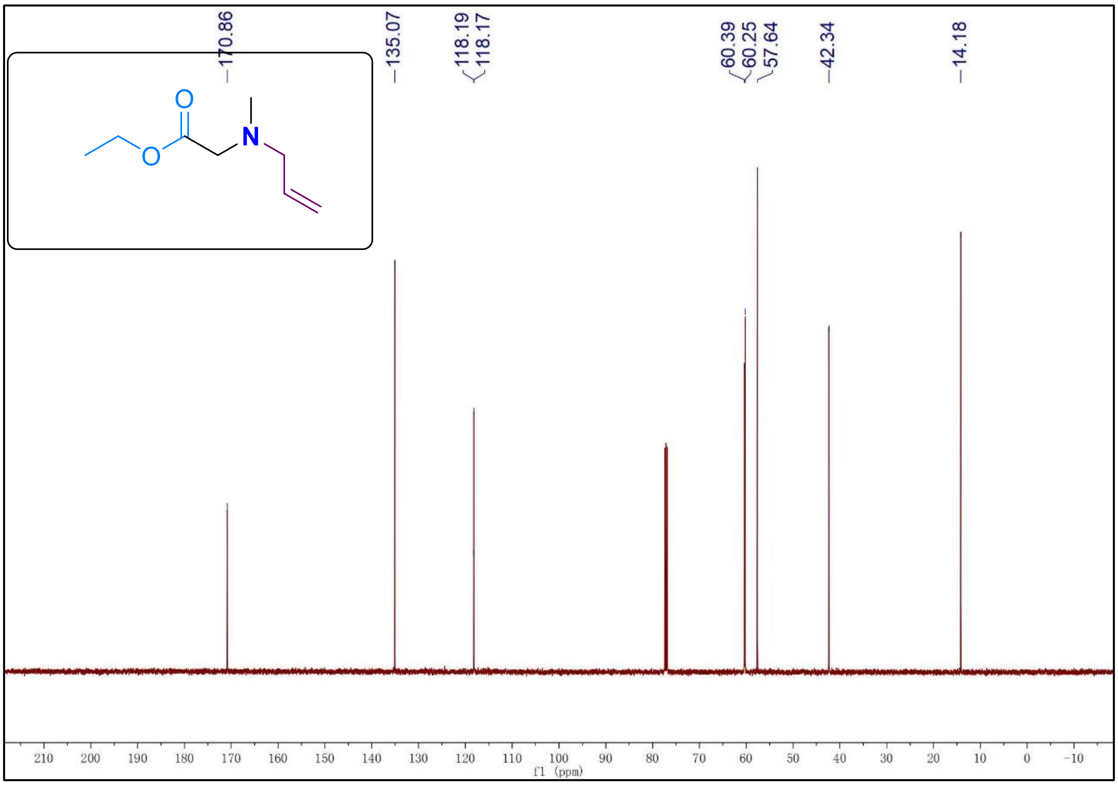

3-methylbut-3-en-1-yl *N*-allyl-*N*-methylglycinate

^{1}H NMR (500 MHz, Chloroform-*d*)

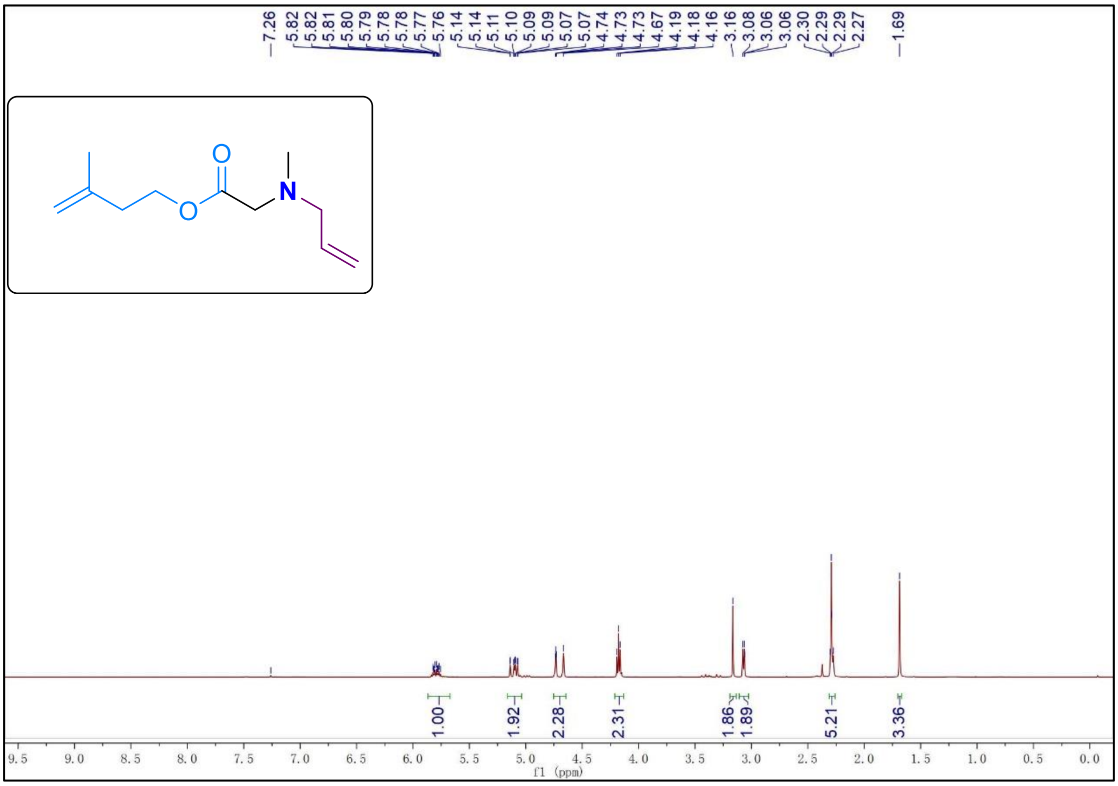

^{13}C NMR (126 MHz, Chloroform-*d*)

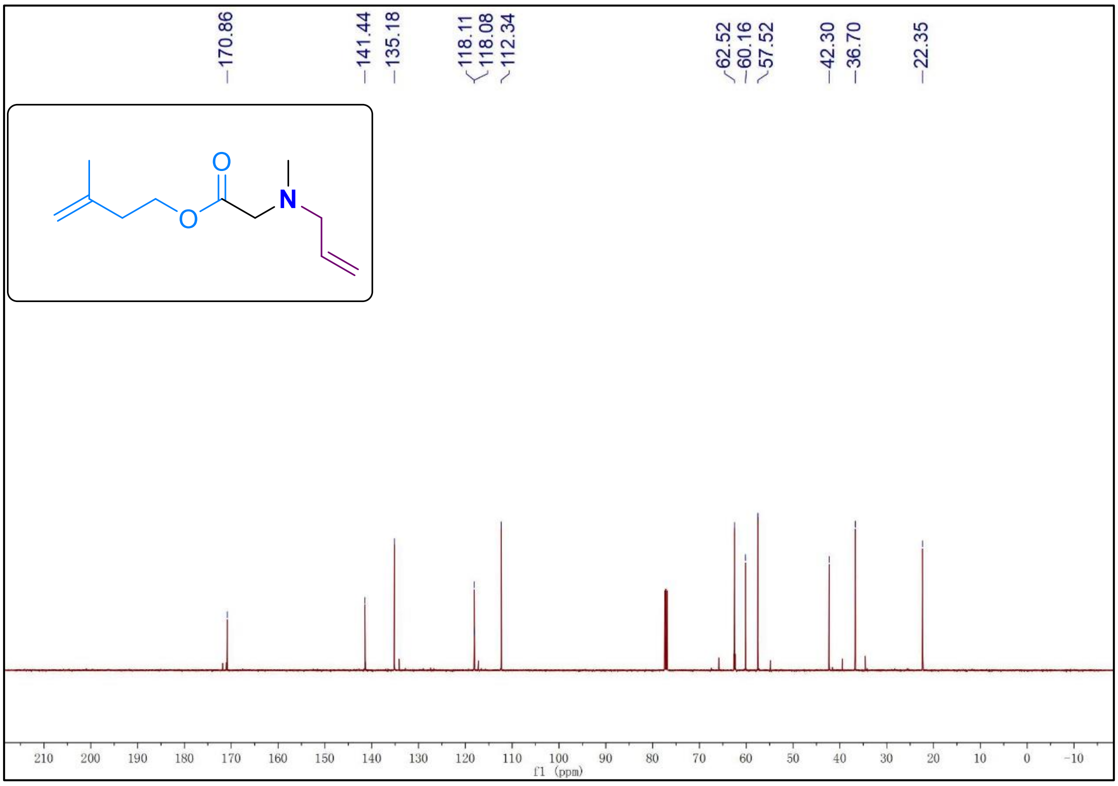

4-methylbenzyl *N*-allyl-*N*-methylglycinate

^{1}H NMR (500 MHz, Chloroform-*d*)

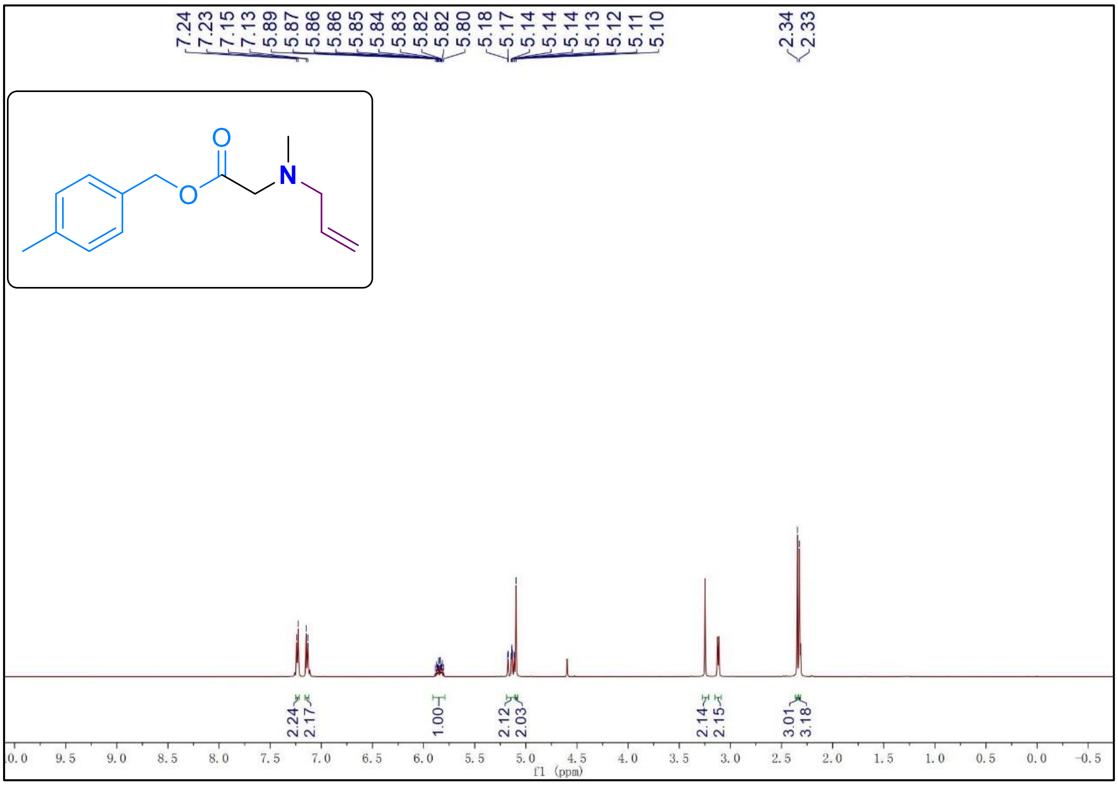

^{13}C NMR (126 MHz, Chloroform-*d*)

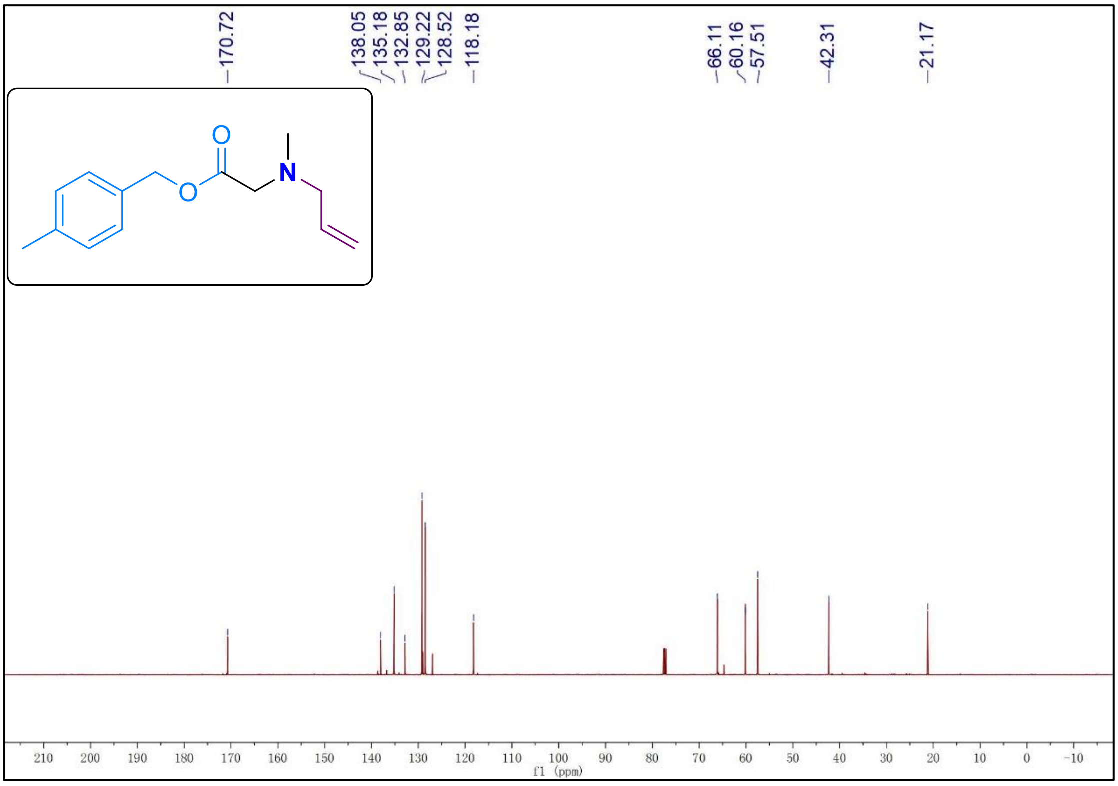

4-(*tert*-butyl)benzyl *N*-allyl-*N*-methylglycinate

^{1}H NMR (500 MHz, Chloroform-*d*)

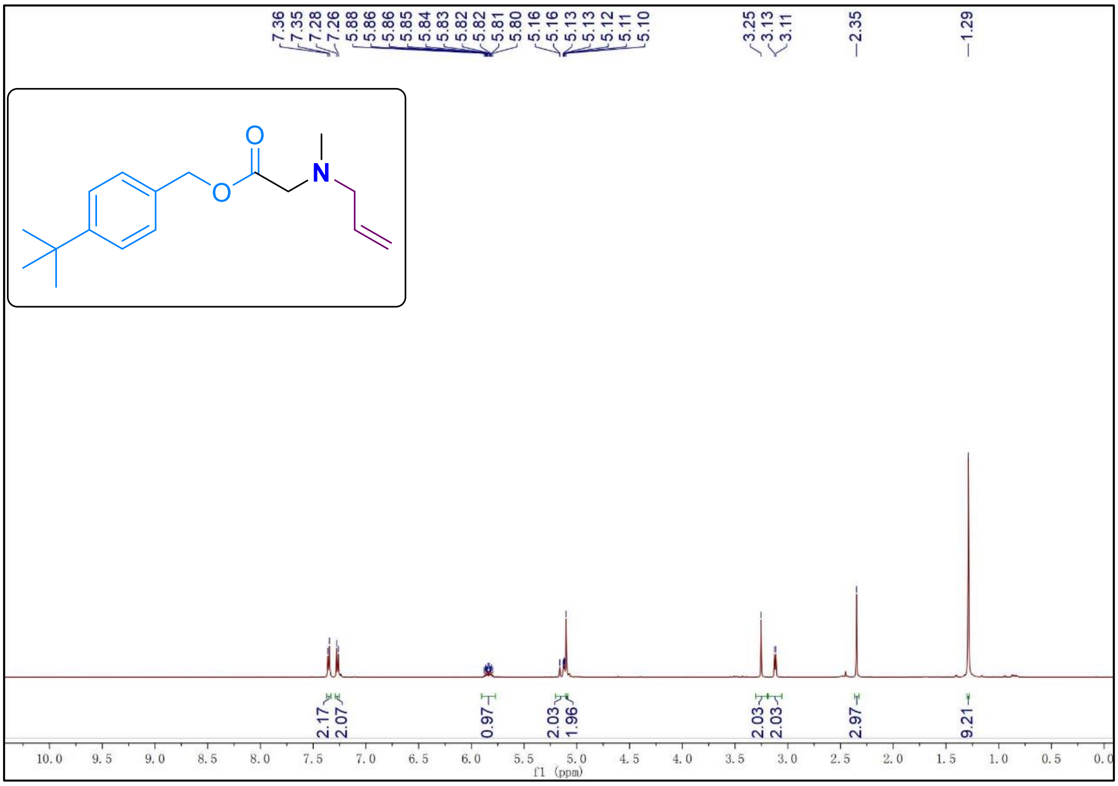

^{13}C NMR (126 MHz, Chloroform-*d*)

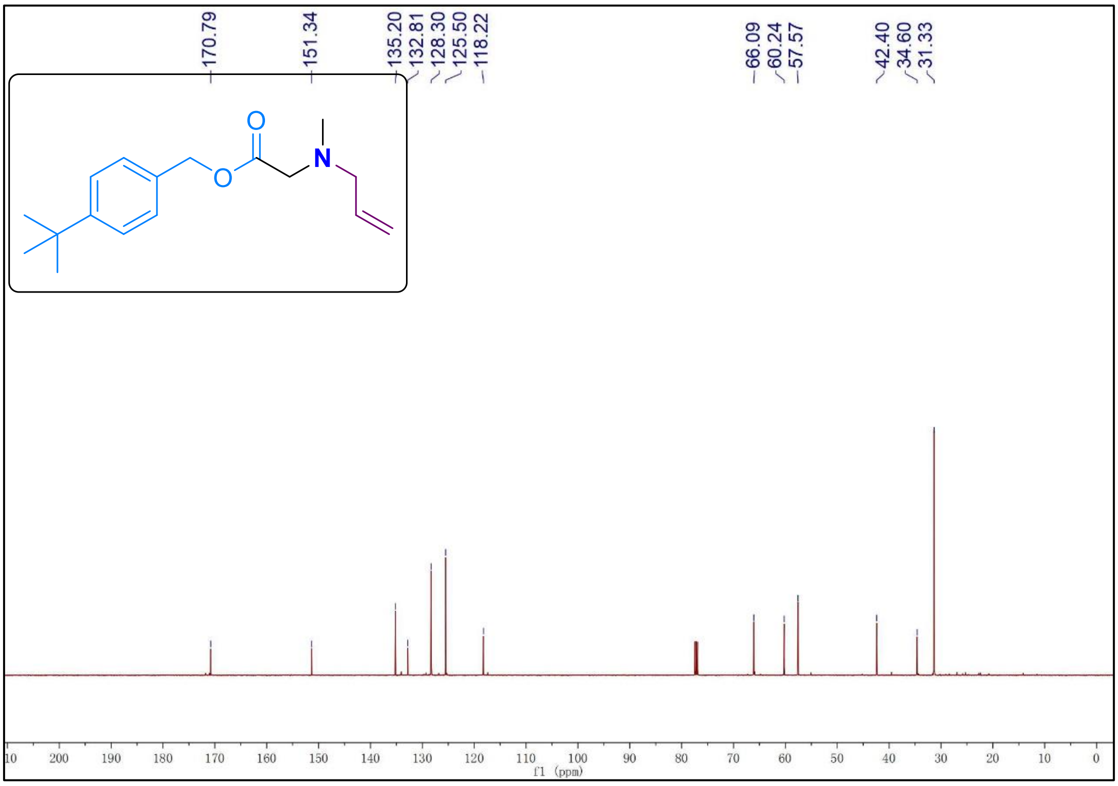

[1,1'-biphenyl]-4-ylmethyl N-allyl-N-methylglycinate

^{1}H NMR (500 MHz, Chloroform-*d*)

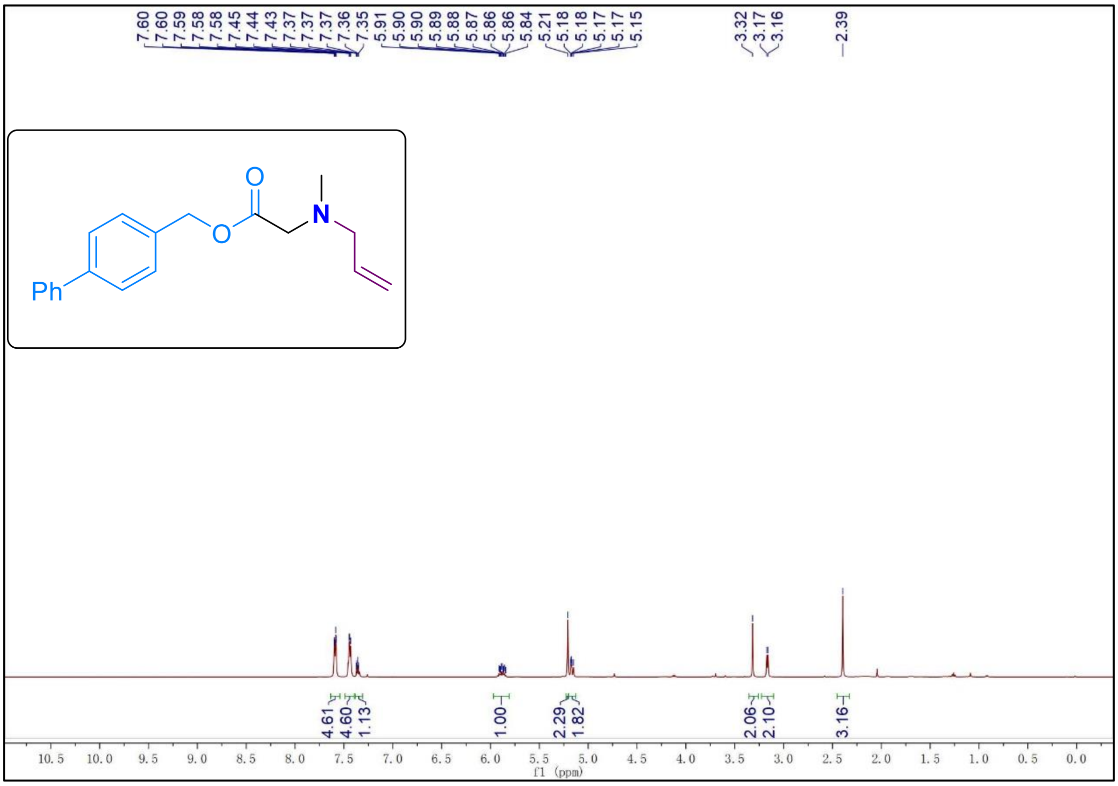

^{13}C NMR (126 MHz, Chloroform-*d*)

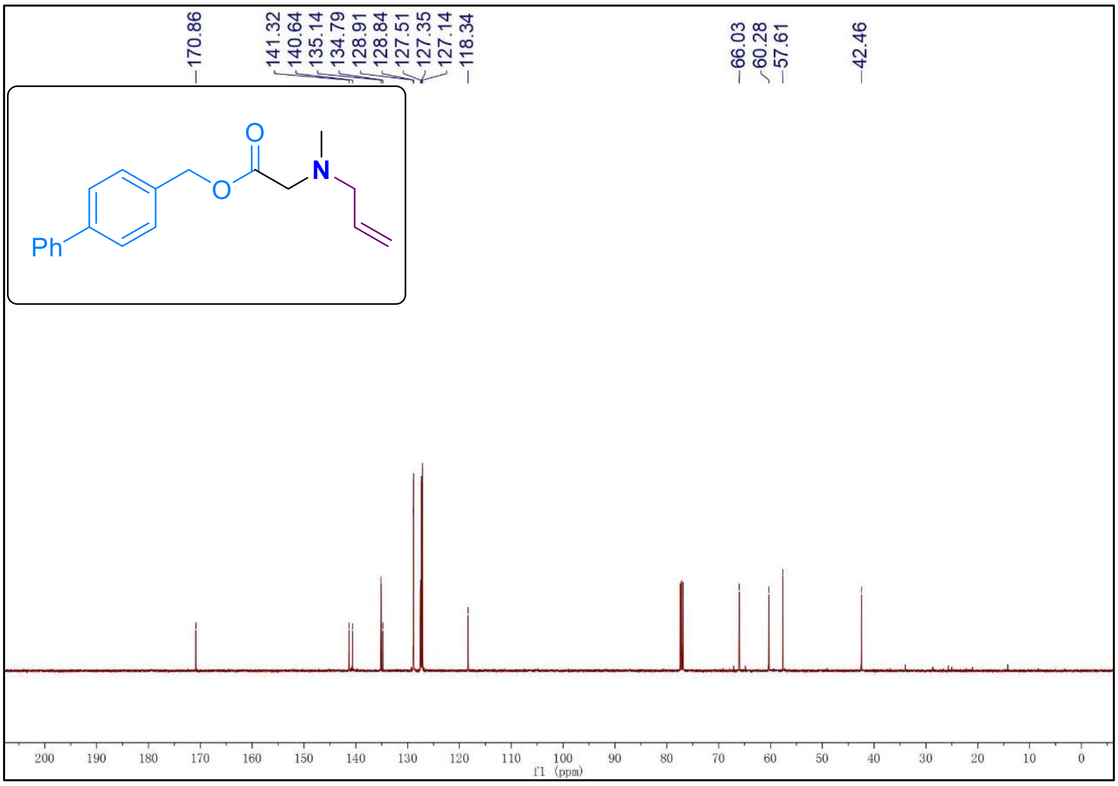

2-iodobenzyl *N*-allyl-*N*-methylglycinate

^{1}H NMR (500 MHz, Chloroform-*d*)

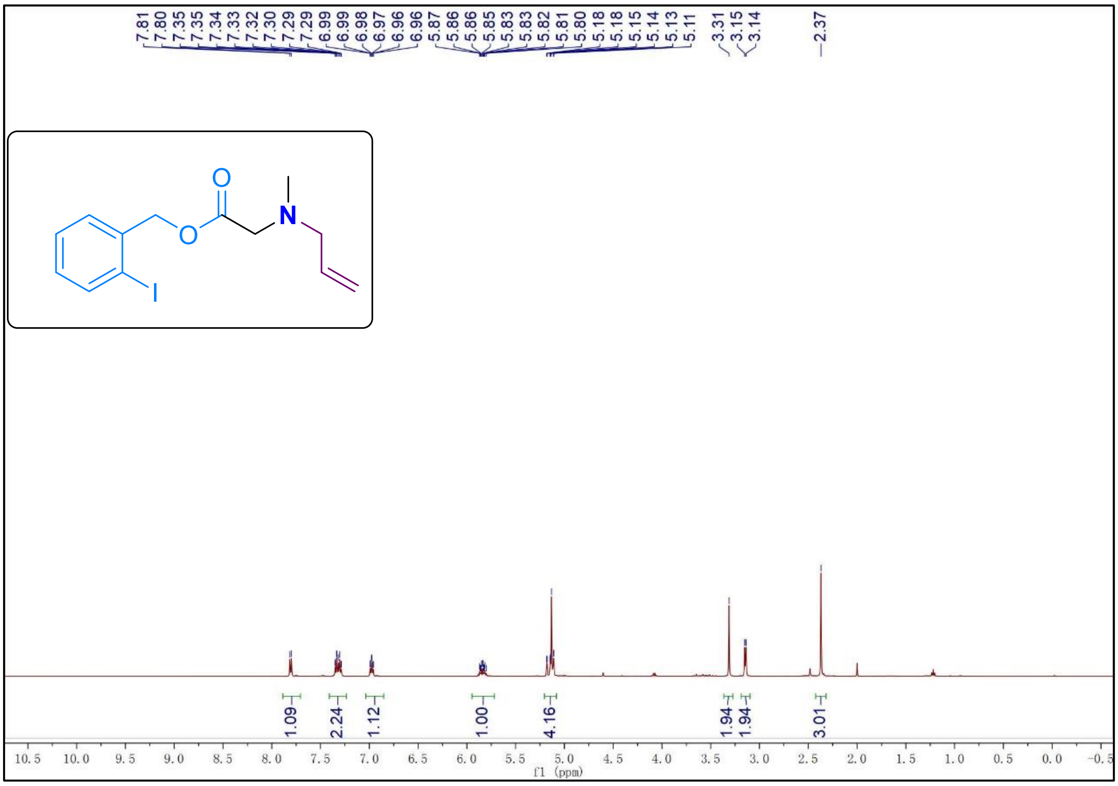

^{13}C NMR (126 MHz, Chloroform-*d*)

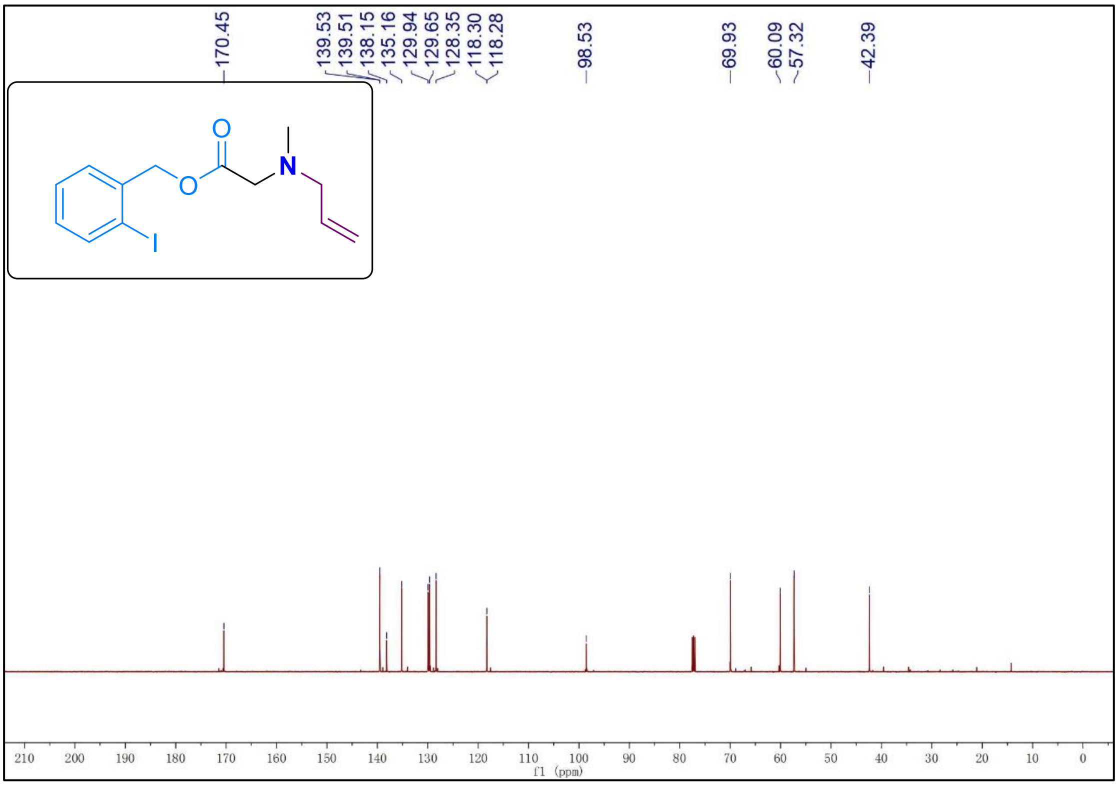

3,5-dichlorobenzyl *N*-allyl-*N*-methylglycinate

^{1}H NMR (500 MHz, Chloroform-*d*)

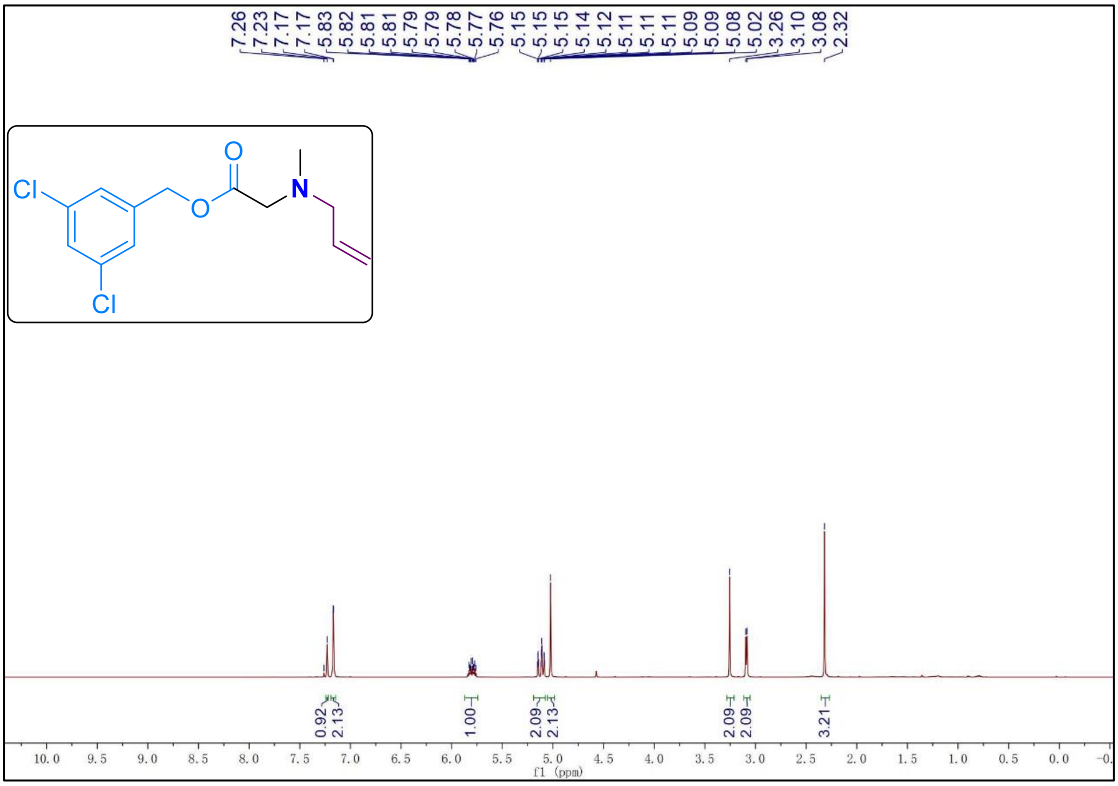

^{13}C NMR (126 MHz, Chloroform-*d*)

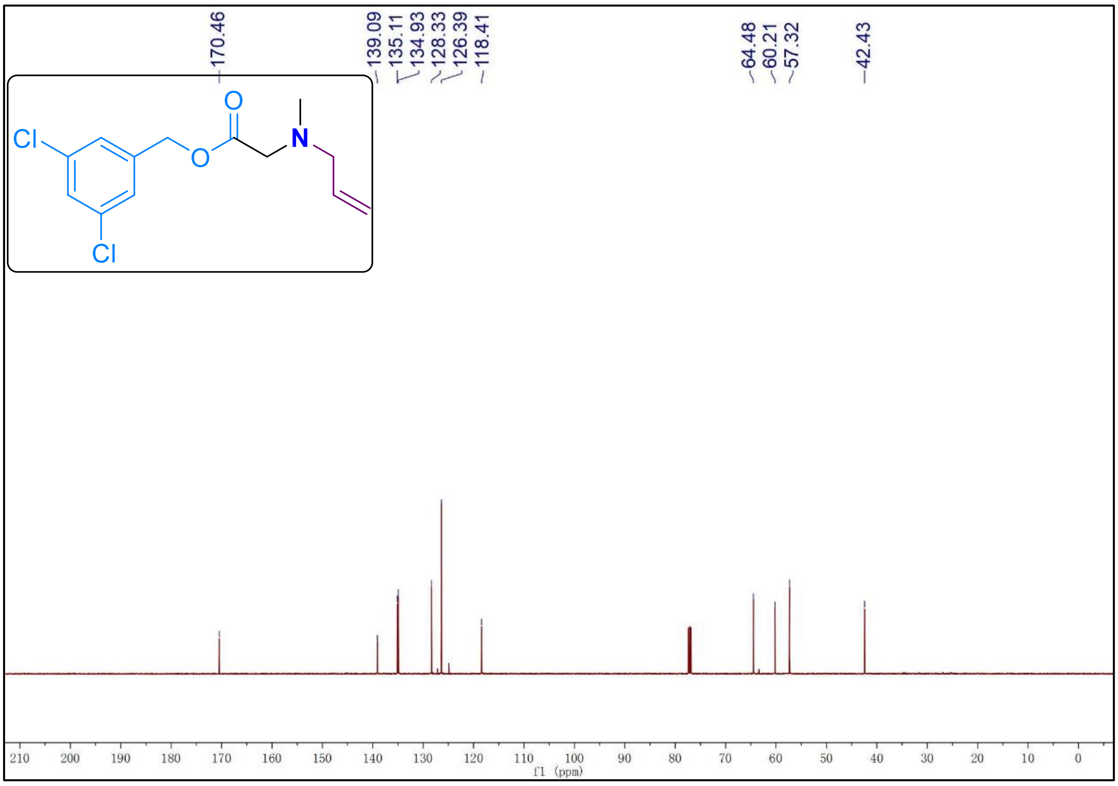

cinnamyl *N*-allyl-*N*-methylglycinate

^{1}H NMR (500 MHz, Chloroform-*d*)

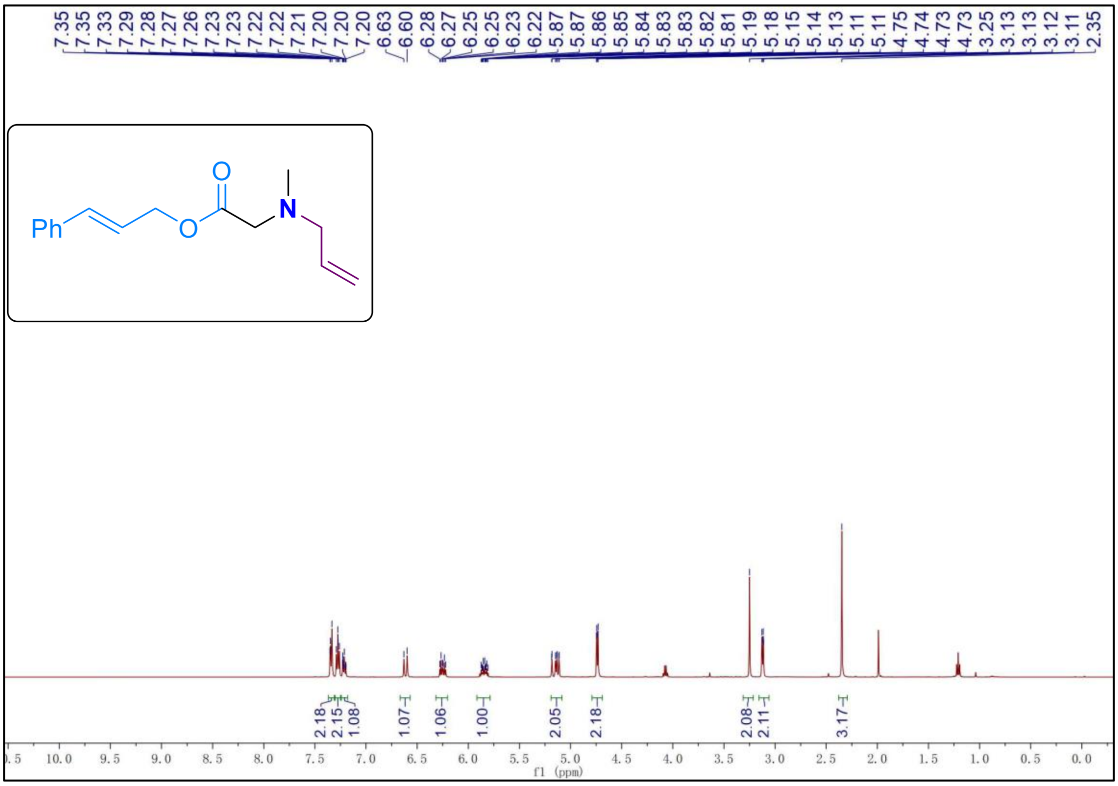

^{13}C NMR (126 MHz, Chloroform-*d*)

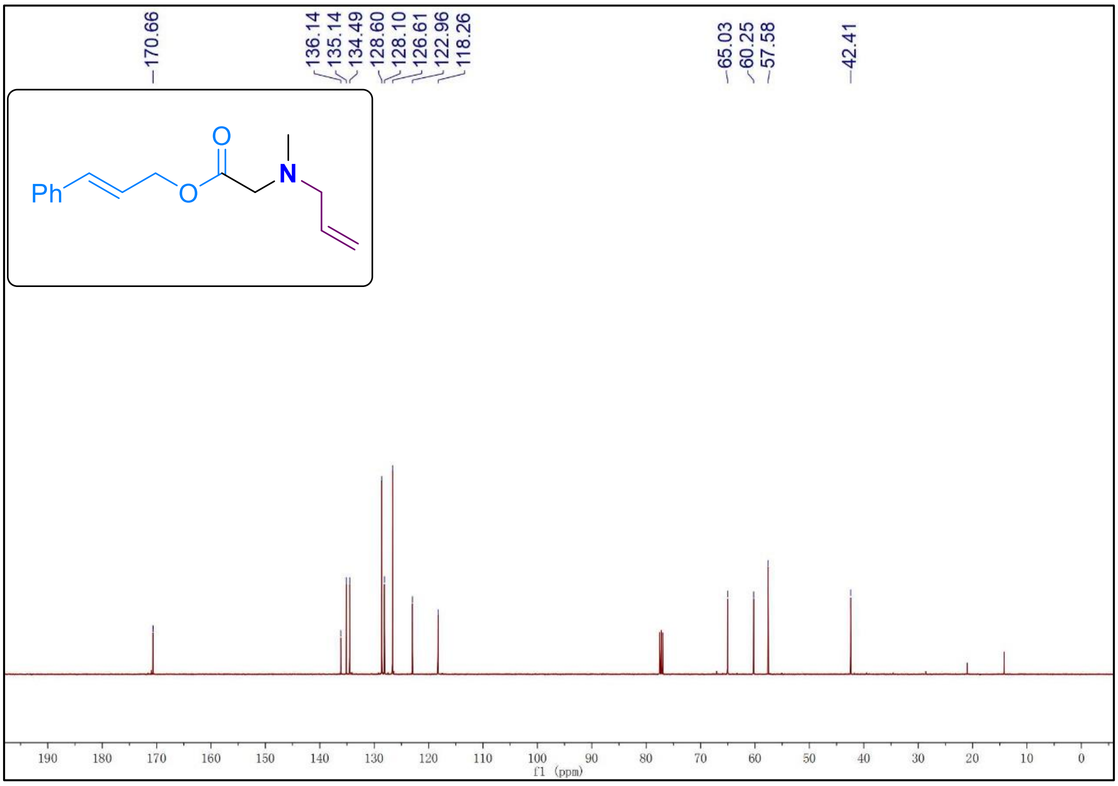

2-(benzyloxy)ethyl *N*-allyl-*N*-methylglycinate

^{1}H NMR (500 MHz, Chloroform-*d*)

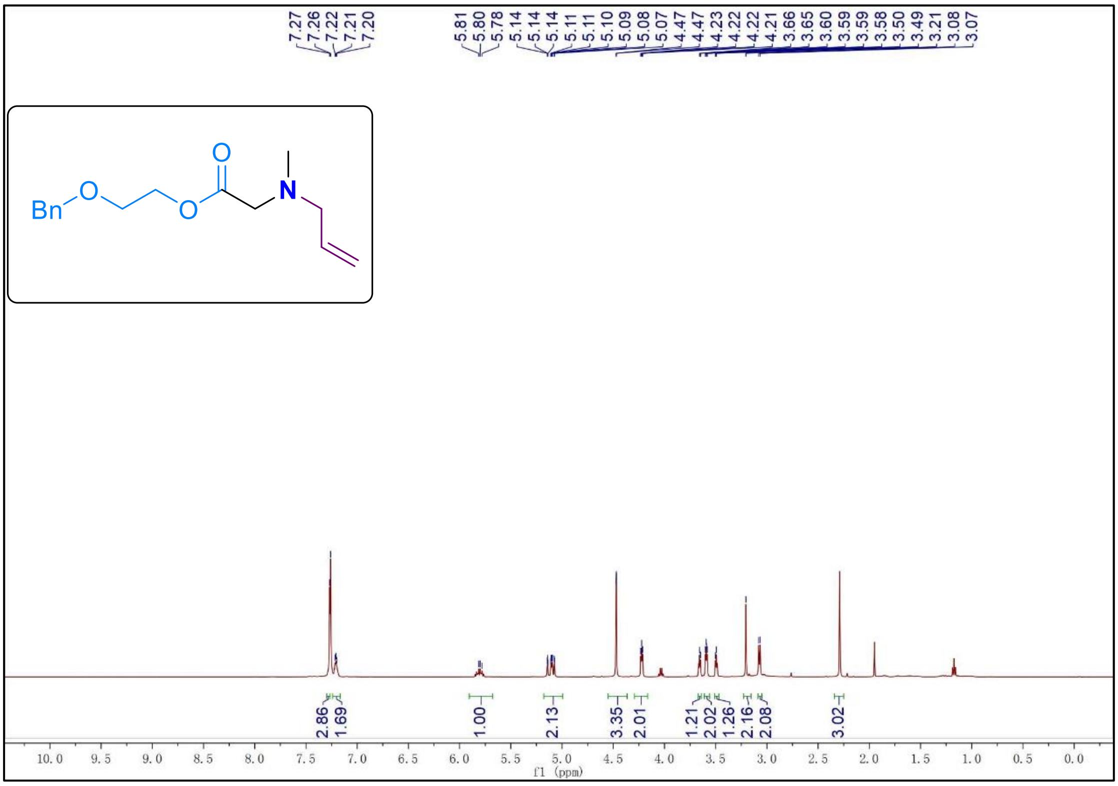

^{13}C NMR (126 MHz, Chloroform-*d*)

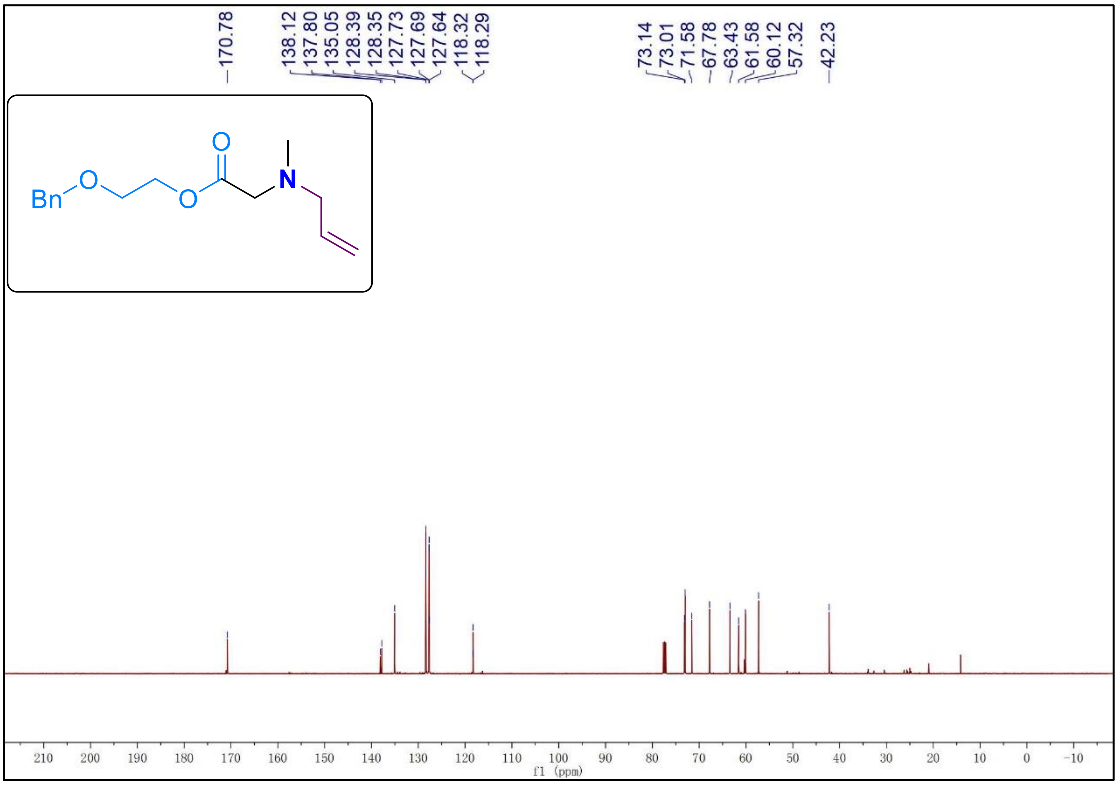

2-(allyl(methyl)amino)-1-cyclopropylethan-1-one

^{1}H NMR (500 MHz, Chloroform-*d*)

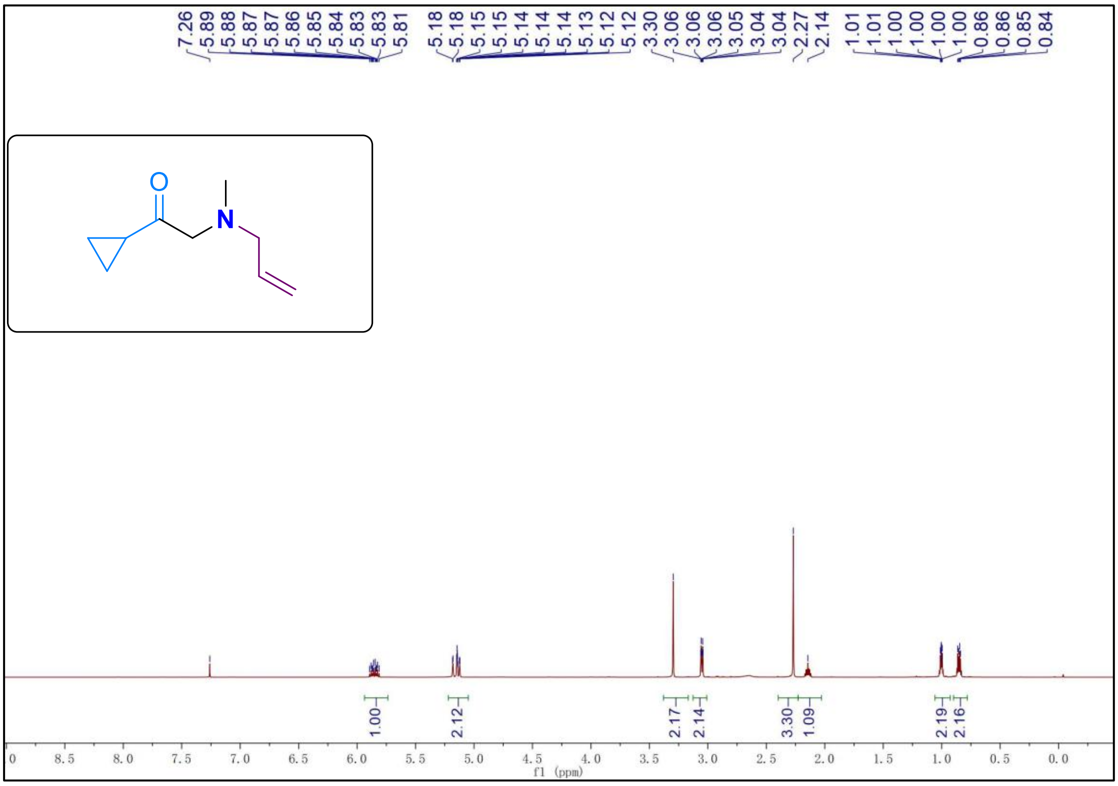

^{13}C NMR (126 MHz, Chloroform-*d*)

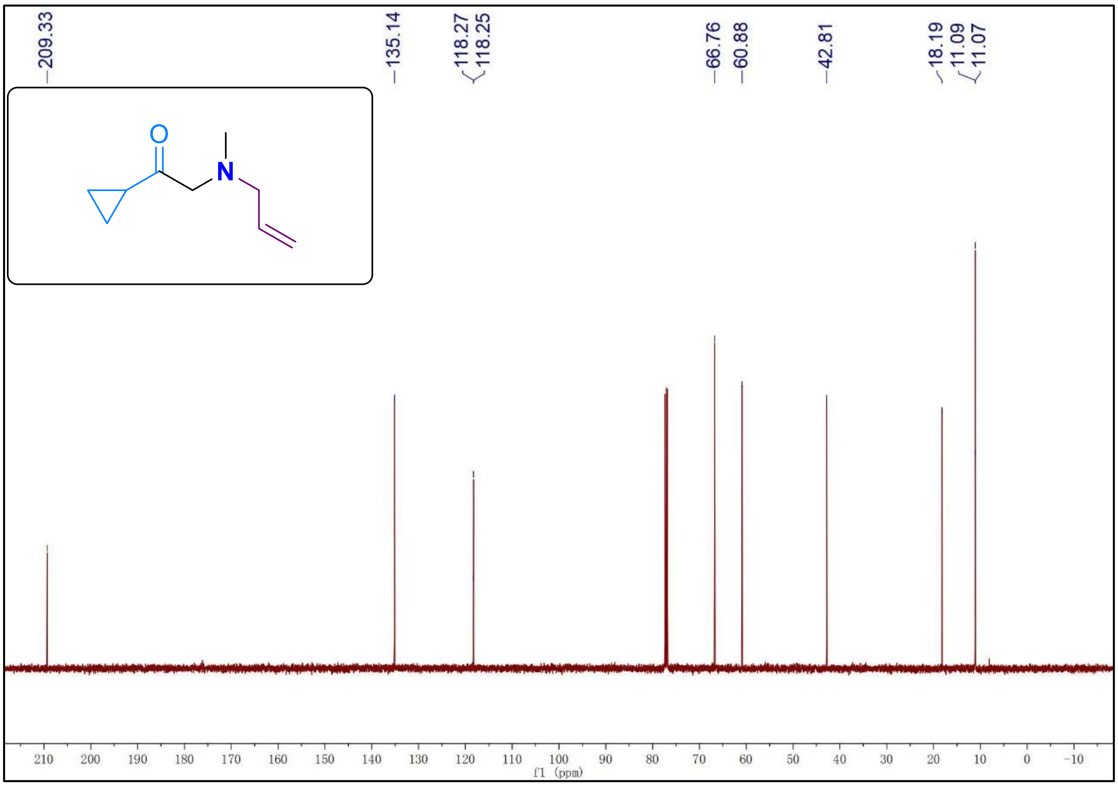

1-(allyl(methyl)amino)-3,3-dimethylbutan-2-one

^{1}H NMR (500 MHz, Chloroform-*d*)

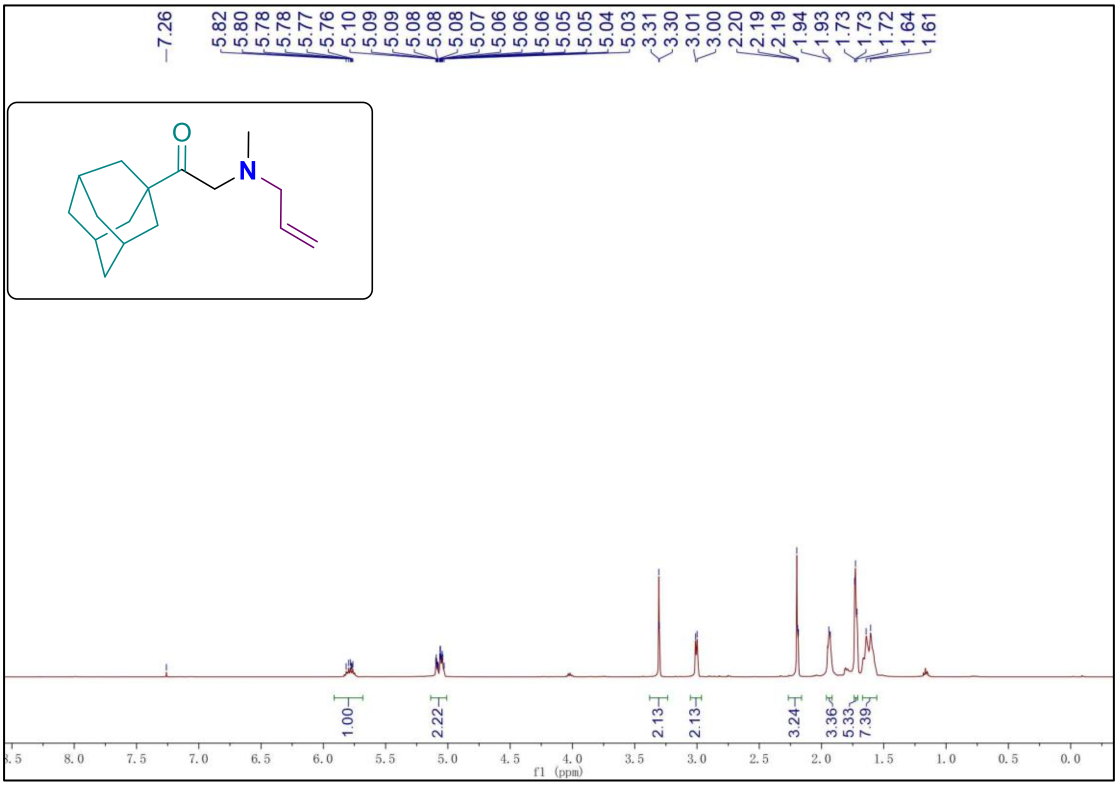

^{13}C NMR (126 MHz, Chloroform-*d*)

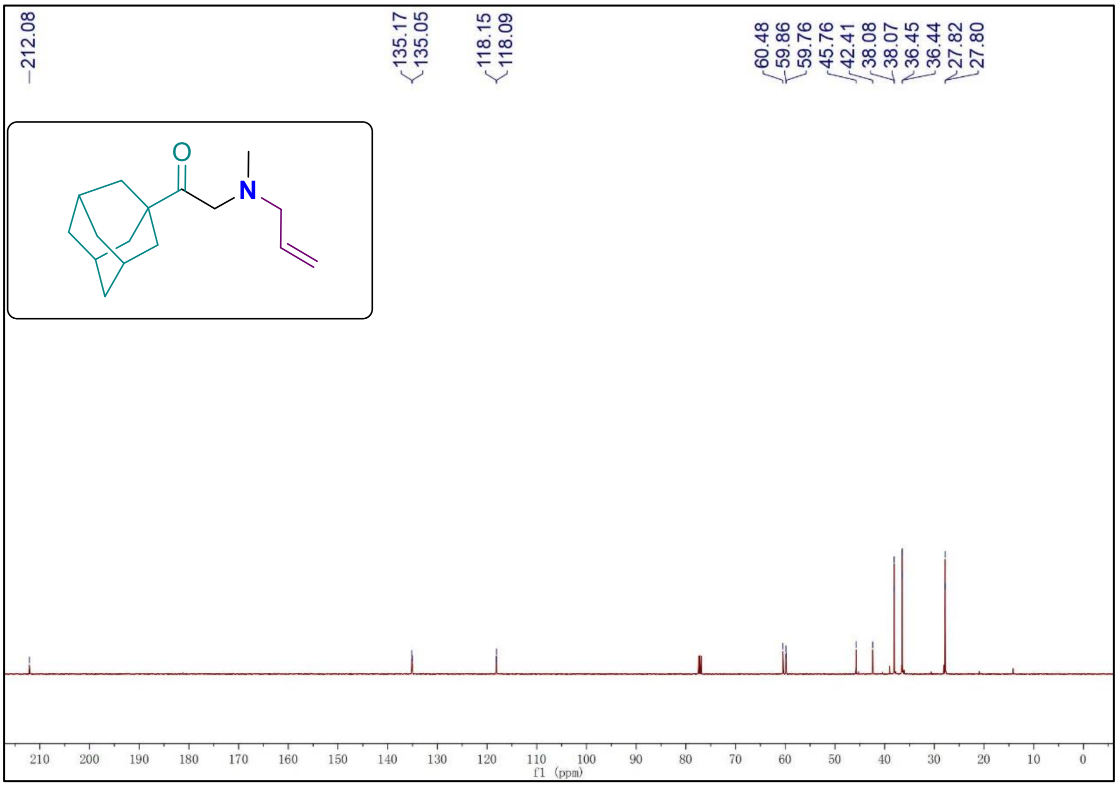

2-(allyl(methyl)amino)-1-(4-hydroxyphenyl)ethan-1-one

^{1}H NMR (500 MHz, Chloroform-*d*)

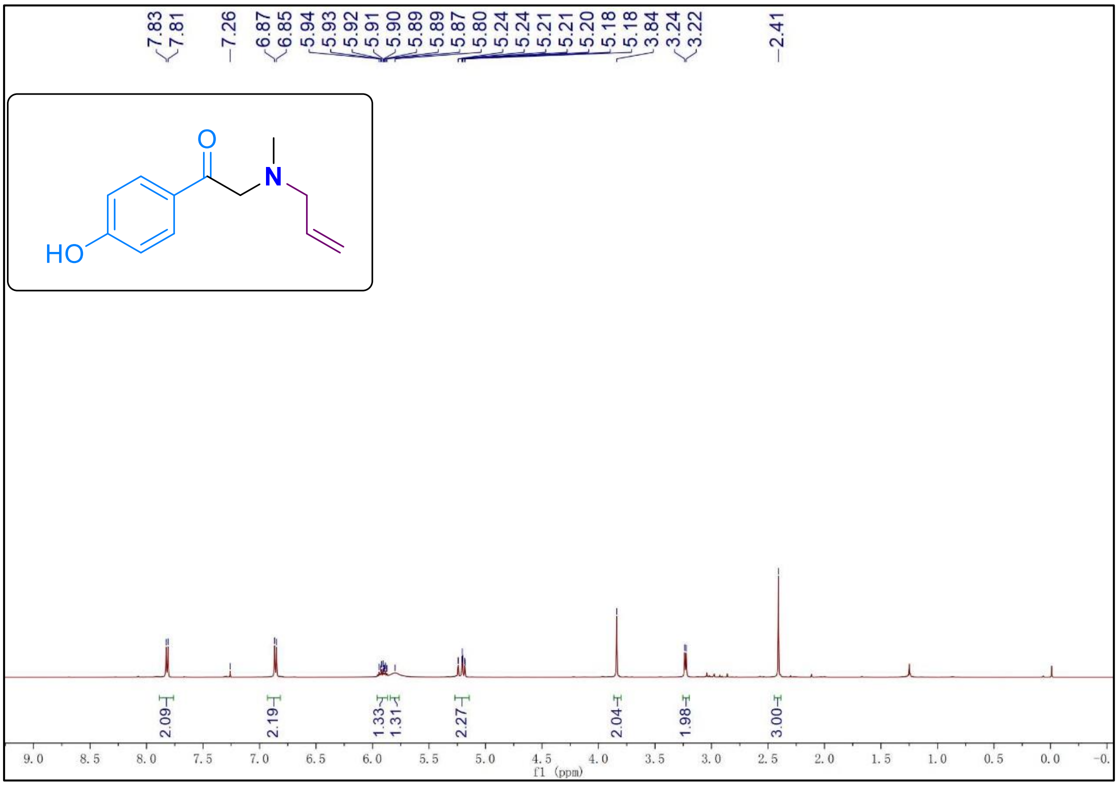

^{13}C NMR (126 MHz, Chloroform-*d*)

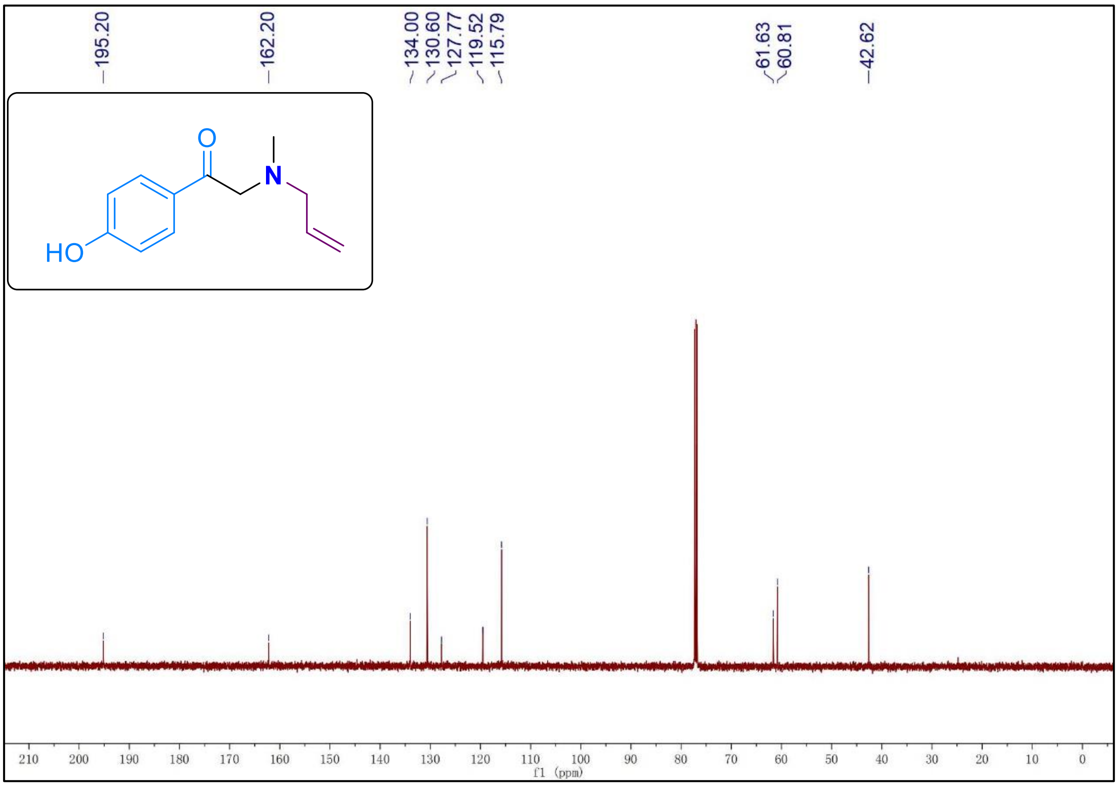

2-(allyl(methyl)amino)-1-(thiophen-2-yl)ethan-1-one

^{1}H NMR (500 MHz, Chloroform-*d*)

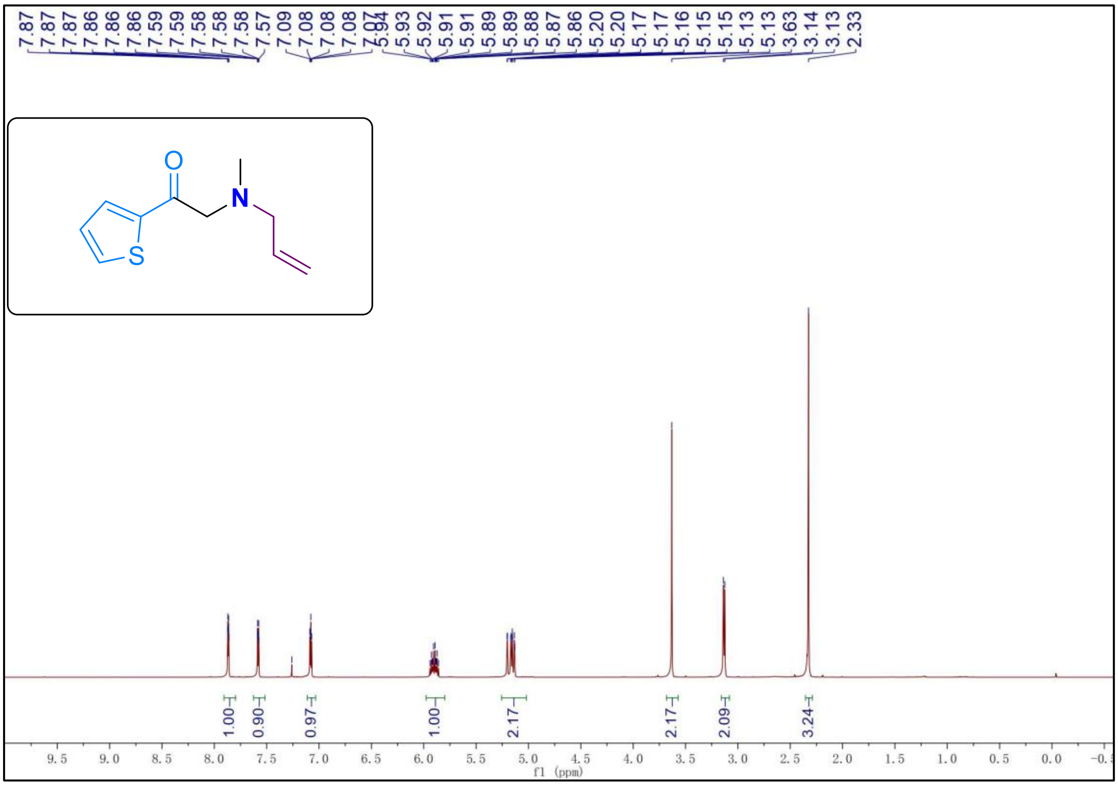

^{13}C NMR (126 MHz, Chloroform-*d*)

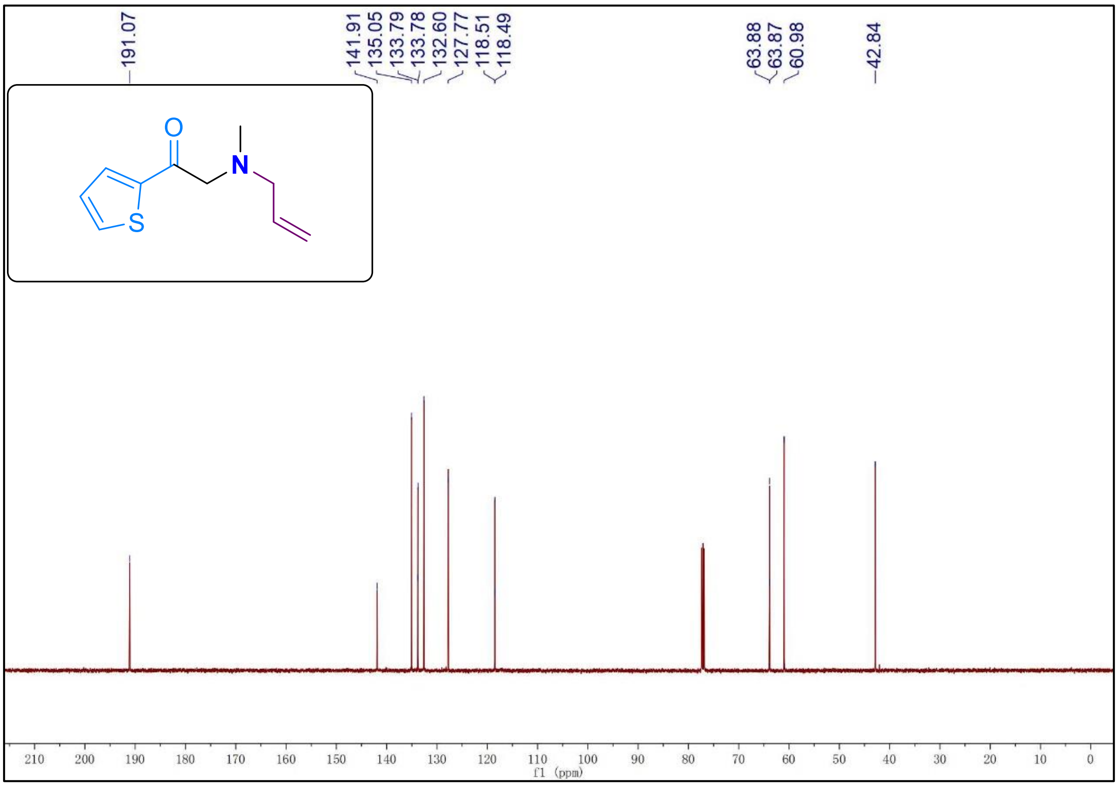

2-(allyl(methyl)amino)acetonitrile

^{1}H NMR (500 MHz, Chloroform-*d*)

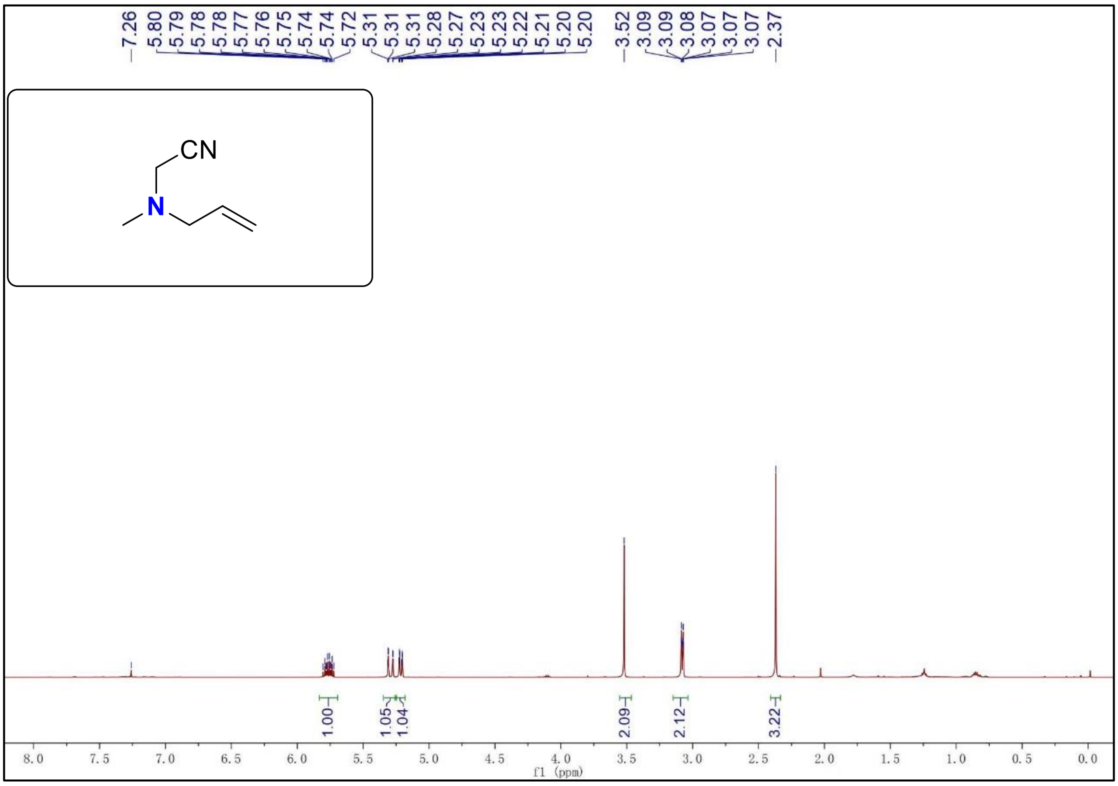

^{13}C NMR (126 MHz, Chloroform-*d*)

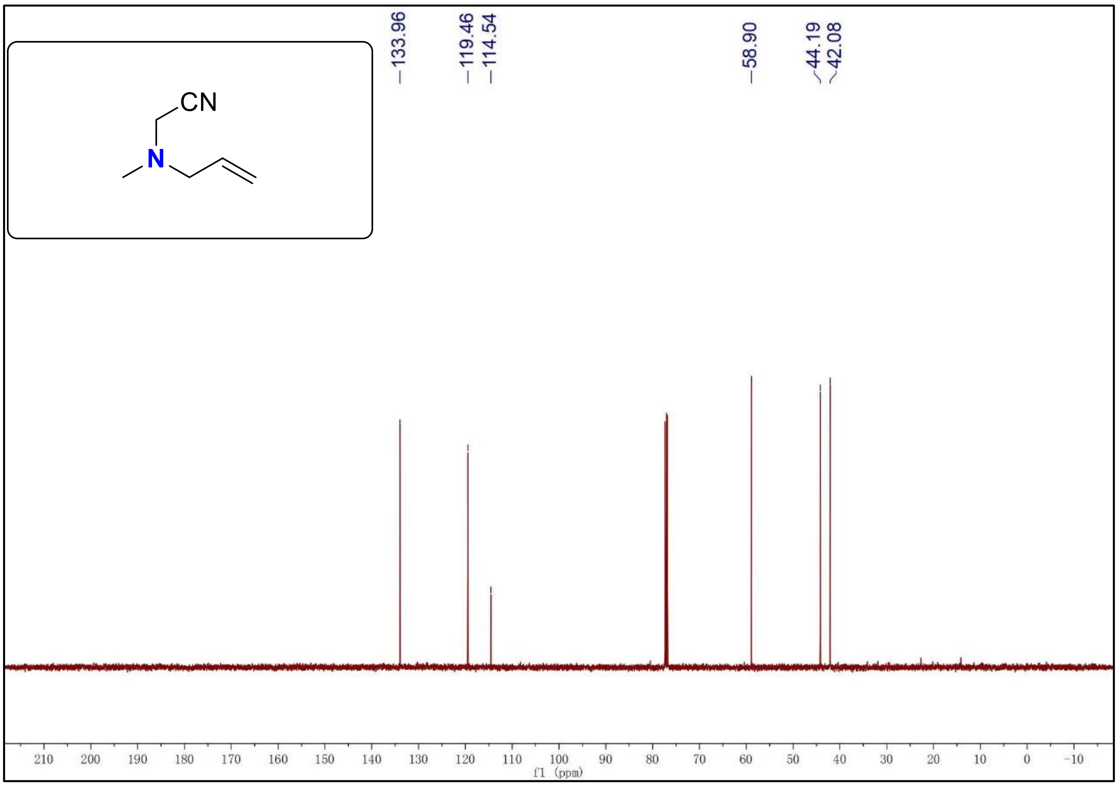

diethyl ((allyl(methyl)amino)methyl)phosphonate

^{1}H NMR (500 MHz, Chloroform-*d*)

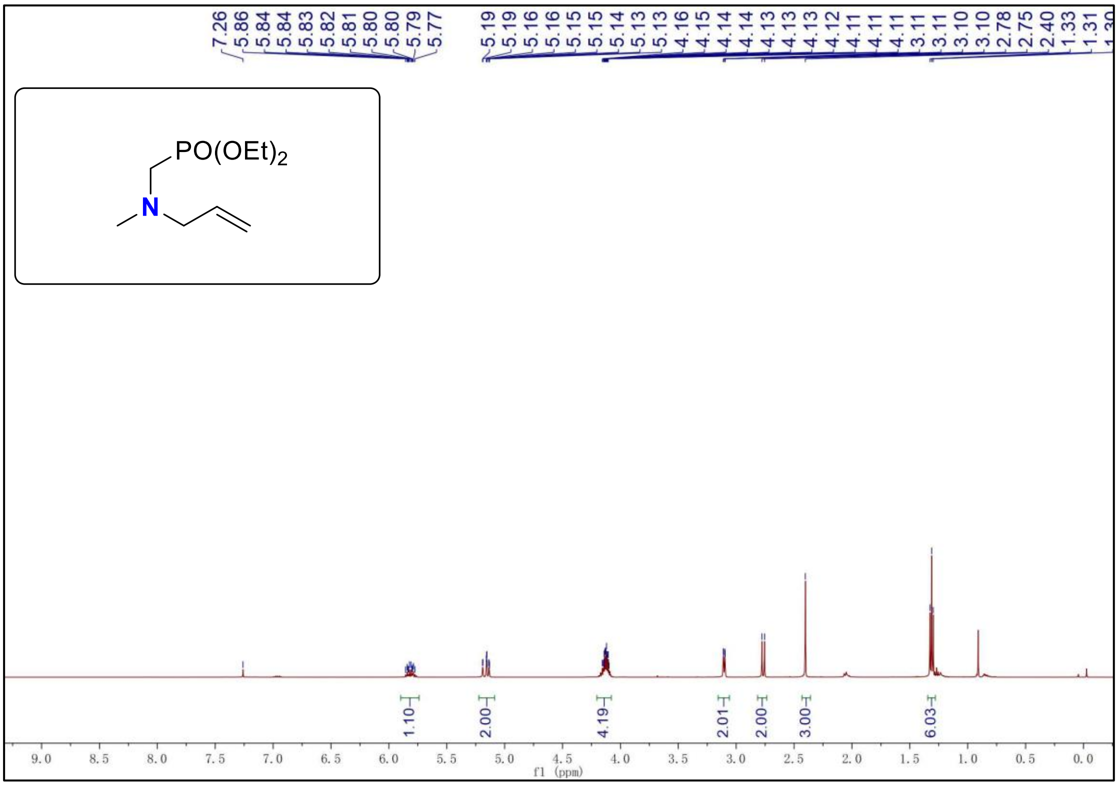

^{13}C NMR (126 MHz, Chloroform-*d*)

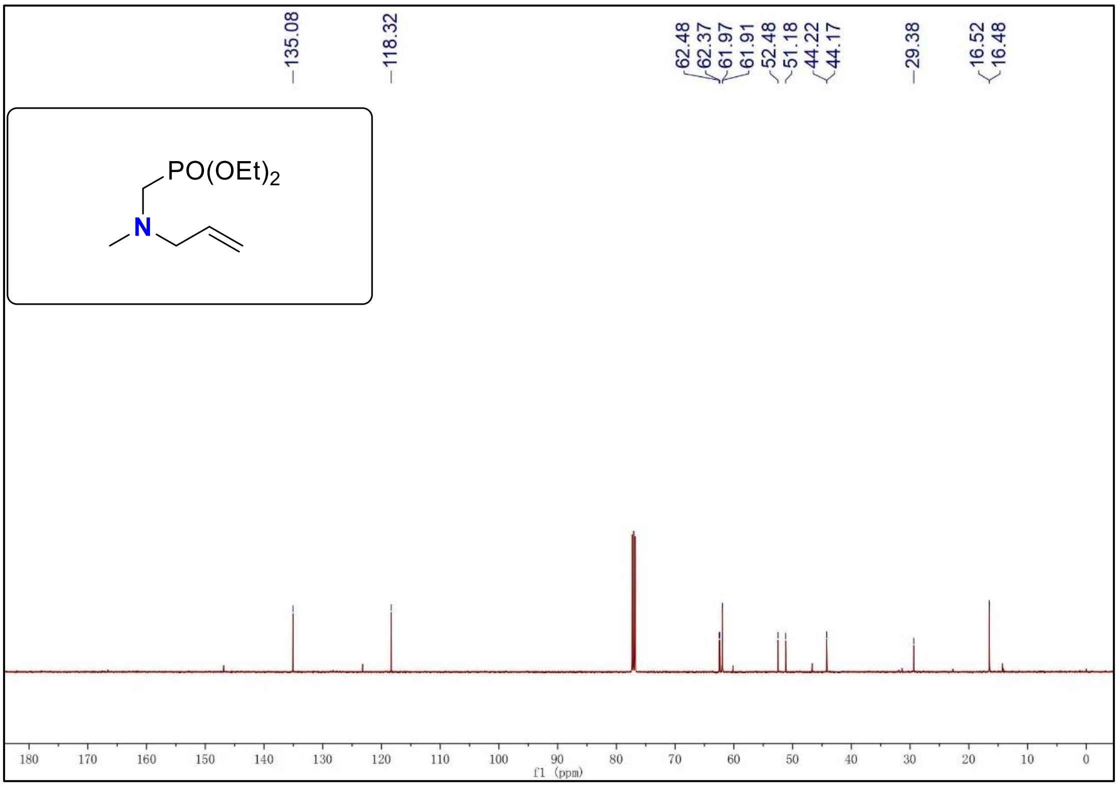

^{31}P NMR (202 MHz, Chloroform-*d*)

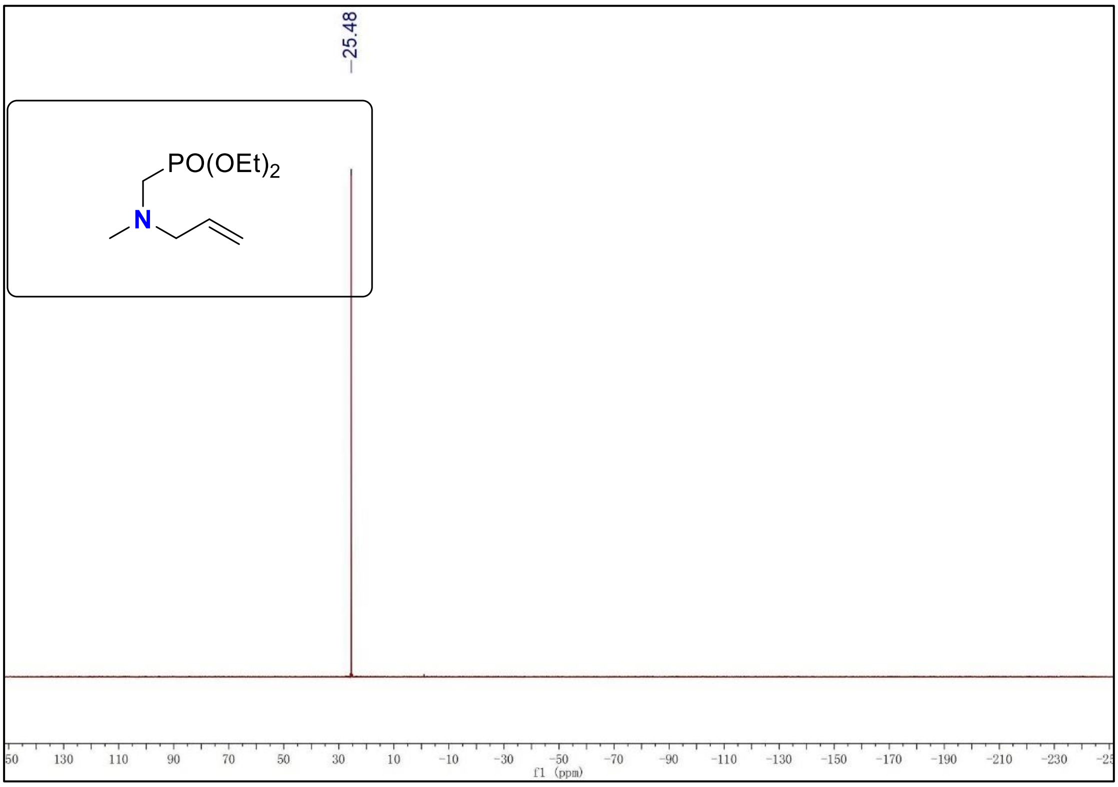

methyl *N*-allyl-*N*-methylalaninate

^{1}H NMR (500 MHz, Chloroform-*d*)

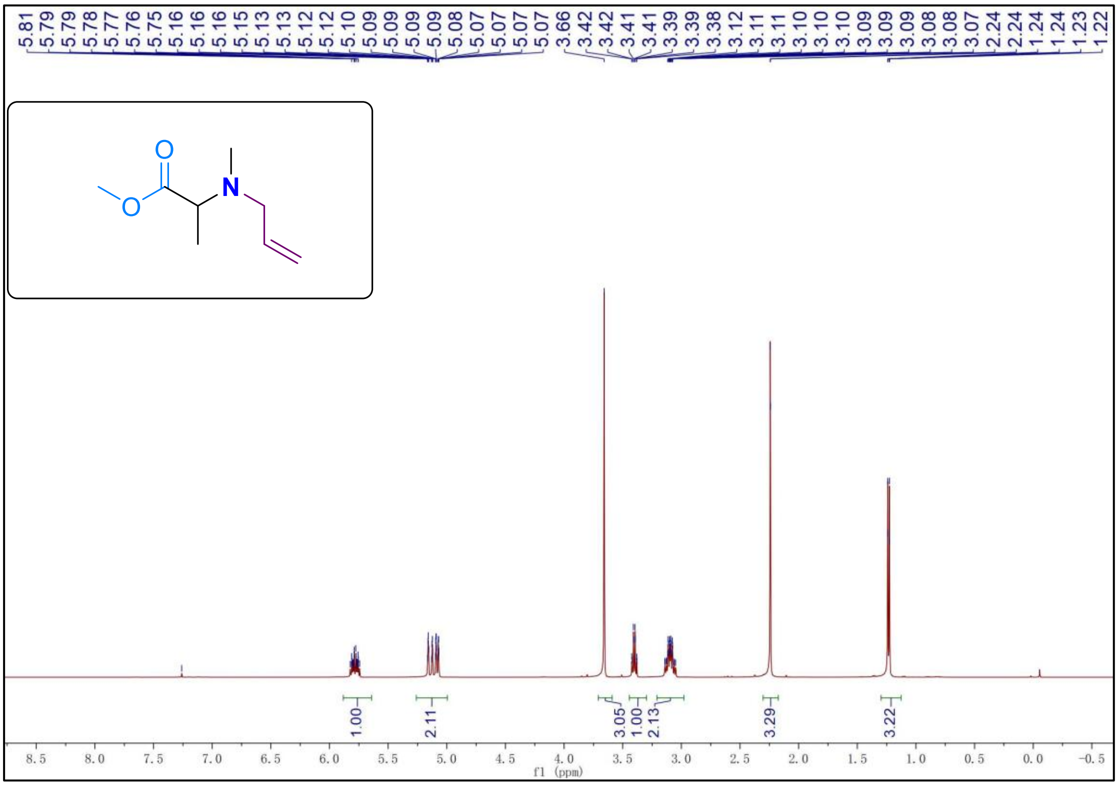

^{13}C NMR (126 MHz, Chloroform-*d*)

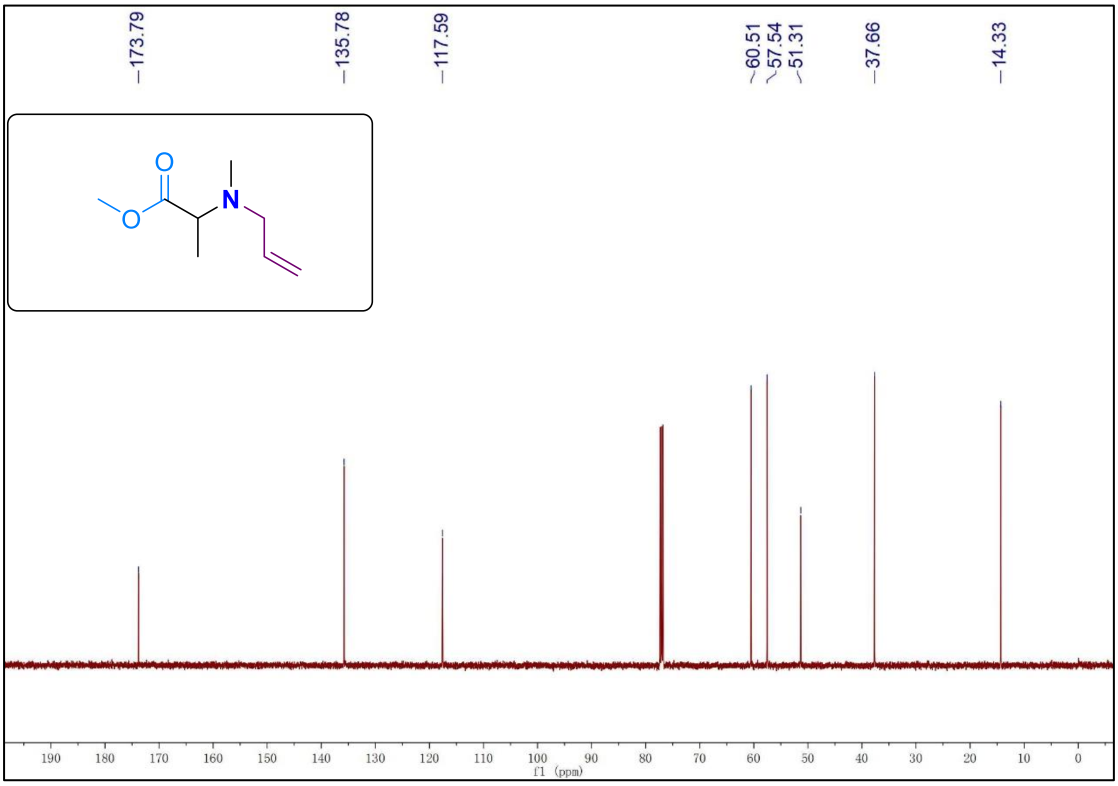

ethyl *N*-allyl-*N*-methylalaninate

^{1}H NMR (500 MHz, Chloroform-*d*)

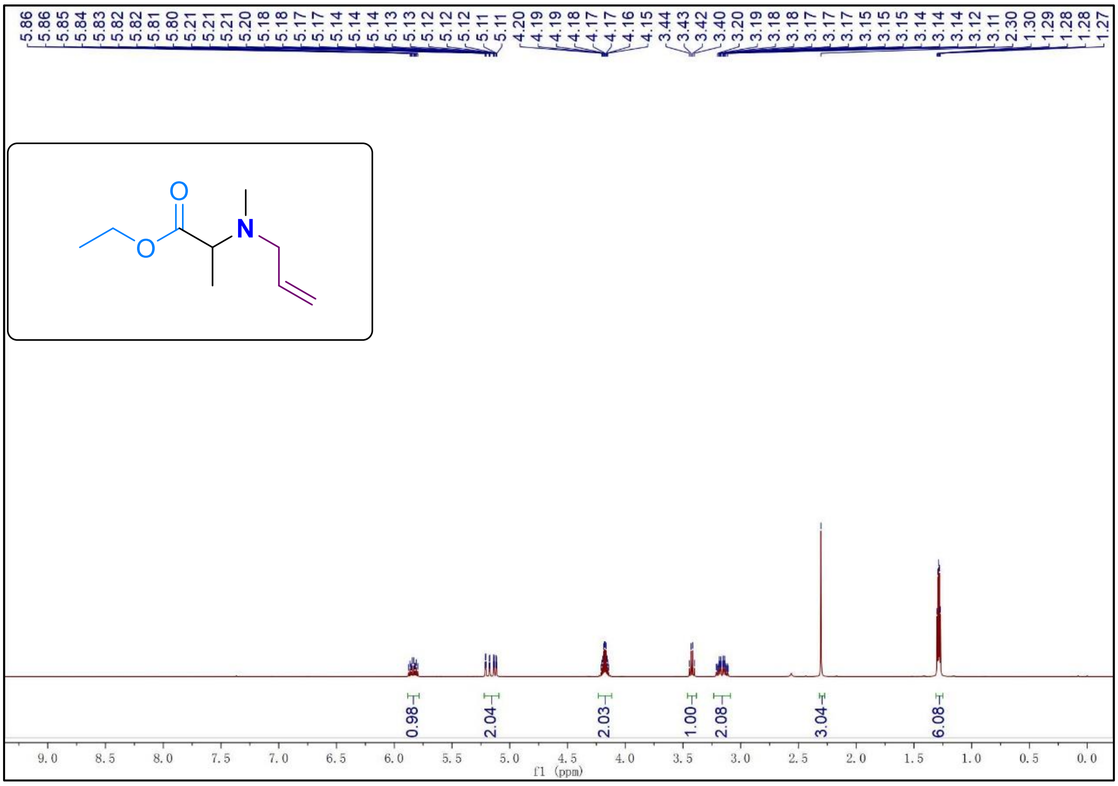

^{13}C NMR (126 MHz, Chloroform-*d*)

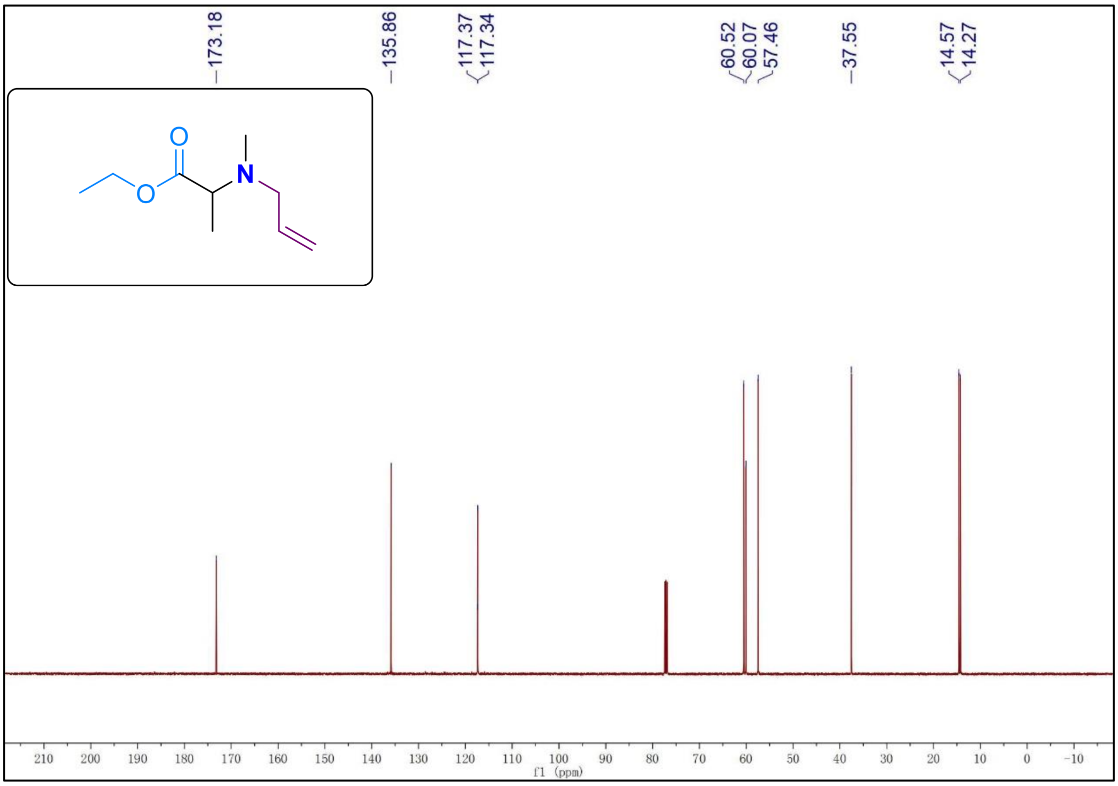

4-iodobenzyl *N*-allyl-*N*-methylalaninate

^{1}H NMR (500 MHz, Chloroform-*d*)

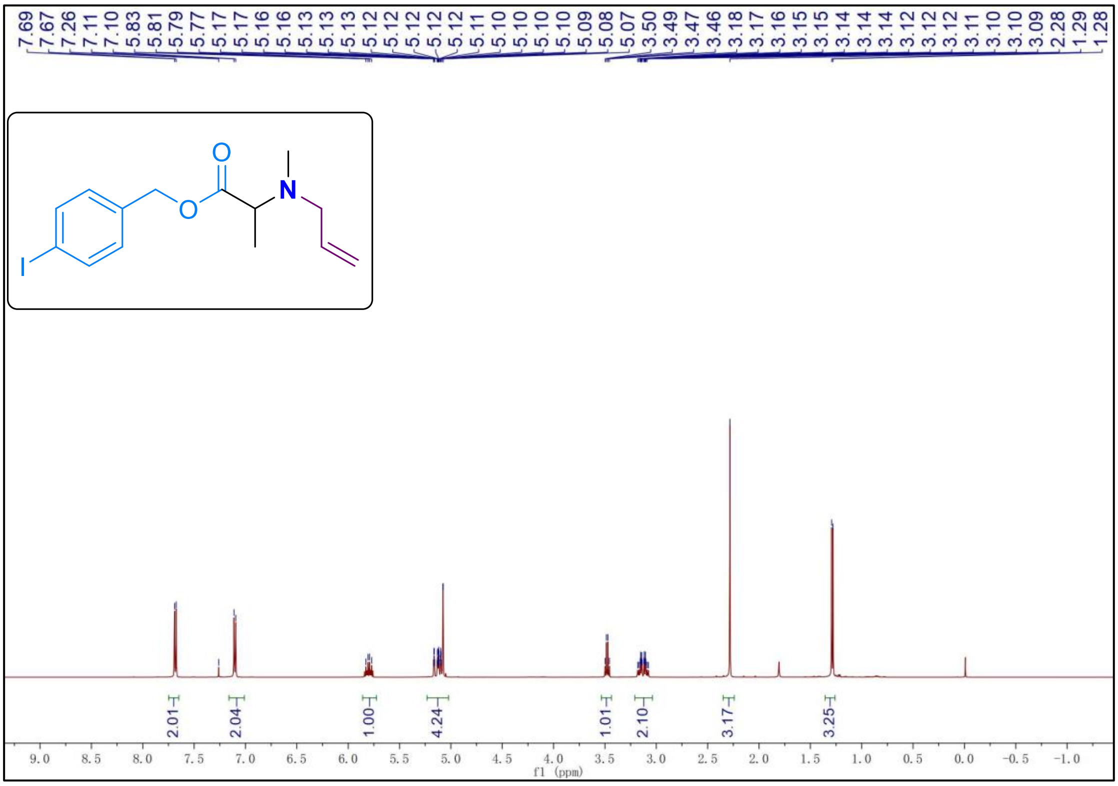

^{13}C NMR (126 MHz, Chloroform-*d*)

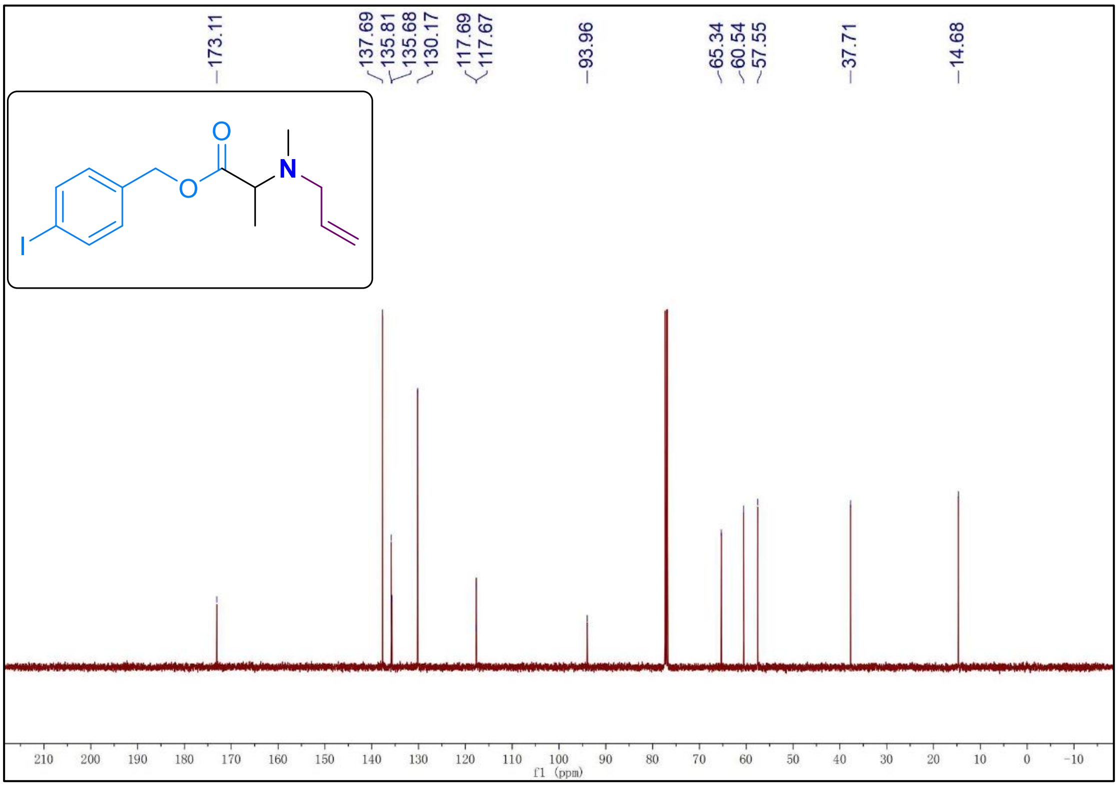

ethyl 2-(allyl(methyl)amino)-2-phenylacetate

^{1}H NMR (500 MHz, Chloroform-*d*)

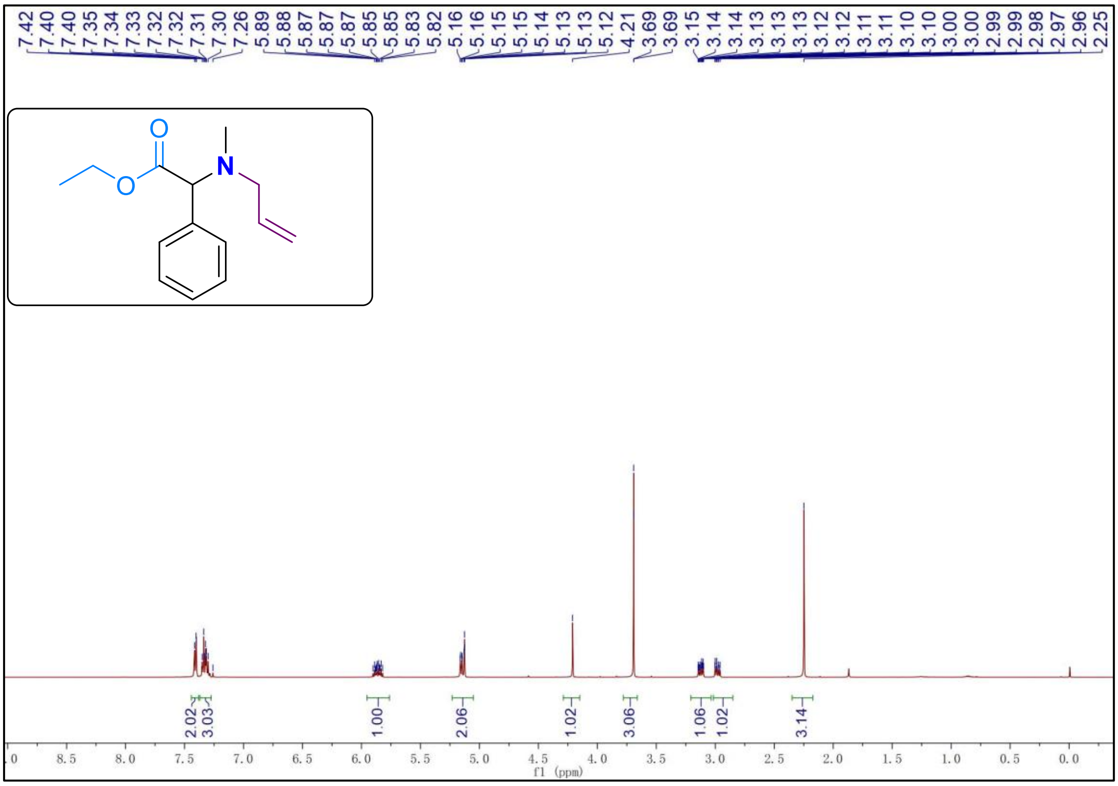

^{13}C NMR (126 MHz, Chloroform-*d*)

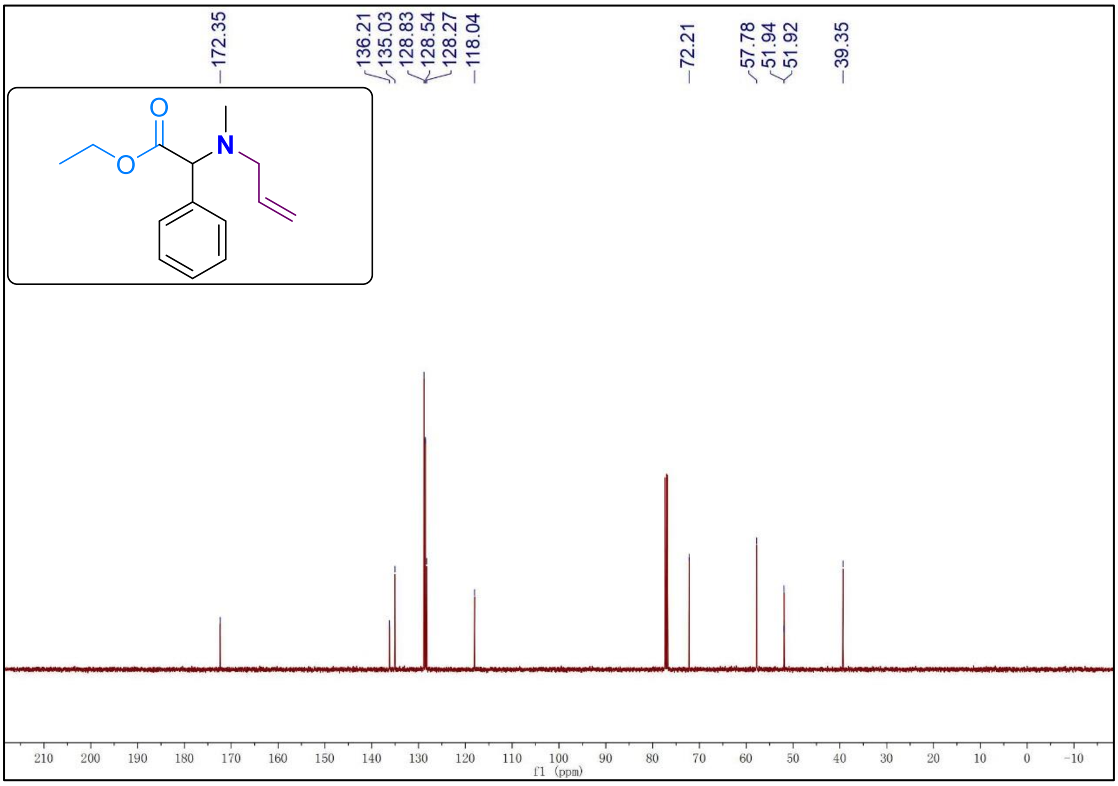

3-(allyl(methyl)amino)dihydrofuran-2(3H)-one

^{1}H NMR (500 MHz, Chloroform-*d*)

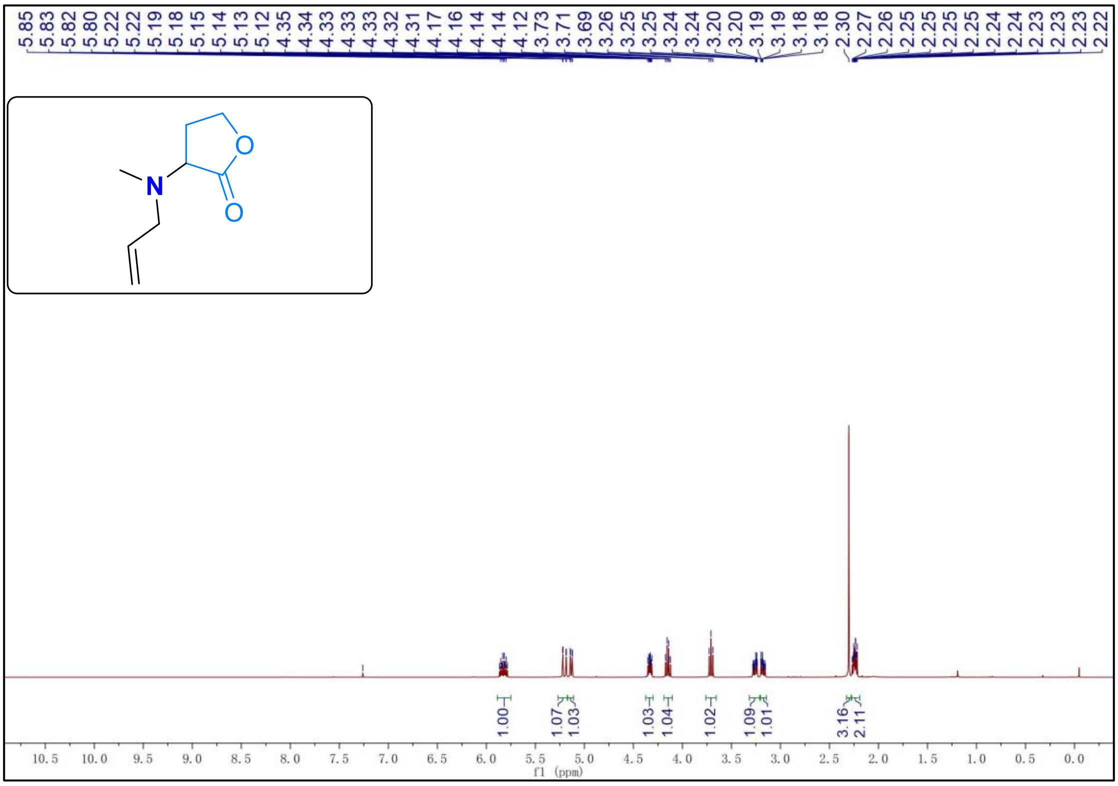

^{13}C NMR (126 MHz, Chloroform-*d*)

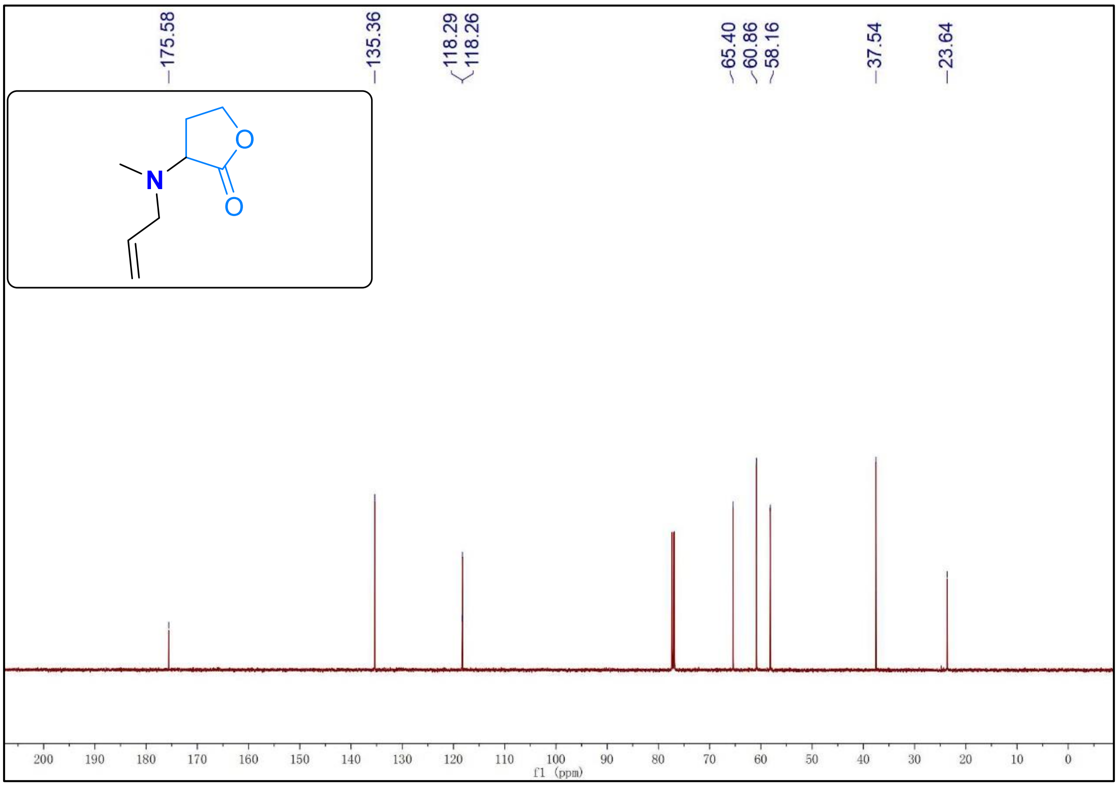

methyl allylprolinate

^{1}H NMR (500 MHz, Chloroform-*d*)

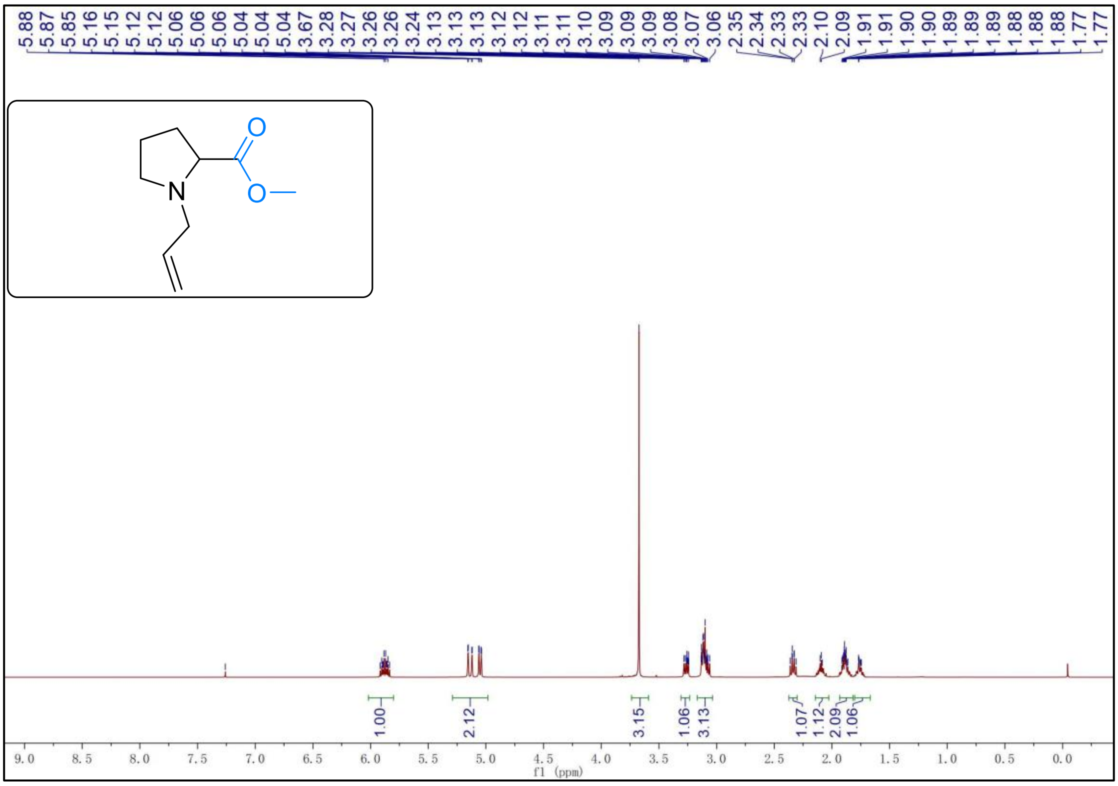

^{13}C NMR (126 MHz, Chloroform-*d*)

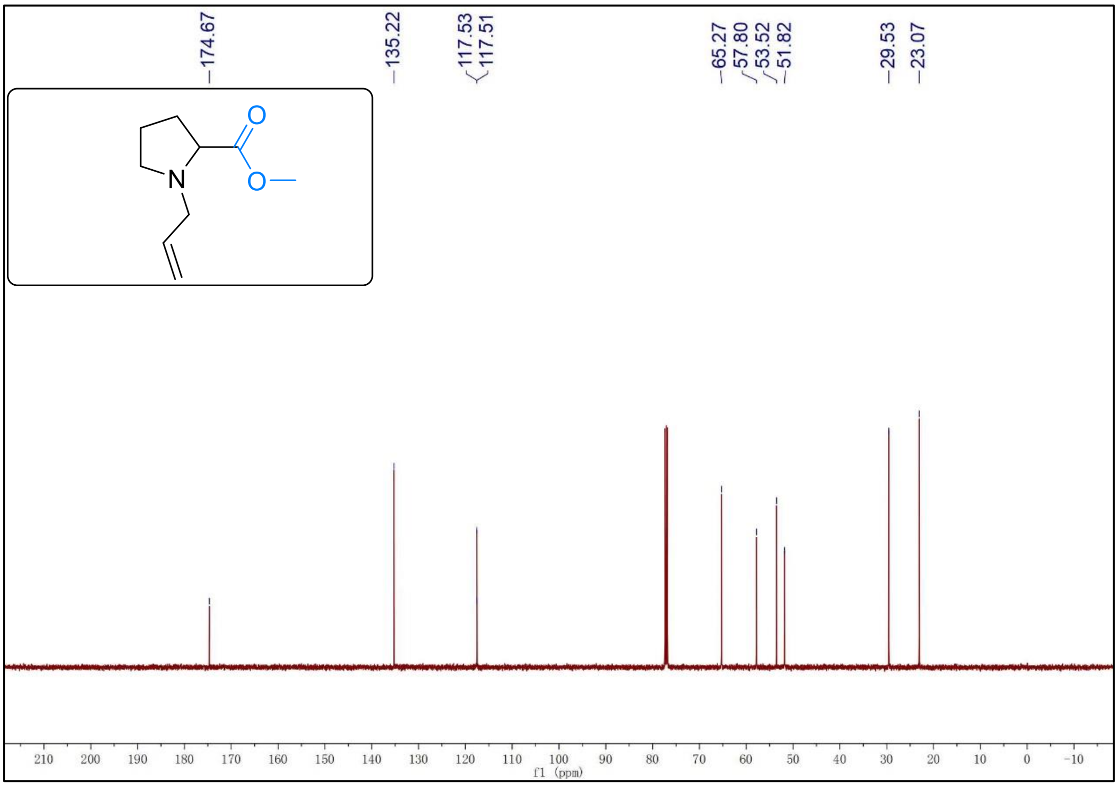

***tert*-butyl allylprolinate**

^{1}H NMR (500 MHz, Chloroform-*d*)

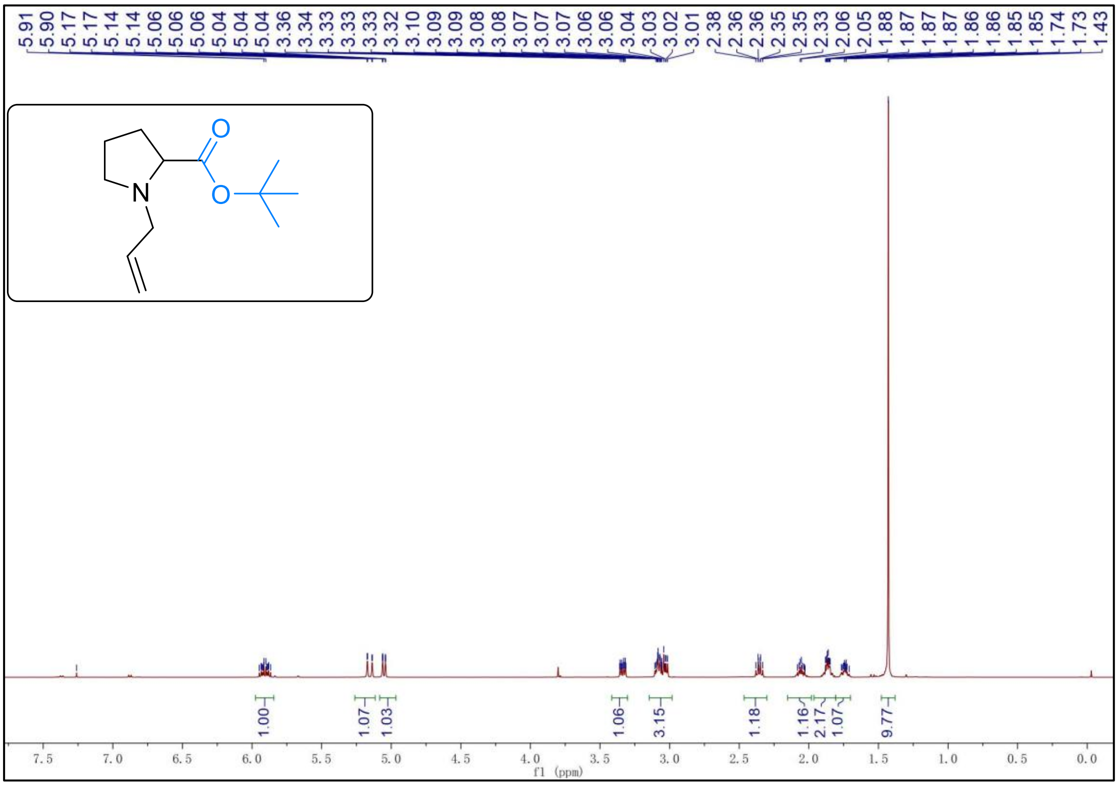

^{13}C NMR (126 MHz, Chloroform-*d*)

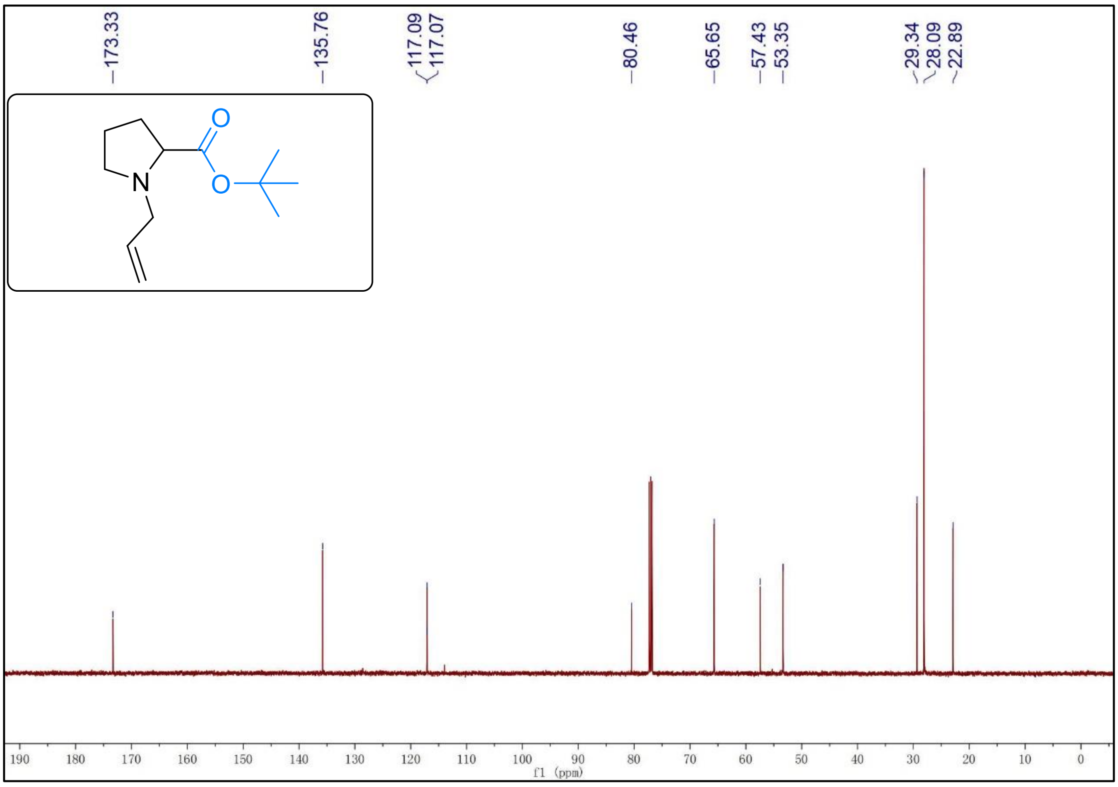

methyl (2-methylallyl)prolinate

^{1}H NMR (500 MHz, Chloroform-*d*)

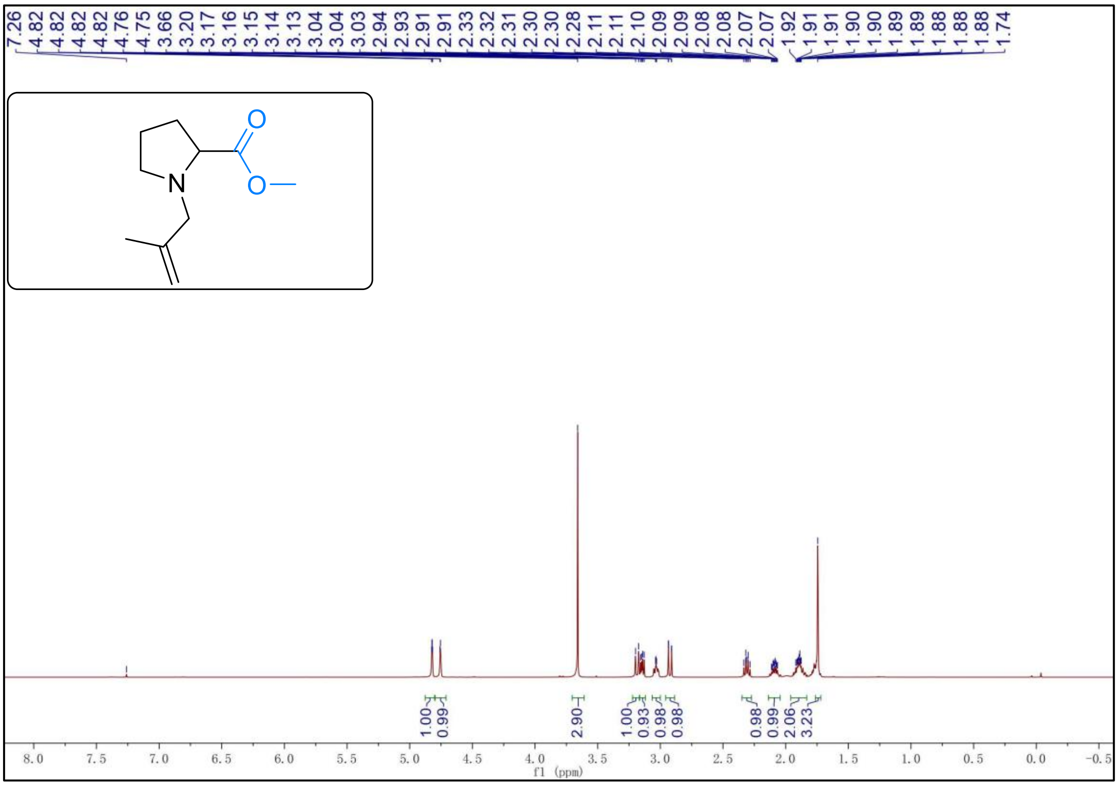

^{13}C NMR (126 MHz, Chloroform-*d*)

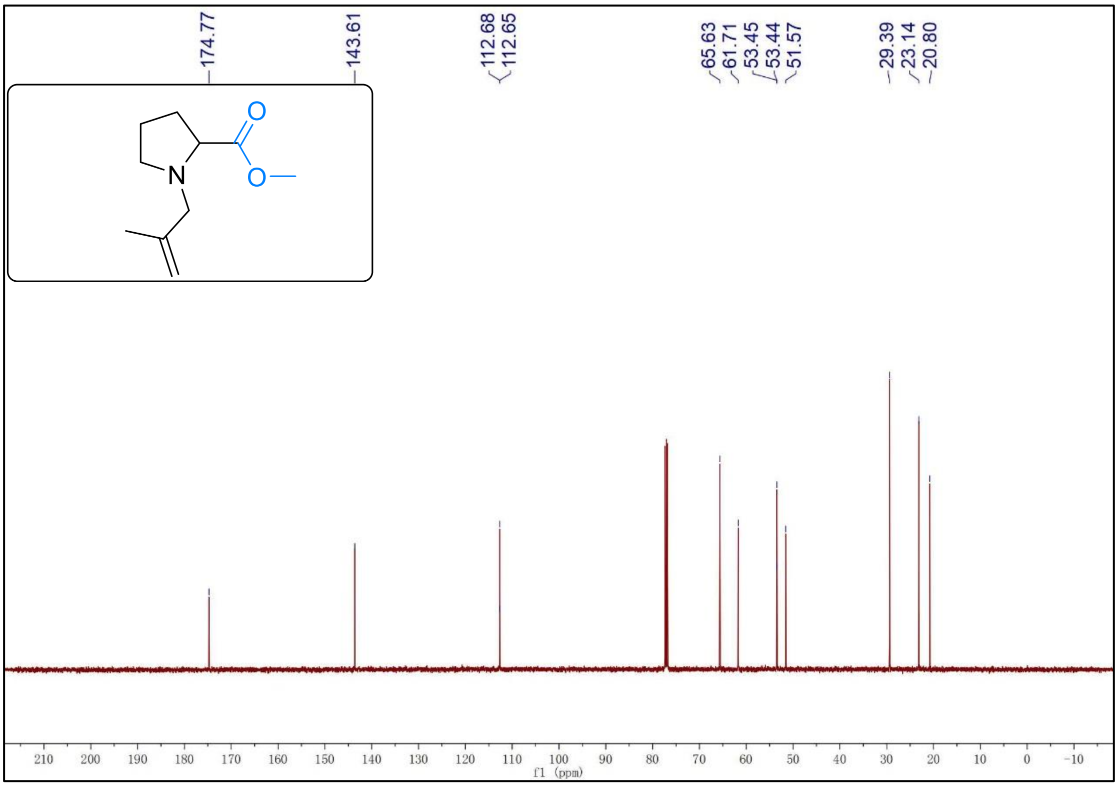

methyl (3-methylbut-2-en-1-yl)prolinate

^{1}H NMR (500 MHz, Chloroform-*d*)

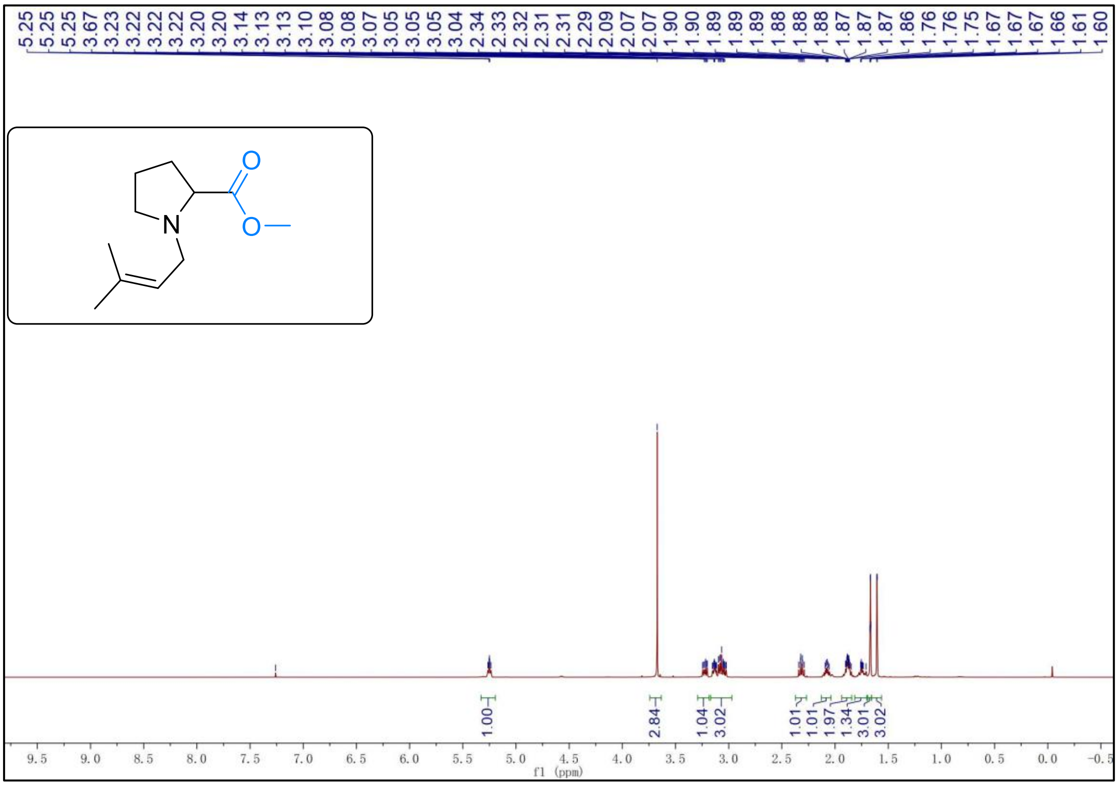

^{13}C NMR (126 MHz, Chloroform-*d*)

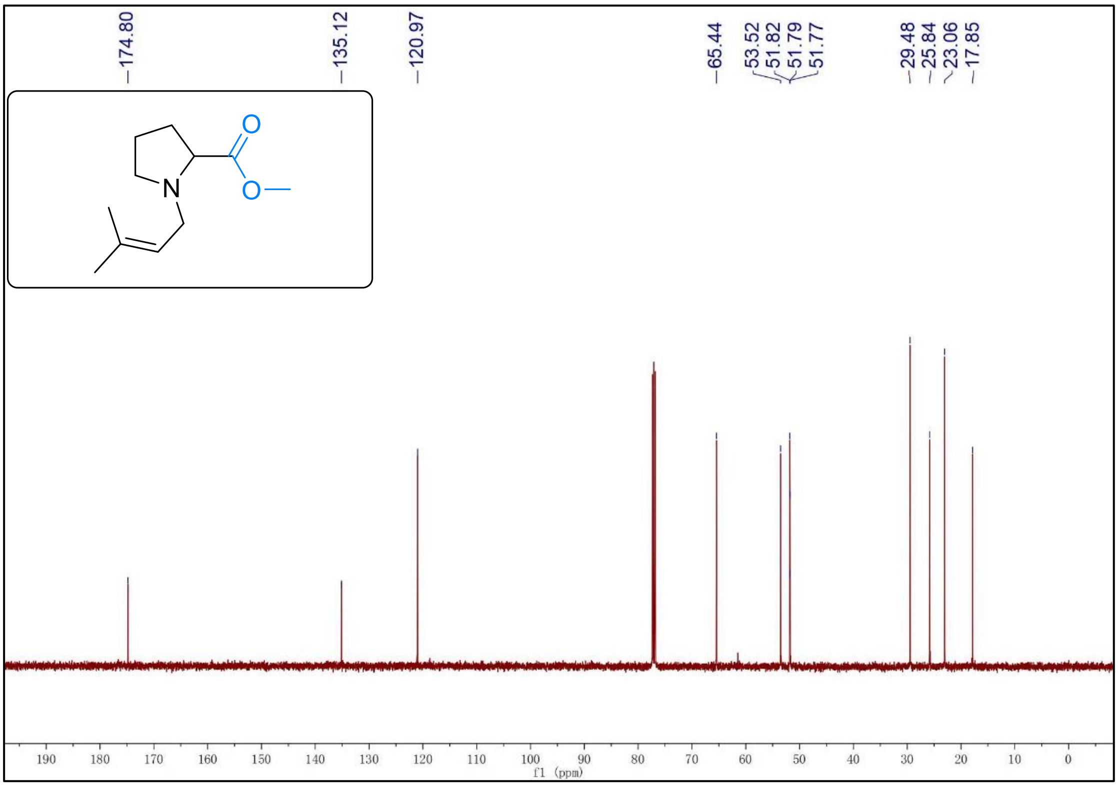

methyl benzylprolinate

^{1}H NMR (500 MHz, Chloroform-*d*)

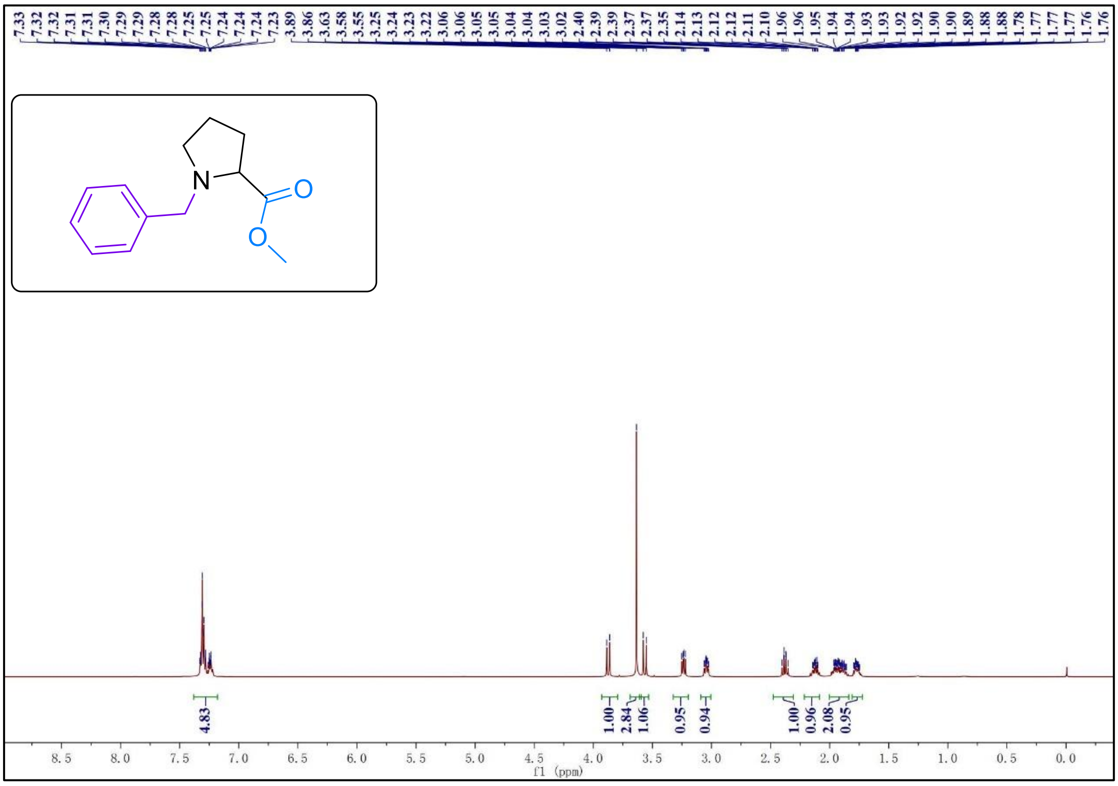

^{13}C NMR (126 MHz, Chloroform-*d*)

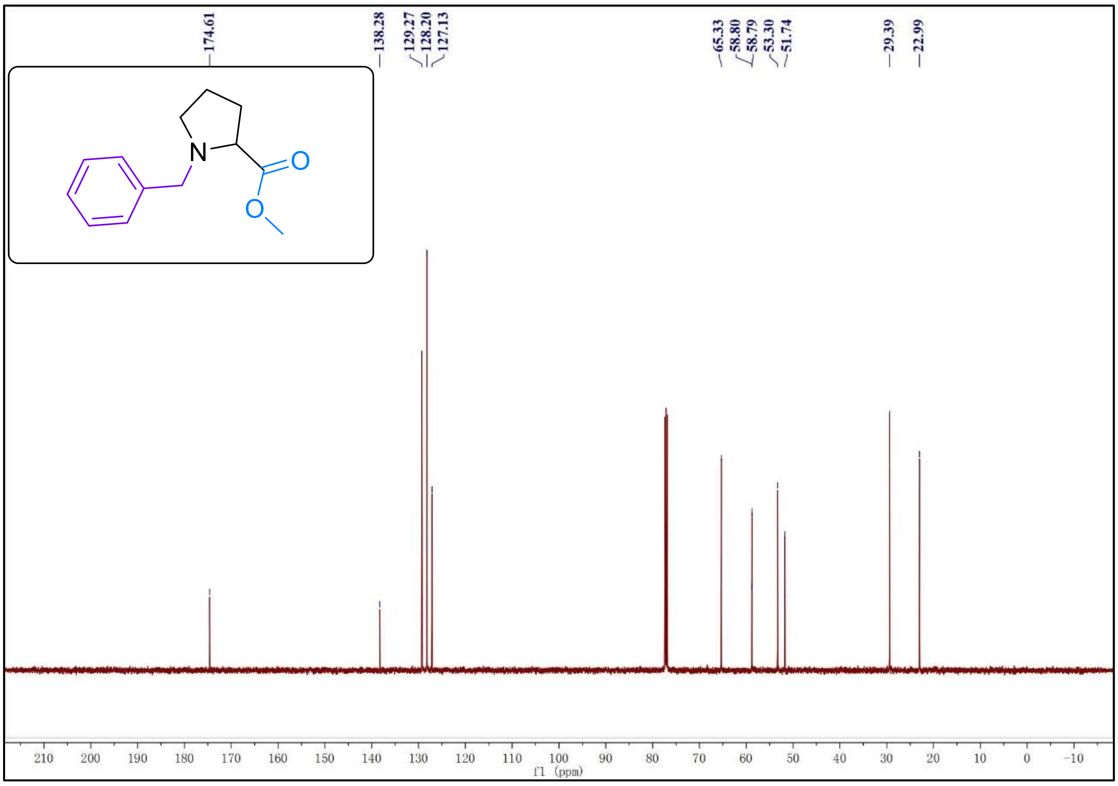

methyl ([1,1'-biphenyl]-4-ylmethyl)prolinate

^{1}H NMR (500 MHz, Chloroform-*d*)

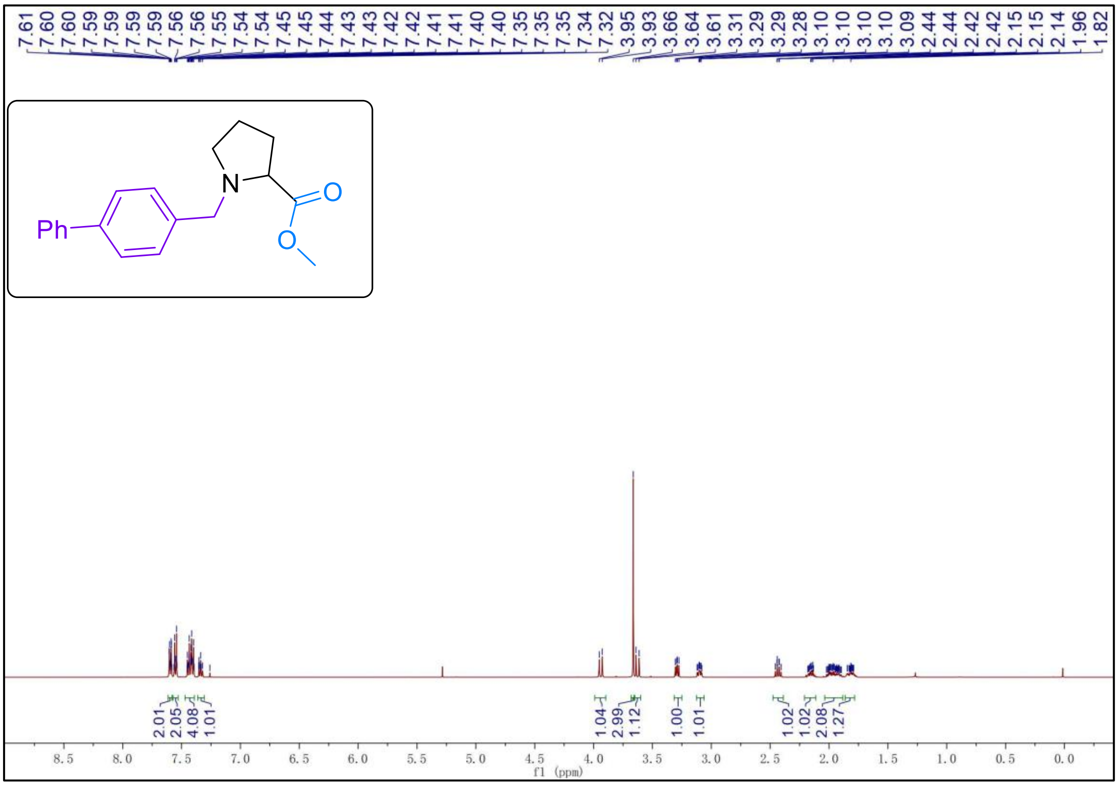

^{13}C NMR (126 MHz, Chloroform-*d*)

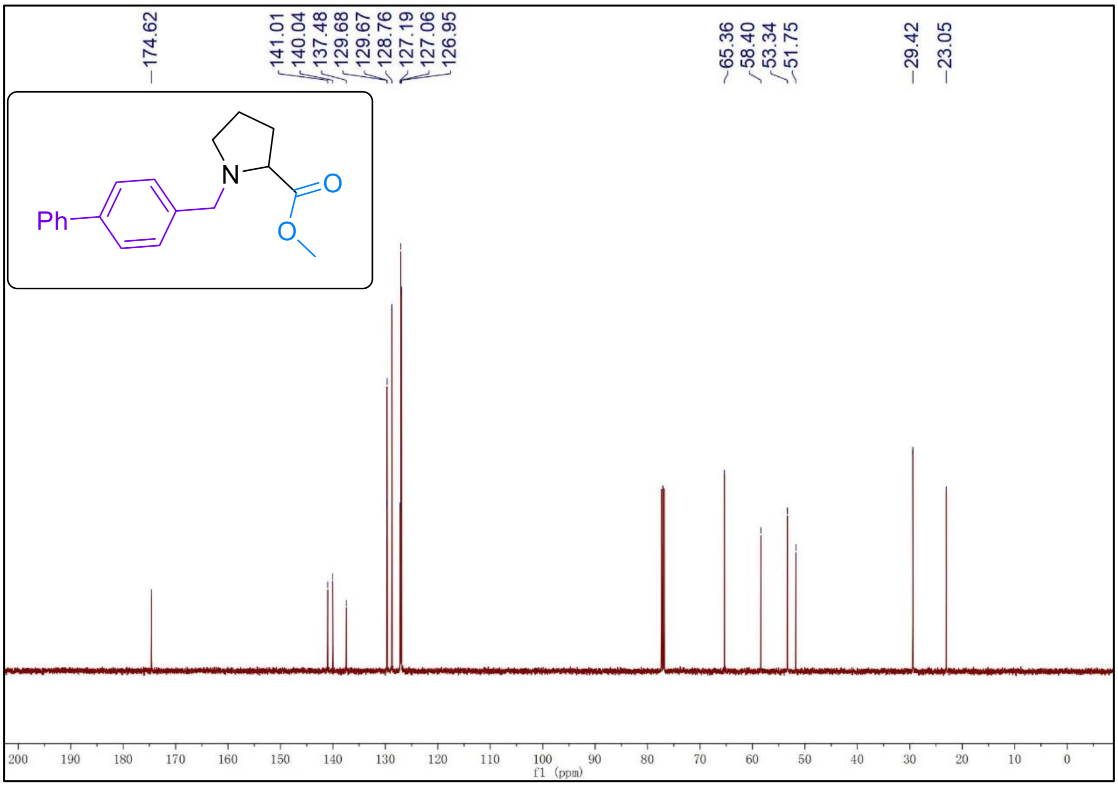

methyl (4-iodobenzyl)prolinate

^{1}H NMR (500 MHz, Chloroform-*d*)

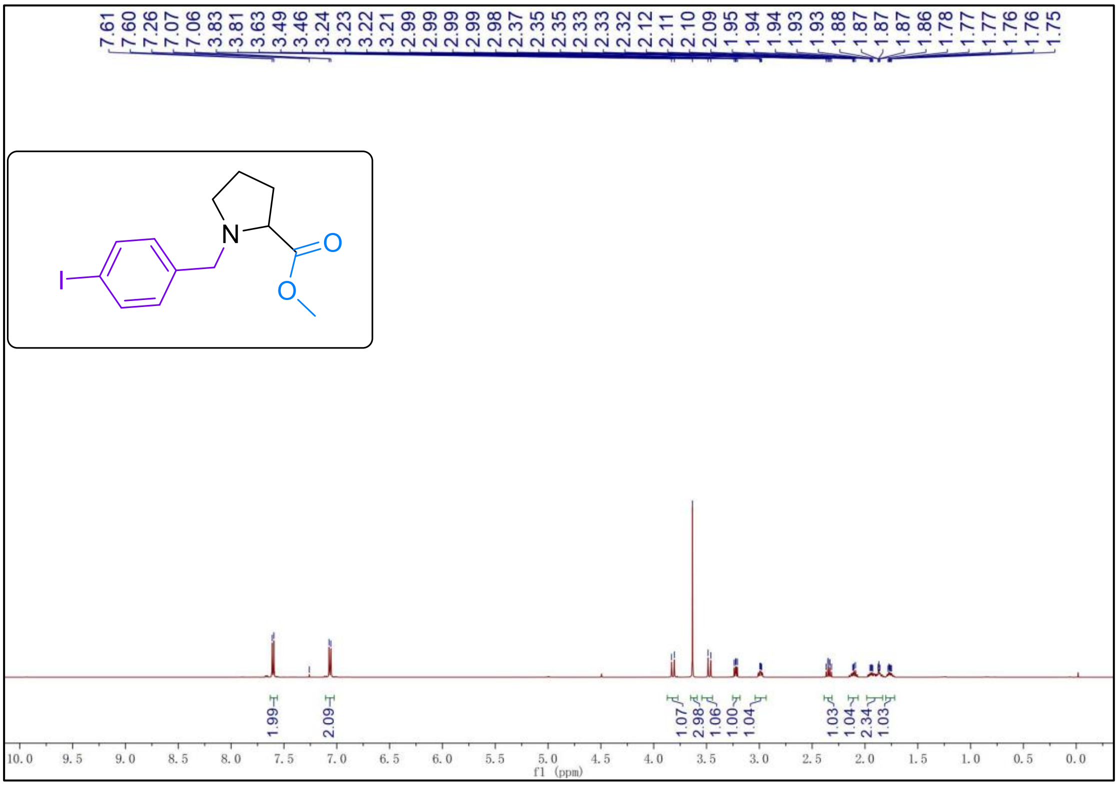

^{13}C NMR (126 MHz, Chloroform-*d*)

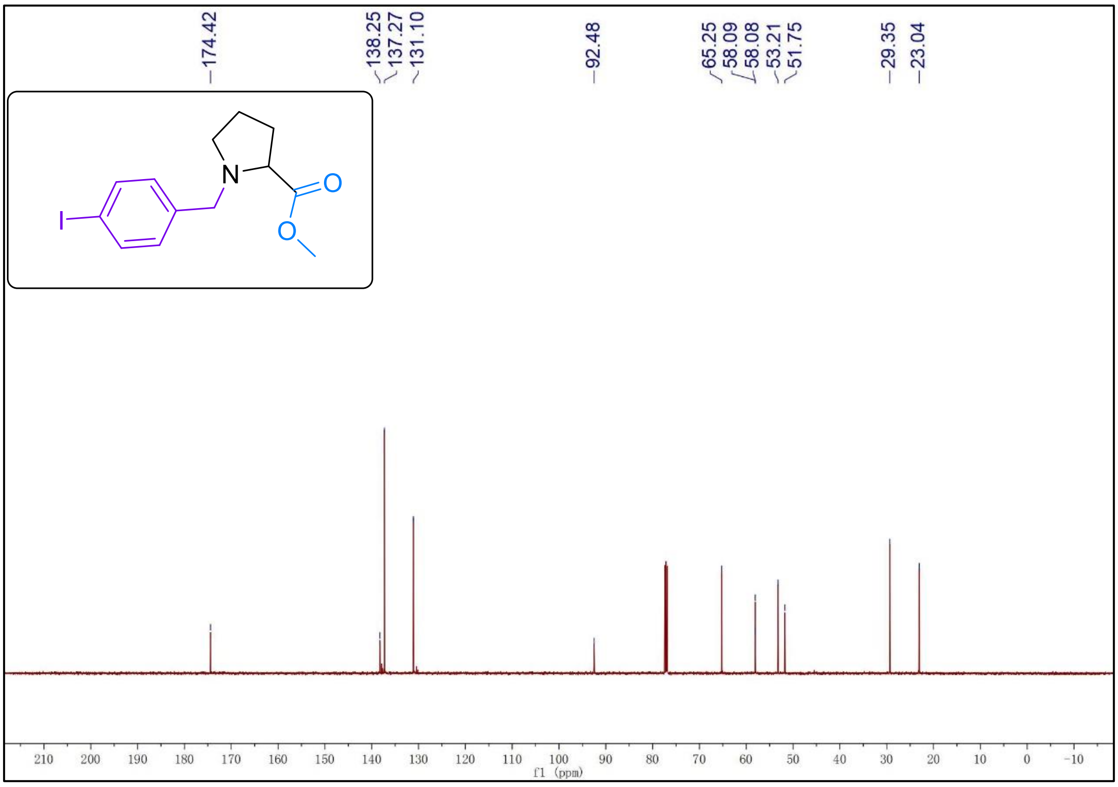

methyl (4-bromobenzyl)prolinate

^{1}H NMR (500 MHz, Chloroform-*d*)

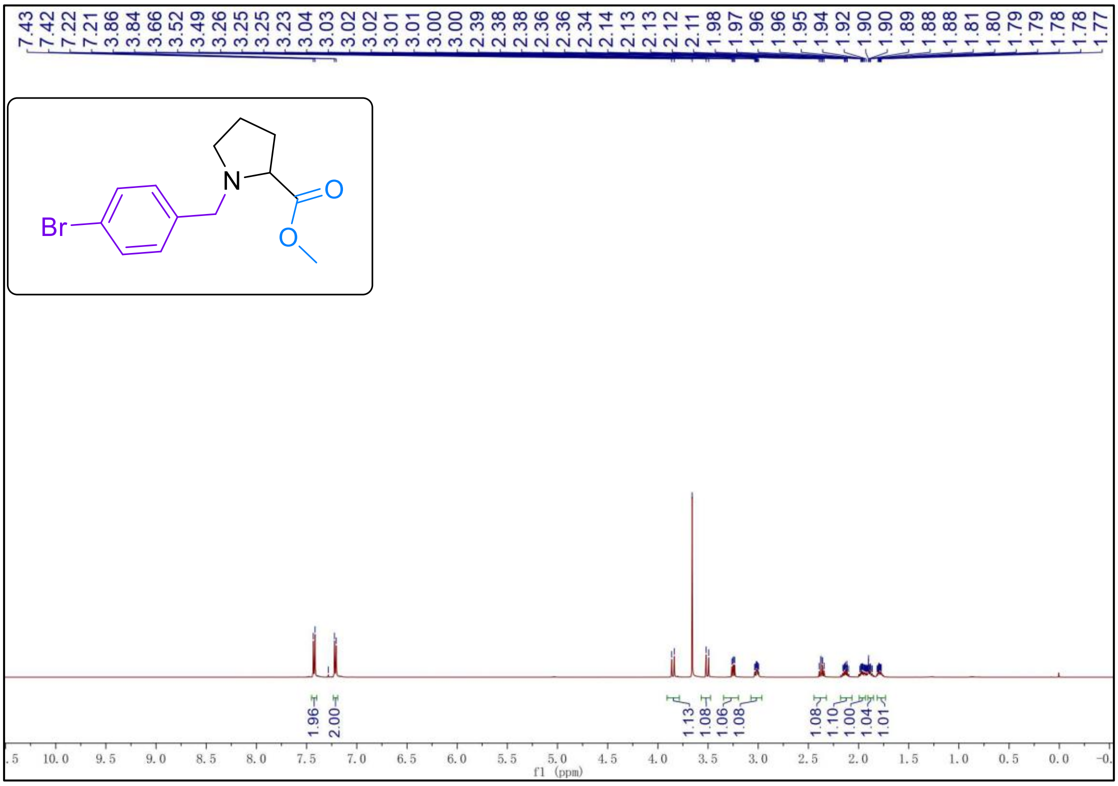

^{13}C NMR (126 MHz, Chloroform-*d*)

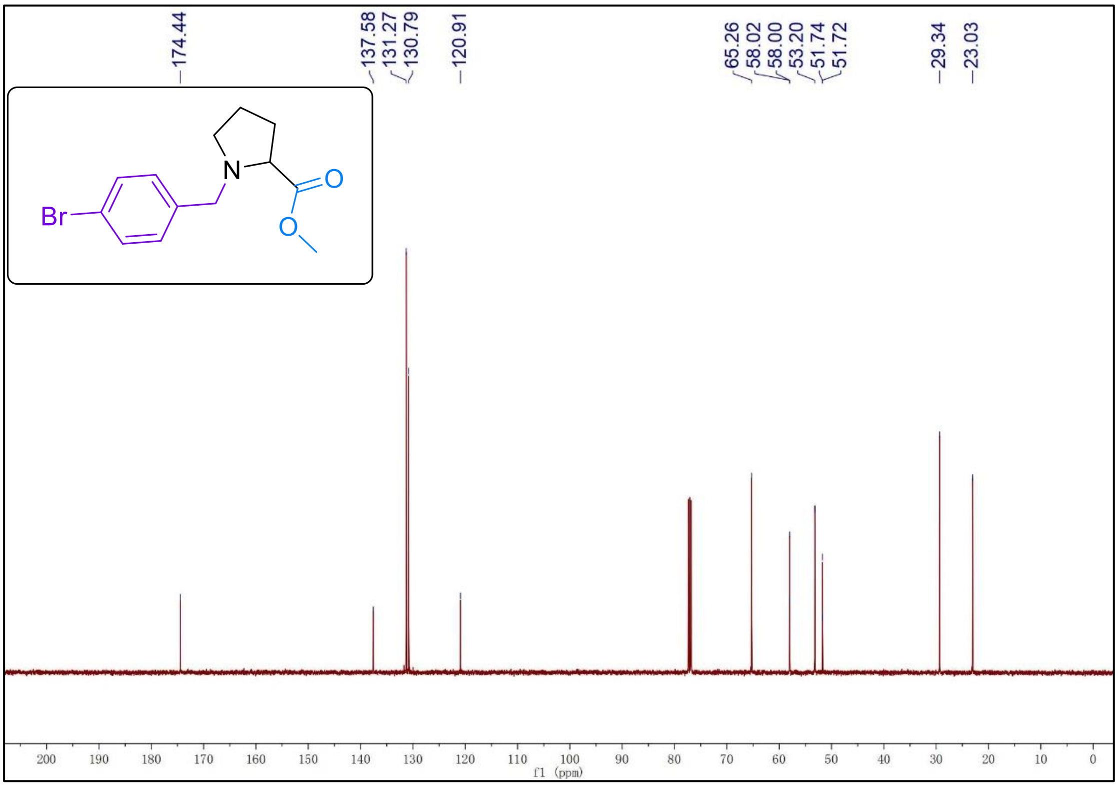

methyl (4-bromo-3-fluorobenzyl)prolinate

^{1}H NMR (500 MHz, Chloroform-*d*)

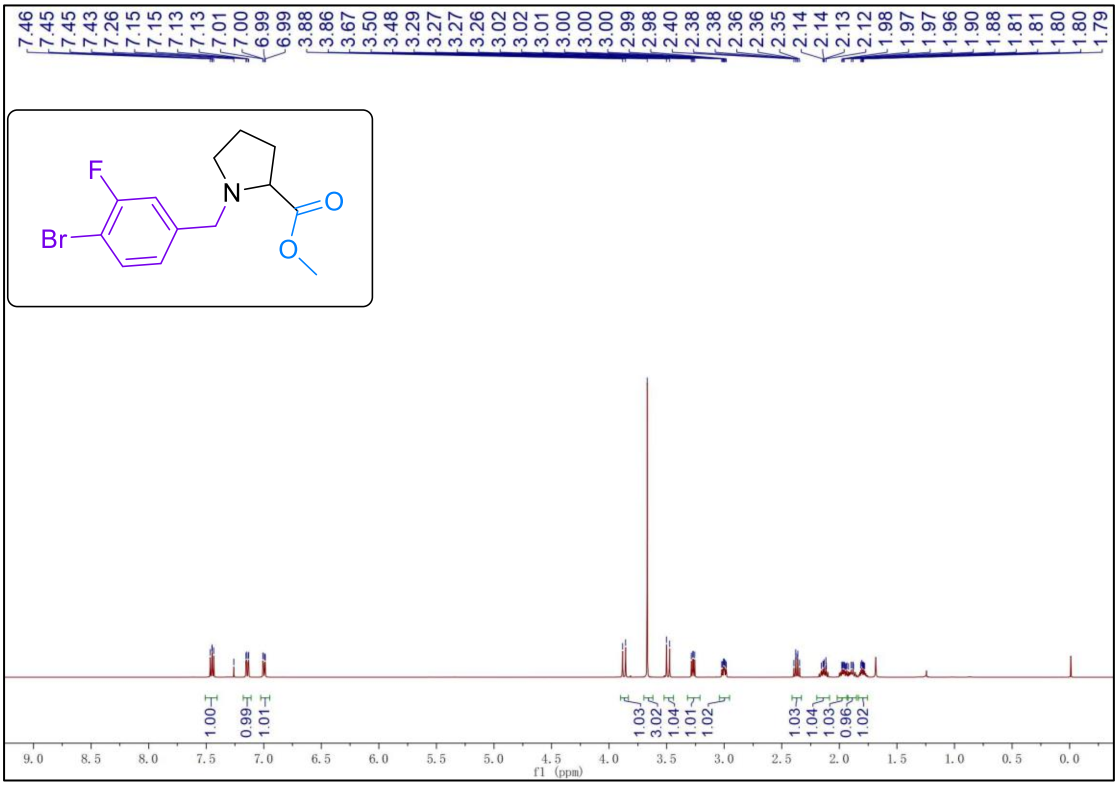

^{13}C NMR (126 MHz, Chloroform-*d*)

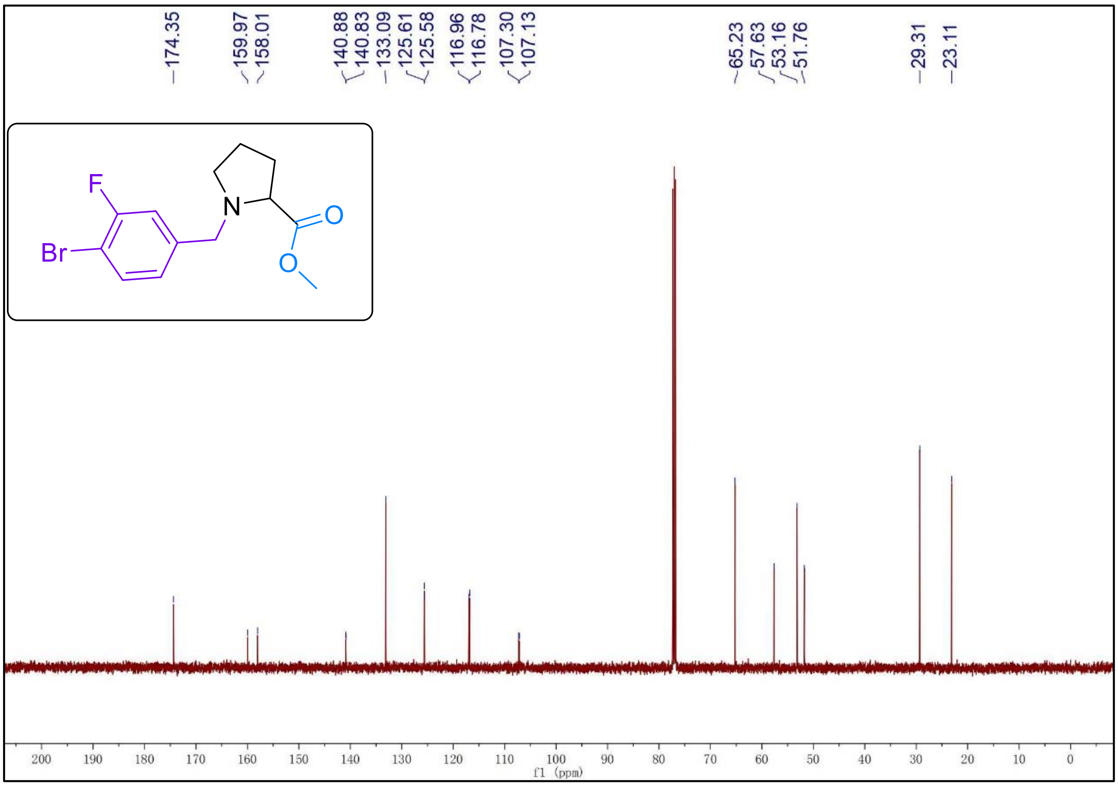

^{19}F NMR (471 MHz, Chloroform-*d*)

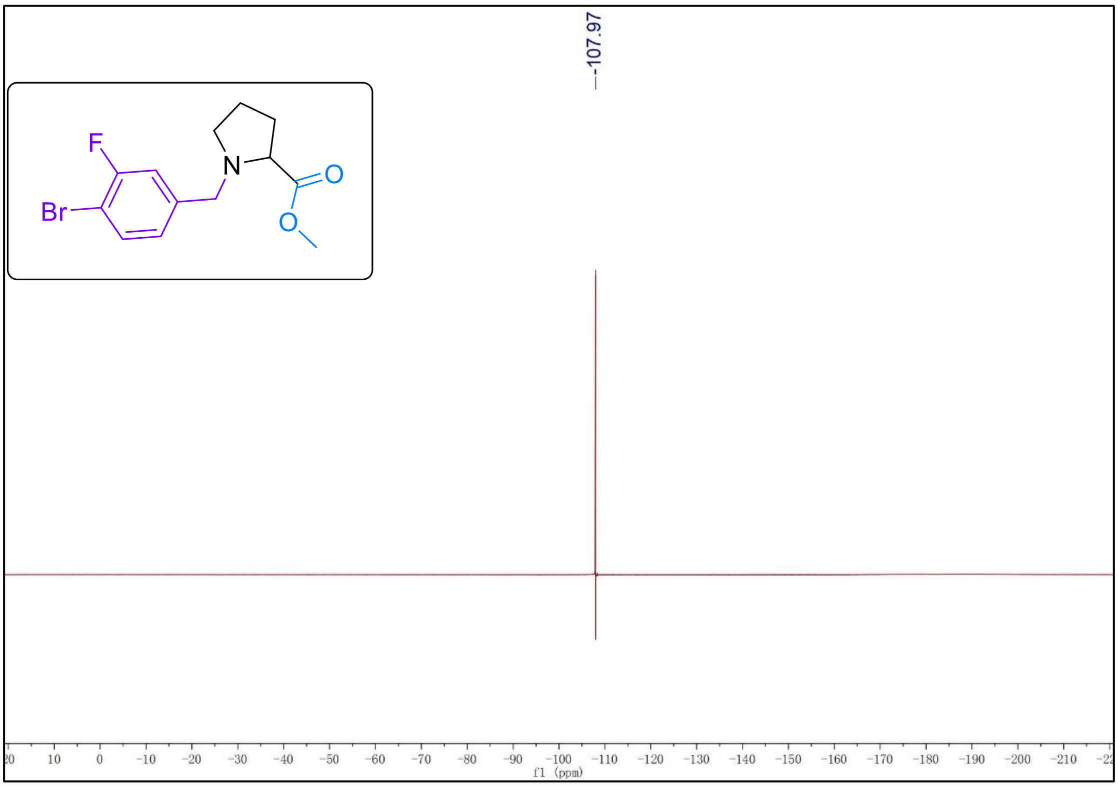

methyl prop-2-yn-1-ylprolinate

^{1}H NMR (500 MHz, Chloroform-*d*)

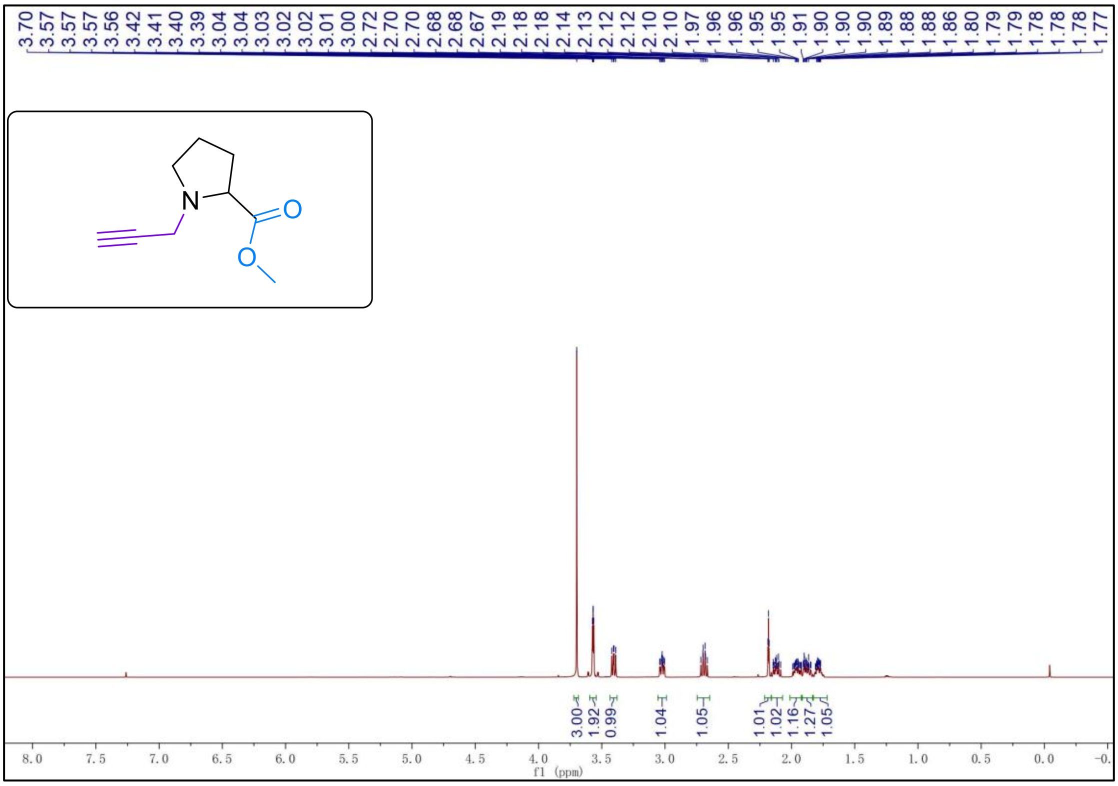

^{13}C NMR (126 MHz, Chloroform-*d*)

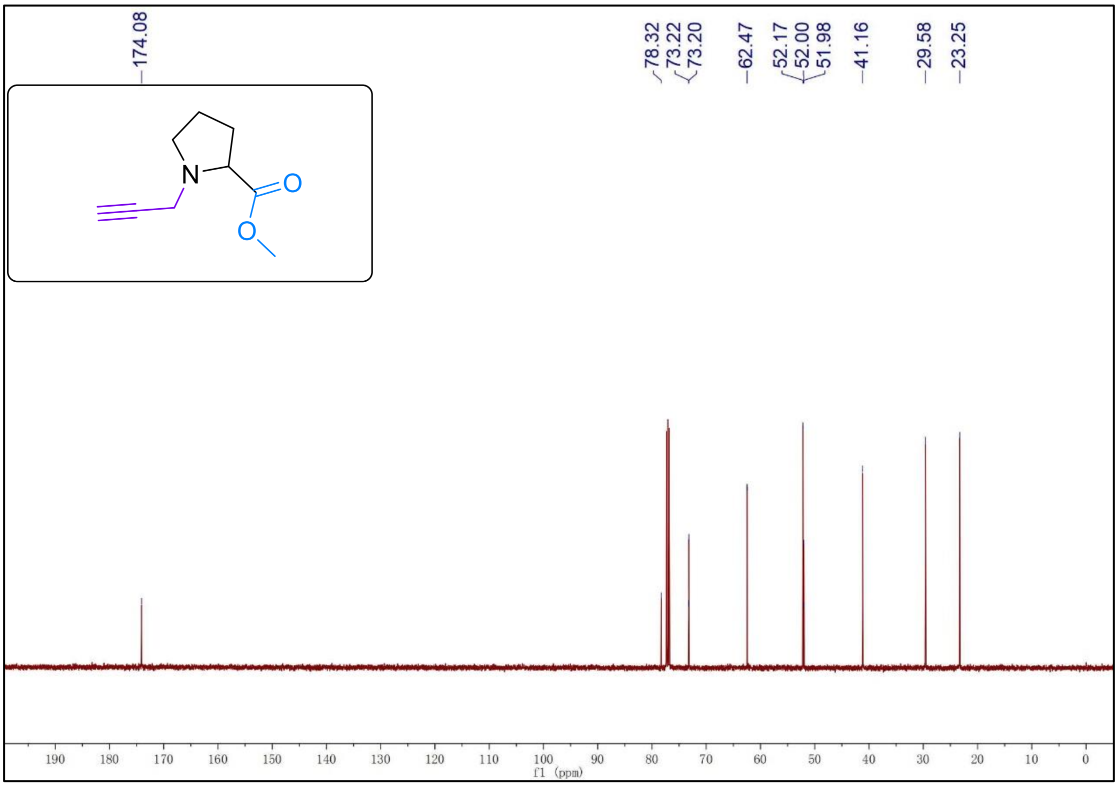

tert-butyl prop-2-yn-1-ylprolinate

^{1}H NMR (500 MHz, Chloroform-*d*)

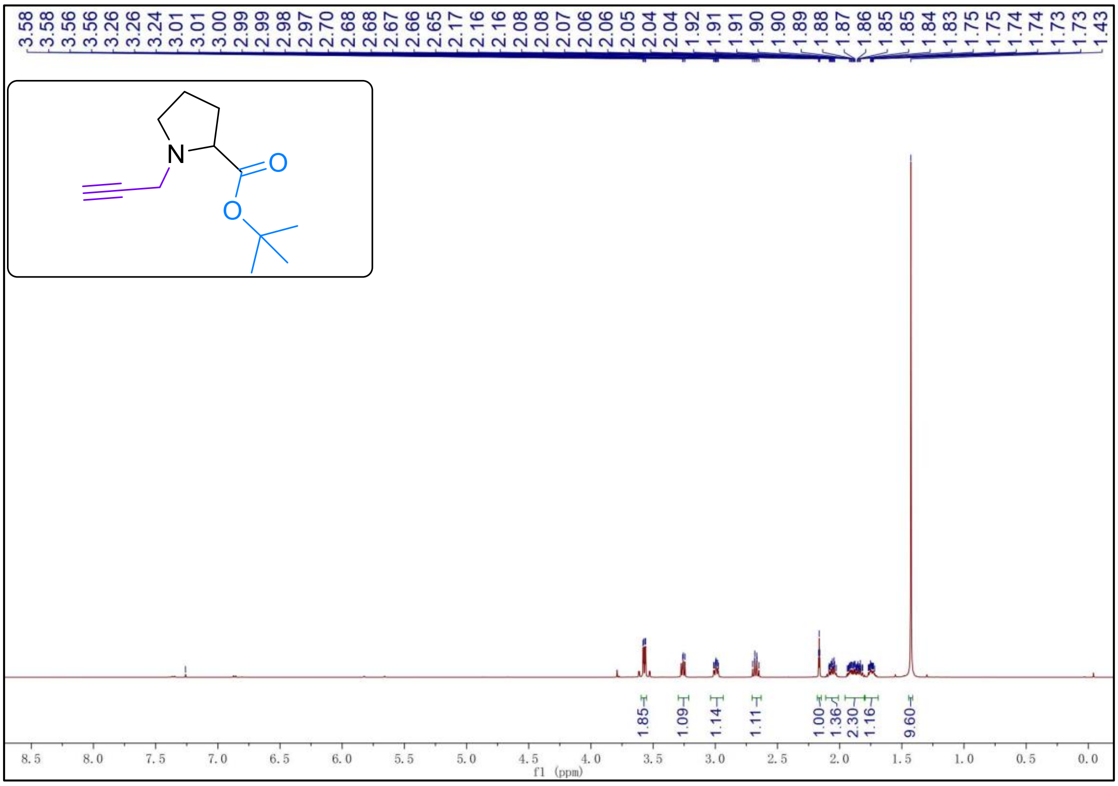

^{13}C NMR (126 MHz, Chloroform-*d*)

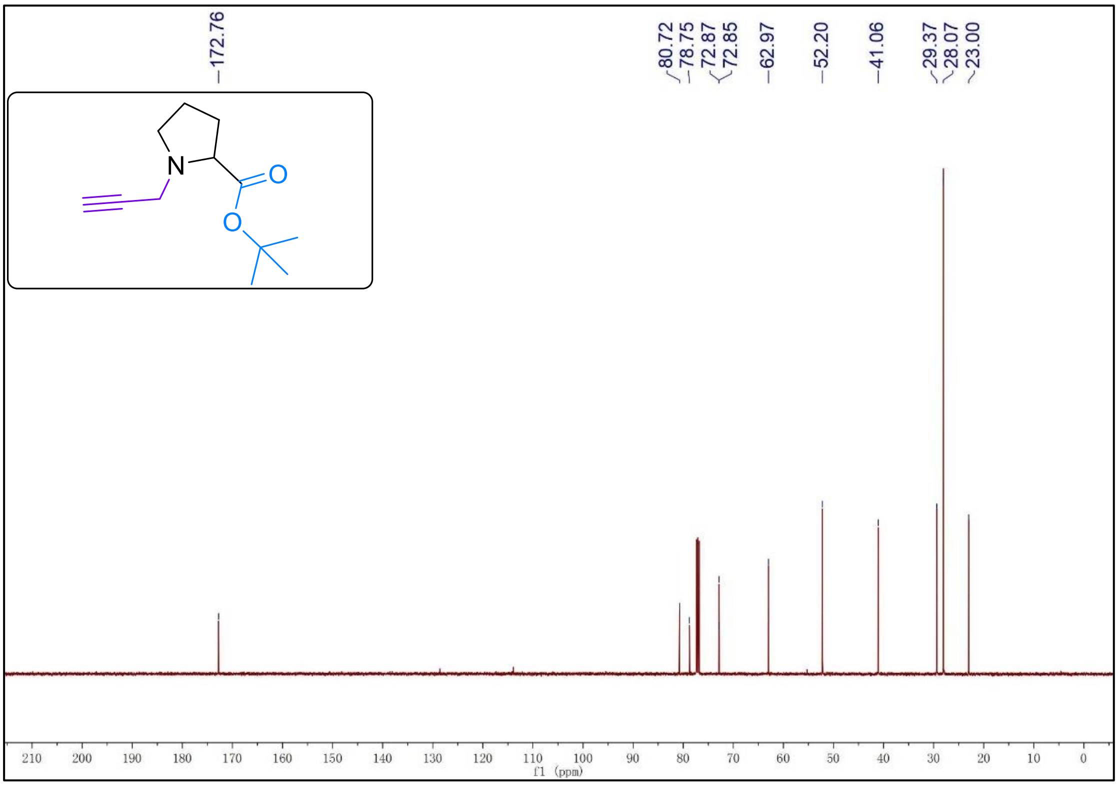

methyl but-2-yn-1-ylprolinate

^{1}H NMR (500 MHz, Chloroform-*d*)

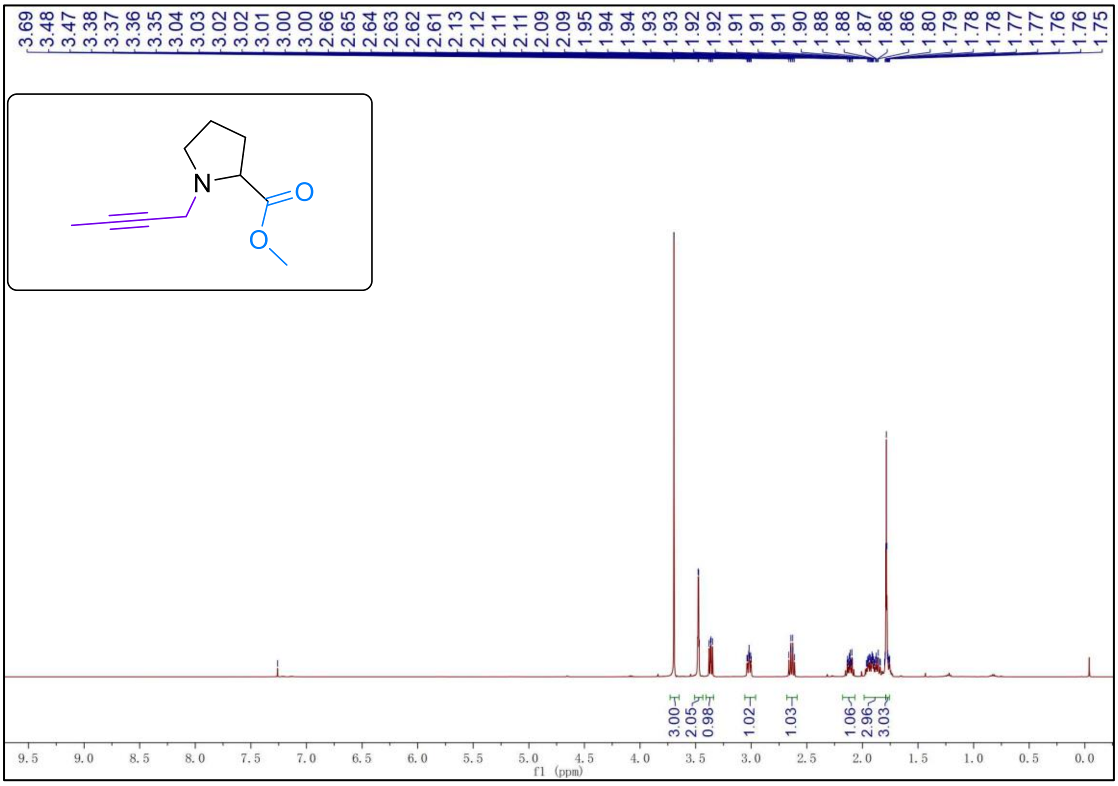

^{13}C NMR (126 MHz, Chloroform-*d*)

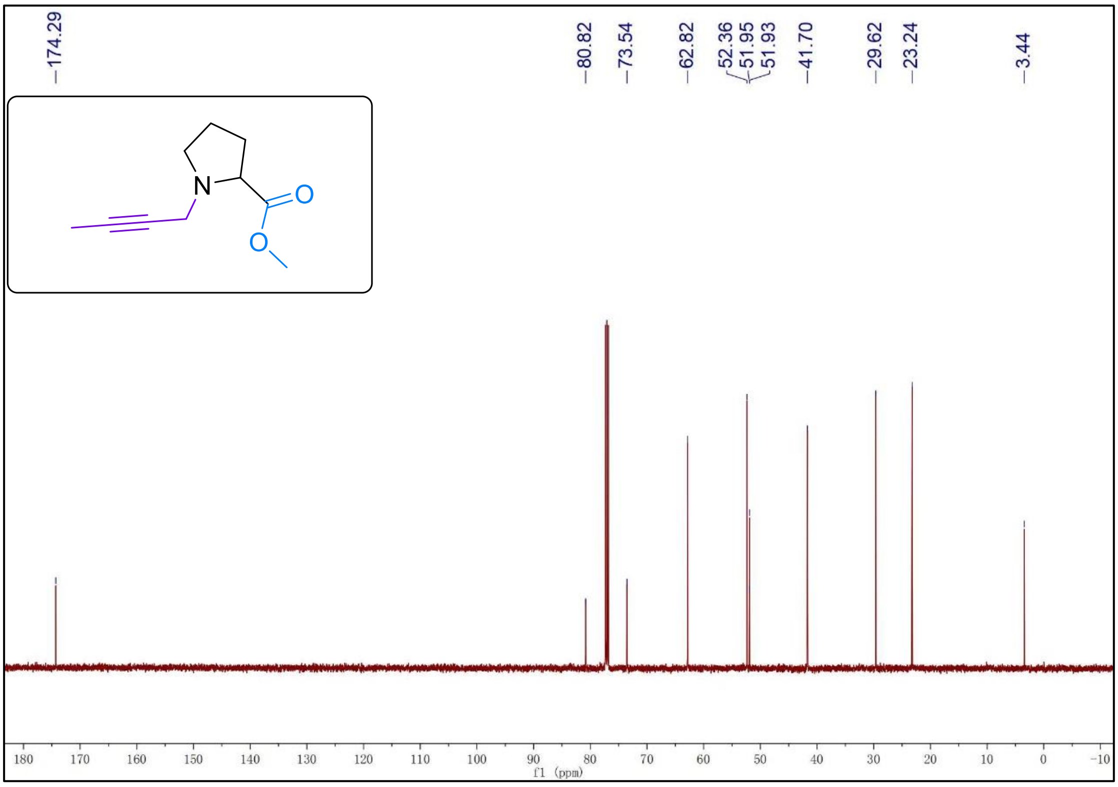

methyl pent-2-yn-1-ylprolinate

^{1}H NMR (500 MHz, Chloroform-*d*)

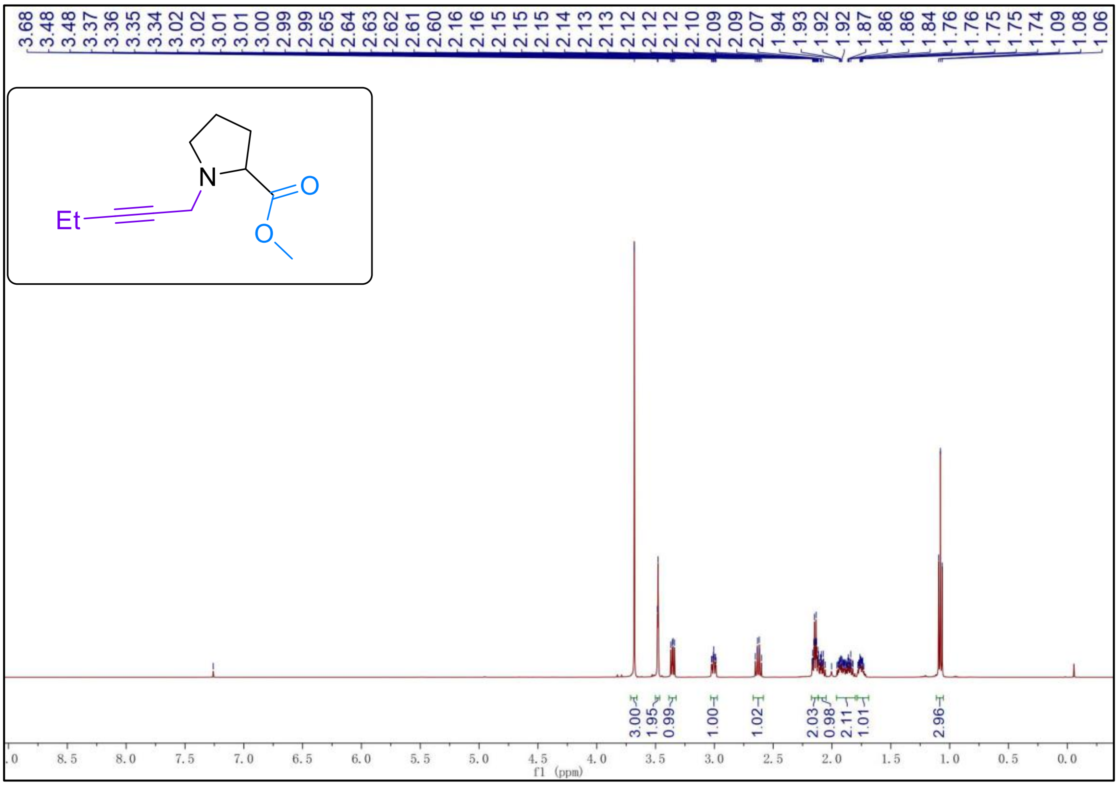

^{13}C NMR (126 MHz, Chloroform-*d*)

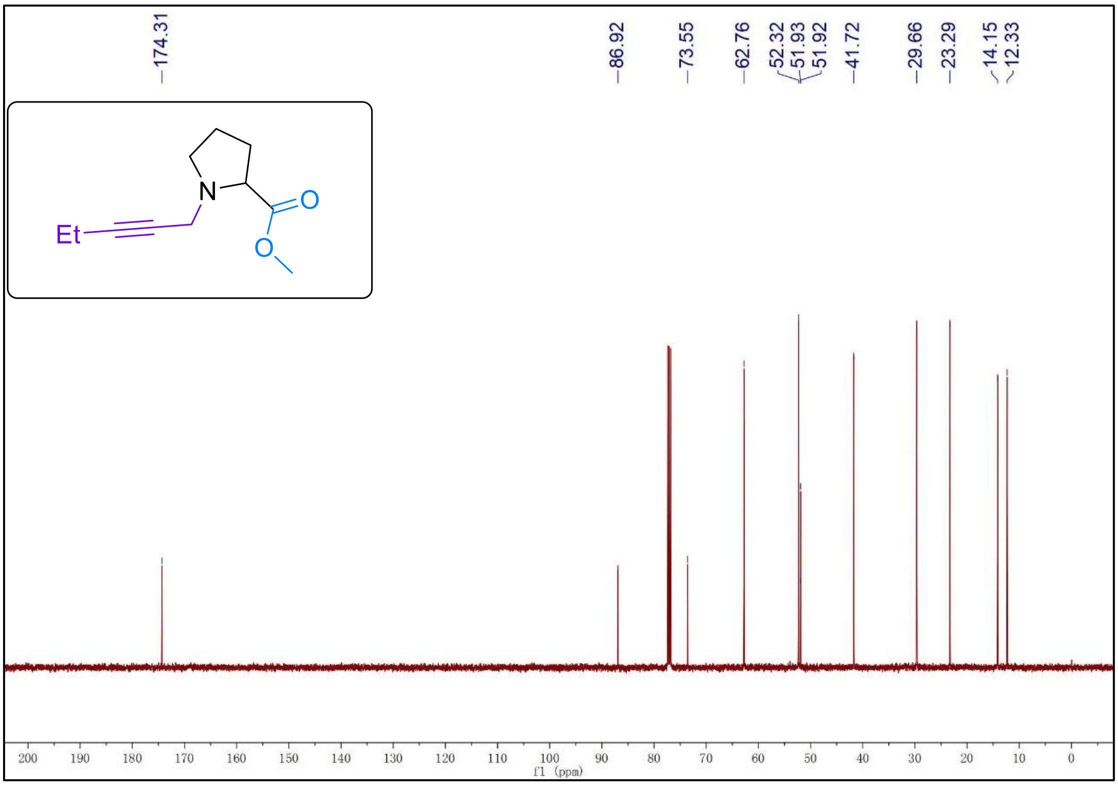

methyl (3-cyclopropylprop-2-yn-1-yl)prolinate

^{1}H NMR (500 MHz, Chloroform-*d*)

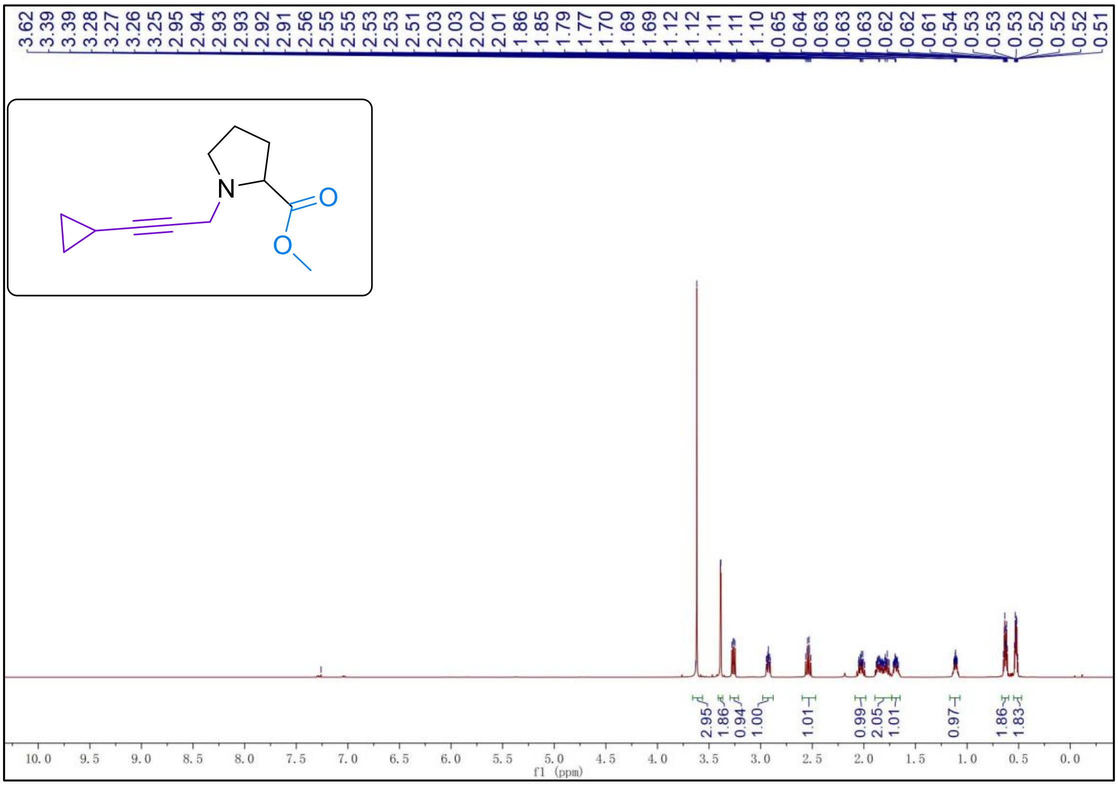

^{13}C NMR (126 MHz, Chloroform-*d*)

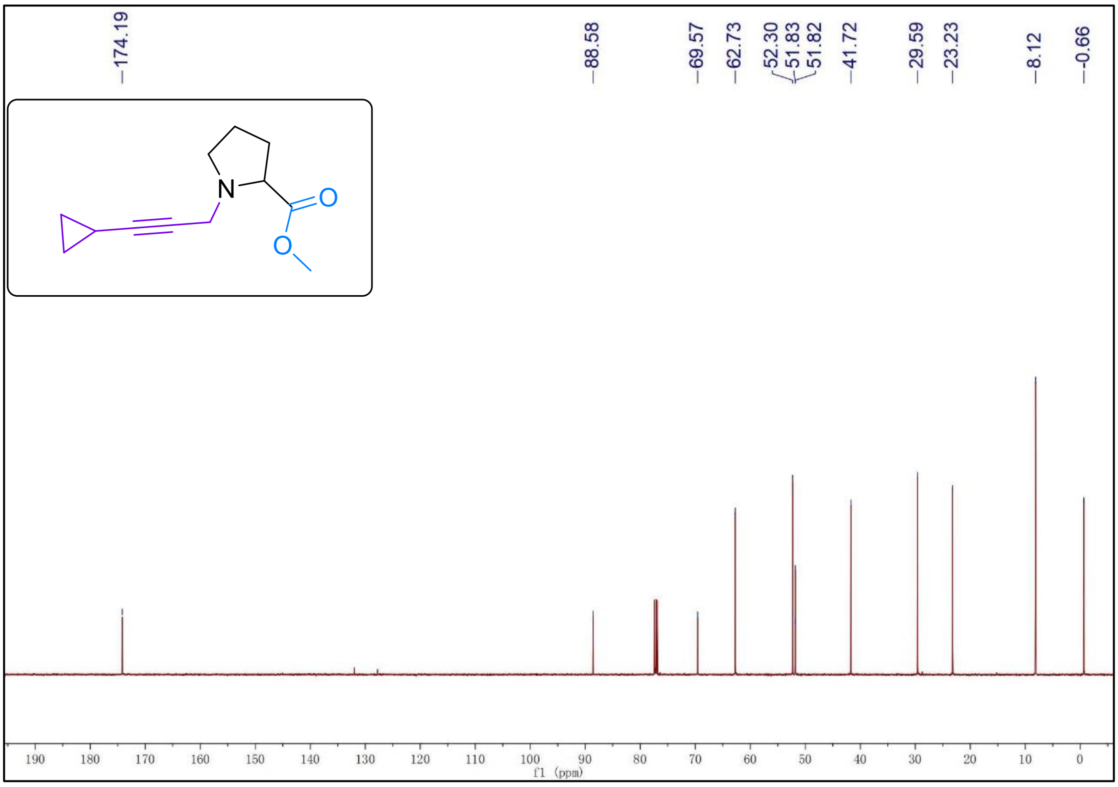

methyl hept-2-yn-1-ylprolinate

^{1}H NMR (500 MHz, Chloroform-*d*)

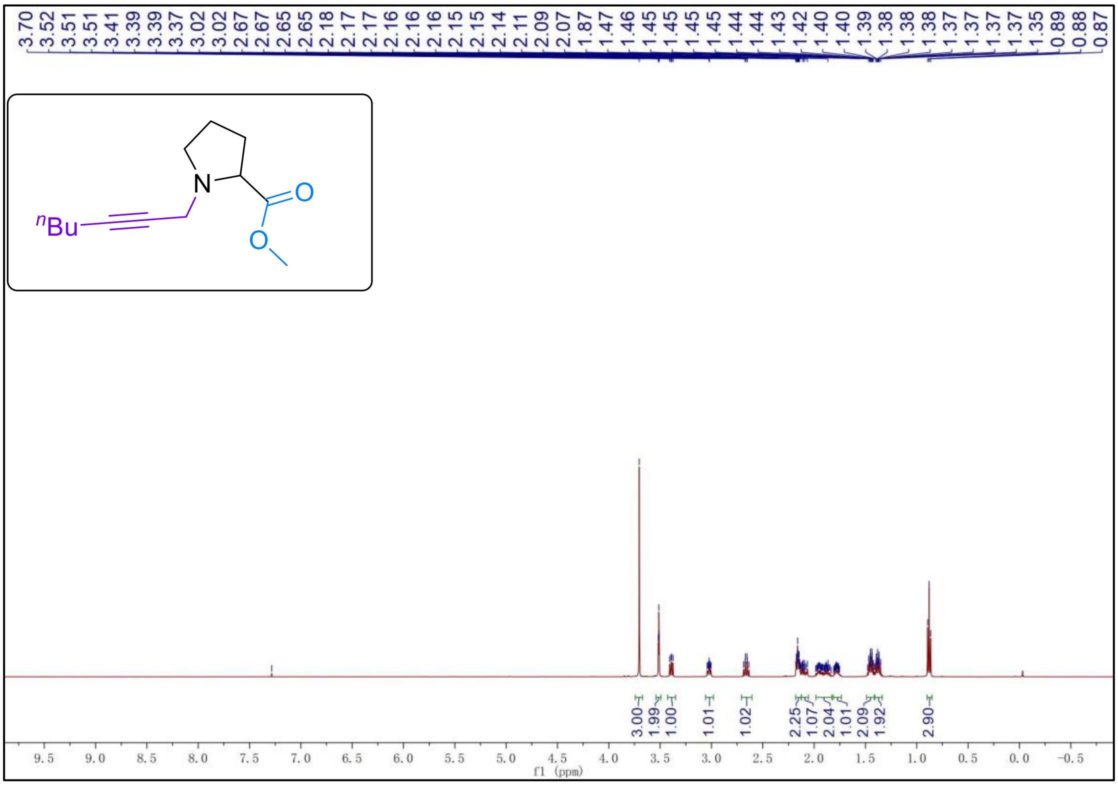

^{13}C NMR (126 MHz, Chloroform-*d*)

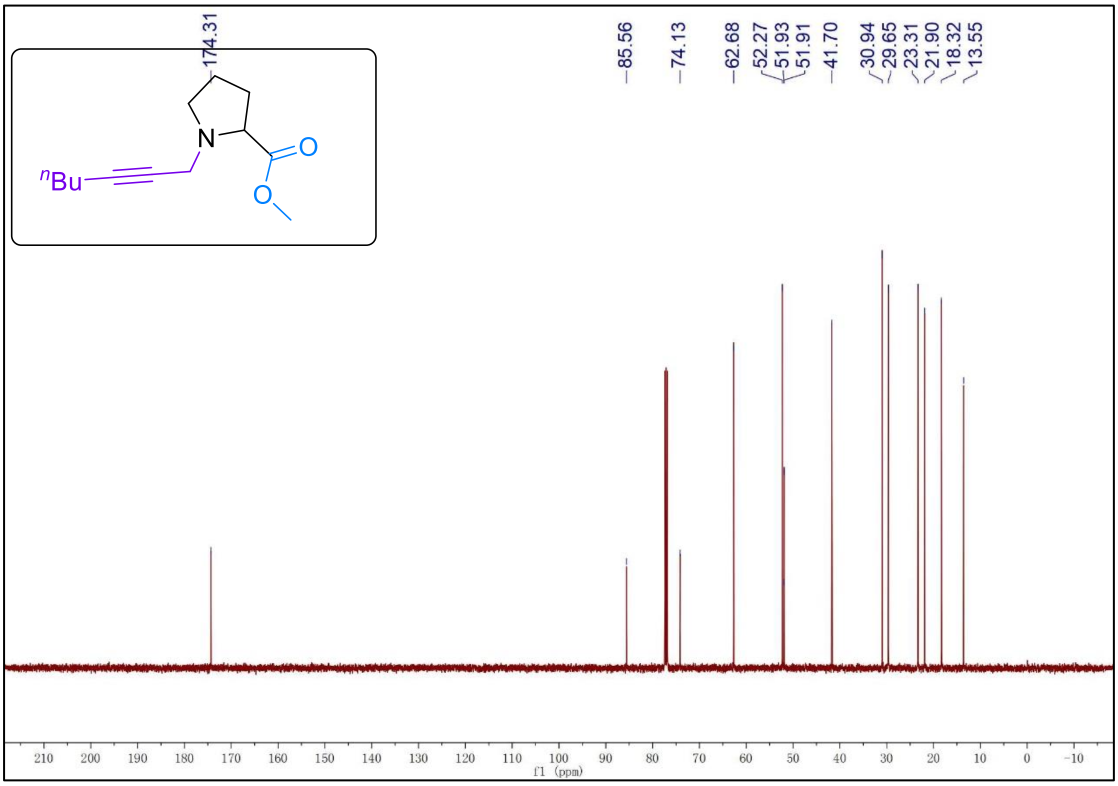

methyl (4,4-dimethylpent-2-yn-1-yl)prolinate

^{1}H NMR (500 MHz, Chloroform-*d*)

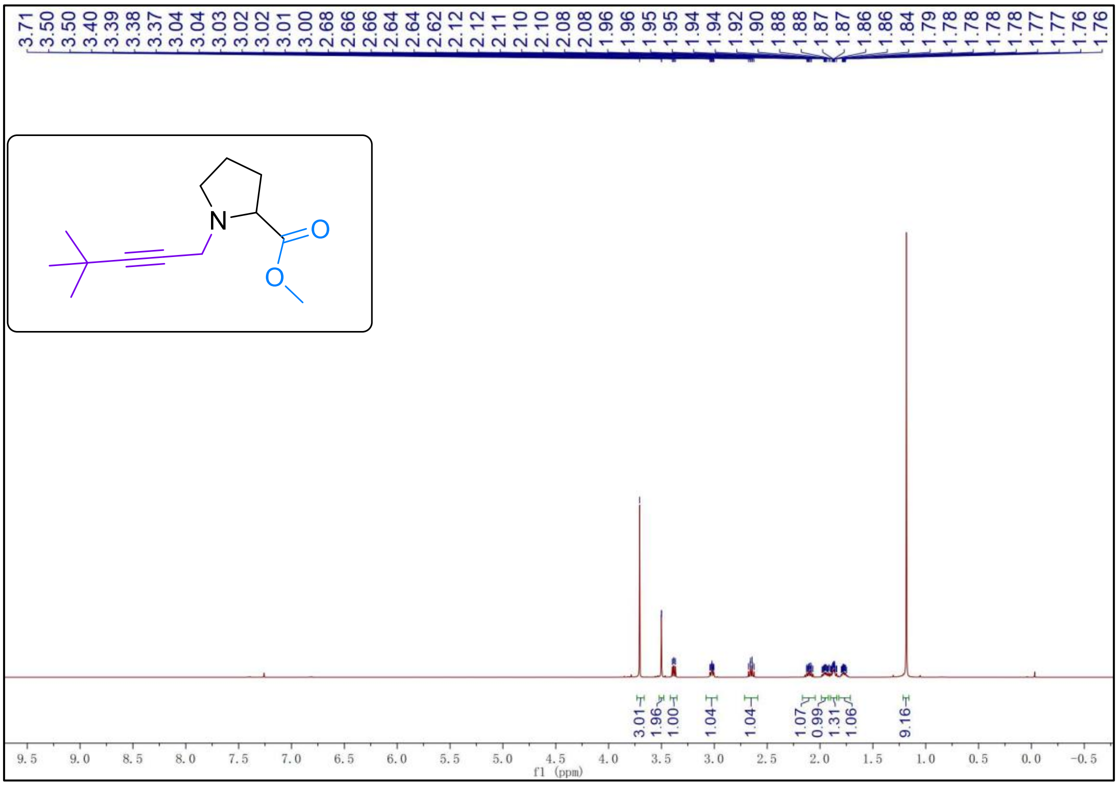

^{13}C NMR (126 MHz, Chloroform-*d*)

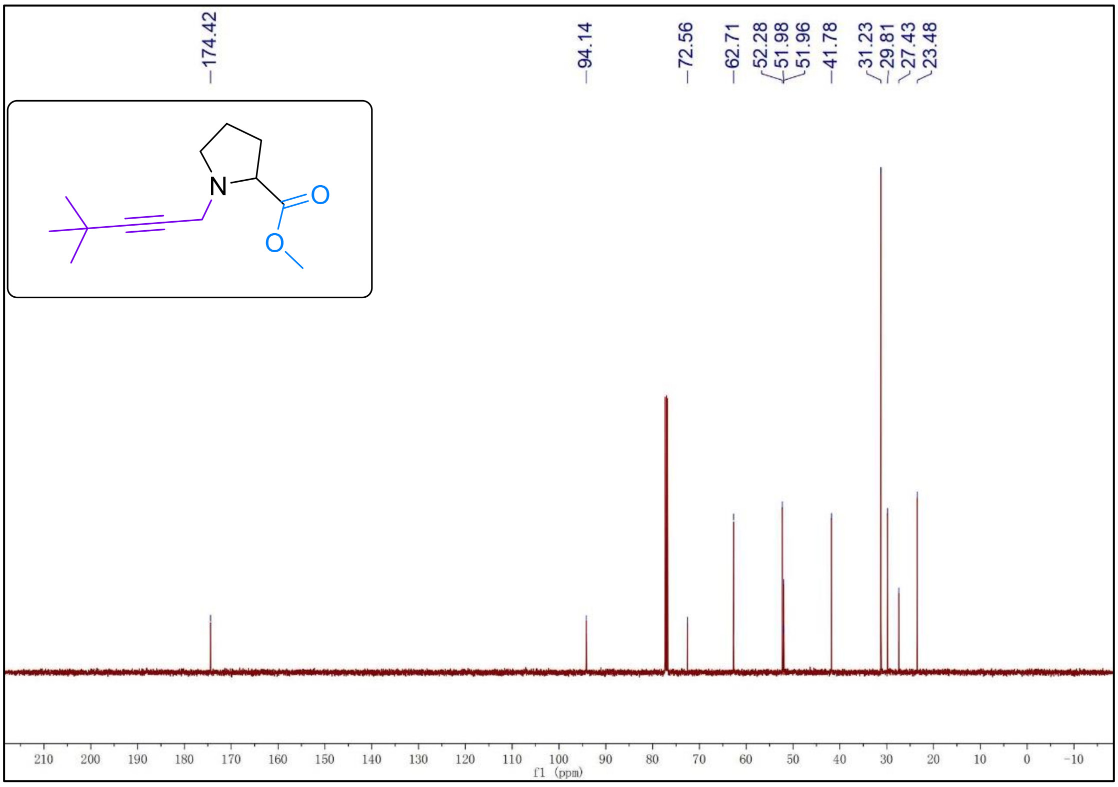

methyl (4-((tetrahydro-2H-pyran-2-yl)oxy)but-2-yn-1-yl)prolinate

^{1}H NMR (500 MHz, Chloroform-*d*)

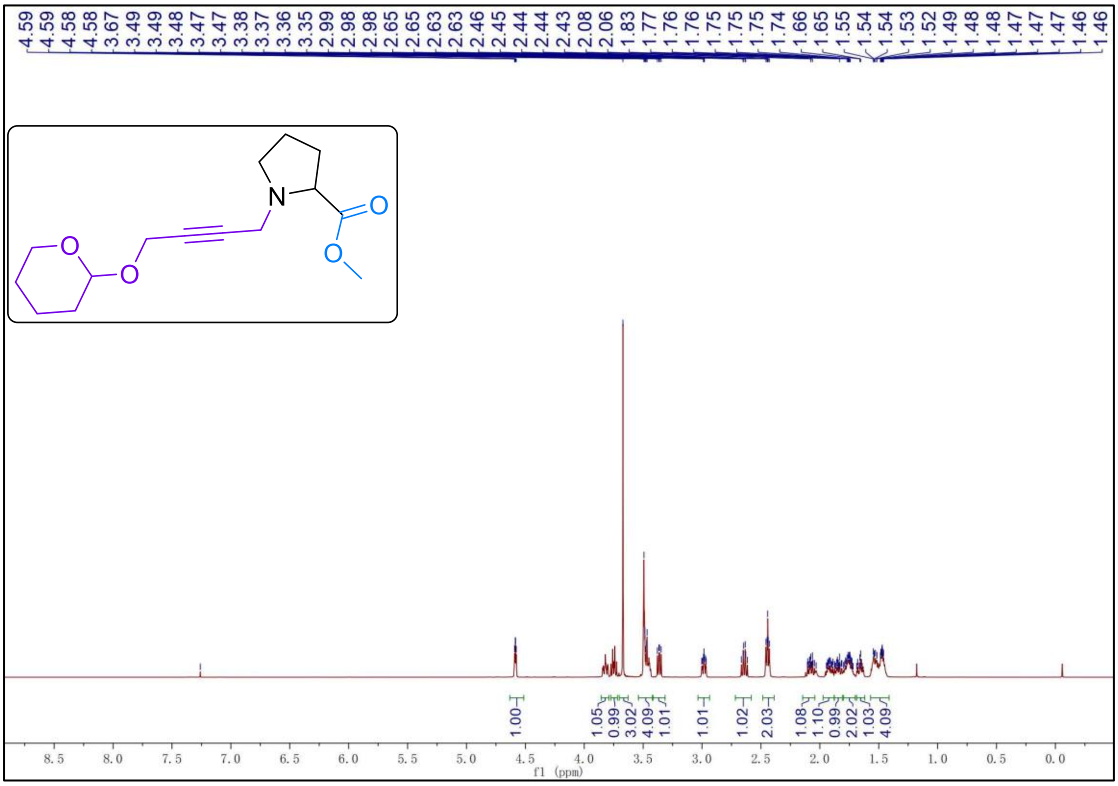

^{13}C NMR (126 MHz, Chloroform-*d*)

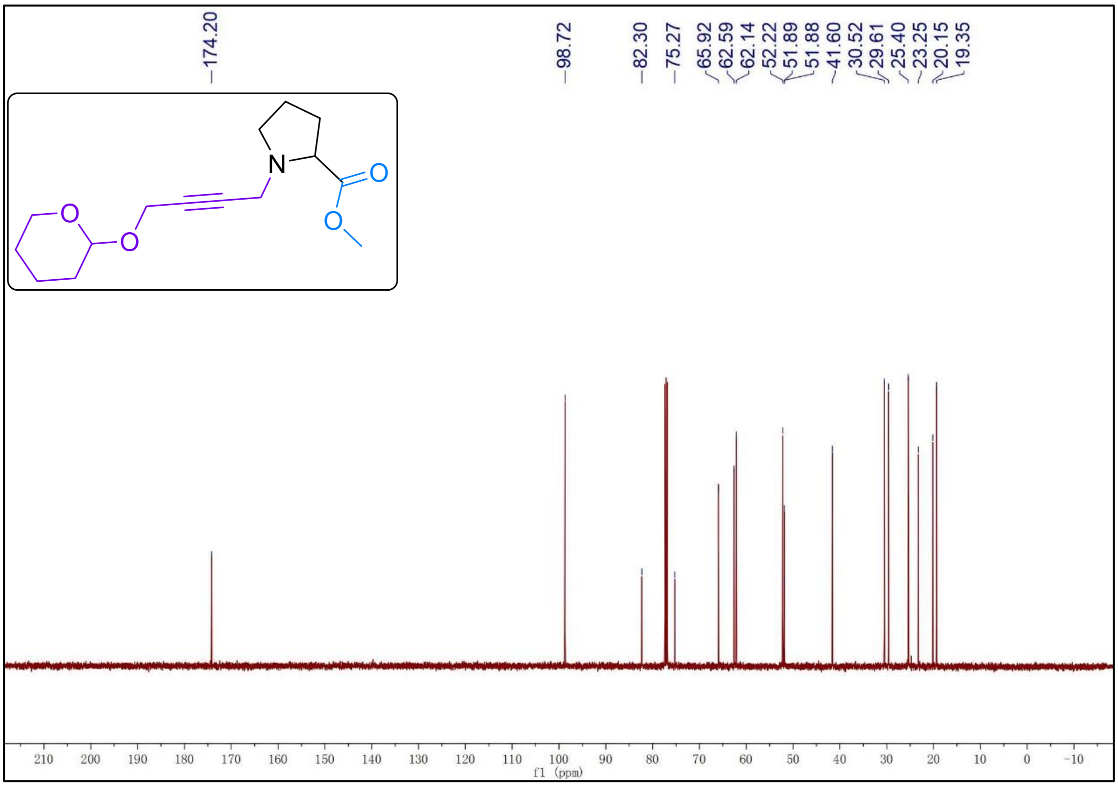

methyl (4-(2-hydroxypropoxy)but-2-yn-1-yl)prolinate

^{1}H NMR (500 MHz, Chloroform-*d*)

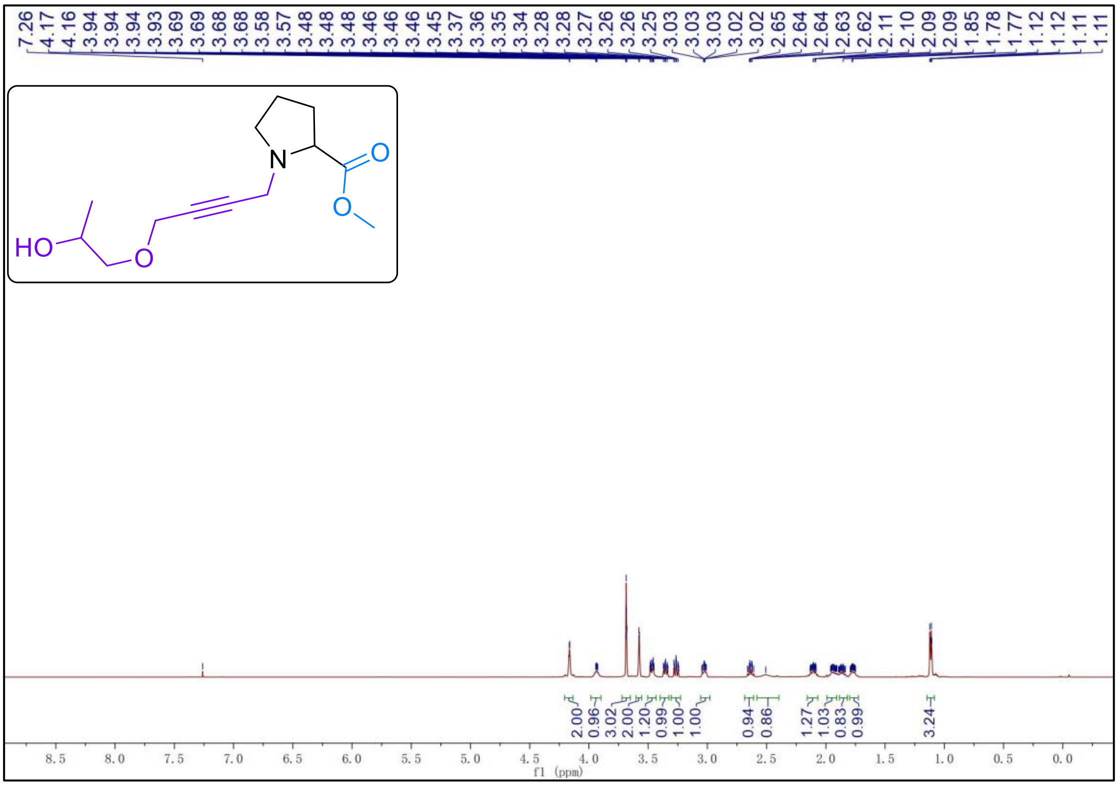

^{13}C NMR (126 MHz, Chloroform-*d*)

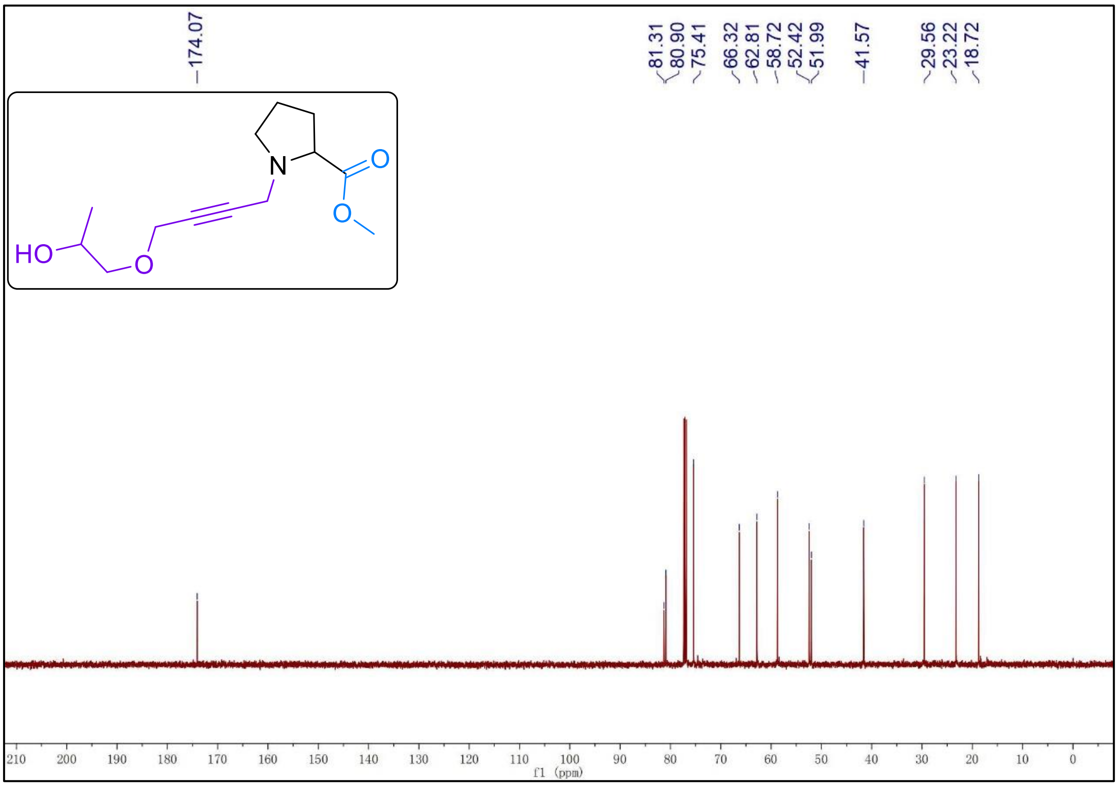

methyl (4-methylpent-4-en-2-yn-1-yl)prolinate

^{1}H NMR (500 MHz, Chloroform-*d*)

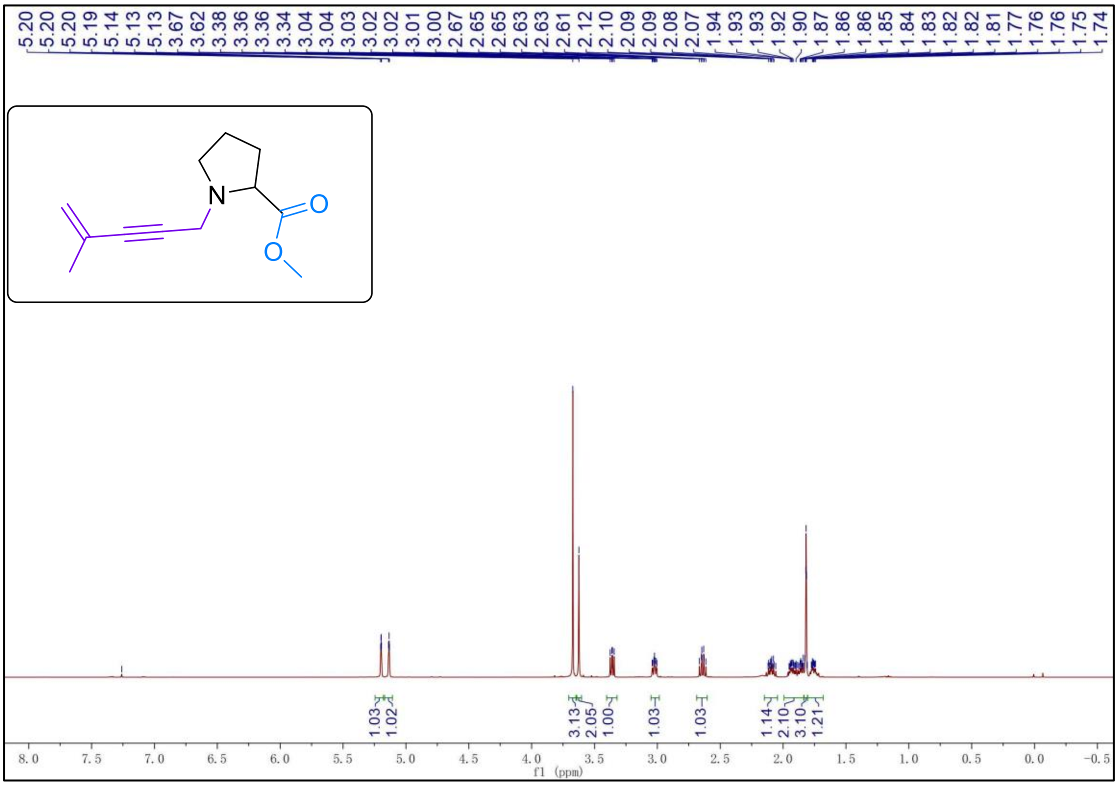

^{13}C NMR (126 MHz, Chloroform-*d*)

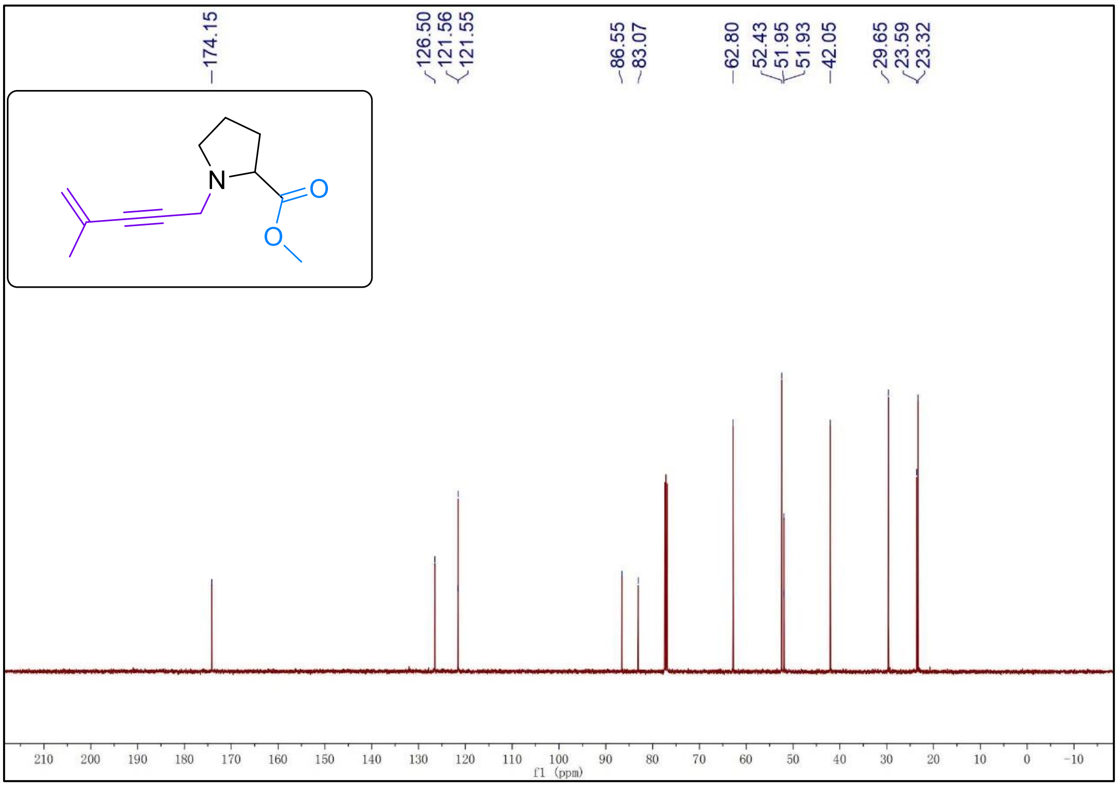

methyl (3-(cyclohex-1-en-1-yl)prop-2-yn-1-yl)prolinate

^{1}H NMR (500 MHz, Chloroform-*d*)

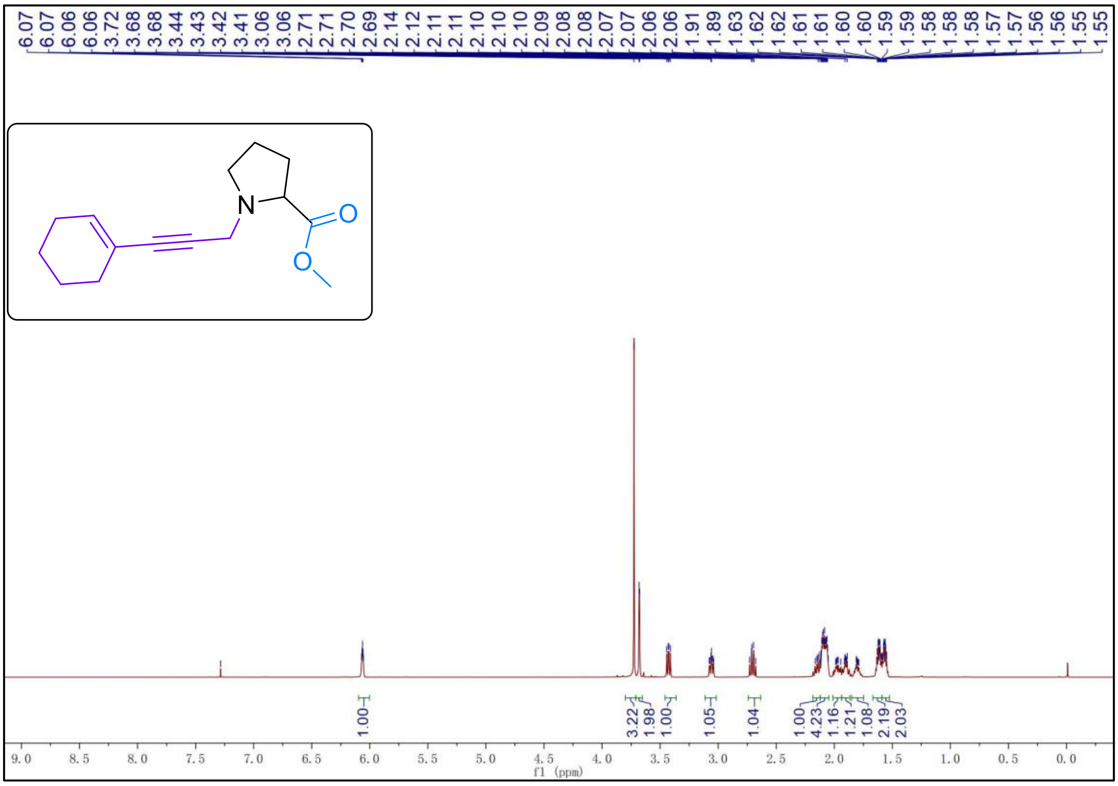

^{13}C NMR (126 MHz, Chloroform-*d*)

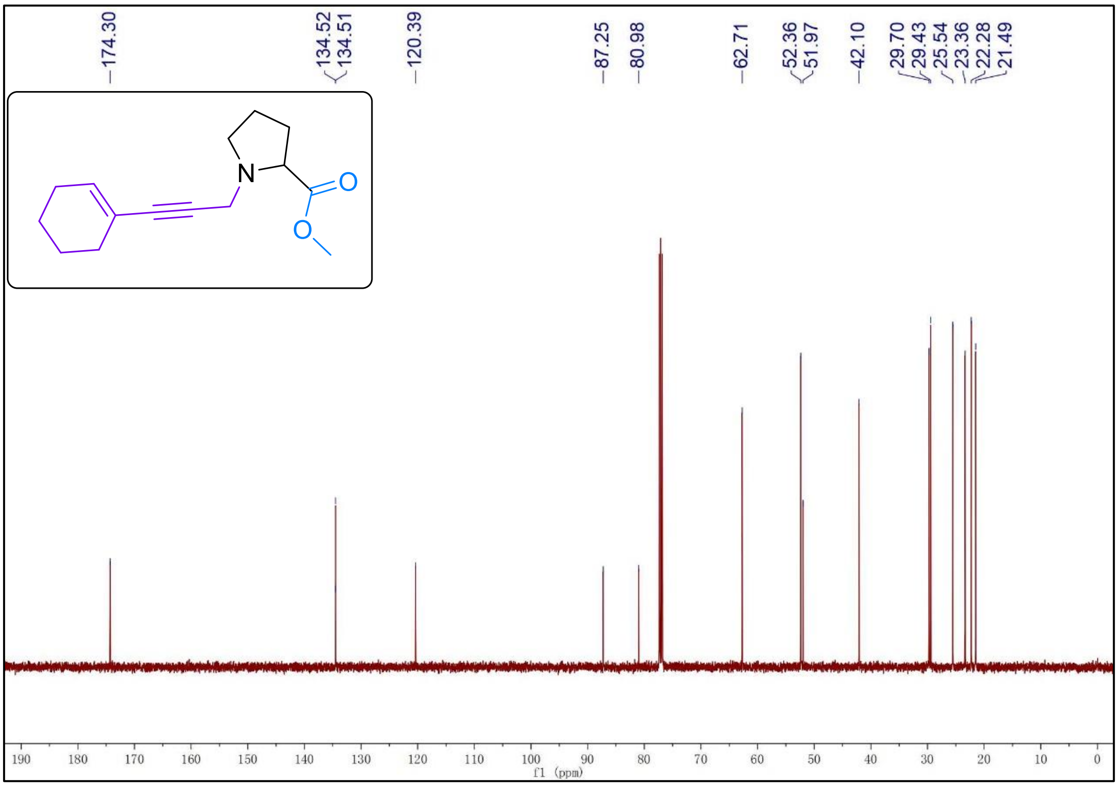

methyl (3-phenylprop-2-yn-1-yl)prolinate

^{1}H NMR (500 MHz, Chloroform-*d*)

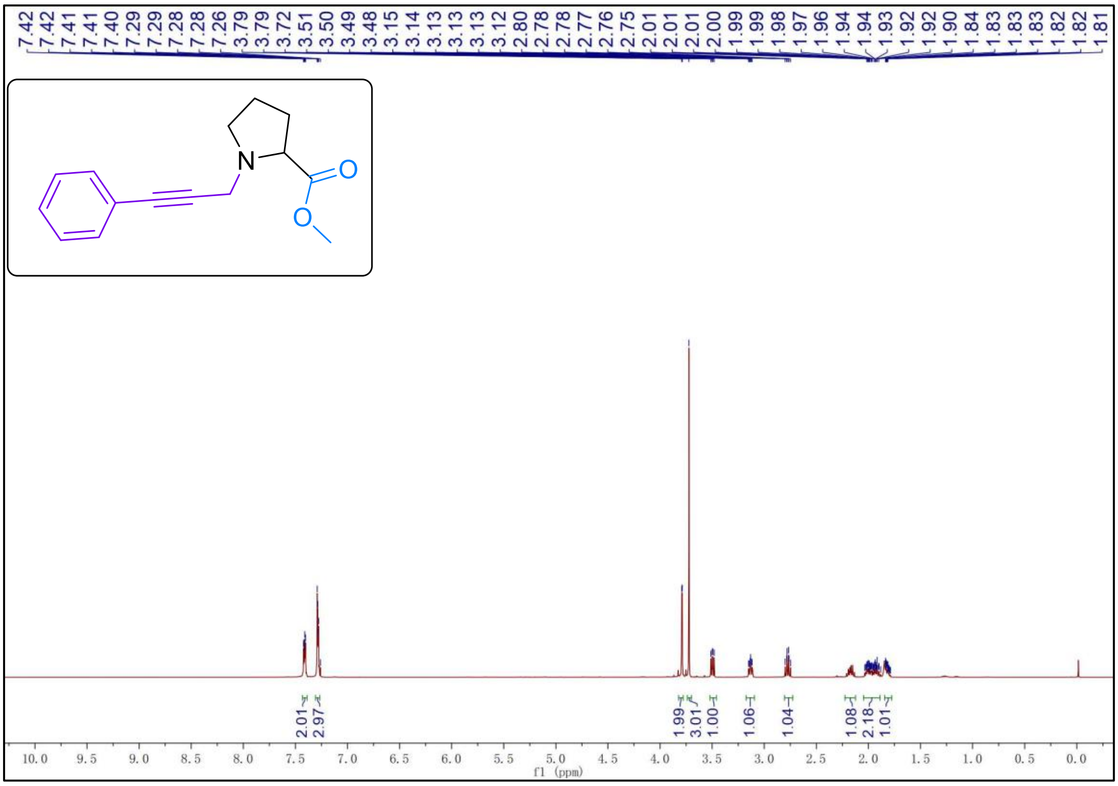

^{13}C NMR (126 MHz, Chloroform-*d*)

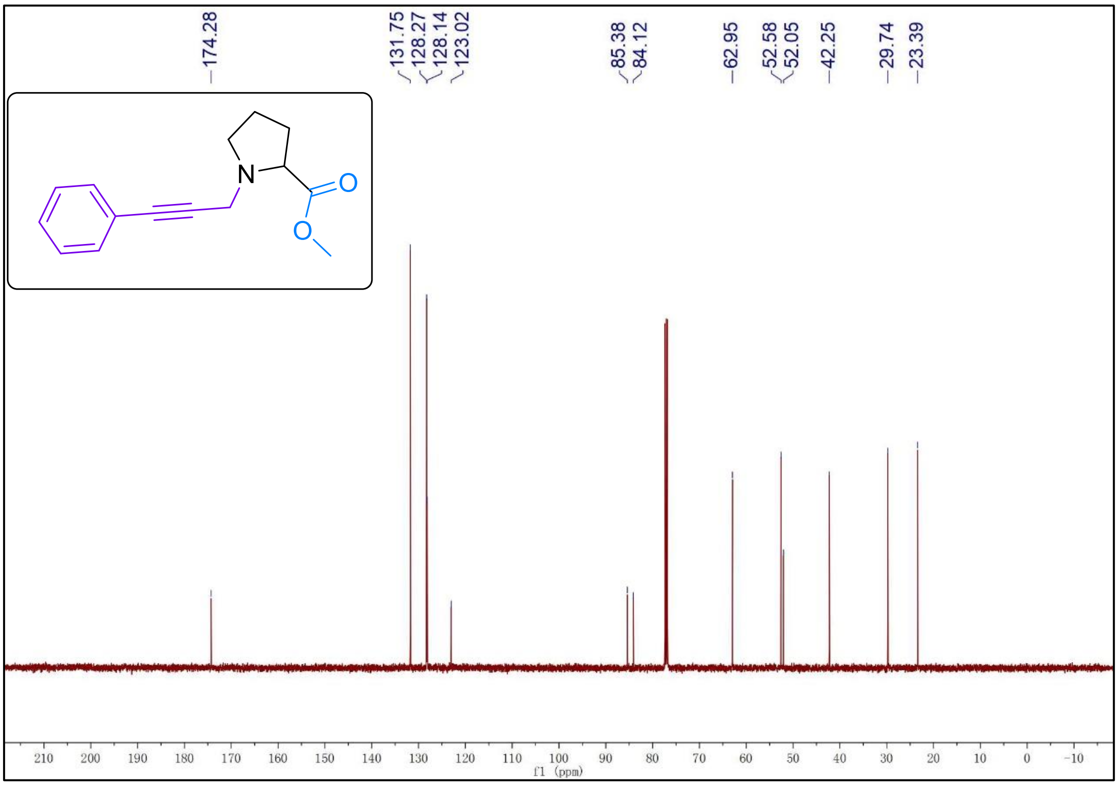

methyl (3-(4-ethylphenyl)prop-2-yn-1-yl)prolinate

^{1}H NMR (500 MHz, Chloroform-*d*)

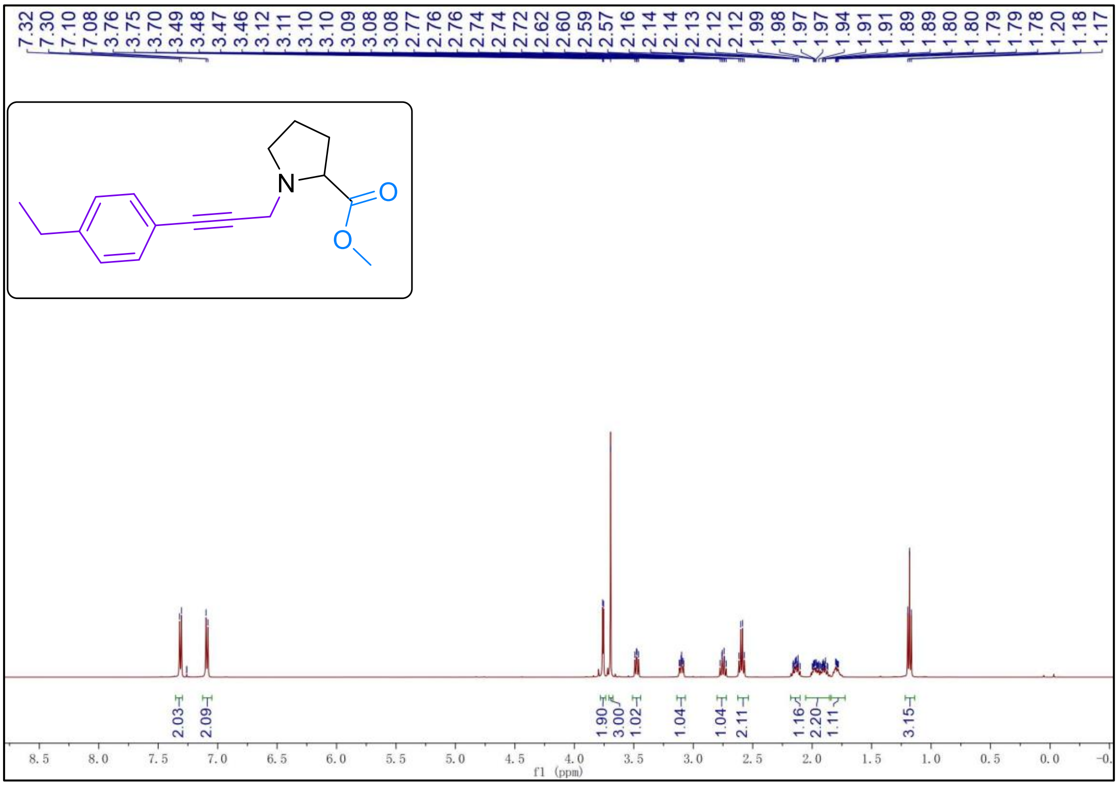

^{13}C NMR (126 MHz, Chloroform-*d*)

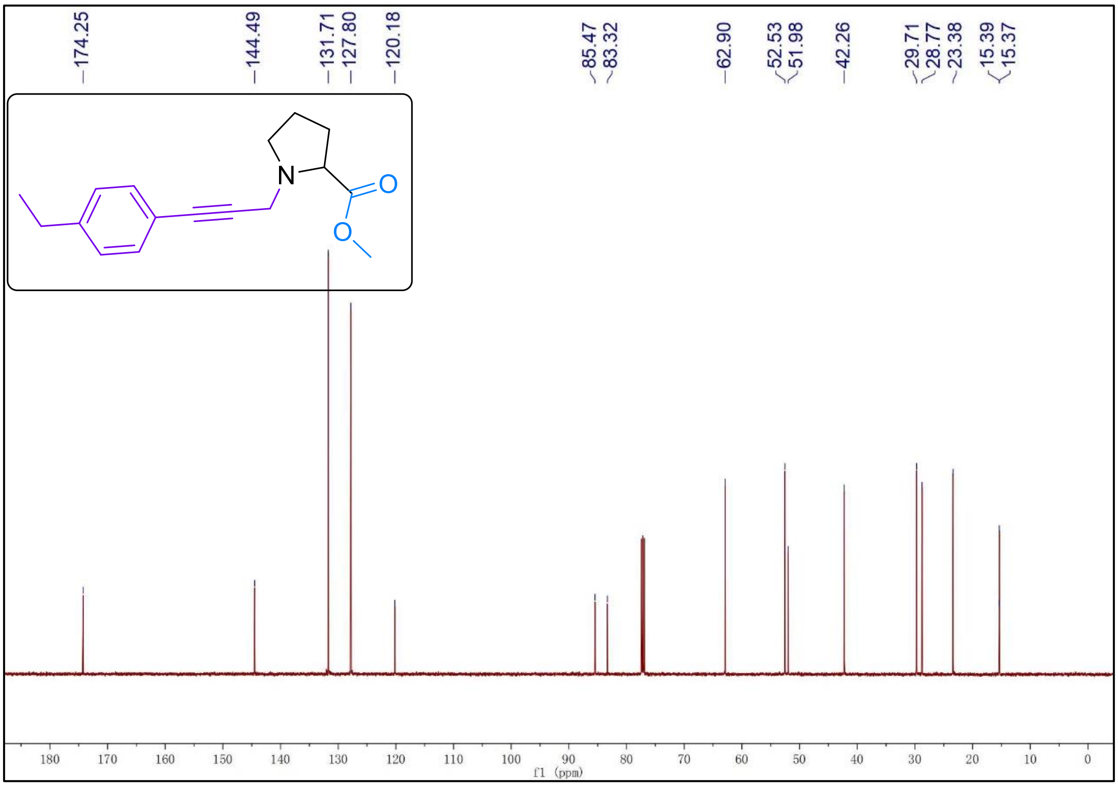

methyl (3-(4-methoxyphenyl)prop-2-yn-1-yl)prolinate

^{1}H NMR (500 MHz, Chloroform-*d*)

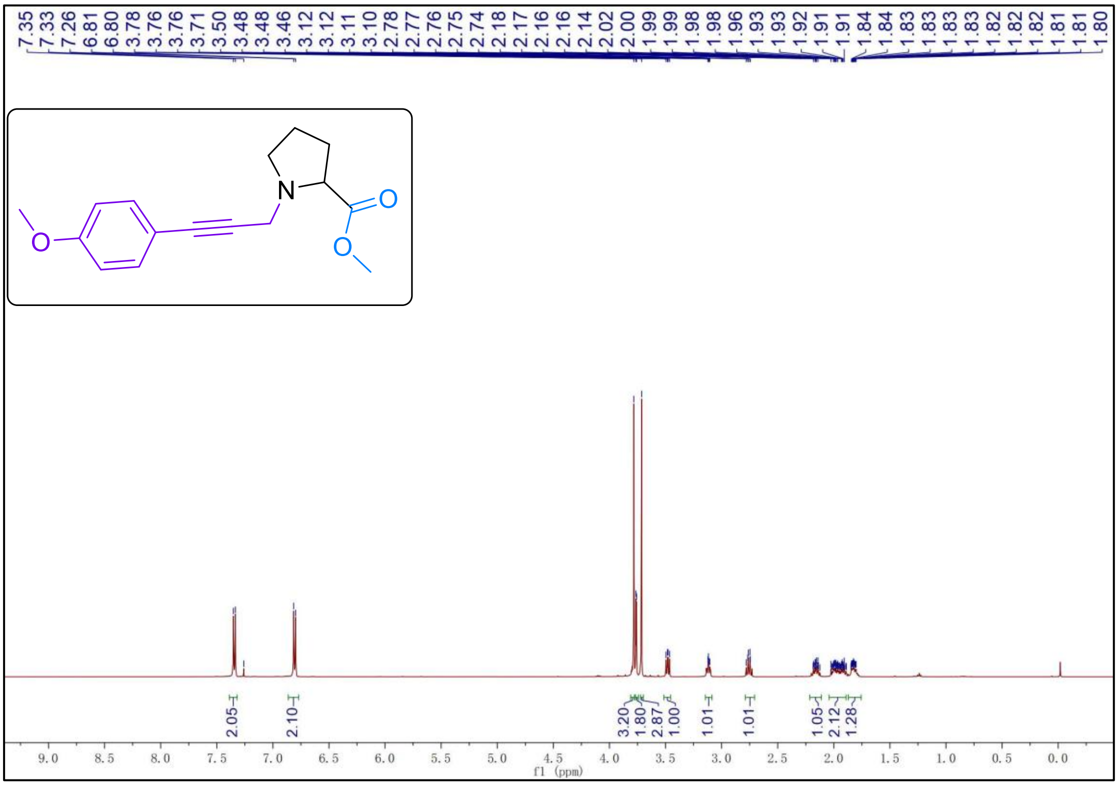

^{13}C NMR (126 MHz, Chloroform-*d*)

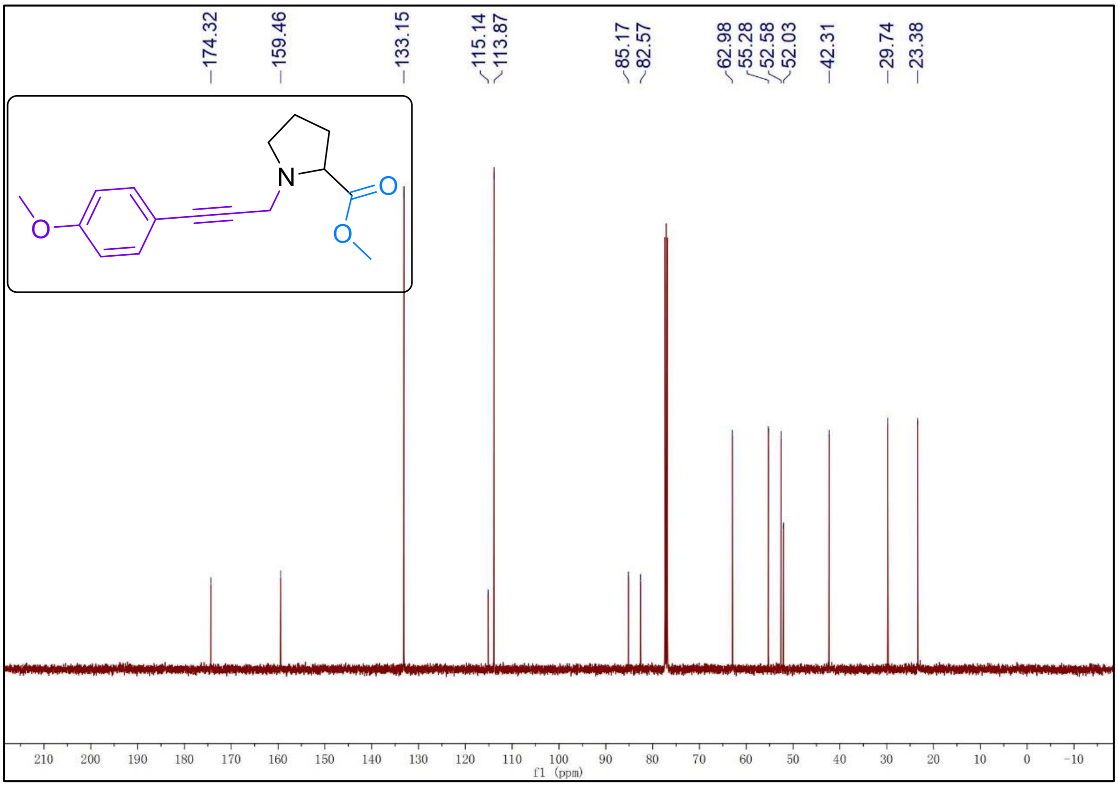

methyl (3-(4-chlorophenyl)prop-2-yn-1-yl)prolinate

^{1}H NMR (500 MHz, Chloroform-*d*)

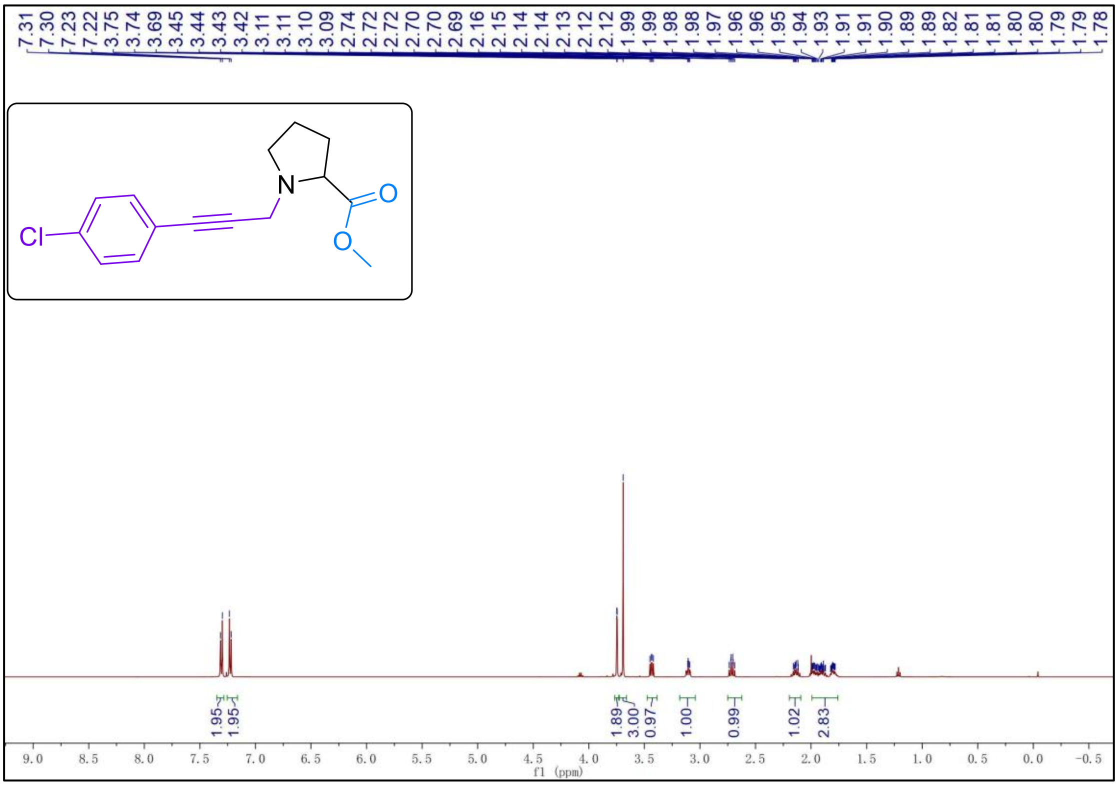

^{13}C NMR (126 MHz, Chloroform-*d*)

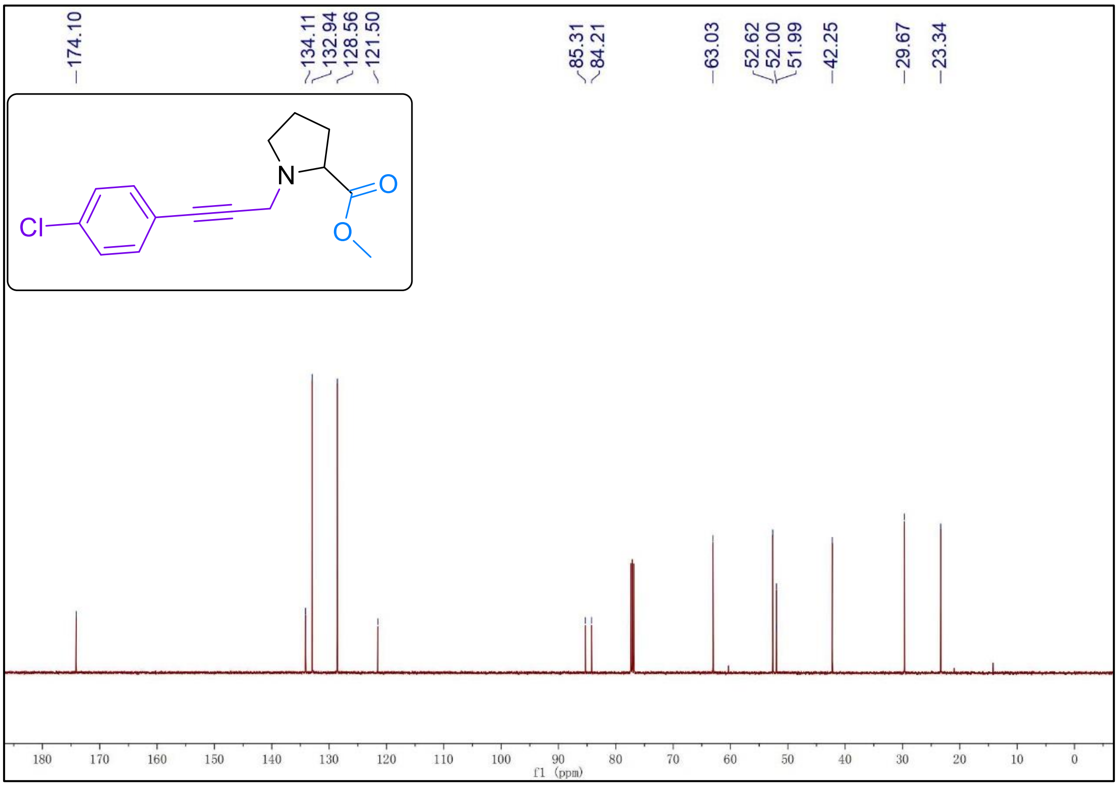

methyl (3-(4-bromophenyl)prop-2-yn-1-yl)prolinate

^{1}H NMR (500 MHz, Chloroform-*d*)

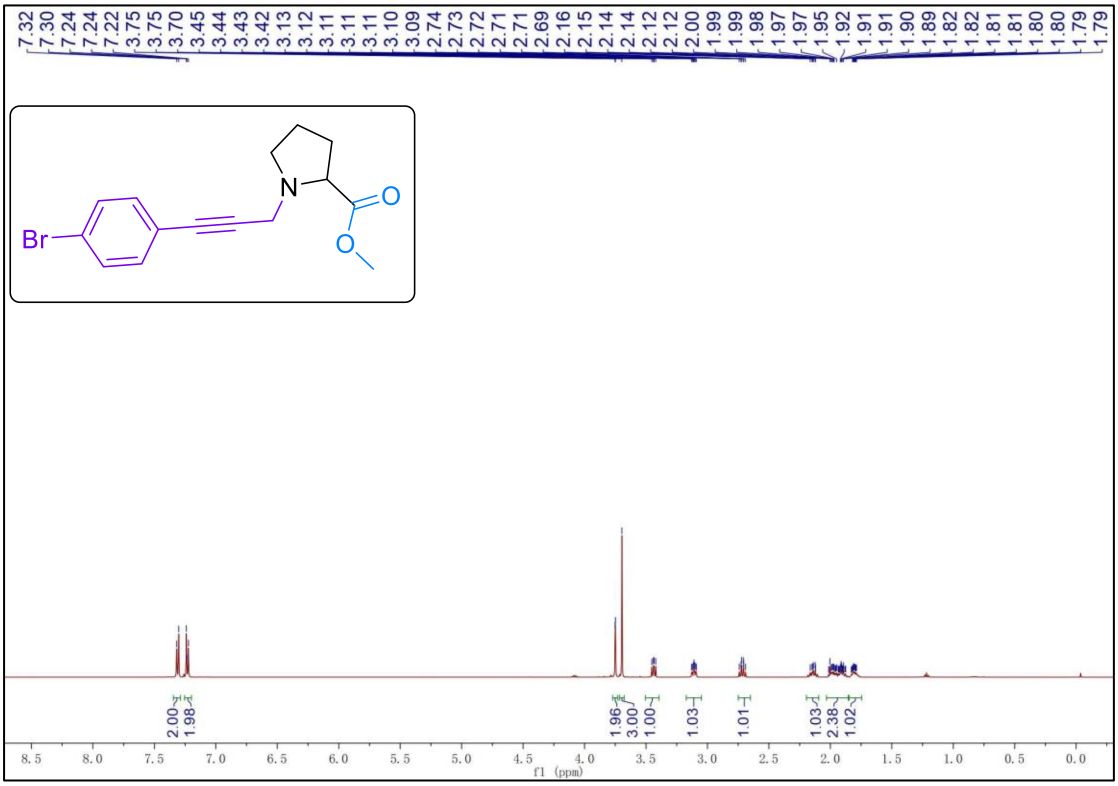

^{13}C NMR (126 MHz, Chloroform-*d*)

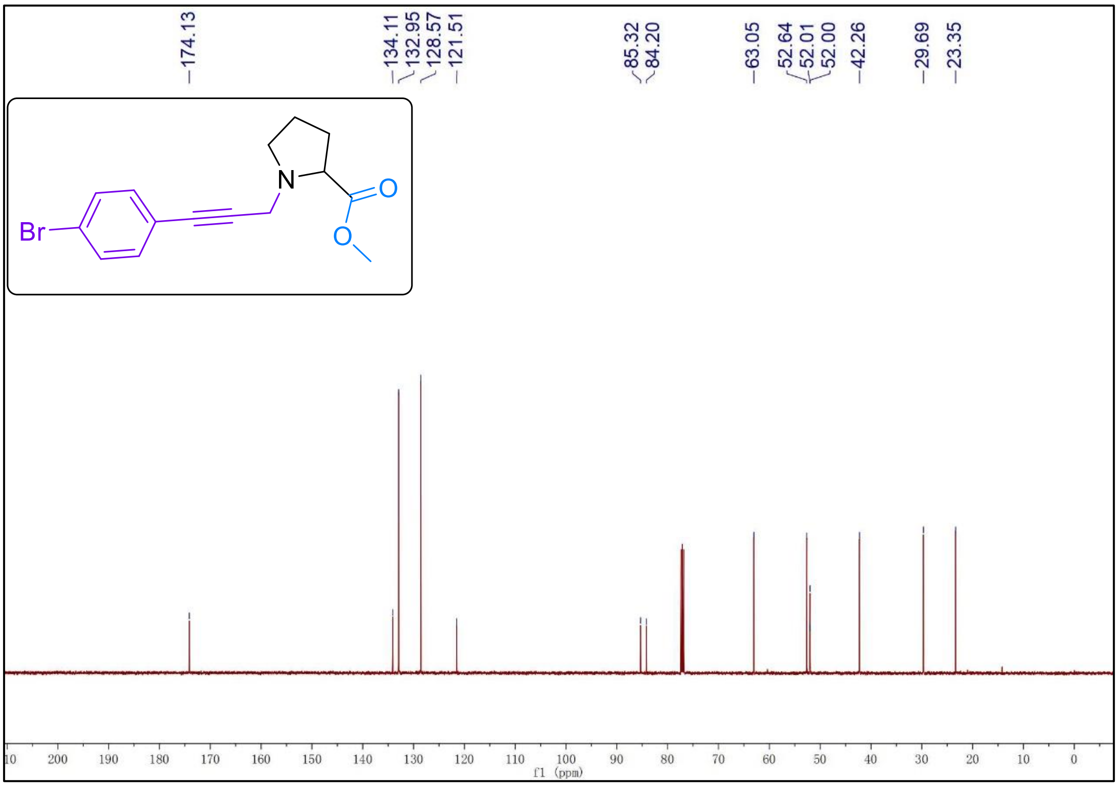

methyl (3-(4-formylphenyl)prop-2-yn-1-yl)prolinate

^{1}H NMR (500 MHz, Chloroform-*d*)

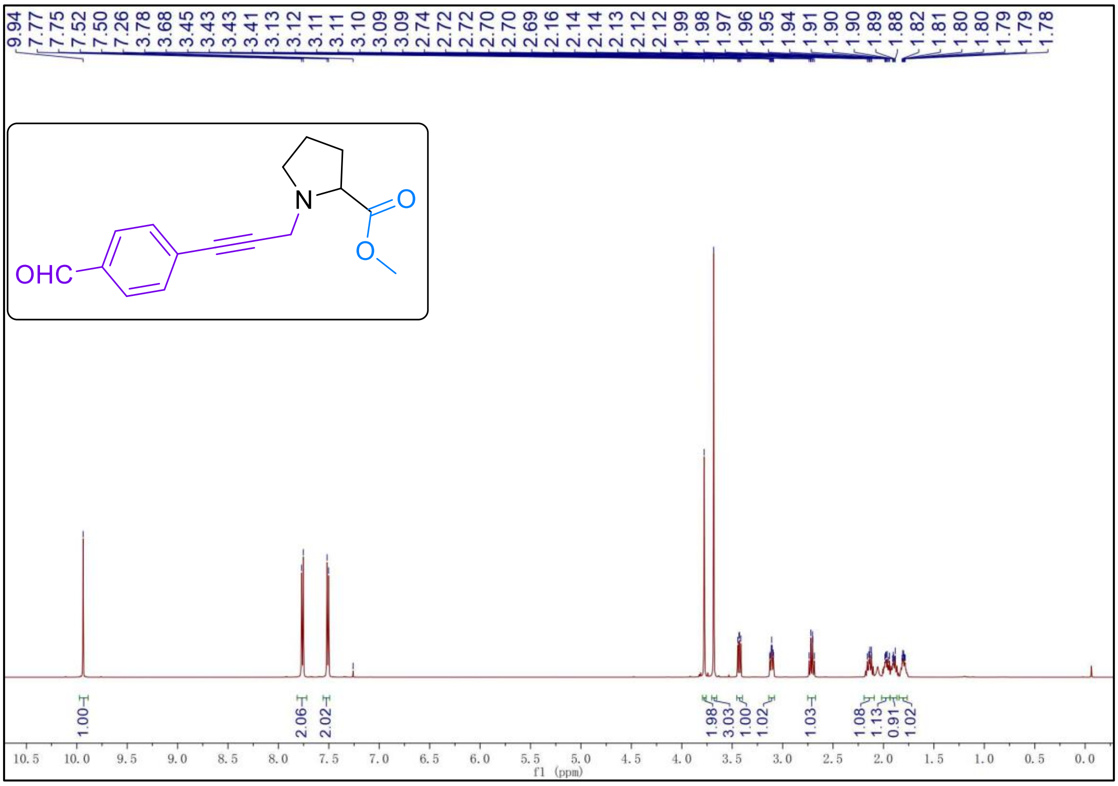

^{13}C NMR (126 MHz, Chloroform-*d*)

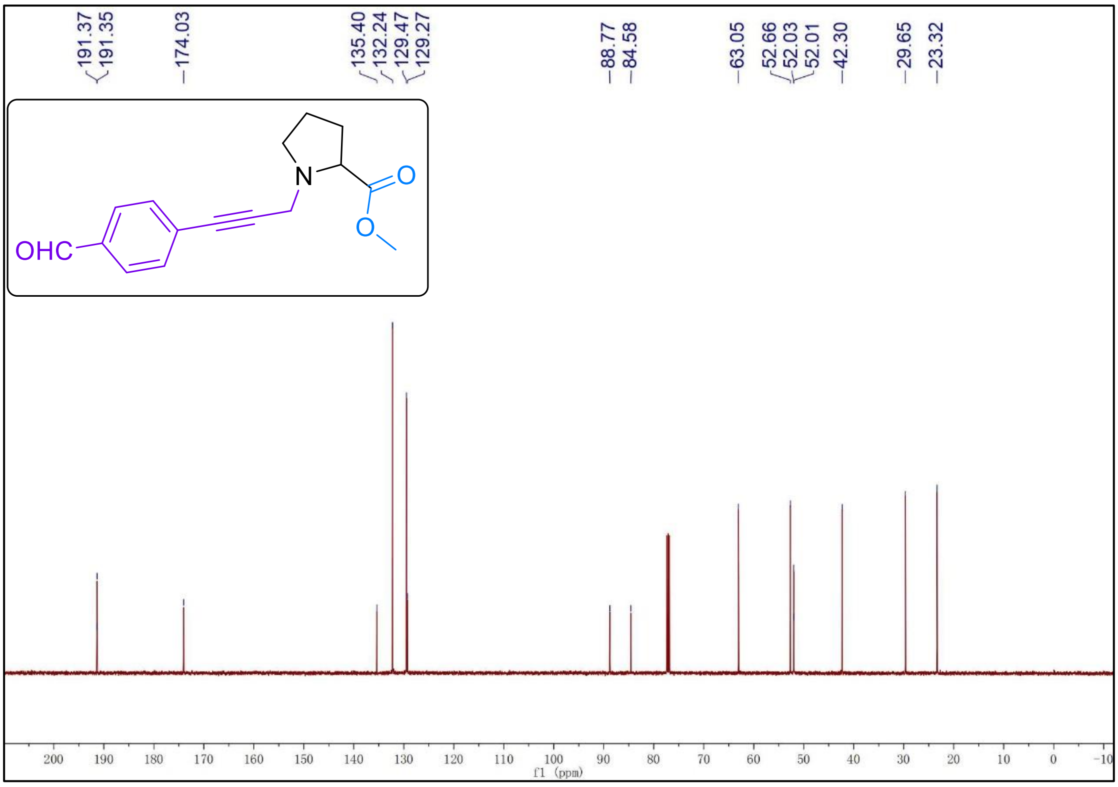

methyl -2-(*N*-methylformamido)pent-4-enoate（3a)

^{1}H NMR (500 MHz, Chloroform-*d*)

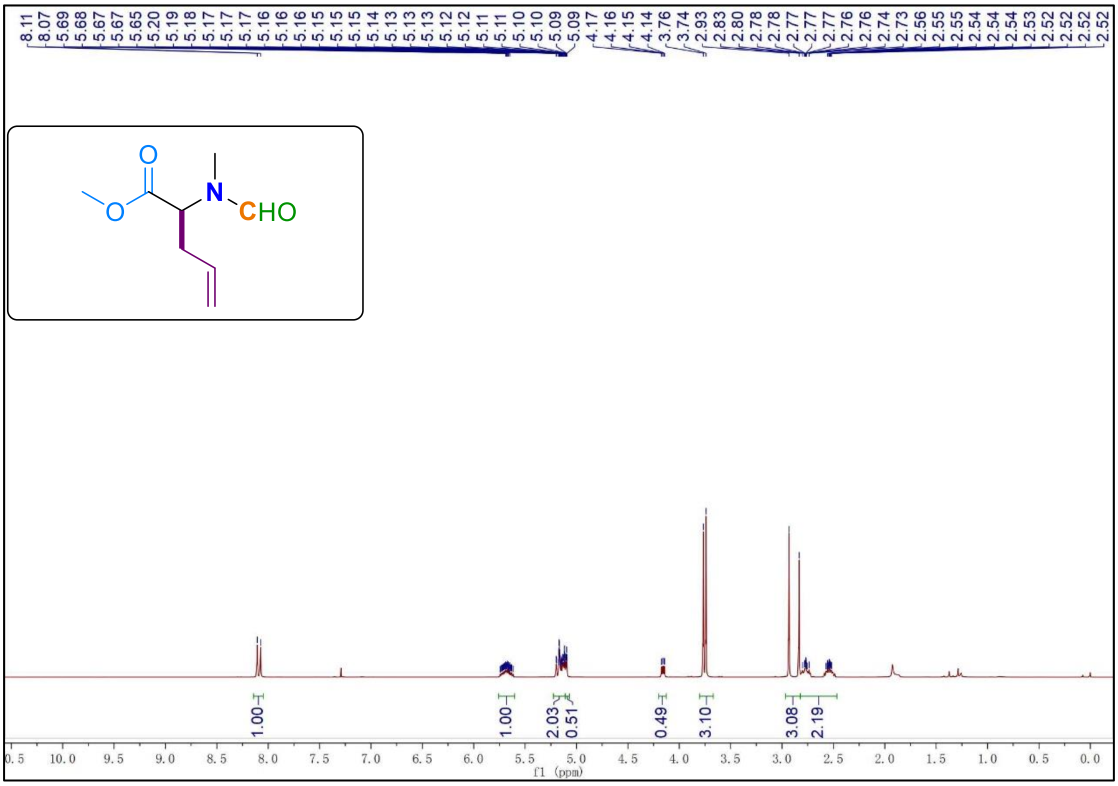

^{13}C NMR (126 MHz, Chloroform-*d*)

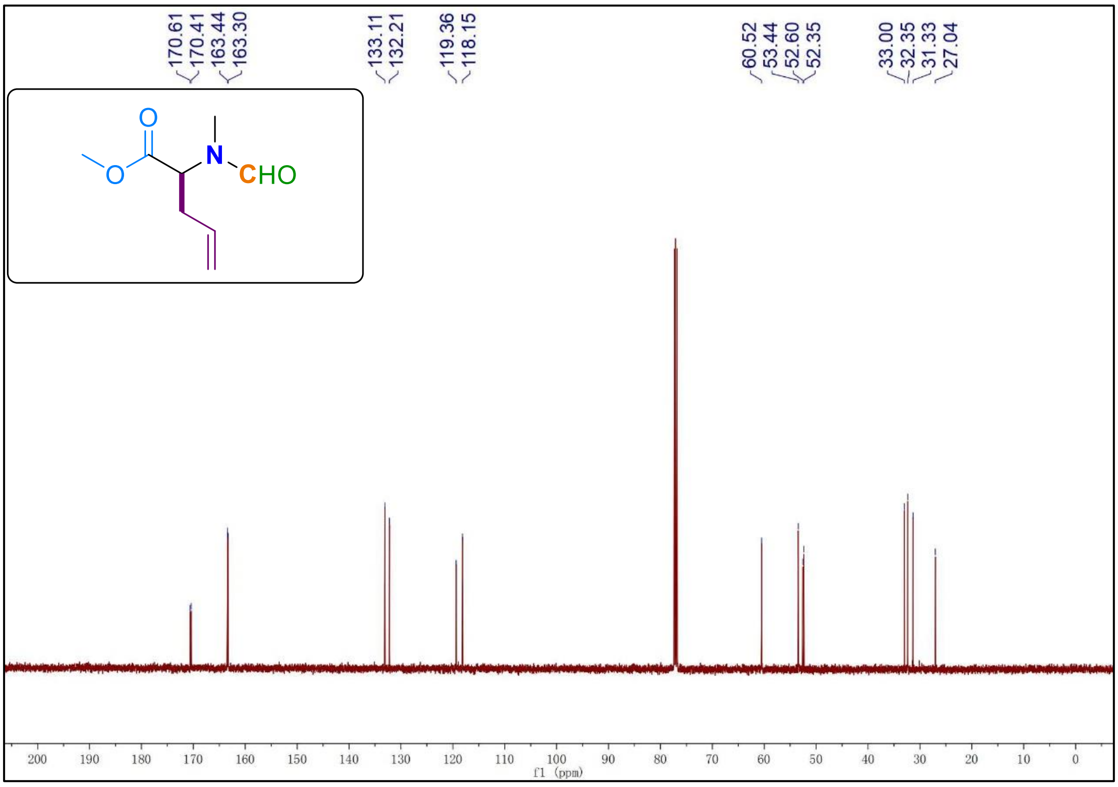

ethyl 2-(*N*-methylformamido)pent-4-enoate (3b)

^{1}H NMR (500 MHz, Chloroform-*d*)

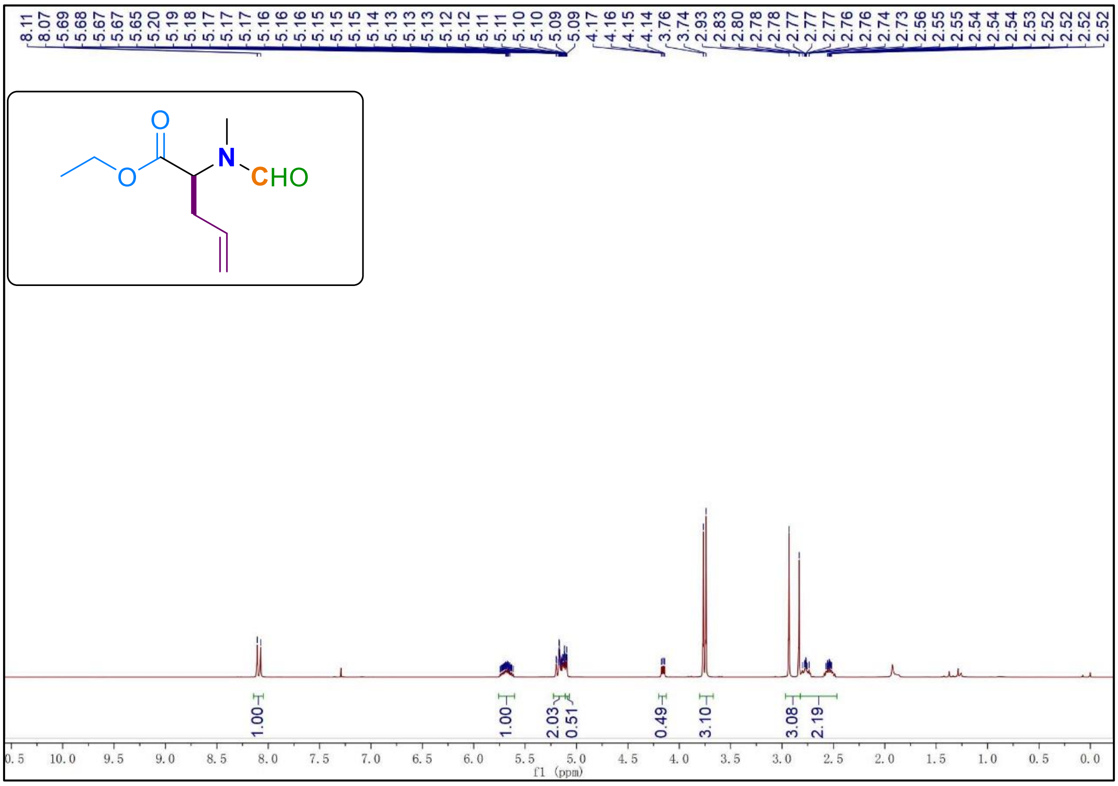

^{13}C NMR (126 MHz, Chloroform-*d*)

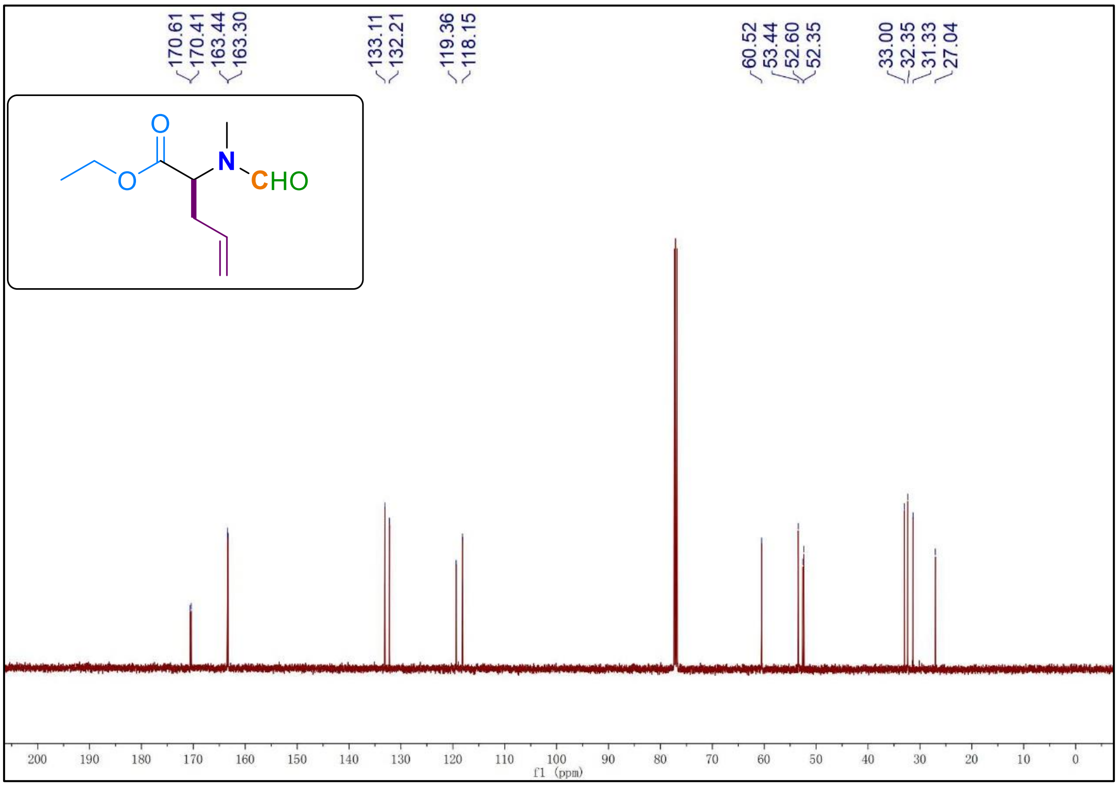

tert-butyl 2-(*N*-methylformamido)pent-4-enoate (3c)

^{1}H NMR (500 MHz, Chloroform-*d*)

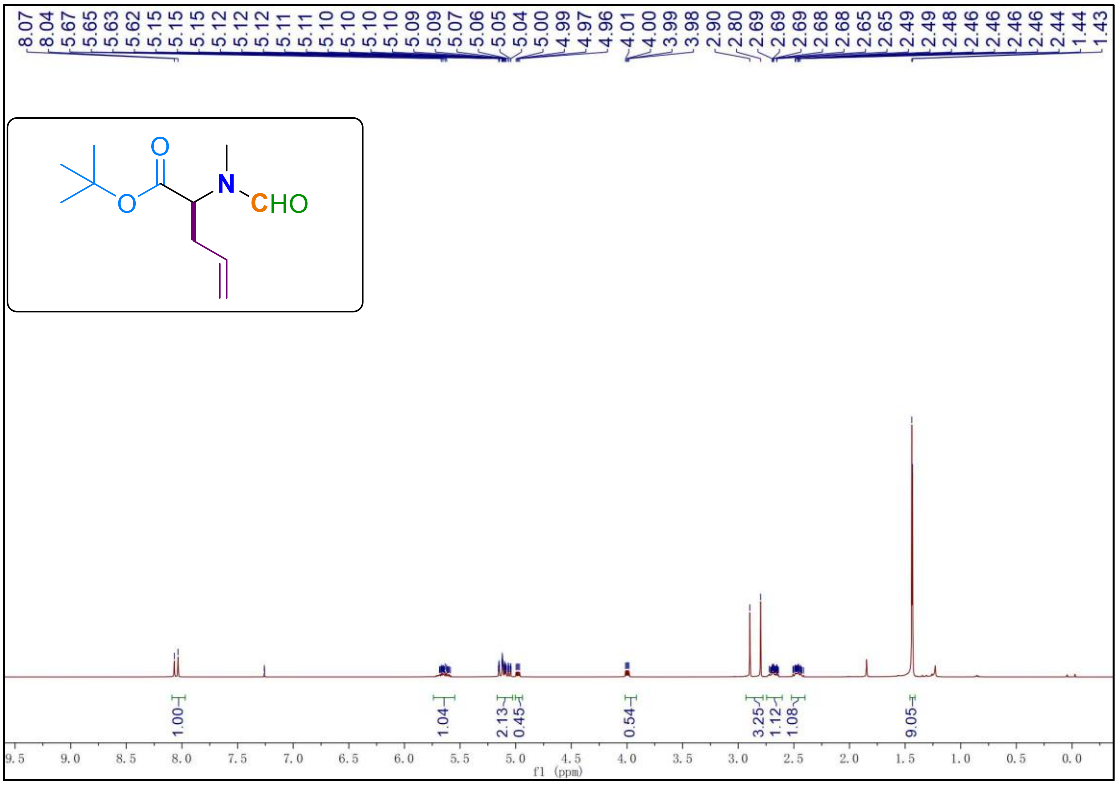

^{13}C NMR (126 MHz, Chloroform-*d*)

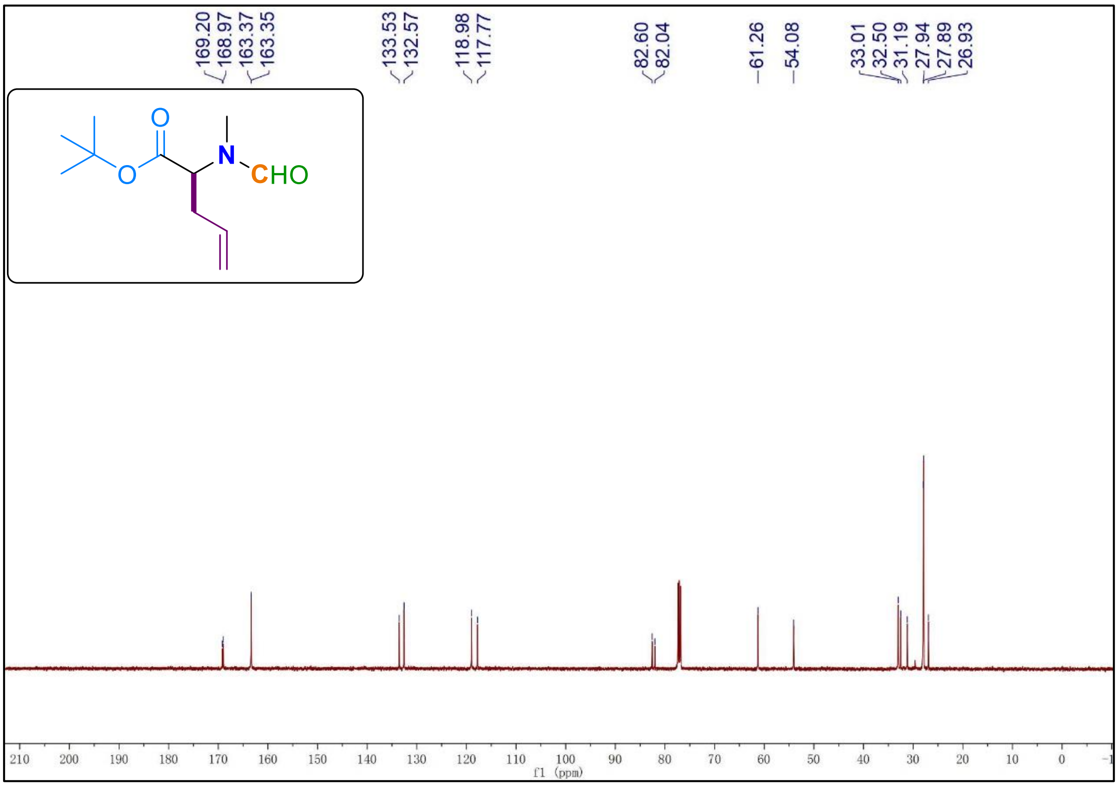

cyclopropylmethyl 2-(*N*-methylformamido)pent-4-enoate (3d)

^{1}H NMR (500 MHz, Chloroform-*d*)

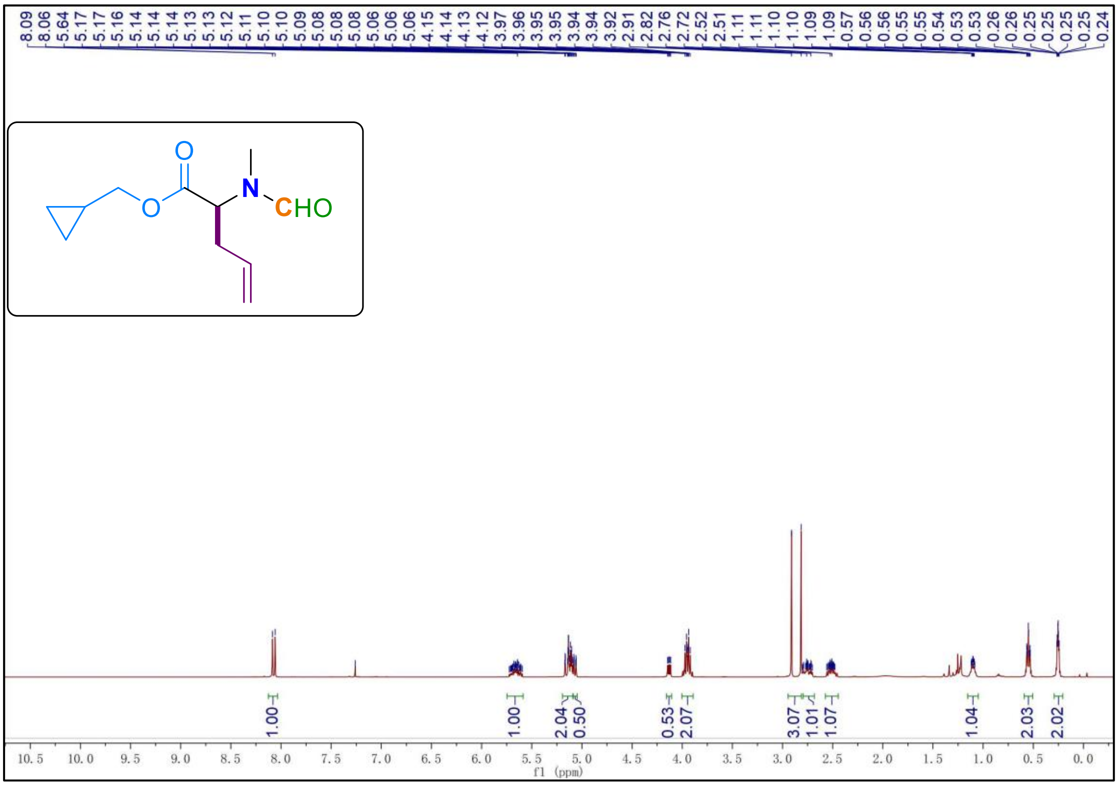

^{13}C NMR (126 MHz, Chloroform-*d*)

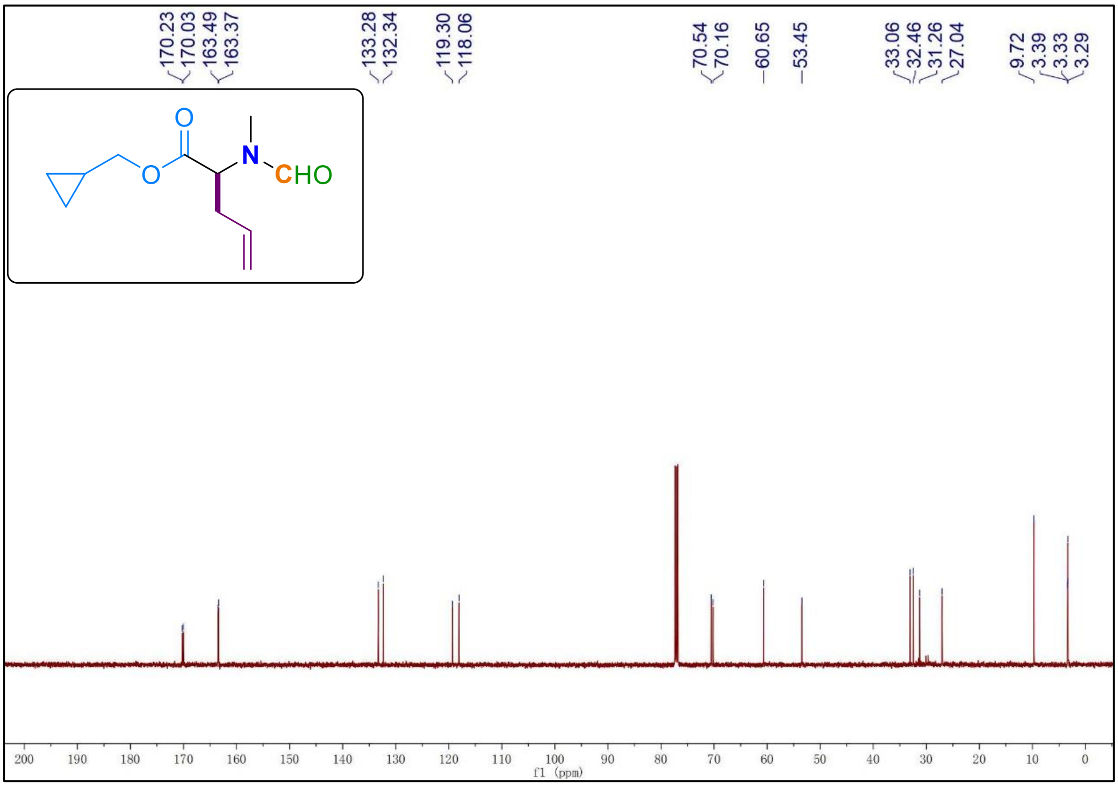

isopentyl 2-(*N*-methylformamido)pent-4-enoate (3e)

^{1}H NMR (500 MHz, Chloroform-*d*)

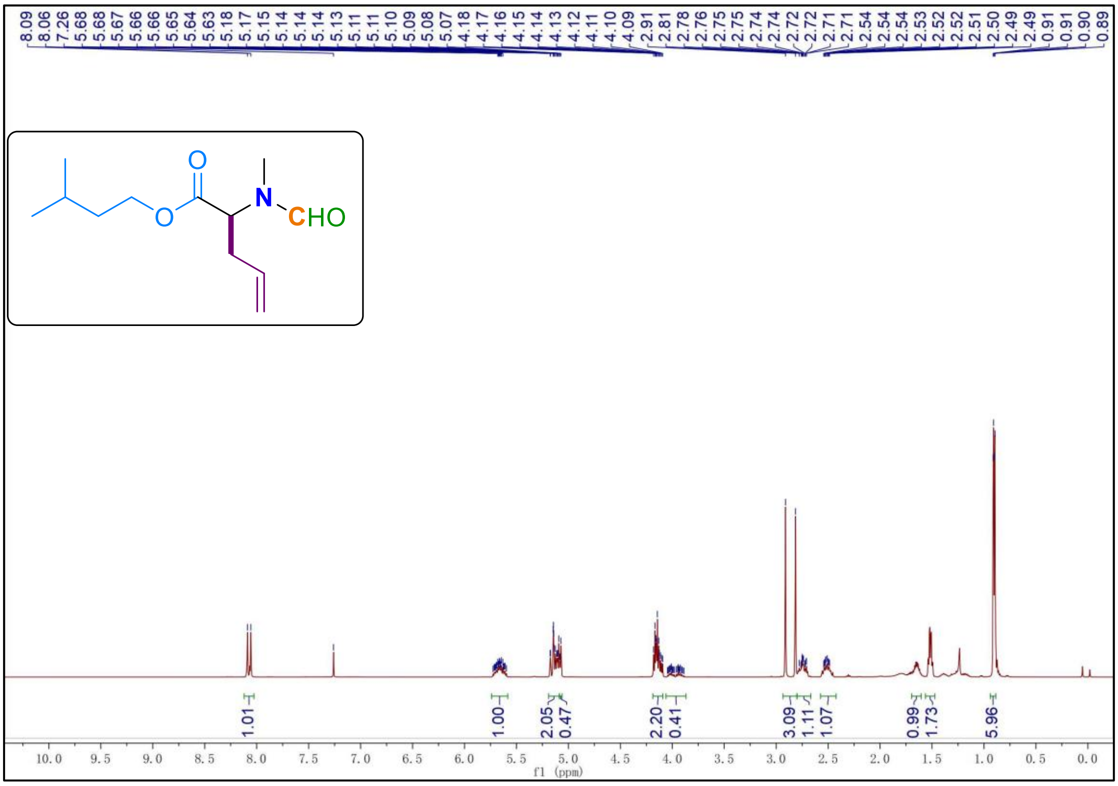

^{13}C NMR (126 MHz, Chloroform-*d*)

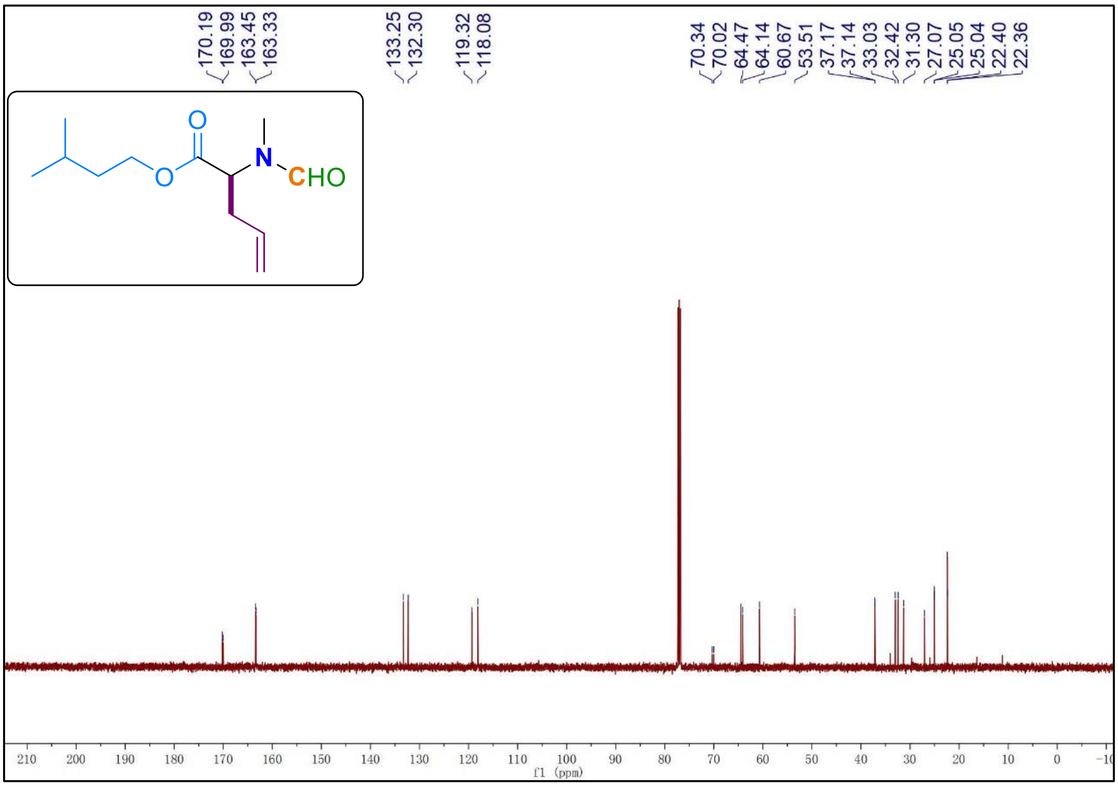

3-methylbut-3-en-1-yl -2-(*N*-methylformamido)pent-4-enoate (3f)

^{1}H NMR (500 MHz, Chloroform-*d*)

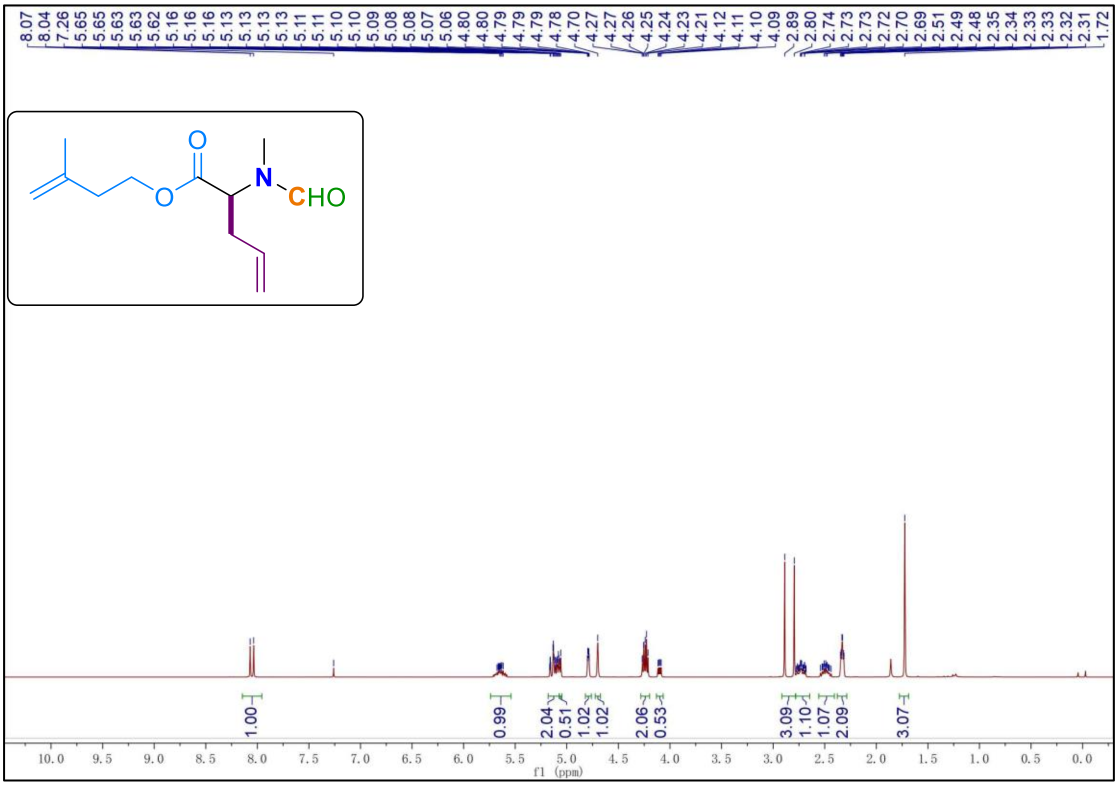

^{13}C NMR (126 MHz, Chloroform-*d*)

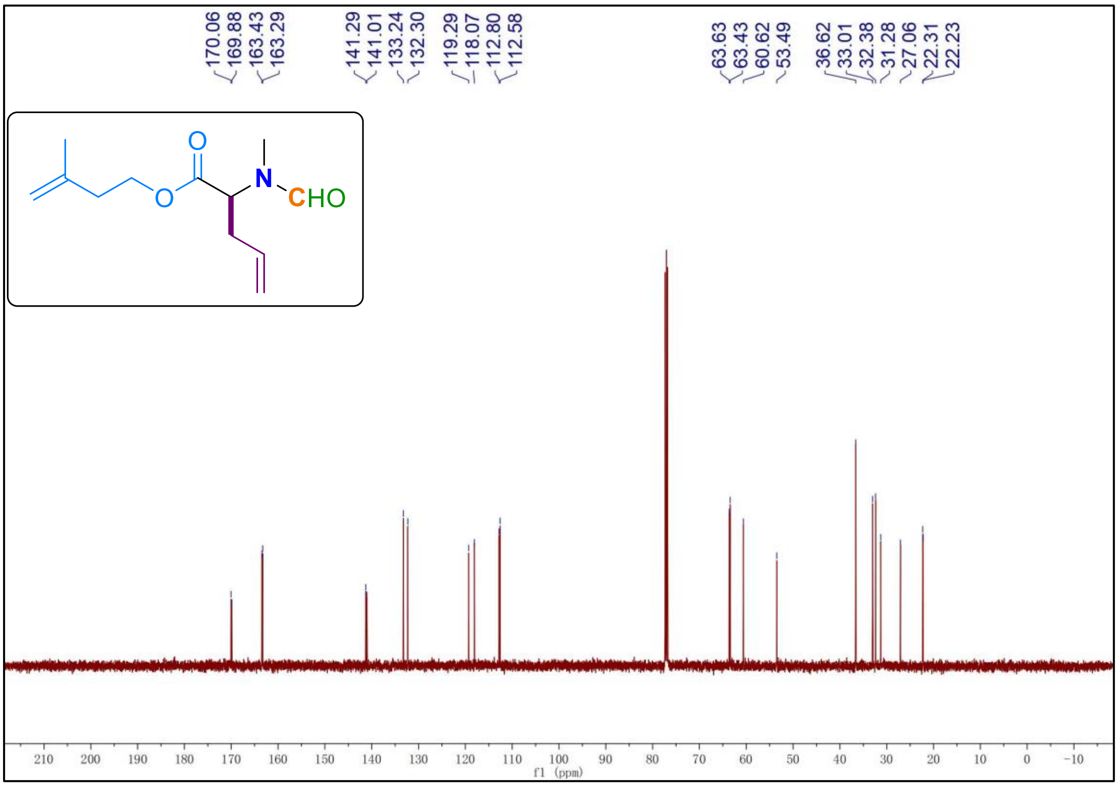

benzyl 2-(*N*-methylformamido)pent-4-enoate (3g)

^{1}H NMR (500 MHz, Chloroform-*d*)

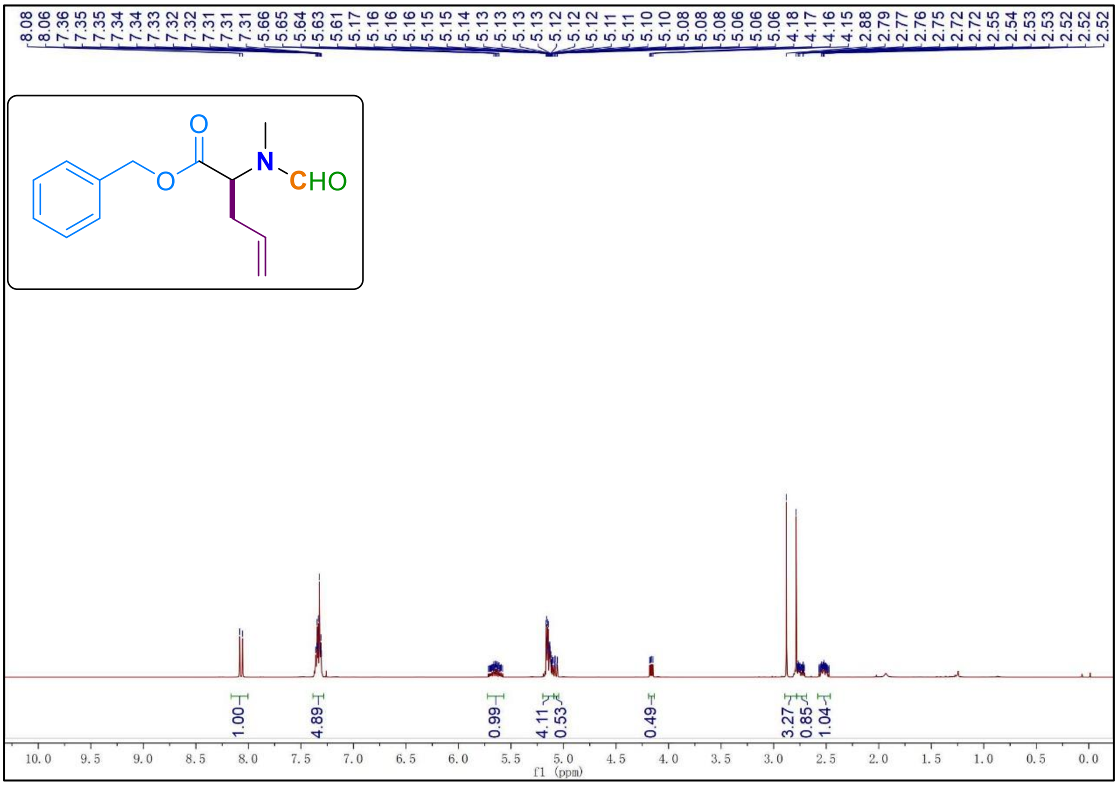

^{13}C NMR (126 MHz, Chloroform-*d*)

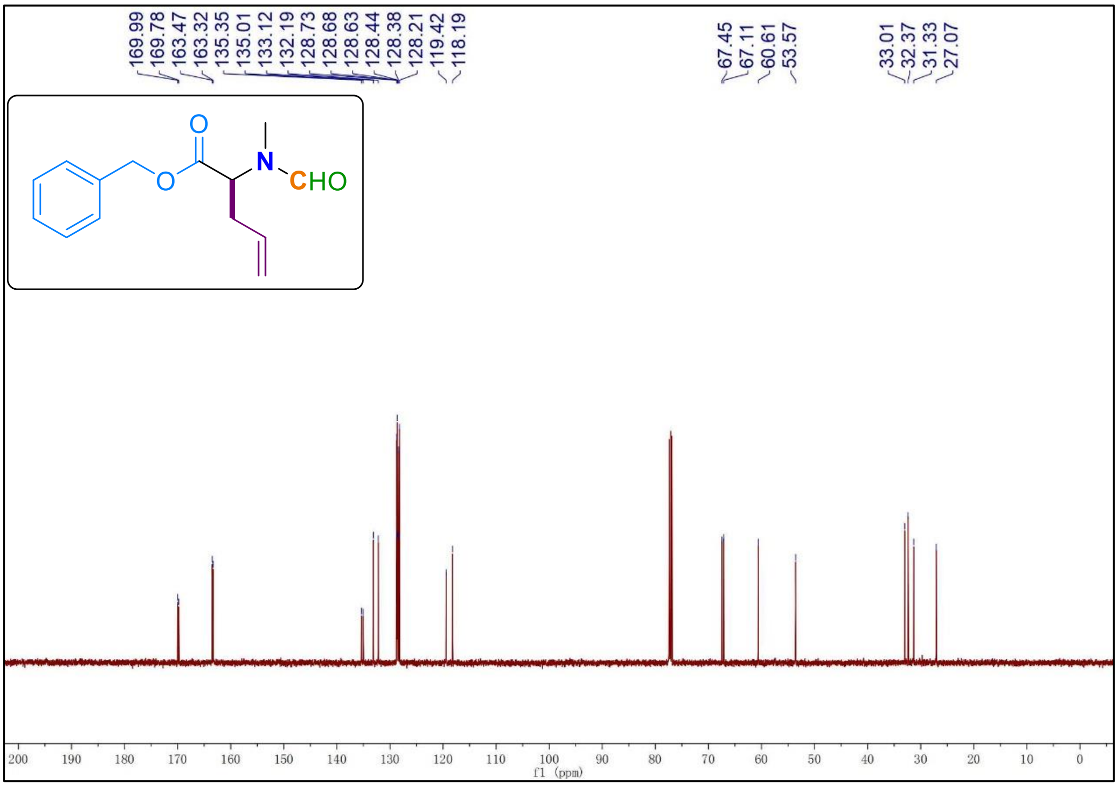

4-methylbenzyl 2-(*N*-methylformamido)pent-4-enoate (3h)

^{1}H NMR (500 MHz, Chloroform-*d*)

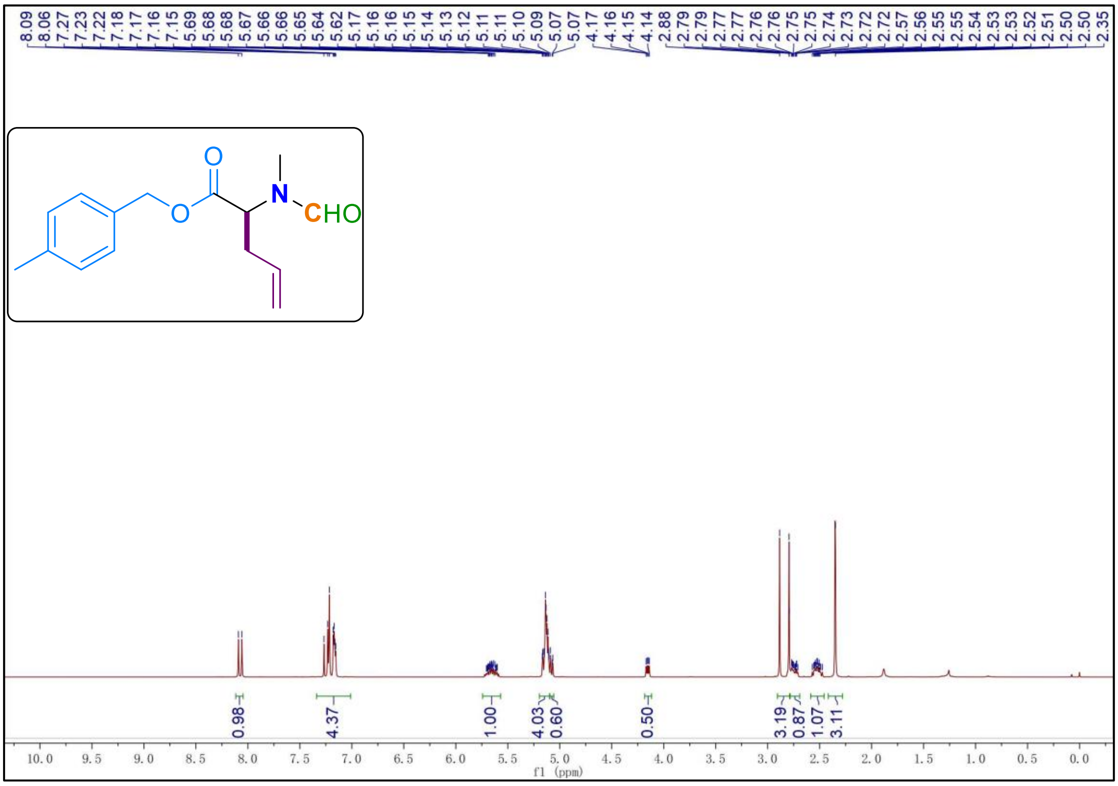

^{13}C NMR (126 MHz, Chloroform-*d*)

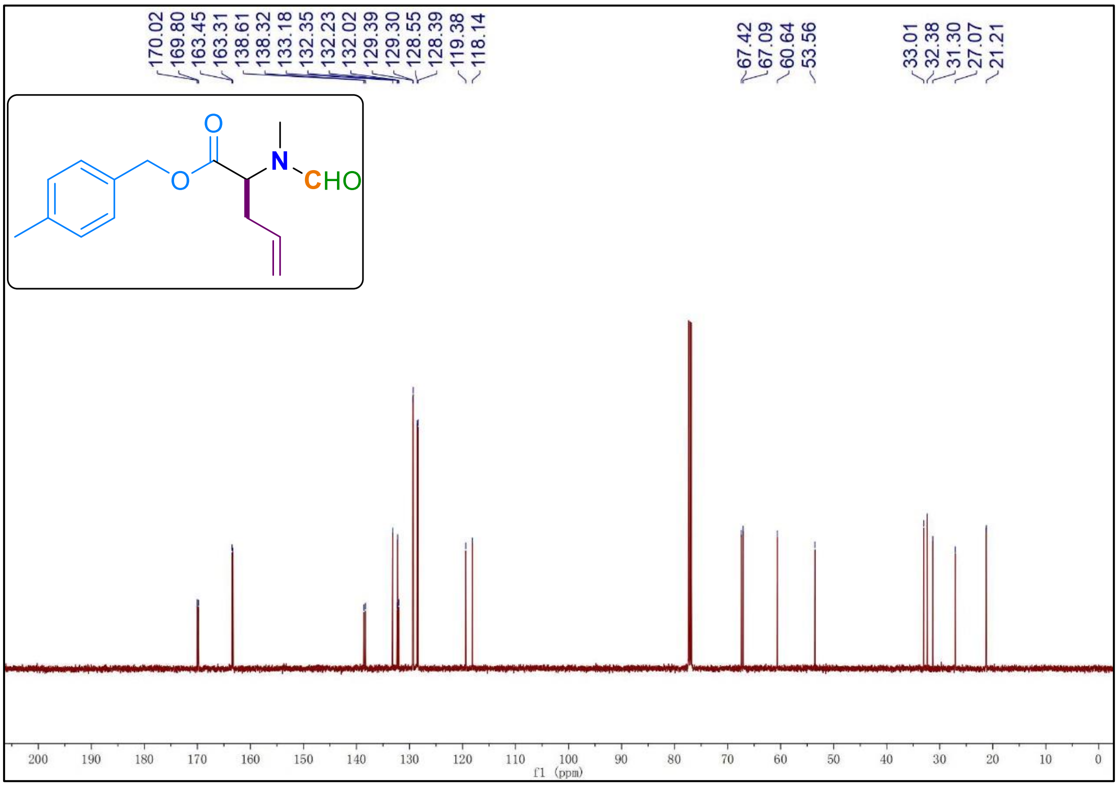

4-(*tert*-butyl)benzyl 2-(*N*-methylformamido)pent-4-enoate (3i)

^{1}H NMR (500 MHz, Chloroform-*d*)

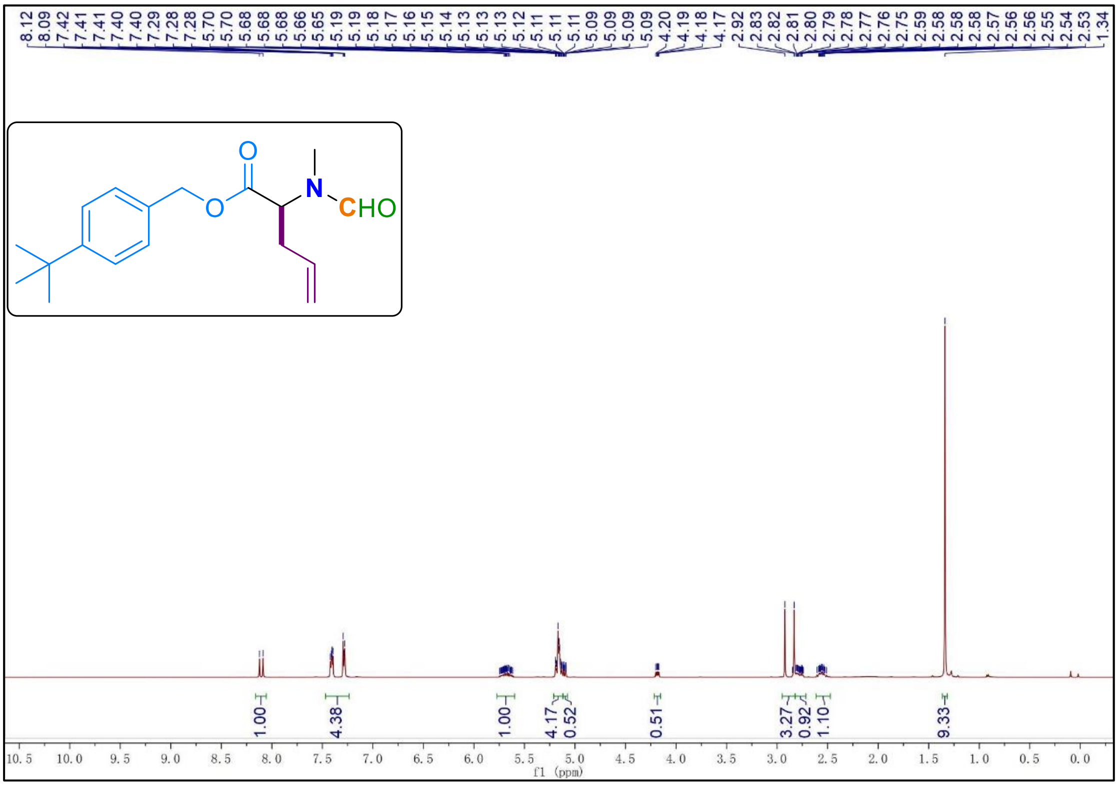

^{13}C NMR (126 MHz, Chloroform-*d*)

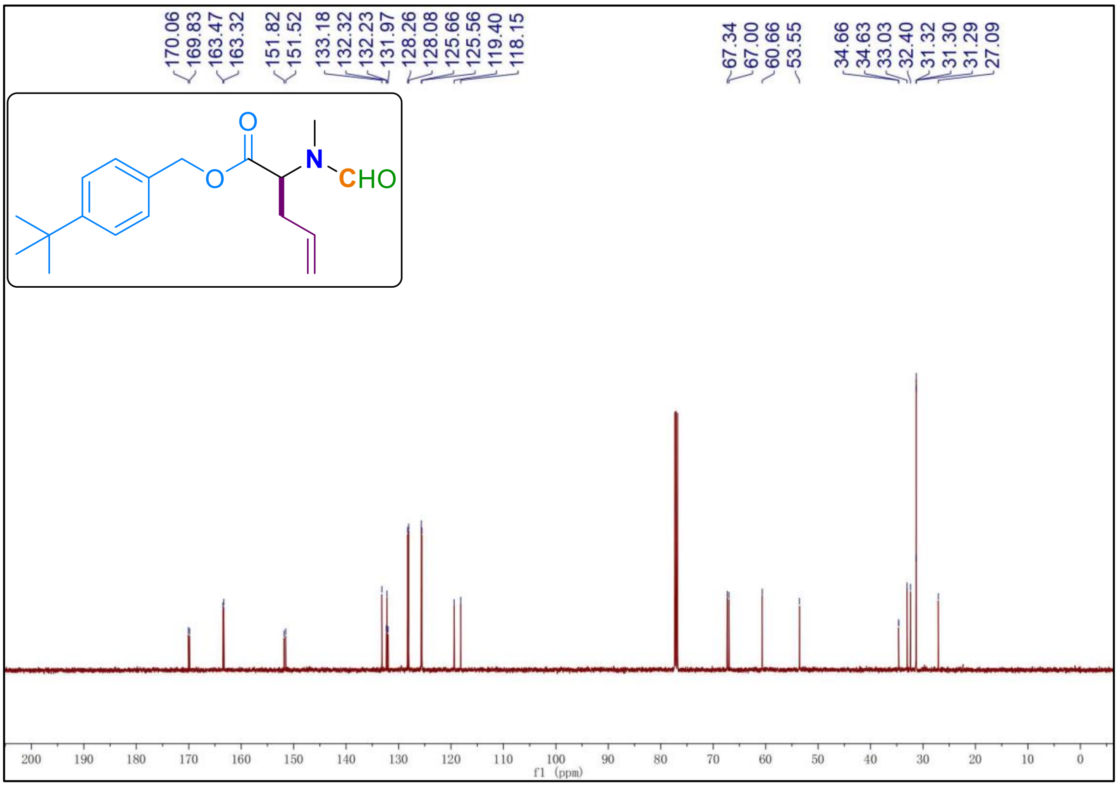

4-(benzyloxy)benzyl 2-(*N*-methylformamido)pent-4-enoate (3j)

^{1}H NMR (500 MHz, Chloroform-*d*)

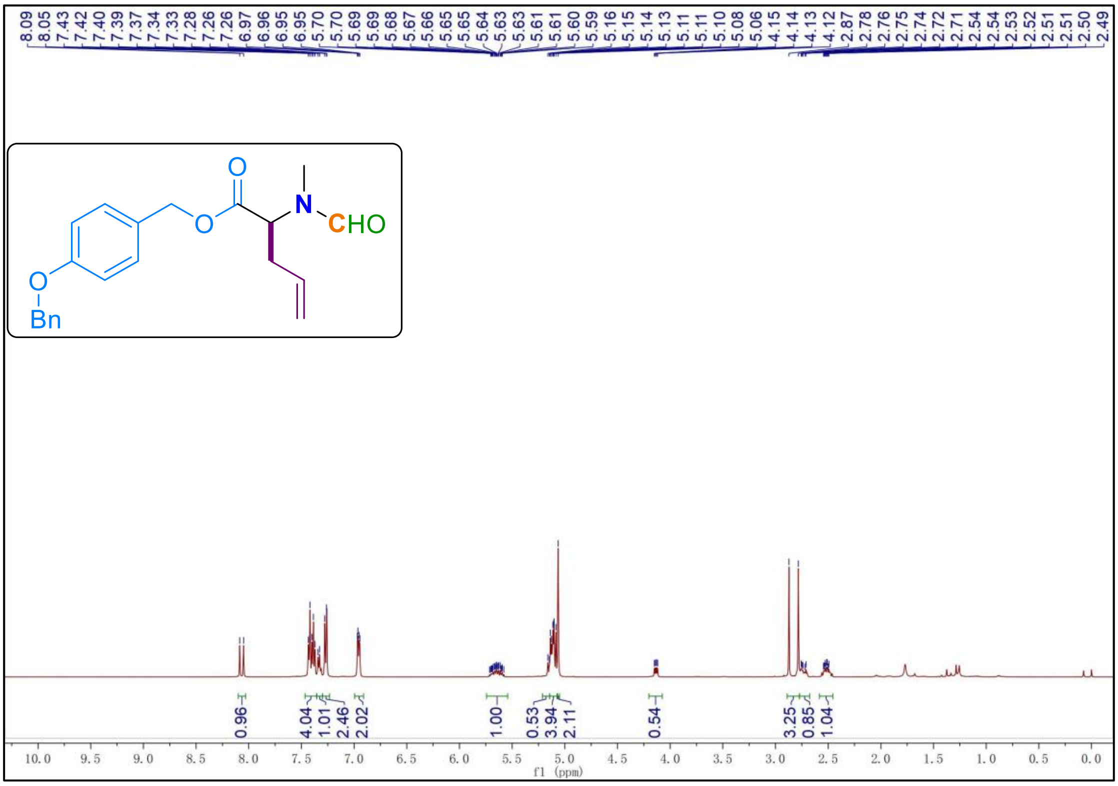

^{13}C NMR (126 MHz, Chloroform-*d*)

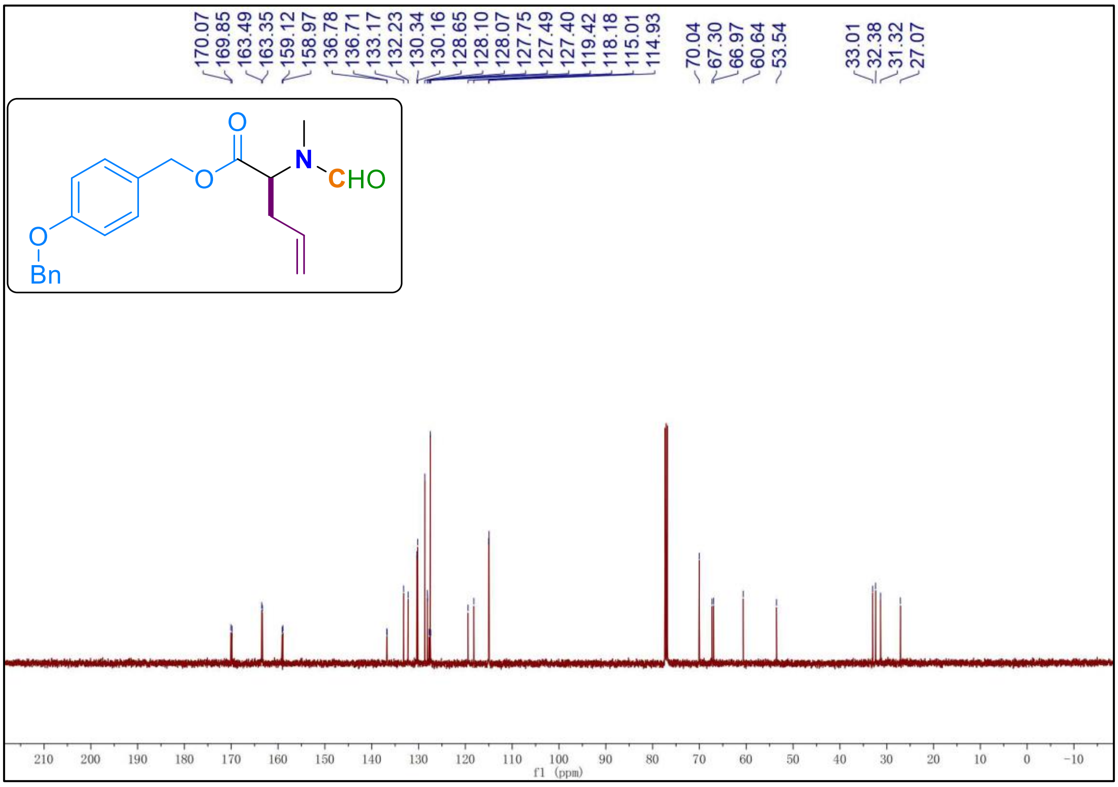

[1,1'-biphenyl]-4-ylmethyl 2-(*N*-methylformamido)pent-4-enoate (3k)

^{1}H NMR (500 MHz, Chloroform-*d*)

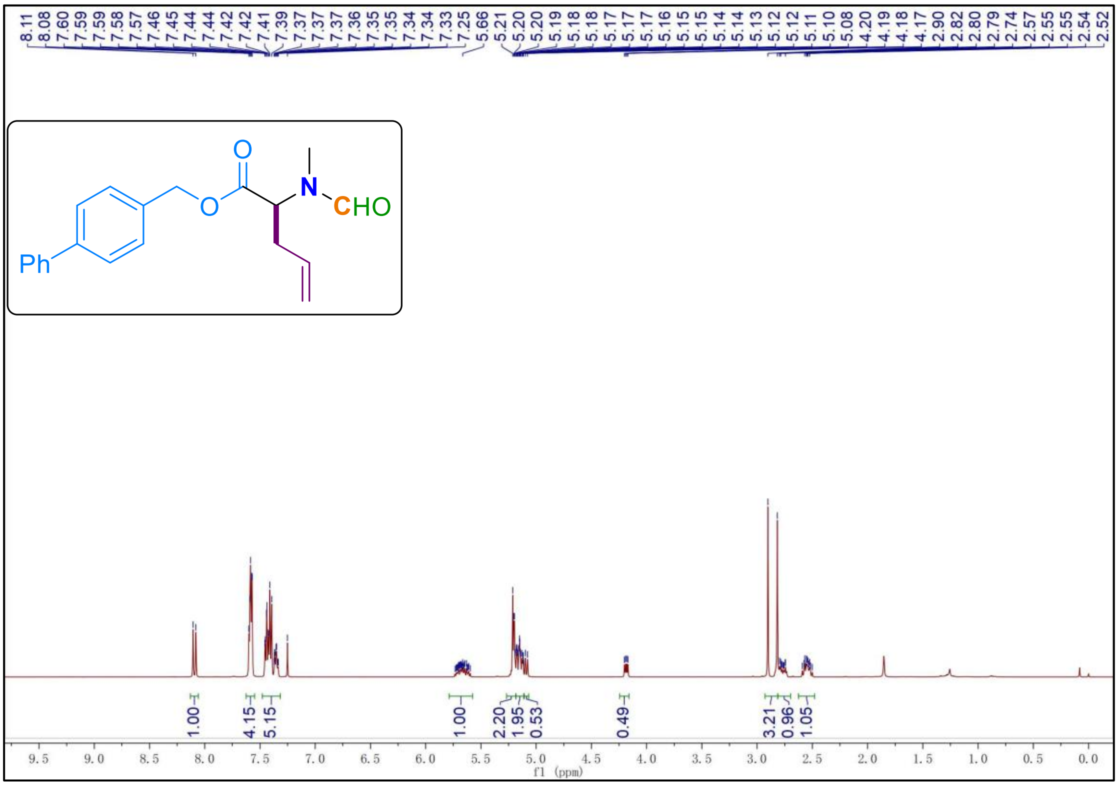

^{13}C NMR (126 MHz, Chloroform-*d*)

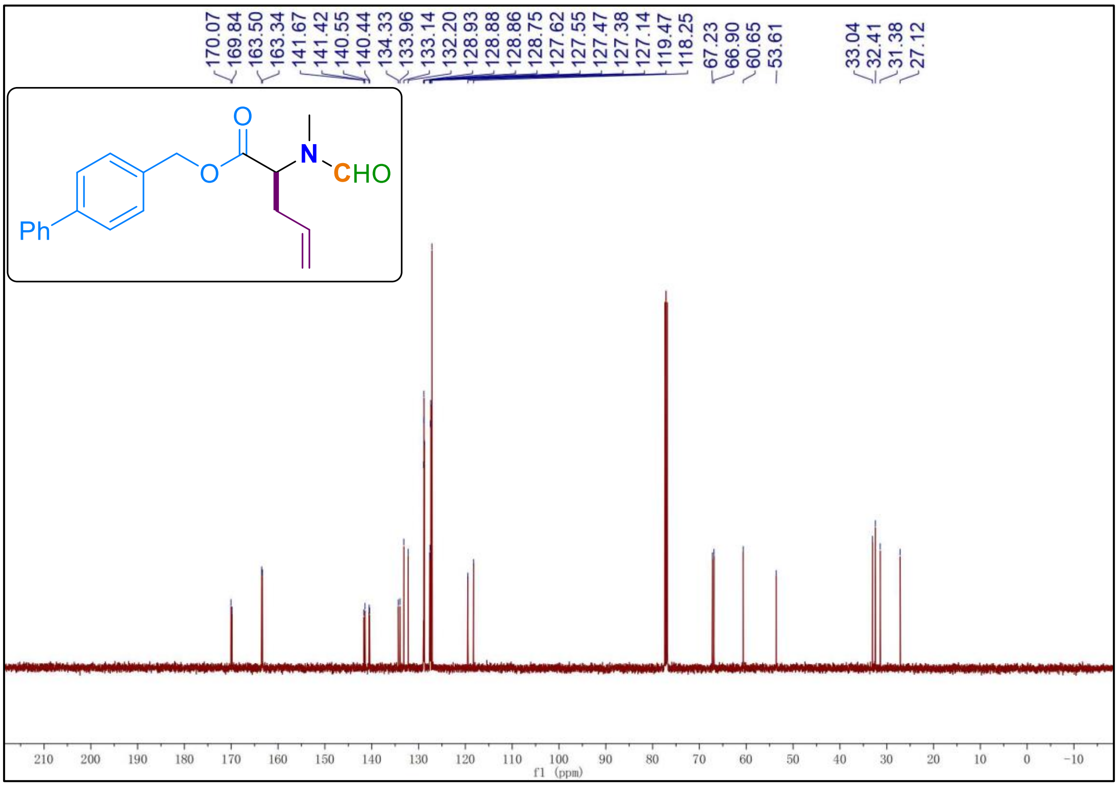

2-iodobenzyl 2-(*N*-methylformamido)pent-4-enoate (3l)

^{1}H NMR (500 MHz, Chloroform-*d*)

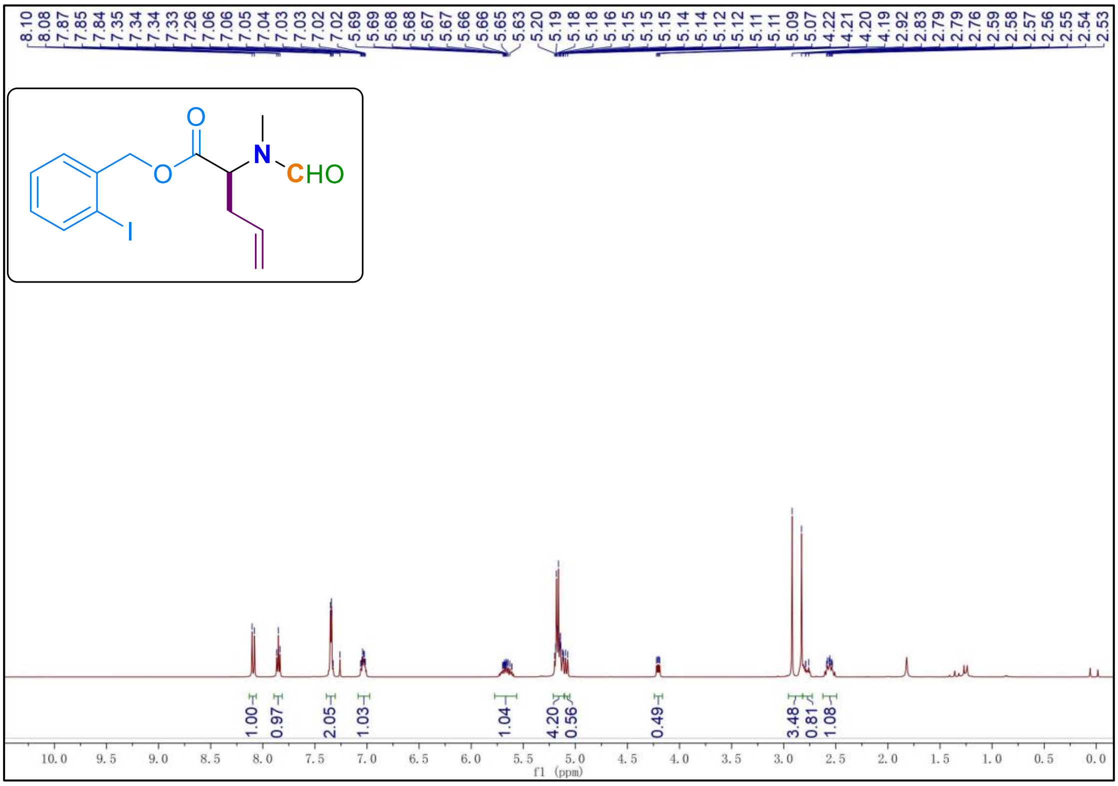

^{13}C NMR (126 MHz, Chloroform-*d*)

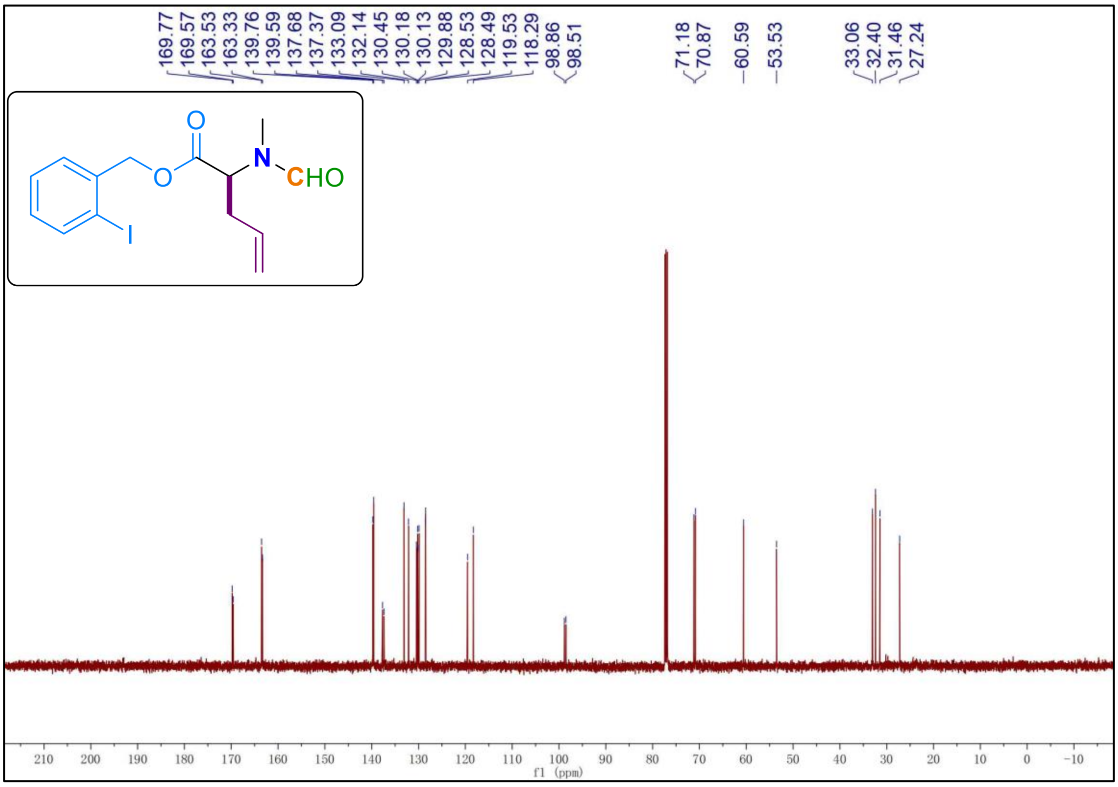

3,5-dichlorobenzyl 2-(*N*-methylformamido)pent-4-enoate (3m)

^{1}H NMR (500 MHz, Chloroform-*d*)

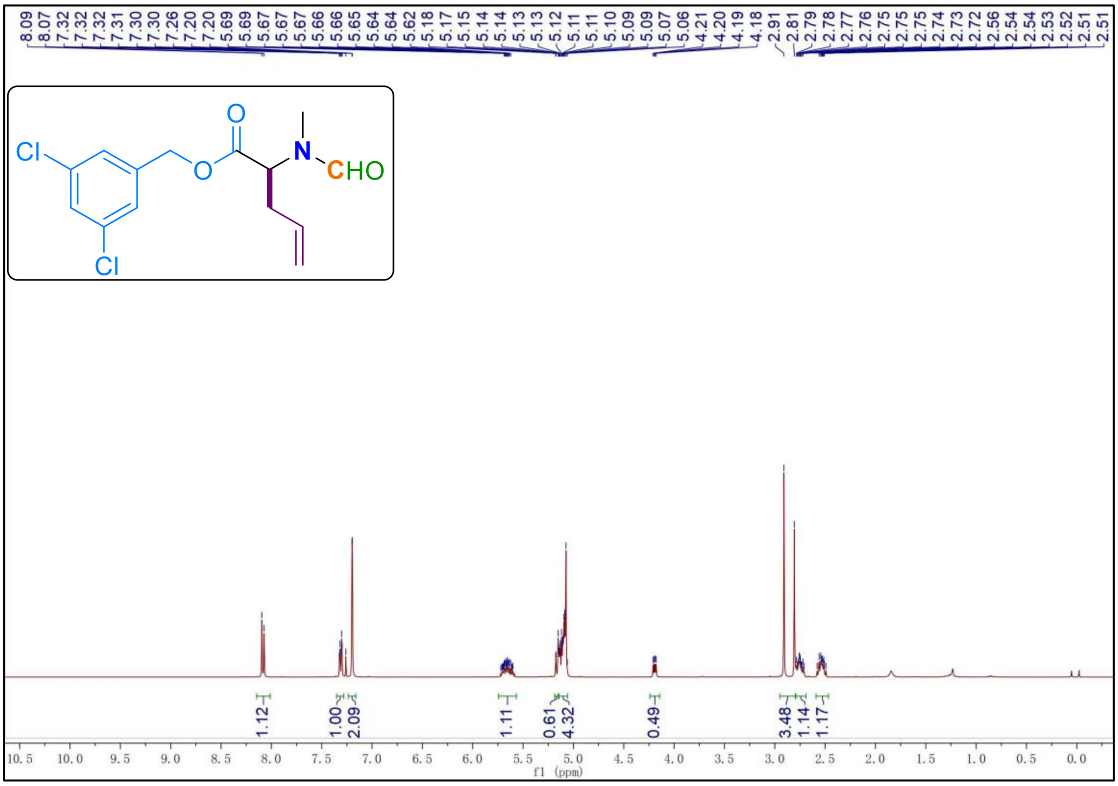

^{13}C NMR (126 MHz, Chloroform-*d*)

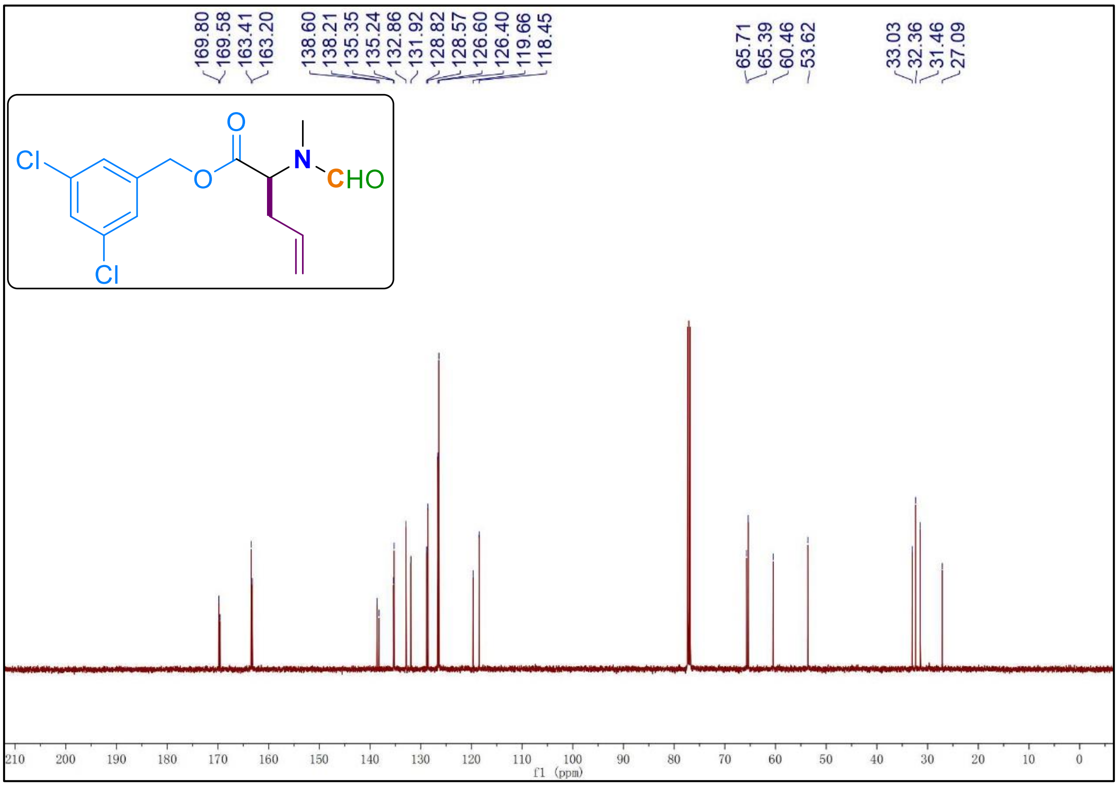

cinnamyl 2-(*N*-methylformamido)pent-4-enoate (3n)

^{1}H NMR (500 MHz, Chloroform-*d*)

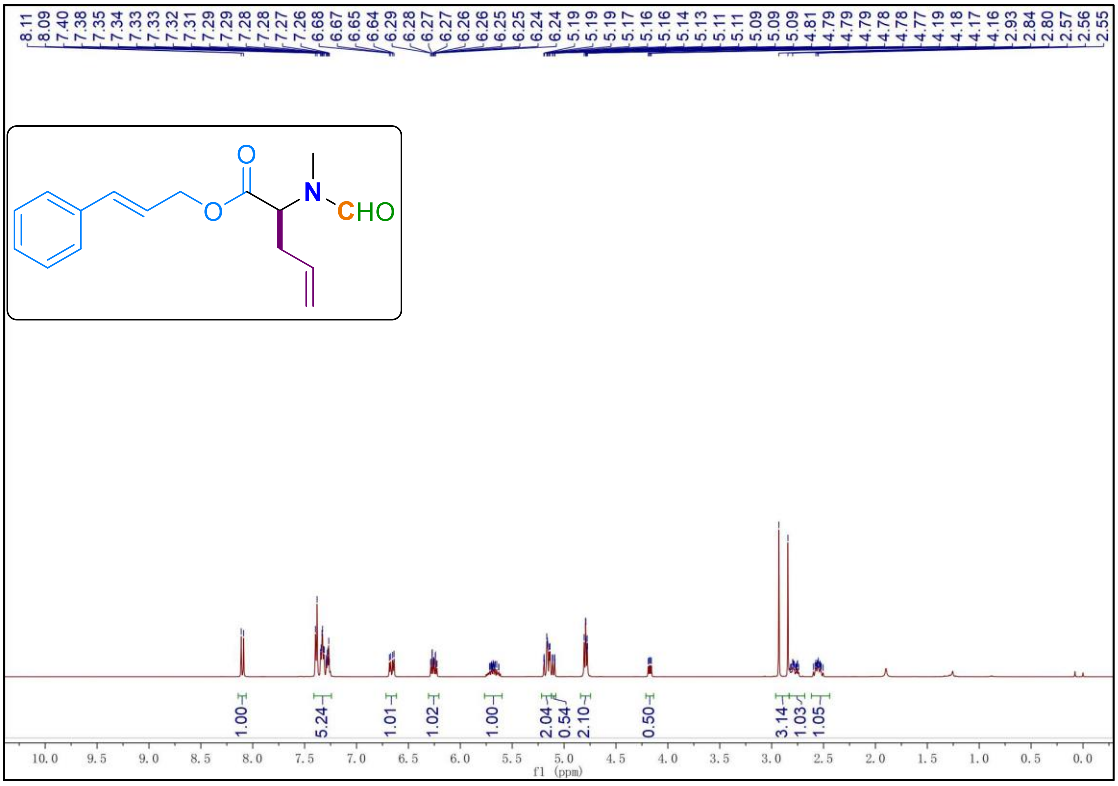

^{13}C NMR (126 MHz, Chloroform-*d*)

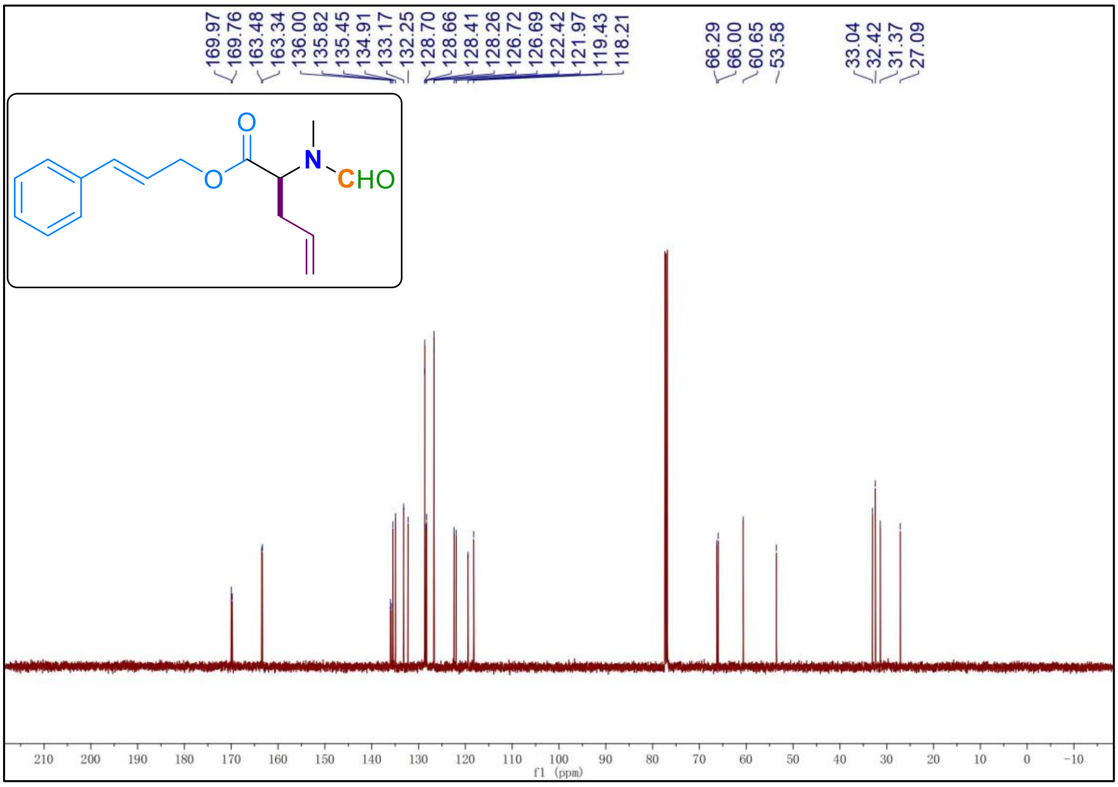

2-(benzyloxy)ethyl 2-(*N*-methylformamido)pent-4-enoate (3o)

^{1}H NMR (500 MHz, Chloroform-*d*)

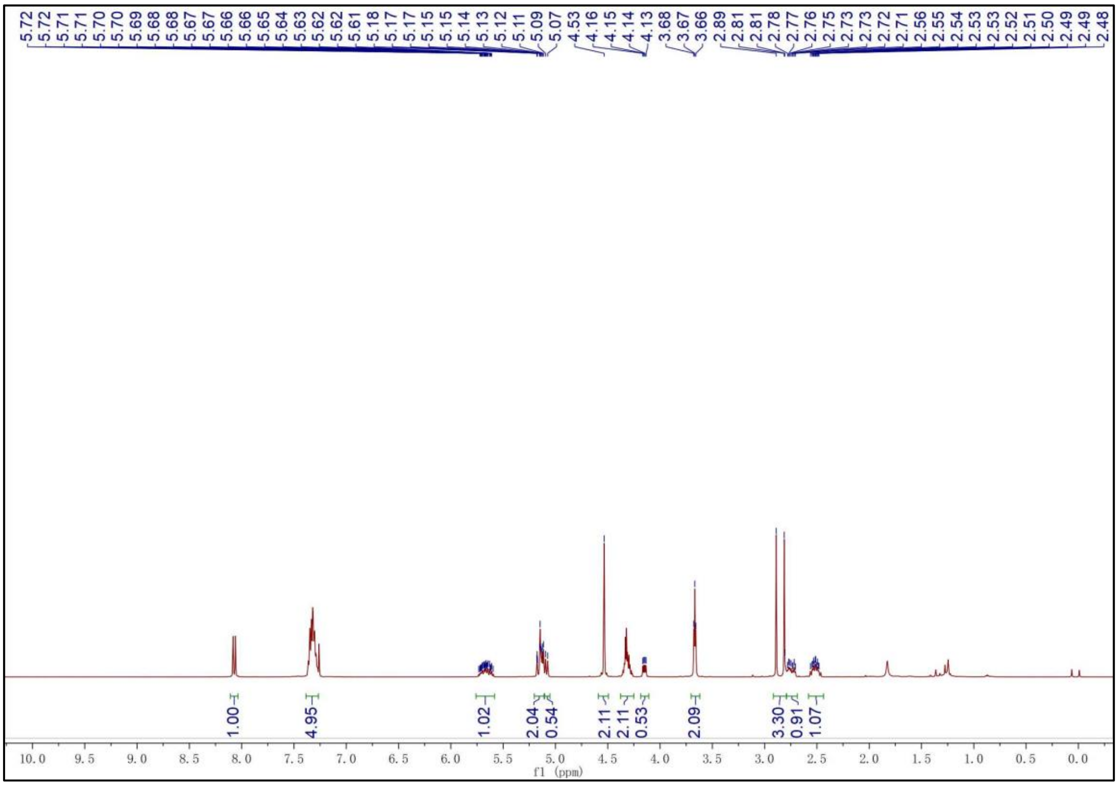

^{13}C NMR (126 MHz, Chloroform-*d*)

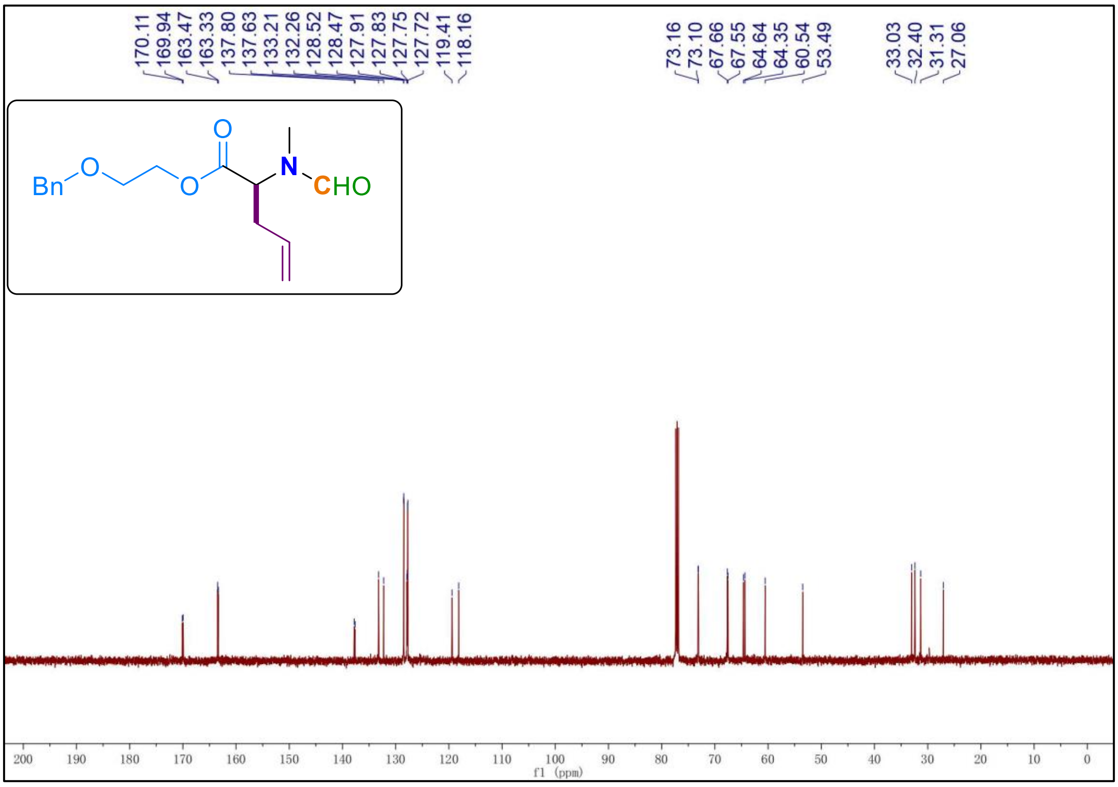

N-(1-cyclopropyl-1-oxopent-4-en-2-yl)-*N*-methylformamide (3p)

^{1}H NMR (500 MHz, Chloroform-*d*)

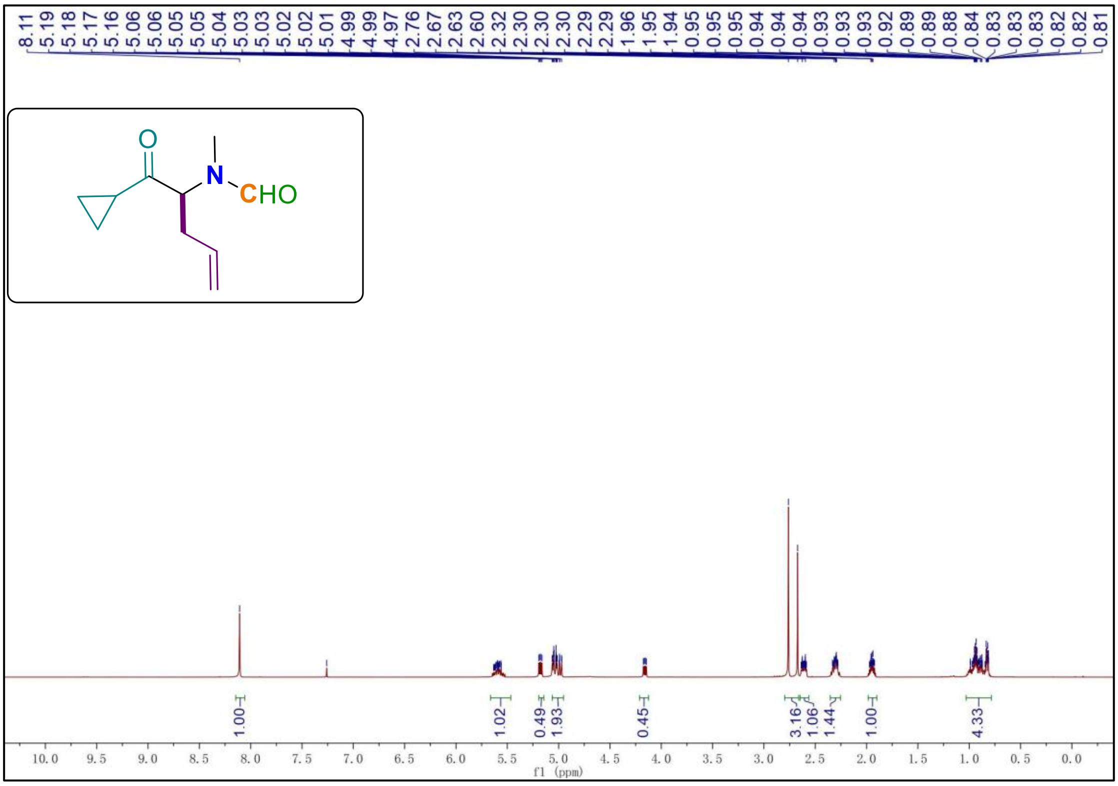

^{13}C NMR (126 MHz, Chloroform-*d*)

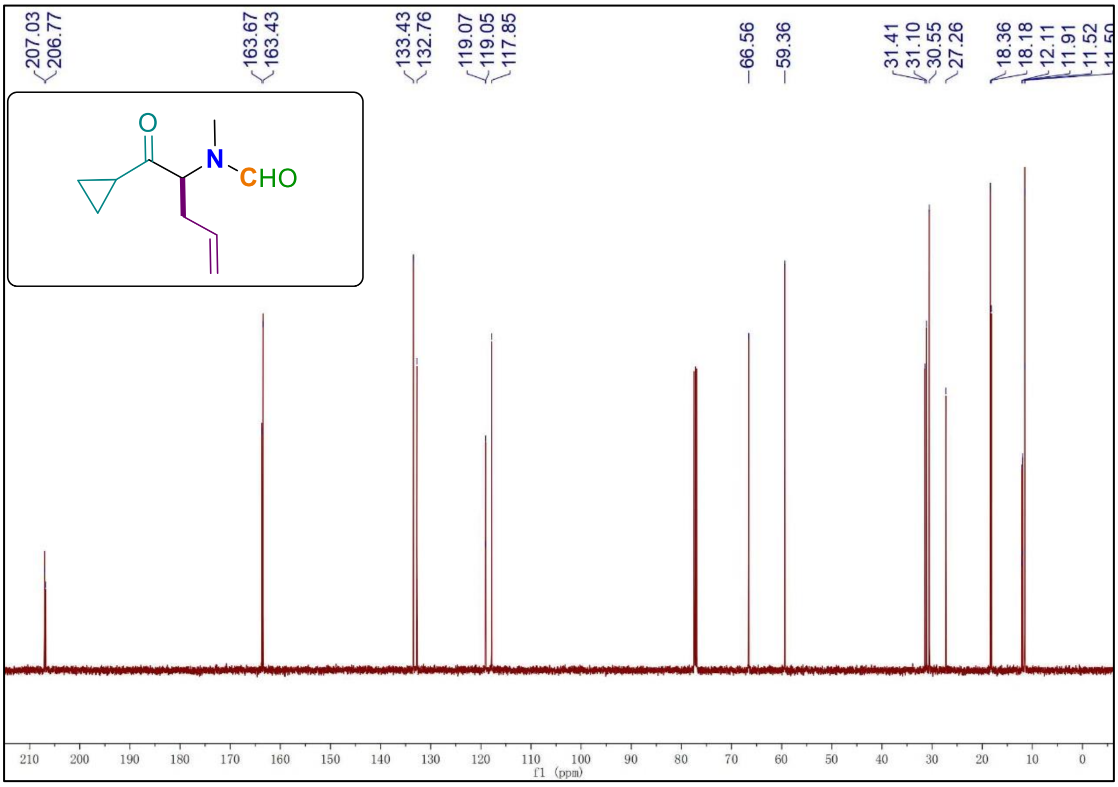

N-(6,6-dimethyl-5-oxohept-1-en-4-yl)-*N*-methylformamide (3q)

^{1}H NMR (500 MHz, Chloroform-*d*)

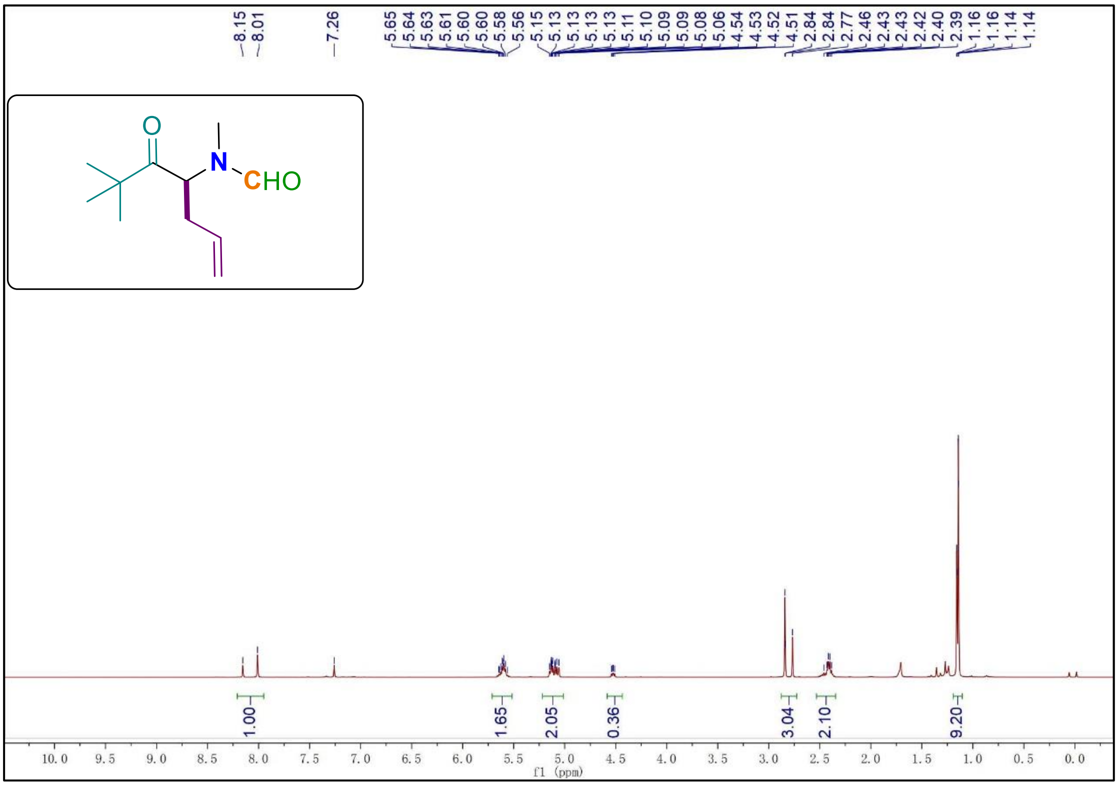

^{13}C NMR (126 MHz, Chloroform-*d*)

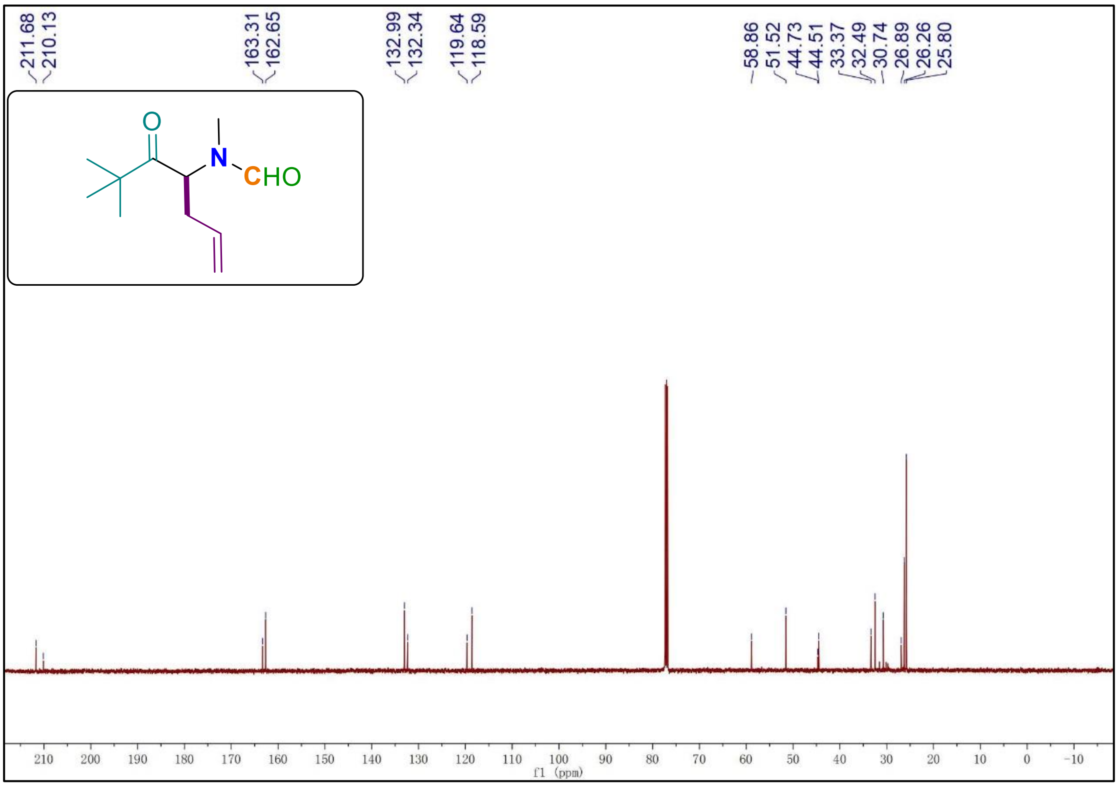

N-(adamantan-1-yl)-1-oxopent-4-en-2-yl)-*N*-methylformamide (3r)

^{1}H NMR (500 MHz, Chloroform-*d*)

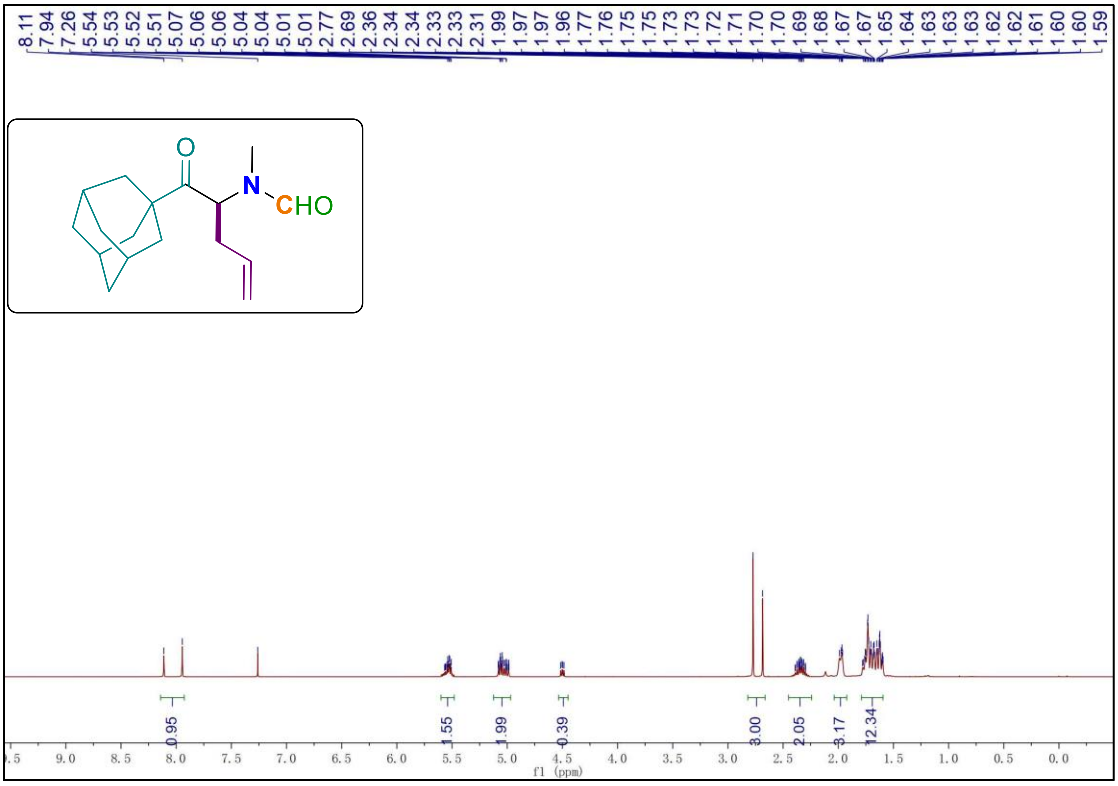

^{13}C NMR (126 MHz, Chloroform-*d*)

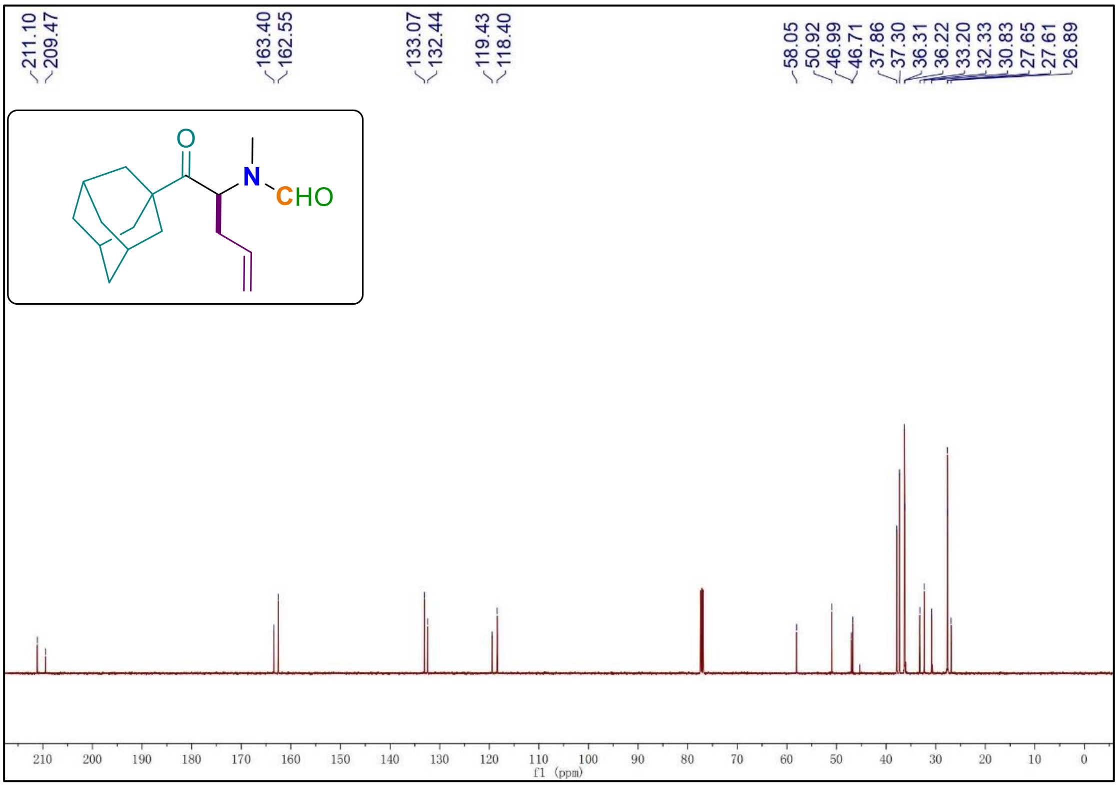

N-methyl-*N*-(1-oxo-1-phenylpent-4-en-2-yl)formamide (3s)

^{1}H NMR (500 MHz, Chloroform-*d*)

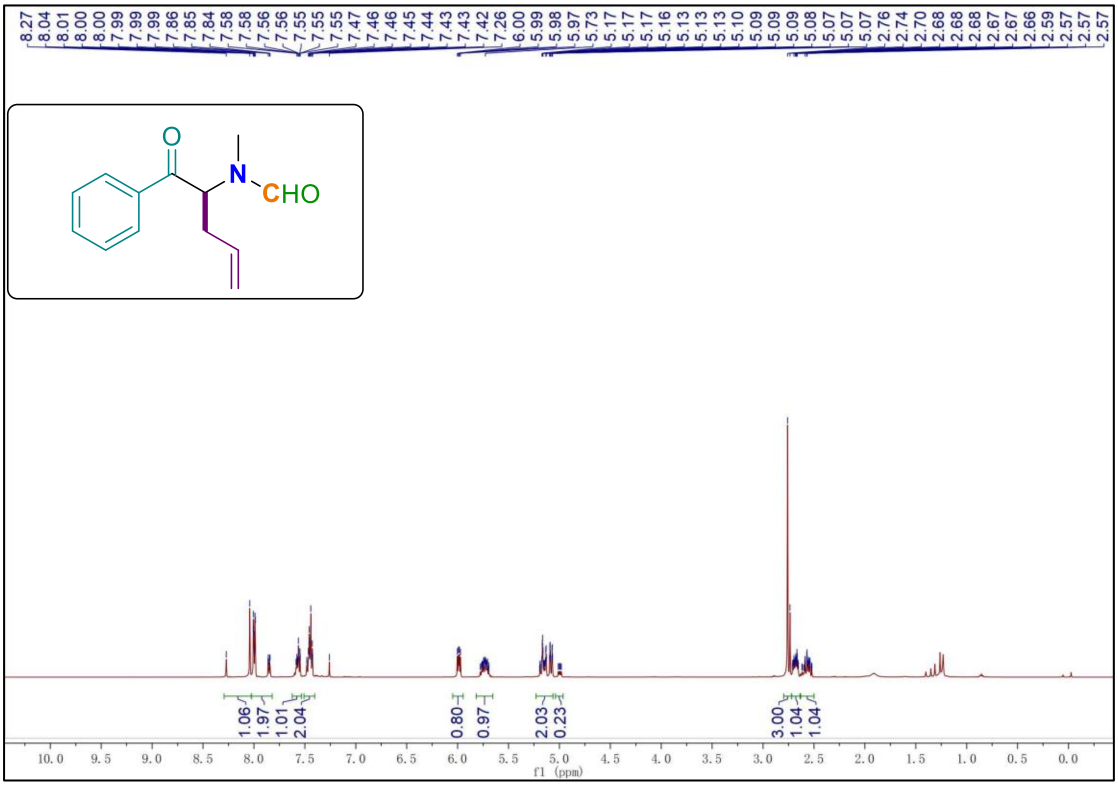

^{13}C NMR (126 MHz, Chloroform-*d*)

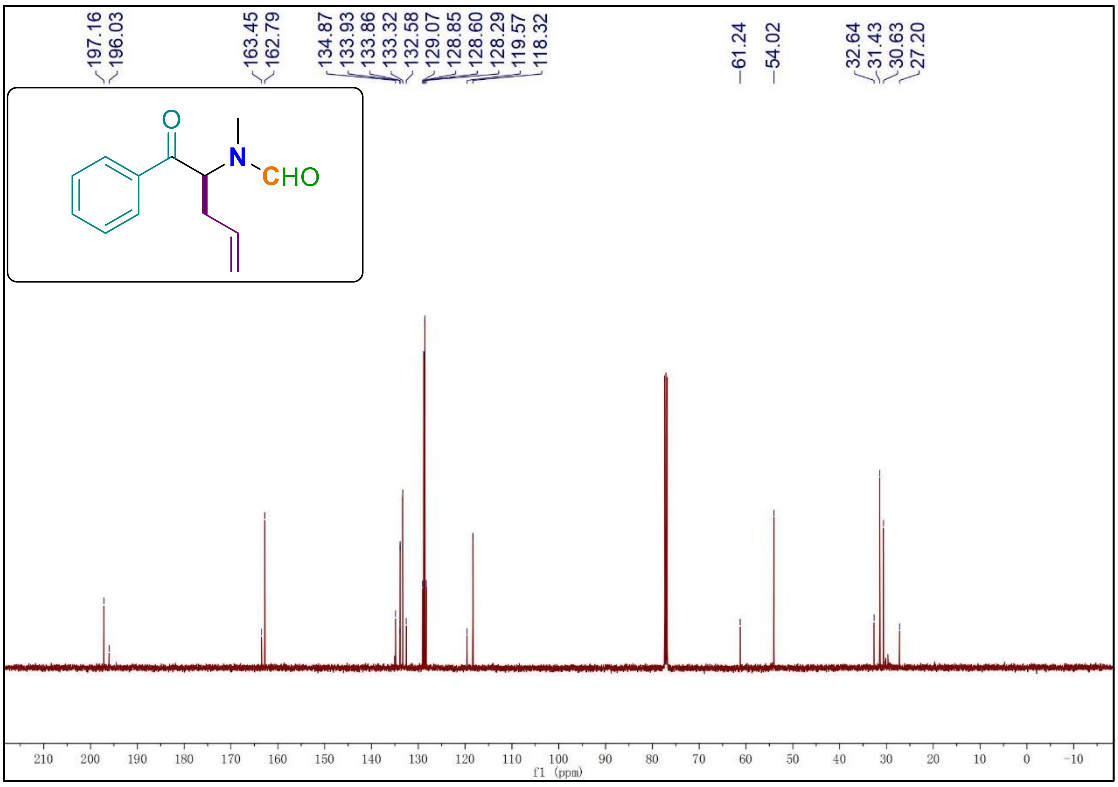

N-(1-([1,1'-biphenyl]-4-yl)-1-oxopent-4-en-2-yl)-*N*-methylformamide (3t)

^{1}H NMR (500 MHz, Chloroform-*d*)

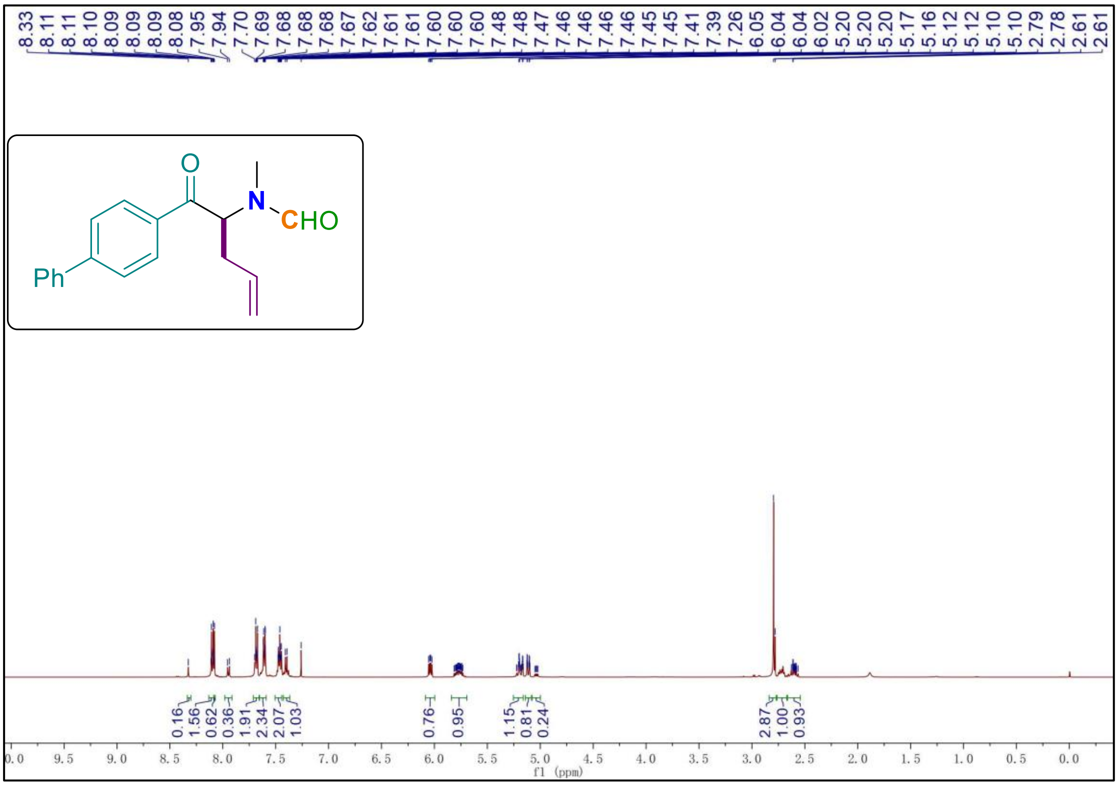

^{13}C NMR (126 MHz, Chloroform-*d*)

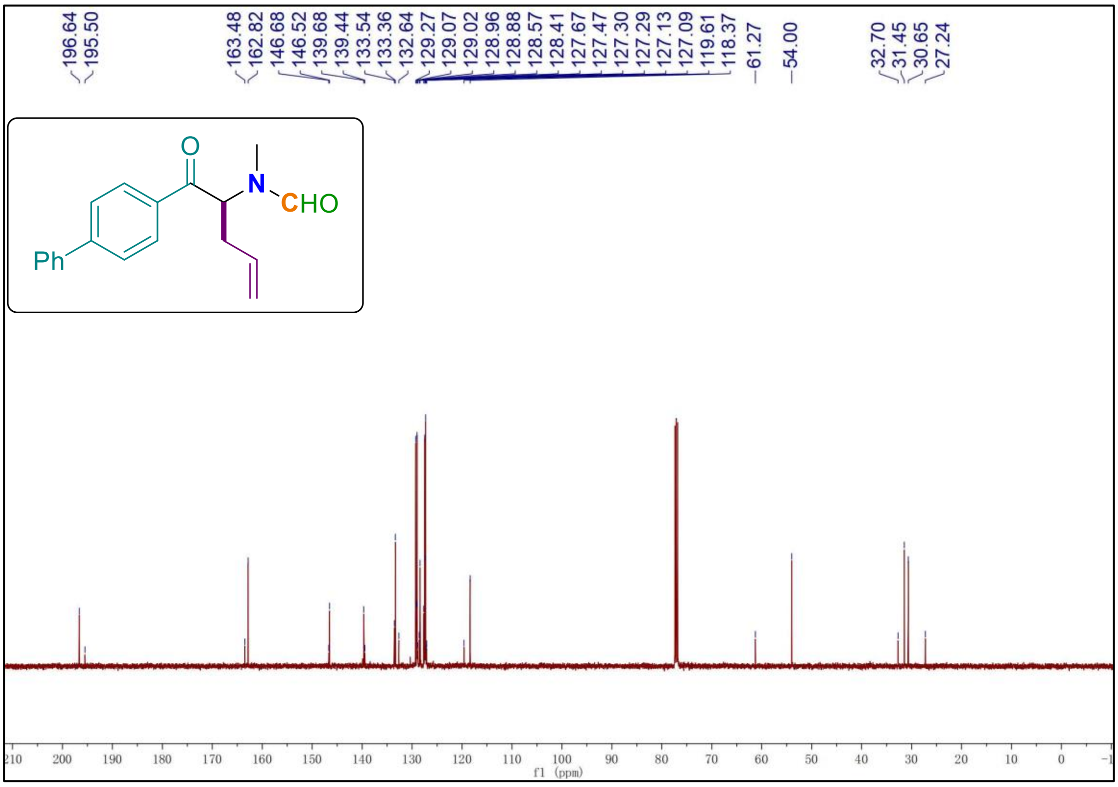

N-(1-(4-methoxyphenyl)-1-oxopent-4-en-2-yl)-*N*-methylformamide (3u)

^{1}H NMR (500 MHz, Chloroform-*d*)

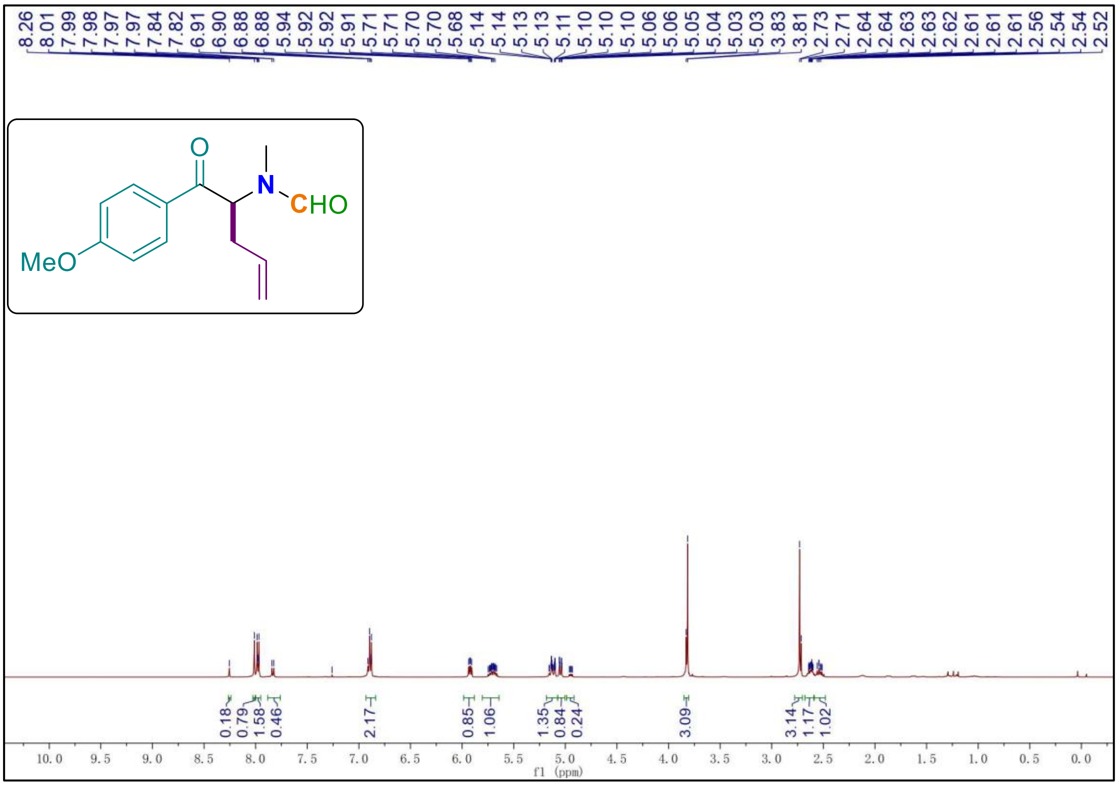

^{13}C NMR (126 MHz, Chloroform-*d*)

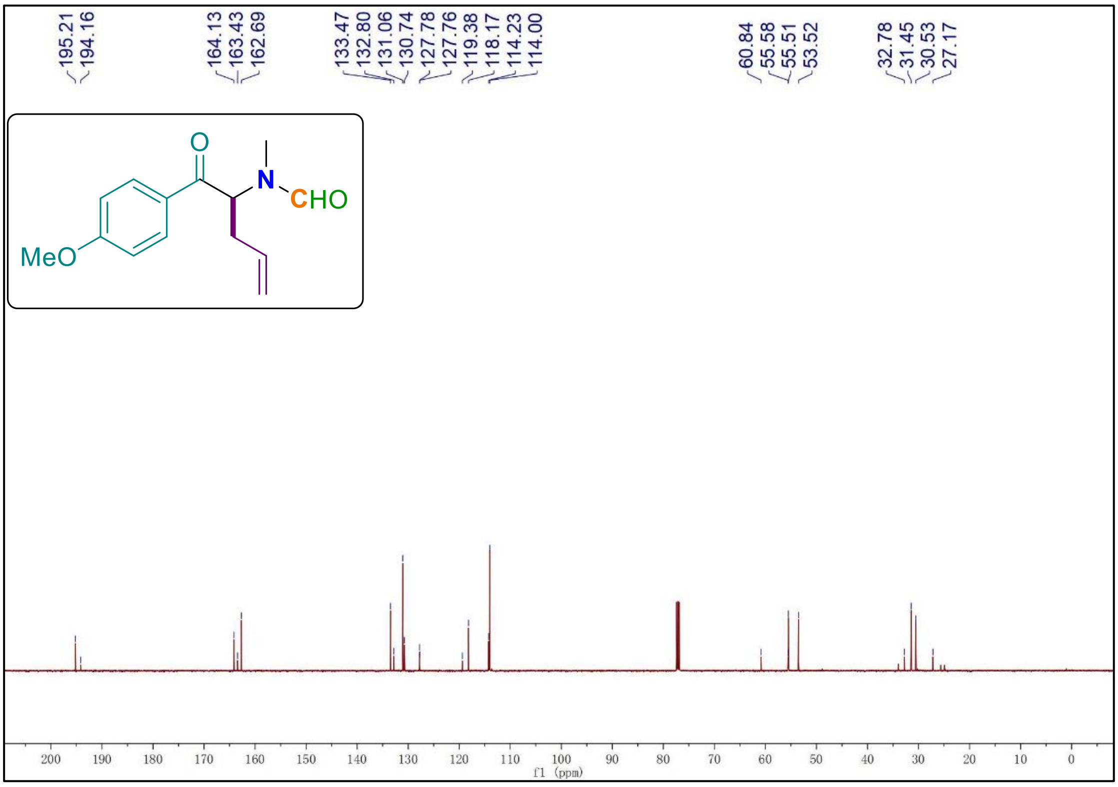

N-(1-(4-bromophenyl)-1-oxopent-4-en-2-yl)-*N*-methylformamide (3v)

^{1}H NMR (500 MHz, Chloroform-*d*)

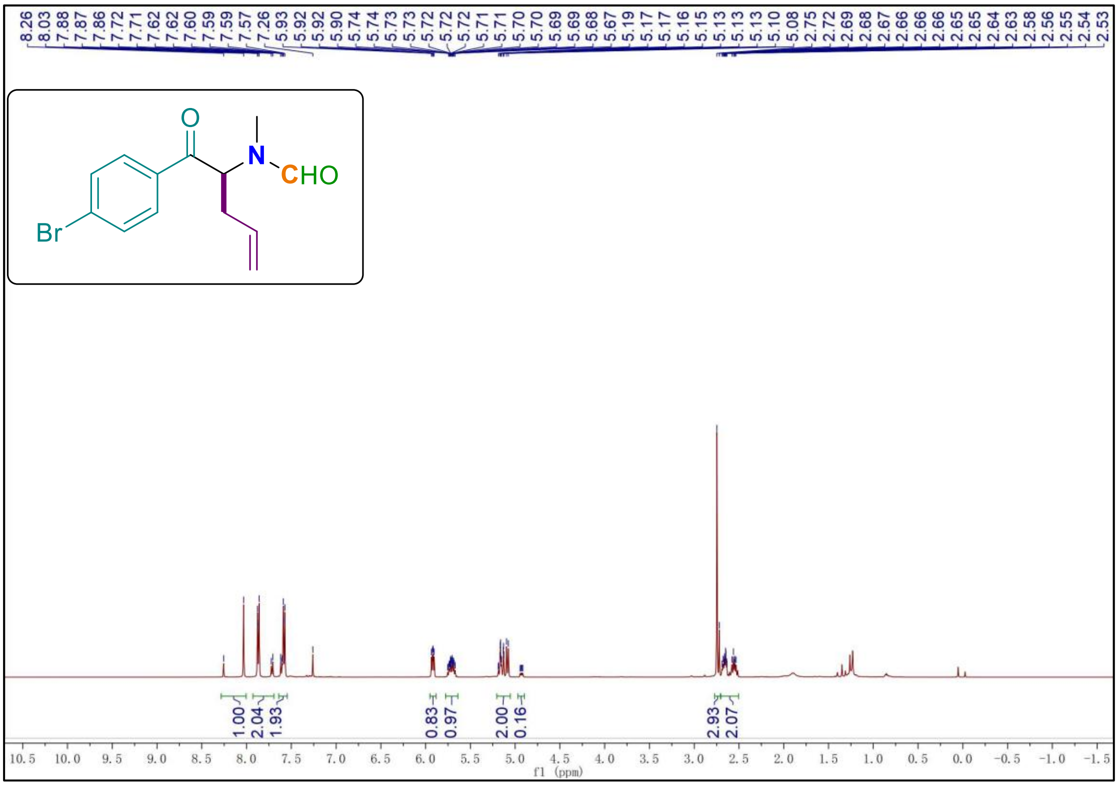

^{13}C NMR (126 MHz, Chloroform-*d*)

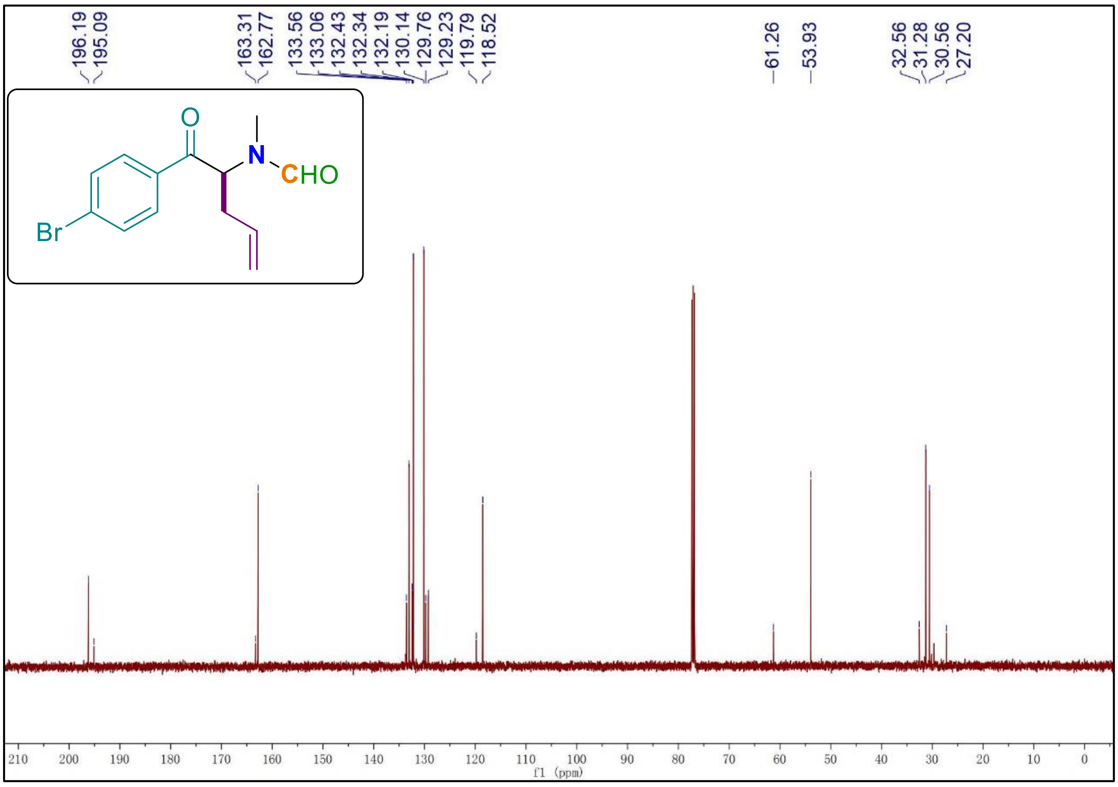

N-(1-(4-hydroxyphenyl)-1-oxopent-4-en-2-yl)-*N*-methylformamide (3w)

^{1}H NMR (500 MHz, Chloroform-*d*)

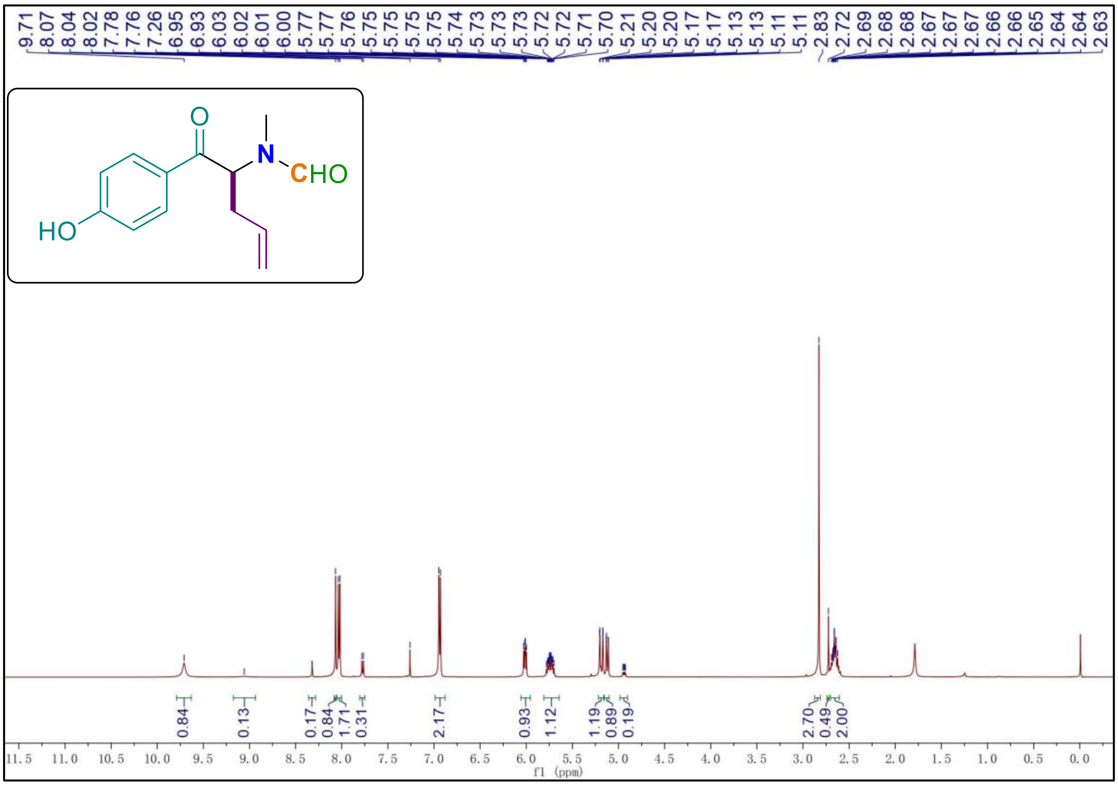

^{13}C NMR (126 MHz, Chloroform-*d*)

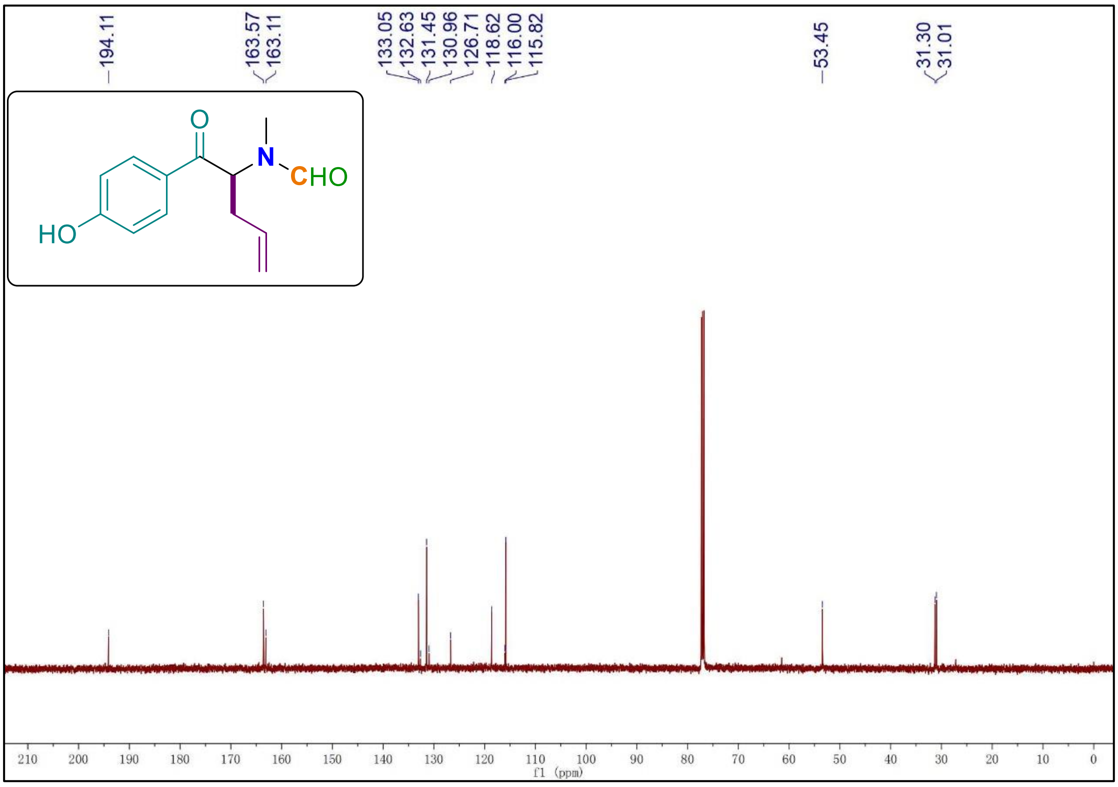

N-(1-(3-methoxyphenyl)-1-oxopent-4-en-2-yl)-*N*-methylformamide (3x)

^{1}H NMR (500 MHz, Chloroform-*d*)

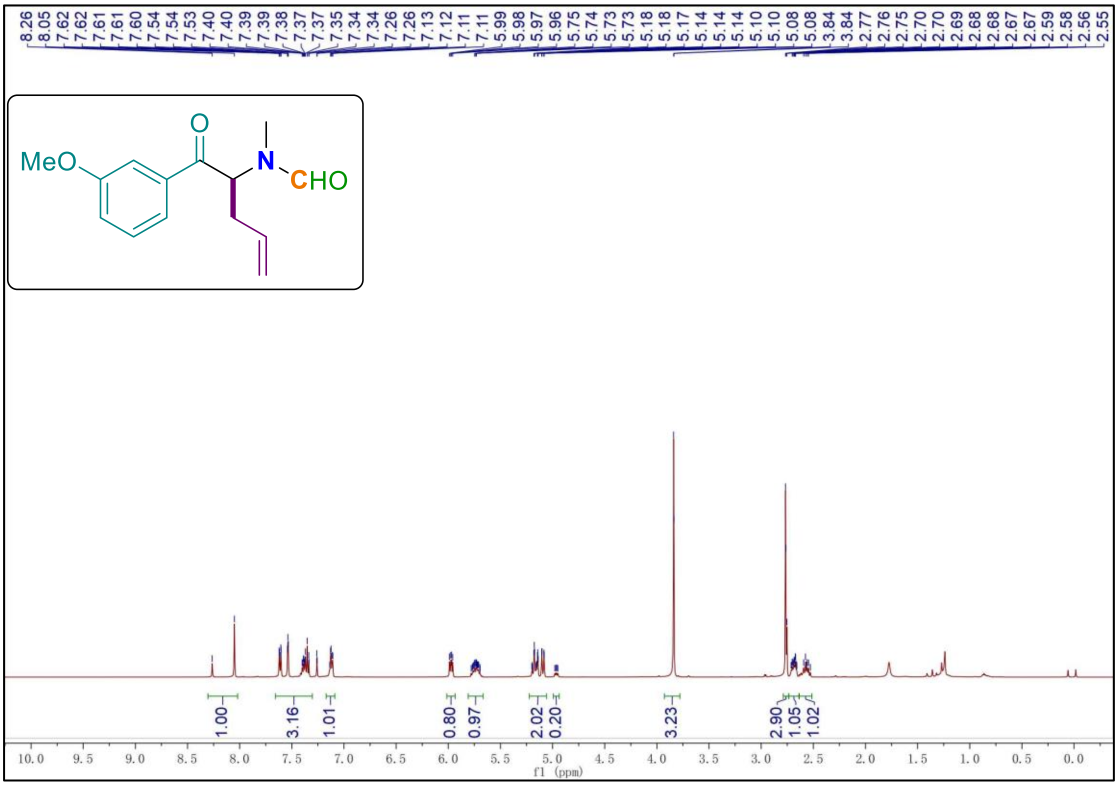

^{13}C NMR (126 MHz, Chloroform-*d*)

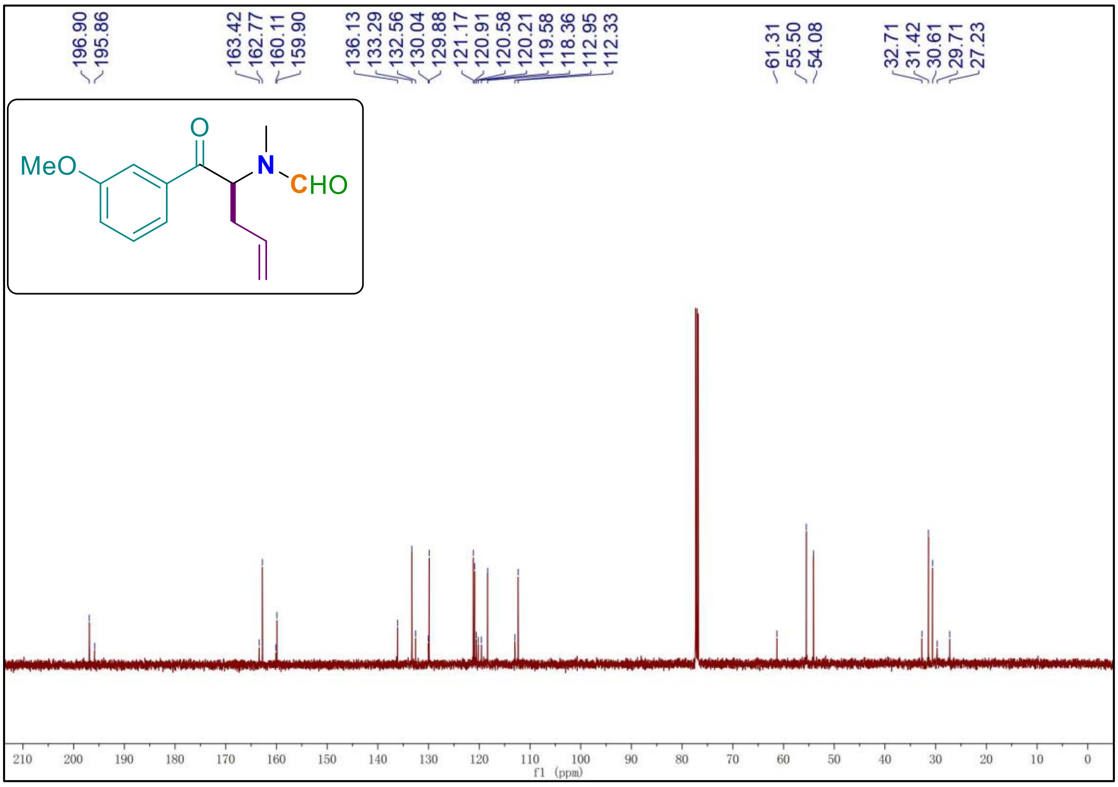

N-methyl-*N*-(1-oxo-1-(thiophen-2-yl)pent-4-en-2-yl)formamide (3y)

^{1}H NMR (500 MHz, Chloroform-*d*)

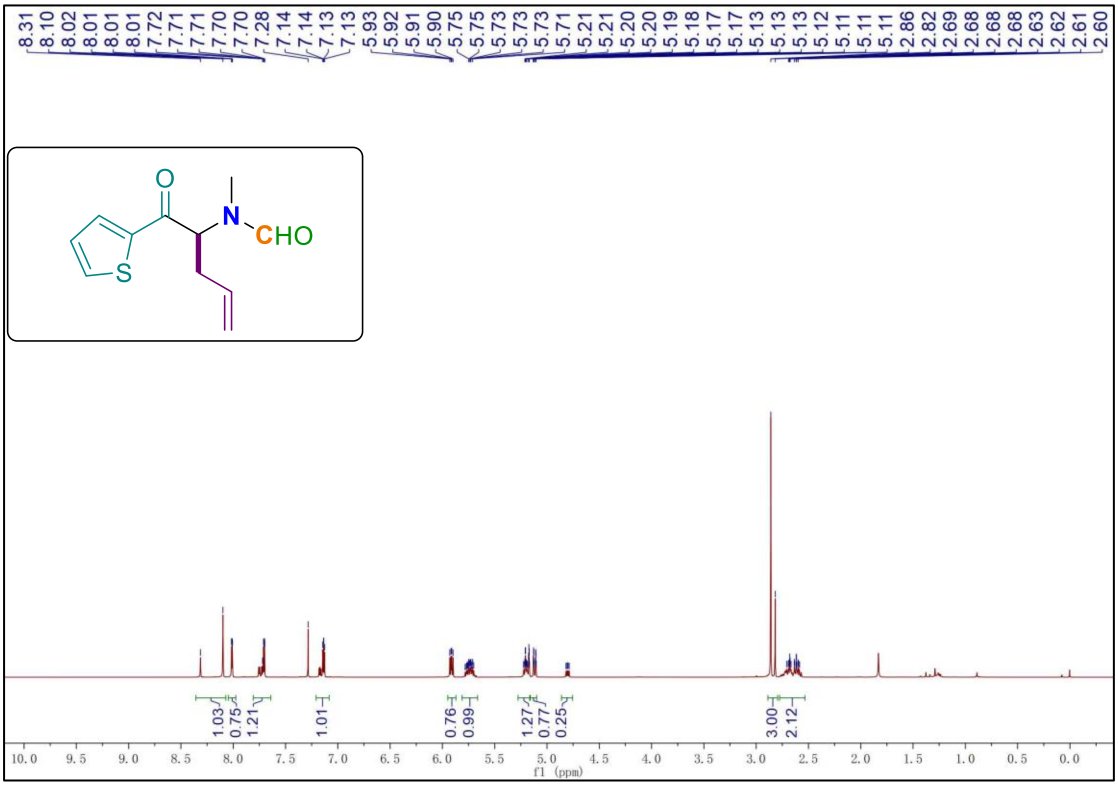

^{13}C NMR (126 MHz, Chloroform-*d*)

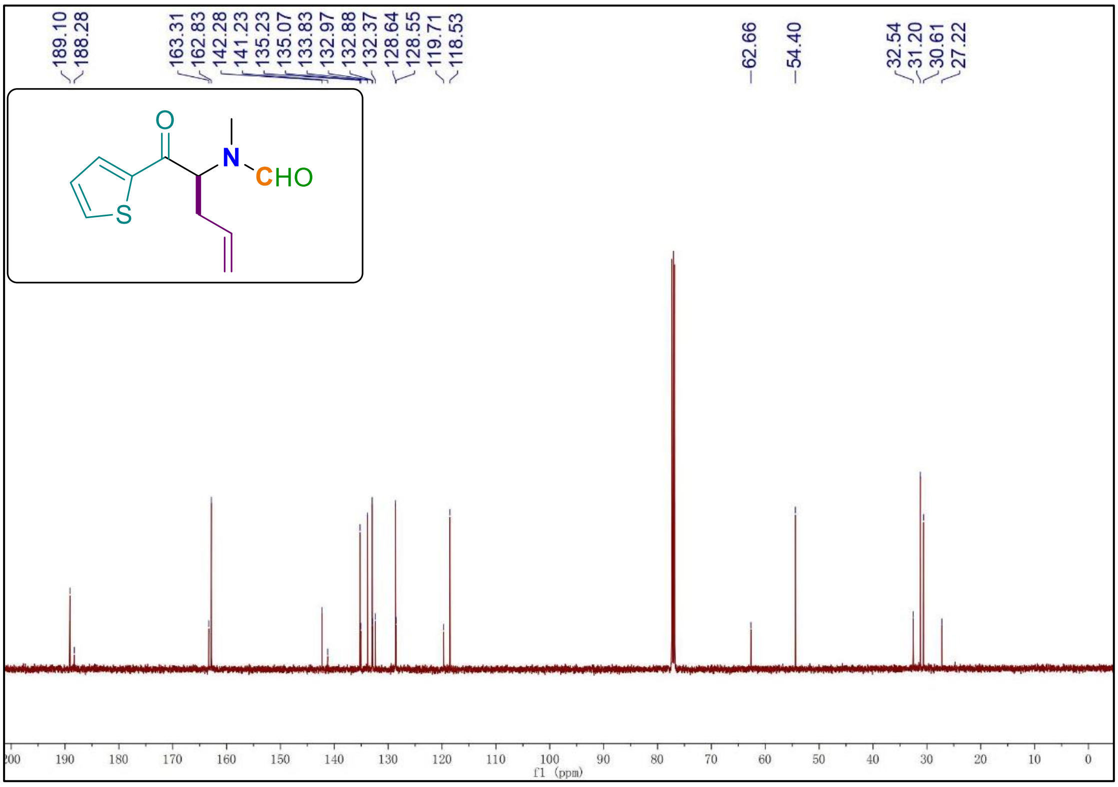

N-methyl-*N*-(1-(naphthalen-2-yl)-1-oxopent-4-en-2-yl)formamide (3z)

^{1}H NMR (500 MHz, Chloroform-*d*)

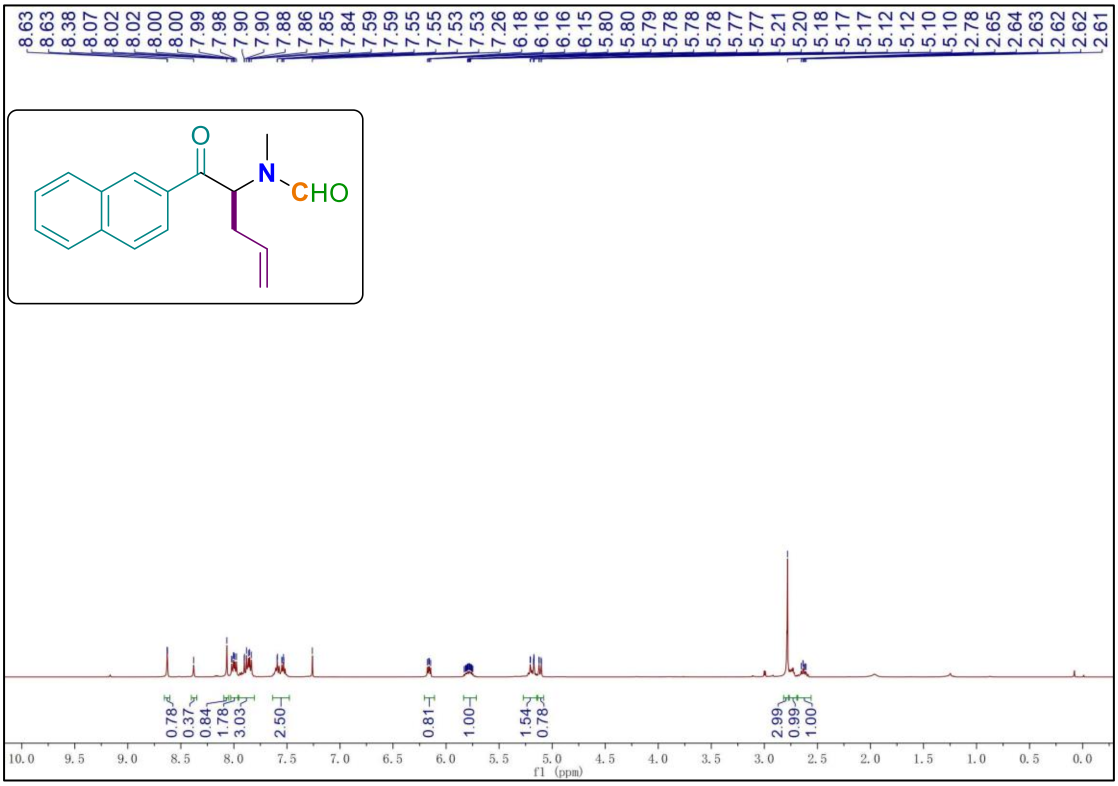

^{13}C NMR (126 MHz, Chloroform-*d*)

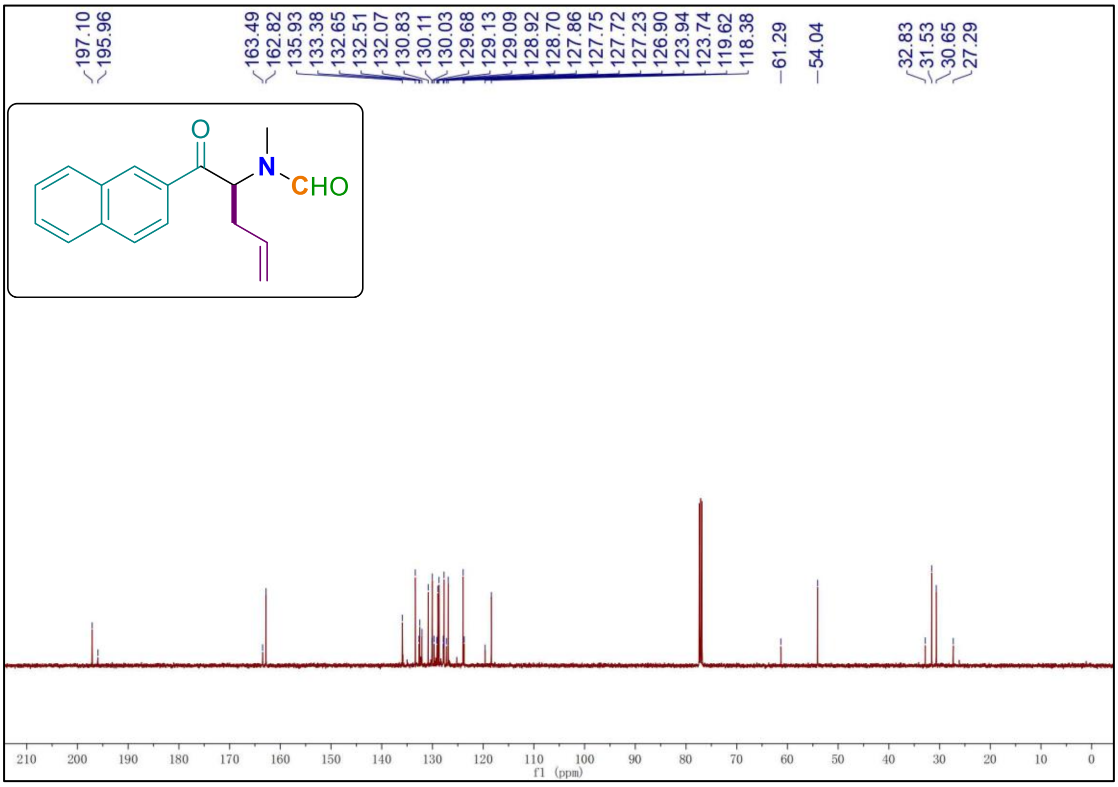

***N*-(1-cyanobut-3-en-1-yl)-N-methylformamide (3za)**

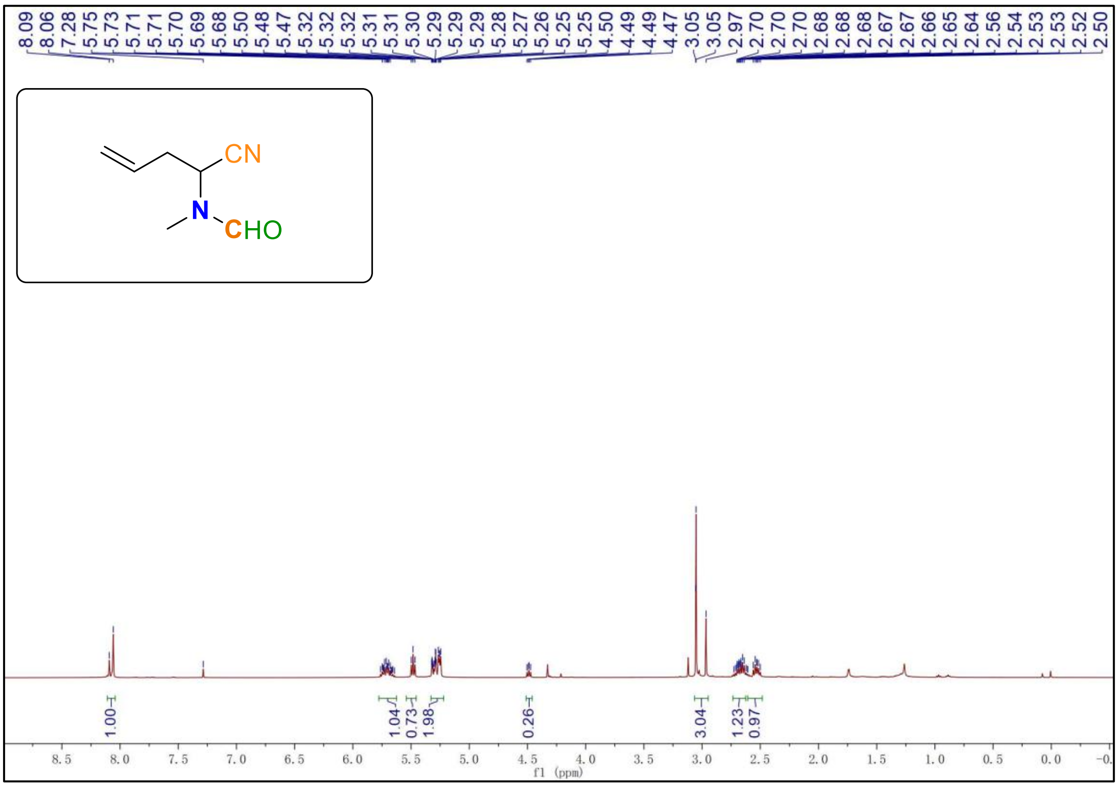

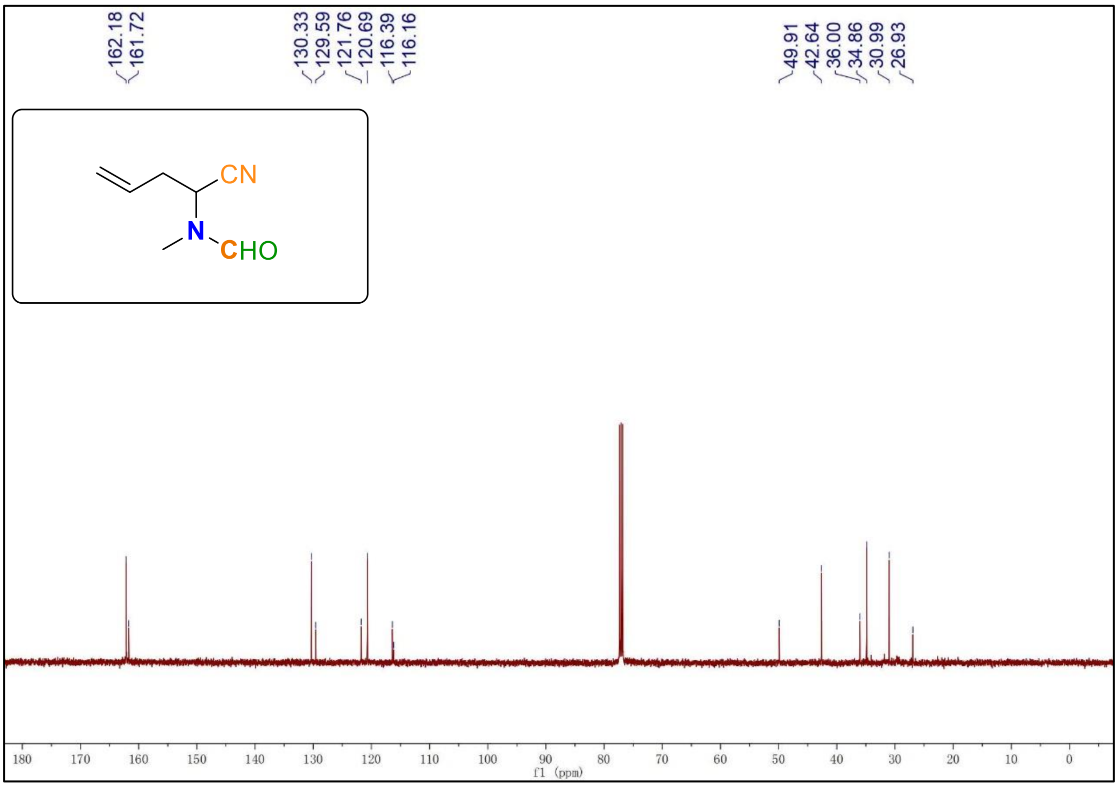

diethyl (1-(N-methylformamido)but-3-en-1-yl)phosphonate (3zb)

^{1}H NMR (500 MHz, Chloroform-*d*)

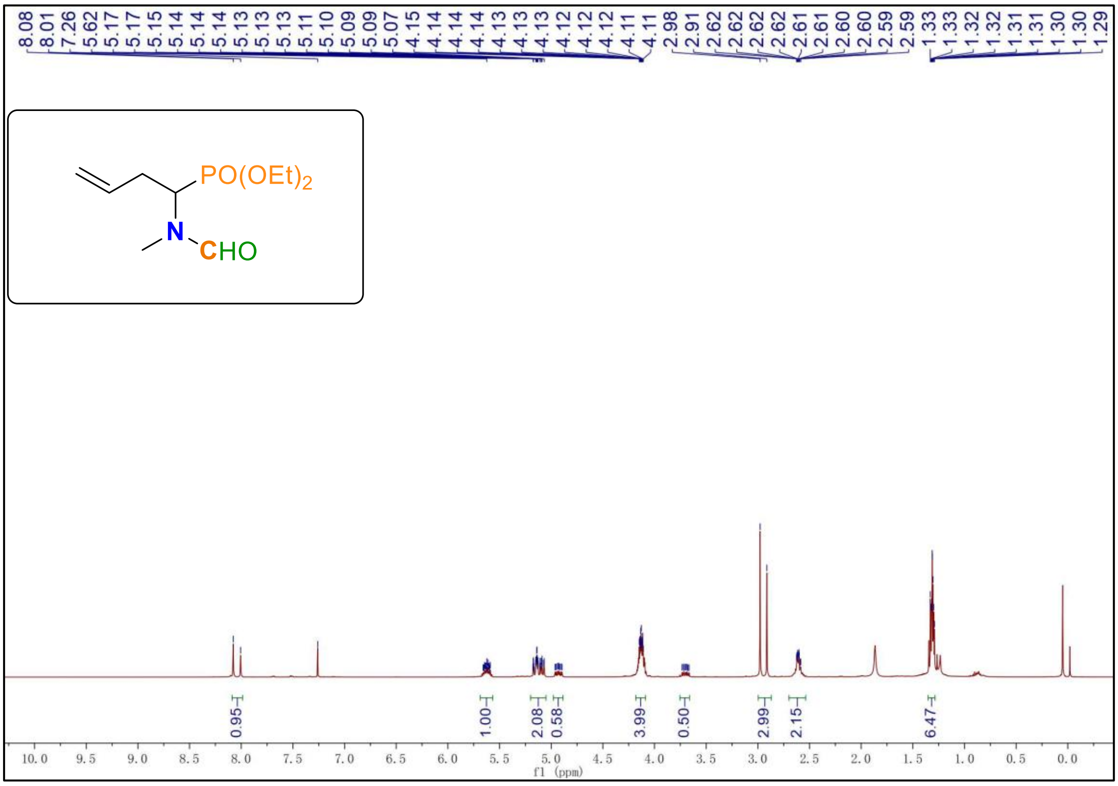

^{13}C NMR (126 MHz, Chloroform-*d*)

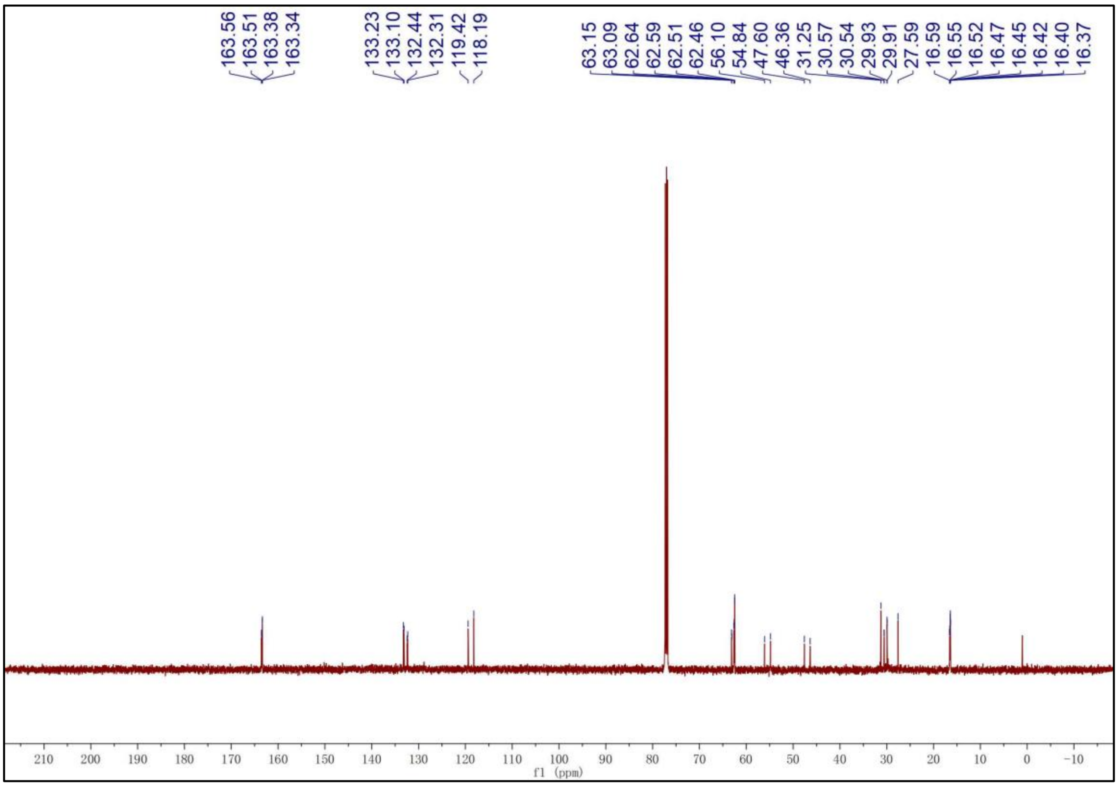

^{31}P NMR (202 MHz, Chloroform-*d*)

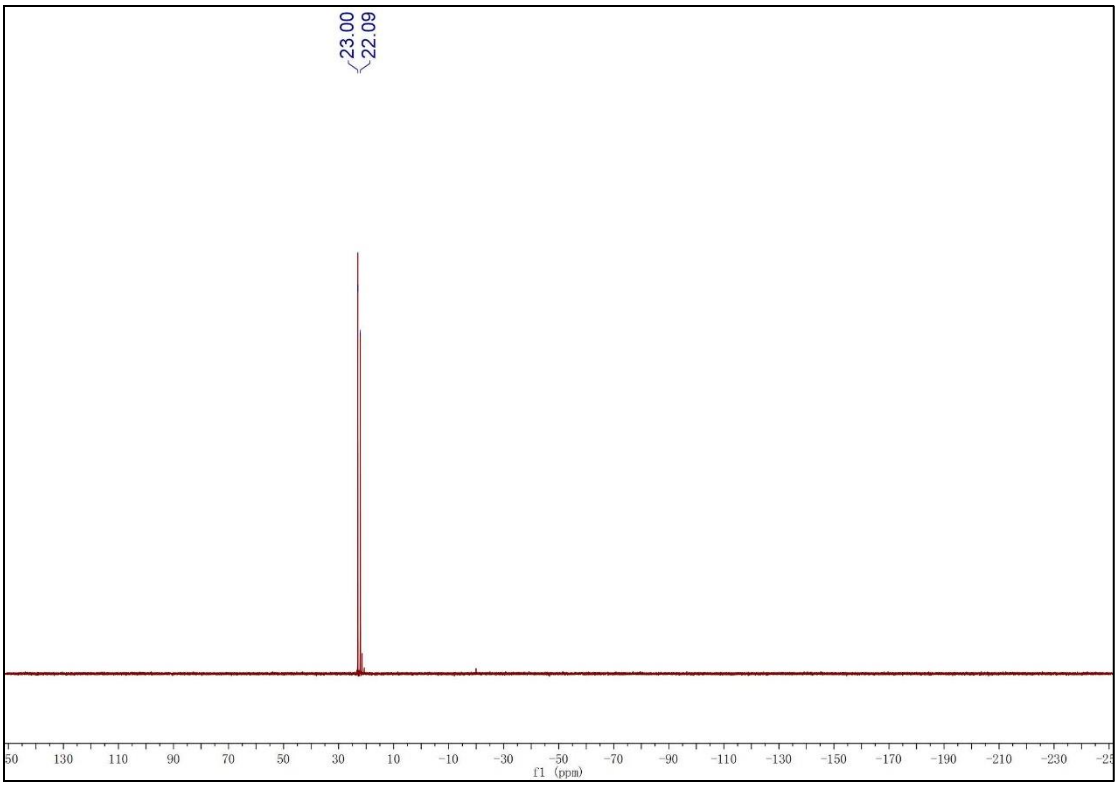

methyl 2-methyl-2-(*N*-methylformamido)pent-4-enoate (6a)

^{1}H NMR (500 MHz, Chloroform-*d*)

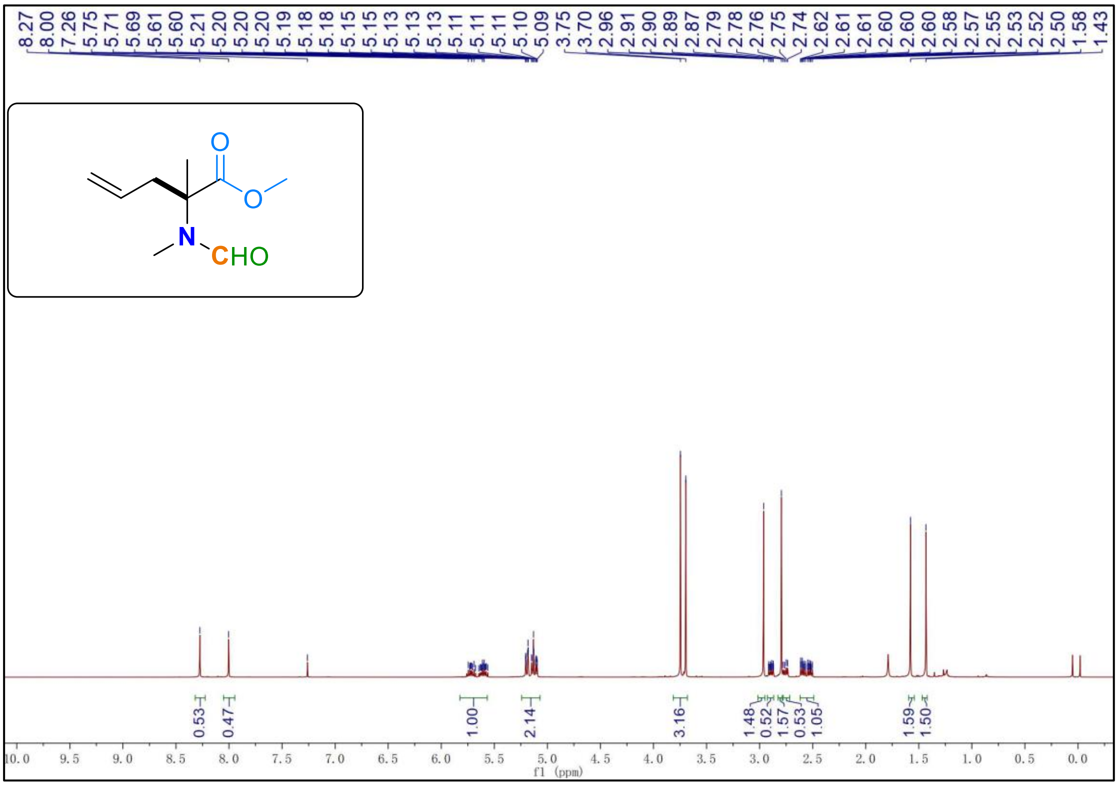

^{13}C NMR (126 MHz, Chloroform-*d*)

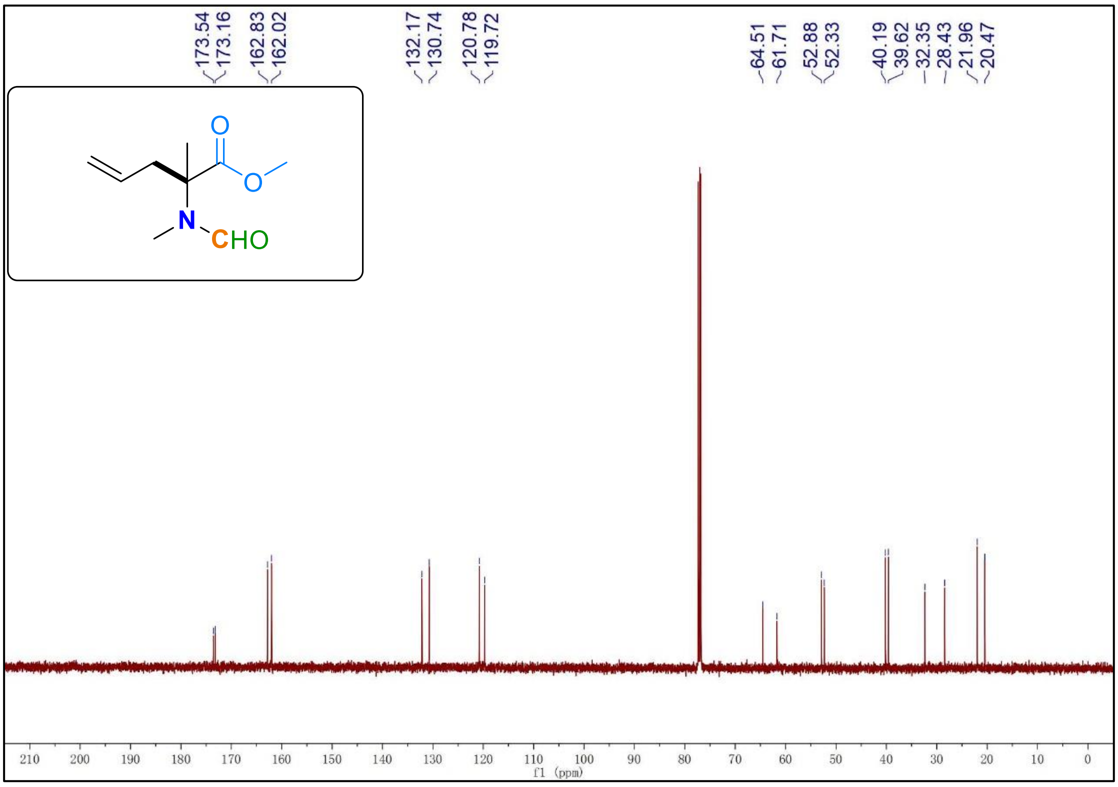

ethyl 2-methyl-2-(*N*-methylformamido)pent-4-enoate (6b)

^{1}H NMR (500 MHz, Chloroform-*d*)

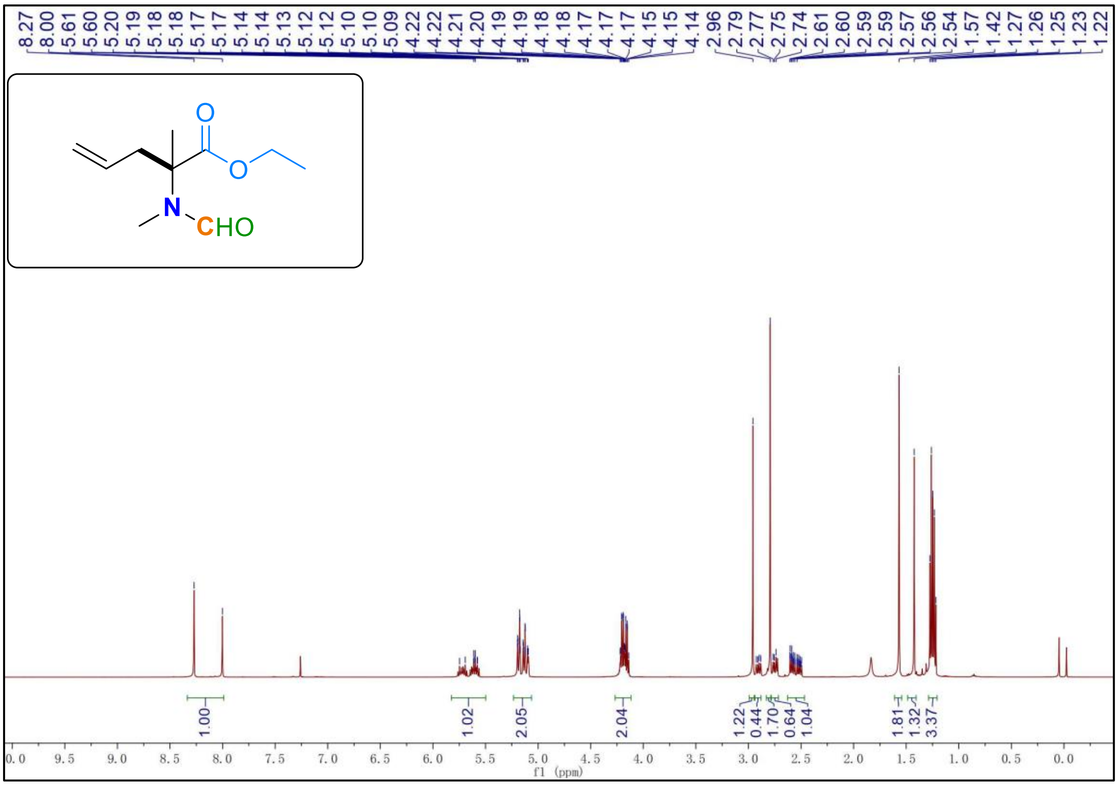

^{13}C NMR (126 MHz, Chloroform-*d*)

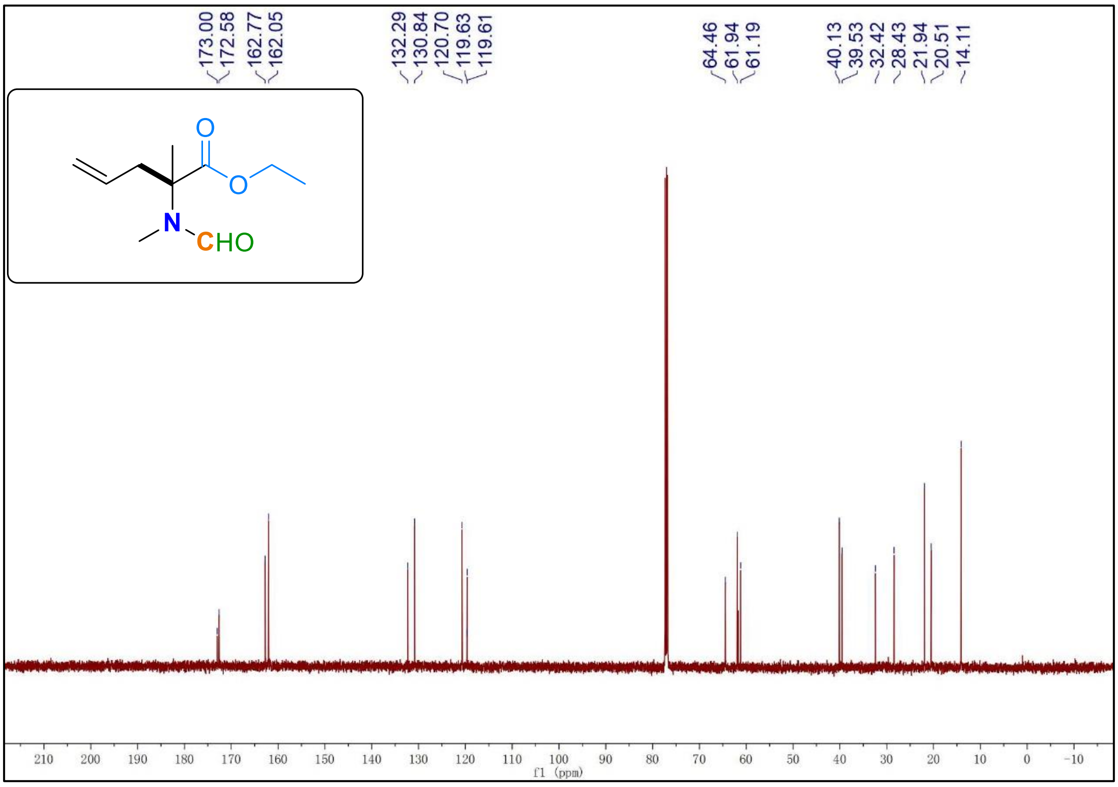

4-iodobenzyl 2-methyl-2-(*N*-methylformamido)pent-4-enoate (6c)

^{1}H NMR (500 MHz, Chloroform-*d*)

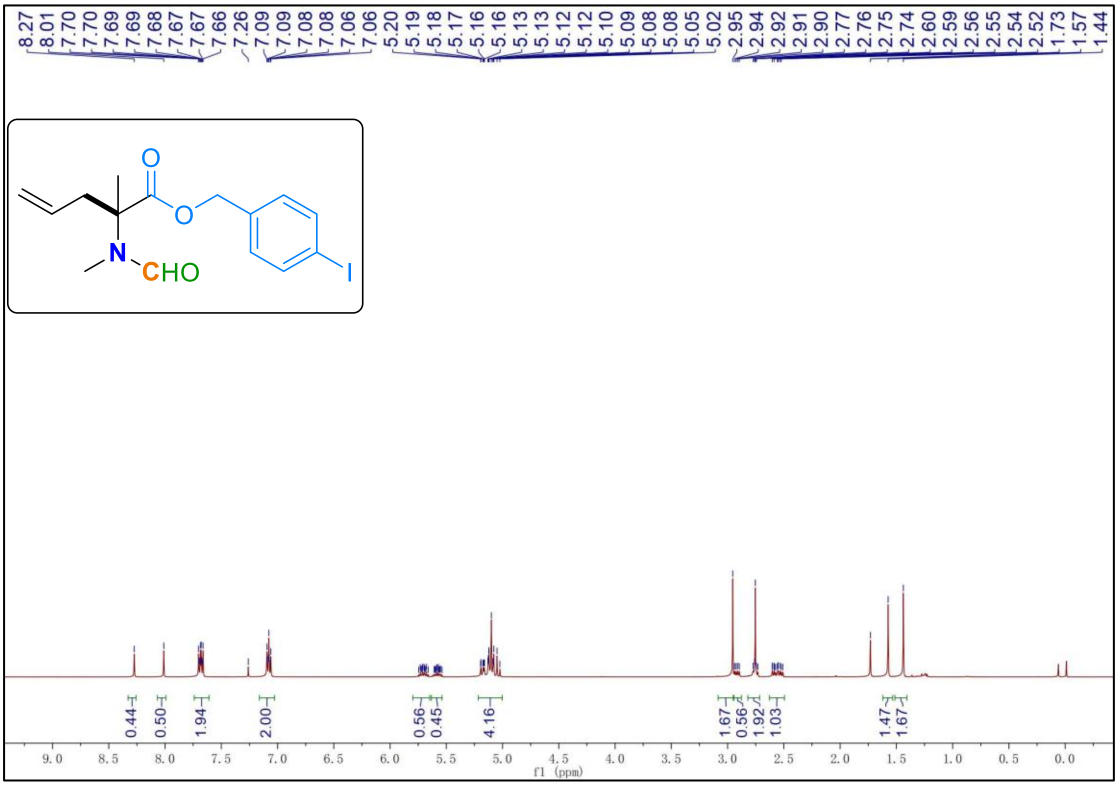

^{13}C NMR (126 MHz, Chloroform-*d*)

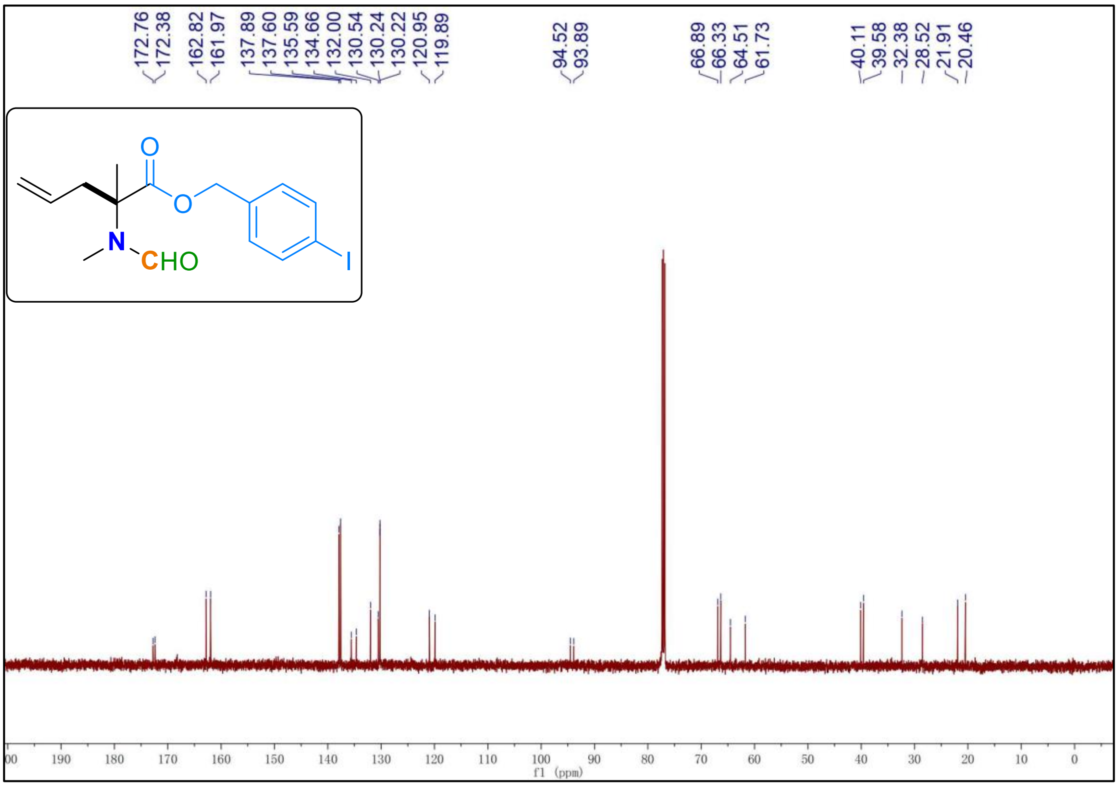

ethyl 2-(*N*-methylformamido)-2-phenylpent-4-enoate (6d)

^{1}H NMR (500 MHz, Chloroform-*d*)

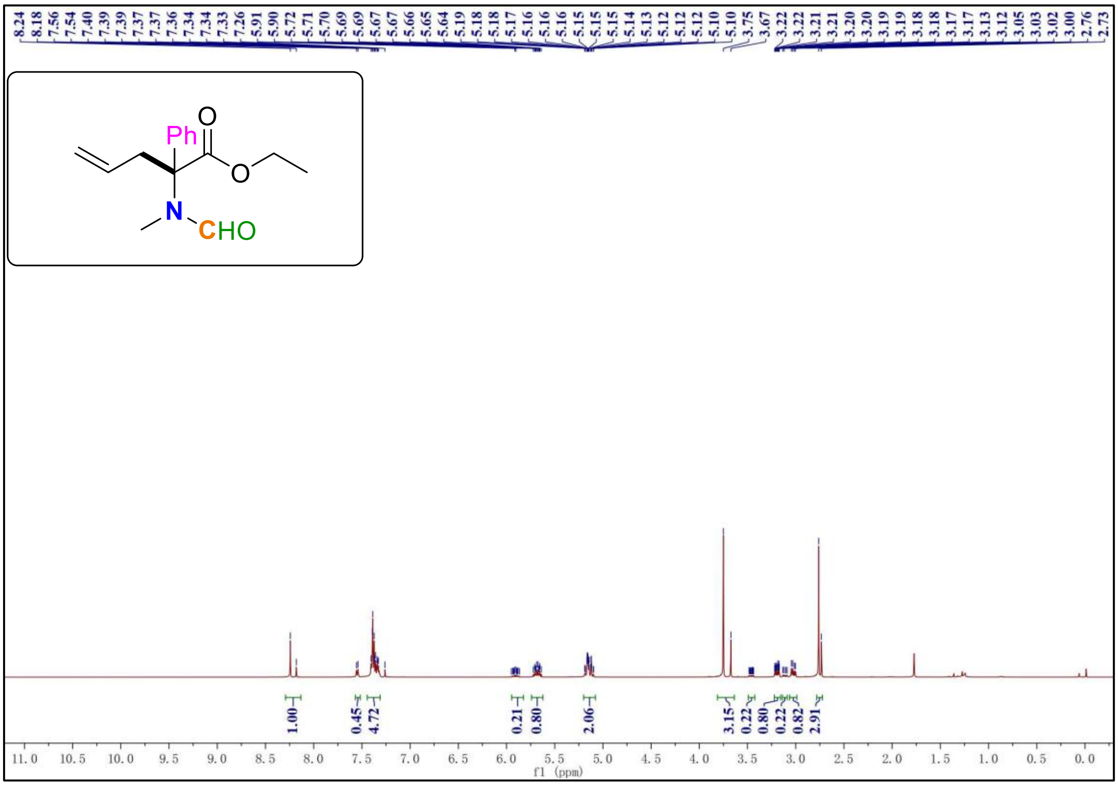

^{13}C NMR (126 MHz, Chloroform-*d*)

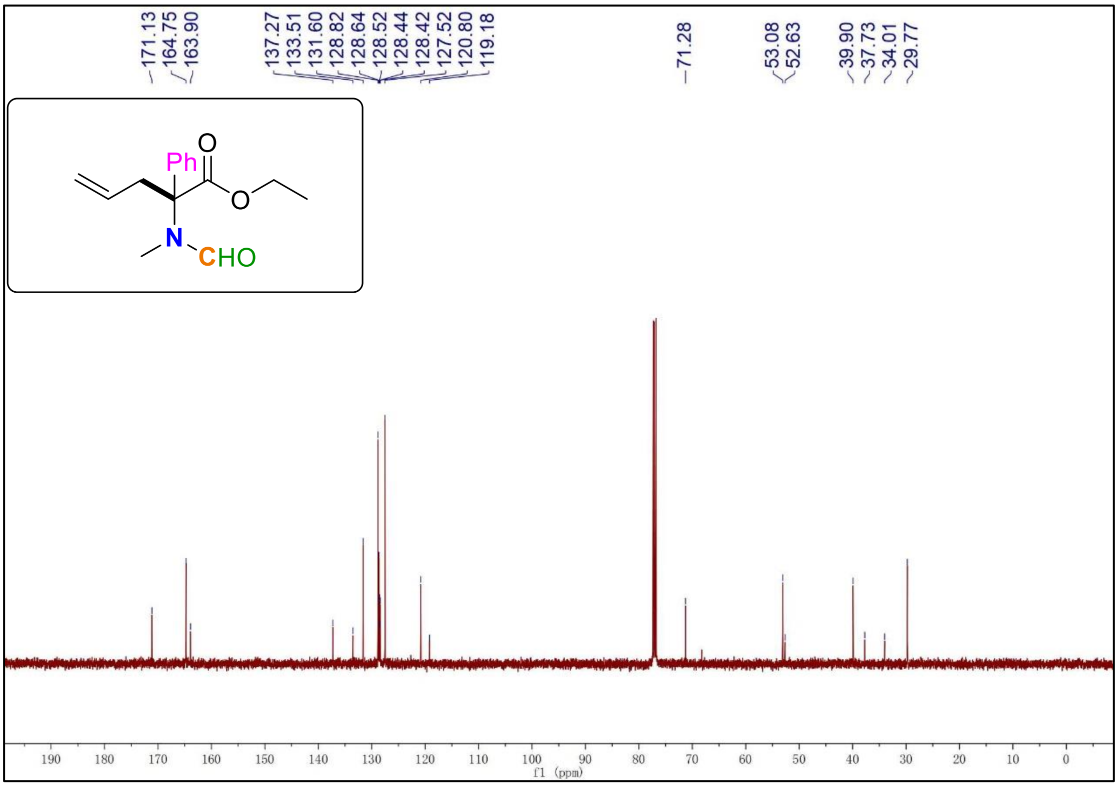

N-(3-allyl-2-oxotetrahydrofuran-3-yl)-*N*-methylformamide (6e)

^{1}H NMR (500 MHz, Chloroform-*d*)

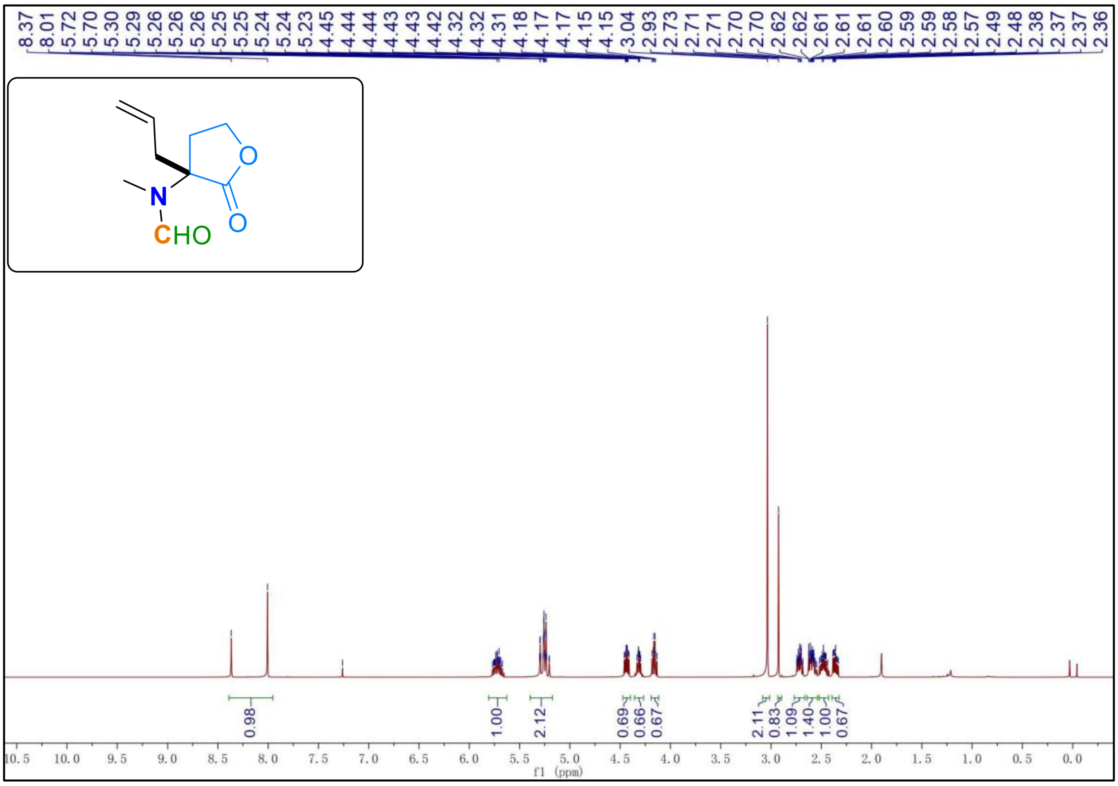

^{13}C NMR (126 MHz, Chloroform-*d*)

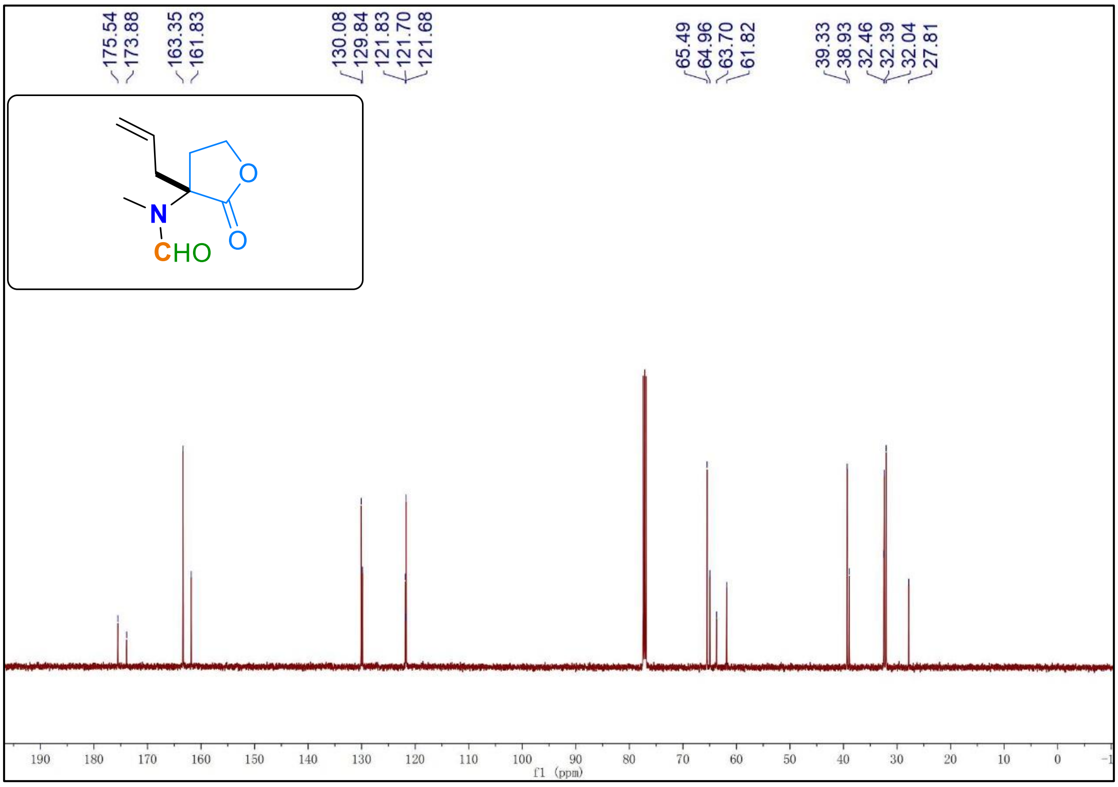

methyl 2-allyl-1-formylpyrrolidine-2-carboxylate (6f)

^{1}H NMR (500 MHz, Chloroform-*d*)

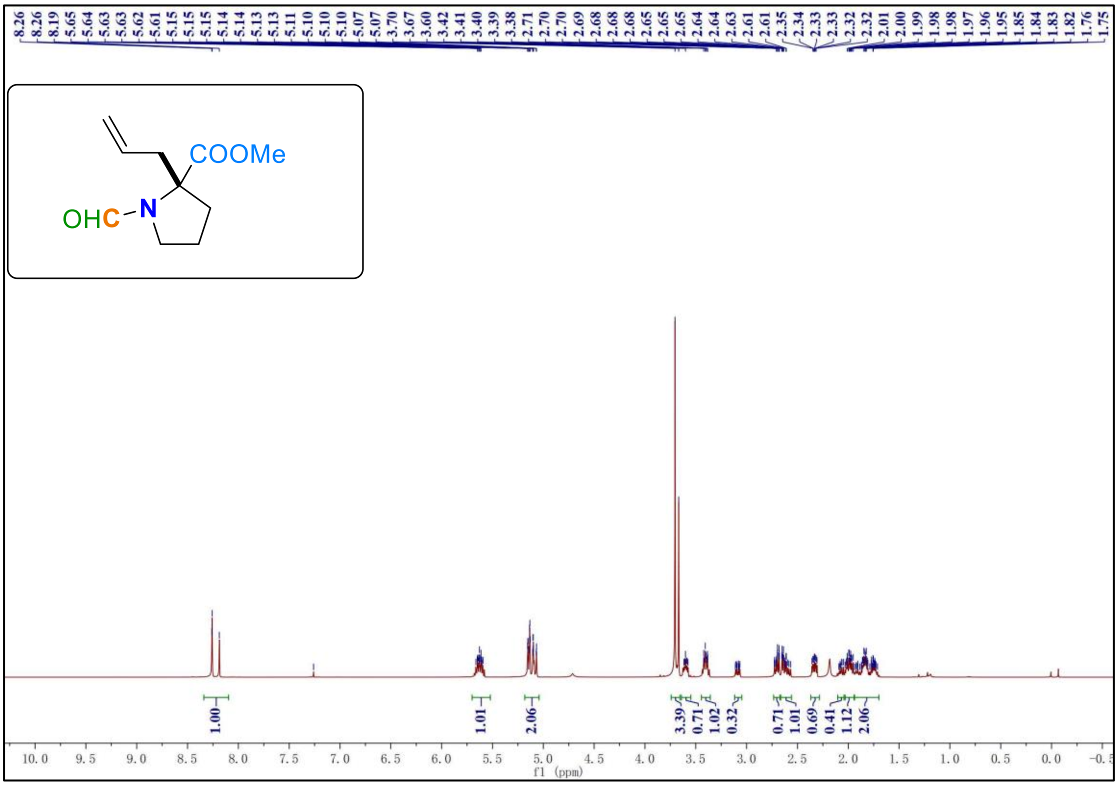

^{13}C NMR (126 MHz, Chloroform-*d*)

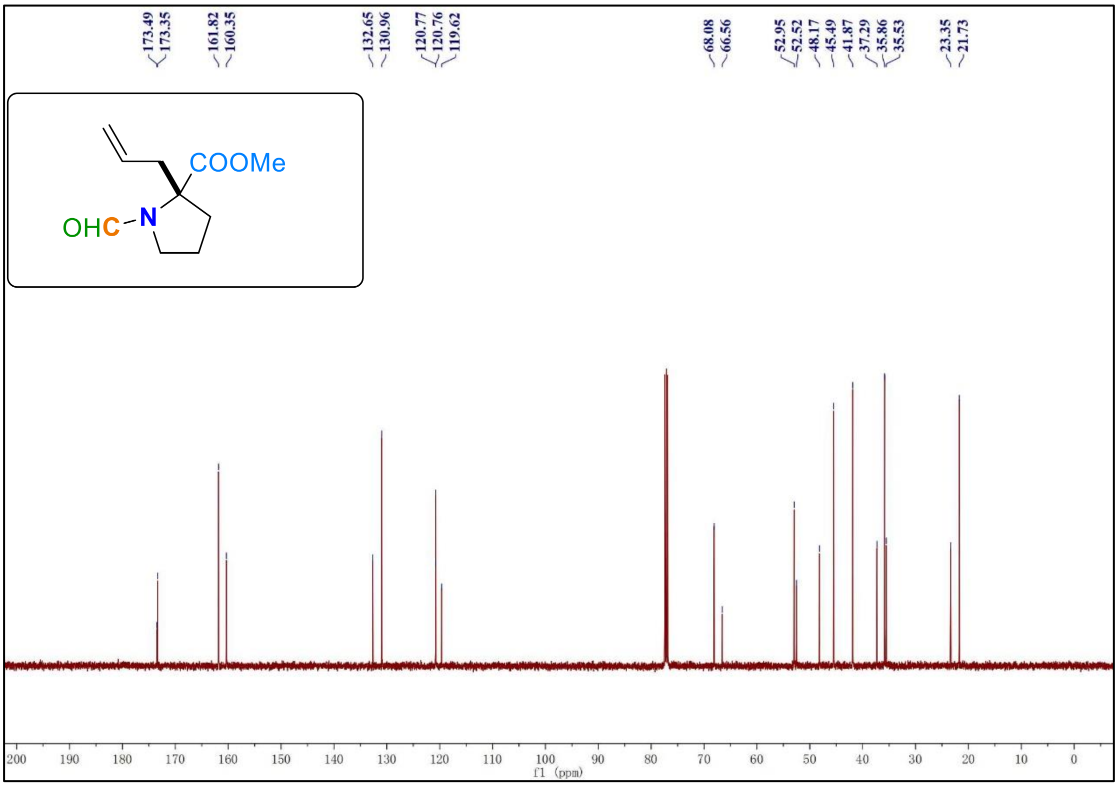

tert-butyl 2-allyl-1-formylpyrrolidine-2-carboxylate (6g)

^{1}H NMR (500 MHz, Chloroform-*d*)

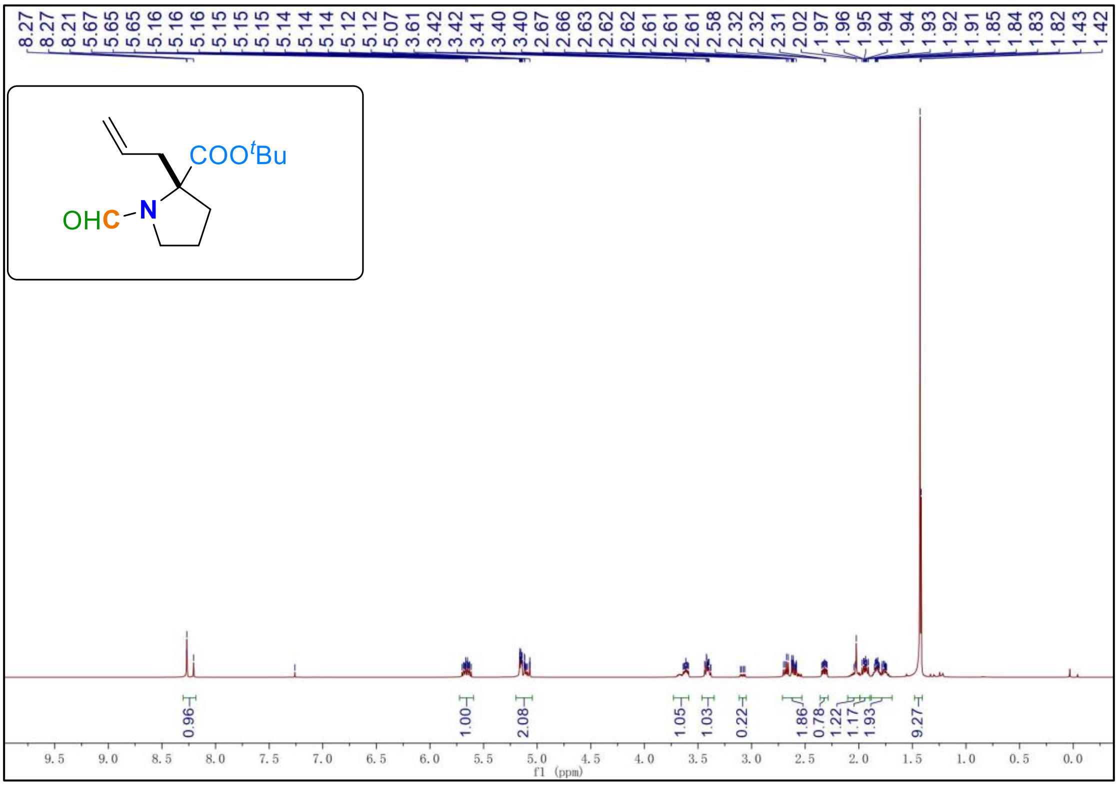

^{13}C NMR (126 MHz, Chloroform-*d*)

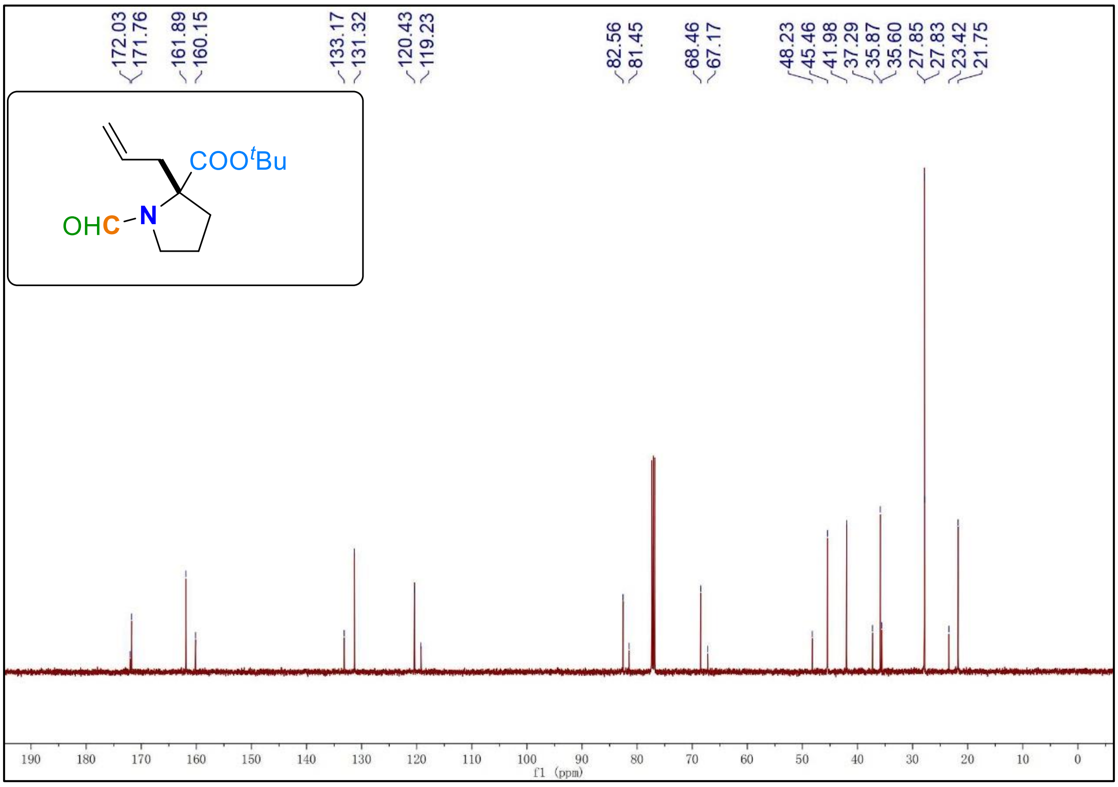

methyl 1-formyl-2-(2-methylallyl)pyrrolidine-2-carboxylate (6h)

^{1}H NMR (500 MHz, Chloroform-*d*)

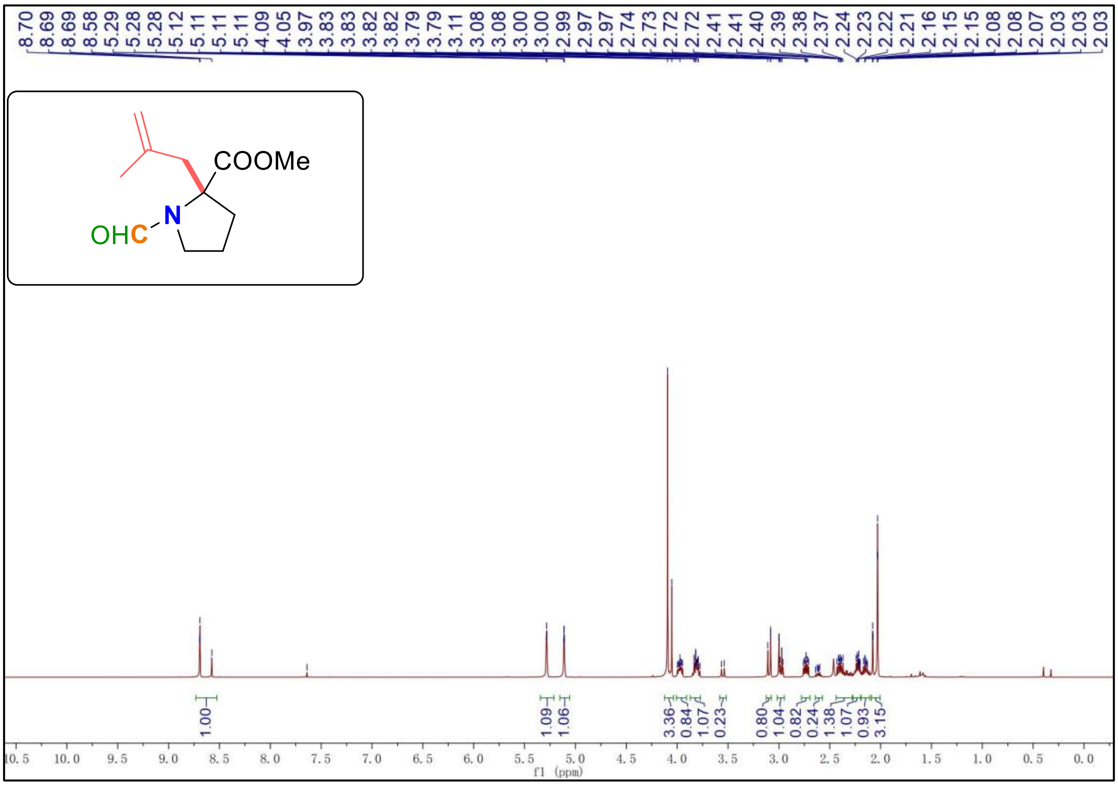

^{13}C NMR (126 MHz, Chloroform-*d*)

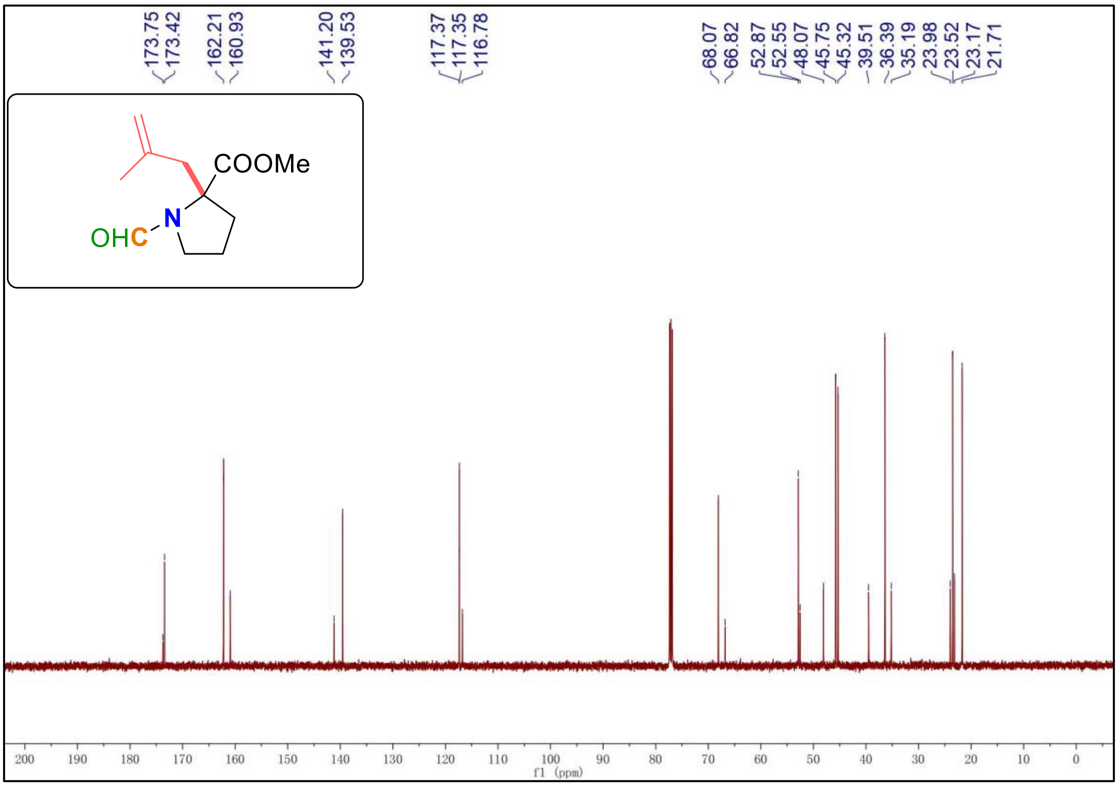

methyl 1-formyl-2-(2-methylbut-3-en-2-yl)pyrrolidine-2-carboxylate (6i)

^{1}H NMR (500 MHz, Chloroform-*d*)

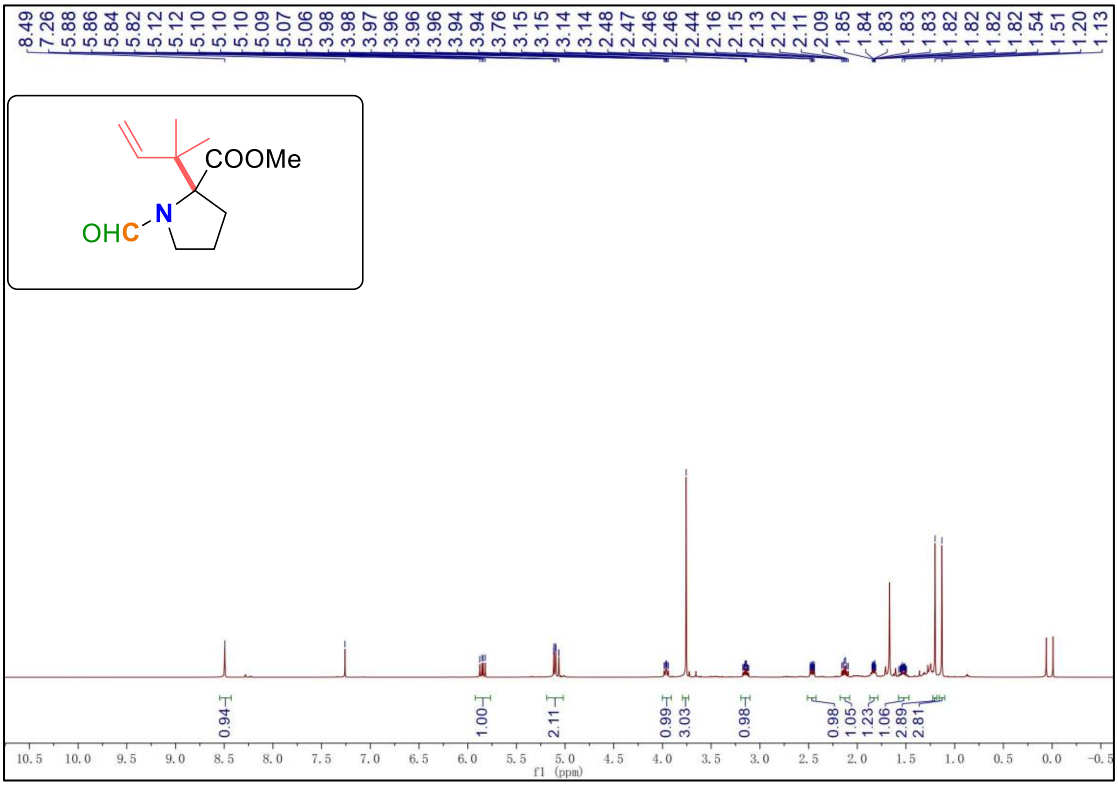

^{13}C NMR (126 MHz, Chloroform-*d*)

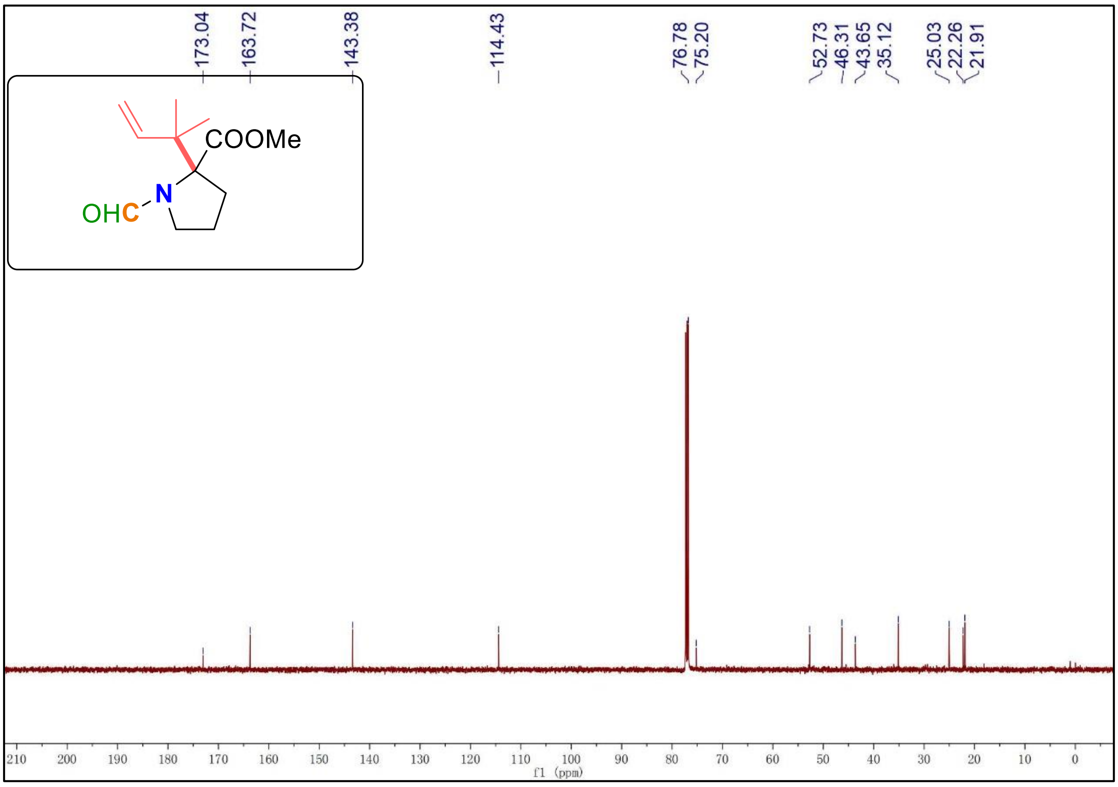

methyl 2-benzyl-1-formylpyrrolidine-2-carboxylate (7a)

^{1}H NMR (500 MHz, Chloroform-*d*)

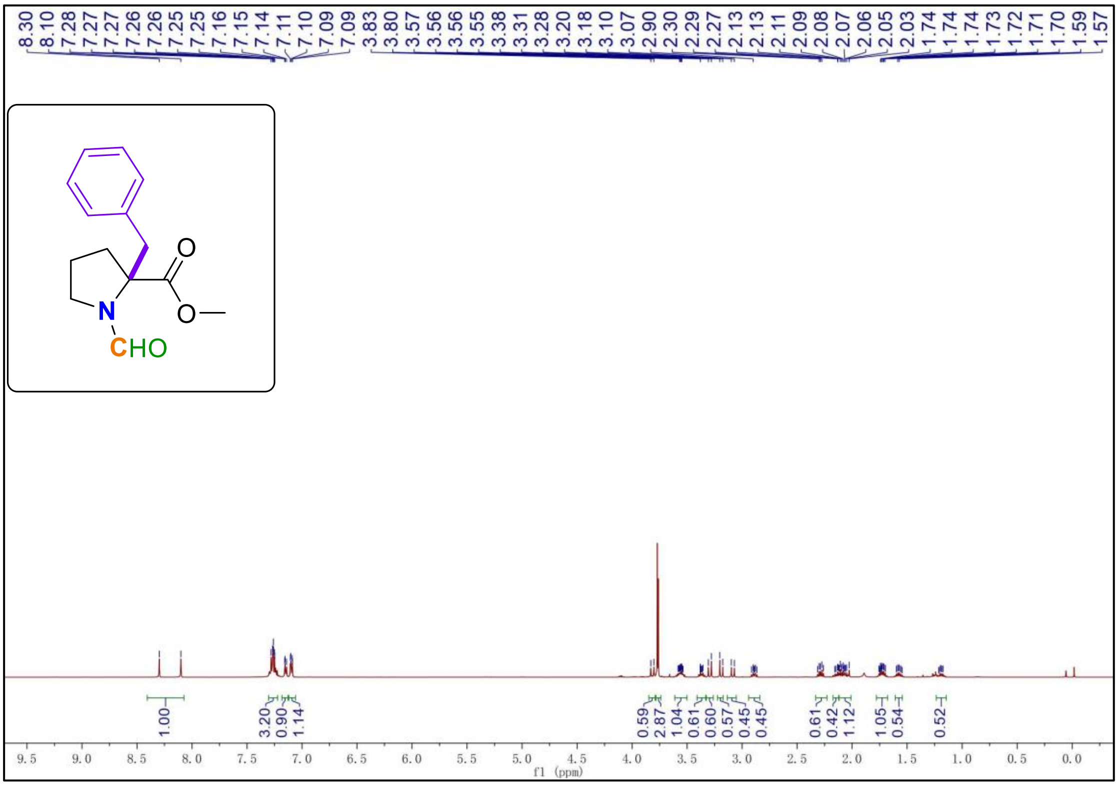

^{13}C NMR (126 MHz, Chloroform-*d*)

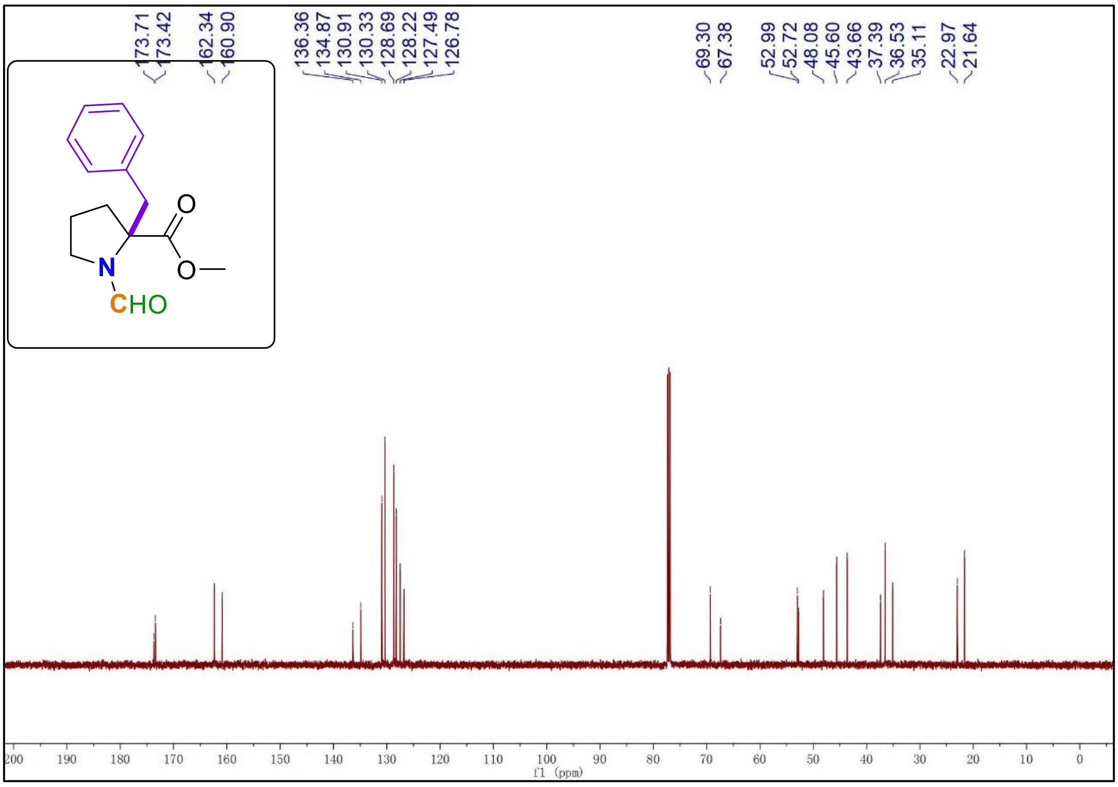

methyl 2-([1,1'-biphenyl]-4-ylmethyl)-1-formylpyrrolidine-2-carboxylate (7b)

^{1}H NMR (500 MHz, Chloroform-*d*)

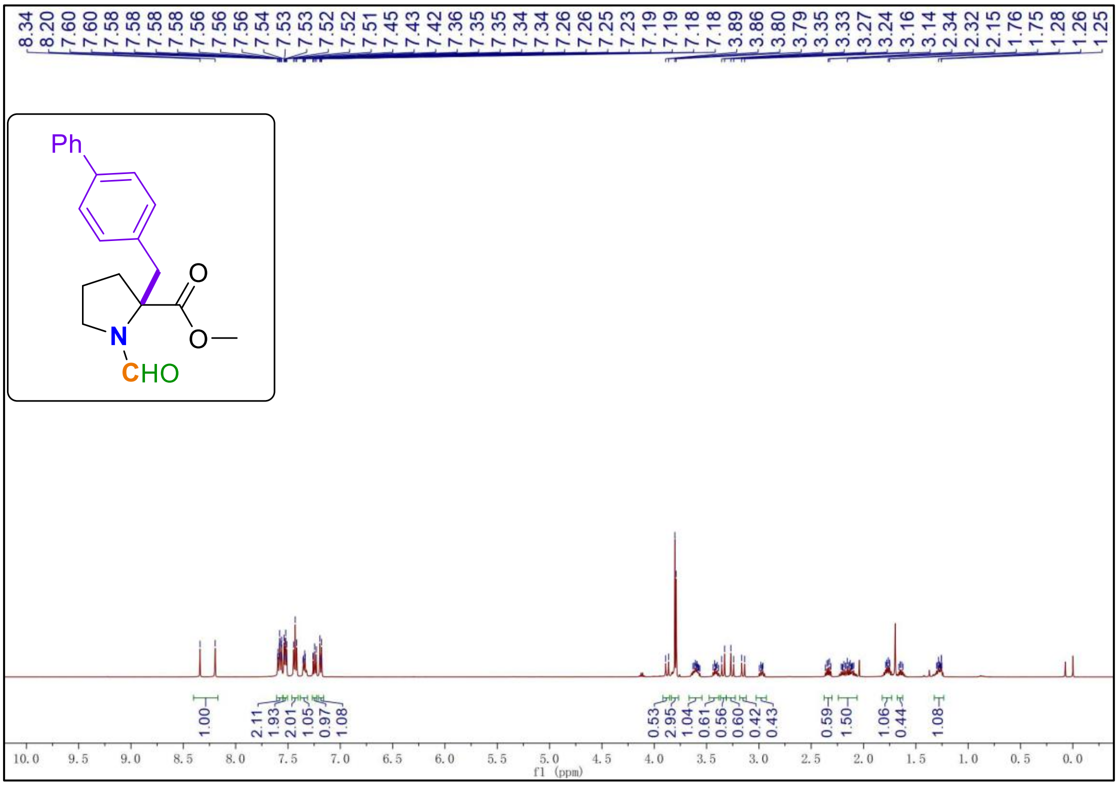

^{13}C NMR (126 MHz, Chloroform-*d*)

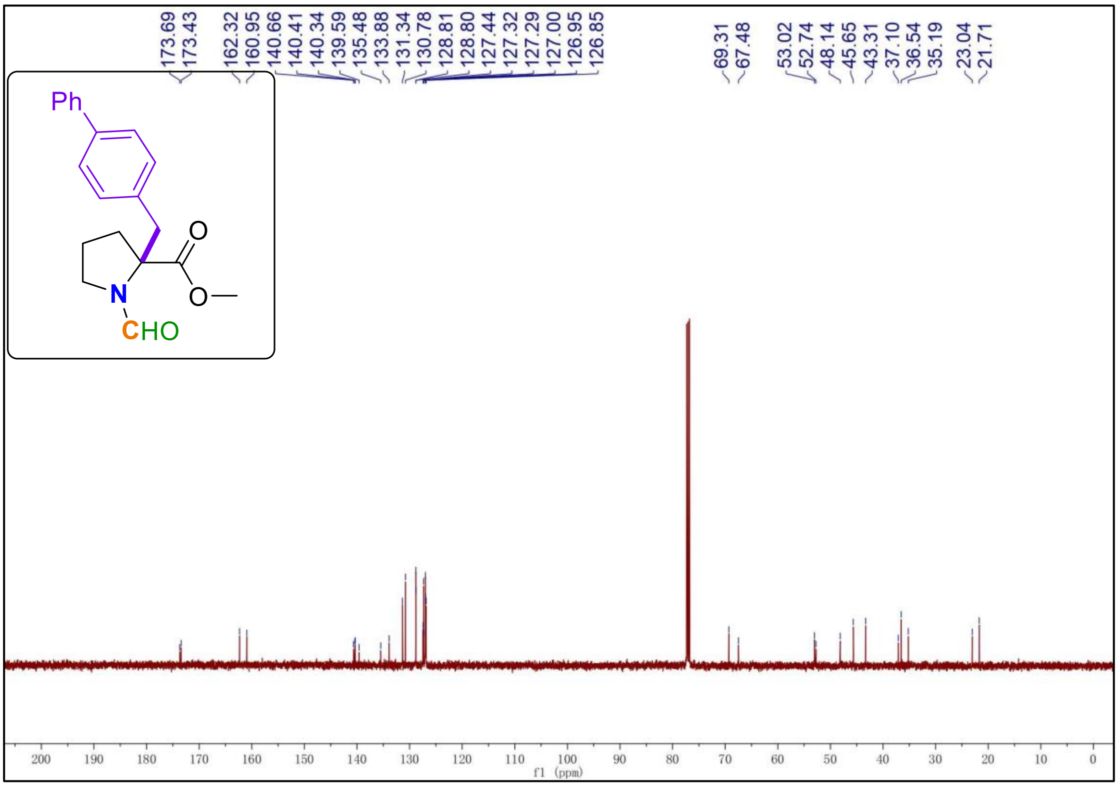

methyl 1-formyl-2-(4-iodobenzyl)pyrrolidine-2-carboxylate (7c)

^{1}H NMR (500 MHz, Chloroform-*d*)

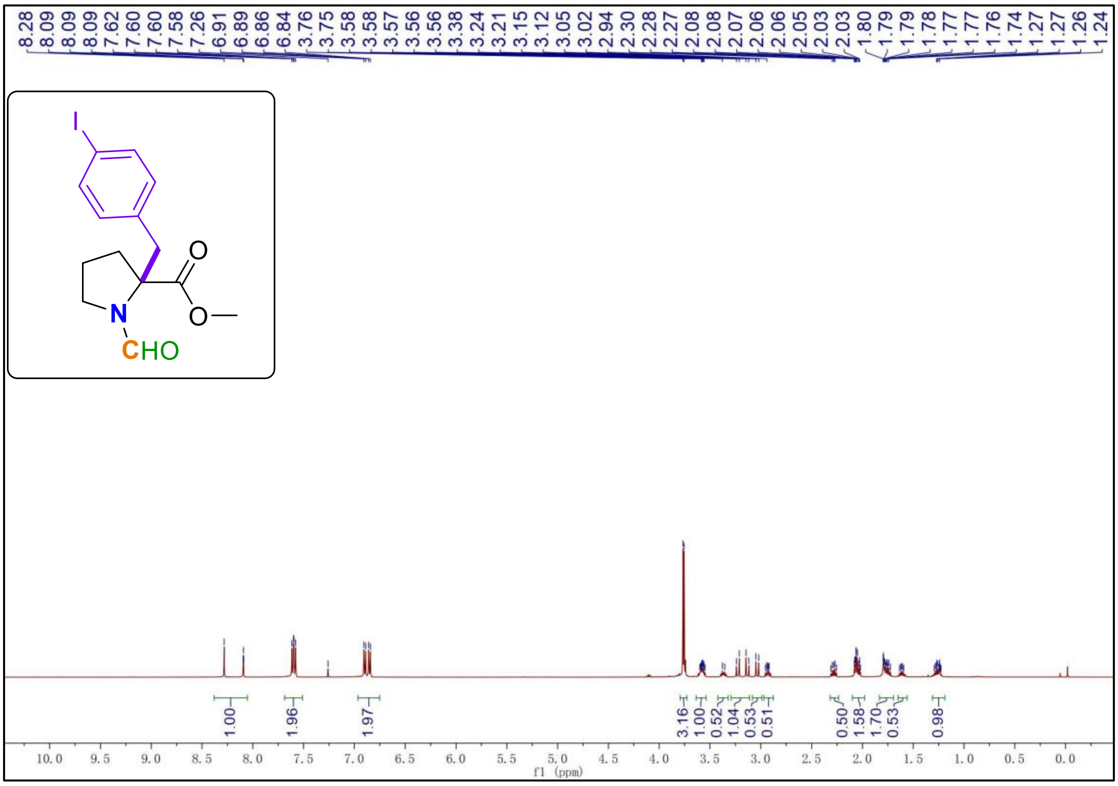

^{13}C NMR (126 MHz, Chloroform-*d*)

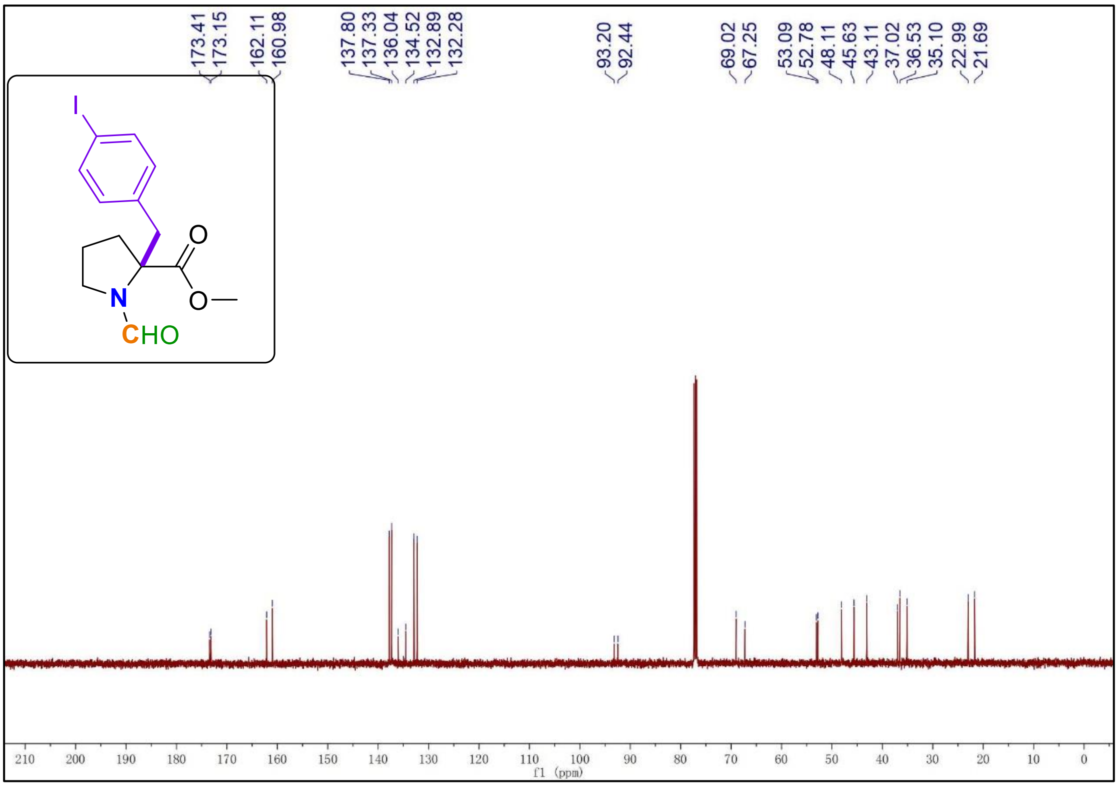

methyl 2-(4-bromobenzyl)-1-formylpyrrolidine-2-carboxylate (7d)

^{1}H NMR (500 MHz, Chloroform-*d*)

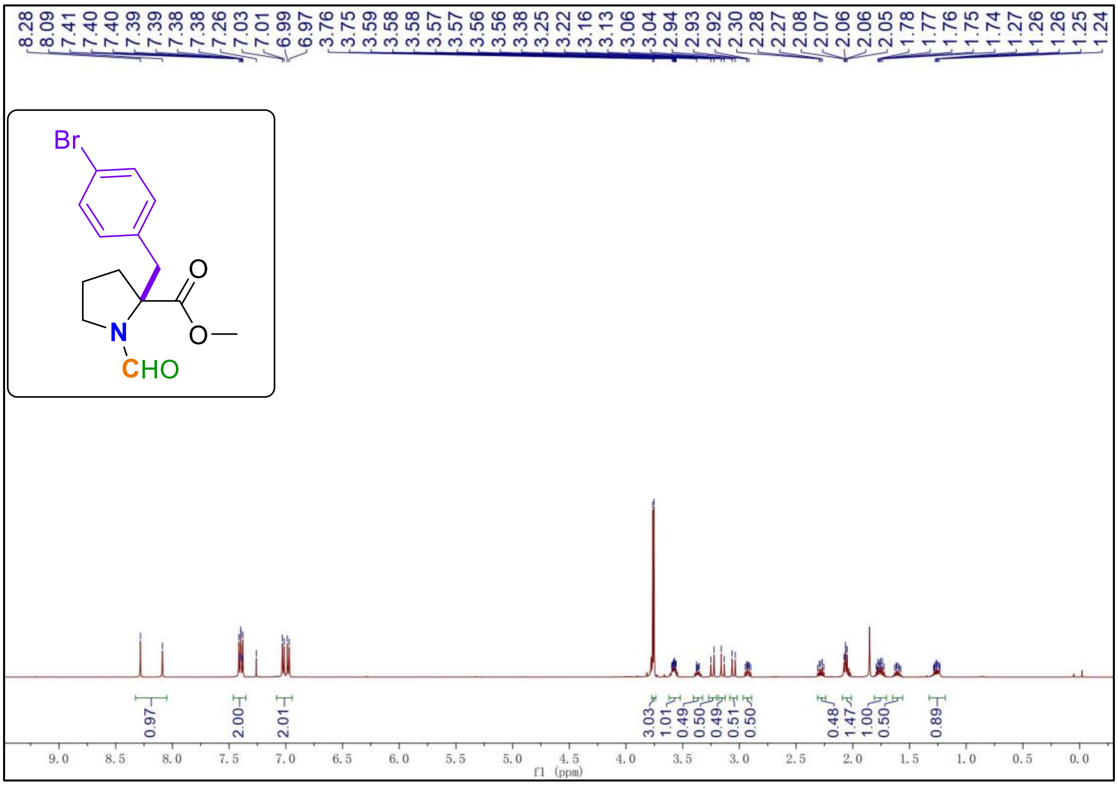

^{13}C NMR (126 MHz, Chloroform-*d*)

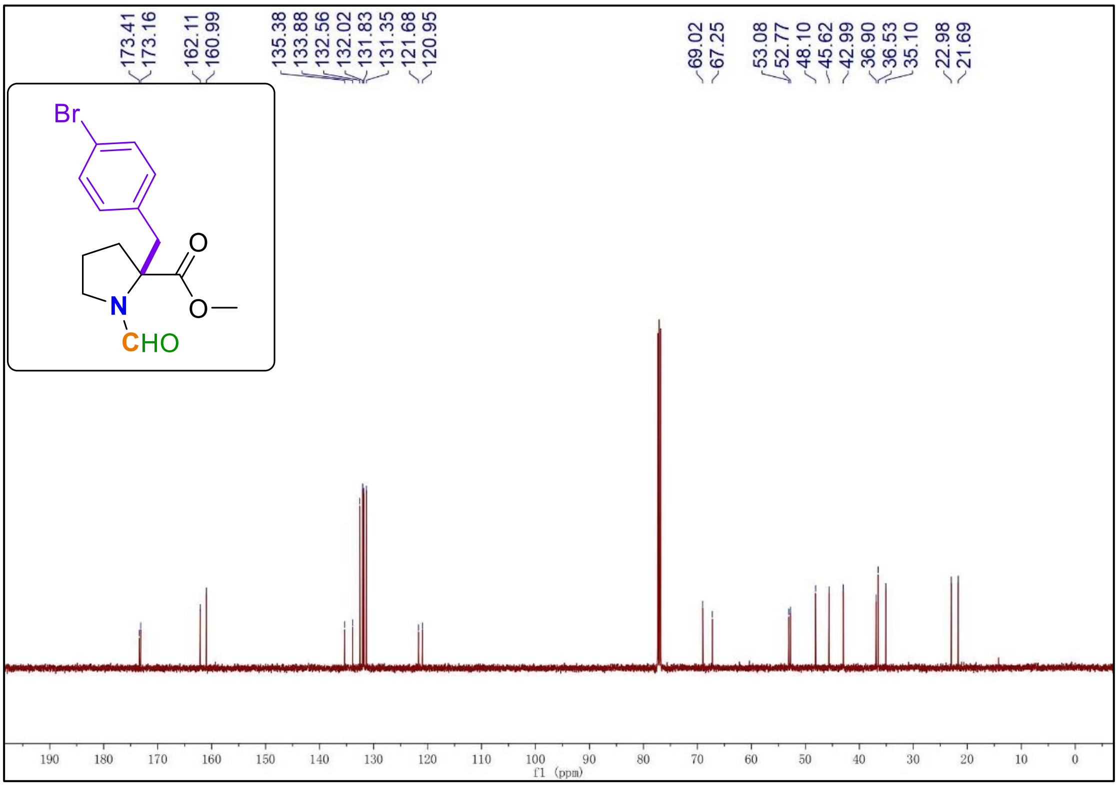

methyl 2-(4-bromo-3-fluorobenzyl)-1-formylpyrrolidine-2-carboxylate (7e)

^{1}H NMR (500 MHz, Chloroform-*d*)

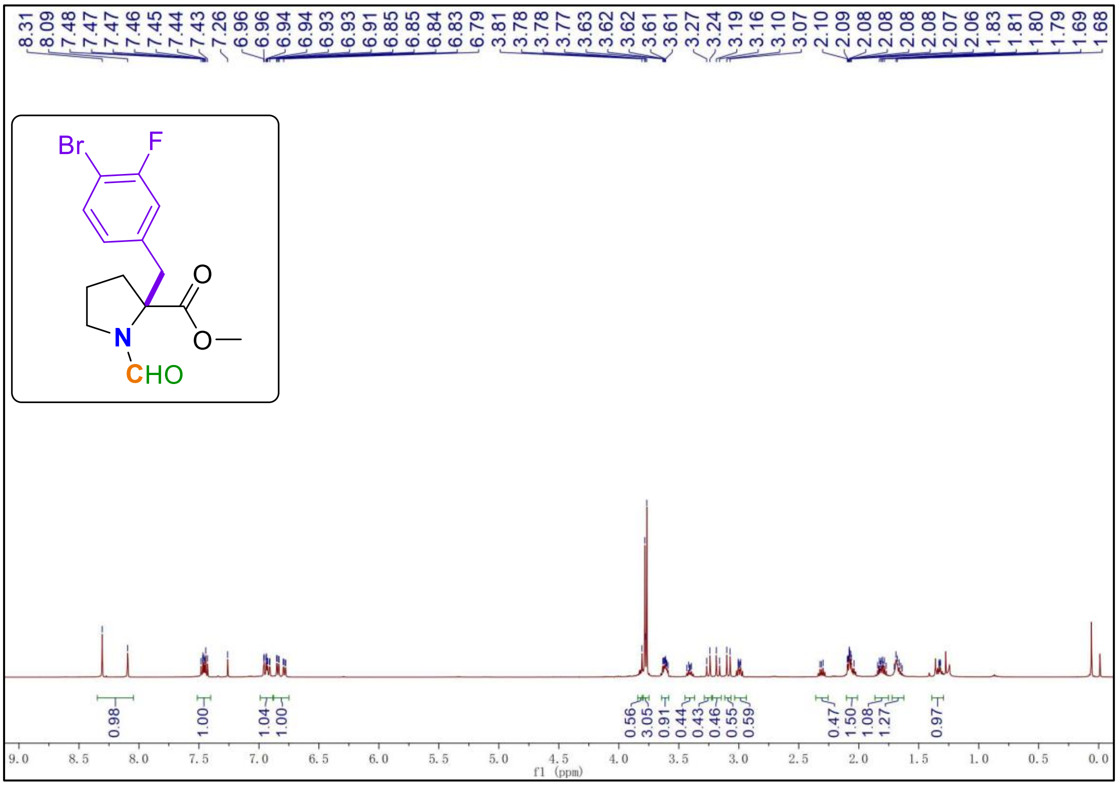

^{13}C NMR (126 MHz, Chloroform-*d*)

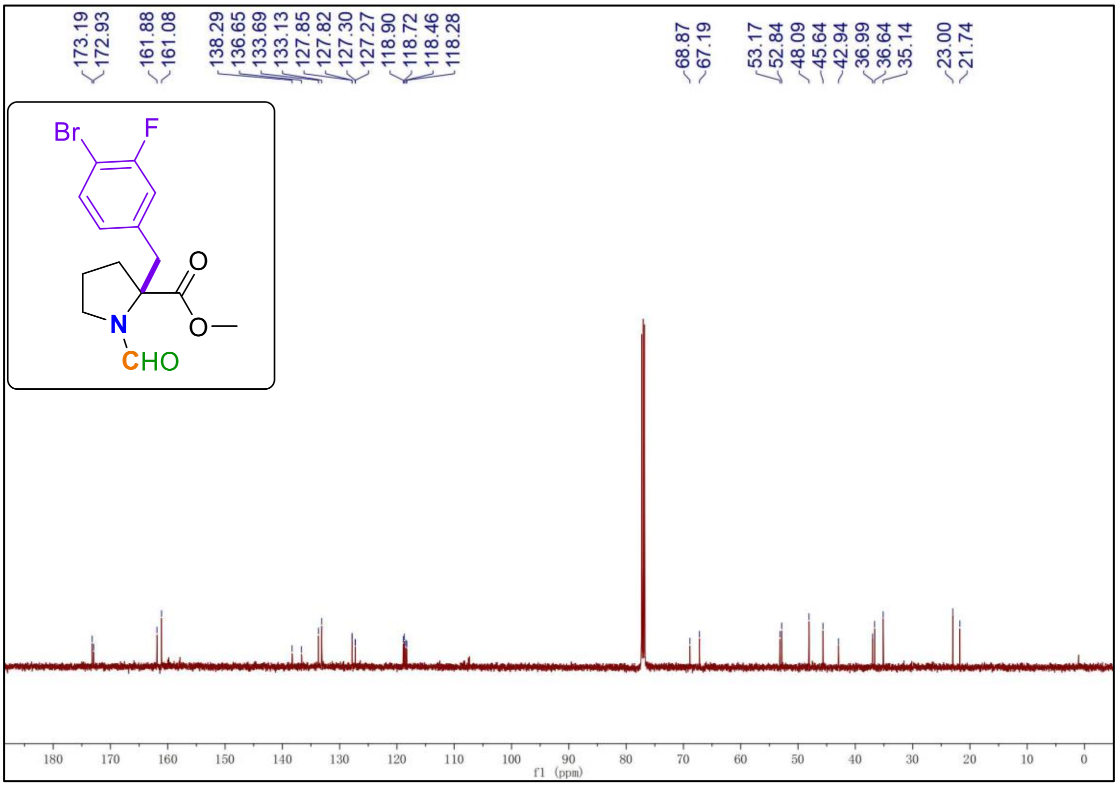

^{19}F NMR (471 MHz, Chloroform-*d*)

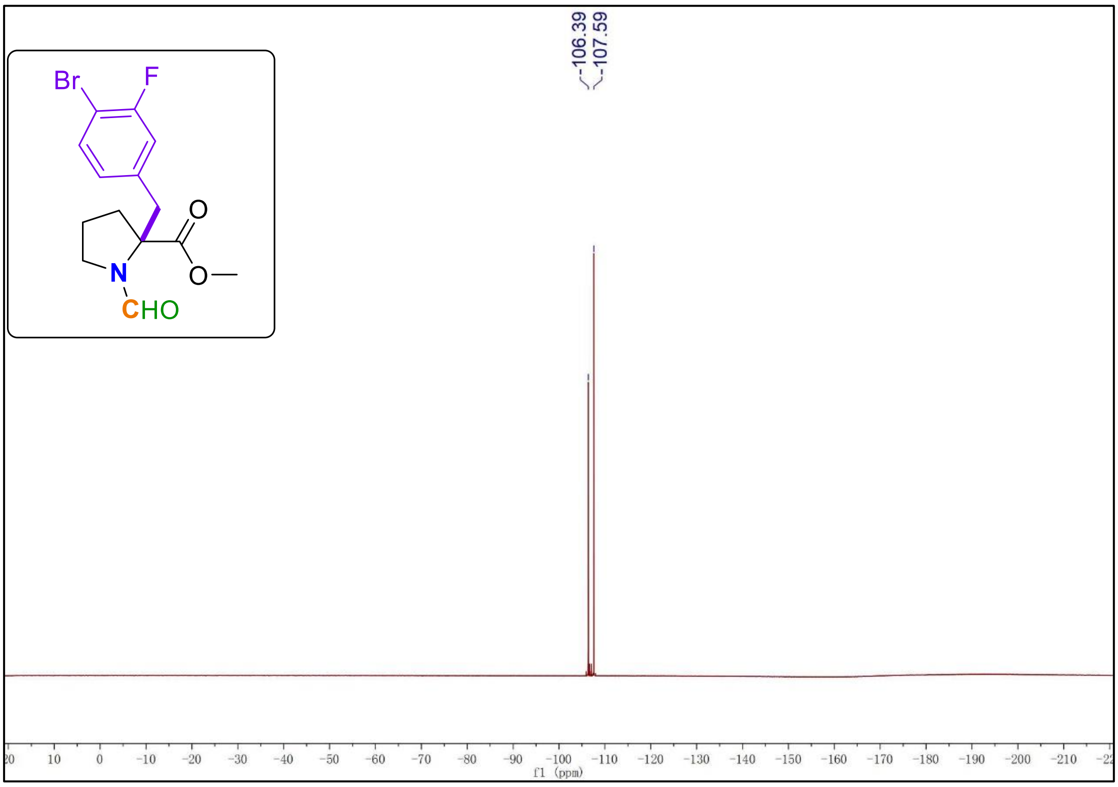

methyl 1-formyl-2-(propa-1,2-dien-1-yl)pyrrolidine-2-carboxylate（9a)

^{1}H NMR (500 MHz, Chloroform-*d*)

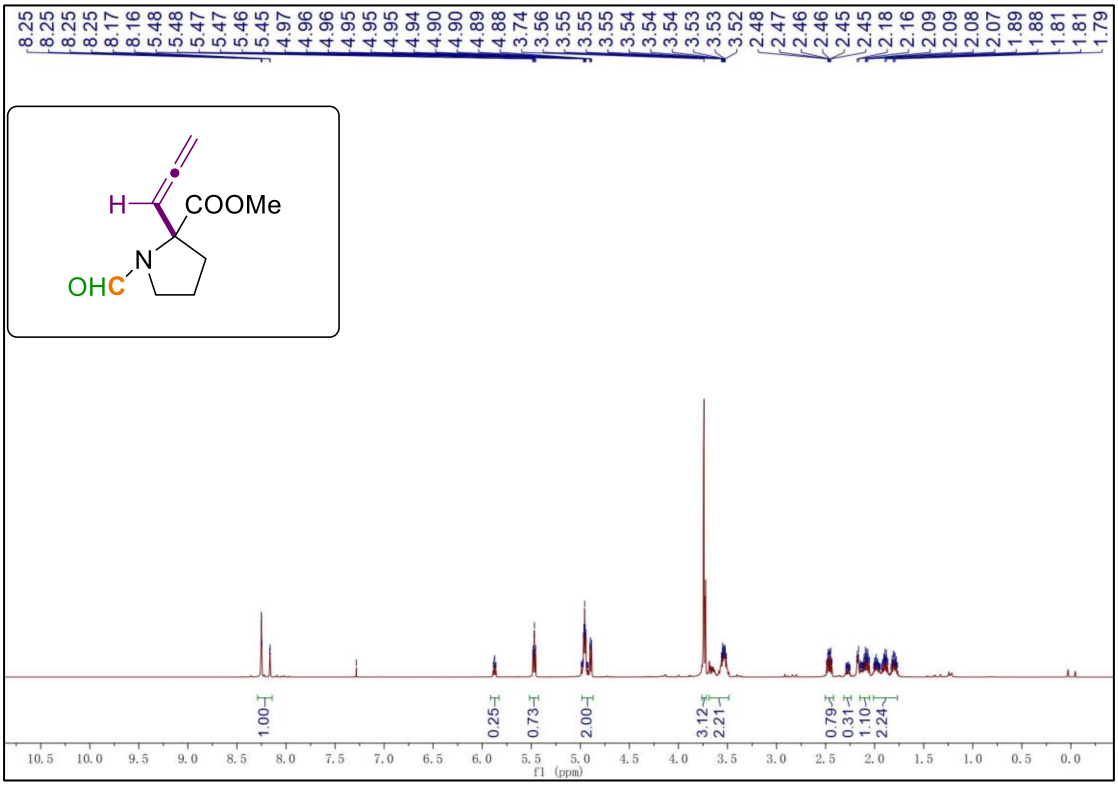

^{13}C NMR (126 MHz, Chloroform-*d*)

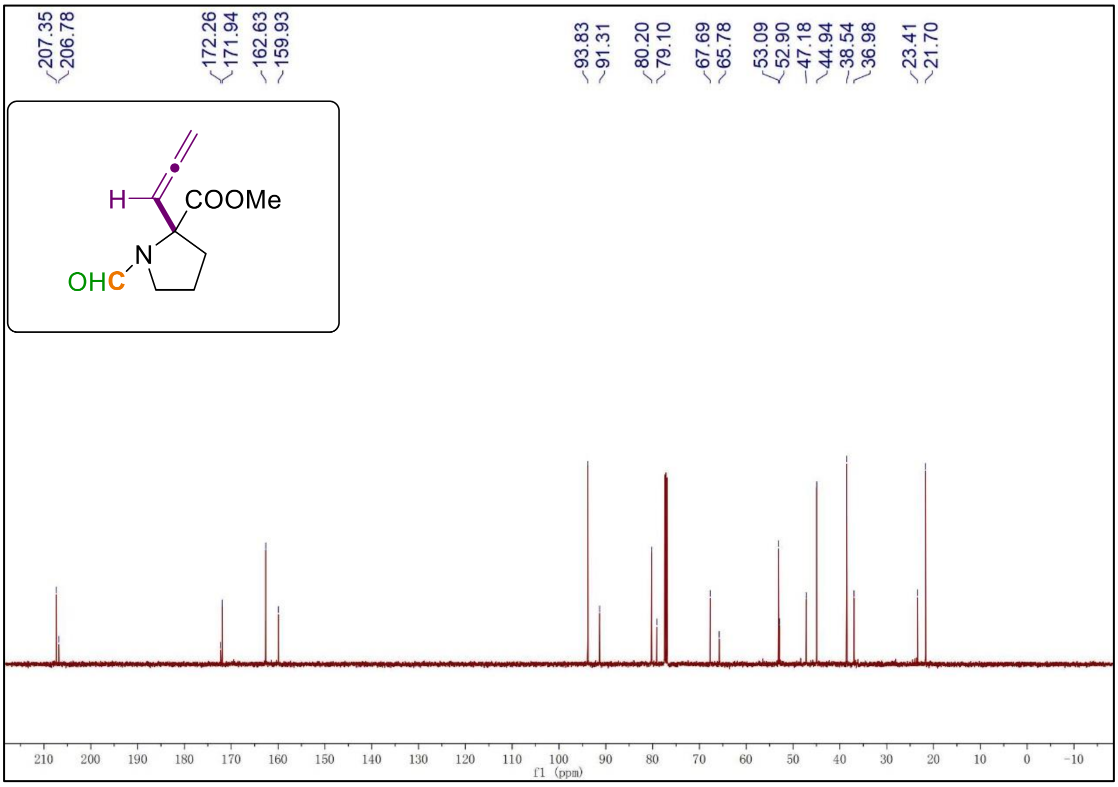

tert-butyl 1-formyl-2-(propa-1,2-dien-1-yl)pyrrolidine-2-carboxylate (9b)

^{1}H NMR (500 MHz, Chloroform-*d*)

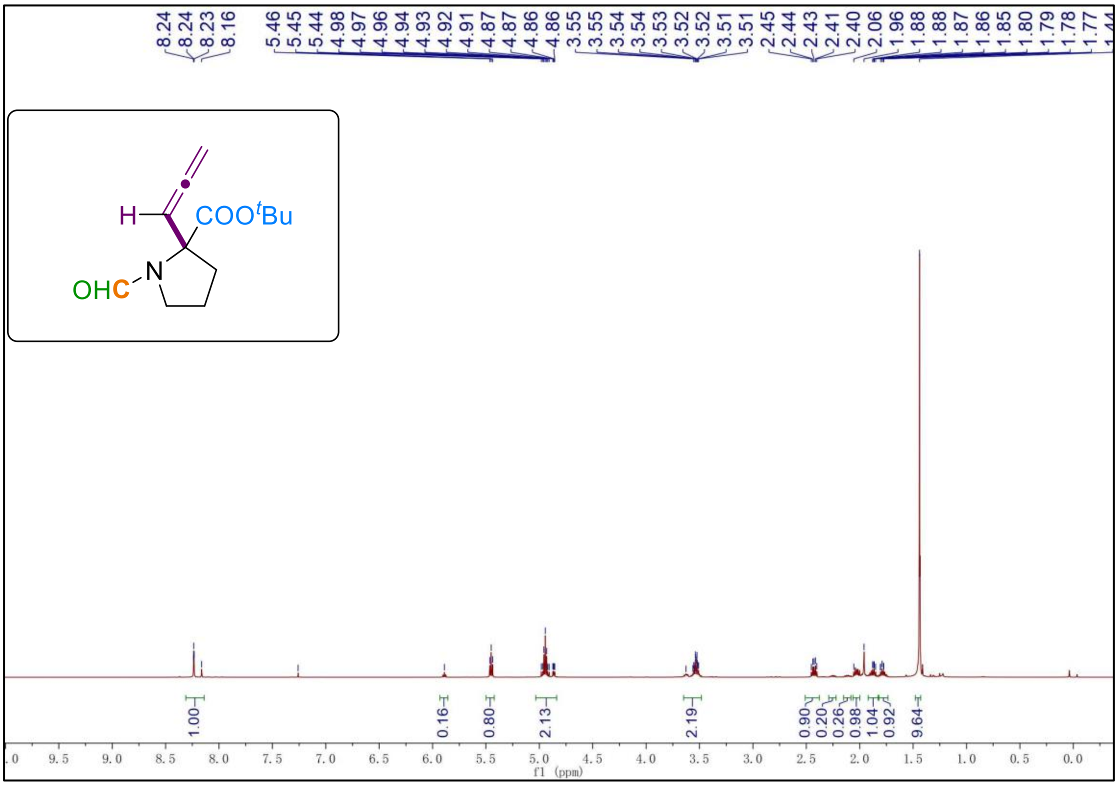

^{13}C NMR (126 MHz, Chloroform-*d*)

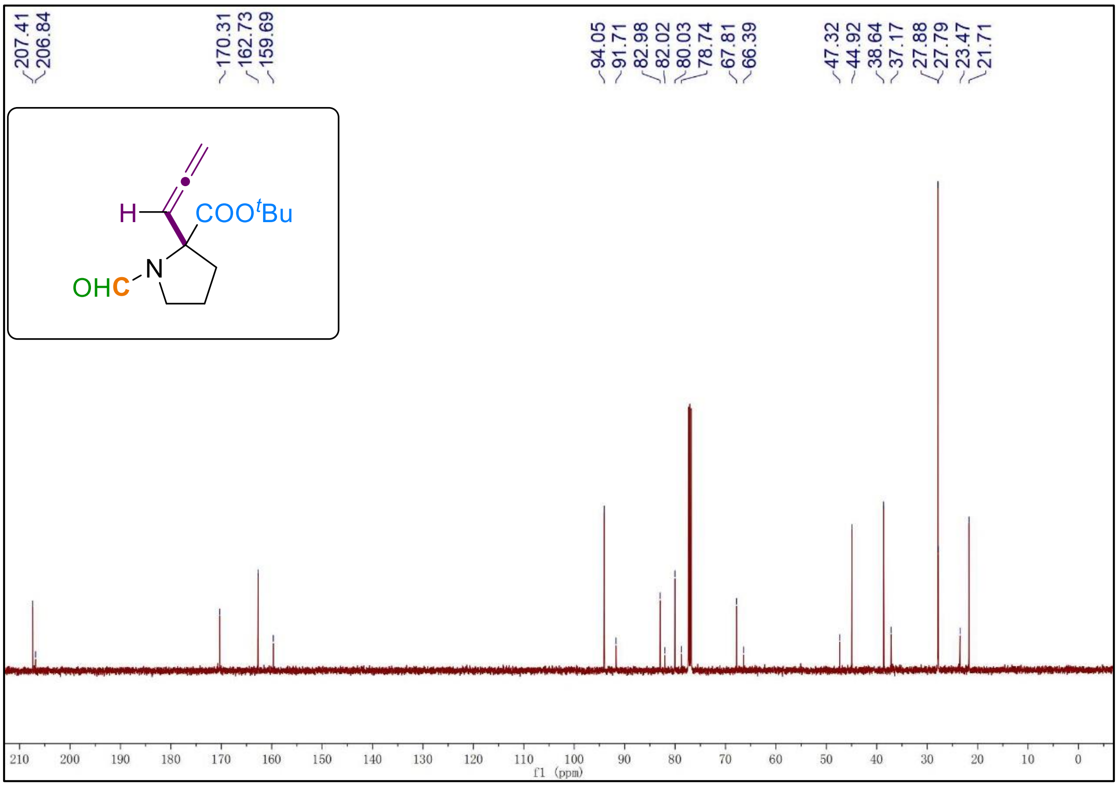

methyl 2-(buta-2,3-dien-2-yl)-1-formylpyrrolidine-2-carboxylate (9c)

^{1}H NMR (500 MHz, Chloroform-*d*)

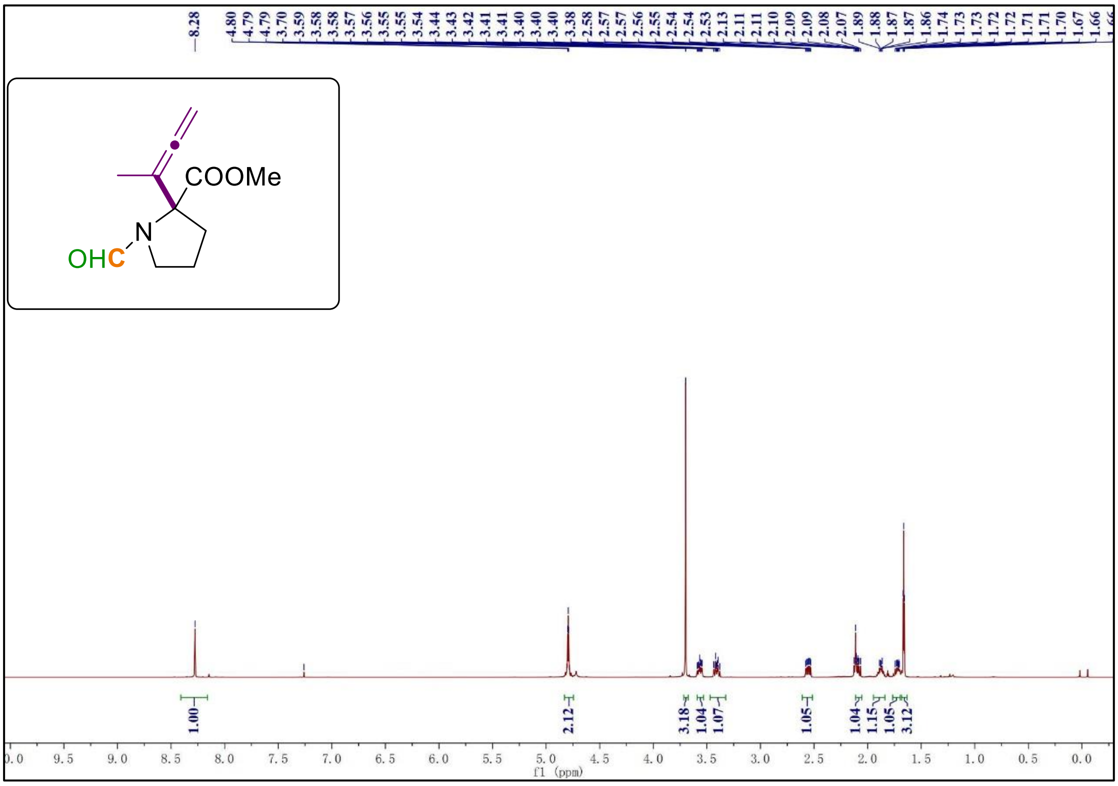

^{13}C NMR (126 MHz, Chloroform-*d*)

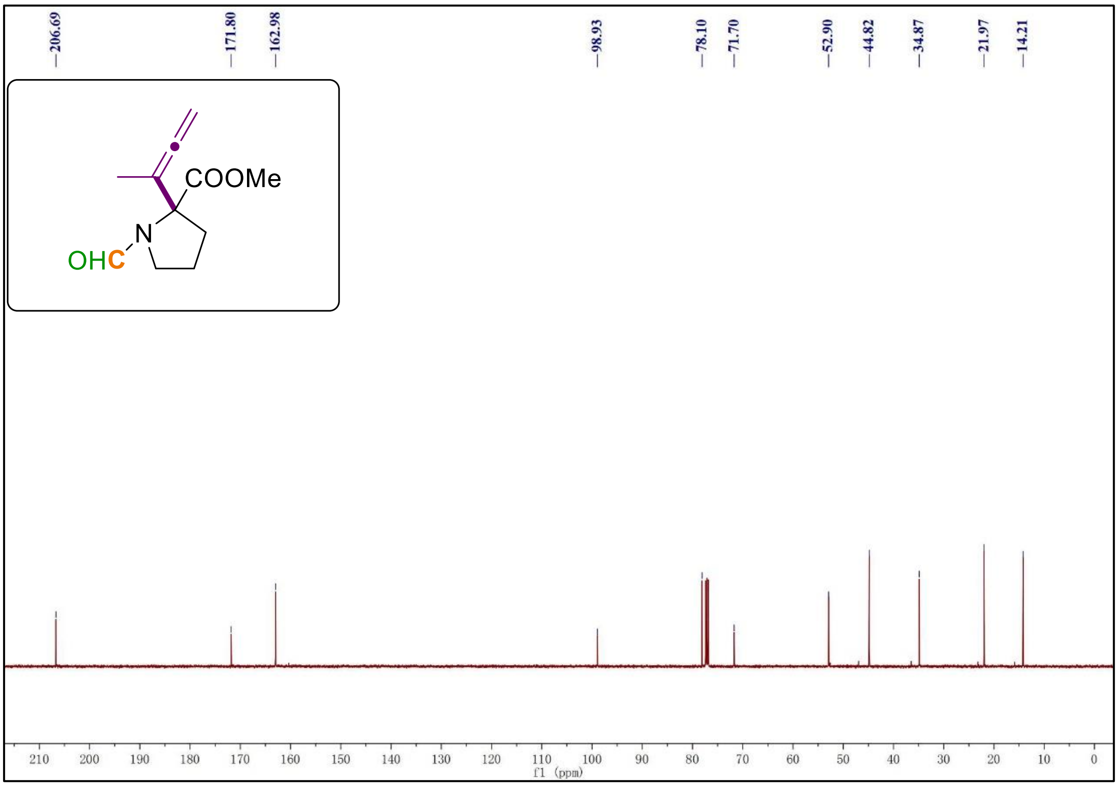

methyl 1-formyl-2-(penta-1,2-dien-3-yl)pyrrolidine-2-carboxylate (9d)

^{1}H NMR (500 MHz, Chloroform-*d*)

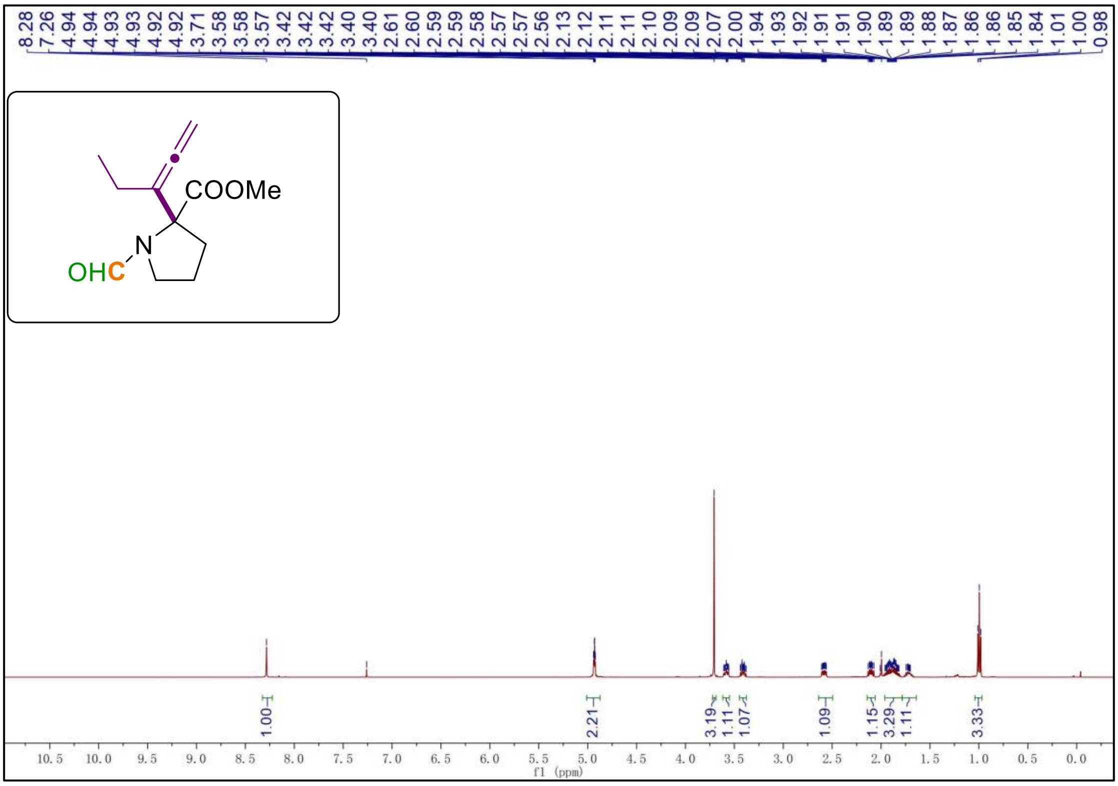

^{13}C NMR (126 MHz, Chloroform-*d*)

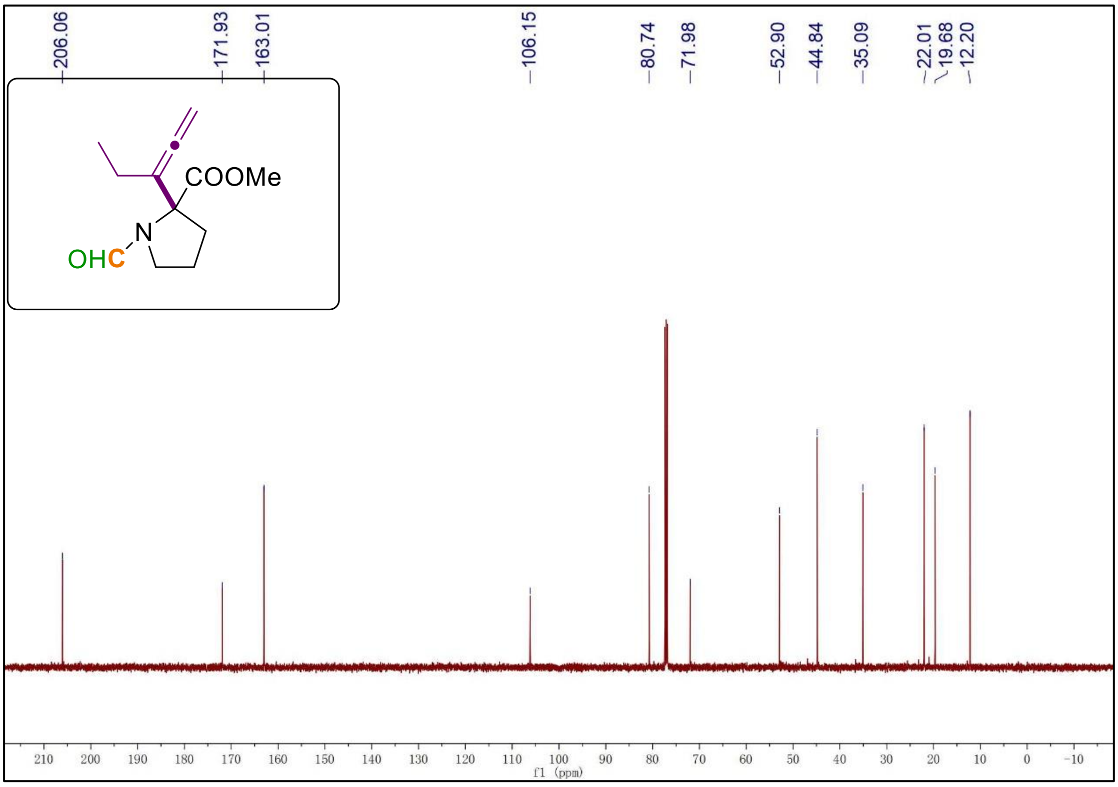

methyl 2-(1-cyclopropylpropa-1,2-dien-1-yl)-1-formylpyrrolidine-2-carboxylate (9e)

^{1}H NMR (500 MHz, Chloroform-*d*)

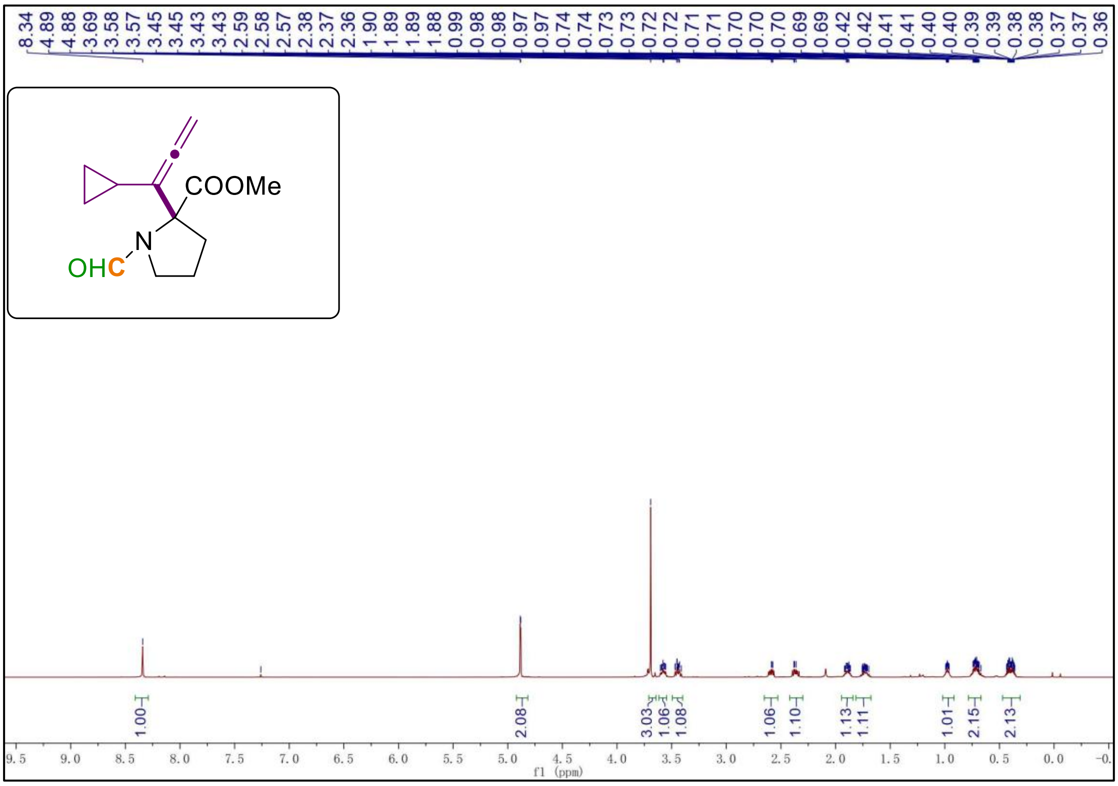

^{13}C NMR (126 MHz, Chloroform-*d*)

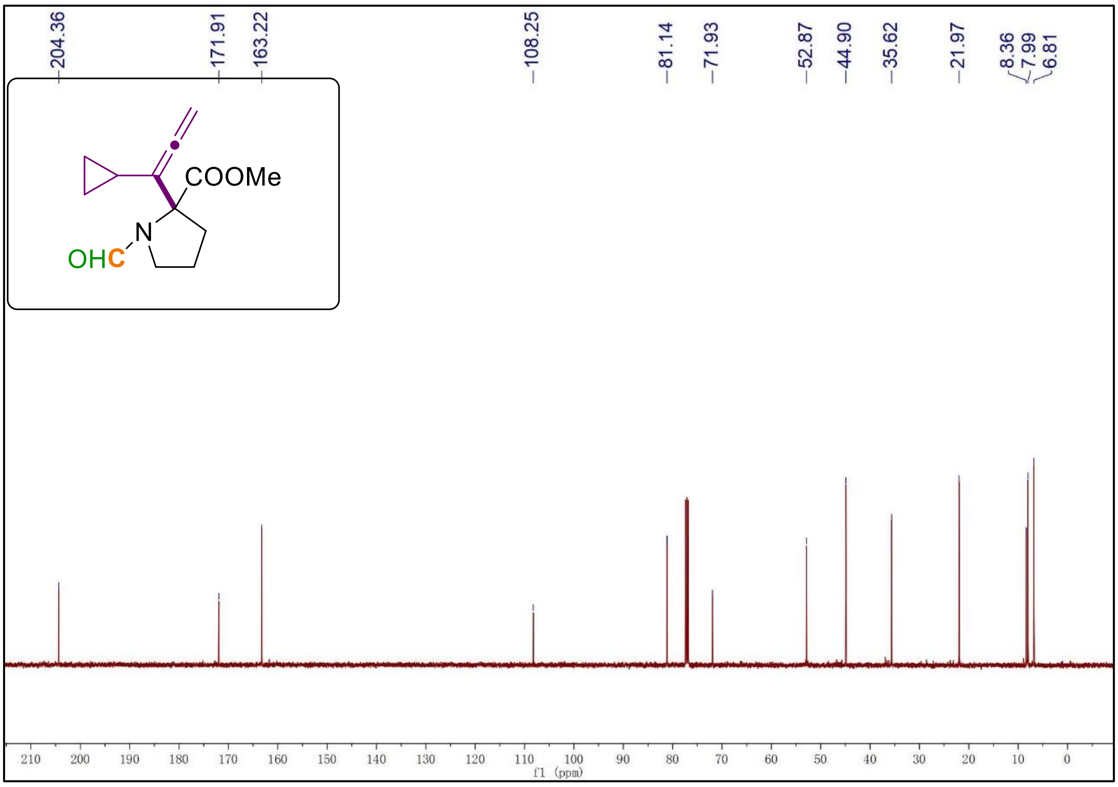

methyl 1-formyl-2-(hepta-1,2-dien-3-yl)pyrrolidine-2-carboxylate (9f)

^{1}H NMR (500 MHz, Chloroform-*d*)

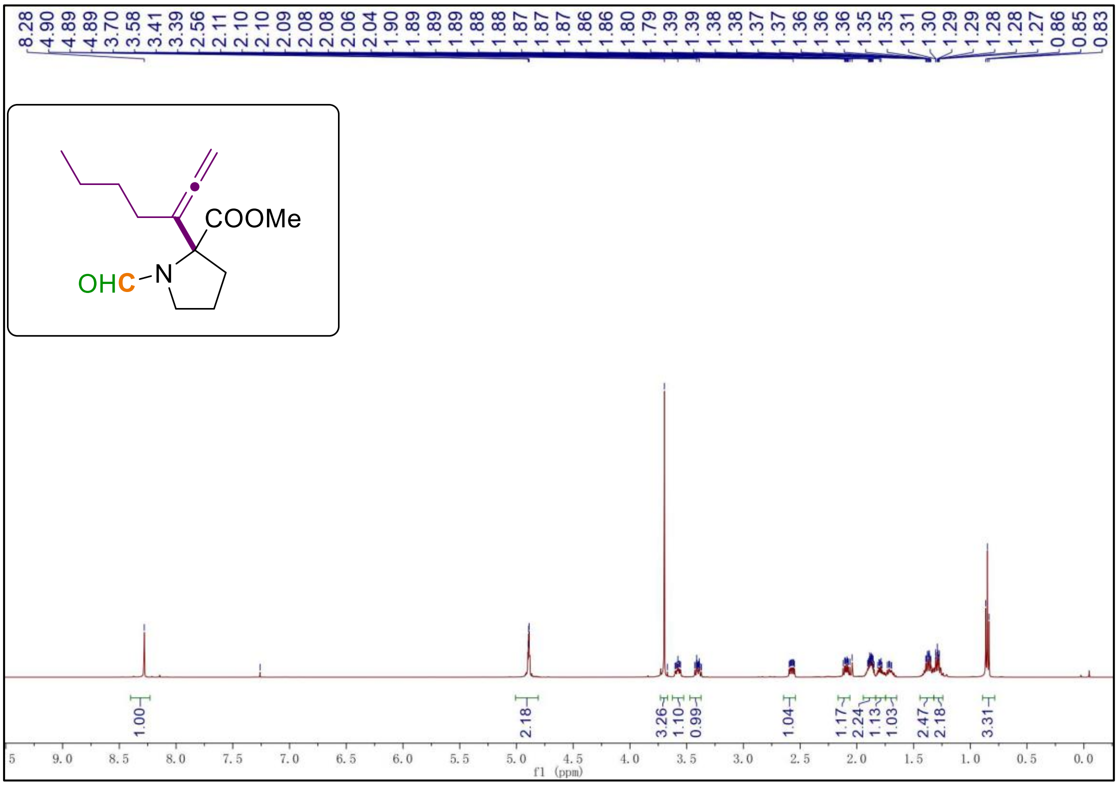

^{13}C NMR (126 MHz, Chloroform-*d*)

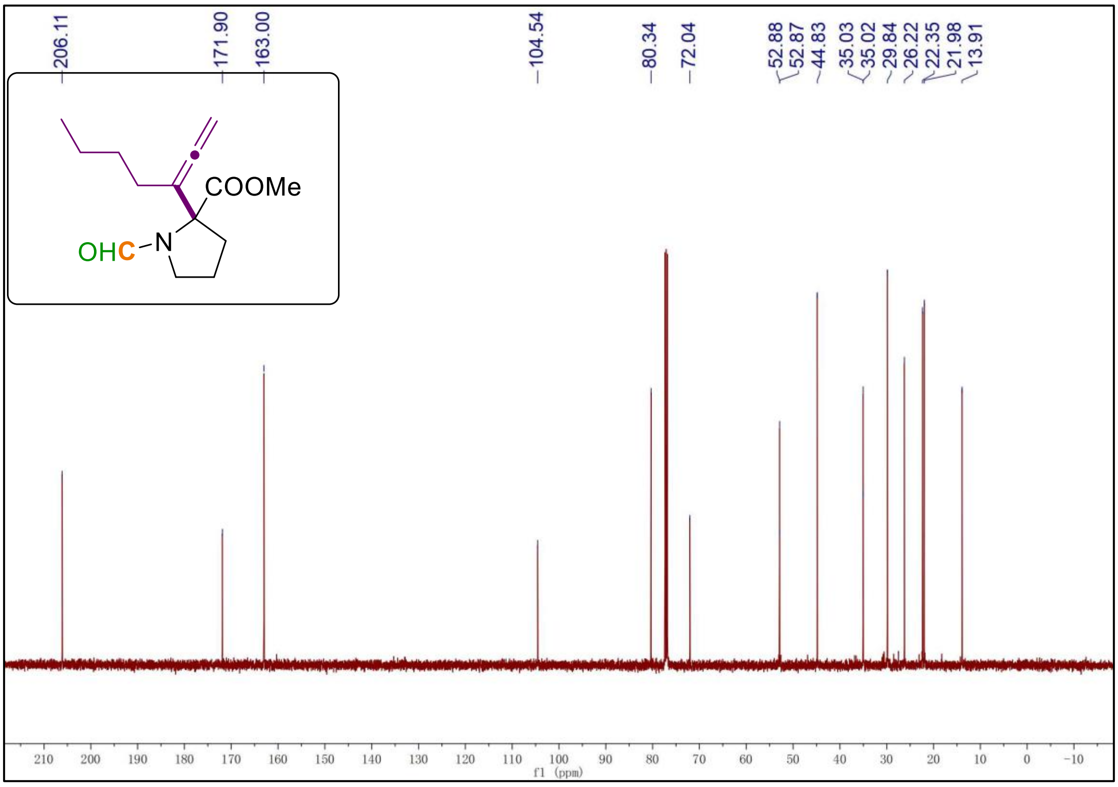

methyl 2-(4,4-dimethylpenta-1,2-dien-3-yl)-1-formylpyrrolidine-2-carboxylate (9g)

^{1}H NMR (500 MHz, Chloroform-*d*)

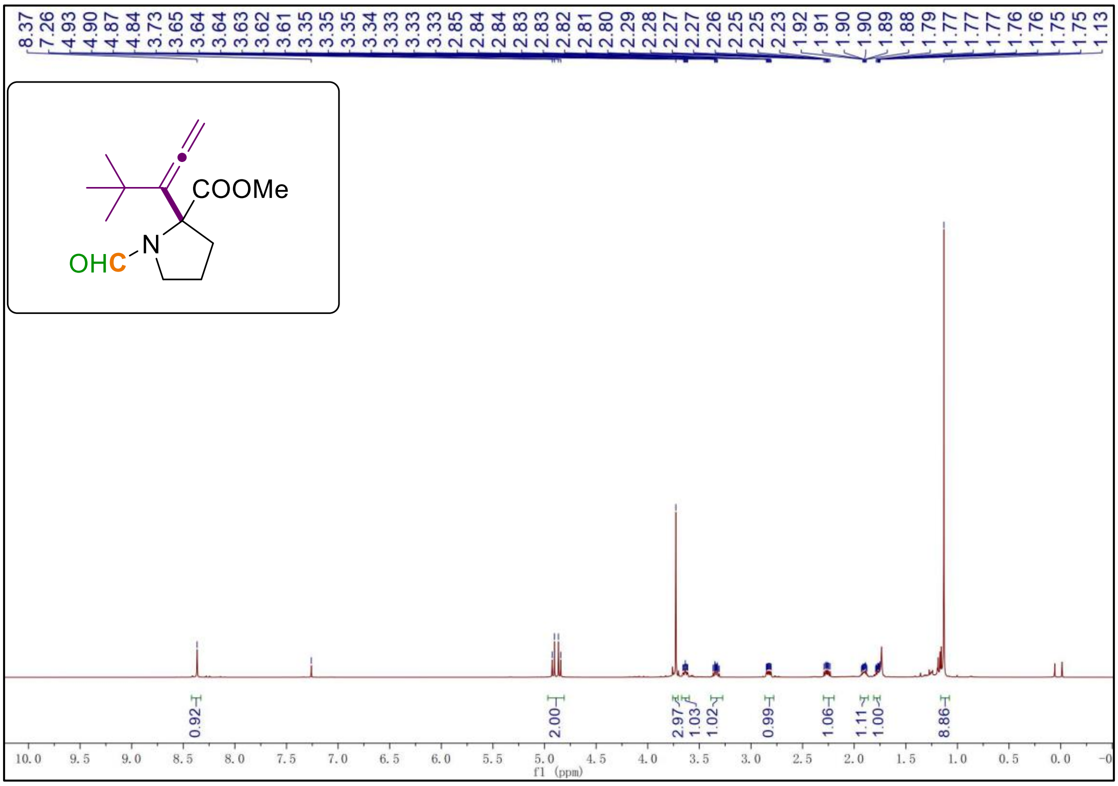

^{13}C NMR (126 MHz, Chloroform-*d*)

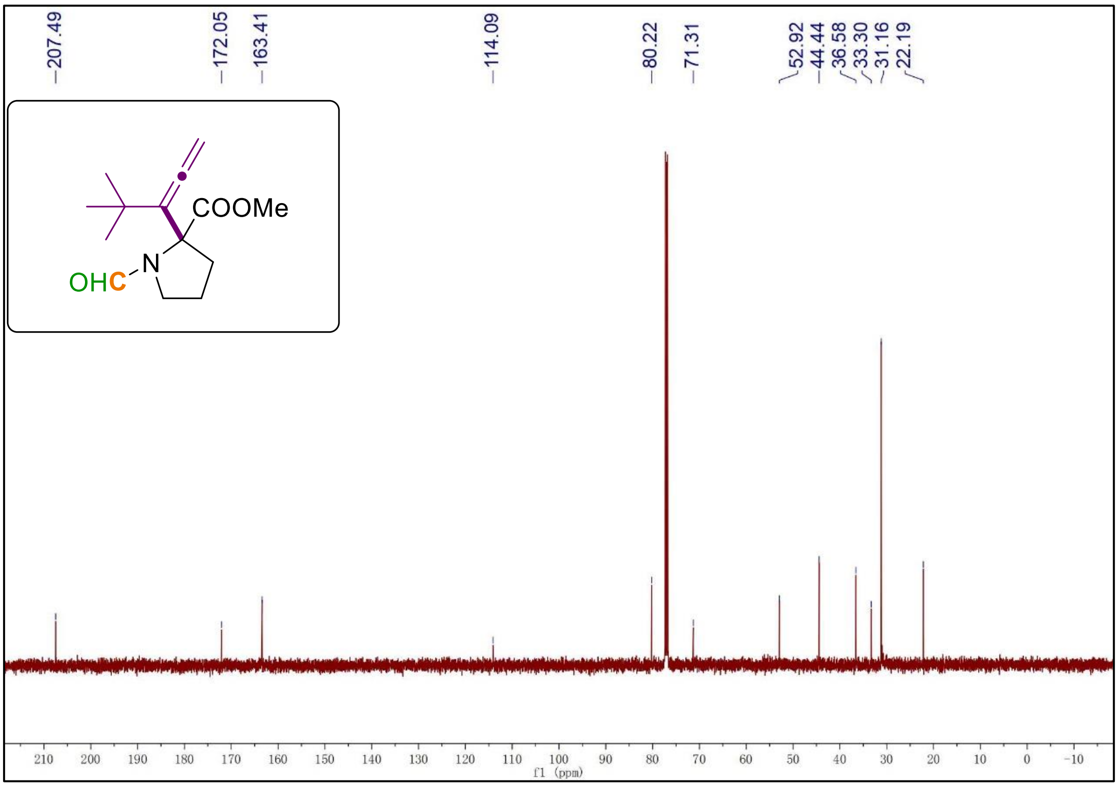

methyl 1-formyl-2-(1-((tetrahydro-2H-pyran-2-yl)oxy)buta-2,3-dien-2-yl)pyrrolidine-2-carboxylate (9h)

^{1}H NMR (500 MHz, Chloroform-*d*)

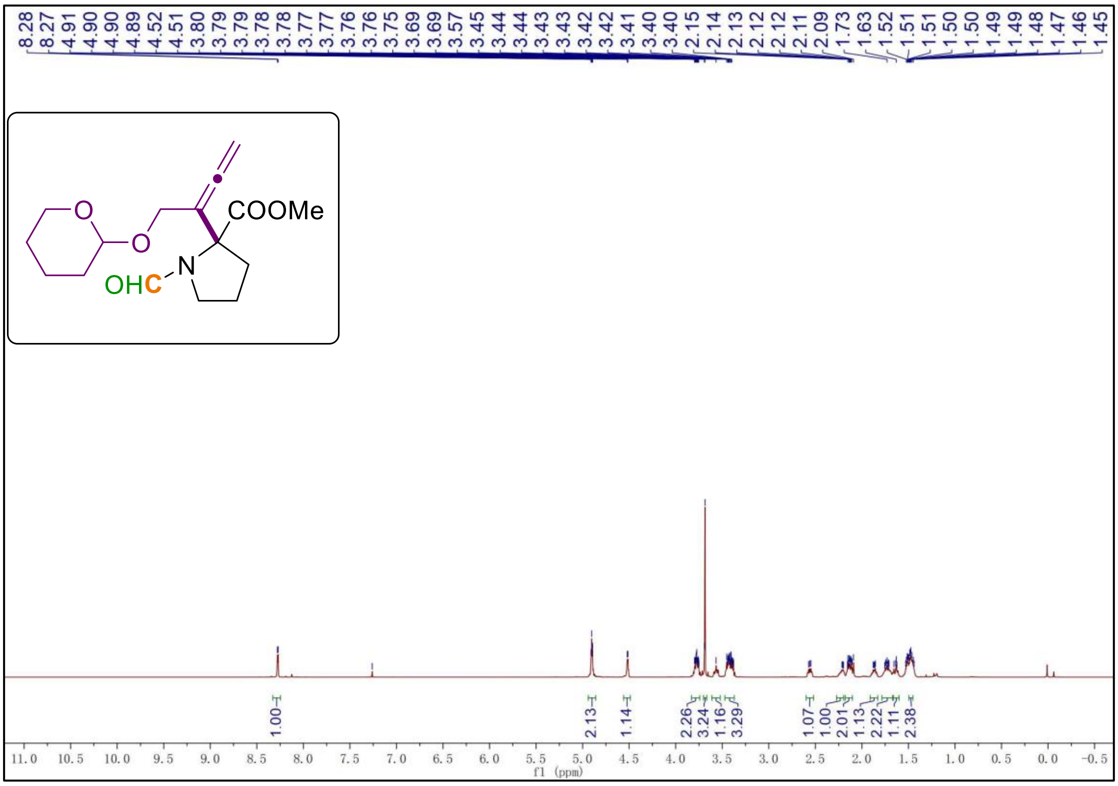

^{13}C NMR (126 MHz, Chloroform-*d*)

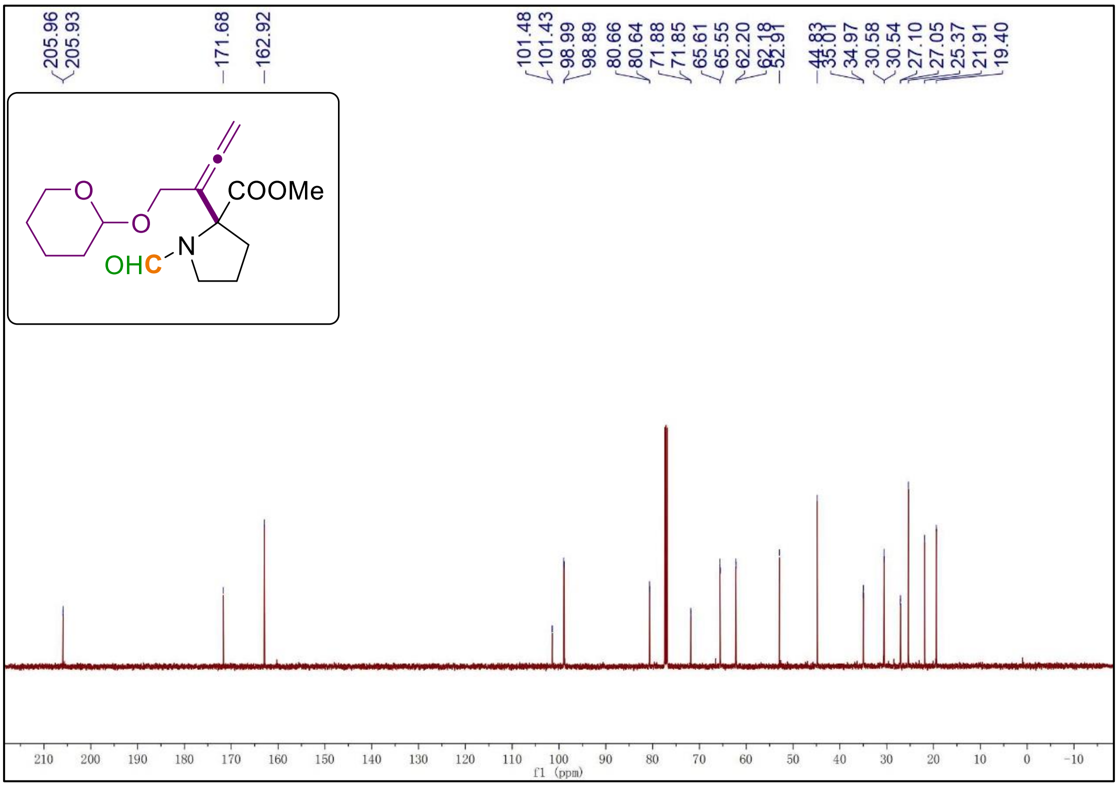

***tert*-butyl 4-(1-(1-formyl-2-(methoxycarbonyl)pyrrolidin-2-yl)propa-1,2-dien-1-yl)piperidine-1-carboxylate（9i)**

^{1}H NMR (500 MHz, Chloroform-*d*)

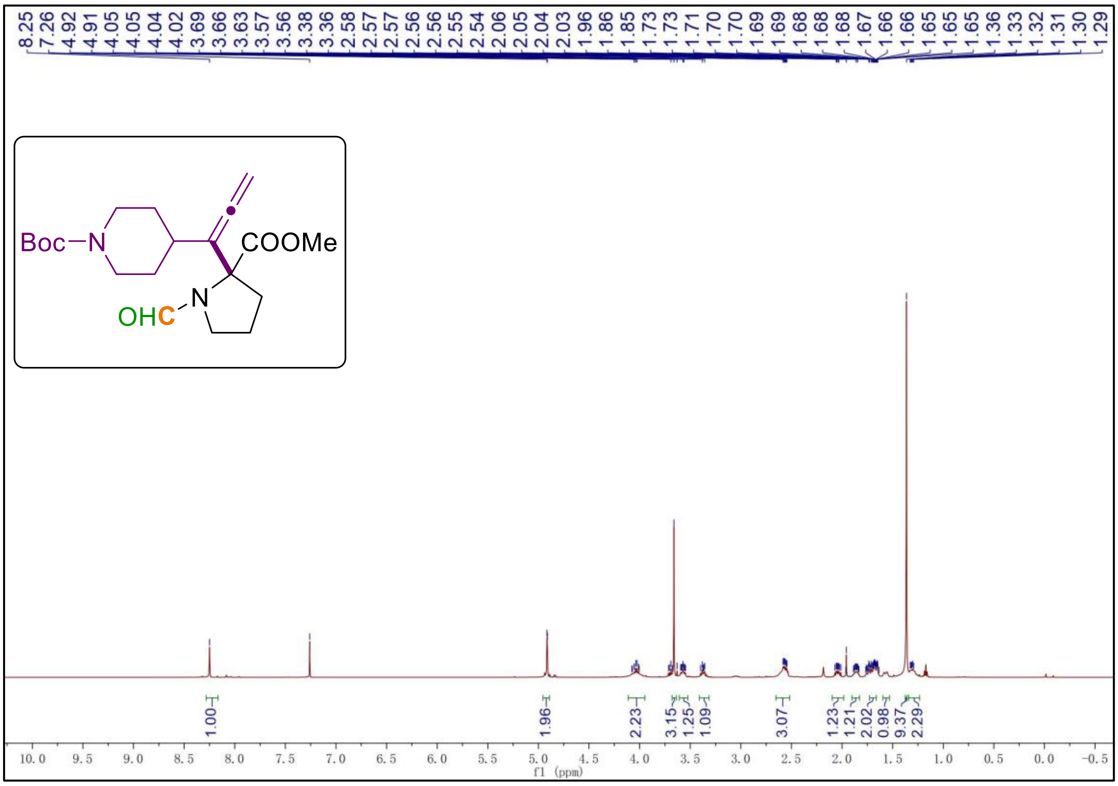

^{13}C NMR (126 MHz, Chloroform-*d*)

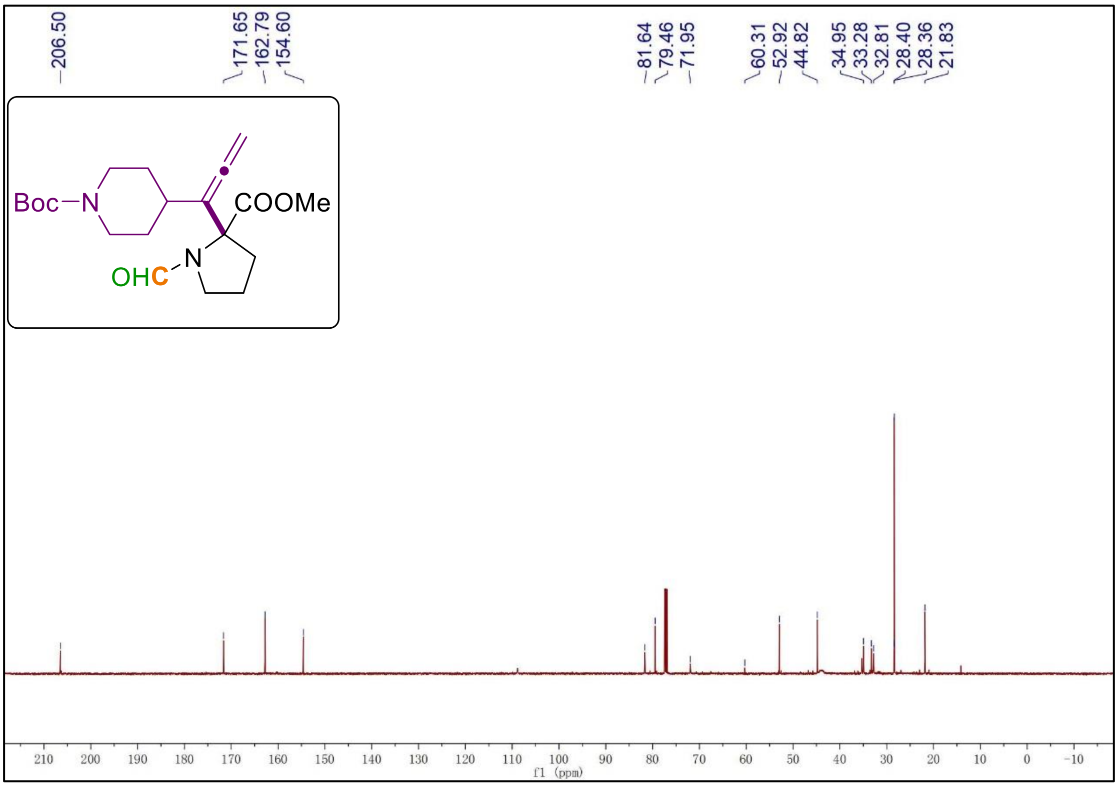

methyl 1-formyl-2-(1-(2-hydroxypropoxy)buta-2,3-dien-2-yl)pyrrolidine-2-carboxylate (9j)

^{1}H NMR (500 MHz, Chloroform-*d*)

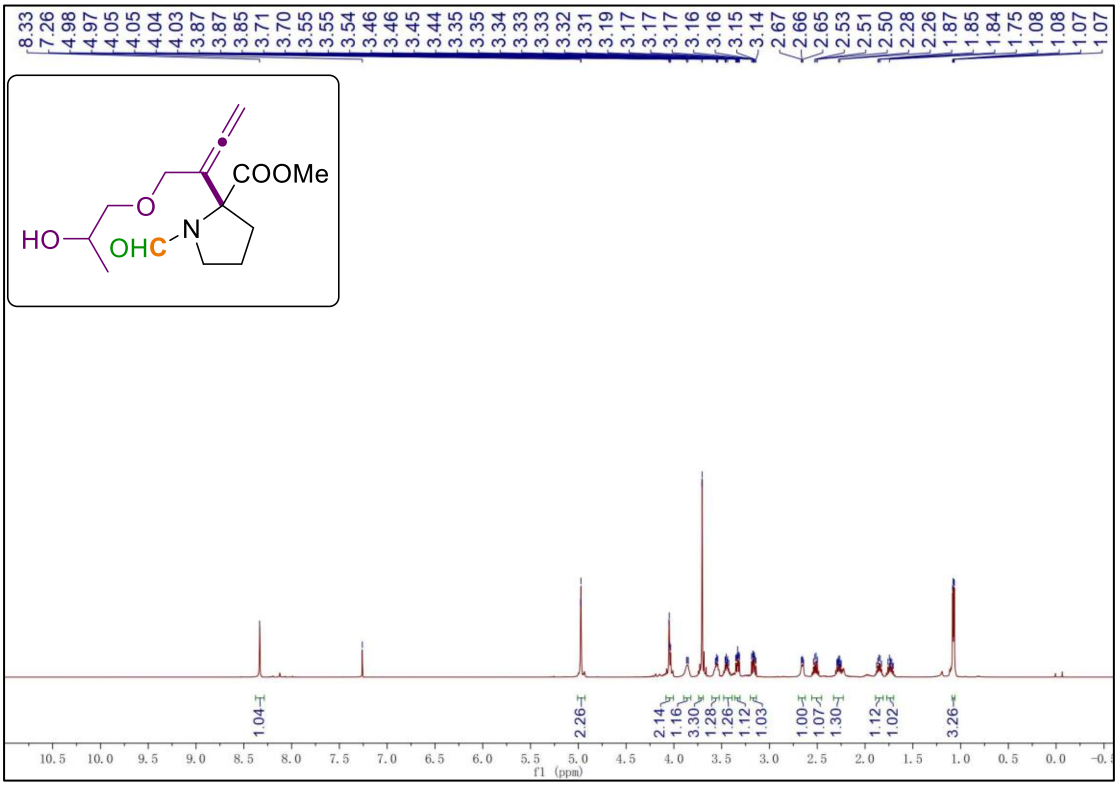

^{13}C NMR (126 MHz, Chloroform-*d*)

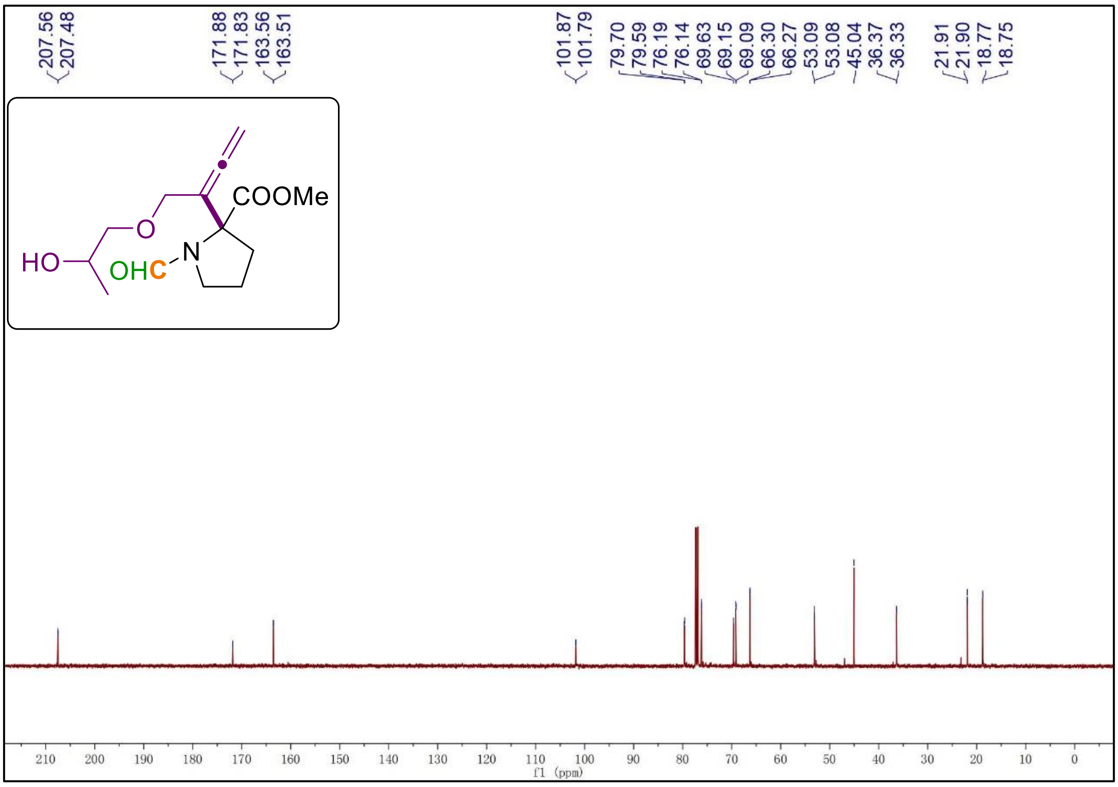

methyl 1-formyl-2-(4-methylpenta-1,2,4-trien-3-yl)pyrrolidine-2-carboxylate (9k)

^{1}H NMR (500 MHz, Chloroform-*d*)

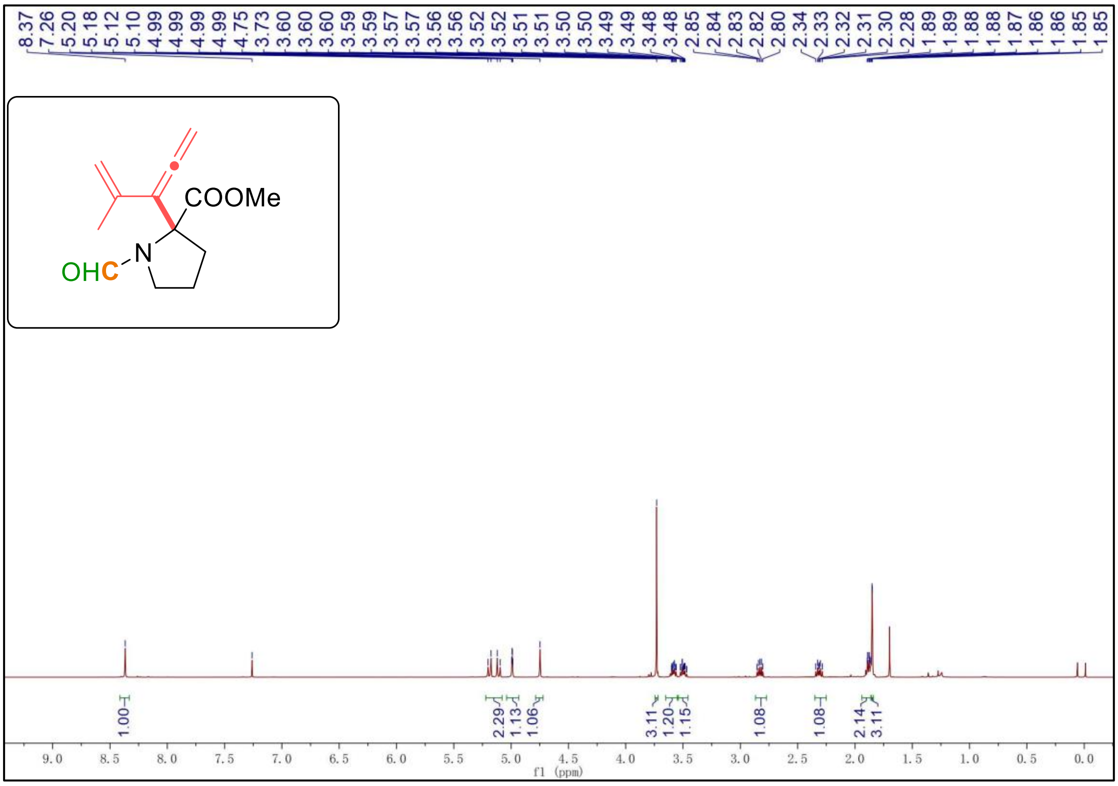

^{13}C NMR (126 MHz, Chloroform-*d*)

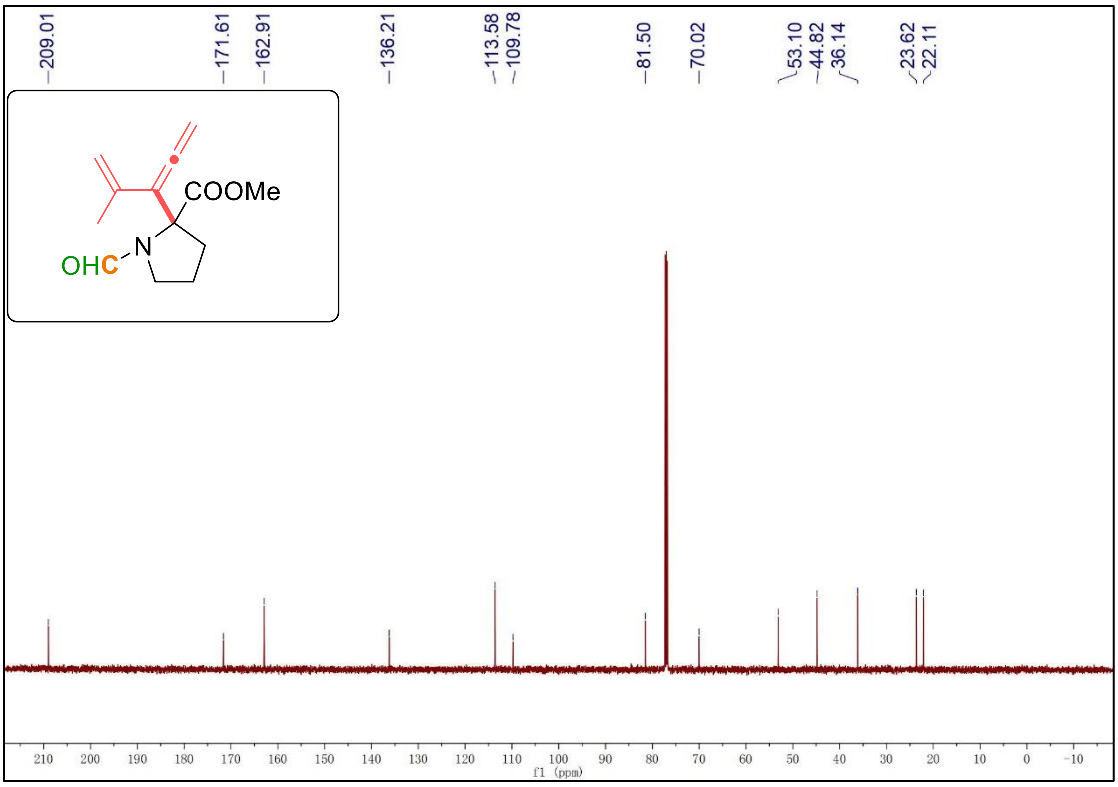

methyl 2-(1-(cyclohex-1-en-1-yl)propa-1,2-dien-1-yl)-1-formylpyrrolidine-2-carboxylate (9l)

^{1}H NMR (500 MHz, Chloroform-*d*)

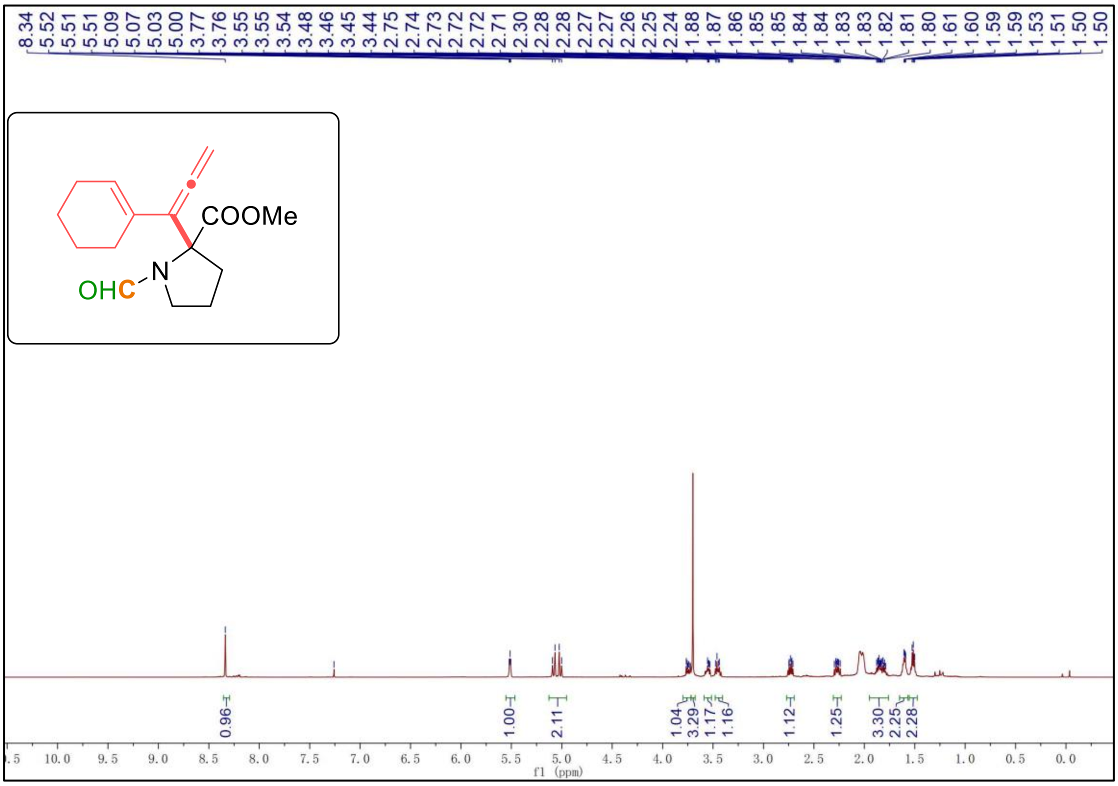

^{13}C NMR (126 MHz, Chloroform-*d*)

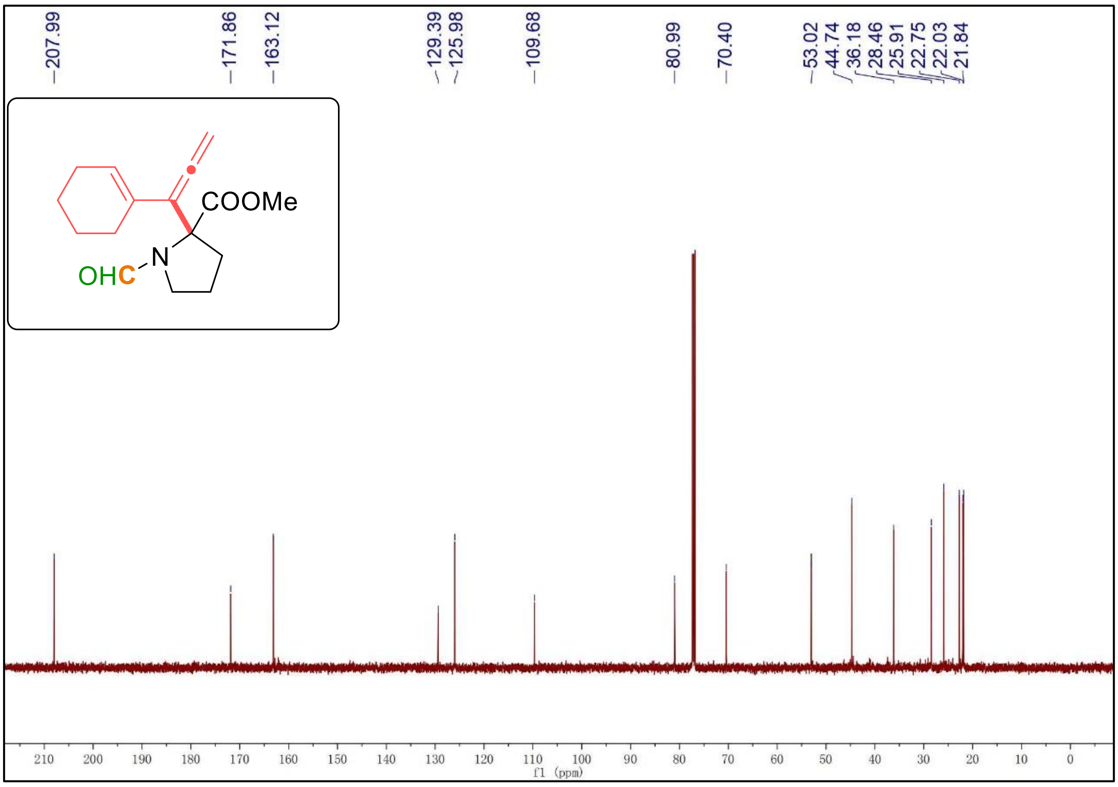

methyl 1-formyl-2-(1-phenylpropa-1,2-dien-1-yl)pyrrolidine-2-carboxylate (9m)

^{1}H NMR (500 MHz, Chloroform-*d*)

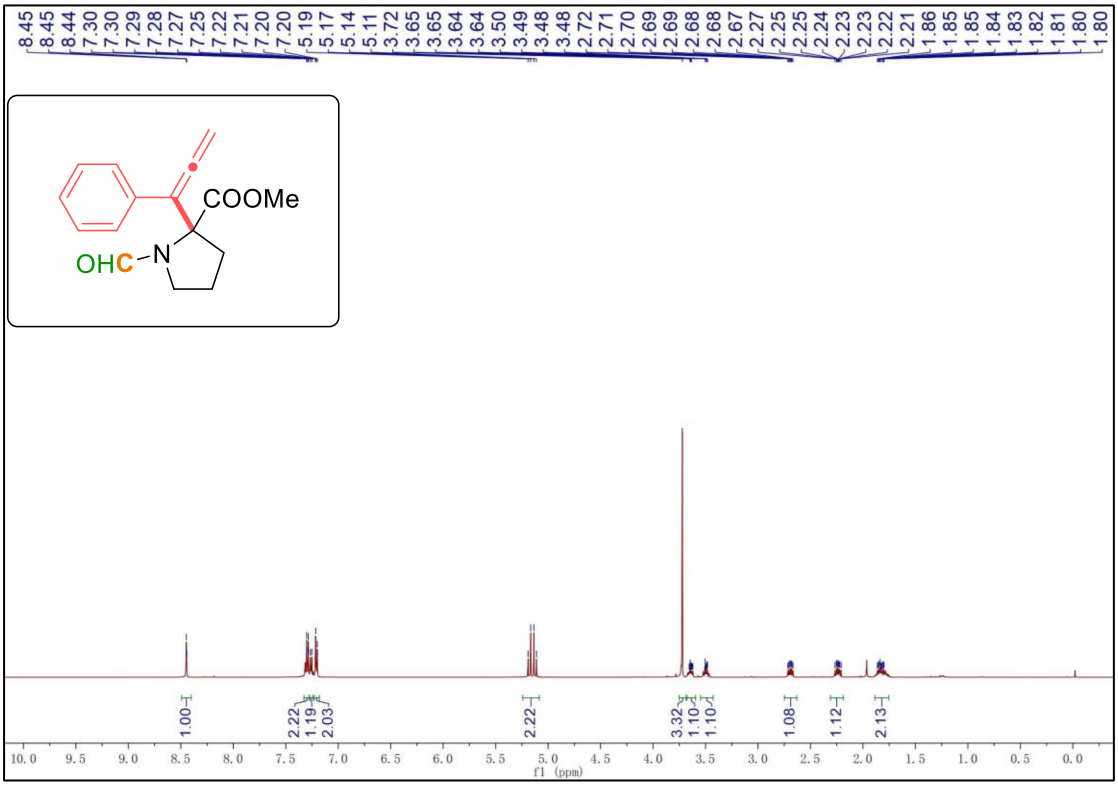

^{13}C NMR (126 MHz, Chloroform-*d*)

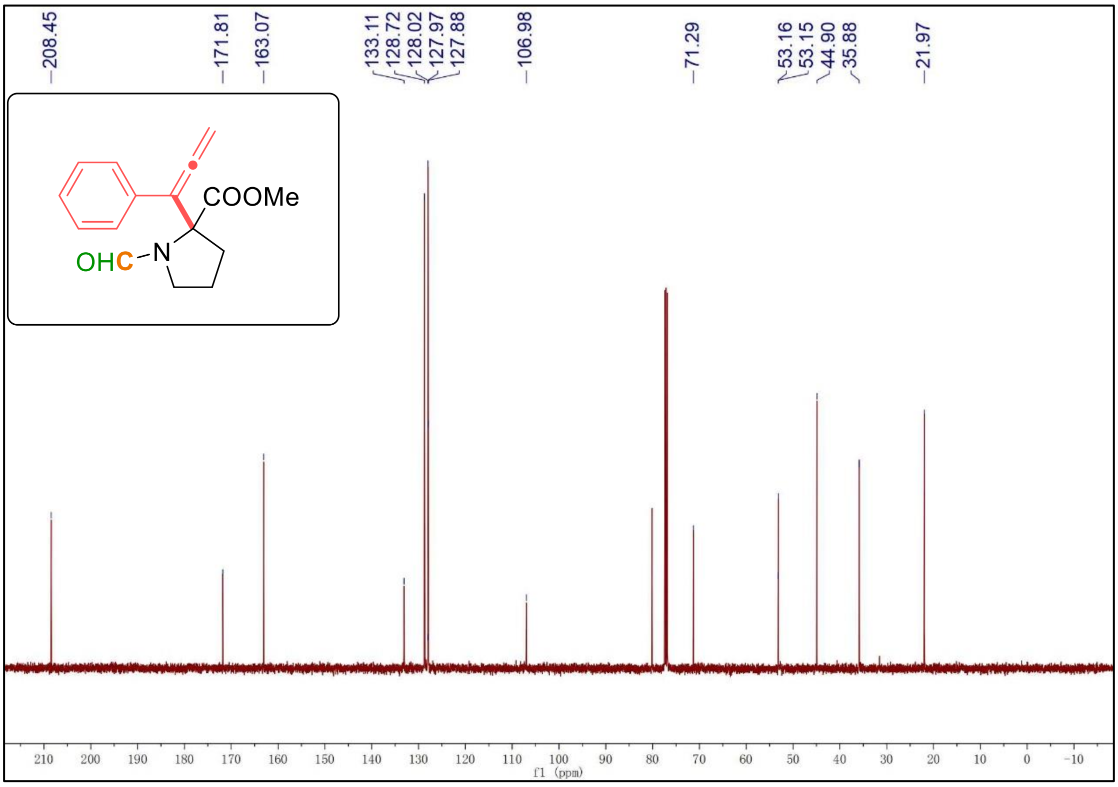

methyl 2-(1-(4-ethylphenyl)propa-1,2-dien-1-yl)-1-formylpyrrolidine-2-carboxylate (9n)

^{1}H NMR (500 MHz, Chloroform-*d*)

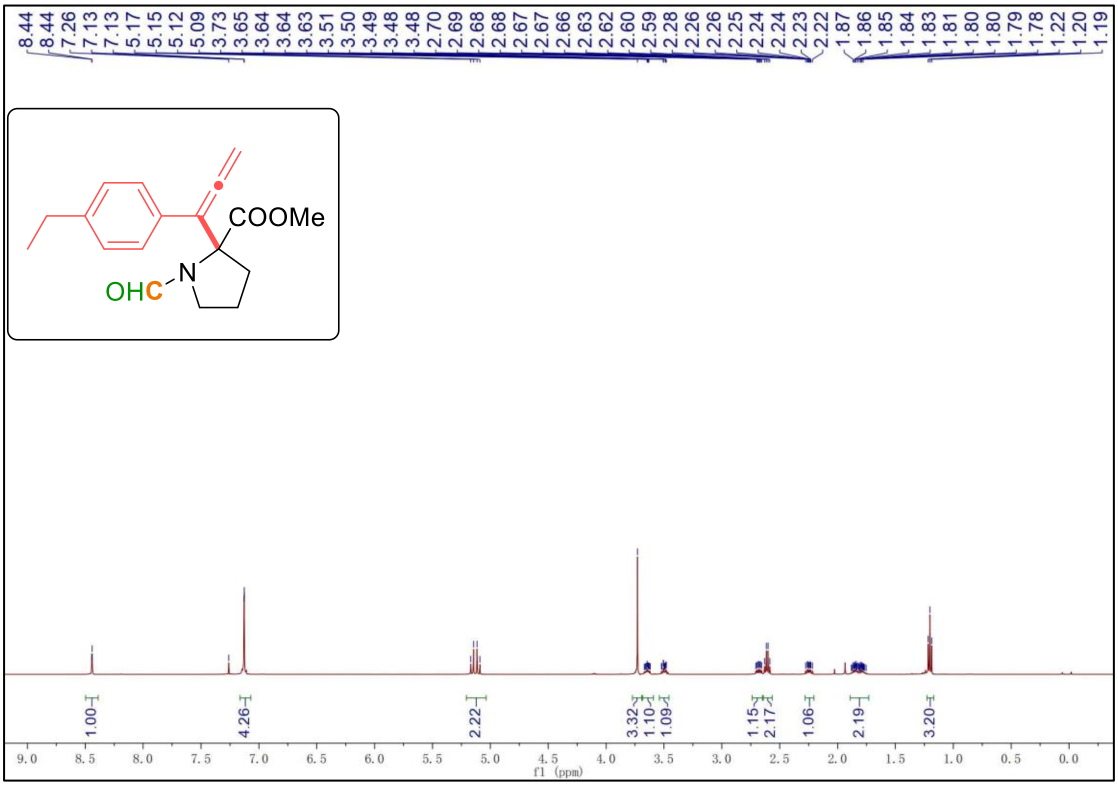

^{13}C NMR (126 MHz, Chloroform-*d*)

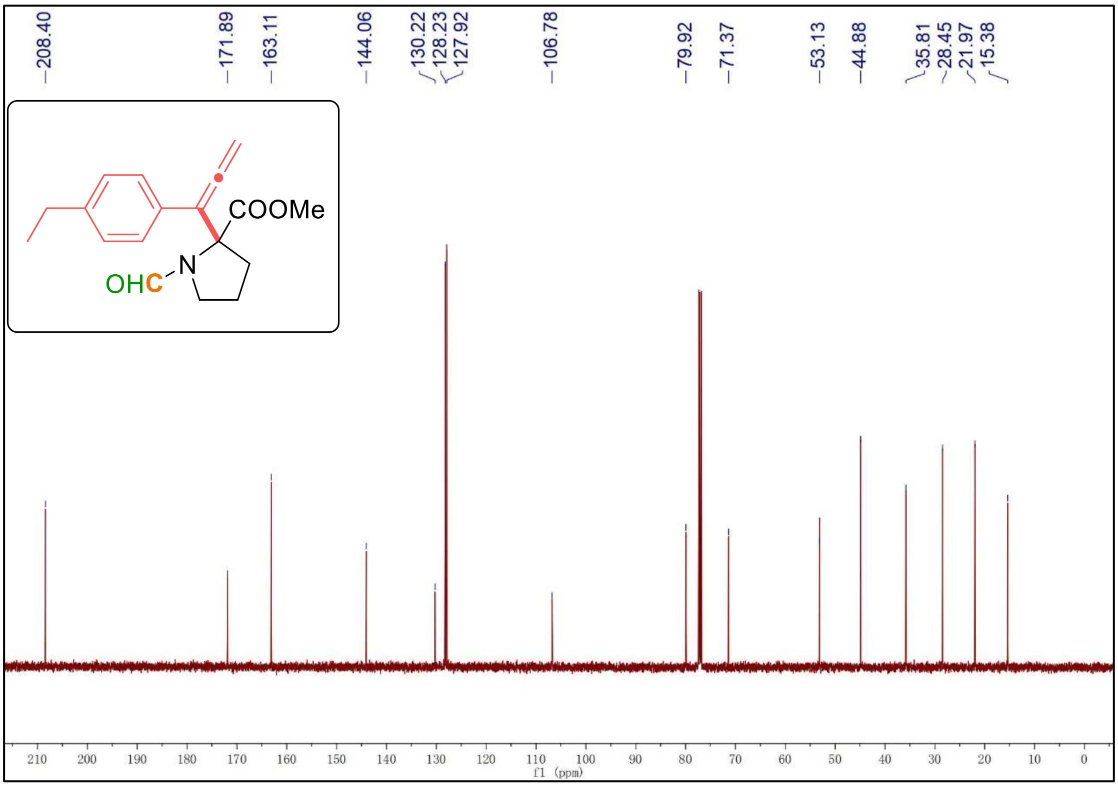

methyl 1-formyl-2-(1-(4-methoxyphenyl)propa-1,2-dien-1-yl)pyrrolidine-2-carboxylate (9o)

^{1}H NMR (500 MHz, Chloroform-*d*)

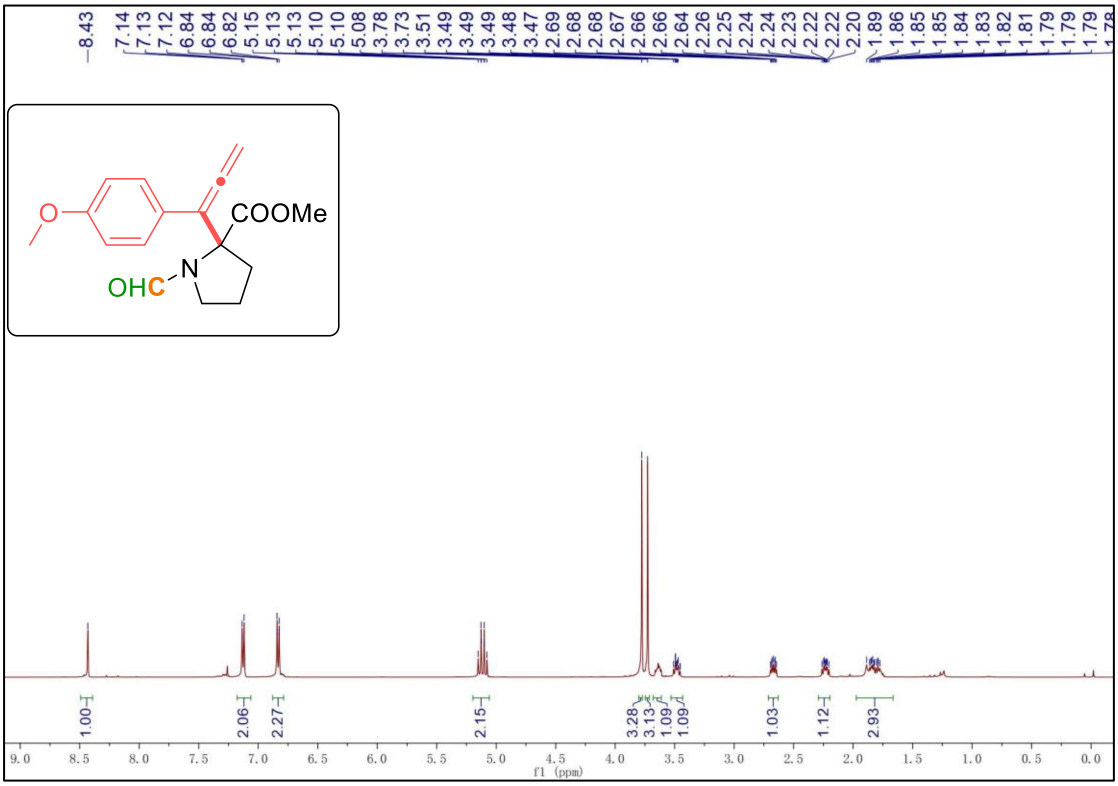

^{13}C NMR (126 MHz, Chloroform-*d*)

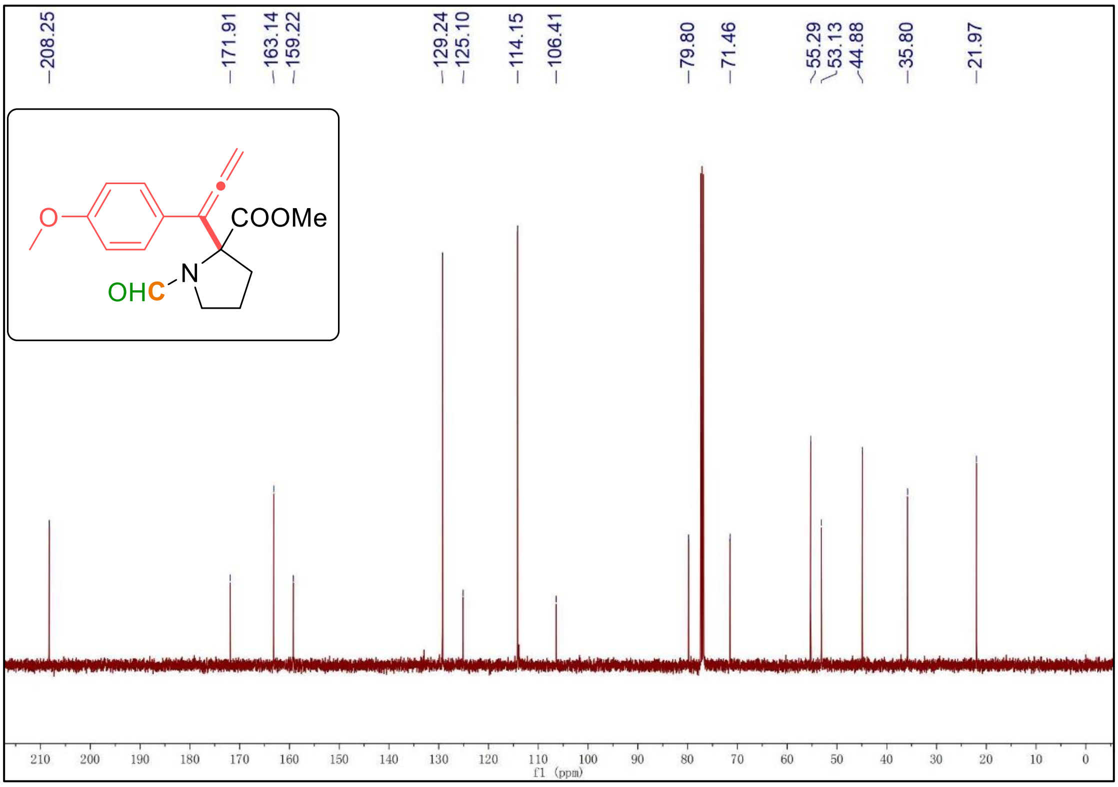

methyl 2-(1-(4-chlorophenyl)propa-1,2-dien-1-yl)-1-formylpyrrolidine-2-carboxylate (9p)

^{1}H NMR (500 MHz, Chloroform-*d*)

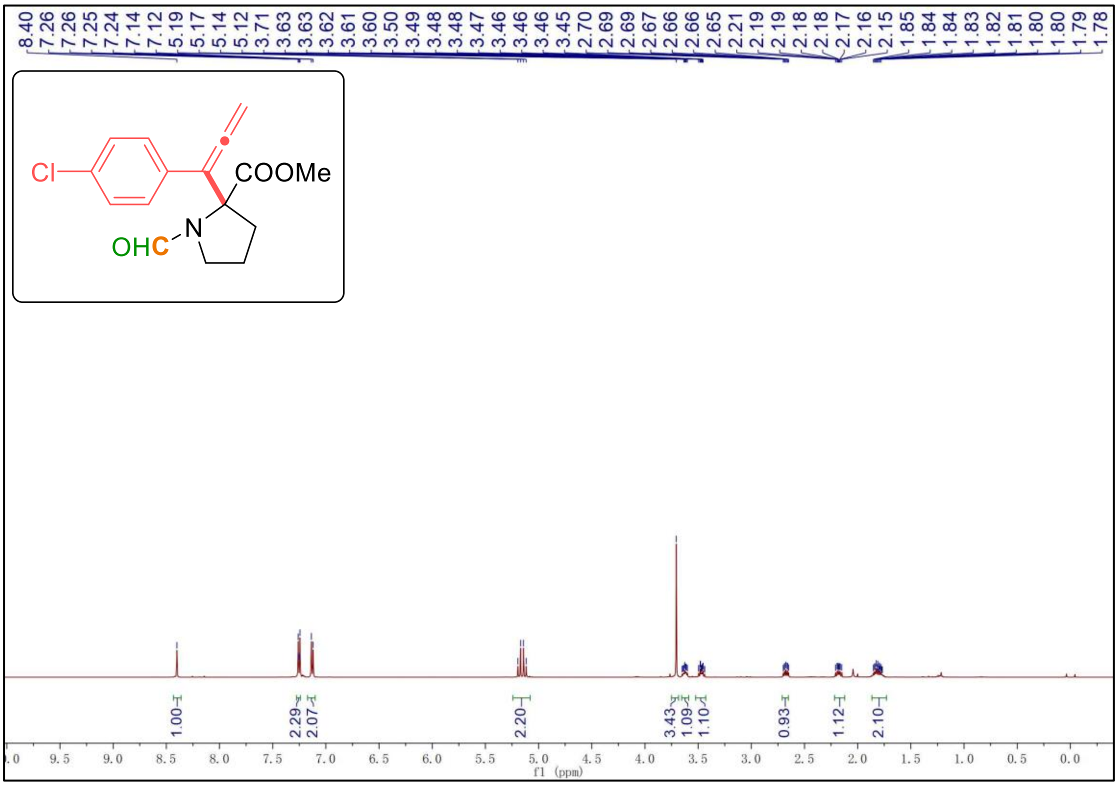

^{13}C NMR (126 MHz, Chloroform-*d*)

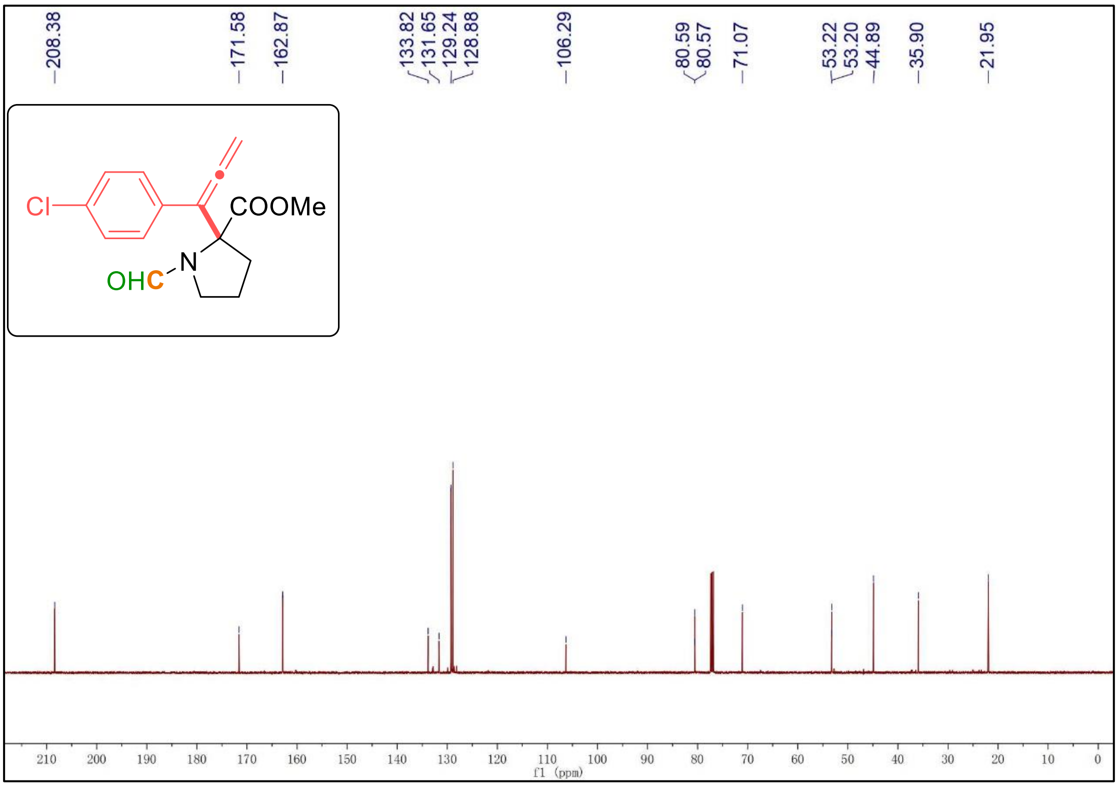

methyl 2-(1-(4-bromophenyl)propa-1,2-dien-1-yl)-1-formylpyrrolidine-2-carboxylate (9q)

^{1}H NMR (500 MHz, Chloroform-*d*)

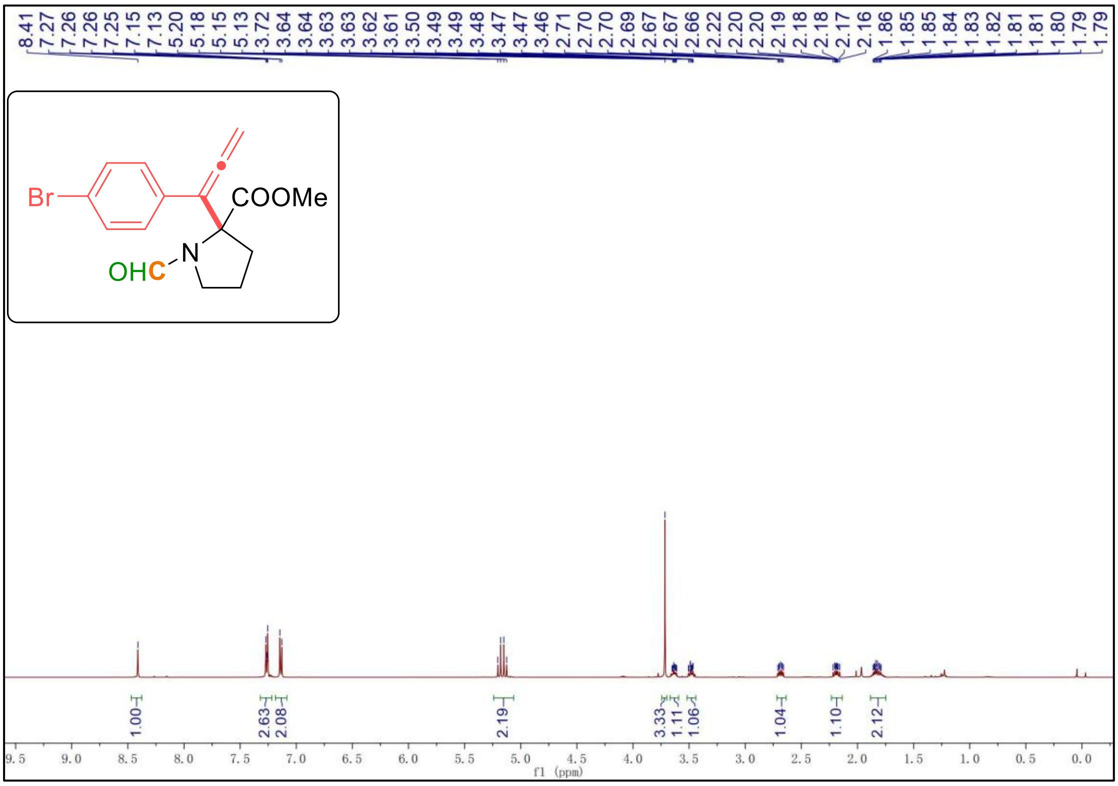

^{13}C NMR (126 MHz, Chloroform-*d*)

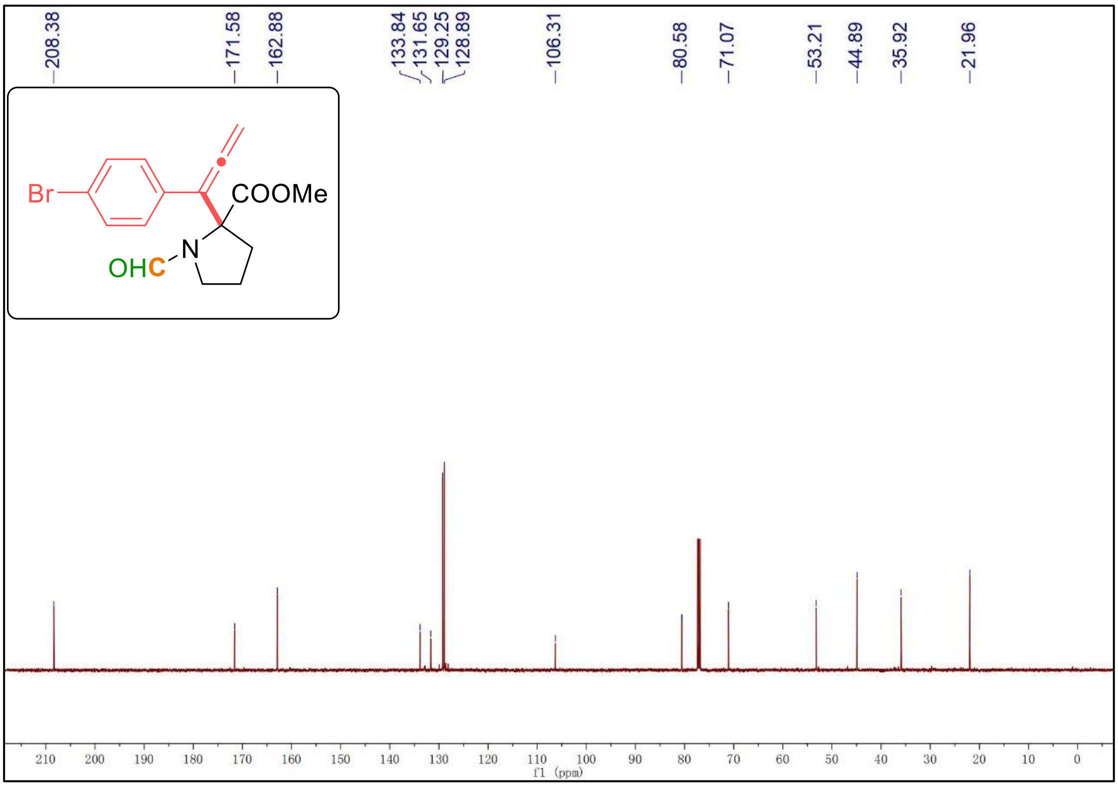

methyl 1-formyl-2-(1-(4-formylphenyl)propa-1,2-dien-1-yl)pyrrolidine-2-carboxylate (9r)

^{1}H NMR (500 MHz, Chloroform-*d*)

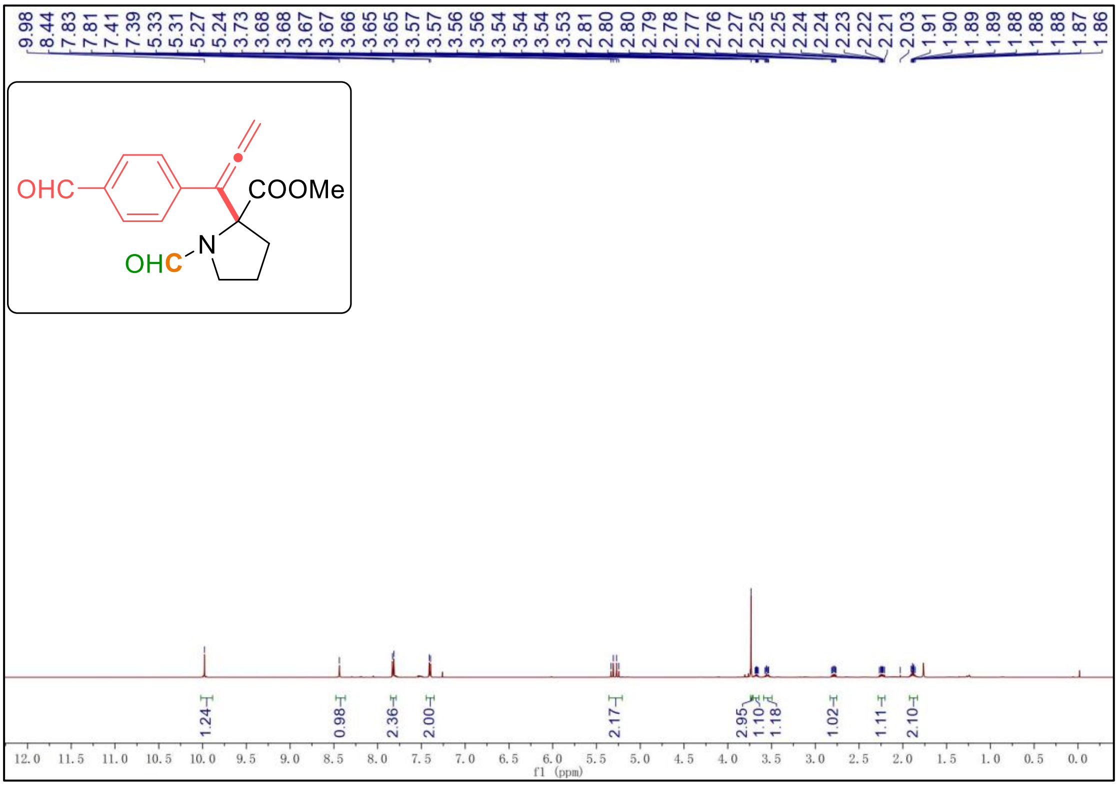

^{13}C NMR (126 MHz, Chloroform-*d*)

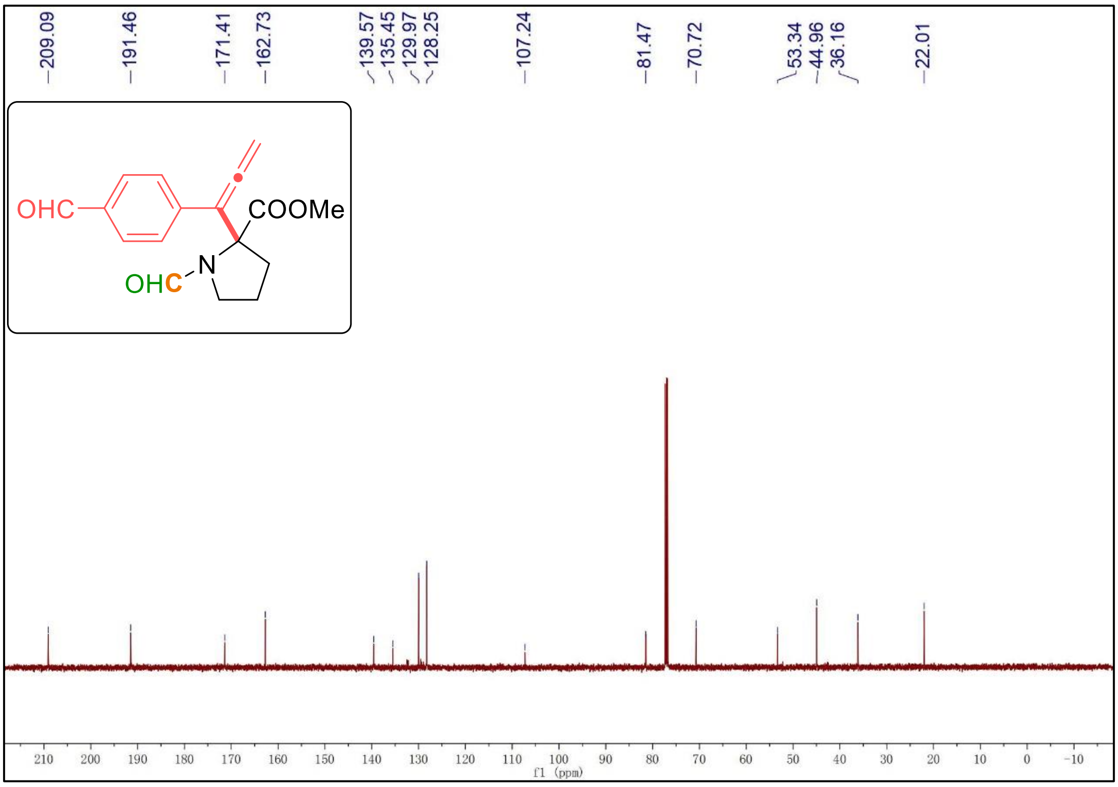

(Z)-hex-3-en-1-yl 2-(*N*-methylformamido)pent-4-enoate (11a)

^{1}H NMR (500 MHz, Chloroform-*d*)

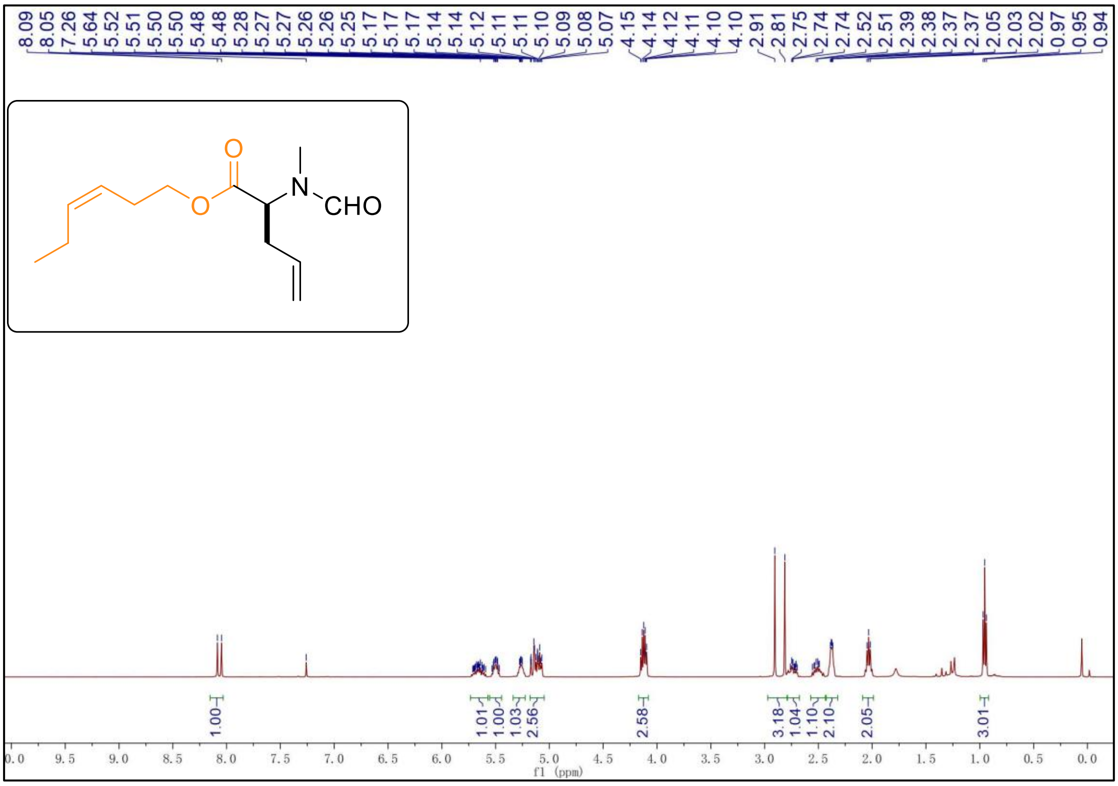

^{13}C NMR (126 MHz, Chloroform-*d*)

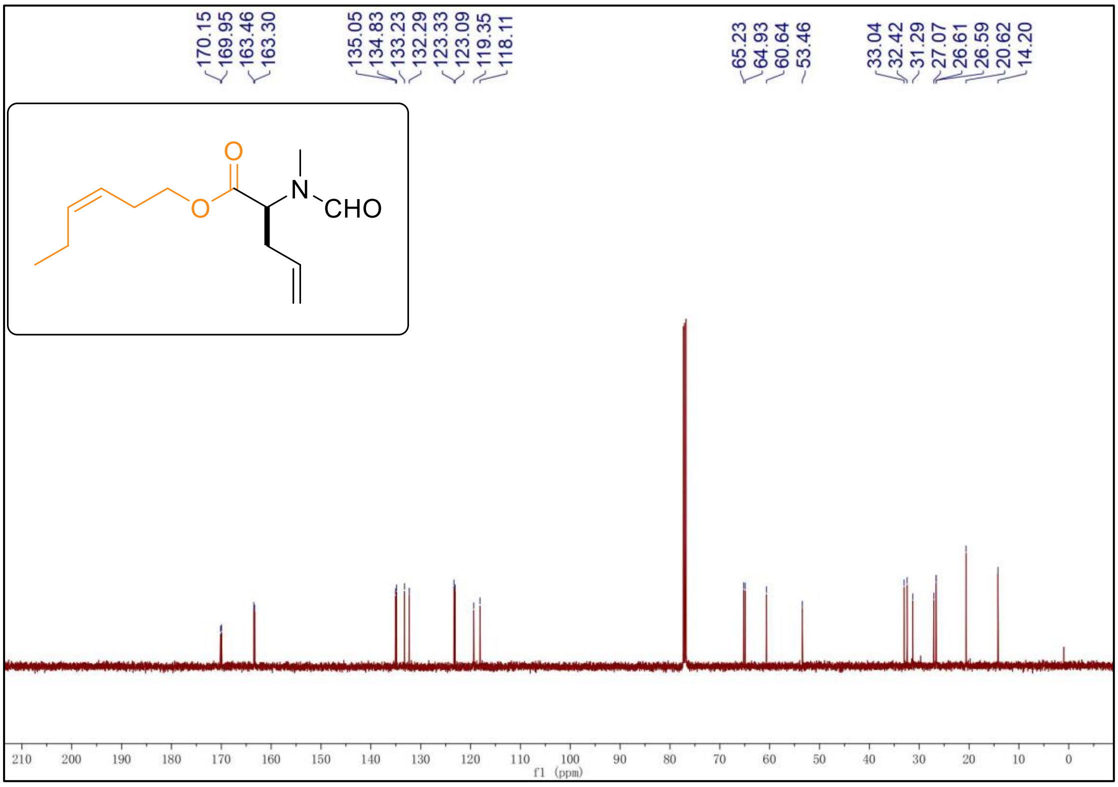

(S)-3,7-dimethyloct-6-en-1-yl 2-(*N*-methylformamido)pent-4-enoate (11b)

^{1}H NMR (500 MHz, Chloroform-*d*)

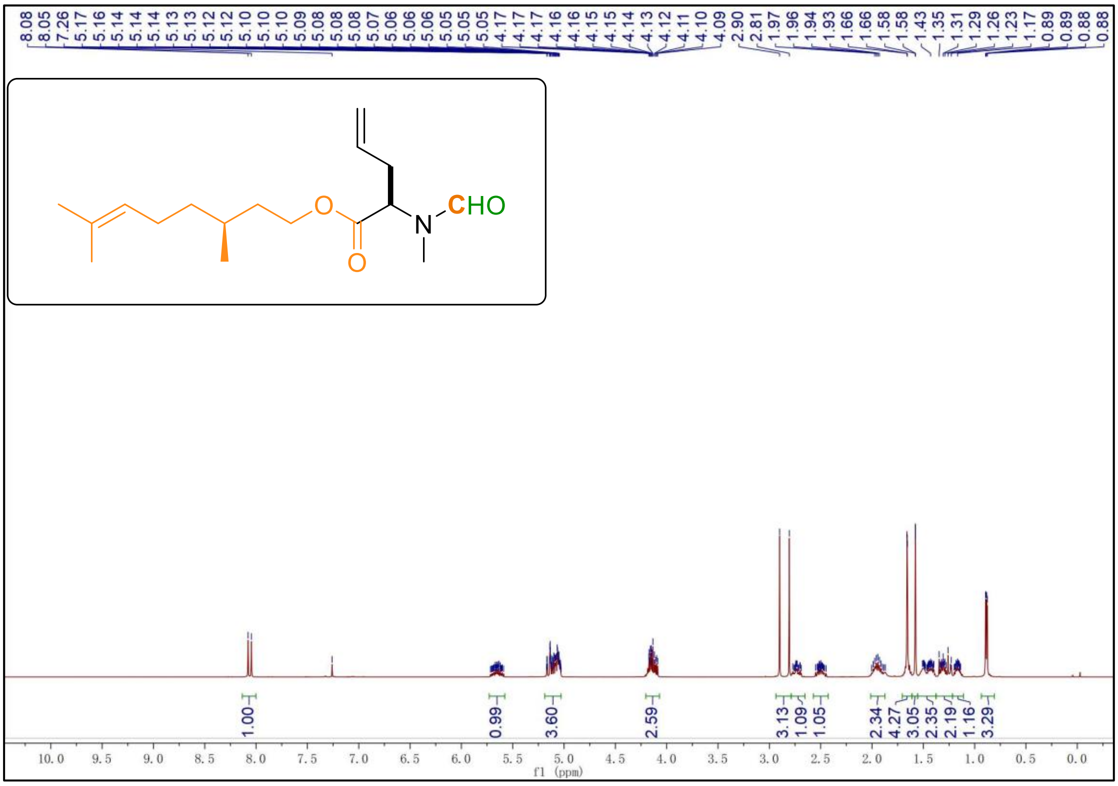

^{13}C NMR (126 MHz, Chloroform-*d*)

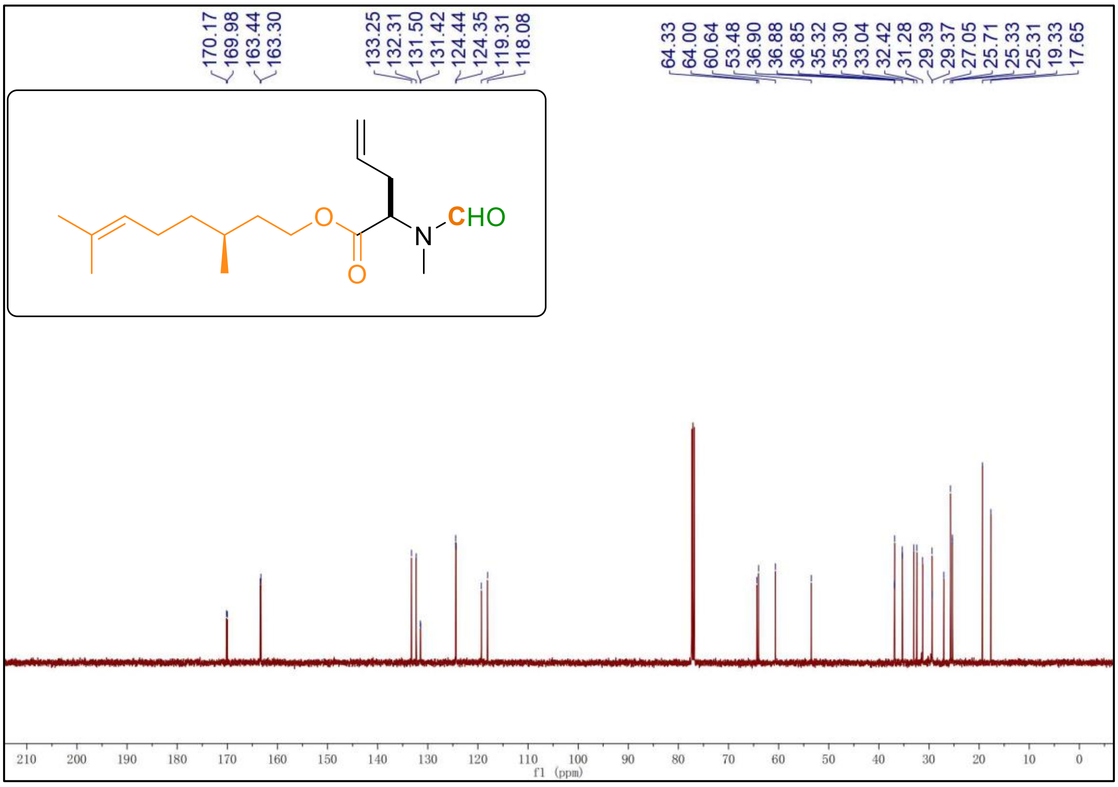

(E)-3,7-dimethylocta-2,6-dien-1-yl 2-(*N*-methylformamido)pent-4-enoate (11c)

^{1}H NMR (500 MHz, Chloroform-*d*)

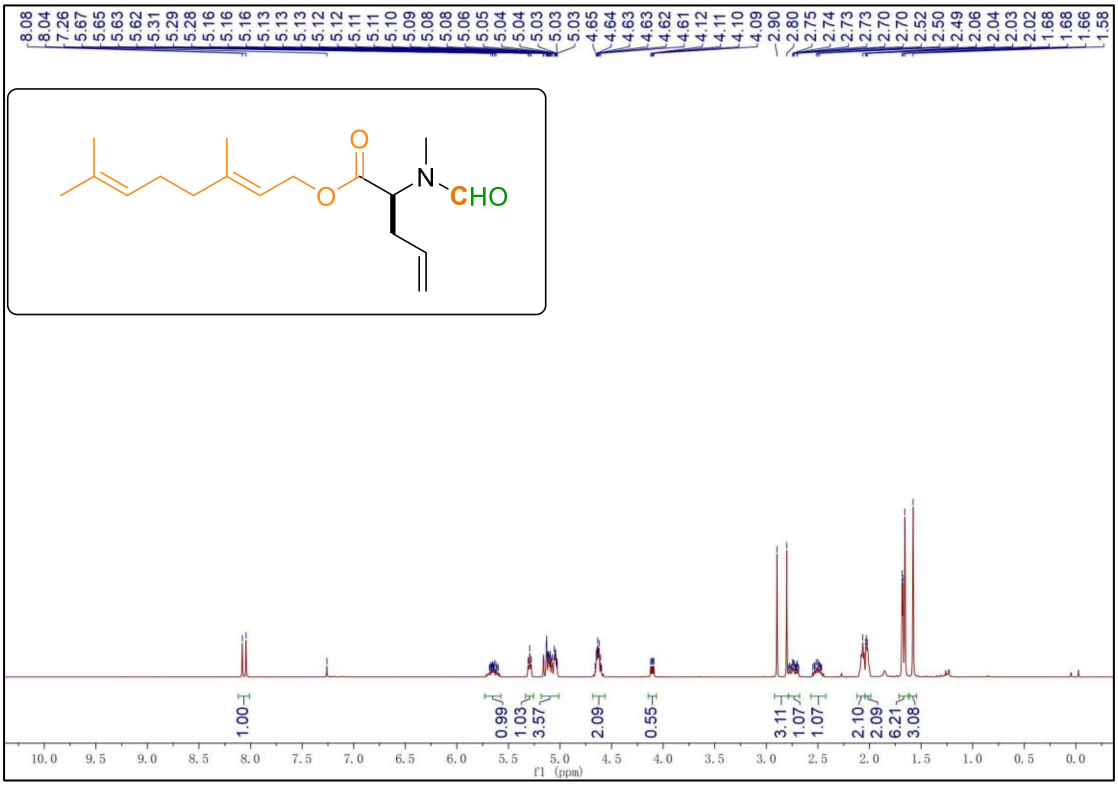

^{13}C NMR (126 MHz, Chloroform-*d*)

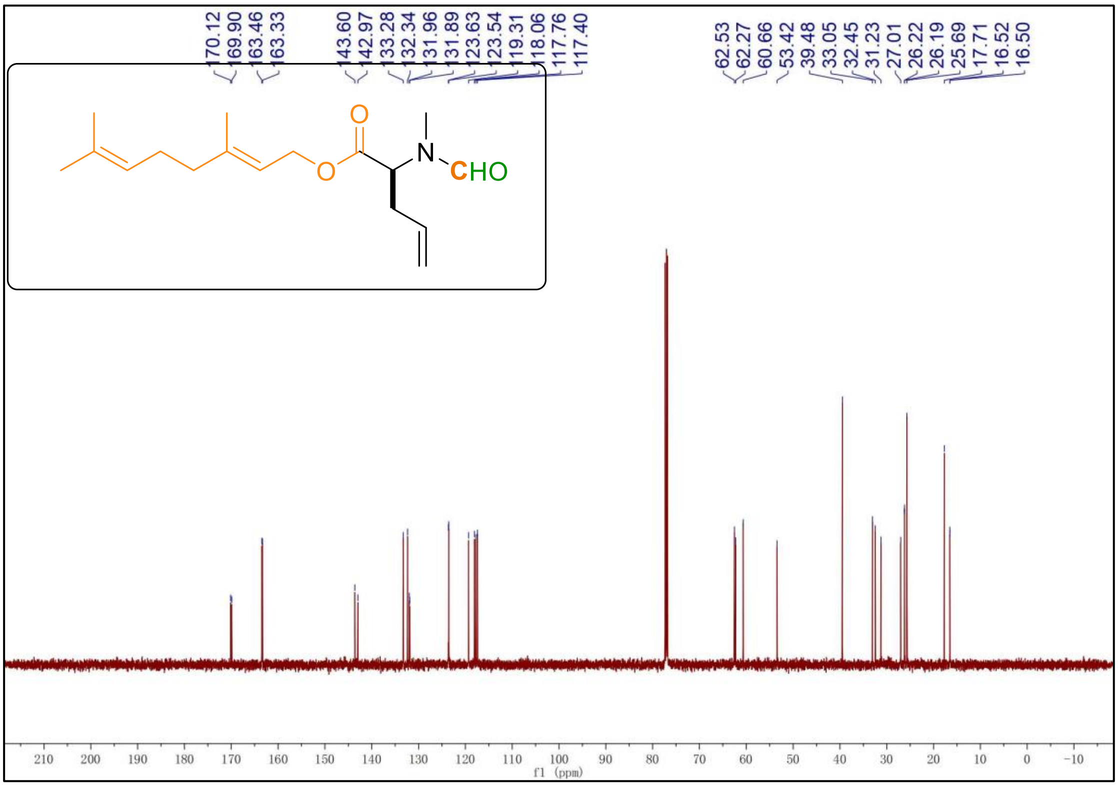

1,3,3-trimethylbicyclo[2.2.1]heptan-2-yl 2-(*N*-methylformamido)pent-4-enoate (11d)

^{1}H NMR (500 MHz, Chloroform-*d*)

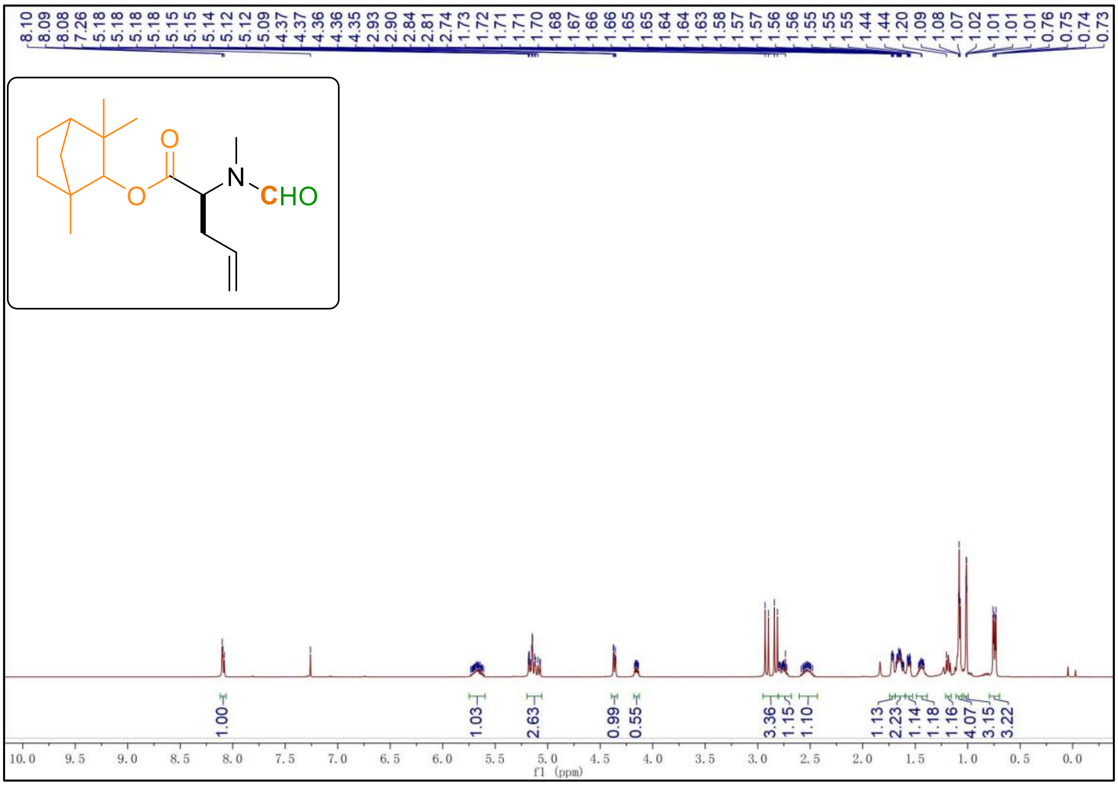

^{13}C NMR (126 MHz, Chloroform-*d*)

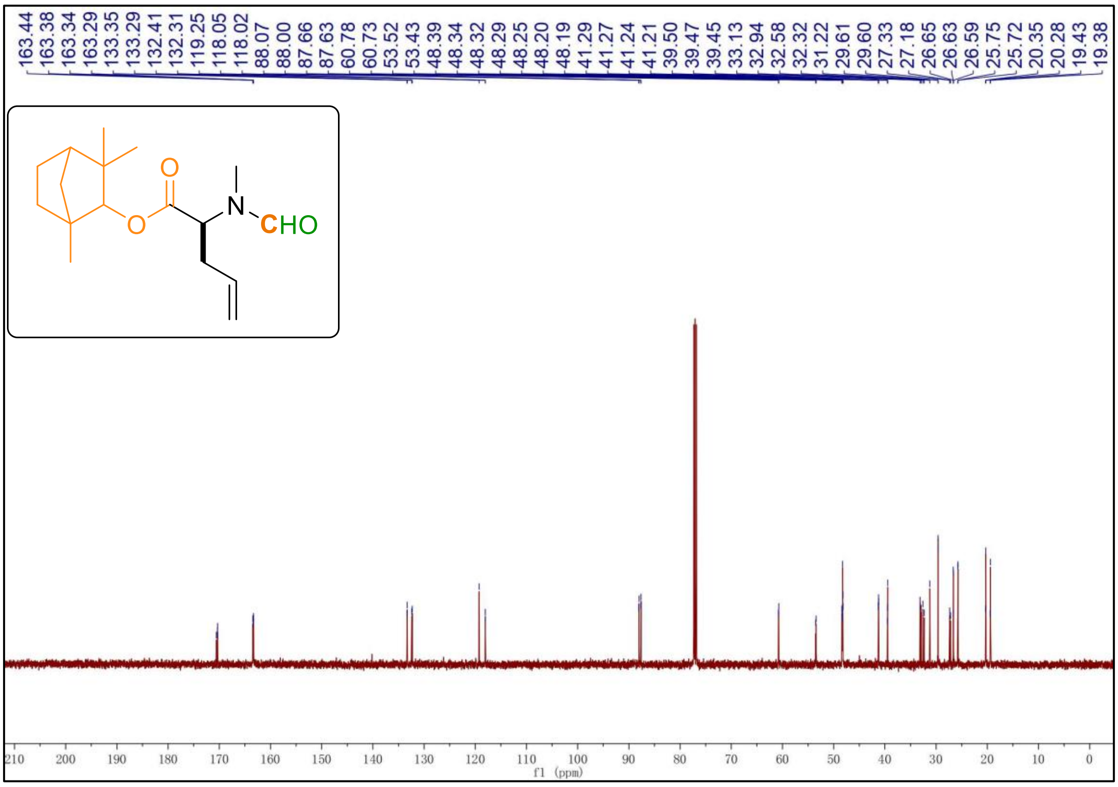

(1S,4S)-1,7,7-trimethylbicyclo[2.2.1]heptan-2-yl 2-(*N*-methylformamido)pent-4-enoate (11e)

^{1}H NMR (500 MHz, Chloroform-*d*)

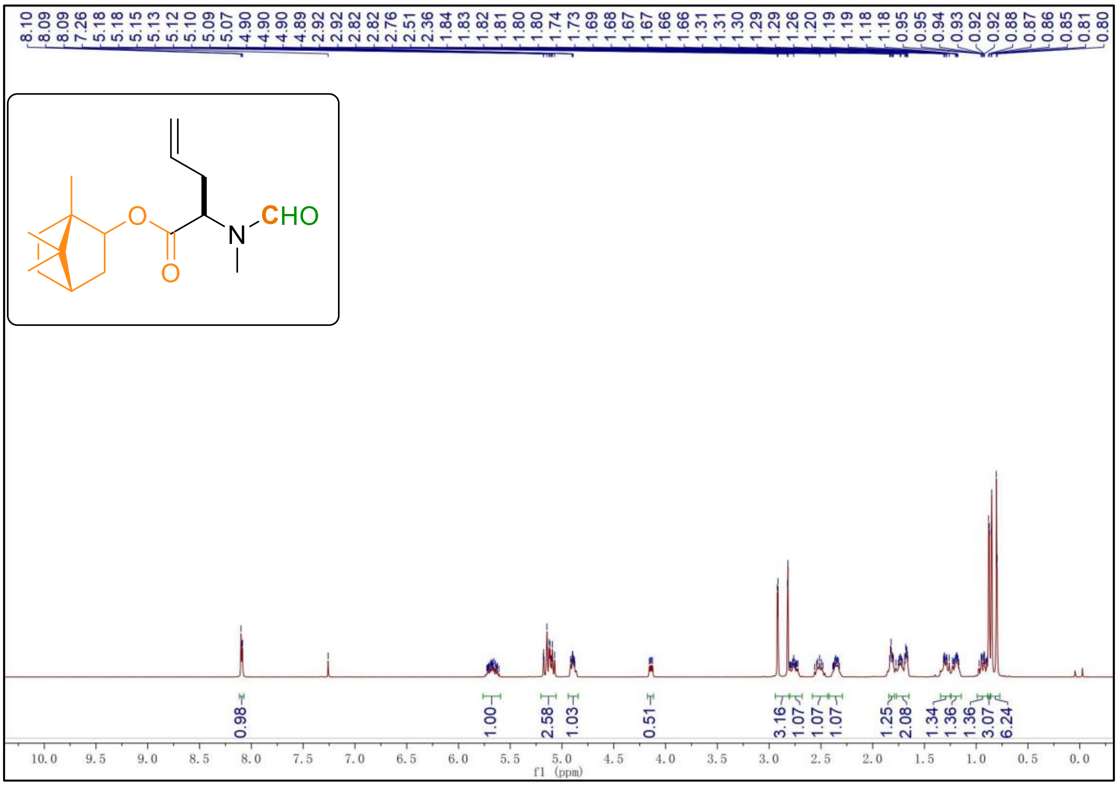

^{13}C NMR (126 MHz, Chloroform-*d*)

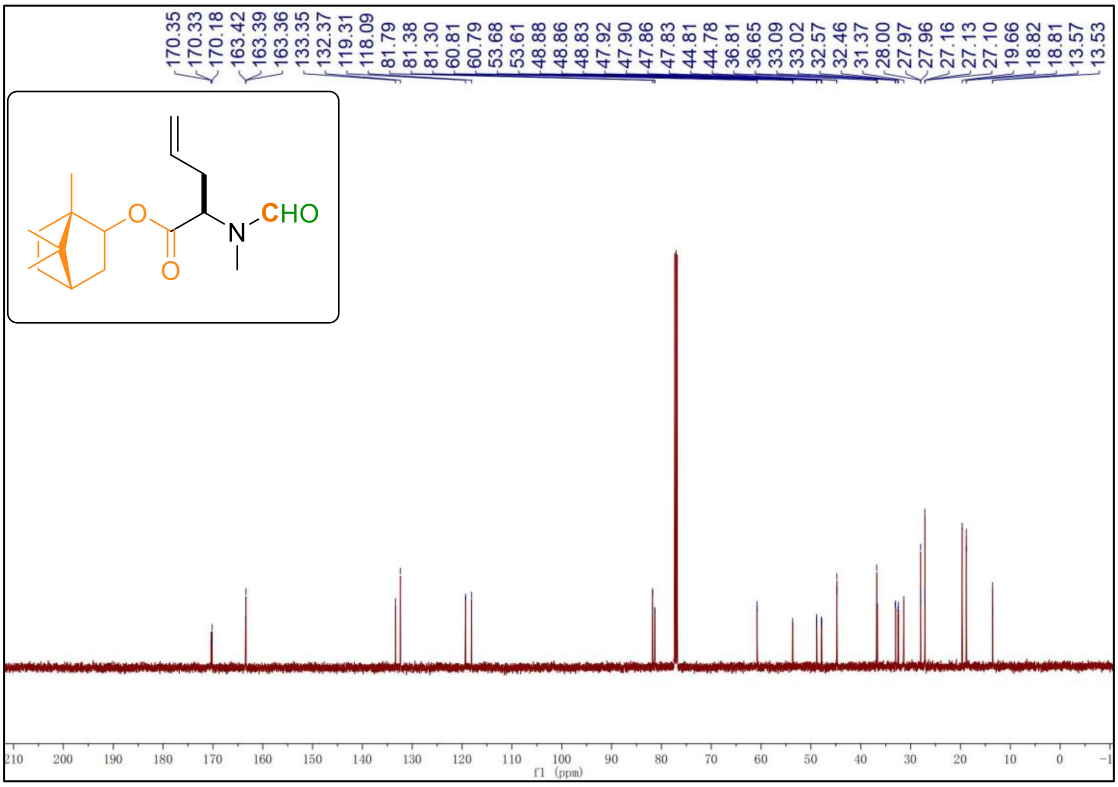

(1R,2S,5R)-2-isopropyl-5-methylcyclohexyl 2-(*N*-methylformamido)pent-4-enoate (11f)

^{1}H NMR (500 MHz, Chloroform-*d*)

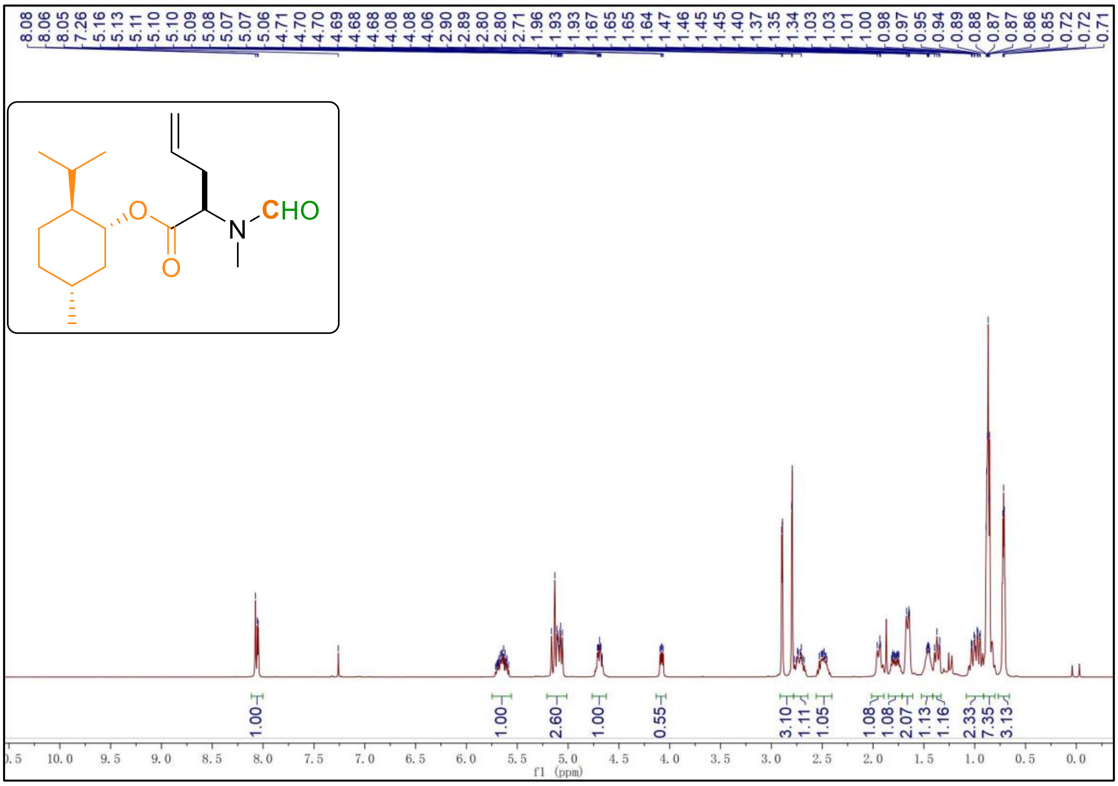

^{13}C NMR (126 MHz, Chloroform-*d*)

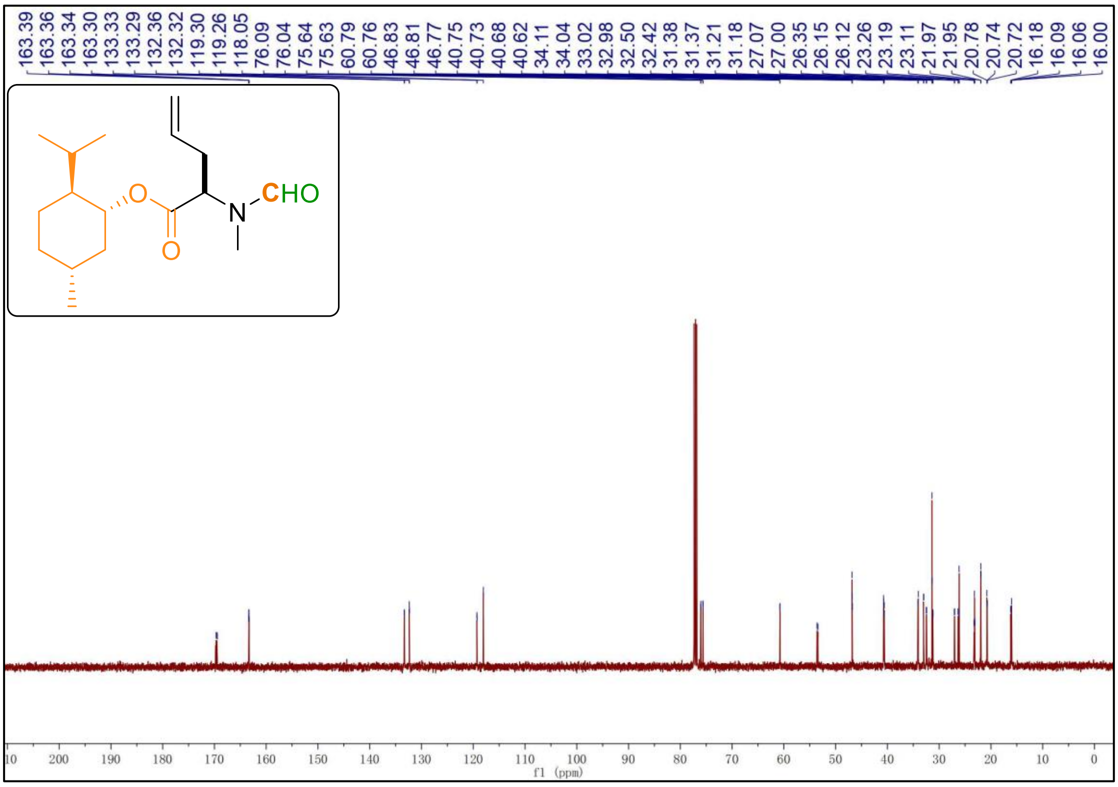

(3aR,5R,6S,6aR)-5-(2,2-dimethyl-1,3-dioxolan-4-yl)-2,2-dimethyltetrahydrofuro[2,3-d][1,3]dioxol-6-yl -2-(*N*-methylformamido)pent-4-enoate (11g)

^{1}H NMR (500 MHz, Chloroform-*d*)

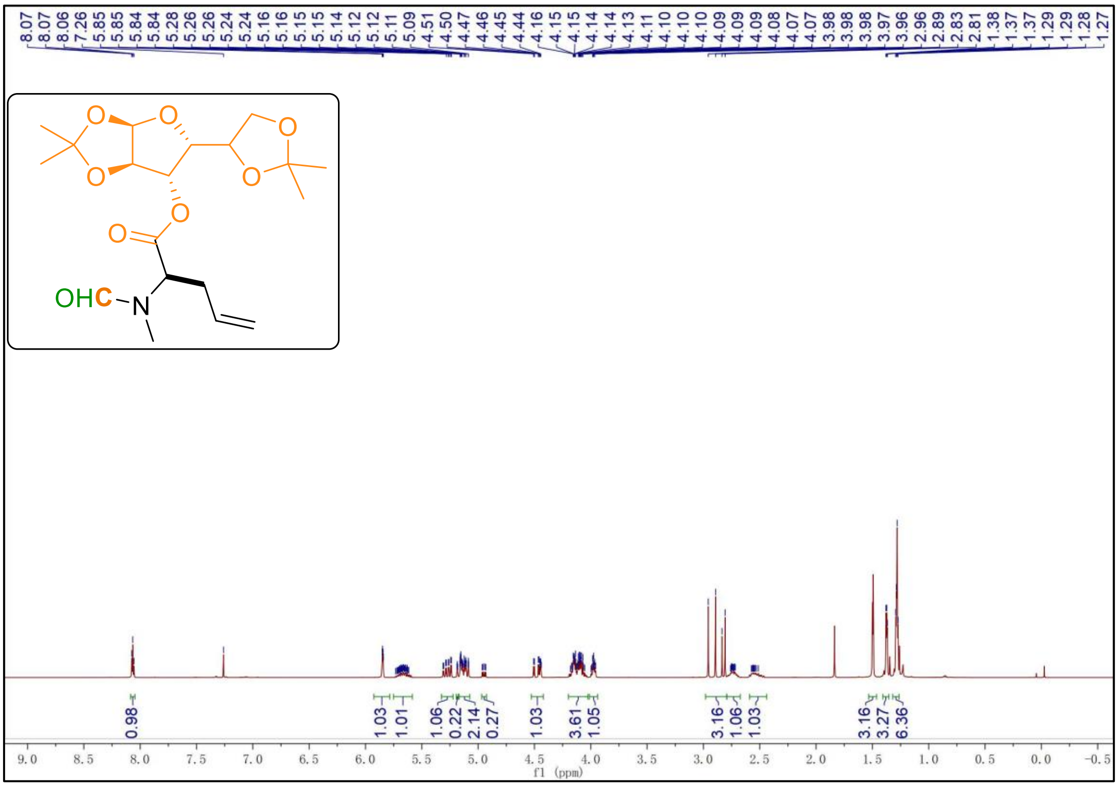

^{13}C NMR (126 MHz, Chloroform-*d*)

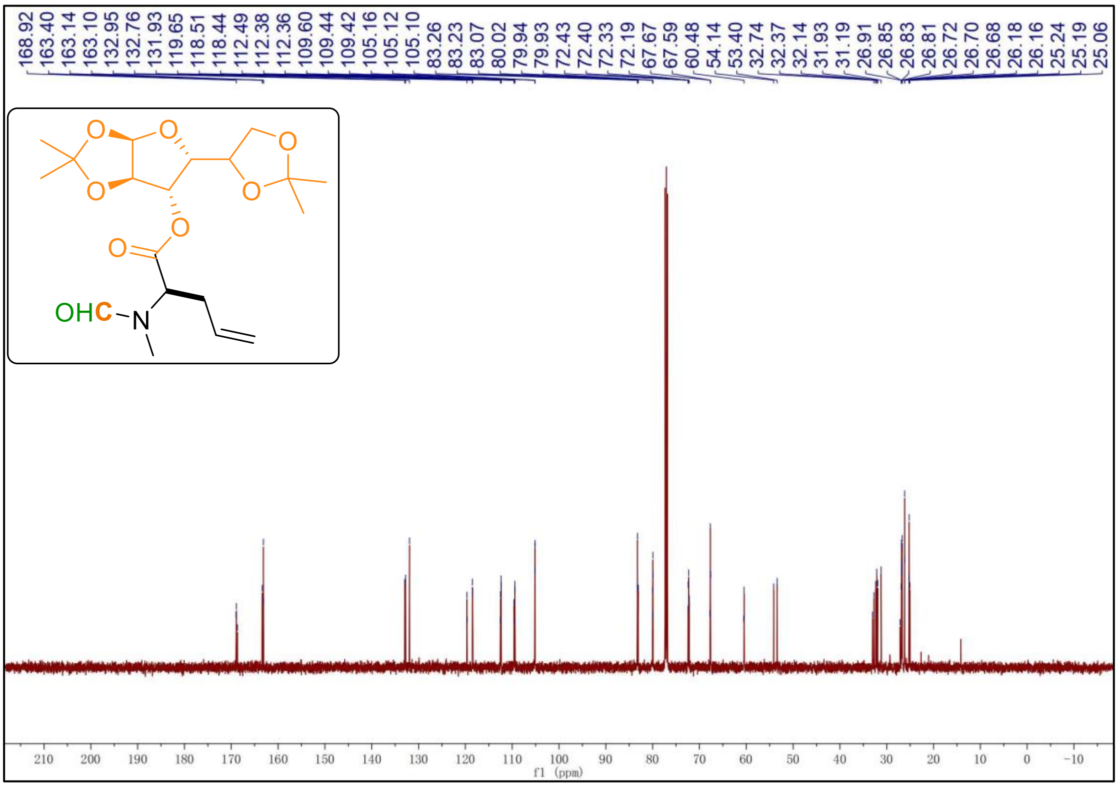

N-methyl-*N*-(5-oxooctan-4-yl)formamide (13)

^{1}H NMR (500 MHz, Chloroform-*d*)

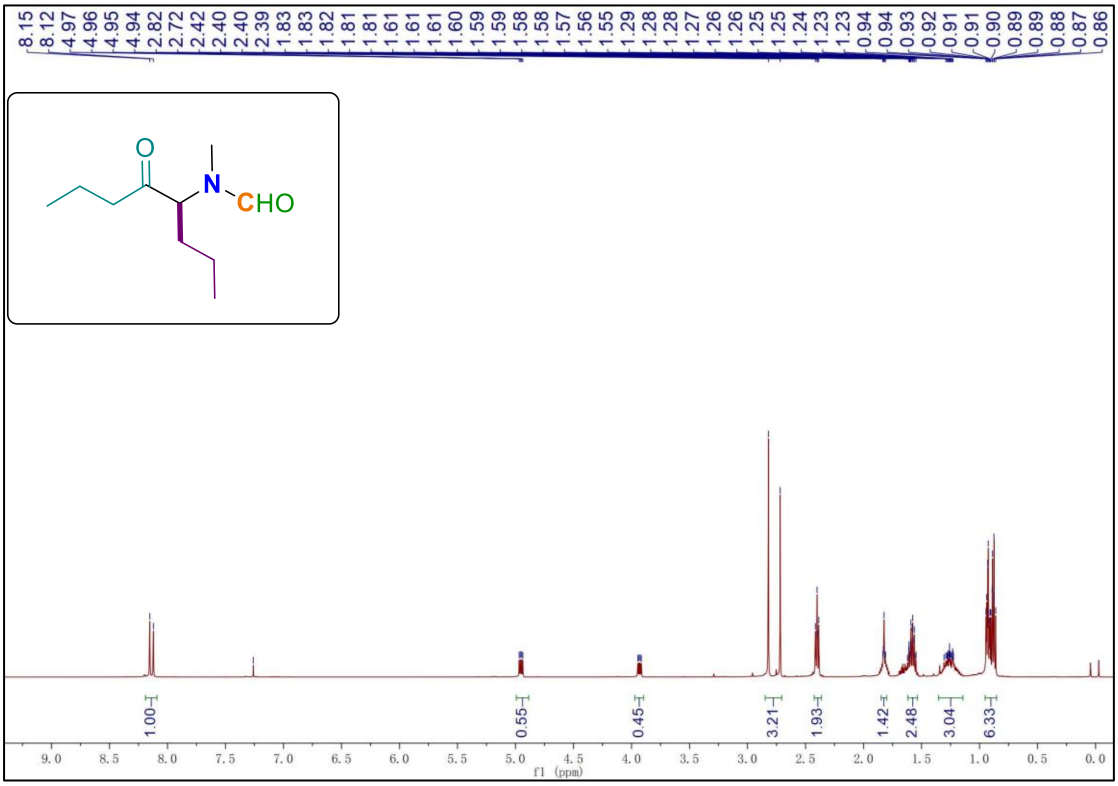

^{13}C NMR (126 MHz, Chloroform-*d*)

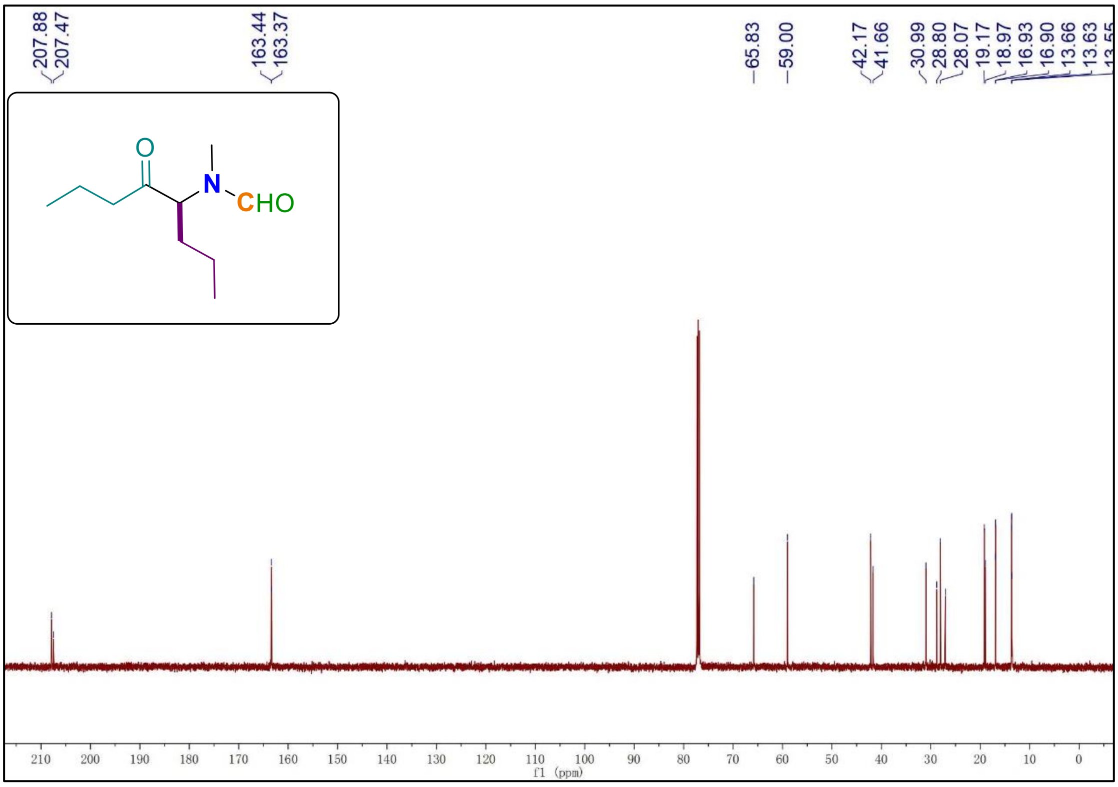

1-cyclopropyl-2-(methylamino)pent-4-en-1-one (14)

^{1}H NMR (500 MHz, Chloroform-*d*)

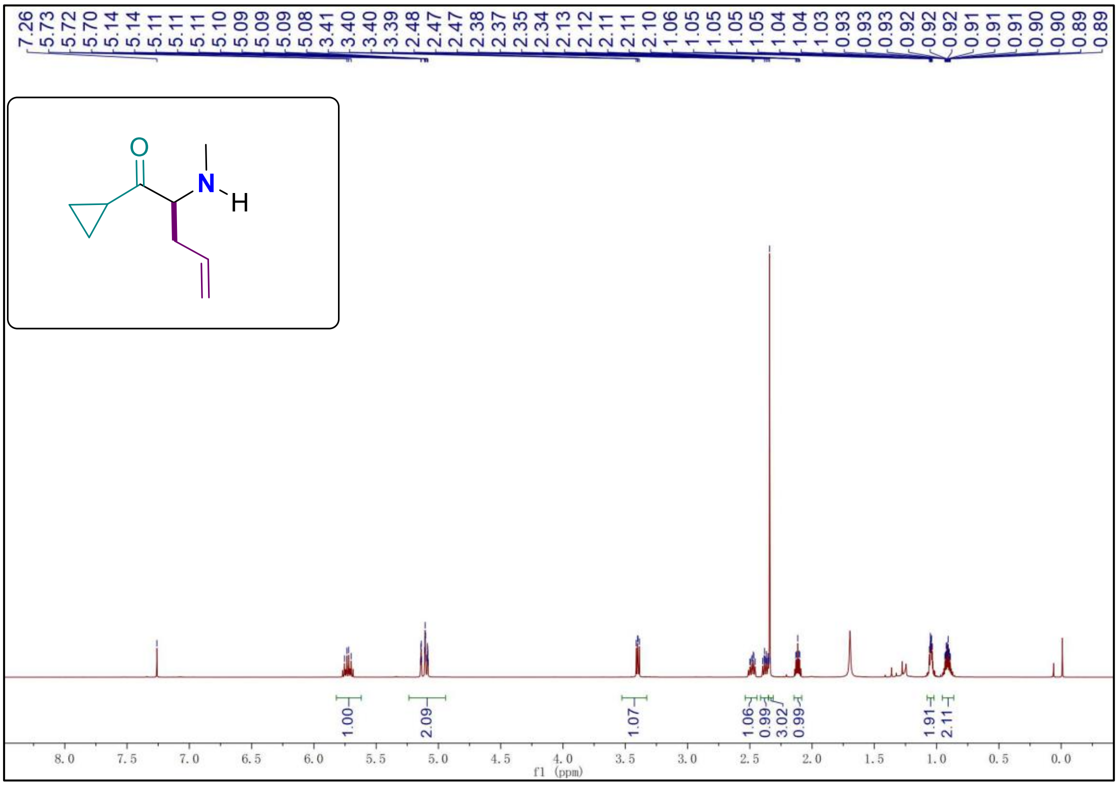

^{13}C NMR (126 MHz, Chloroform-*d*)

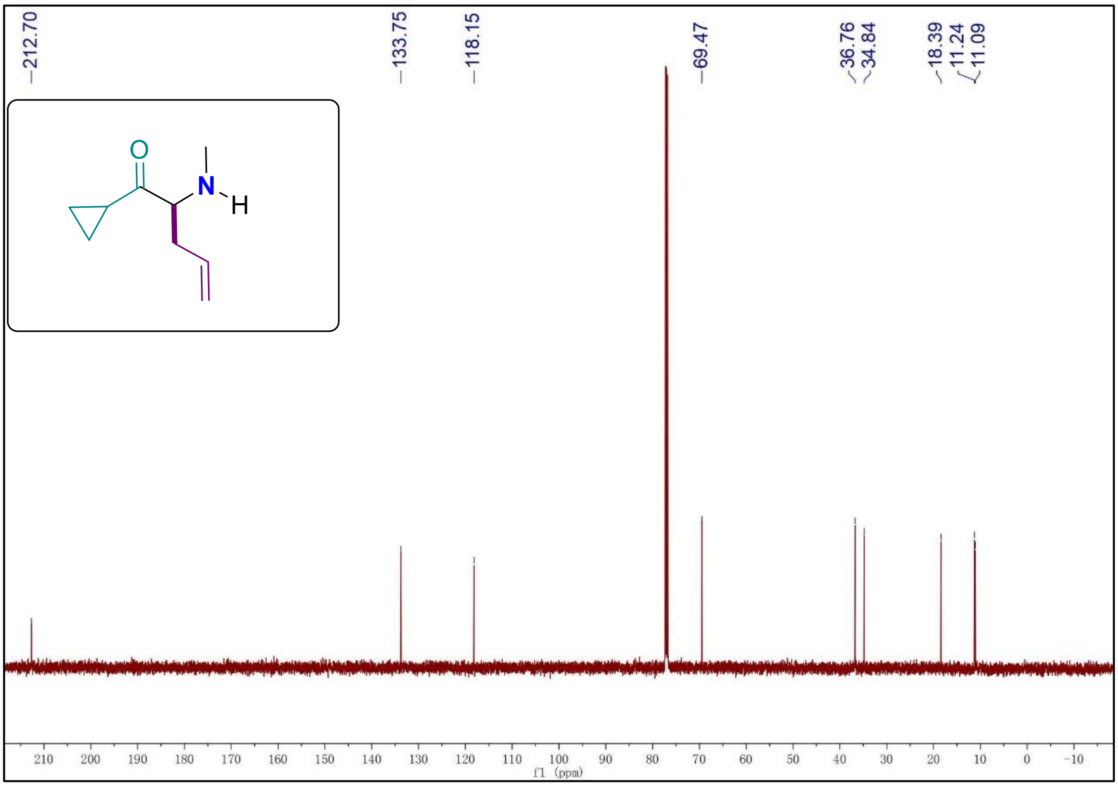

3. References

1. Soheili, A.; Tambar, U K. Tandem catalytic allylic amination and [2,3]-stevens rearrangement of tertiary amines. *J. Am. Chem. Soc*. **133**, 12956-12959 (2011).
2. Tang, S.; Xu, Z.-H.; Liu, T.; Wang, S.-W.; Yu, J.; Liu, J.; Hong, Y.; Chen, S.-L.; He, J.; Li, J.-H. Radical 1,4-Aryl Migration Enabled Remote Cross-Electrophile Coupling of α-Amino-β-Bromo Acid Esters with Aryl Bromides. *Angew. Chem. Int. Ed.,* **60**, 21360-21367 (2021).
3. Zhang, J.; Chen, Z.-x.; Du, T.; Li, B.; Gu, Y.; Tian, S.-K. Aryne-Mediated [2,3]-Sigmatropic Rearrangement of Tertiary Allylic Amines. *Org. Lett.* **18**, 4872-4875 (2016).
4. Zhang, N.; Muench, W.; Nubbemeyer, U. Synthesis and derivatization of substituted (R)- and (S)-C-allylglycines. *Adv. Synth. Catal.,* **346**, 1335-1354 (2004).
5. Xi, S.; Jiang, Y.; Yang, J.; Yang, J.; Miao, D.; Chen, B.; Huang, W.; He, L.; Qiu, H.; and Min Zhang, M. Generation and [2,3]-Sigmatropic Rearrangement of Ammonium Ylides from Cyclopropyl Ketones for Chiral Indolizidines with Bridgehead Quaternary Stereocenters. *Org. Lett.,* **24**, 6957-6961 (2022).
6. Zhang, Z.-H.; Dong, X.-Y.; Du, X.-Y.; Gu, Q.-S.; Li, Z.-L.; Liu, X.-Y., Copper-catalyzed enantioselective Sonogashira-type oxidative cross-coupling of unactivated C(sp^{3})-H bonds with alkynes. *Nature Communications* **10**, 5689 (2019).
7. Li, H.; Qian, H.-F.; Feng, G. Diversity-oriented synthesis of azo disperse dyes with improved fastness properties via employing Ugi four-component reaction. D*ye Pigm*. **165**, 415-420 (2019).
